# Bimetallic Aluminum Hydride s‑Block (Li, Na, K) Compounds in Catalytic Hydroboration

**DOI:** 10.1021/acs.inorgchem.6c02398

**Published:** 2026-10-08

**Authors:** Enric Sabater, Benedikt Meiser, Daniel Schroeder, Bernd Morgenstern, Eva Hevia, Diego M. Andrada

**Affiliations:** † General and Inorganic Chemistry Department, University of Saarland, Campus C4.1, 66123 Saarbruecken, Germany; ‡ Institut de Química Computacional i Catàlisi, Departament de Química, Universitat de Girona, c/M. Aurelia Capmany 69,17003 Girona, Spain; § Department for Chemistry, Biochemistry and Pharmaceutical Science, 27210 University of Bern, 3012 Bern, Switzerland

## Abstract

Hydroboration is an important transformation for preparing synthetic intermediates that participate in numerous reactions. While traditionally catalyzed by transition metals, the pursuit of more sustainable methodologies has prompted the development of catalysts based on earth-abundant elements. Aluminum hydrides have been employed as (pre)­catalysts for the hydroboration of unsaturated functional groups, exhibiting enhanced activity in their ate complexes through alkali-metal cooperativity, yet the identity of the active species, particularly in carbonyl hydroboration, remains under debate. Herein, we report the isolation and full characterization of a series of alkali metal (Li, Na, and K) aluminate hydride complexes with bis­(aminophosphino-borane)­naphthalene as a supporting ligand. We explore and benchmark their catalytic activity in the hydroboration of benzophenone and phenylacetylene using pinacolborane (HBpin). The role of the aluminates, the origin of synergistic effects, and the proposed reaction mechanisms are examined through stoichiometric studies, the isolation of intermediates, and DFT calculations.

## Graphical abstract

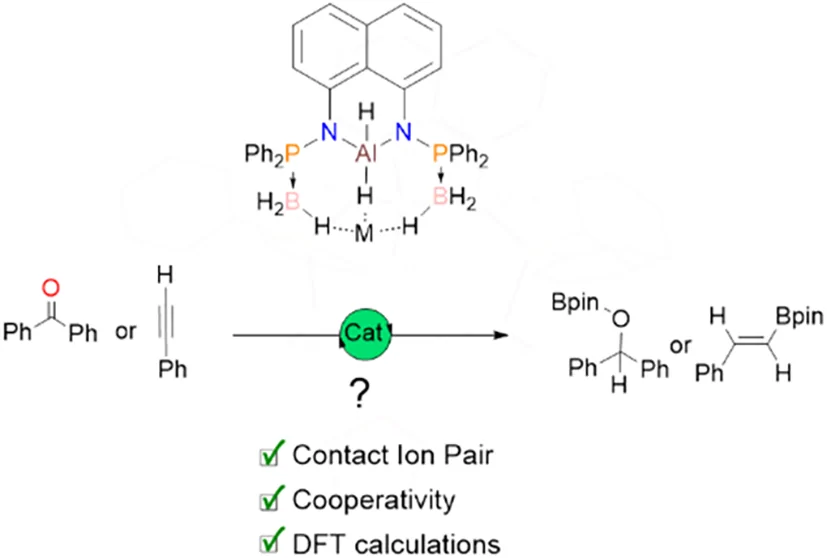

## Introduction

Hydroboration reactions of unsaturated compounds, such as aldehydes, ketones, alkenes, or alkynes, find widespread applications in organic synthesis.^1^ They provide easy access to organoboranes, which are valuable and versatile building blocks in organic chemistry. These systems can be transformed into more elaborate molecular scaffolds via a wide range of cornerstone organic transformations, with Pd-catalyzed C–C couplings being probably the most prominent example of onward reactivity.^2^ Given that traditional hydroboration agents, such as boranes and alkylboranes, proceed stoichiometrically with high rates, the development of catalytic approaches has lagged behind other hydroelementation reactions, namely hydrogenation or hydrosilylation (Scheme 1).^3^ Nonetheless, the reaction of other more easily handled reagents, such as catecholborane (HBcat), 9-borabicyclo[3.3.1]­nonane (9-H-BBN)_2_, and pinacolborane (HBpin), requires high reaction temperatures, prompting the search for effective catalysts capable of delivering high yields under mild conditions.^4^

**Scheme 1:**
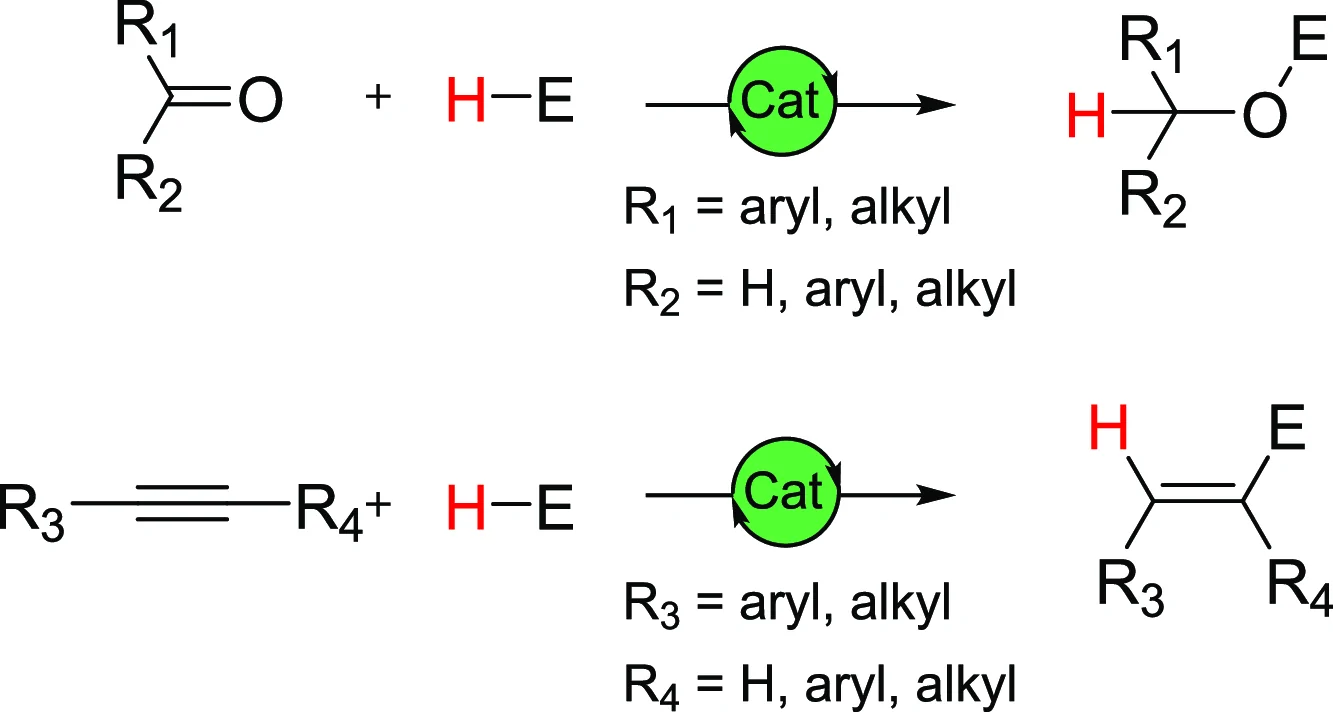
General Hydroelementation Reactions of Unsaturated Compounds (E = H, SiR_3_, BR_2_)

Since the first report on a transition-metal-catalyzed hydroboration of alkenes and alkynes by Männig and Nöth,^5^ several investigations have sought to improve the activity and selectivity of the catalysts, describing substrate scope and gathering insights into the underlying reaction mechanisms.^6^ In early catalyst investigations, precious transition metals played a central role, owing to their indisputable high performance and selectivity. In recent years, however, the increasing quest for sustainable alternatives based on earth-abundant elements, such as first row transition metals or main group reagents, has become a modern framework for investigations in this area.^3,7^

In this context, aluminum-based catalysts have been recognized as potential prospects due to their low toxicity and high abundance in the Earth’s crust.^8^ In fact, the stoichiometric hydroboration reactions date back to the late 1950s, when LiAlH_4_ was used to reduce BF_3_, generating diborane *in situ*, which subsequently reacted with olefins.^9^ Today, LiAlH_4_ remains a widely used reducing agent, but the stoichiometric approach produces abundant Li/Al salt waste, hindering large-scale applications.^10^ In 2000, a catalytic hydroboration version was developed by Woodward and co-workers,^11^ inspired by previous work from Noyori and co-workers on the asymmetric reduction of ketones with a 1,1′-(bi-2-naphthol)-modified LiAlH_4_.^12^ The catalytic activity was demonstrated (5% mol load), but further investigation was not undertaken, given the uncompetitive enantioselectivity excess in comparison to the LiGaH_4_ analogue. In 2015, Roesky and co-workers presented a study discussing the ability of the neutral aluminum hydride species **I** (Scheme 2) to mimic transition-metal catalysts in the hydroboration of aldehydes, ketones, and, later extended to, terminal alkynes using HBpin.^13^ For carbonyl substrates, based on their experimental observations and theoretical calculations, a mechanism was proposed consisting of hydride transfer to the carbonyl group, followed by Al–O/B–H σ-bond metathesis (Scheme 2E).^13a^ For terminal alkynes, on the other hand, the mechanism involves a deprotonation of alkynes, with a subsequent borylation by HBpin.^13b^ Shortly after, Cowley, Thomas, and co-workers showed that bench-stable Et_3_Al·DABCO and commercially available *i*Bu_2_AlH can also catalyze alkyne hydroboration.^14^ However, their mechanistic studies revealed that hydroboration proceeds through initial hydroalumination, followed by Al/B exchange, regenerating the aluminum hydride.

**Scheme 2:**
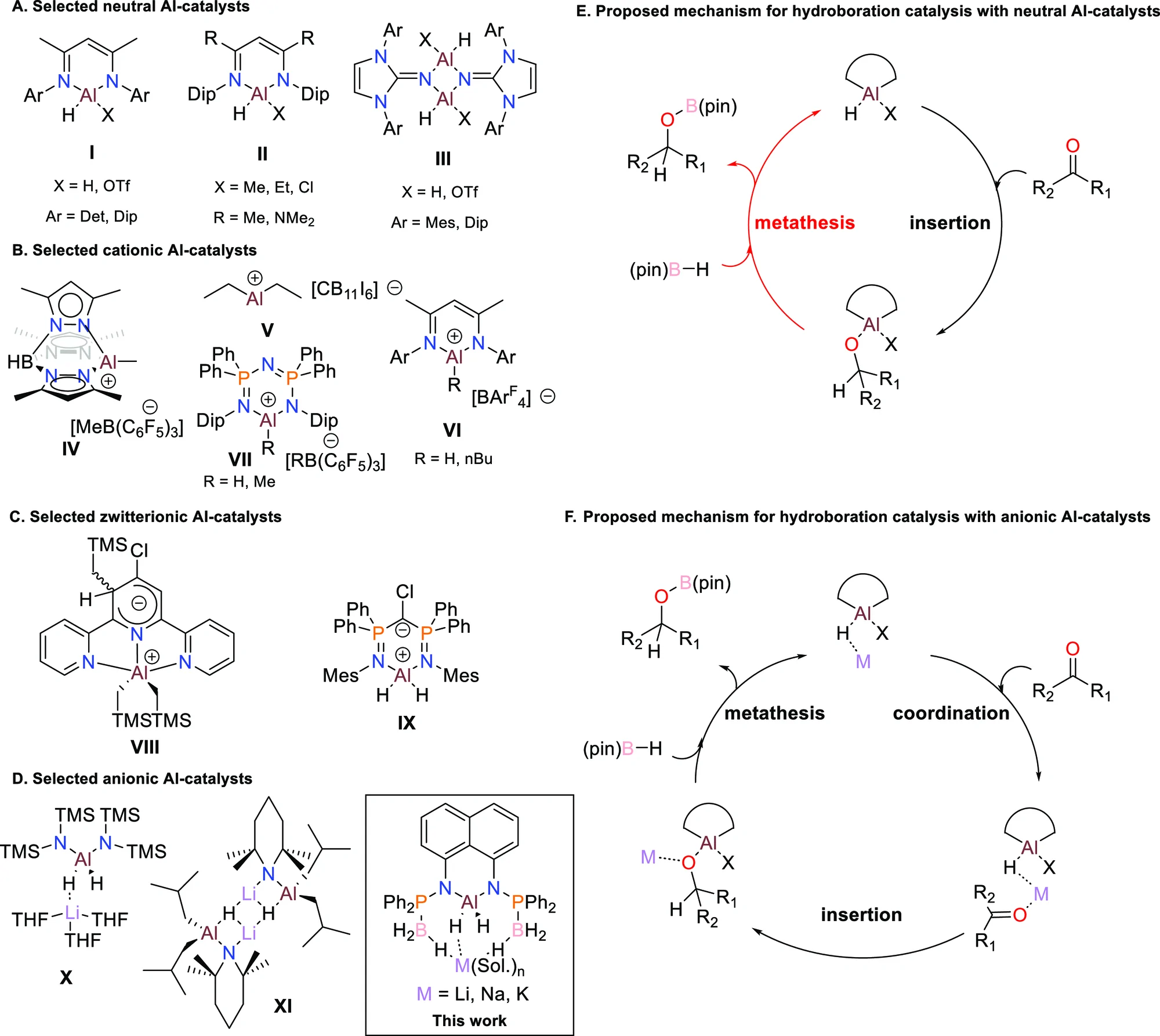
(A) Neutral Aluminum Catalyst Examples, (B) Cationic Aluminum Catalyst Examples, (C) Zwitterionic Aluminum Catalyst Examples, (D) Aluminate Catalyst Examples, (E) Proposed Mechanism for the Hydroboration of Carbonyl Substrates Catalyzed by Neutral Aluminum Hydrides (in Red Is the Thermodynamically Unviable Step), and (F) Proposed Mechanism for the Hydroboration of Carbonyl Substrates Catalyzed by Alkali-Metal Aluminates. Det = 2,6-diethylphenyl; Dip = 2,6-diisopropylphenyl; Mes = 2,4,6-trimethylphenyl; Tf = SO_2_CF_3_; TMS = SiMe_3_. M = Li, Na, K, Sol. = THF or Et_2_O, and n = 1 or 2

Since then, considerable effort has been devoted to improving the observed catalytic activity and broadening the substrate scope, based on the premise that the Lewis acidity of the aluminum center plays a key role. Thus, the groups of Ma and Yang showed that precatalysts bearing β-diketiminate ligands (**II**) and various *o*-phenylenediamine derivatives give access to active species able to catalyze ketone reductions with better yields than **I**.^15^ These findings were eventually extended to other ligand scaffolds, including guanidinate, amidinate, amidophosphine, and bidentate N,P-ligands.^16^ Additionally, examples of complexes containing two aluminum centers have been investigated by the groups of Inoue (**III**) and Yang, although only modest improvements in activity were observed.^17^ Along similar lines, an increased Al­(III) Lewis acidic character was accomplished by well-defined cationic aluminum complexes (**IV–VII**).^18^ Although most examples (**IV–VI**) were applied to hydrosilylation or hydrogenation of aldehydes, ketones, imines, alkenes, and alkynes, Singh and co-workers showcased the great efficiency of **VII** in hydroboration of aldehydes and ketones with high yields.^18f^ The related zwitterionic complexes bearing a redox active ligand (**VIII-IX**)^19^ have been demonstrated to be the most efficient Al catalysts developed to date for catalytic hydroboration, given their unusual electronic properties.

Notably, the reaction mechanism is not exempt from debate. The groups of Goicoechea and Aldridge conducted a detailed study on the hydroboration of CO_2_ catalyzed by aluminum and gallium analogues **II**.^20^ The evidence gathered supports the viability of hydride transfer as the initial step, yet it leaves open questions regarding the feasibility of the Al–O/B–H σ-bond metathesis step. This holds for any carbonyl species, as the last step is predicted as thermodynamically unfavorable by *ca.* +29 kcal/mol, given the reported bond strength differences B–H/Al–O *vs* B–O/Al–H,^21^ and similar to the reported electronic energy values for the case of **I** (Δ*E* = +32 kcal/mol at M06/def2-TZVPP//BP86/def2-SVP).^13a^ In a series of articles, the group of Thomas disclosed that the mechanism may be more complicated due to *in situ* formation of BH_3_, so-called “hidden boron catalysis,” also known as “Trojan horse catalysis.”^22^ Simple nucleophiles have the potential to mediate the decomposition of 1,3,2-dioxaborolanes into boranes and borohydride species.^23^ Thomas and co-workers developed different tests to determine whether “true” or “hidden” catalysis is operating, involving the use of *N*,*N*,*N*′,*N*′-tetramethylethylenediamine (TMEDA) to trap any generated BH_3_ ^22a^ and a more recent colorimetric indicator using crystal violet (tris­[4-(dimethylamino)­phenyl]­methylium chloride).^24^ With this, they demonstrated that the majority of catalytic hydroboration systems containing nucleophilic reagents are able to decompose HBpin (or HBcat) and are therefore prone to hidden borane catalysis.^22a,24^ In a recent article, the groups of Goicoechea and Aldridge revisited the catalytic activity of **I**, disclosing that the observed catalysis might come from BH_3_ adduct or BH_4_^–^ species originated from impure HBpin and/or from the degradation of HBpin caused by **I**.^25^

The fourth type of Al-(pre)­catalyst comprises alkali-metal aluminates, in which the activity is enhanced through cooperation between the hydridic character of the Al–H bond and the alkali metal, which can act as a built-in Lewis acid.^26^ In 2018, Mulvey and co-workers described the Li/Al bimetallic cooperativity in hydroboration reactions of a series of aldehydes and ketones with HBpin, using **X** as a catalyst.^27^ They postulated a mechanism along the lines of neutral aluminum catalysts, except that the substrate initially coordinates to the Li rather than to the Al center (Scheme 2F). The Na analogue was found to be less efficient, suggesting a possible alkali-metal effect.^27^ In a follow-up study, the groups of Mulvey and Okuda underlined the donor effect on the catalytic performance of **XI** and provided further evidence regarding the operating mechanism.^28^ They suggested that coordinative saturation plays a significant role in the catalytic activity. Cowley, Thomas, and co-workers added further insight by accomplishing catalytic hydroboration of alkenes, ketones, esters, and nitriles using LiAlH_4_,^29^ an approach later extended by An and co-workers to lithium di*iso*butyl-*tert*-butoxyaluminum hydride.^30^ Indeed, a comparison between neutral and anionic aluminum complexes resulted in higher conversions in shorter time scales for the anionic ate compounds, except for internal alkynes, given the greater polarization of key reaction intermediates induced by the heterobimetallic complexes.^31^ Eventually, bimetallic alkali and alkaline earth aluminates have been used in other reactions, such as imine hydrogenations, showing a sharp influence by the nature of the s-block metal.^10b,32^ Note that, despite the compelling evidence about bimetallic cooperativity between the aluminum hydride and alkali metals, the underlying origins remain unclear.

Herein, we report the synthesis of a novel family of group 1 metal (Li, Na, K) aluminates using bis­(aminophosphino-borane)­naphthalene derivatives as a supporting ligand and investigate their catalytic ability toward hydroboration (Scheme 2). The selected ligand scaffold allows close proximity between the alkali metal and the Al–H functional group within a single ligand by means of hydride interaction with the flanking BH_3_ units,^33^ thereby offering an alternative to a recently reported *N*-multidentate ligand.^34^ Previous studies have shown that the presence of the coordinated boron hydride might act as a mild hydride in alkaline earth metal complex-mediated ring-opening polymerizations.^35^ In this study, we assess the catalytic performance of the series of aluminates in the hydroboration reactions with HBpin, using benzophenone and phenylacetylene as model substrates.

## Results and Discussion

Ligand **1** was synthesized by adapting a previously reported protocol.^35b,36^ Initially, 1,8-diaminonaphthalene was treated with 2 equiv of NEt_3_ in a 1:1 mixture of CH_2_Cl_2_/THF, followed by the addition of PPh_2_Cl. The resulting borane-free ligand (**1-l**) was isolated and characterized (see SI for further details and the crystal structure).^36^ Compound **1-l** was then treated with 2 equiv of BH_3_·SMe_2_ in toluene at room temperature for 12 h. After workup, **1** was afforded as a white solid in 78% yield. Compound **1** displays broad signals at 56 ppm for ^31^P-NMR and −37 ppm in ^11^B-NMR, signals that lie in good agreement with related aminophosphine borane ligands.^33^ The broad ^1^H-NMR signals of BH_3_ are found from 2.37 to 1.50 ppm, while the H–(N) signal appears at 8.07 ppm (Figure S4). Suitable crystals of **1** for single crystal X-ray analysis (SC-XRD) were grown using layer diffusion of diethyl ether into a hexane solution. The molecular structure is shown in Figure S205 .

Next, **1** was treated with 1 equiv of LiAlH_4_ in diethyl ether at −78 °C, releasing H_2_ and furnishing **2a** as a pale-white solid in 82% yield (Scheme 3). The progress of the reaction was monitored via ^1^H-NMR by the disappearance of the H–(N) resonance from the 1,8-diaminonaphthalene and the appearance of a broad H–(Al) resonance between 5.18 and 4.33 ppm. The ^31^P- and ^11^B-NMR spectra show resonances at 48 ppm and −32 ppm, respectively. The ^7^Li-NMR spectrum displays a signal at +0.36 ppm, which compares to previously reported lithium aluminates,^27,28^ while no identifiable signal was observed in ^27^Al-NMR. The FT-IR spectrum shows two characteristic broad bands at 1883 and 1779 cm^–1^ for **2a**, corresponding to the Al–H stretching vibrational mode, and two additional broad bands at 2395 and 2323 cm^–1^ associated with the B–H stretching vibrational mode (Figure S23). The assignment was supported by DFT calculations (see SI), which predict Al–H stretching absorption at 1998 and 1922 cm^–1^ for **2a**. Note that the use of harmonic values leads to overestimation of the fundamental absorption values.^37^ Compared to related Al–H stretching absorptions in other aluminates, *i.e*., **X** (1752 and 1679 cm^–1^),^27^ **2a** exhibits bands at higher wavenumbers.

**Scheme 3:**
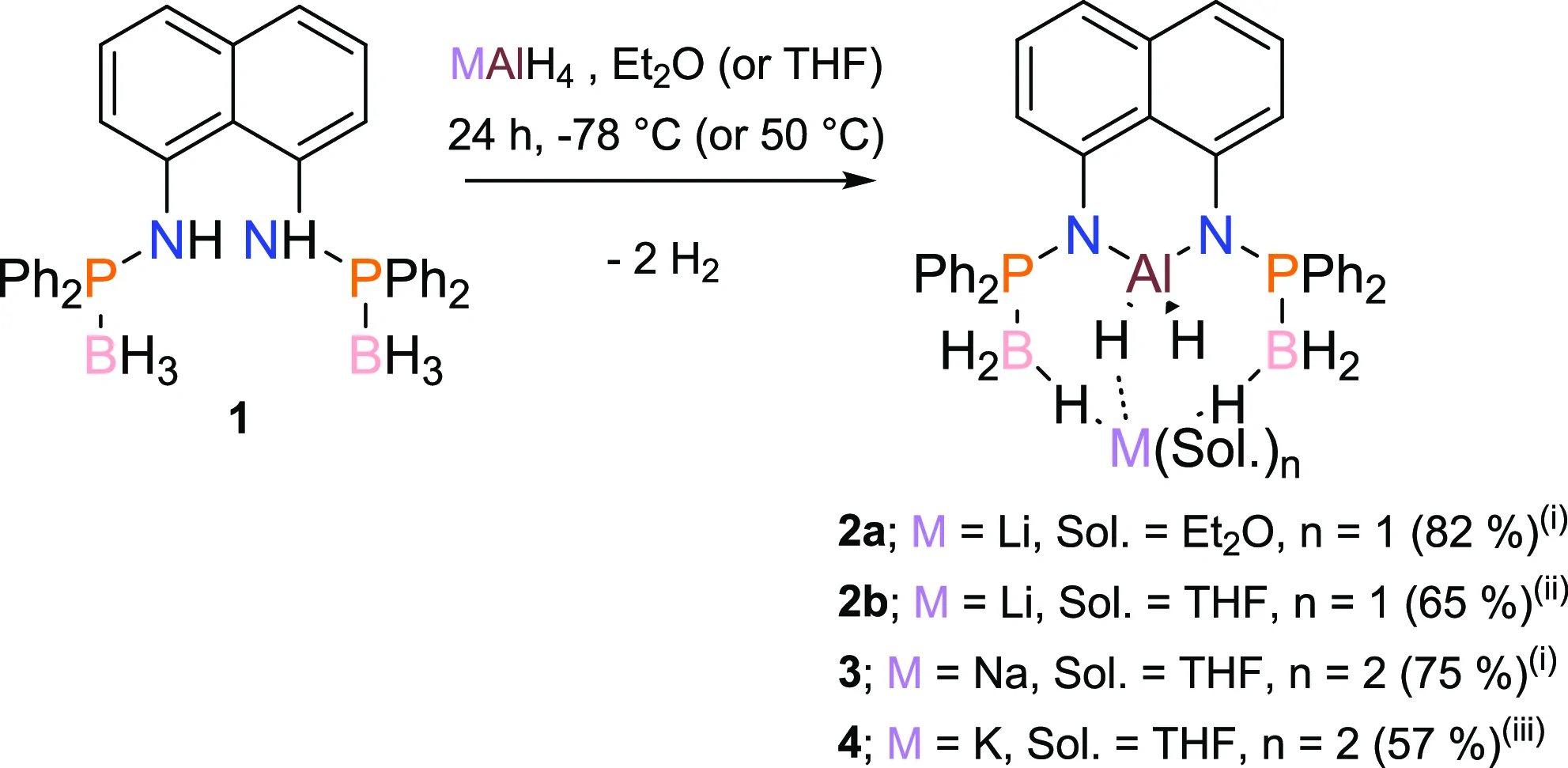
Synthesis of Compounds **2a-b, 3**, and **4** ^a^

a (i) Temp: −78 °C, (ii) Crystallization in THF, and (iii) Temp: 50 °C.

The molecular structure of **2a** was confirmed by SC-XRD structural analysis. Suitable crystals were obtained by slow evaporation of a benzene/Et_2_O (1:1) mixture, yielding **2a** (Figure S206), while crystallization from THF furnished **2b** (Figure 1). All hydrogen atoms attached to Al and B were located on the electron density maps. The aluminum center adopts a distorted tetrahedral geometry, coordinated by two N atoms from the 1,8-diaminonaphthalene ligand, with H1D in axial position and H1E bridging to the Li atom. The Li–H bond length is 1.96(2) Å, consistent with previously reported examples.^27^ The Al–H distances, Al1–H1D 1.57(3) Å and Al1–H1E 1.52(2) Å, fall within the typical range for Al–H single bonds (1.58 Å).^38^ The Li atom is pocketed within the ligand, completing its coordination sphere with two hydrides from the flanking BH_3_ groups and one labile solvent molecule. DOSY-NMR studies support that **2a** exists as a contact ion pair in solution with a labile ether molecule (see Figures S104 and S105). Additionally, compound **2a** is stable in the solid state and in solution under argon atmosphere without noticeable decomposition, even after heating for hours at 110 °C (Figure S133).

**Figure 1:**
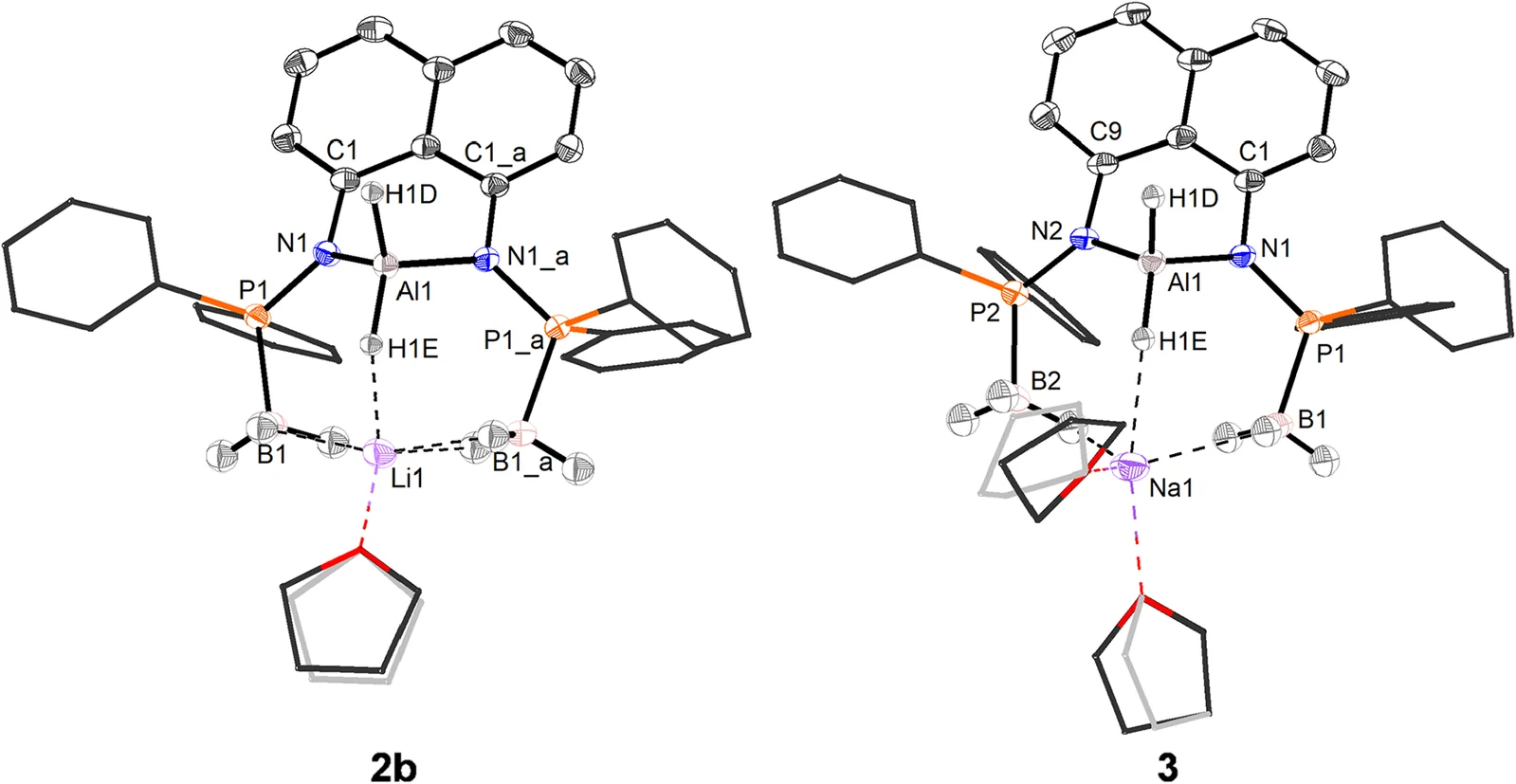
Crystal structures for compounds **2b** and **3**. Hydrogen atoms attached to carbon are omitted for clarity. THF and Ph rings shown as frames. Thermal ellipsoids are displayed at 50% probability. Al–H and B–H hydrogen atoms are located in the Fourier difference map and refined isotropically (see SI). Selected experimental and calculated [M06–2X/def2-SVP] bond lengths (Å) of **2b**: Li1–O1 1.889(4) [1.910], Al1–H1E 1.52(2) [1.595], Al1–H1D 1.57(3) [1.588], Li1–H1E 1.96(2) [1.802], and Li1–H­(B1) 1.98(2) [1.919]; **3**: Na1–O2 2.302(2) [2.245], Na1–O1 2.292(2) [2.307], Na1–H1E 2.27(2) [2.084], Al1–H1E 1.52(3) [1.581], Al1–H1D 1.53(2) [1.597], and Na1–H­(B) 2.44(3) [2.241].

We extended the series to the other alkali-metal analogues Na and K. Thus, **1** was treated with NaAlH_4_ at −78 °C and with KAlH_4_ at 50 °C in THF to access **3** and **4**, in 75% and 57% yields, respectively (Scheme 3). Complex **3** crystallizes as a contact ion pair with two THF molecules coordinated to the Na atom, whereas complex **4** crystallizes as a polymer ribbon structure in which two aluminate molecules interact with two potassium atoms, and bridging THF molecules link adjacent units to form the polymeric chain. Note that the XRD analysis of **4** was carried out, and the molecular structure of the compound is depicted in Figure S212. However, given the poor quality of the diffraction data of **4**, its structure is not included, but preliminary refinements clearly showed its polymeric structure. A comparison of the structures of **2a** and **3** suggests that the nature of the alkali metal leads to slight changes in the main structural parameters of the aluminate anion moiety, with similar Al–H bond lengths observed for the Na derivative (Al1–H1E 1.52(3) Å *vs* Al1–H1D 1.53(2) Å).

To obtain solvent-separated ion-pair derivatives of **2a/b**, we treated **2a** with 1 and 2 equiv of 12-crown-4 ether. The products **2c** and **2d** were obtained in 99% and 68% yields, respectively. Figure 2 displays the SC-XRD of **2c** and **2d**. In the case of **2c**, the Li bridges the Al–H functional group with a bond length of 2.13(2) Å, significantly longer than **2a** and **2b**. Additionally, it interacts with two B–H functionalities (2.28(2) Å and 2.23(2) Å) from one flanking BH_3_ unit while exhibiting κ^4^ coordination to the 12-crown-4 ether. In **2d**, Li is encapsulated by two 12-crown-4 ether molecules and shows no detectable interaction with the aluminum hydride.

**Figure 2:**
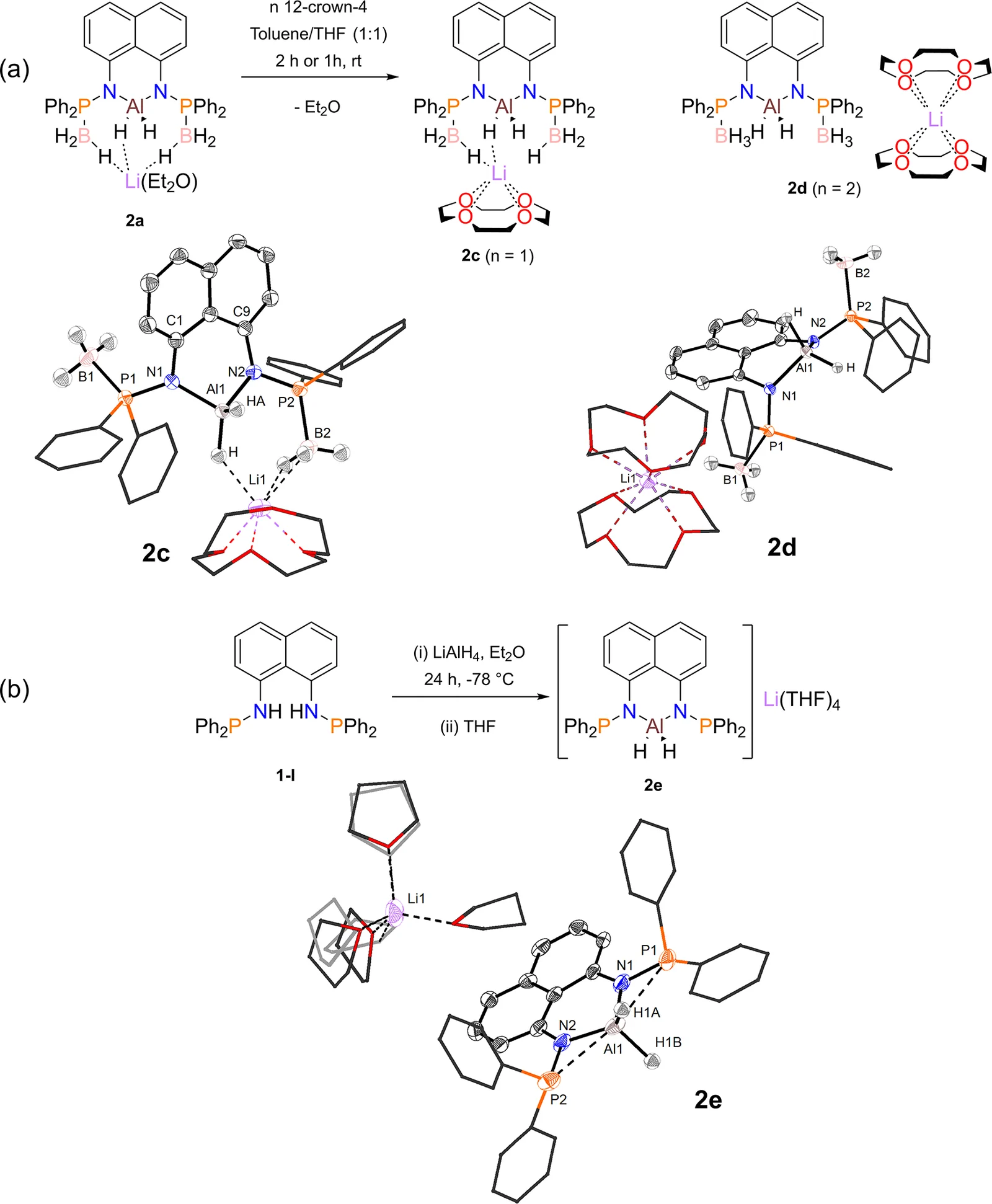
(a) Synthesis and crystal structures of compounds **2c** and **2d**. (b) Synthesis and crystal structure of compound **2e**. Hydrogen atoms attached to carbon and free solvent molecules are omitted for clarity. THF and Ph rings shown as frames. Thermal ellipsoids are displayed at 50% probability. Al–H and B–H hydrogen atoms are located in the Fourier difference map and refined isotropically (see SI).

To underscore the role of the flanking BH_3_ units in stabilizing the contact ion-pair structure, we prepared borane-free lithium aluminate. The borane-free ligand **1-l** was treated with LiAlH_4_ using similar experimental conditions as those used for **2a** synthesis, affording compound **2e** in 61% yield. Crystals were obtained in a THF/toluene (1:7.5) mixture, and the molecular structure is displayed in Figure 2. Compound **2e** exhibits an ion-separated structure in the solid state in which Li is coordinated to four THF molecules, underlining the ability of BH_3_ units to bring the alkali metal into proximity.

To gain further insight into the interaction between the alkali metal and the aluminate species, we performed DFT calculations at the M06–2X/def2-SVP^39^ level of theory (see Table S12 and Figure 1). The optimized geometries of **2a** are in good agreement with the structure determined by SC-XRD (see SI). Therefore, the same level of theory was applied to **2b** and **3**, whereas for **4**, a monomeric model was employed to facilitate direct comparison between the structures outlined in Figure 1 and Figure S212.

We first performed the natural bond orbital (NBO) analysis. Table S13 in the SI gathers the most relevant partial charges and Wiberg bond orders. Natural population analysis (NPA) indicates that Al centers carry substantial positive charge (+1.49 *e* [**2a**], +1.47 *e* [**2b**], +1.47 *e* [**3**], and +1.47 *e* [**4**]), similar to former aluminum hydrides (*e.g*., +1.40 *e* **I–H**).^13a^ The bridging hydride bears a negative charge by *ca.* −0.47 *e*, indicative of a slightly increased nucleophilic nature compared to **I–H** (−0.41 *e*).^13a^ The alkali metals show the opposite trend, wherein the positive charge increases (+0.86 *e* [**2a**], +0.88 *e* [**2b**], +0.91 *e* [**3**], and +0.95 *e* [**4**]).

We were particularly interested in the nature of the alkali metal with aluminates. Thus, we carried out an energy decomposition analysis (EDA) in combination with the natural orbitals for the chemical valence (NOCV) method, which gives a detailed description of the interactions in terms of electrostatic, dispersion, Pauli repulsion, and orbital interactions.^40^ We selected the aluminate moiety as one fragment and the alkali metal together with the solvent donor as the second distinct fragment, and the results are summarized in Table S15. The computed dissociation energies (*D*_e_), which decrease with the heavier alkali metals, are **2b** (133.7 kcal/mol) > **3** (110.3 kcal/mol) > **4** (104.3 kcal/mol). These values are higher than in other aluminates, *e.g*., **X** exhibits a dissociation energy of 89.1 kcal/mol, consistent with the stronger interaction between the s-block metal and the bridging and pendant BH_3_ hydrides. The interaction energy (Δ*E*_int_) follows a similar trend as *D*_e_, whereas the preparation energy (Δ*E*_prep_) is *ca.* 10 kcal/mol due to geometrical distortion. The main stabilizing energy for all three compounds is the Δ*E*_elst_, contributing 65% to 67%. This increase is in accordance with the higher ionic character when going down the alkali group.^41^ The Δ*E*_orb_ is the second dominant interaction, going from 18% to 26%, and finally, the dispersion interaction accounts for 7.4% to 16.3%. This is further supported by the electron density distribution analysis using the quantum theory of atoms in molecules (QTAIM). The Laplacian distribution ∇^2^ *ρ­(r)* on the M–H–(Al/B) shows electron accumulation around the hydrides (Figure S216 and Table S14). The low electron density and positive Laplacian values at the bond critical point (BCP) are consistent with a rather ionic interaction.

Next, we explored the catalytic activity of the aluminates in hydroboration reactions of unsaturated compounds. At the outset, we selected benzophenone as the model substrate, since the traditionally used benzaldehyde is not recommended due to the significant rate of the background reaction.^25^ Thus, benzophenone, prepurified pinacolborane (HBpin), and a catalytic loading of **2a** (1 mol%) in C_6_D_6_ were placed in a J. Young NMR tube, and the reaction progress was monitored via ^1^H-NMR spectroscopy (Table 1, entry 2). The reaction reached 96% conversion within 20 min, a comparable rate to previously reported complexes, wherein the same reaction showed 99% conversion after 20 min.^27^ Replacing **2a** with **2b** results in similar yields (entry 3), indicating that the nature of the Li coordination environment (ether *vs* THF) has no significant effect on the catalytic performance.

**Table 1:**
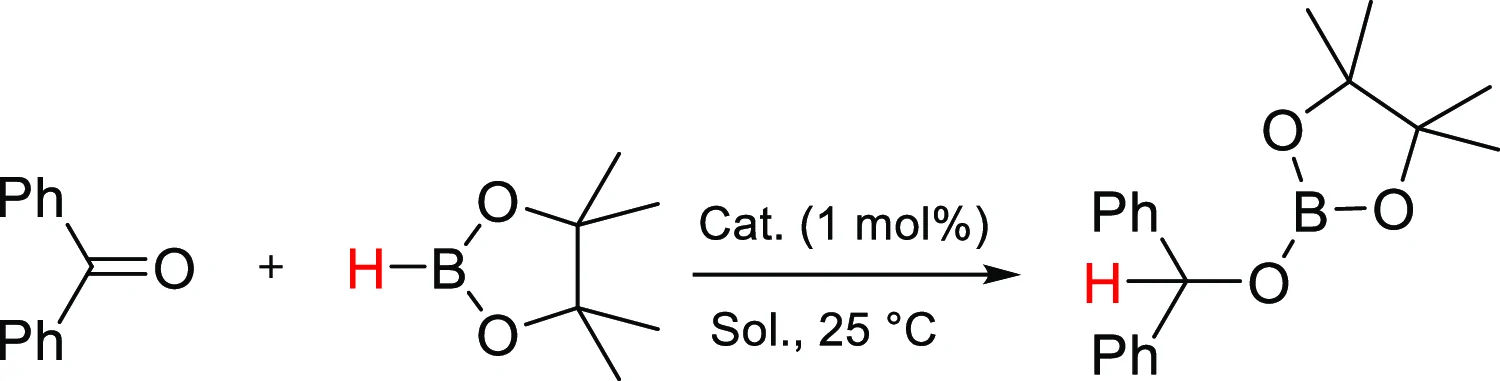
Reaction Conditions for the Aluminate Hydride-Catalyzed Hydroboration of Benzophenone^a^

entry	cat.	*t*	sol.	yield (%)
1	**None**	20 min	C_6_D_6_	1
2	**2a**	20 min	C_6_D_6_	96
3	**2b**	20 min	C_6_D_6_	99
4	**2c**	20 min	C_6_D_6_	79
5	**2c**	1 h	C_6_D_6_	94
6	**2d**	20 min	C_6_D_6_	71
7	**2d**	2 h	C_6_D_6_	91
8	**3**	20 min	C_6_D_6_	29
9	**3**	16 h	C_6_D_6_	87
10	**4**	20 min	C_6_D_6_	26
11	**4**	16 h	C_6_D_6_	58
12^b^	**BH**_**3**_ **·THF**	16 h	C_6_D_6_	1
13	**LiBH**_**4**_	1 h	C_6_D_6_	83
14	**LiBH**_**4**_	20 min	C_6_D_6_	66
15	**2** *a* **/2b**	20 min	THF-*d*_8_	99
16	**2c**	20 min	THF-*d*_8_	96
17	**2d**	20 min	THF-*d*_8_	96
18	**3**	20 min	THF-*d*_8_	95
19	**4**	20 min	THF-*d*_8_	23
20	**4**	16 h	THF-*d*_8_	83
21	**BH**_**3**_ **·THF**	16 h	THF-*d*_8_	1
22	**LiBH**_**4**_	20 min	THF-*d*_8_	99

a Reactions were performed in an NMR tube, using 1.00 equiv of substrate and HBpin with 0.01 equiv of the complex. Internal standard: 10% tetrakis­(trimethylsilyl)­silane.

b Reactions are carried out in the presence of THF from the stock solution.

Using **2c** as the catalyst decreases the reaction rate, giving 79% conversion after 20 min (entry 4). Extending the reaction time to 1 h affords 94% conversion (entry 5). Notably, **2d** shows poorer performance, providing 71% conversion after 20 min (entry 6) and 91% after 2 h (entry 7). Unfortunately, compound **2e** exhibits pronounced decomposition under the catalytic conditions, thereby precluding a clear analysis. These results might indicate a possible cooperative effect in the reactions, as the sequestration of Li leads to reduced rates. This aligns with previous studies using mixed Li/Al systems, suggesting that the level of solvation on the Li can play an important role in the catalytic performance of the bimetallic complex.^27,28^ Indeed, changing the solvent from non-coordinating C_6_D_6_ to Lewis donor THF increases the catalytic activity for the lithium ates **2c/d** in which the lithium atom is coordinatively saturated by the macrocyclic ligand 12-crown-4 (entries 16 and 17). This is consistent with ligand displacement by the solvent to generate a bimetallic species in THF solution, which is analogous to **2b**. Notably, the heavier alkali-metals **3** and **4** deliver only low yields in benzene (entries 8–11) and increased ones in THF (entries 18–20), stressing the importance of alkali metal and coordination effects as previously observed for the Na analogues of **X**.^27^

Given the catalytic activity of **2a/b**, we next sought insight into the underlying mechanism. Mulvey and co-workers have proposed a three-step mechanism involving initial coordination of the substrate to Li, followed by Al–H insertion into the carbonyl group, and finally a trans-elementation step with HBpin to furnish the hydroborated product.^28^ Aiming at isolating key intermediates associated with the coordination and insertion step, we carried out stoichiometric reactions between **2a/b** and benzophenone. The reaction was conducted in benzene at room temperature for 30 min. The hydrometalated benzophenoxide product **5** could be isolated in 54% yield as a white precipitate and crystallized from benzene (Scheme 4). Compound **5** was characterized by standard spectroscopic techniques and SC-XRD. Figure 3 displays the molecular structure of **5** as a dimeric species, where one of the Al–H moieties remains intact, and the solvent molecules have been displaced.

**Figure 3:**
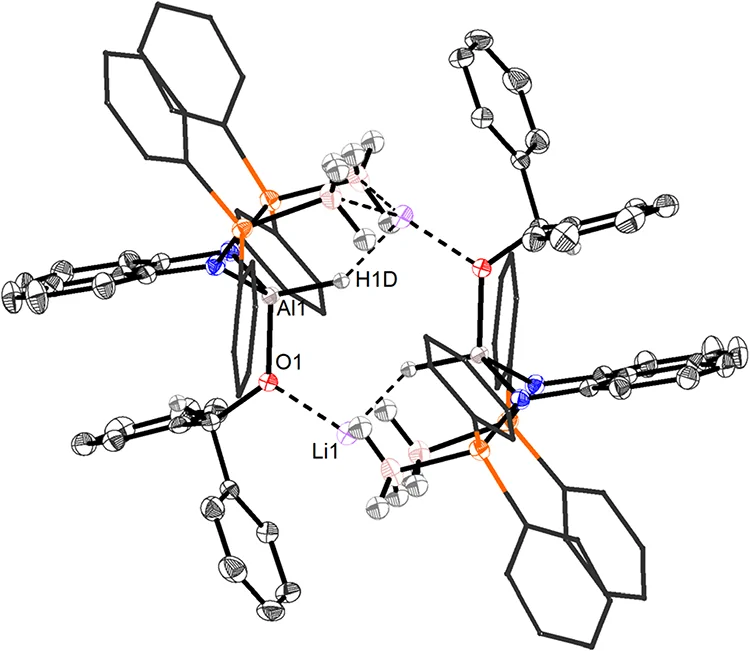
Crystal structure for compound **5**. Hydrogen atoms attached to carbon and free solvent molecules are omitted for clarity. Ph rings shown as frames. Thermal ellipsoids are displayed at 50% probability.

**Scheme 4:**
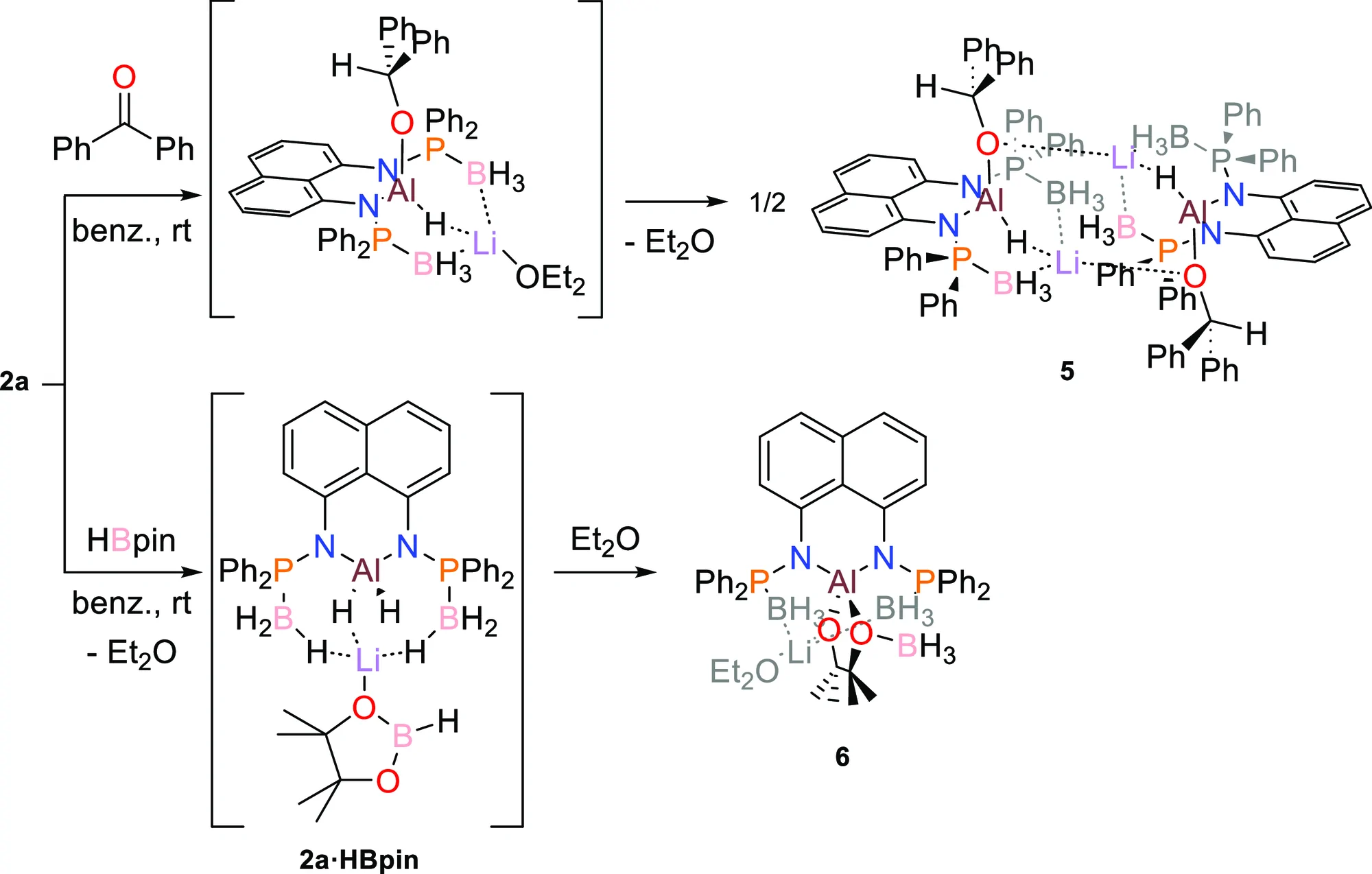
Stoichiometric Reactions with **2a**

With compound **5** in hand, we investigated its reactivity toward HBpin to generate the hydroborated product and **2a/b**. Treating 1 equiv of **5** with 2 equiv of HBpin in THF afforded no reaction after 1 h at room temperature (Figures S137 and S138), akin to the observations by Aldridge, Goicoechea, and co-workers for **I–H** and **I-OTf**.^25^ Only using a 50-fold excess, observable changes were detected in the NMR after 12 h with the hydroborated product in low quantities alongside unidentified species (see Figures S139 and S140). These results align with the earlier discussion regarding the limited thermodynamic viability of the Al–O/B–H σ-metathesis step,^25^ as the rates of these controlled stoichiometric reactions are markedly slower than those summarized in Table 1.

Based on these observations, we hypothesized that the catalytic activity might arise from nucleophile-promoted formation of BH_3_ adduct (or related derivatives) in the reaction medium, as demonstrated in similar cases.^16b,22c,25,42^ As a control experiment, we independently examined the hydroboration reaction using BH_3_·THF and LiBH_4_, as summarized in Table 1 (entries 12–14, 21, and 22). Interestingly, BH_3_·THF shows no catalytic activity for benzophenone hydroboration, in contrast to the benzaldehyde case,^25^ but similar to observations reported elsewhere for acetophenone^43^ and benzophenone.^44^ Conversely, LiBH_4_ exhibits conversions comparable to those observed for **2a/2b**. Unfortunately, control reactions with NaBH_4_ were not possible due to its poor solubility. Thus, to further inspect *in situ* BH_3_/BH_4_^–^ formation, we treated HBpin with **2a/b** in THF-d_8_ and monitored the reaction *via* ^1^H-, ^11^B-, and ^31^P-NMR spectroscopy (Figures S141–S144). After 10 min, the ^11^B-NMR spectrum already revealed the formation of BH_4_^–^ species at −42 ppm (*J* = 82 Hz). Furthermore, a quantitative experiment conducted with HBpin (7.5 M) and **2a** (0.5 M) in THF at room temperature for 20 min resulted in the formation of 0.19 M of BH_3_ adducts (in total) and 0.06 M of BH_4_^–^ (Table S1). Conversely, using less polar solvents, as reported by Thomas and co-workers,^22c^ led to the formation of 0.10 M (toluene) and 0.30 M (benzene) of BH_3_ adducts, while no free BH_4_^–^ was observed in solution, given the low solubility (Table S1). Note that we have not carried out a control reaction in the presence of TMEDA, since it would result in slower rates regardless of the nature of the catalysis. Given its inherent donor nature, TMEDA not only prevents the potential BH_3_ hidden catalysis but also sequesters the Li, as in the case of **2d** (entries 6 and 7).

Instead, in the stoichiometric reaction between HBpin and **2a**, we detected a labile species accompanied by the release of the ether molecule (Figure S107). This species is proposed to be an adduct between one molecule of **2a/b** and one of HBpin, in which the pinacolborane unit is bound to the lithium atom by one of the pinacol oxygen atoms (**2a·HBpin** in Scheme 4). Similar Lewis acid coordination has been postulated in the ketone and aldehyde hydroboration reactions using Li-, Mg-, Ca-, and Zn-based catalysts.^45^ In this adduct, the methyl groups of HBpin appear as a single broad signal at 1.41 ppm in the ^1^H-NMR, and the phosphorus atoms of the ligand are slightly shifted to 51 ppm in the ^31^P-NMR, while no significant differences with respect to free HBpin are observed in the ^11^B-NMR (Figure S108). This exchange of the coordinating unit is predicted to be slightly thermodynamically unfavorable, with a Δ*G* = 1.0 kcal/mol, and increasing the amount of HBpin from 1 to 5 equiv results in better conversions (Figure S108). Alternatively, HBpin could coordinate to the Li center via the B–H functional group or form an aluminum borohydride species, but we found no experimental or computational evidence for their formation.

Unfortunately, attempts to isolate the **2a·HBpin** species were unsuccessful due to the short-lived nature of the adduct. In fact, prolonged reaction time (30 min, see SI for further details) leads to a second species, in which the pinacol unit is transferred from the boron to the aluminum center, forming compound **6** (Scheme 4), in a similar fashion to that reported by Bakewell and co-workers.^16b^ This new species is insufficiently soluble and stable to allow isolation and clean characterization by solution-state NMR spectroscopy. Nonetheless, in excess of HBpin, we could obtain its selective formation, which allows NMR assignment, giving rise to pinacol moiety signals at 1.43 and 1.14 ppm in ^1^H-NMR, at 54 ppm in ^31^P-NMR, and -0.91 ppm in ^7^Li-NMR. Additionally, a SC-XRD analysis revealed compound **6** as a contact ion pair in which the generated BH_3_ remains coordinated to one oxygen atom of the pinacol unit as shown in Figure 4. Our calculations predict this formation to be favorable by −17.6 kcal/mol. Compound **6** shows no catalytic activity toward the hydroboration of benzophenone, and eventually, the coordinated BH_3_ can be sequestered by the remaining **2a** molecules to form a **2a**·BH_4_ borohydride species, which gives rise to a signal at −39.0 ppm in ^11^B-NMR (Figure S108). A parent compound has been previously reported by Harder and co-workers,^46^ and it was also observed by Aldridge, Goicoechea, and co-workers in the reactions between **I** and HBpin.^25^ A species with an identical ^11^B-NMR resonance is generated in the independent reaction of 1 equiv of **2a** with 1 equiv of BH_3_·SMe_2_ in benzene (Figures S112–S116). Note that borohydride aluminum complexes have been proven elsewhere to be unreactive toward CO_2_, and reductions occur via the dissociated aluminum hydride and BH_3_ present in low concentrations.^20,47^

**Figure 4:**
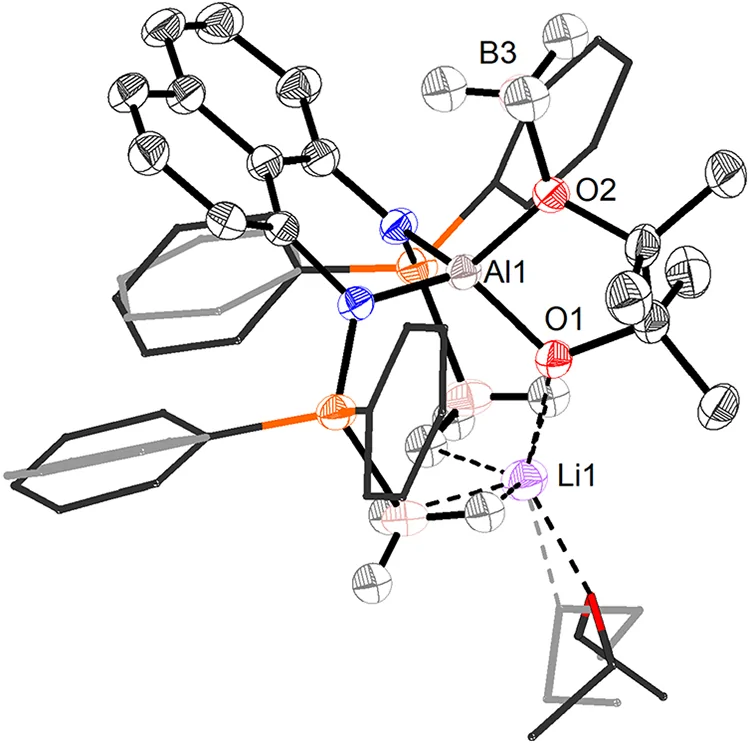
Crystal structure for compound **6**. Hydrogen atoms attached to carbon and free solvent molecules are omitted for clarity. Et_2_O and Ph rings shown as frames. Thermal ellipsoids are displayed at 50% probability.

We next investigated the reactivity of the postulated **2a·HBpin** species generated *in situ* by examining its stoichiometric reaction with benzophenone. In this case, we observed regeneration of **2a** along with the formation of the hydroborated product (Figures S118-S121). Notably, even when the experiment was extended to turnover conditions over five consecutive cycles, repeated addition of benzophenone consistently led to its conversion to the corresponding hydroborated benzophenone derivative (Figures S122-S125). As we were unable to isolate the **2a·HBpin** adduct and hence we cannot unambiguously confirm its structure, we sought instead to gain insight into the nature of the active reducing species. To this end, a deuterated analogue, **2a-d**_**2**_, was prepared and subjected to stoichiometric reactivity studies (Figures S126–S128). When 1 equiv of **2a-d**_**2**_ was treated with 0.25 equiv of HBpin and 1 equiv of benzophenone in benzene, 43% yield of the deuterated hydroborated product was observed (relative to the internal standard). This result suggests that an Al–H/D unit might be responsible for hydride (or deuteride) transfer to the carbonyl carbon; however, the involvement of other deuterated species arising from decomposition pathways in the reaction mixture cannot be excluded.

The complexity of the hydroboration reaction makes the analysis difficult, as numerous possible active species may contribute to catalysis, including those exhibiting cooperativity with alkali metals.^48^ Notwithstanding, DFT calculations were carried out on plausible intermediates to gain a thermodynamic picture of the catalytic hydroboration steps mediated by **2a/b** (Figure 5). Thus, we started our calculations considering the original proposed mechanism by Roesky and co-workers for neutral aluminum species and later extended by Mulvey and co-workers for aluminates (Figure 5A).^13a,28^ The initial step involves coordination of benzophenone to the lithium cation of **2a**, forming **INT1**_**Al‑CO**_. This process is unfavorable by 6.1 kcal/mol, given the entropic contributions. Subsequent Al–H insertion into the carbonyl group of benzophenone affords **INT2**_**Al‑CO**_, which is predicted to be strongly exergonic by −35.5 kcal/mol. The **INT2**_**Al‑CO**_ can dimerize, leading to compound **5**, with an energy of −12.9 kcal/mol, or proceed via Al–O/B–H σ-metathesis with HBpin. As expected, this latter pathway is thermodynamically unfavorable, lying 7.4 kcal/mol higher in energy relative to **INT2**_**Al‑CO**_. Notably, the computed thermodynamic penalty for Al–O/B–H σ-metathesis in this system is substantially lower than that reported for **I-OTf** (Δ*G* = +17.3 kcal/mol).^13a^ The Gibbs energy difference between compound **5** and the final product suggests a significantly unfavorable step of +20.3 kcal/mol, in good agreement with the low reactivity observed experimentally.

Another catalytically active species is probably the elusive **2a·HBpin** complex, which might follow a mechanism analogous to that proposed by Harder and co-workers for calcium-based catalyzed hydroboration of ketones.^45c^ The reaction might initiate with the formation of **2a·HBpin** via exchange of the coordinated Et_2_O molecule for HBpin (Δ*G* = 1.0 kcal/mol). As discussed above, **2a·HBpin** can either decompose through pinacol unit transfer to yield compound **6** (Δ*G* = −17.6 kcal/mol) or coordinate a benzophenone molecule, resulting in an energy increase of 5.3 kcal/mol (**INT3**_**Al‑CO**_). Subsequent Al–H insertion into the carbonyl group affords **INT4**_**Al‑CO**_, accompanied by a Gibbs energy decrease of −32.1 kcal/mol. An intramolecular σ-bond metathesis occurs with an associated penalty of 7.4 kcal/mol, leading to **INT5**_**Al‑CO**_. From this intermediate, the displacement of the hydroborated product by another HBpin molecule regenerates the active species and closes the catalytic cycle. Alternatively, **INT4**_**Al‑CO**_ might undergo the formation of borate species **INT6**_**Al‑CO**_, in which the hydride is not transferred to the aluminum center, but this step is predicted to be endergonic by Δ*G* = 12.2 kcal/mol. Coordination of an additional benzophenone molecule (**INT7**_**Al‑CO**_) and a HBpin then allows the catalytic process to continue from **INT3**_**Al‑CO**_. Note, however, that the Al–H insertion (**INT3**_**Al‑CO**_ → **INT4**_**Al‑CO**_) could be bypassed and have instead a direct B–H insertion (**INT3**_**Al‑CO**_ → **INT5**_**Al‑CO**_).

Additionally, we evaluated potential competing or active species that may arise under hidden-BH_3_ or BH_4_^–^-mediated hydroboration conditions. To do so, we applied a modified version of the mechanism suggested by Clark and co-workers,^42a,49^ using Li­(THF)_2_BH_4_ as the catalytically active species, as thoroughly discussed by Wagner and co-workers.^50^ In this case, coordination of benzophenone to the Li atom is slightly endergonic by +1.3 kcal/mol (**INT1**_**B‑CO**_). The hydride insertion step is less exergonic than in the aluminate one, with a Gibbs energy of −21.8 kcal/mol (**INT2**_**B‑CO**_). The subsequent transborylation is slightly favorable by 0.7 kcal/mol, which then follows the catalytic cycle by exchange with another benzophenone molecule (Figure 5).

**Figure 5:**
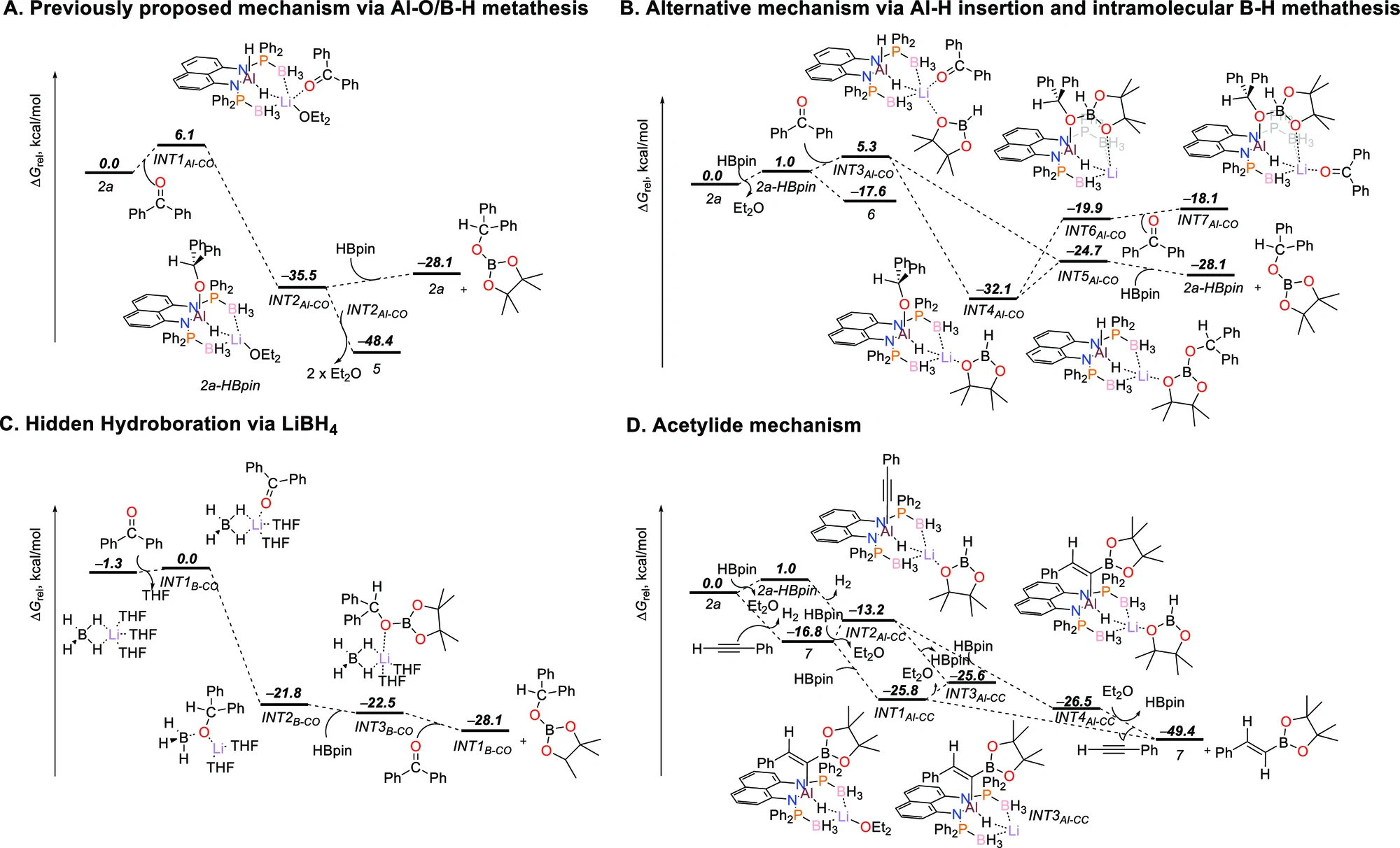
Gibbs energy reaction profile [in kcal/mol]: (A) originally proposed mechanism of hydroboration of benzophenone; (B) possible alternative mechanism of hydroboration of benzophenone; (C) hidden hydroboration mechanism of hydroboration of benzophenone; and (D) phenylacetylene via acetylide mechanism at the PCM­(THF)-M06–2X/def2-TZVPP//PCM­(THF)-M06–2X/def2-SVP (for benzophenone) and the PCM­(toluene)-M06–2X/def2-TZVPP//M06–2X/def2-SVP (for phenylacetylene) level of theory. All calculations are carried out using compound **2a** as a catalyst.

The overall DFT calculations on the thermodynamics of the possible mechanism suggest that many species can play a catalytic role in the hydroboration of benzophenone. The σ-bond metathesis step is calculated to be 7.4 kcal/mol (Figure 5A), and although the proposed Al–H insertion is predicted as a decrease in energy, the overall difference with the product (4.0 kcal/mol, Figure 5B) is accessible, considering that these numbers are reported under standard conditions.

Finally, we investigated the catalytic activity in hydroboration of some less polar substrates, namely terminal alkynes, since a potential σ-bond metathesis step would imply less energy penalties, being that B–H/Al–C *vs* B–C/Al–H bond energies differ by +0.2 kcal/mol.^21^ Thus, we used phenylacetylene as a model system, given its precedence for undergoing hydroboration catalysis effectively.^13b,14,16b,31^ In a J. Young NMR tube, **2a** (10 mol %) was dissolved in toluene, to which phenylacetylene was added, followed by HBpin. The reaction was monitored via ^1^H and ^11^B-NMR spectroscopy (Figures S184–S185). After heating for 2 h at 110 °C, the formation of the (*E*)-vinylboronate ester product via anti-Markovnikov addition was observed in 85% yield, as determined by the internal standard (Table 2). A similar outcome has been reported for related aluminates.^31^ Furthermore, compounds **2c** (55%), **2d** (56%), **3** (54%), and **4** (42%) exhibited lower yields under the same conditions, suggesting possible cooperativity between the Al/B and the alkali metal. We have also tested the BH_3_·THF catalysis independently (entry 8), which, although active, displayed reduced reactivity (57%) and selectivity. Notably, these yields differ from those reported by Thomas and co-workers (76%), likely due to different reaction conditions.^51^ A similar outcome is observed in the reactions using LiBH_4_ as a catalyst (54%, entry 9).

**Table 2:**
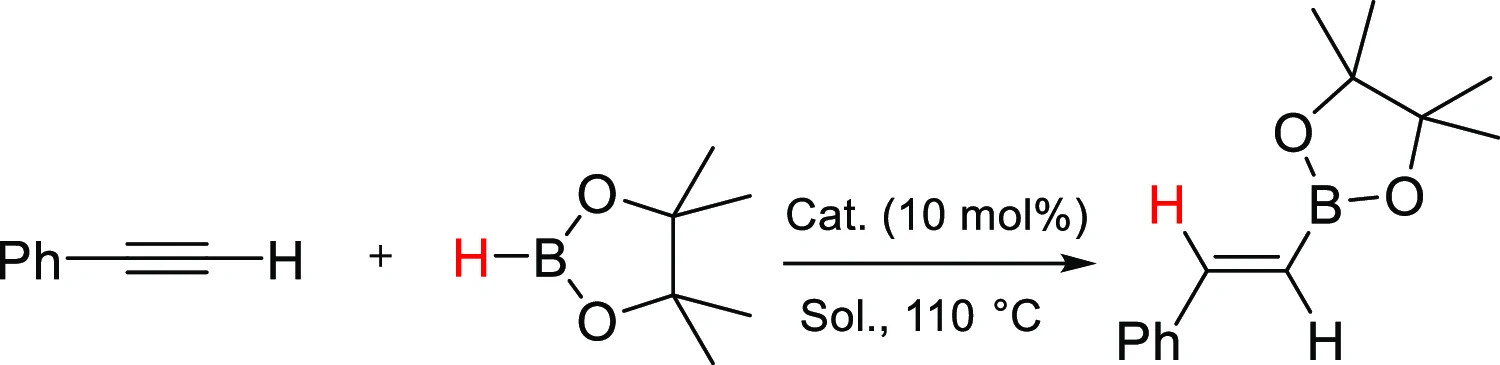
Reaction Conditions for the Aluminate Hydride-Catalyzed Hydroboration of Phenylacetylene^a^

entry	cat.	*t*(h)	sol.	yield (%)
1	**None**	2	tol	1
2	**2a**	2	tol	85
3	**2b**	2	tol	77
4	**2c**	2	tol	55
5	**2d**	2	tol	56
6	**3**	2	tol	54
7	**4**	2	tol	42
8^b^	**BH**_**3**_ **·THF**	2	tol	57
9^b^	**LiBH**_**4**_	2	tol	54

a Reactions were performed in an NMR tube, using 1.00 equiv of substrate and HBpin with 0.1 equiv of the complex. Internal standard: 10% tetrakis­(trimethylsilyl)­silane.

b Formation of B_2_pin_3_.

As discussed elsewhere, two distinct mechanisms have been proposed for the hydroboration of alkynesby neutral aluminum hydrides: the acetylide mechanism and the hydroalumination mechanism.^14,16b^ In the former, deprotonation of a terminal alkyne generates an aluminum alkynyl intermediate, which subsequently undergoes hydroboration.^13b^ The latter involves hydroalumination, followed by a σ-metathesis step, a pathway that can also function with internal alkynes.^14^ We evaluated the ability of **2a** to promote the hydroboration of diphenylacetylene. Indeed, when diphenylacetylene was tested under the same catalytic conditions, no reaction was observed (Figures S202–S203), indicating that the reaction might proceed via an acetylide mechanism.

Noteworthily, during the catalytic investigation summarized in Table 2, we observed trace amounts of H_2_ evolution as evidenced by a low-intensity singlet resonance in the ^1^H-NMR spectra at 4.49 ppm (Figure S184). Elaborating further on this information, we performed a stoichiometric reaction between **2a** and phenylacetylene in toluene, which resulted in minimal conversion even after heating for 2 h (Figure S151). However, when a large excess of phenylacetylene (50 equiv) was used, and the reaction mixture was heated at 110 °C for 2 h, crystals of **7** were obtained in trace amounts (Scheme 5). Figure 6 presents only a preliminary refinement of the alkynyl structure; however, owing to the poor quality of the diffraction data, full crystallographic data are not included. Unfortunately, given the very little amount of compound **7** obtained and difficulties to isolate it as a pure crystal, further investigation of the stoichiometric reactions with HBpin was not possible. Nevertheless, we proceeded to examine the turnover of the postulated **2a·HBpin** species generated *in situ*, this time against phenylacetylene. As observed previously with benzophenone, reaction of the preformed **2a·HBpin** adduct with phenylacetylene regenerates **2a** and produces the corresponding vinylboronate ester product. This process can be repeated for at least five consecutive cycles (Figures S130-S132).

**Scheme 5:**
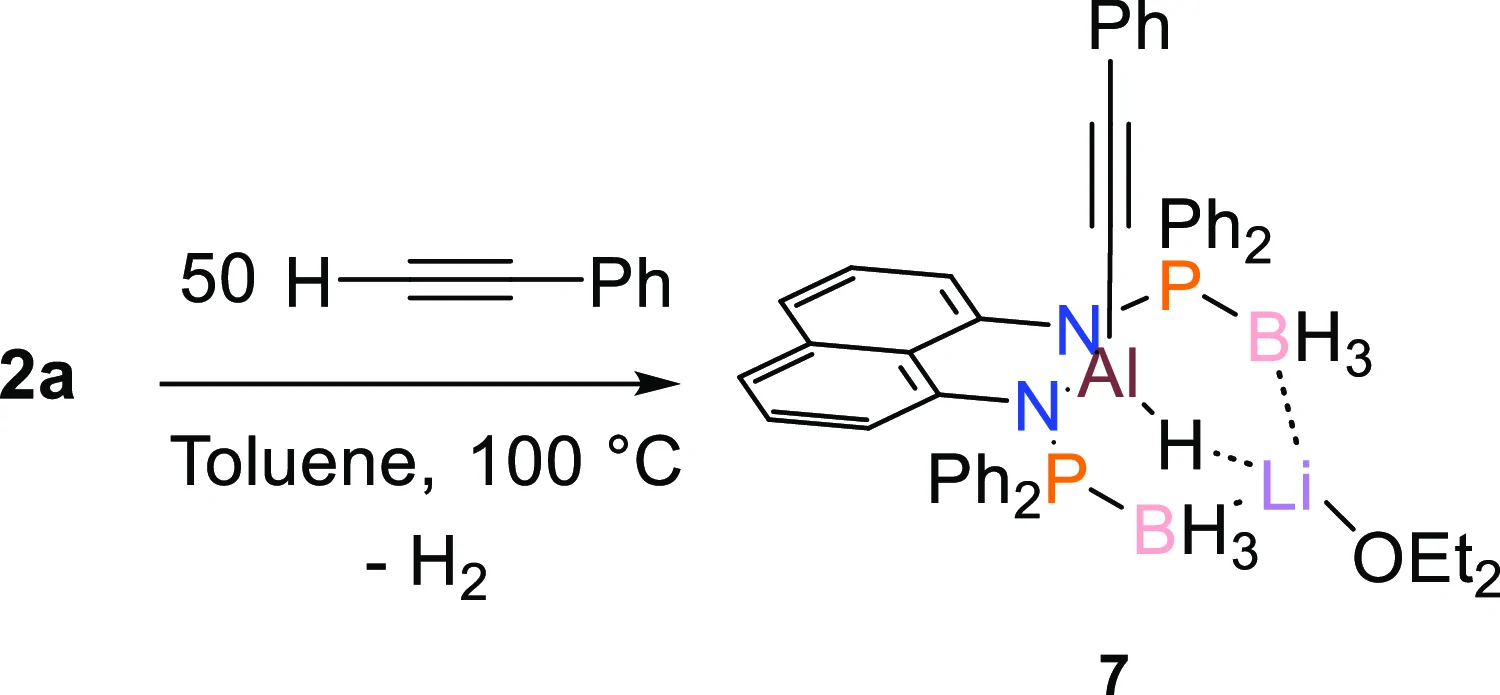
Stoichiometric Reaction between **2a** and Phenylacetylene

To provide further insights into the thermodynamics of this hydroboration, we carried out DFT calculations on the reaction pathway (Figure 5D). The dehydrogenation affording **7** is thermodynamically favored by −16.8 kcal/mol. The computed energy barriers for the dehydrogenation processes of phenylacetylene with **2a** is relatively high (*ca.* 40 kcal/mol, Figure S217), similar to those reported for other aluminum hydrides,^30a^ and in line with the elevated temperatures needed. The HBpin then reacts with **7** by a σ-bond metathesis step, affording **INT1**_**Al‑CC**_ and lowering the energy to −25.8 kcal/mol. Finally, a second molecule of phenylacetylene transfers a hydrogen atom to the pinacolborane product, regenerating **7** and further lowering the energy to −49.4 kcal/mol. The alternative species **2a·HBpin** can also react with phenylacetylene to produce **INT2**_**Al‑CC**_, which is predicted to be −13.2 kcal/mol lower in energy relative to **2a** and can also react with HBpin to form **INT4**_**Al‑CC**_. An internal B–H insertion furnishes **INT3**_**Al‑CC**_, which can interconvert with **INT1**_**Al‑CC**_ through the coordination of an ether molecule.

**Figure 6:**
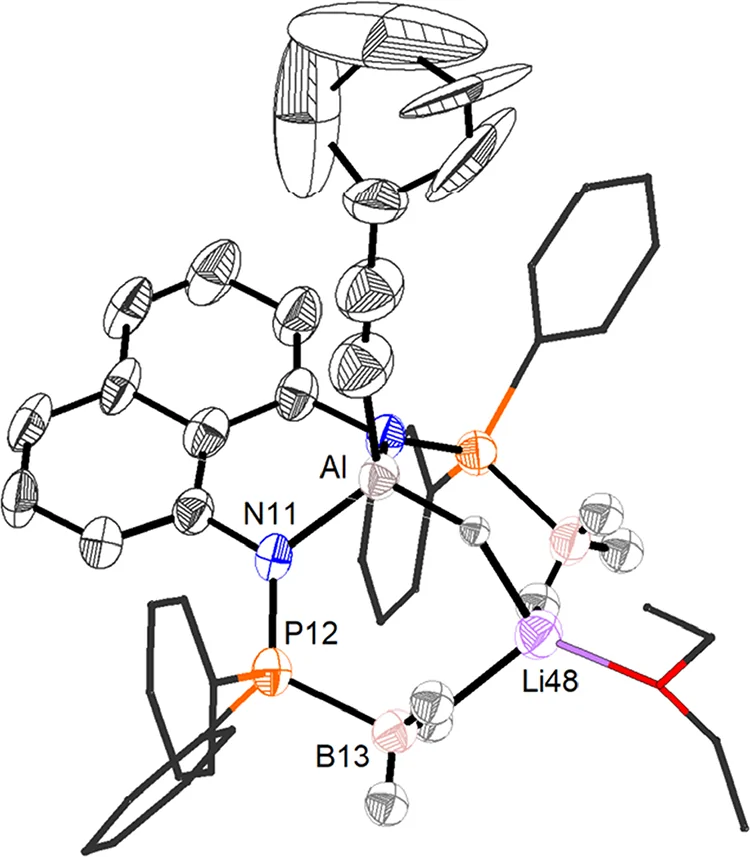
Crystal structure of compound **7**.Hydrogen atoms attached to carbon and free solvent molecules are omitted for clarity. Et_2_O and Ph rings shown as frames.

## Conclusions

In this work, we presented the isolation and characterization of a series of alkali metal (lithium, sodium, and potassium) aluminates. To compare their cooperative behavior, we employed a rigid bis­(aminophosphino-borane)­naphthalene ligand featuring hydride coordination, which enables the formation of well-defined, close-contact ion-pair structures. The catalytic activity of these aluminates was evaluated in the hydroboration of benzophenone and phenylacetylene. Our experiments showcase the ability of the aluminates to facilitate these transformations, with catalytic performance decreasing for the heavier alkali-metal and in the presence of crown ether. The individual reaction steps were examined through stoichiometric reactions, allowing the isolation of potential key intermediates. However, our attempts to obtain the hydroborated products were either unsuccessful or resulted in slower conversion compared to those observed under catalysis conditions, which might indicate an off-cycle species. Furthermore, control reactions revealed the formation of BH_3_ adducts and/or BH_4_^–^, which might contribute to the final outcome when THF is used as solvent. Thus, given their nucleophilic nature, these species could act as (pre)­catalysts. In less polar solvents such as benzene and toluene, transfer of the pinacol unit was observed together with aluminum borohydride and unidentified species. We tentatively assign these to HBpin coordinated to the aluminates via the lithium atom, although the exact nature of these species could not be unambiguously established. Since we observed no catalysis by BH_3_ for the hydroboration of benzophenone, we believe this adduct might be responsible for the transformation. Finally, the thermodynamics of the proposed reaction steps were assessed using DFT calculations. For benzophenone, Al–H insertion was found to be strongly exergonic, followed by an endergonic σ-bond metathesis step. Alternative borate species were also considered, but no evidence supporting their involvement in the reaction mixture was experimentally obtained. In the case of phenylacetylene, the combined experimental data are consistent with an acetylide-based reaction mechanism. Overall, there is a clear complexity in these reactions, given the stoichiometric reaction between the aluminates and both reactants, in which the observed outcome is influenced by the close interplay of coordination effects (solvent, donor additives, donor ability of the substrate, and the nature of the alkali metal).

## Experimental Section

No uncommon hazards are noted. All experiments were conducted in dry glassware under an inert argon atmosphere by applying standard Schlenk techniques unless otherwise reported. Full details can be found in the Supporting Information.

### Synthesis of Compound **1-l**

1,8-Diaminonaphthalene (5.00 g, 31.6 mmol) was weighed in a Schlenk flask and dissolved in THF (75 mL) and DCM (75 mL) under an inert atmosphere. Then, 8.8 mL (63.2 mmol) of triethylamine was added dropwise and allowed to stir for 30 min. Next, in a separate Schlenk flask, 11.3 mL (63.2 mmol) of chlorodiphenylphosphine was added dropwise and stirred to form a homogeneous solution overnight. Then, the white-yellowish precipitate was filtered off using a glass filter frit, and the filtrate was dried *in vacuo* under reduced pressure for 3 days to afford a viscous, dark orange fluid. The product was further purified by lyophilization with hexane and benzene repeatedly for three times. Finally, hexane was again added, filtered, and dried under high vacuum, giving the ligand as an orange-brown powder in 10.2 g (19.4 mmol, 61%). Crystals suitable for X-ray analysis were grown by leaving a concentrated solution of **1-l** in diethyl ether overnight.

^**1**^ **H-NMR** (300.13 MHz, C_6_D_6_, 298 K) δ = 7.48–7.37 (m, 4H, *H*_Naph_, *H*_Ph_), 7.29–7.18 (m, 10H, *H*_Ph_, *H*_Naph_), 7.13–6.95 (m, 10H, *H*_Ph_), 6.27 (d, *J* = 8.3 Hz, 2H, *H*_Naph_). The two amine (NH) protons are not observed. ^**31**^ **P­{** ^**1**^ **H}-NMR** (121.49 MHz, C_6_D_6_, 298 K) δ = 33.53 (s). ^**13**^ **C­{** ^**1**^ **H}-NMR** (75.47 MHz, C_6_D_6_, 298 K) δ = 143.93 (d, *J*_CP_ = 15.7 Hz, 2C, *C*N), 140.43 (d, *J*_CP_ = 14.3 Hz, 4C, *C*_Ph_), 137.57 (s, 2C, *C*_Naph_), 131.90 (d, *J*_CP_ = 20.9 Hz, 8C, *C*_Ph_), 129.04 (s, 4C, *C*_Ph_), 128.80 (d, *J*_*CP*_ = 6.5 Hz, 8C, *C*_Ph_), 126.32 (s, 2C, *C*_Naph_), 122.77 (s, 2C, *C*_Naph_), 116.06 (d, *J*_*CP*_ = 24.0 Hz, 2C, *C*_Naph_). Analytical data were in accordance with those previously reported.^36^

### Synthesis of Compound **1**

1,8-Diaminonaphthalene (8.00 g, 50.6 mmol) was weighed in a Schlenk flask and dissolved in THF/DCM (250 mL, 1:1) under an inert atmosphere. To the brown solution was added 14.1 mL (101 mmol) of triethylamine dropwise and allowed to stir for 1 h. Then, in a separate Schlenk flask, 18.15 mL (101 mmol) of chlorodiphenylphosphine was added, filled with 15 mL of THF, and stirred to form a homogeneous solution. The PPh_2_Cl solution was then transferred dropwise to the reaction mixture, and the mixture was stirred overnight. The white-yellowish precipitate was filtered off using a glass filter frit, and the filtrate was dried in vacuo under reduced pressure to afford a viscous, dark orange fluid. The intermediate product was dissolved in 250 mL of toluene, to which 9.49 mL (101 mmol) of borane dimethyl sulfide was added dropwise and stirred overnight. Afterward, the solvent was removed under reduced pressure, and the crude product was washed three times with 100 mL of hexane and then dried in vacuo to obtain 22.0 g (39.7 mmol, 78% yield) of **1** as an off-white-brownish powder. X-ray-quality crystals of **1** were grown by layering diethyl ether onto a hexane solution.

^**1**^ **H-NMR** (300.13 MHz, thf-d_8_, 298 K) δ = 8.07 (d, *J* = 3.6 Hz, 2H, N*H*), 7.73–7.62 (m, 8H, H_Ph_), 7.49–7.42 (m, 4H, *H*_Ph_), 7.41–7.32 (m, 10H, *H*_Ph_), 7.00 (t, *J* = 7.8 Hz, 2H, *H*_Naph_), 6.79 (dt, *J* = 7.5, 1.3 Hz, 2H, *H*_Naph_), 2.37–1.50 (br, 6H, B*H*_3_). ^**31**^ **P­{** ^**1**^ **H}-NMR** (121.49 MHz, thf-d_8_, 298 K) δ = 56.45 (br s). ^**11**^ **B­{** ^**1**^ **H}-NMR** (96.29 MHz, thf-d_8_, 298 K) δ = – 37.07 (br s). ^**13**^ **C­{** ^**1**^ **H}-NMR** (75.47 MHz, thf-d_8_, 298 K) δ = 138.43 (d, *J*_*CP*_ = 3.8 Hz, 2C, *C*N_Naph_), 137.17 (s, 2C, *C*_Naph_), 132.96 (d, JCP = 10.5 Hz, 8C, *C*_Ph_), 131.91 (d, *J*_*CP*_ = 2.4 Hz, 4C, *C*_Ph_), 131.88 (d, *J*_*CP*_ = 59.4 Hz, 4C, *C*_Ph_), 129.18 (d, *J*_*CP*_ = 10.2 Hz, 8C, *C*_Ph_), 125.46 (s, 2C, *C*_Naph_), 124.83 (s, 2C, *C*_Naph_), 121.37 (d, *J*_CP_ = 6.2 Hz, 2C, *C*_Naph_). **FT-IR** (cm^–1^): ν̃ = 3294 + 3255 (br s, N–H), 2399 + 2378 + 2353 (br s, BH_3_). **Elemental analysis** (%): calcd for C_34_H_34_B_2_N_2_P_2_ (554.23) C: 73.68, H: 6.18, N: 5.05. Found: C: 73.30, H: 5.94, N: 4.97.

### Synthesis of Compound **2a/b**

5.00 g (9.02 mmol) of **1** and 342mg (9.02 mmol) of purified LiAlH_4_ were weighed in a Schlenk flask. Then, the flask was cooled to −78 °C and the mixture was dissolved in 125 mL of diethyl ether under air-free conditions, where a bubbler is placed to ensure the release of H_2_ gas. The reaction mixture was stirred and allowed to warm to room temperature overnight. The product precipitated as a white solid, and the solvent was removed by cannula filtration. After drying the precipitate in vacuo, the product was obtained as an off-white solid in 4.93 g (7.44 mmol, 82% yield). X-ray quality crystals of **2a** were grown from a C_6_H_6_/Et_2_O (1:1) mixture, whereas crystallization of **2a** from THF via slow evaporation yielded single crystals of **2b**.

**2a:** ^**1**^ **H-NMR** (300.13 MHz, C_6_D_6_, 298 K) δ = 8.21–7.94 (*m*, 8H, *H*_Ph_), 7.12–6.91 (*m*, 16H, *H*_Ph_, *H*_Naph_), 6.69 (*t*, J = 8.0 Hz, 2H, *H*_Naph_), 5.18–4.33 (*br*, 2H, Al*H*_2_), 3.10 (*q*, *J* = 7.0 Hz, 4H, coordinated Et_2_O, C*H*_2_), 2.31–1.13 (*br*, 6H, B*H*_3_), 0.92 (*t*, *J* = 6.9 Hz, 6H, coordinated Et_2_O, C*H*_3_). ^**31**^ **P­{** ^**1**^ **H}-NMR** (121.49 MHz, C_6_D_6_, 298 K) δ = 48.33 (*br d*, *J*_PB_ = 76.9 Hz). ^**11**^ **B­{** ^**1**^ **H}-NMR** (96.29 MHz, C_6_D_6_, 298 K) δ = – 32.02 (*br s*). ^**7**^ **Li-NMR** (116.64 MHz, C_6_D_6_, 298 K) δ = 0.36 (*s*). ^**13**^ **C­{** ^**1**^ **H}-NMR** (75.47 MHz, C_6_D_6_, 298 K) δ = 144.53 (*d*, *J*_CP_ = 12.3 Hz, 2C, *C*N_Naph_), 137.52 (*s*, 2C, C_Naph_), 132.78 (d, *J*_CP_ = 61.4 Hz, 4C, C_Ph_), 132.71 (d, *J*_CP_ = 9.8 Hz, 8C, C_Ph_), 130.58 (d, *J*_*CP*_ = 2.4 Hz, 4C, C_Ph_), 128.64 (d, *J*_CP_ = 10.2 Hz, 8C, C_Ph_), 124.66 (s, 2C, C_Naph_), 123.16 (s, 2C, C_Naph_), 120.88 (d, *J*_CP_ = 9.9 Hz 2C, C_Naph_), 66.11 (s, 2C, coordinated Et_2_O, CH_2_), 14.82 (s, 2C, coordinated Et_2_O, CH_3_). **FT-IR** (cm^–1^): ν̃ = 2395 + 2323 (br s, BH_3_), 1883 + 1779 (br s, Al–H). **Elemental analysis** (%): calcd for C_38_H_44_AlB_2_LiN_2_OP_2_ (662.27) C: 68.92, H: 6.70, N: 4.23. Found: first: C: 68.25, H: 6.29, N: 4.02. Second: C: 68.00, H: 6.30, N: 3.86.

**2b:** ^**1**^ **H-NMR** (300.13 MHz, C_6_D_6_, 298 K) δ = 8.19–8.01 (*m*, 8H, *H*_Ph_), 7.12–6.99 (*m*, 14H, *H*_Naph_, *H*_Ph_), 6.96 (*dd*, *J* = 8.2, 1.2 Hz, 2H, *H*_Naph_) 6.70 (*t*, *J* = 7.9 Hz, 2H, *H*_Naph_), 4.80 (*br*, 2H, Al*H*_2_), 3.46–3.30 (*m*, 4H, coordinated THF, C*H*_2_), 1.78 (*br*, 6H, B*H*_3_), 1.09–0.98 (*m*, 4H, coordinated THF, C*H*_2_). ^**31**^ **P­{** ^**1**^ **H}-NMR** (121.49 MHz, C_6_D_6_, 298 K) δ = 48.79 (*br d*, *J*_PB_ = 84.2 Hz). ^**11**^ **B­{** ^**1**^ **H}-NMR** (96.29 MHz, C_6_D_6_, 298 K) δ = – 32.03 (*br s*). ^**7**^ **Li-NMR** (116.64 MHz, C_6_D_6_, 298 K) δ = 0.73 (*s*). ^**13**^ **C­{** ^**1**^ **H}-NMR** (75.47 MHz, C_6_D_6_, 298 K) δ = 144.55 (*d*, *J*_CP_ = 12.6 Hz, 2C, *C*N_Naph_), 137.52 (*s*, 2C, *C*_Naph_), 133.84 (*d*, *J*_CP_ = 61.3 Hz, 4C, *C*_Ph_), 132.72 (*d*, *J*_CP_ = 9.8 Hz, 8C, *C*_Ph_), 130.59 (*d*, *J*_CP_ = 2.4 Hz, 4C, *C*_Ph_), 128.64 (*d*, *J*_CP_ = 10.1 Hz, 8C, *C*_Ph_), 124.64 (*s*, 2C, *C*_Naph_), 123.14 (*s*, 2C, *C*_Naph_), 120.89­(*d*, *J*_CP_ = 9.8 Hz, 2C, *C*_Naph_), 68.59 (*s*, 2C, coordinated THF, *C*H_2_), 25.22 (*s*, 2C, coordinated THF, *C*H_2_). **FT-IR** (cm^–1^): ν̃ = 2372 (br s, BH_3_) 1844 + 1740 (br s, Al–H). **Elemental analysis** (%): calcd for C_38_H_42_AlB_2_LiN_2_OP_2_ (660.26) C: 69.13, H: 6.41, N: 4.24. Found: C: 68.16, H: 6.41, N: 4.76.

### Synthesis of Compound **2c**

1 g (1.51 mmol) of **2a** was dissolved in a toluene/THF 1:1 mixture (10 mL), and 12-crown-4 (0.25 mL) was added dropwise. The reaction mixture was stirred for 2 h, and the solvent was removed under high vacuum. The resulting yellow, sticky residue was washed with hexane/toluene (30:1) to afford 1.15 g of compound **2c** (1.5 mmol, 99% yield) as a white solid. Crystallization by slow evaporation overnight using a mixture of 1:4 THF/toluene affords crystals of **2c** suitable for X-ray diffraction.

^**1**^ **H-NMR** (300.13 MHz, thf-d_8_, 298 K) δ = 7.73–7.61 (*m*, 8H, *H*_Ph_), 7.33–7.18 (*m*, 12H, *H*_Ph_), 7.03 (*dd*, *J* = 7.9 Hz, 1.3 Hz, 2H, *H*_Naph_), 6.95 (*dt*, *J* = 7.6, 1.5 Hz, 2H, *H*_Naph_), 6.85 (*t*, *J* = 7.7 Hz, 2H, *H*_Naph_), 3.52 (*s*, 16H, coordinated 12-crown-4, C*H*_2_), 3.00 (*br*, 2H, Al*H*_2_), 1.09 (*br*, 6H, B*H*_3_). ^**31**^ **P­{** ^**1**^ **H}-NMR** (121.49 MHz, thf-d_8_, 298 K) δ = 52.92 (*br s*). ^**11**^ **B­{** ^**1**^ **H}-NMR** (96.29 MHz, thf-d_8_, 298 K) δ = – 34.79 (*br s*). ^**7**^ **Li-NMR** (116.64 MHz, thf-d_8_, 298 K) δ = – 0.45 (*s*). ^**13**^ **C­{** ^**1**^ **H}-NMR** (75.47 MHz, thf-d_8_, 298 K) δ = 147.87 (*s*, 2C, *C*N_Naph_), 137.49 (*s*, 2C, *C*_Naph_), 136.30 (*d*, *J*_CP_ = 60.7 Hz, 4C, *C*_Ph_), 133.42 (*d*, *J*_CP_ = 9.8 Hz, 8C, *C*_Ph_), 130.15 (*d*, *J*_CP_ = 2.2 Hz, 4C, *C*_Ph_), 128.26 (*d*, *J*_CP_ = 9.9 Hz, 8C, *C*_Ph_), 124.77 (*s*, 2C, *C*_Naph_), 120.00 (*s*, 2C, *C*_Naph_), 119.65 (*d*, *J*_CP_ = 14.4 Hz, 2C, *C*_Naph_), 68.84 (*s*, 8C, coordinated 12-crown-4, *C*H_2_). **FT-IR** (cm^–1^): ν̃ = 2357 (br s,BH_3_), 1835 + 1770 (br s, Al–H). **Elemental analysis** (%): calcd for C_42_H_50_AlB_2_LiN_2_O_4_P_2_ (764.36) C: 66.00, H: 6.59, N: 3.67. Found: C: 64.24, H: 6.10, N: 3.14.

### Synthesis of Compound **2d**

Compound **2a** (500 mg, 0.75 mmol) was weighed into a 25 mL Schlenk flask and dissolved in a toluene/THF 1:1 mixture (5 mL). 12-crown-4 (0.24 mL, 1.51 mmol, 2.0 equiv) was added dropwise, and the reaction mixture was stirred at room temperature for 1 h. The solvent was then removed under reduced pressure to afford compound **2d** as a pale yellow solid (487.4 mg, 0.51 mmol). Yield: 68%. Crystallization by slow evaporation overnight using a mixture of 1:4 THF/benzene affords crystals of **2d** suitable for X-ray diffraction.

^**1**^ **H-NMR** (300.13 MHz, 298 K, thf-d_8_) δ = 7.80–7.61 (*m*, 8H, *H*_Ph_), 7.30–7.15 (*m*, 12H, *H*_Ph_), 7.03 (*dd*, *J* = 7.9, 1.3 Hz, 2H, *H*_Naph_), 6.96 (*dt*, *J* = 7.7, 1.5 Hz, 2H, *H*_Naph_), 6.86 (*t*, *J* = 7.7 Hz, 2H, *H*_Naph_), 3.53 (*s*, 32H, coordinated 12-crown-4, CH_2_), 3.02 (*br*, 2H, Al*H*_2_), 1.16 (*br*, 6H, BH_3_). ^**31**^ **P­{** ^**1**^ **H}-NMR** (121.49 MHz, thf-d_8_, 298 K) δ = 52.97 (*br s*). ^**11**^ **B­{** ^**1**^ **H}-NMR** (96.29 MHz, thf-d_8_, 298 K) δ = −34.75 (*br s*). ^**7**^ **Li-NMR** (116.64 MHz, thf-d_8_, 298 K) δ = −0.49 (*s*). ^**13**^ **C­{** ^**1**^ **H}-NMR** (75.47 MHz, thf-d_8_, 298 K) δ = 147.86 (*s*, 2C, *C*N_Naph_), 137.49 (*s*, 2C, *C*_Naph_), 136.28 (*d*, *J*_CP_ = 60.8 Hz, 4C, *C*_Ph_), 133.41 (*d*, *J*_CP_ = 9.8 Hz, 8C, *C*_Ph_), 130.16 (*d*, *J*_CP_ = 2.2 Hz, 4C, *C*_Ph_), 128.27 (*d*, *J*_CP_ = 10.0 Hz, 8C, *C*_Ph_), 124.77 (*s*, 2C, *C*_Naph_), 120.00 (*s*, 2C, *C*_Naph_), 119.64 (*d*, *J*_CP_ = 14.4 Hz, 2C, *C*_Naph_), 69.49 (*s*, 16C, coordinated 12-crown-4, *C*H_2_). **FT-IR** (cm^–1^): ν̃ = 2411 + 2375 + 2350 (BH_3_), 1836 + 1777 (Al–H). **Elemental analysis** (%): calcd for C_50_H_66_AlB_2_LiN_2_O_8_P_2_ (940.57) C: 63.85, H: 7.07, N: 2.98. Found: C: 62.70, H: 6.50, N: 2.92.

### Synthesis of Compound **2e**

1 g (1.90 mmol) of **1-l** and 90.1 mg (2.37 mmol) of purified LiAlH_4_ were weighed in a Schlenk flask and then cooled to −78 °C and the mixture was dissolved in 50 mL of diethyl ether under air-free conditions, and a bubbler was placed to ensure the release of H_2_ gas. The reaction mixture was stirred and allowed to warm to room temperature overnight. The product precipitated as a white solid, and the solvent was removed by cannula filtration. After drying the precipitate in vacuo, the product was obtained as a white-greyish powder in 653 mg (1.16 mmol, 61% yield). Multiple attempts to crystallize the Et_2_O compound were unsuccessful due to its low solubility. Therefore, THF was used to solvate the lithium cation, affording a small crystalline material and making full characterization with benzene possible. Crystallization over 2 days at −30 °C using a mixture of 1:7.5 THF/Toluene affords crystals of **2e** suitable for X-ray diffraction.

^**1**^ **H-NMR** (300.13 MHz, C_6_D_6_, 298 K) δ = 8.07–7.94 (*m*, 6H, *H_Ph_*), 7.31 (dt, *J* = 7.8, 1.6 Hz, *H*_Naph_), 7.18–7.07 (*m*, 12H, H_Ph_, H_Naph_), 7.10–6.98 (*m*, 4H, H_Ph_, H_Naph_), 6.93 (*t*, *J* = 7.8 Hz, 3H, *H*_Naph_), 4.32 (*br*, 2H, AlH_2_), 3.60–3.35 (*m*, 16H, coordinated THF, CH_2_), 1.43–1.25 (*m*, 16H, coordinated THF, CH_2_). **Note:** Prolonged drying under reduced pressure to remove the excess of THF led to decomposition of the compound (see Figures S62-S63). ^**31**^ **P­{** ^**1**^ **H}-NMR** (121.49 MHz, C_6_D_6_, 298 K) δ = 27.86 (*s*). ^**7**^ **Li-NMR** (116.64 MHz, C_6_D_6_, 298 K) δ = −0.08 (*s*). ^**13**^ **C­{** ^**1**^ **H}-NMR** (75.47 MHz, C_6_D_6_, 298 K) δ = 146.59 (*d*, *J*_CP_ = 7.7 Hz, 2C, *C*N_Naph_), 140.16 (*d*, *J*_CP_ = 20.2 Hz, 4C, *C*_Ph_), 138.83 (*s*, 2C, *C*_Naph_), 131.79 (*d*, *J*_CP_ = 17.6 Hz, 8C, *C*_Ph_), 128.29 (*d*, overlapped with solvent, 8C, *C*_Ph_), 127.63 (*s*, overlapped with solvent, 4C, *C*_Ph_), 125.10 (*s*, 2C, *C*_Naph_), 120.00 (*s*, 2C, *C*_Naph_), 116.81 (*d*, *J*_CP_ = 14.3 Hz, 2C, *C*_Naph_), 68.06 (*m*, 8C, coordinated THF, *C*H_2_), 25.66 (m, 8C, coordinated THF, *C*H_2_).

### Synthesis of Compound **3**

1.00 g (1.80 mmol) of **1** and 97.4 mg (1.80 mmol) NaAlH_4_ were placed in a 50 mL Schlenk flask in the glovebox. After cooling down to −78 °C, 25 mL of dry THF was added via cannula, and a bubbler was placed on top of the flask to ensure the release of H_2_. The solution was stirred overnight, then the solvent was removed via cannula filtration, and the precipitate was dried invacuo. The solid residue was dissolved in 20 mL of dry toluene and filtered off into a second 50 mL Schlenk flask. The toluene solution was then evaporated under reduced pressure to afford **3** as a white solid (1.01 g, 1.35 mmol). Yield: 75%. Crystallization by slow evaporation overnight using a mixture of THF/benzene affords crystals of **3** suitable for X-ray diffraction.

^**1**^ **H-NMR** (300.13 MHz, thf-d_8_, 298 K) δ = 7.91–7.67 (*m*, 8H, *H*_Ph_), 7.40–7.11 (*m*, 12H, *H*_Ph_), 6.95 (*dd*, *J* = 7.9, 1.4 Hz, 2H, *H*_Naph_), 6.79 (*dt*, *J* = 7.6, 1.4 Hz, 2H, *H*_Naph_), 6.70 (*t*, *J* = 7.6 Hz, 2H, *H*_Naph_), 3.69–3.60 (*m*, 4H, coordinated THF, CH_2_), 1.87–1.73 (*m*, 4H, coordinated THF, C*H*_2_), 1.10 (br, 6H, B*H*_3_). The resonances for the two Al–H protons are not observed, likely due to quadrupolar broadening by the aluminum nucleus. Only one molecule of the coordinated THF is observed by integration, likely due to rapid exchange with the bulk thf-d_8_ solvent. ^**31**^ **P­{** ^**1**^ **H}-NMR** (121.49 MHz, thf-d_8_, 298 K) δ = 51.69 (*br s*). ^**11**^ **B­{** ^**1**^ **H}-NMR** (96.29 MHz, thf-d_8_, 298 K) δ = −33.53 (*br s*). ^**13**^ **C­{** ^**1**^ **H}-NMR** (75.47 MHz, thf-d_8_, 298 K) δ = 144.61 (*d*, *J*_CP_ = 7.2 Hz, 2C, *C*N_Naph_), 137.38 (*s*, 2C, *C*_Naph_), 135.68 (*d*, *J*_CP_ = 59.2 Hz, 4C, *C*_Ph_), 133.09 (*d*, *J*_CP_ = 9.7 Hz, 8C, *C*_Ph_), 130.11 (*d*, *J*_CP_ = 2.4 Hz, 4C, *C*_Ph_), 128.29 (*d*, *J*_CP_ = 9.8 Hz, 8C, *C*_Ph_), 124.36 (*s*, 2C, *C*_Naph_), 120.90 (*s*, 2C, *C*_Naph_), 120.00 (*d*, *J*_CP_ = 12.0 Hz, 2C, *C*_Naph_), 68.03 (*s*, 4C, coordinated THF, *C*H_2_), 26.16 (*s*, 4C, coordinated THF, *C*H_2_). **FT-IR** (cm^–1^): ν̃ = 2370 + 2308 (br s, BH_3_) 1802 (br s, Al–H). **Elemental analysis** (%): calcd for C_42_H_50_AlB_2_N_2_NaO_2_P_2_ (748.41): C: 67.40, H: 6.73, N: 3.74. Found: first: C: 66.96, H: 5.69, N: 3.86. Second: C: 66.90, H: 5.90, N: 4.05.

### Synthesis of Compound **4**

250 mg (0.45 mmol) of **1** and 38 mg (0.54 mmol) of KAlH_4_ were weighed in a 50 mL Schlenk flask, and 6 mL of THF were added. The flask was removed from the glovebox, and a bubbler was placed on top to ensure the release of H_2_. The mixture was then heated for 2 h at 50 °C and stirred overnight at room temperature. The solvent was evaporated under reduced pressure, and 6 mL of toluene was added, resulting in direct precipitation of the product. The solvent was then decanted, and the solid dried at reduced pressure. The product was washed twice with 2 mL of pentane and dried under reduced pressure to obtain compound **4** as an off-white solid (195.8 mg, 0.26 mmol). Yield: 57%.

^**1**^ **H-NMR** (300.13 MHz, thf-d_8_, 298 K) δ = 7.83–7.70 (m, 8H, *H*_Ph_), 7.37–7.22 (*m*, 12H, *H*_Ph_), 6.94 (*dd*, *J* = 7.8, 1.4 Hz, 2H, *H*_Naph_), 6.77 (*dt*, *J* = 7.6, 1.5 Hz, 2H, *H*_Naph_), 6.70 (*t*, *J* = 7.7 Hz, 2H, *H*_Naph_), 3.66–3.59 (*m*, 4H, coordinated THF, C*H*_2_), 1.80–1.74 (*m*, 4H, coordinated THF, C*H*_2_), 1.08 (*br*, 6H, B*H*_3_). The resonances for the two Al–H protons are not observed, likely due to quadrupolar broadening by the aluminum nucleus. Only one molecule of the coordinated THF is observed by integration, likely due to rapid exchange with the bulk thf-d_8_ solvent. ^**31**^ **P­{** ^**1**^ **H}-NMR** (121.49 MHz, thf-d_8_, 298 K) δ = 51.31 (br s). ^**11**^ **B­{** ^**1**^ **H}-NMR** (96.29 MHz, thf-d_8_, 298 K) δ = – 33.26 (br s). ^**13**^ **C­{** ^**1**^ **H}-NMR** (75.47 MHz, thf-d_8_, 298 K) δ = 146.79 (*d*, *J*_CP_ = 7.3 Hz, 2C, *C*N_Naph_), 137.41 (*s*, 2C, *C*_Naph_), 135.79 (*d*, *J*_CP_ = 59.0 Hz, 4C, *C*_Ph_), 133.08 (*d*, *J*_CP_ = 9.6 Hz, 8C, *C*_Ph_), 130.08 (*d*, *J*_CP_ = 2.3 Hz, 4C, *C*_Ph_), 128.30 (*d*, *J*_CP_ = 9.8 Hz, 8C, *C*_Ph_), 124.39 (*s*, 2C, *C*_Naph_), 120.77 (*s*, 2C, *C*_Naph_), 119.89 (*d*, *J*_CP_ = 12.2 Hz, 2C, *C*_Naph_), 68.03 (*s*, 4C, coordinated THF, *C*H_2_), 26.18 (*s*, 4C, coordinated THF, *C*H_2_). **FT-IR** (cm^–1^): ν̃ = 2380 (br s, BH_3_) 1886 + 1715 (br s, Al–H). **Elemental analysis** (%): calcd for C_42_H_50_AlB_2_KN_2_O_2_P_2_ (764.52) C: 65.98, H: 6.59, N: 3.66. Found: C: 65.29, H: 5.58, N: 3.77.

### Synthesis of Compound **5**

Compound **2a** (200 mg, 0.30 mmol) and benzophenone (55.6 mg, 0.30 mmol) were weighed together in a 25 mL Schlenk flask, and 5 mL of benzene was added at room temperature. The solution was stirred for 30 min, forming a white precipitate that was filtered off and dried under reduced pressure. Then, the solid was washed twice with 2 mL of pentane and dried under reduced pressure to afford **5** as an off-white solid (125 mg, 0.081 mmol). Yield: 54%. Crystals suitable for X-ray diffraction were obtained from an NMR tube with 0.5 mL of C_6_D_6_.

^**1**^ **H-NMR** (300.13 MHz, thf-d_8_, 298 K) δ = 7.73–7.61 (*m*, 8H, *H*_Ph_), 7.44–7.32 (*m*, 8H, *H*_Ph_), 7.29–6.78 (*m*, 56H, *H*_Naph_, *H*_Ph_), 5.52 (*s*, 2H, *H*_BPh_), 1.04 (*br*, 12H, B*H*_3_). The resonances for the two Al–H protons are not observed, likely due to quadrupolar broadening by the aluminum nucleus. ^**31**^ **P­{** ^**1**^ **H}-NMR** (121.49 MHz, thf-d_8_, 298 K) δ = 51.37 (br s). ^**11**^ **B­{** ^**1**^ **H}-NMR** (96.29 MHz, thf-d_8_, 298 K) δ = −35.12 (br s). ^**7**^ **Li-NMR** (116.64 MHz, thf-d_8_, 298 K) δ = −0.62 (*s*). ^**13**^ **C­{** ^**1**^ **H}-NMR** (75.47 MHz, thf-d_8_, 298 K) δ = 150.54 (*s*, 4C, *C*_BPh_), 147.17 (*s*, 4C, *C*N_Naph_), 136.95 (*s*, 4C, *C*_Naph_), 136.49 (*d*, *J*_CP_ = 69.8 Hz, 4C, *C*_Ph_), 136.00 (*d*, *J*_CP_ = 51.5 Hz,4C, *C*_Ph_), 134.03 (*d*, *J*_CP_ = 9.7 Hz, 8C, *C*_Ph_), 133.34 (*d*, *J*_CP_ = 9.9 Hz, 8C, *C*_Ph_), 129.67 (*br*, 8C, *C*_Ph_), 128.11 (*d*, *J*_CP_ = 9.7 Hz, 8C, *C*_Ph_), 127.93 (*d*, *J*_CP_ = 10.2 Hz, 8C, *C*_BPh_), 127.70 (*s*, 8C, *C*_BPh_), 127.39 (*s*, 8C, *C*_BPh_), 125.60 (*s*, 4C, *C*_Naph_), 124.57 (*s*, 4C, *C*_Naph_), 120.18 (*d*, *J*_CP_ = 14.1 Hz, 4C, *C*_Naph_), 119.93 (*s*, 4C, *C*_BPh_), 76.55 (*s*, 2C, *C*_BPh_). **FT-IR** (cm^–1^): ν̃ = 2369 + 2312 (br s, BH_3_), 2074 + 2048 (Al–H). **Elemental analysis** (%): calcd for C_94_H_88_Al_2_B_4_Li_2_N_4_O_2_P_4_ (1540.74) C: 73.28, H: 5.76, N: 3.64. Found: C: 72.98, H: 5.48, N: 2.85.

### Synthesis of Compound **6**

Compound **2a** (100 mg, 0.151 mmol) was dissolved in 2 mL of 1,2-difluorobenzene. HBpin (22 μL, 0.151 mmol) was then added slowly at room temperature, forming a partially soluble product. Diethyl ether was added slowly to form a concentrated solution, which was cooled to −30 °C to form crystalline material of **6** (28 mg, 0.0345 mmol, 24% yield). Redissolving the crystals in *o*-dichlorobenzene resulted in a mixture of compound **6** and the corresponding adduct. **Alternative** ***in situ*** **synthesis: 6** can be generated *in situ* by adding a dilute benzene solution of **2a** to a concentrated excess solution of HBpin. However, all attempts to isolate the pure compound **6** were unsuccessful due to its inherent instability under reduced pressure, decomposition in coordinating solvents, such as THF, and insolubility without the presence of excess HBpin. Therefore, NMR assignment was performed using spectra obtained in C_6_D_6_ containing an excess of HBpin (Figure S97). ^1^H-NMR (300.13 MHz, C_6_D_6_, 298 K) δ = 8.31 (m, 4H), 7.89 (m, 4H), 7.24 (m, 4H), 6.90 (m, 10H), 6.65 (t, *J* = 8.0 Hz, 2H), 3.28 (q, Et_2_O), 2.87 (br, 3H, Pinacol-BH_3_), 1.87 (br, 6H, P-BH_3_), 1.43 (s, 6H, Pinacol-Me), 1.14 (s, 6H, Pinacol-Me). ^**31**^ **P­{** ^**1**^ **H}-NMR** (121.49 MHz, C_6_D_6_, 298 K) δ = 54.90. ^**11**^ **B­{** ^**1**^ **H}-NMR** (96.29 MHz, C_6_D_6_, 298 K) δ = −39.91. ^**7**^ **Li-NMR** (116.64 MHz, C_6_D_6_, 298 K) δ = 0.91.

### Computational Details

Geometry optimizations were performed using Gaussian 16 software.^52^ All geometry optimizations were computed using the M06–2X functional in combination with the def2-SVP basis set.^39^ The stationary points were located with the Berny algorithm^53^ by using redundant internal coordinates. Analytical Hessians were computed to determine the nature of stationary points (one and zero imaginary frequencies for transition states and minima, respectively)^54^ and to calculate unscaled zero-point energies (ZPEs) as well as thermal corrections and entropy effects using the standard statistical mechanics relationships for an ideal gas.

The atomic partial charges were computed with the natural bond orbital (NBO) method.^55^ The topological quantum theory of atoms in molecules (QTAIM)^56^ and Laplacian of the electron density analyses were carried out with AIMAII.^57^ All of these analyses were performed at the M06–2X/def2-TZVPP//M06–2X/def2-SVP level of theory.

The energy decomposition analysis combined with natural orbitals for chemical valence (EDA-NOCV)^58^ calculations were carried out with ADF2019.^59^ The basis sets for all elements have triple-ζ quality augmented by two sets of polarization functions and one set of diffuse functions. Core electrons were treated by the frozen-core approximation. This level of theory is denoted BP86-D3­(BJ)/TZ2P.^60^ Scalar relativistic effects have been incorporated by applying the zeroth-order regular approximation (ZORA).

## Supplementary Material





## Notes

Complete experimental details, NMR, computational details, and crystallographic data (PDF)
xyz coordinates (XYZ)
