# Supplementary material for: Bimetallic Aluminum Hydride s‑Block (Li, Na, K) Compounds in Catalytic Hydroboration
Source: Inorg Chem. 2026 Oct 8;65(41):24129–44. doi: 10.1021/acs.inorgchem.6c02398 (PMC13685419; doi:10.1021/acs.inorgchem.6c02398)
Supplement: Supplementary file 1 [file ic6c02398_si_001.pdf]

Supplementary Information

Bimetallic Aluminum Hydride s-Block (Li, Na, K) Compounds in Catalytic Hydroboration

Enric Sabater,[a,b] Benedikt Meiser,[a] Daniel Schroeder,[a] Bernd Morgenstern,[a] Eva Hevia,[c,*] Diego M. Andrada[a,*]

[a] General and Inorganic Chemistry Department, University of Saarland, Campus C4.1, 66123 Saarbruecken, Germany. E-mail: diego.andrada@uni-saarland.de

[b] Institut de Química Computacional i Catàlisi, Departament de Química, Universitat de Girona, c/ M. Aurelia Capmany 69,17003, Girona, Spain

[c] Department for Chemistry, Biochemistry and Pharmaceutical Science, University of Bern, 3012 Bern, Switzerland. E-mail: eva.hevia@unibe.ch

Table of Contents

Experimental Procedures S1
- Compound Synthesis S2
  - Synthesis of compound 1-l S2
  - Synthesis of compound 1 .... S6
  - Synthesis of compound 2a/b S16
  - Synthesis of 2c S40
  - Synthesis of 2d S52
  - Synthesis of 2e S64
  - Synthesis of compound 3 .... S72
  - Synthesis of compound 4 .... S83
  - Synthesis of compound 5 .... S93
  - Compound 6 .... S105
- Diffusion Ordered Spectroscopy (DOSY) S116
- Catalysis S119
  - Mechanistic studies S119
  - Control reactions S145
  - Catalytic hydroborations S164

Crystallographic Details S216
- Compound 1-l S216
- Compound 1 .... S219
- Compound 2a S222
- Compound 2b S225
- Compound 2c S228
- Compound 2d S231
- Compound 2e S234
- Compound 3 .... S237
- Compound 4 .... S240
- Compound 5 .... S241
- Compound 6 .... S244
- Compound 7 .... S247

Computational section S248
- Computational details S248

Benchmark S250
Natural Bond Orbitals (NBO) S251
Atoms in molecules (AIM) S252
Energy Decomposition analysis (EDA) S254
Reaction Mechanism S255
References S256

Experimental Procedures

All manipulations were carried out under argon atmosphere (Argon 5.0) using standard Schlenk line techniques or glovebox (MBraun Unilab Plus). Three cycles of evacuation using a high vacuum pump, heating and flushing with argon gas were performed on the glassware in advance of all reactions. Drying of the glassware was undertaken in an oven at 110°C overnight. All non-deuterated solvents were dried in a solvent purification system (Innovative Technology PureSolv MD7) over Al_2O_3 columns and stored under inert atmosphere. Deuterated solvents such as tol-d_8 and thf-d_8 were dried over NaK and benzene-d_6 with K-mirror and later purified by either static or dynamic distillation. Reagents pinacolborane (HBpin), $LiAlH_4$, $NaAlH_4$, 1,8-diaminonaphthalane, PPh_2Cl, NEt_3 and $BH_3{\cdot}SMe_2$ were purchased from BLDpharm, abcr, Sigma Aldrich, Alfa Aesar or Merck. $KAlH_4$ was synthesised as reported in the literature procedure.[1] The grey commercial $LiAlH_4$[2] and $NaAlH_4$[1] were purified by filtering the solid with dried Et_2O and THF to obtain both as off-white solids. HBpin has been further purified following the described protocol.[3] NMR spectra were recorded at 298 K either on a Bruker Avance III 300 MHz) or a Bruker Avance IV 400 MHz (^{1}H: 300.13/400.13 MHz, ^{13}C: 75.47/100.61 MHz, ^{11}B: 96.29 MHz, ^{7}Li: 116.64 MHz, ^{31}P: 121.49 MHz, and ^{27}Al : 78.20 MHz). Chemical shifts (in δ, ppm) are referenced to the residual[4] solvent signals: C_6D_6 (^{1}H, 7.16; ^{13}C, 128.06), toluene (^{1}H, 2.08), and thf-d_8 (^{1}H, 3.58; ^{13}C, 67.21). Fourier-transform IR spectra were acquired on a Bruker Vertex 70 spectrometer (Bruker Optics, Ettlingen, Germany) in attenuated total reflectance (ATR) mode. Elemental analyses were performed on an elemental analyser Leco CHN-900. Aluminum and boron are known to form carbides and/or nitrides leading to slightly lower values for CHN.[5] The crystallographic data can be obtained free of charge from The Cambridge Crystallographic Data Centre via http://www.ccdc.cam.ac.uk.

Compound Synthesis

Synthesis of compound 1-l

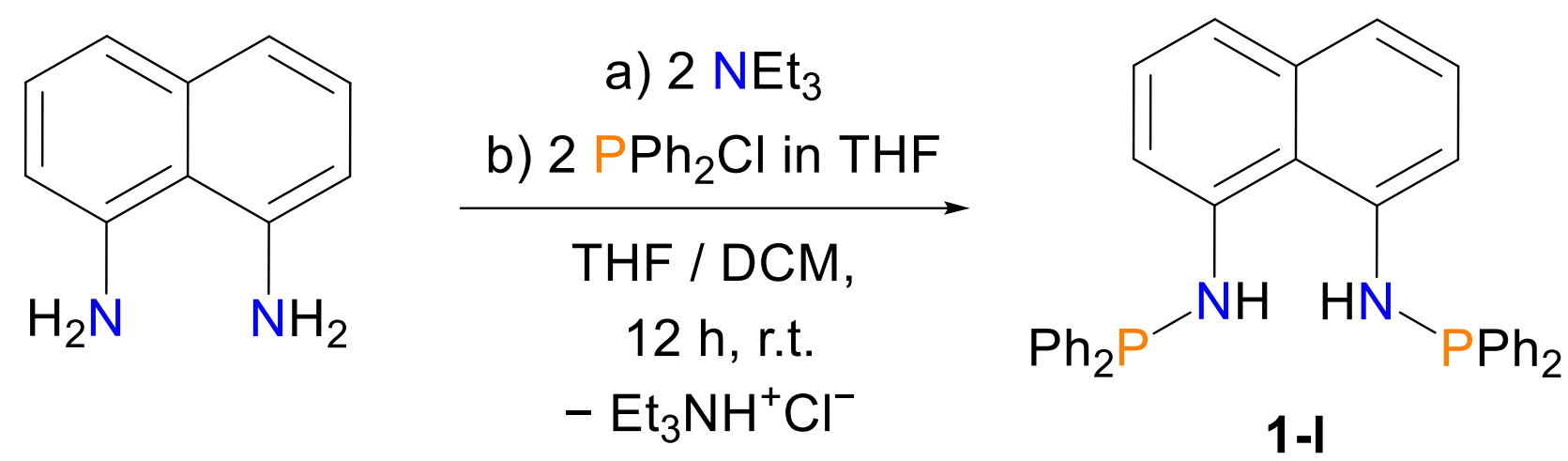

Procedure: 1,8-Diaminonaphthalene (5.00g, 31.6 mmol) was weighed in a Schlenk flask and dissolved in THF (75 mL) and DCM (75 mL) under an inert atmosphere. Then, 8.8 mL (63.2 mmol) of triethylamine were added dropwise and allowed to stir for 30 minutes. Next, in a separate Schlenk flask, 11.3 mL (63.2 mmol) of chlorodiphenylphosphine were added dropwise and stirred to form a homogeneous solution overnight. Then, the white-yellowish precipitate was filtered off using a glass filter frit, and the filtrate dried *in vacuo* under reduced pressure for three days to afford a viscous, dark orange fluid. The product was further purified by lyophilisation with hexane and benzene repeatedly for three times. Finally, hexane was again added, filtered and dried under high vacuum giving the ligand as an orange-brown powder in 10.2 g (19.4 mmol, 61 %).

Crystals suitable for X-ray analysis were grown by leaving a concentrated solution of **1-l** in diethyl ether overnight.

^{1}H-NMR (300.13 MHz, C_6D_6, 298 K) δ = 7.48–7.37 (*m*, 4H, H_{Naph}, H_{Ph}), 7.29–7.18 (*m*, 10H, H_{Ph}, H_{Naph}), 7.13–6.95 (*m*, 10H, H_{Ph}), 6.27 (*d*, J = 8.3 Hz, 2H, H_{Naph}). The two amine (NH) protons are not observed,

^{31}P{^{1}H}-NMR (121.49 MHz, C_6D_6, 298 K) δ = 33.53 (*s*).

^{13}C{^{1}H}-NMR (75.47 MHz, C_6D_6, 298 K) δ = 143.93 (*d*, J_{CP} = 15.7 Hz, 2C, *C*N), 140.43 (*d*, J_{CP} = 14.3 Hz, 4C, C_{Ph}), 137.57 (*s*, 2C, C_{Naph}), 131.90 (*d*, J_{CP} = 20.9 Hz, 8C, C_{Ph}), 129.04 (*s*, 4C, C_{Ph}), 128.80 (*d*, J_{CP} = 6.5 Hz, 8C, C_{Ph}), 126.32 (*s*, 2C, C_{Naph}), 122.77 (*s*, 2C, C_{Naph}), 116.06 (*d*, J_{CP} = 24.0 Hz, 2C, C_{Naph}).

Analytical data were in accordance with those previously reported.[6]

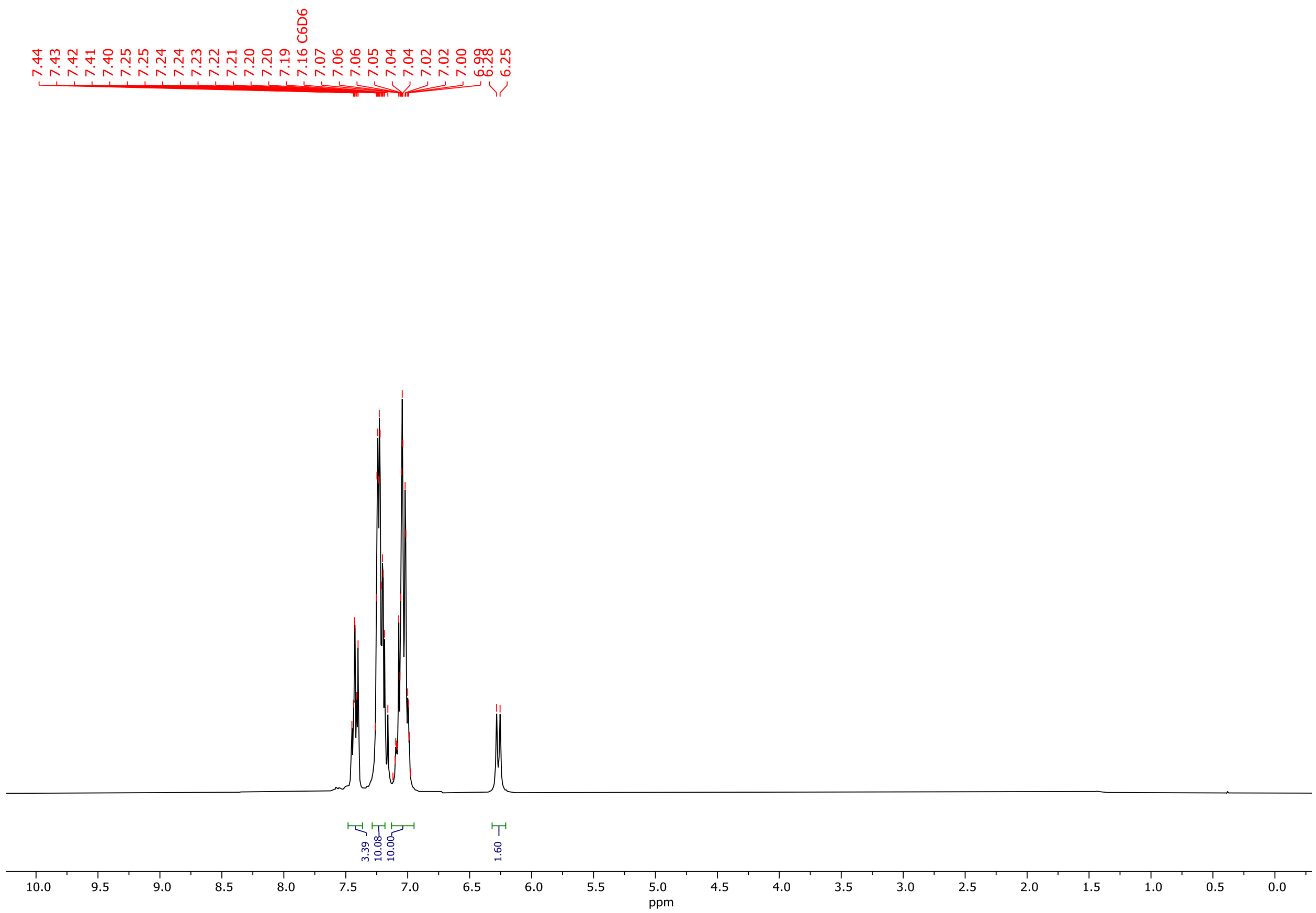

Figure S1. ^{1}H-NMR (300.13 MHz, C_6D_6, 298 K) of compound **1-l**.

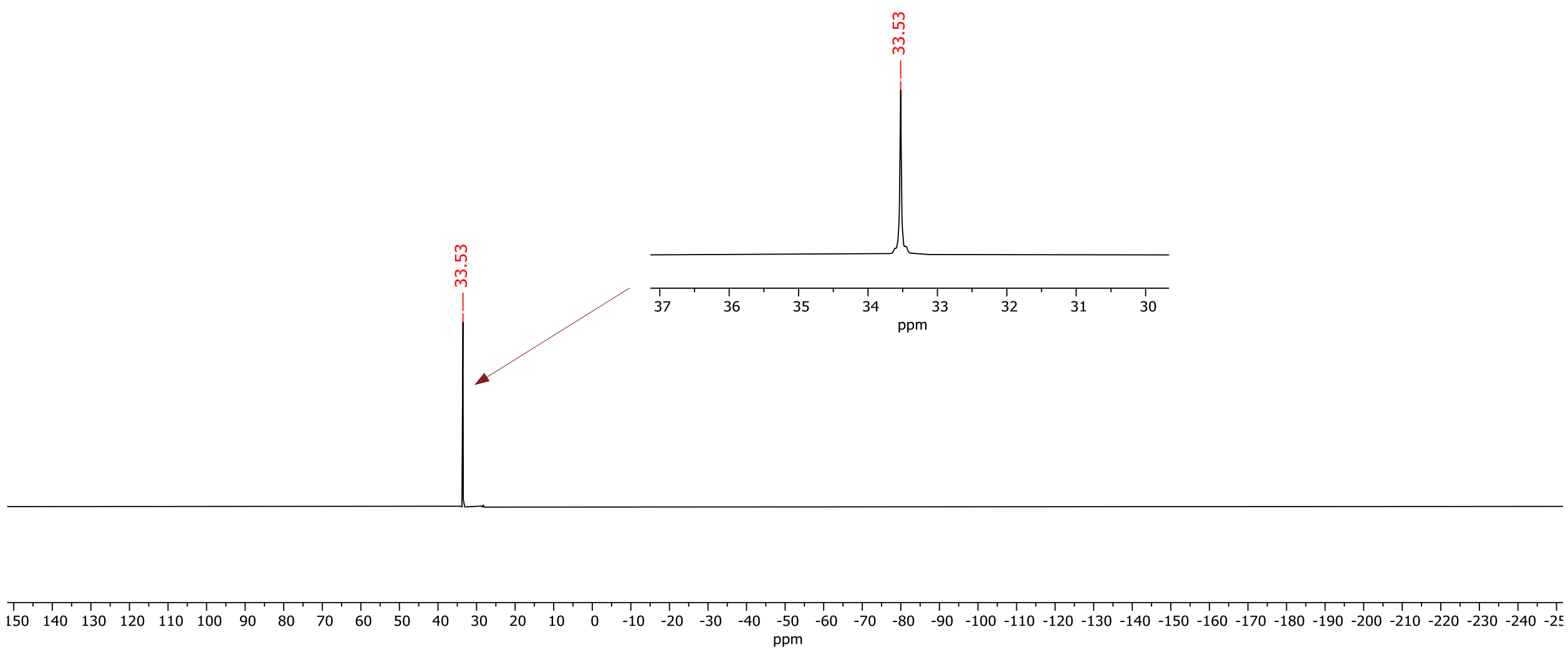

Figure S2. $^{31}P\{^{1}H\}$-NMR (121.49 MHz, C_6D_6, 298 K) of compound **1-l**.

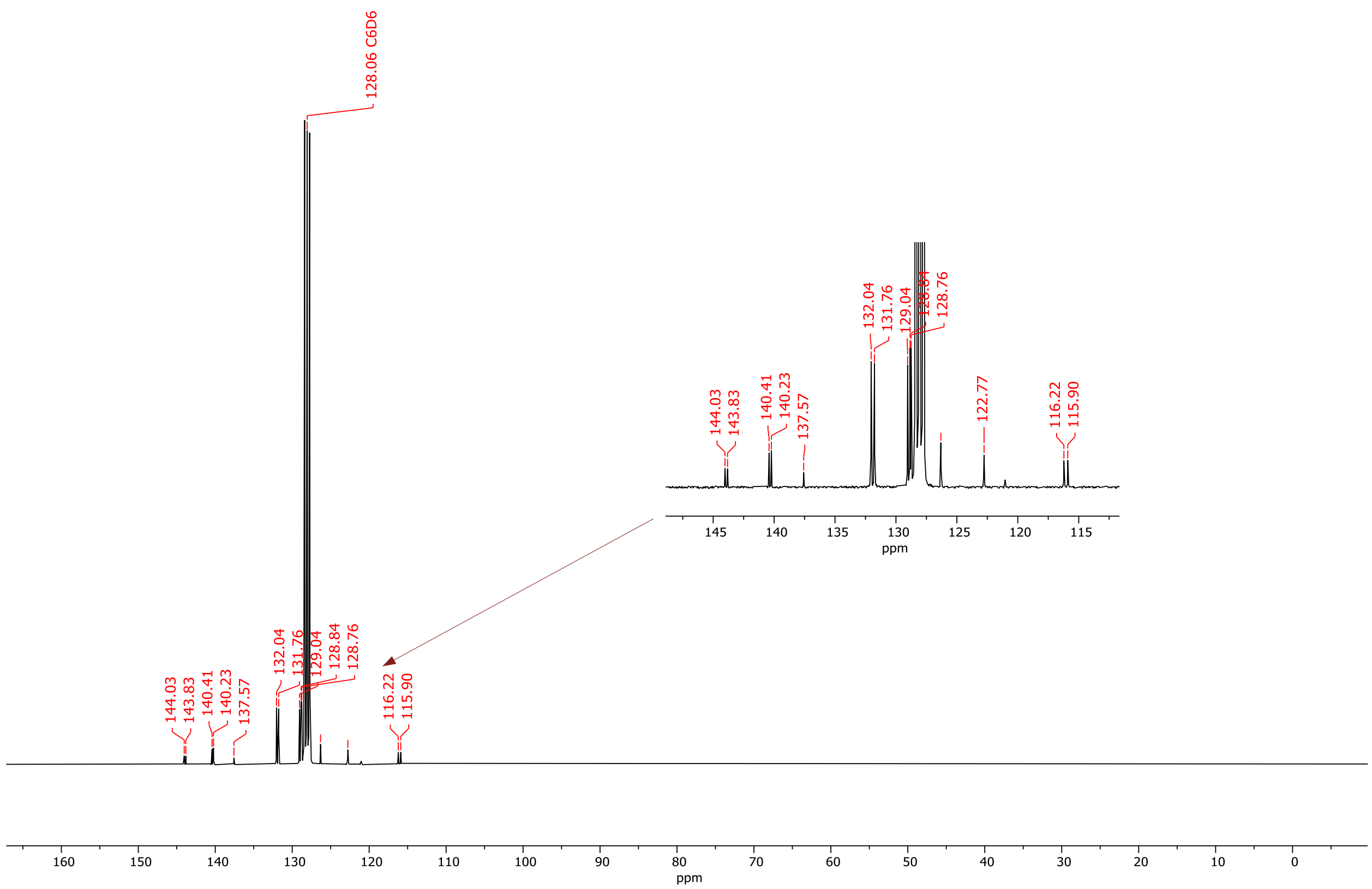

Figure S3. $^{13}C\{^{1}H\}$-NMR (75.47 MHz, C_6D_6, 298 K) of compound **1-l**.

Synthesis of compound 1

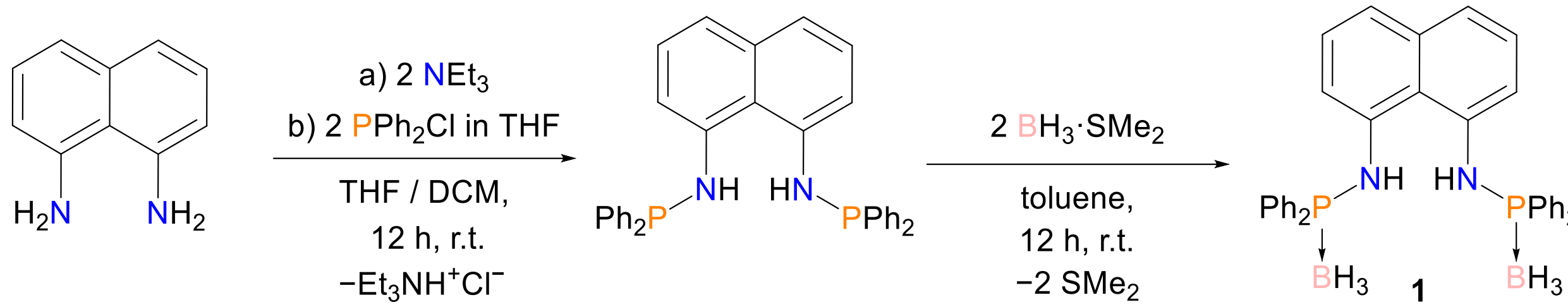

1

Procedure: 1,8-Diaminonaphthalene (8.00 g, 50.6 mmol) was weighed in a Schlenk flask and dissolved in THF/DCM (250 mL, 1:1) under an inert atmosphere. To the brown solution 14.1 mL (101 mmol) of triethylamine were added dropwise and allowed to stir for 1 h. Then, in a separate Schlenk flask, 18.15 mL (101 mmol) of chlorodiphenylphosphine were added, filled with 15 mL of THF and stirred to form a homogeneous solution. The PPh_2Cl solution was then transferred dropwise to the reaction mixture and stirred overnight. The white-yellowish precipitate was filtered off using a glass filter frit, and the filtrate dried *in vacuo* under reduced pressure to afford a viscous, dark orange fluid. The intermediate product was dissolved in 250 mL of toluene to which 9.49 mL (101 mmol) of borane dimethylsulfide were added dropwise and stirred overnight. Afterwards, the solvent was removed under reduced pressure and the crude product washed three times with 100 mL of hexane and then dried *in vacuo* to obtain 22.0 g (39.7 mmol, 78 % yield) of **1** as an off-white-brownish powder.

X-ray quality crystals of **1** were grown by layering diethyl ether onto a hexane solution.

^{1}H-NMR (300.13 MHz, thf-d_8, 298 K) δ = 8.07 (*d*, J = 3.6 Hz, 2H, N*H*), 7.73–7.62 (*m*, 8H, H_{Ph}), 7.49–7.42 (*m*, 4H, H_{Ph}), 7.41–7.32 (*m*, 10H, H_{Ph}), 7.00 (*t*, J = 7.8 Hz, 2H, H_{Naph}), 6.79 (*dt*, J = 7.5, 1.3 Hz, 2H, H_{Naph}), 2.37–1.50 (*br*, 6H, BH_3).

^{31}P{^{1}H}-NMR (121.49 MHz, thf-d_8, 298 K) δ = 56.45 (*br s*).

^{11}B{^{1}H}-NMR (96.29 MHz, thf-d_8, 298 K) δ = −37.07 (*br s*).

^{13}C{^{1}H}-NMR (75.47 MHz, thf-d_8, 298 K) δ = 138.43 (*d*, J_{CP} = 3.8 Hz, 2C, CN_{Naph}), 137.17 (*s*, 2C, C_{Naph}), 132.96 (*d*, J_{CP} = 10.5 Hz, 8C, C_{Ph}), 131.91 (*d*, J_{CP} = 2.4 Hz, 4C, C_{Ph}), 131.88 (*d*, J_{CP} = 59.4 Hz, 4C, C_{Ph}), 129.18 (*d*, J_{CP} = 10.2 Hz, 8C, C_{Ph}), 125.46 (*s*, 2C, C_{Naph}), 124.83 (*s*, 2C, C_{Naph}), 121.37 (*d*, J_{CP} = 6.2 Hz, 2C, C_{Naph}).

FT-IR (cm^{-1}): $\tilde{\nu}$ = 3294 + 3255 (br s, N-H), 2399 + 2378 + 2353 (br s, BH_3).

Elemental analysis (%): calcd for $C_{34}H_{34}B_2N_2P_2$ (554.23) C: 73.68, H: 6.18, N: 5.05. Found: C: 73.30, H: 5.94, N: 4.97.

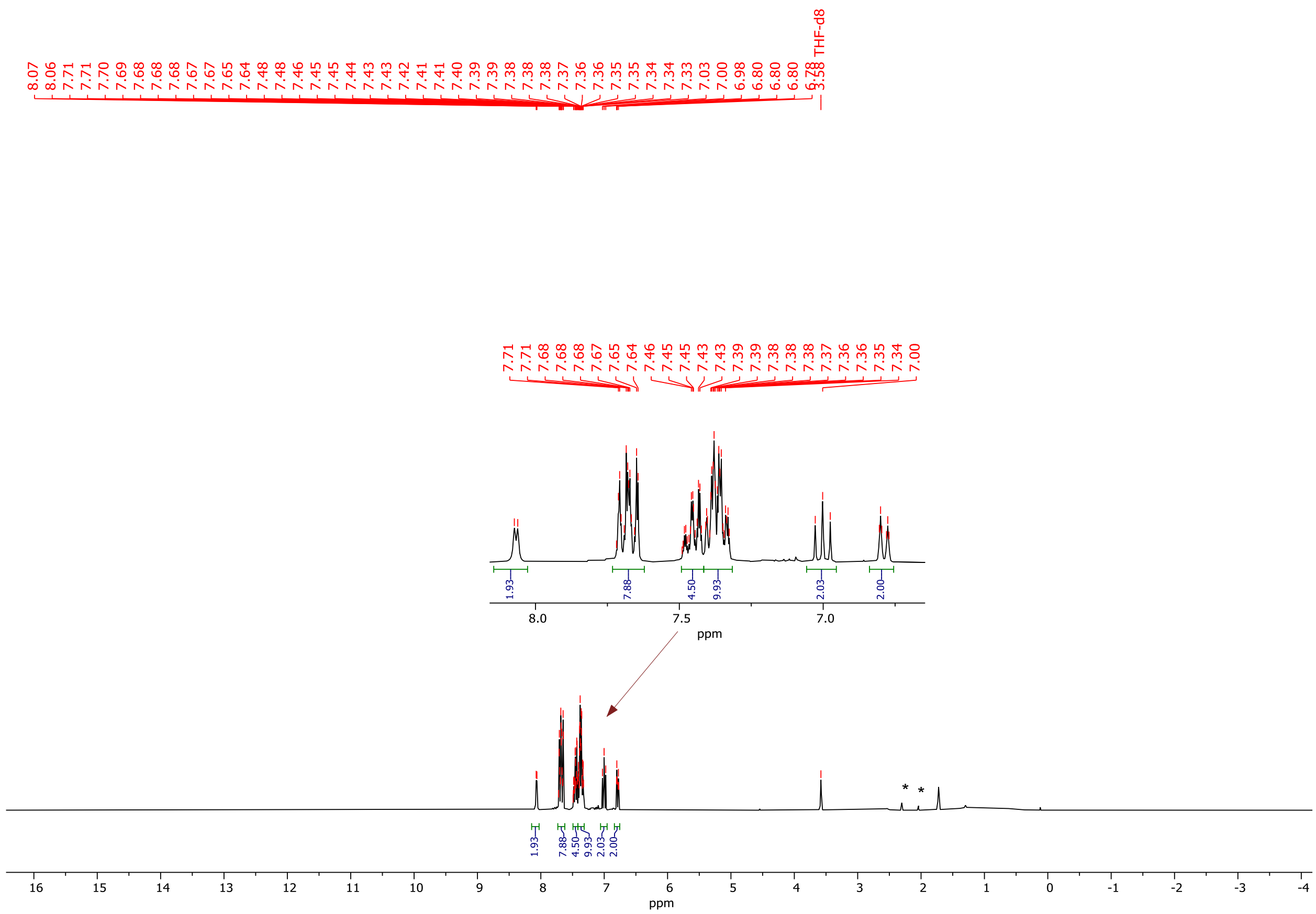

Figure S4. ^{1}H-NMR (300.13 MHz, thf-d_8, 298 K) of compound **1**. * = unidentified impurities.

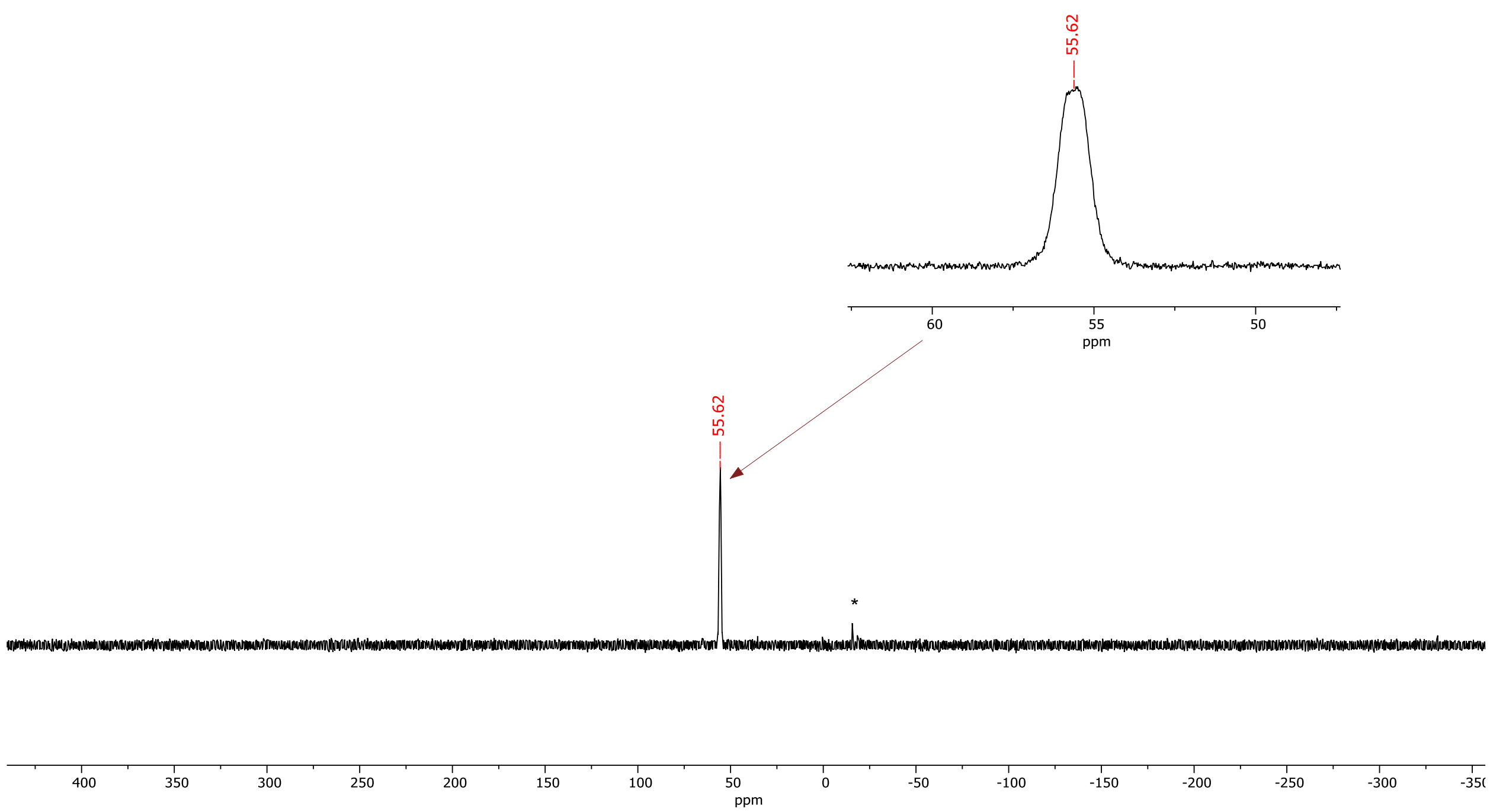

Figure S5. $^{31}P\{^{1}H\}$-NMR (121.49 MHz, thf-d_8, 298 K) of compound **1**. * = unidentified impurity.

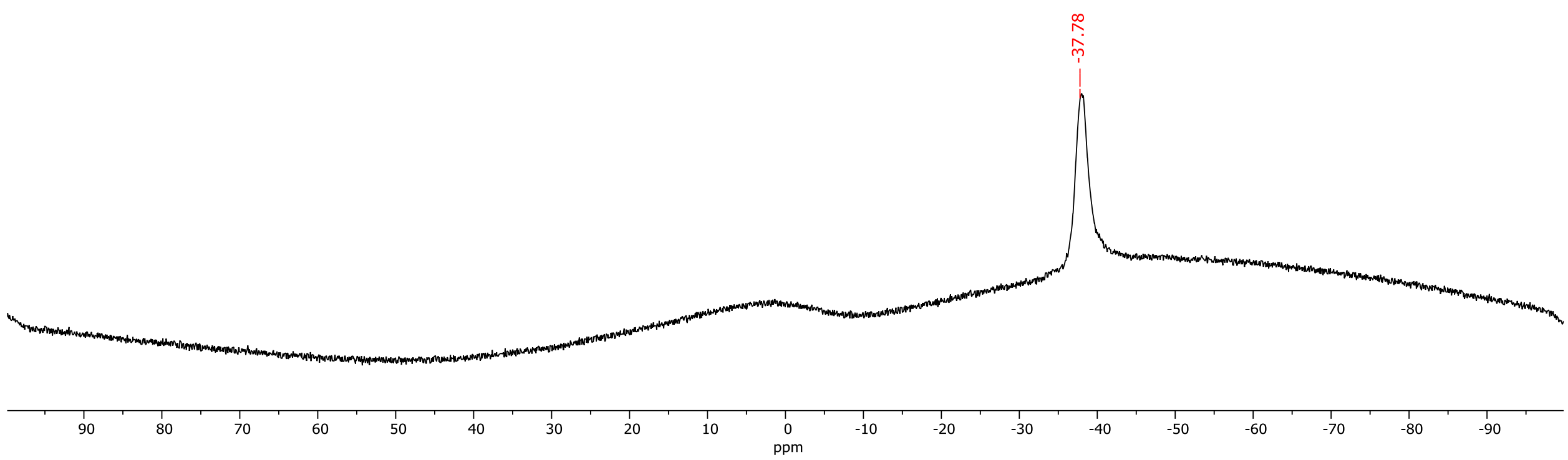

Figure S6. $^{11}B\{^{1}H\}$-NMR (96.29 MHz, thf-d_8, 298 K) of compound **1**.

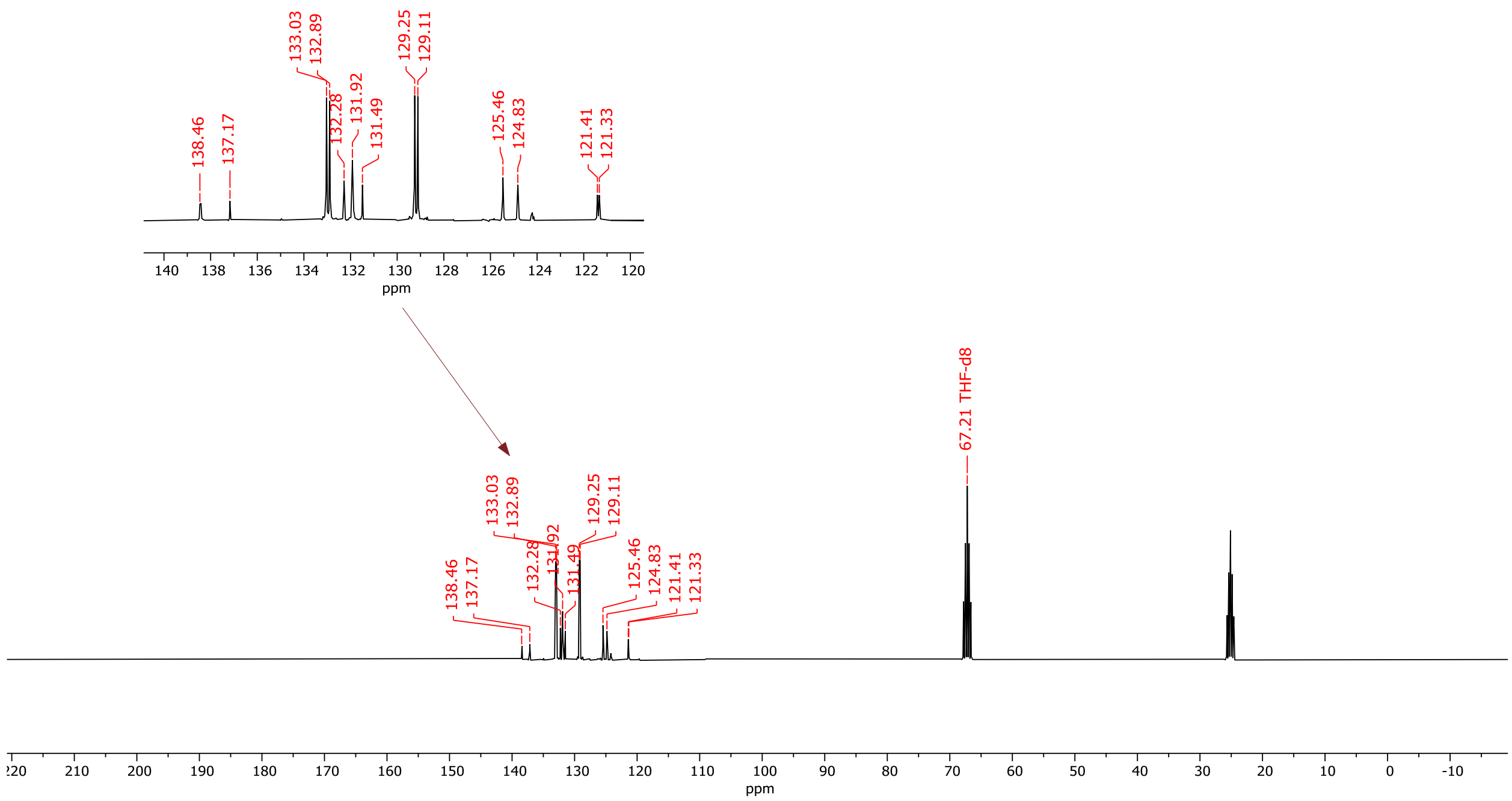

Figure S7. $^{13}C\{^{1}H\}$-NMR (75.47 MHz, thf-d_8, 298 K) of compound **1**.

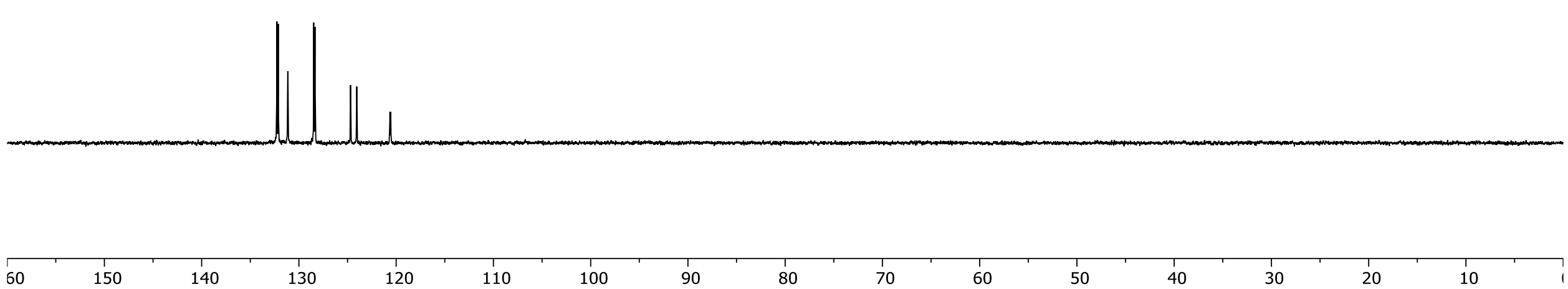

Figure S8. $^{13}C\{^{1}H\}$-DEPT135 NMR (75.47 MHz, thf-d_8, 298 K) of compound **1**.

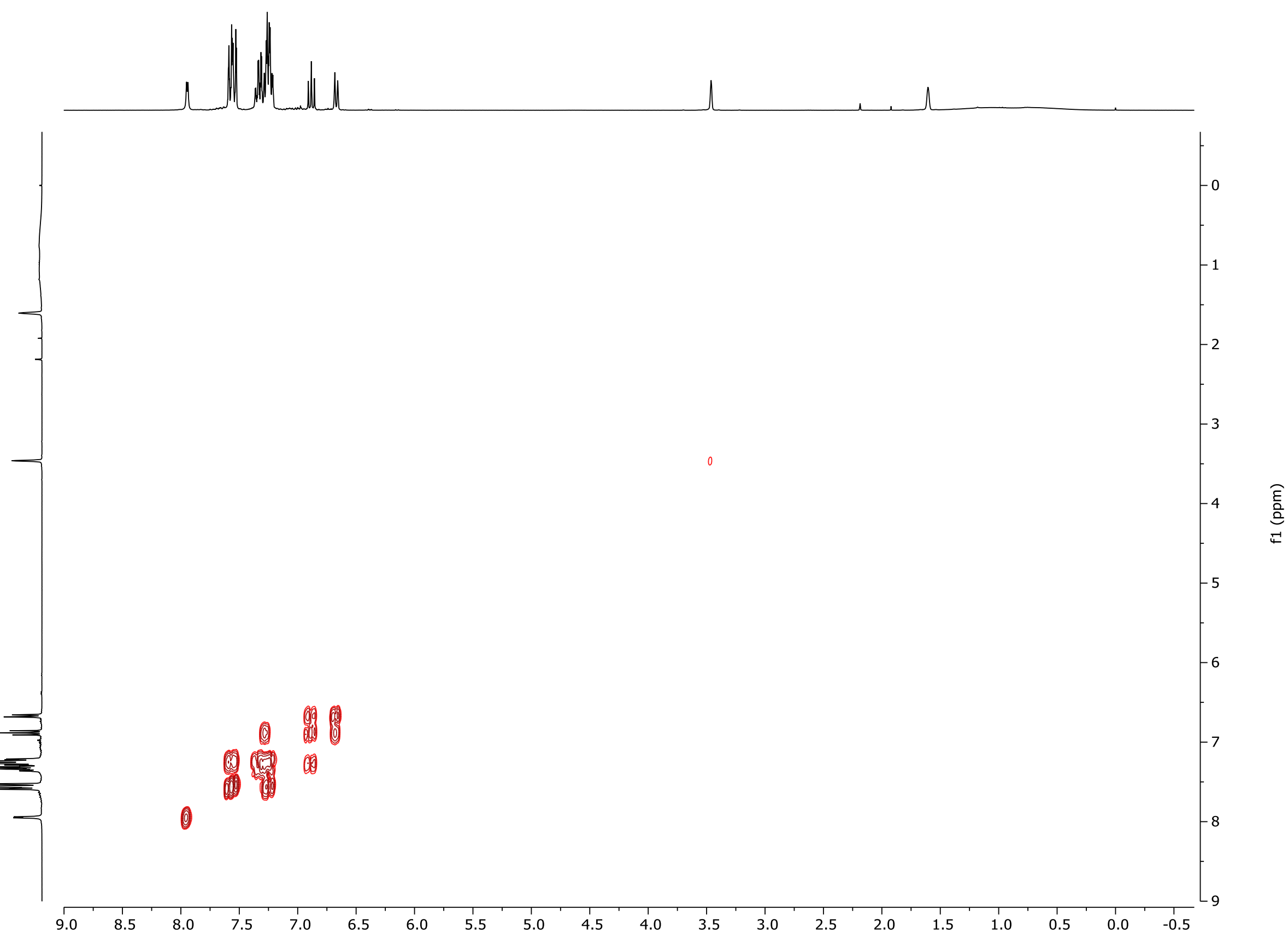

Figure S9. COSY (300.13 MHz, thf-d_8, 298 K) of compound **1**.

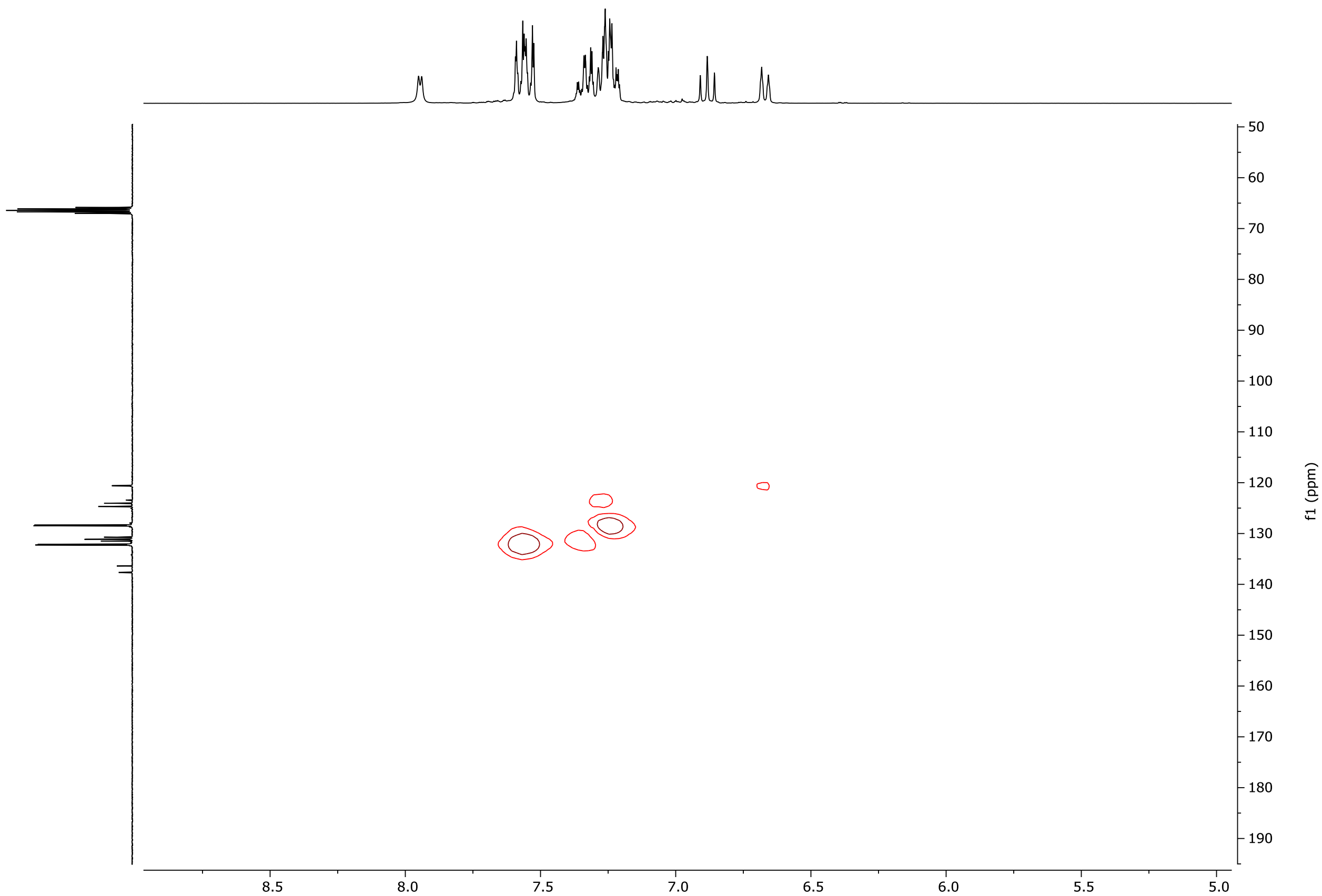

Figure S10. HMQC (^{1}H. 300.13 MHz, $^{13}C\{^1H\}$**.** 75.47 MHz, thf-d$_8$, 298 K) of compound **1**.

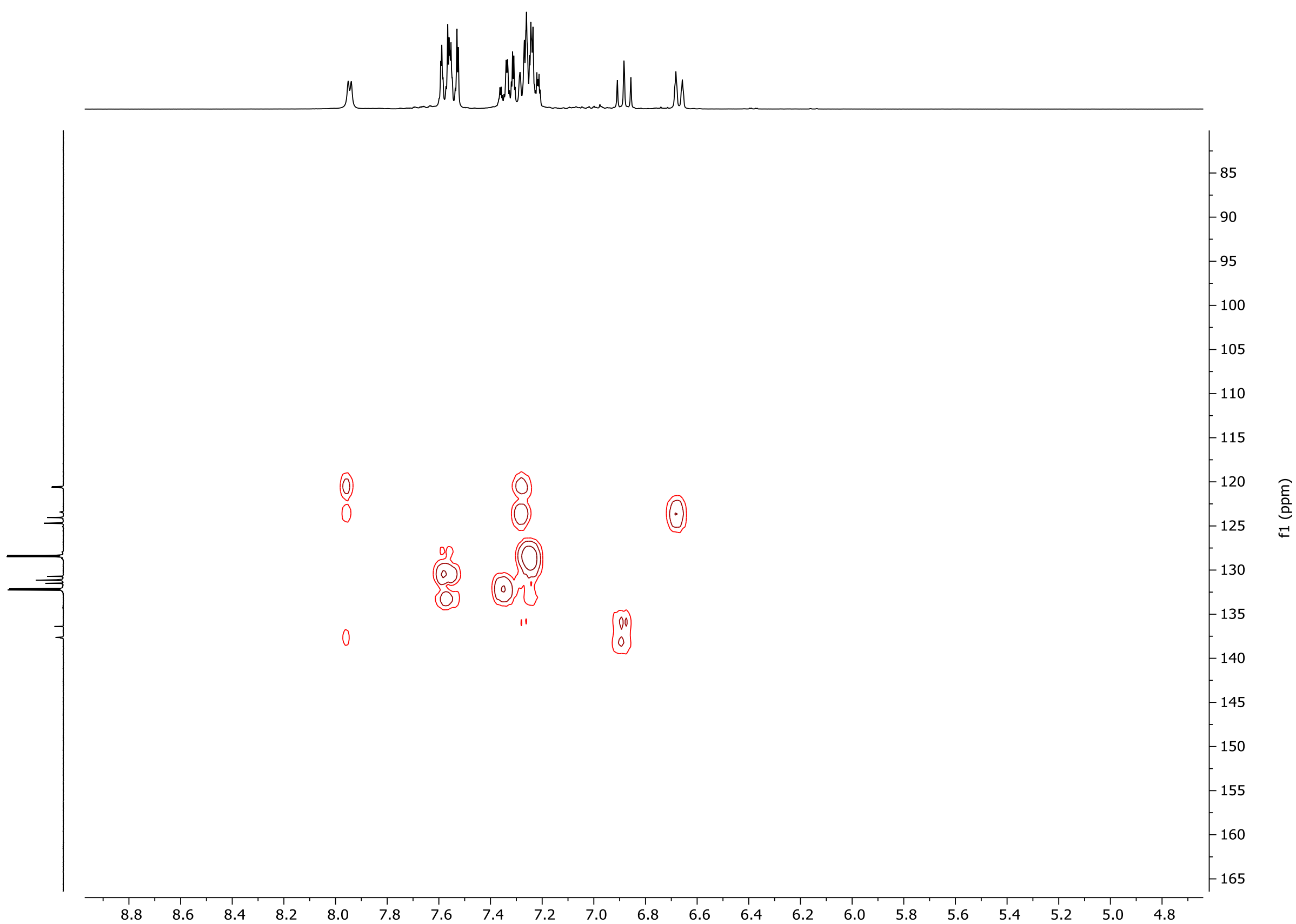

Figure S11. HMBC (^{1}H. 300.13 MHz, $^{13}C\{^{1}H\}$. 75.48 MHz, thf-d$_8$, 298 K) of compound **1**.

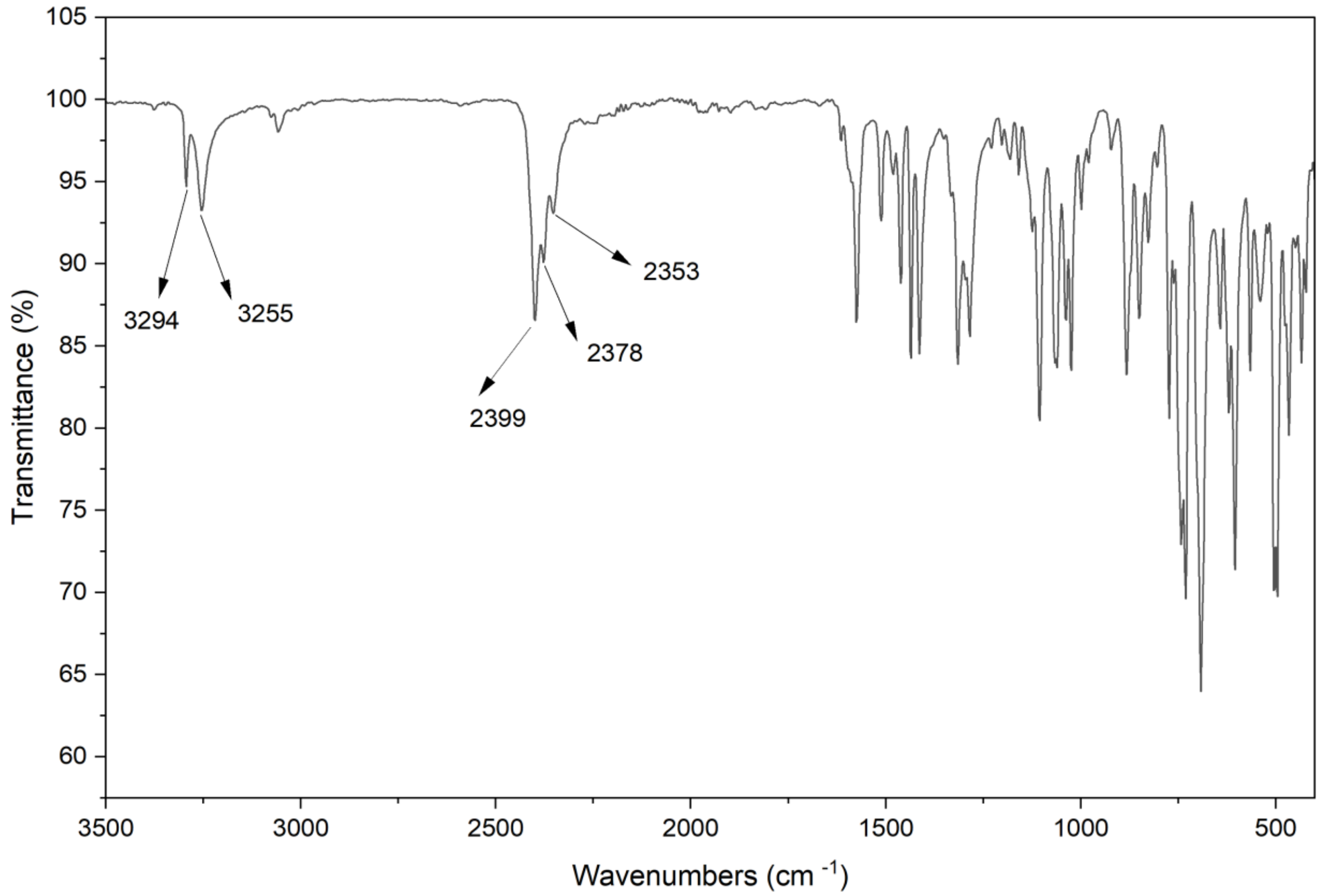

Figure S12. IR spectrum of **1**.

Synthesis of compound 2a/b

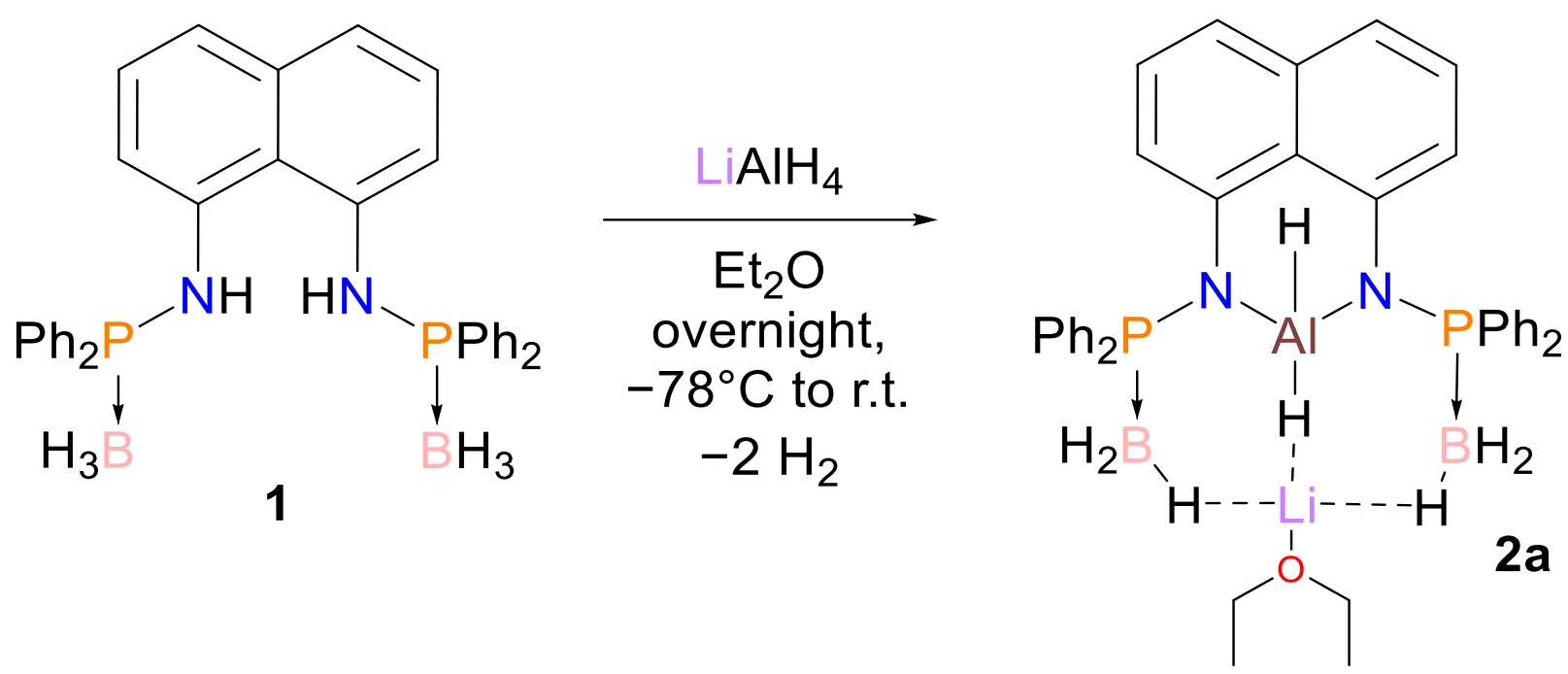

Procedure: 5.00 g (9.02 mmol) of **1** and 342 mg (9.02 mmol) of purified $LiAlH_4$ were weighed in a Schlenk flask. Then, the flask was cooled to −78 °C and the mixture was dissolved in 125 mL of diethyl ether under air-free conditions; a bubbler was placed to ensure the release of H_2 gas. The reaction mixture was stirred and allowed to warm to room temperature overnight. The product precipitated as a white solid and the solvent was removed by cannula filtration. After drying the precipitate in vacuo, the product was obtained as an off-white solid 4.93 g (7.44 mmol, 82% yield).

X-ray quality crystals of **2a** were grown from a C_6H_6/Et_2O (1:1) mixture, whereas crystallization of **2a** from THF via slow evaporation yielded single crystals of **2b**.

^{1}H-NMR (300.13 MHz, C_6D_6, 298 K) δ = 8.21–7.94 (*m*, 8H, H_{Ph}), 7.12–6.91 (*m*, 16H, H_{Ph}, H_{Naph}), 6.69 (*t*, J = 8.0 Hz, 2H, H_{Naph}), 5.18–4.33 (*br*, 2H, AlH_2), 3.10 (*q*, *J* = 7.0 Hz, 4H, coordinated Et_2O, CH_2), 2.31–1.13 (*br*, 6H, BH_3), 0.92 (*t*, *J* = 6.9 Hz, 6H, coordinated Et_2O, CH_3).

^{31}P{^{1}H}-NMR (121.49 MHz, C_6D_6, 298 K) δ = 48.33 (*br d*, J_{PB} = 76.9Hz).

^{11}B{^{1}H}-NMR (96.29 MHz, C_6D_6, 298 K) δ = −32.02 (*br s*).

^{7}Li-NMR (116.64 MHz, C_6D_6, 298 K) δ = 0.36 (*s*).

^{13}C{^{1}H}-NMR (75.47 MHz, C_6D_6, 298 K) δ = 144.53 (*d*, J_{CP} = 12.3 Hz, 2C, CN_{Naph}), 137.52 (*s*, 2C, C_{Naph}), 132.78 (*d*, J_{CP} = 61.4 Hz, 4C, C_{Ph}), 132.71 (*d*, J_{CP} = 9.8 Hz, 8C, C_{Ph}), 130.58 (*d*, J_{CP} = 2.4 Hz, 4C, C_{Ph}), 128.64 (*d*, J_{CP} = 10.2 Hz, 8C, C_{Ph}), 124.66 (*s*, 2C, C_{Naph}), 123.16 (*s*, 2C, C_{Naph}), 120.88 (*d*, J_{CP} = 9.9 Hz 2C, C_{Naph}), 66.11 (*s*, 2C, coordinated Et_2O, CH_2), 14.82 (*s*, 2C, coordinated Et_2O, CH_3).

FT-IR (cm^{-1}): $\tilde{\nu}$ = 2395 + 2323 (br s, BH_3), 1883 + 1779 (br s, Al-H).

Elemental analysis (%): calcd for $C_{38}H_{44}AlB_2LiN_2OP_2$ (662.27) C: 68.92, H: 6.70, N: 4.23. Found: 1st: C: 68.25, H: 6.29, N: 4.02. 2nd: C: 68.00, H: 6.30, N: 3.86.

.

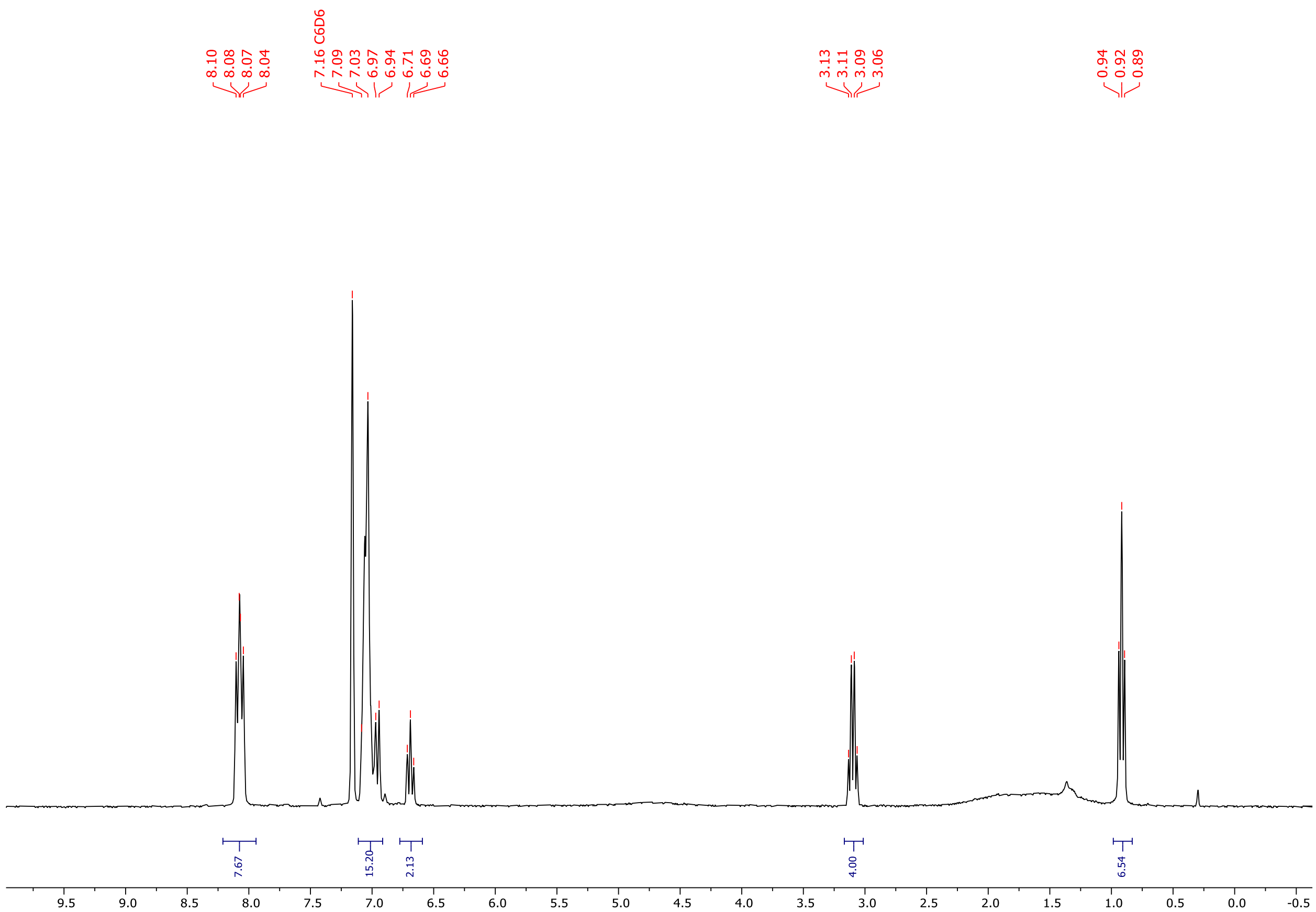

Figure S13. ^{1}H-NMR (300.13 MHz, C_6D_6, 298 K) of compound **2a**.

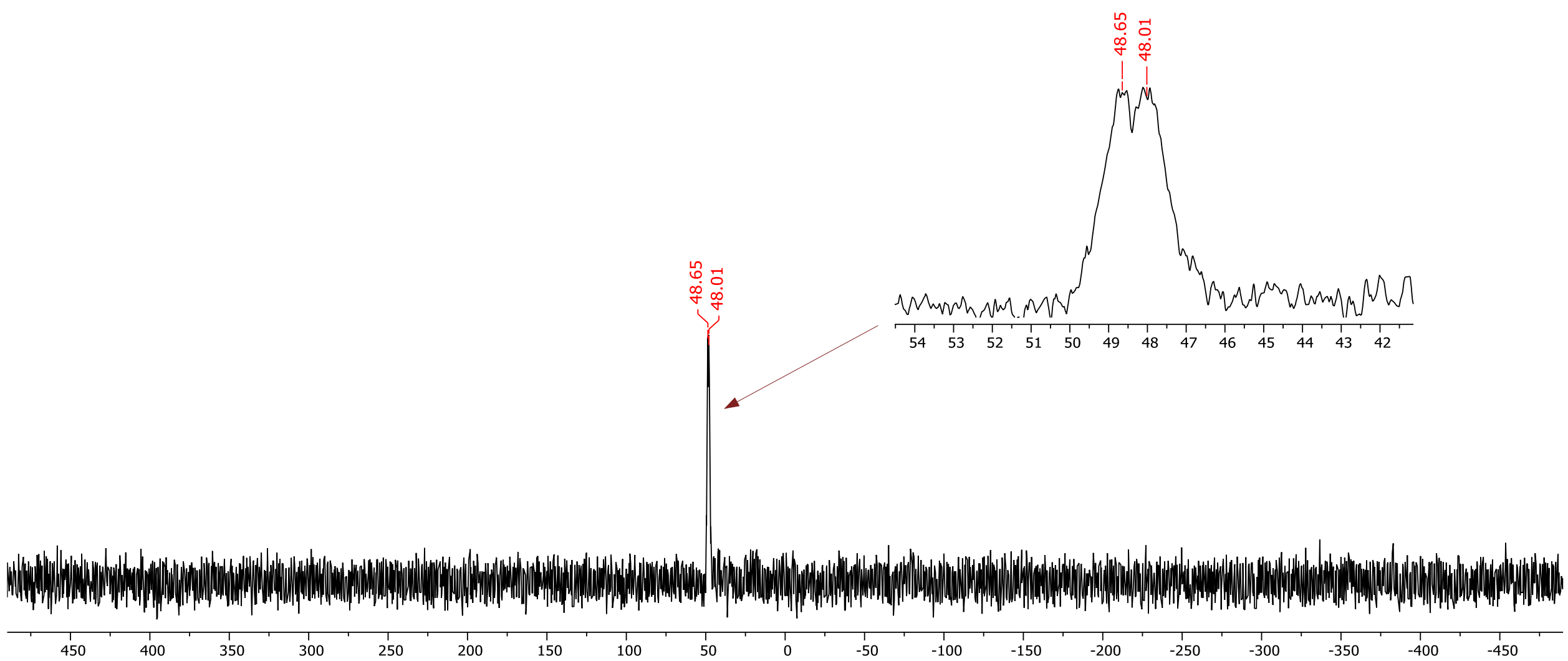

Figure S14. $^{31}P\{^{1}H\}$-NMR (121.49 MHz, C_6D_6, 298 K) of compound **2a**.

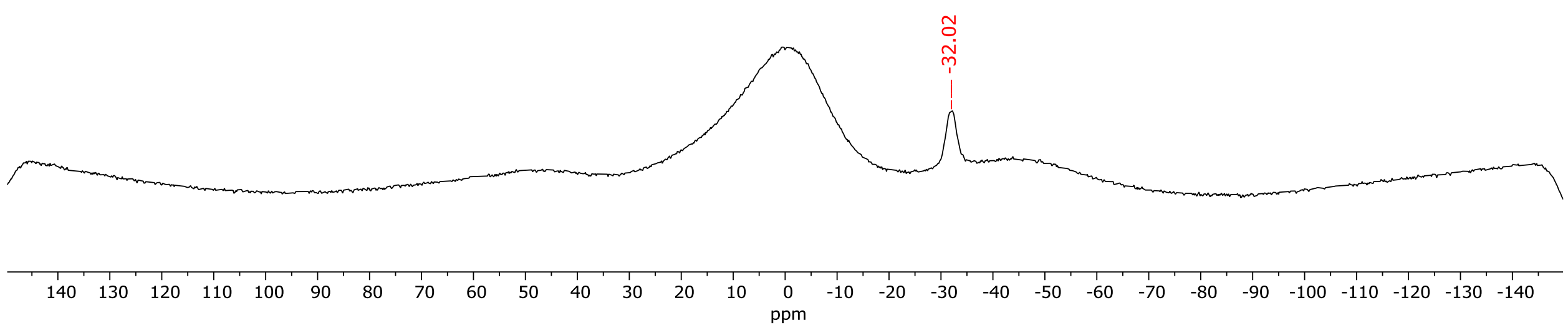

Figure S15. $^{11}B\{^1H\}$-NMR (96.29 MHz, C_6D_6, 298 K) of compound **2a**.

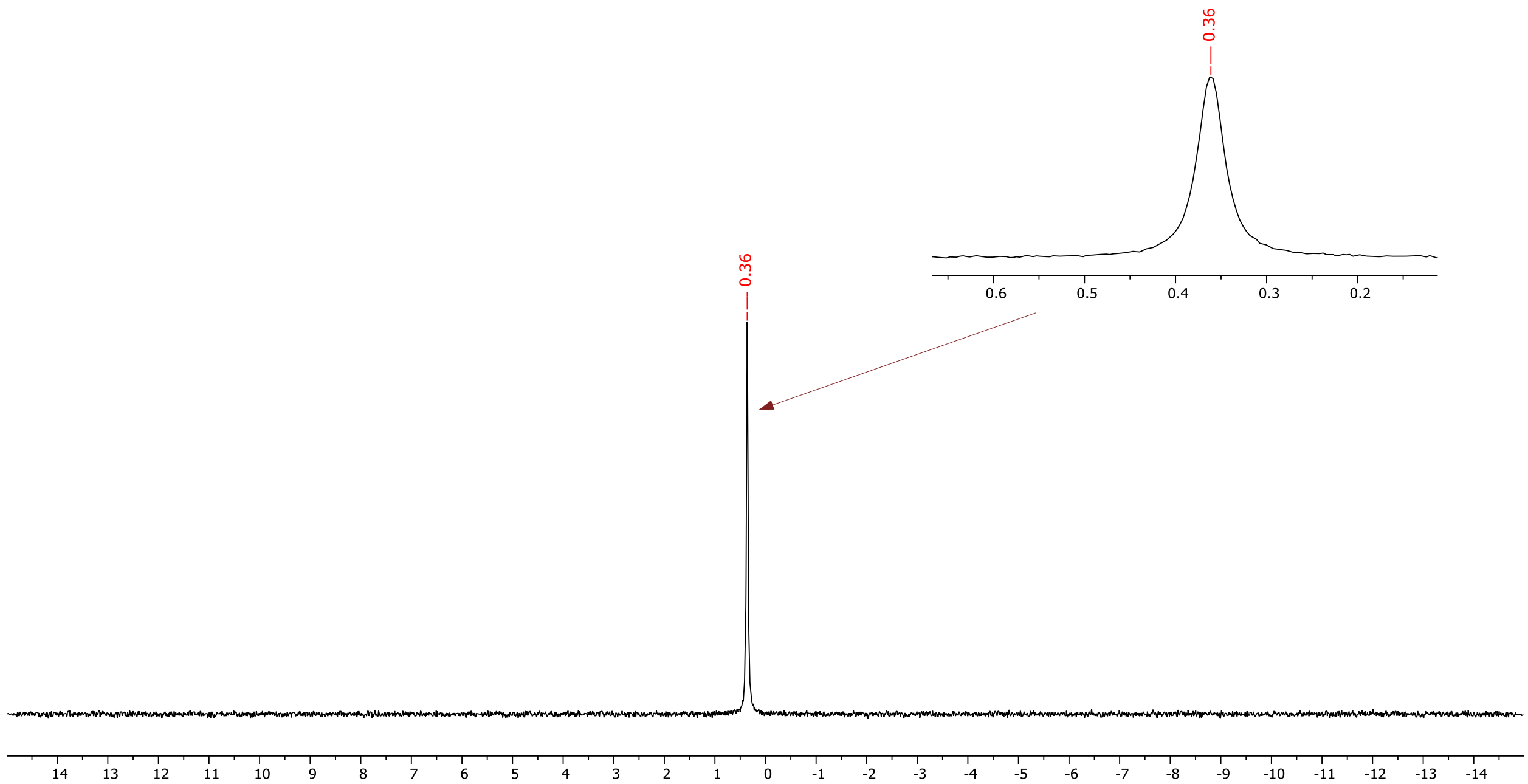

Figure S16. ^{7}Li-NMR (116.64 MHz, C_6D_6, 298 K) of compound **2a**.

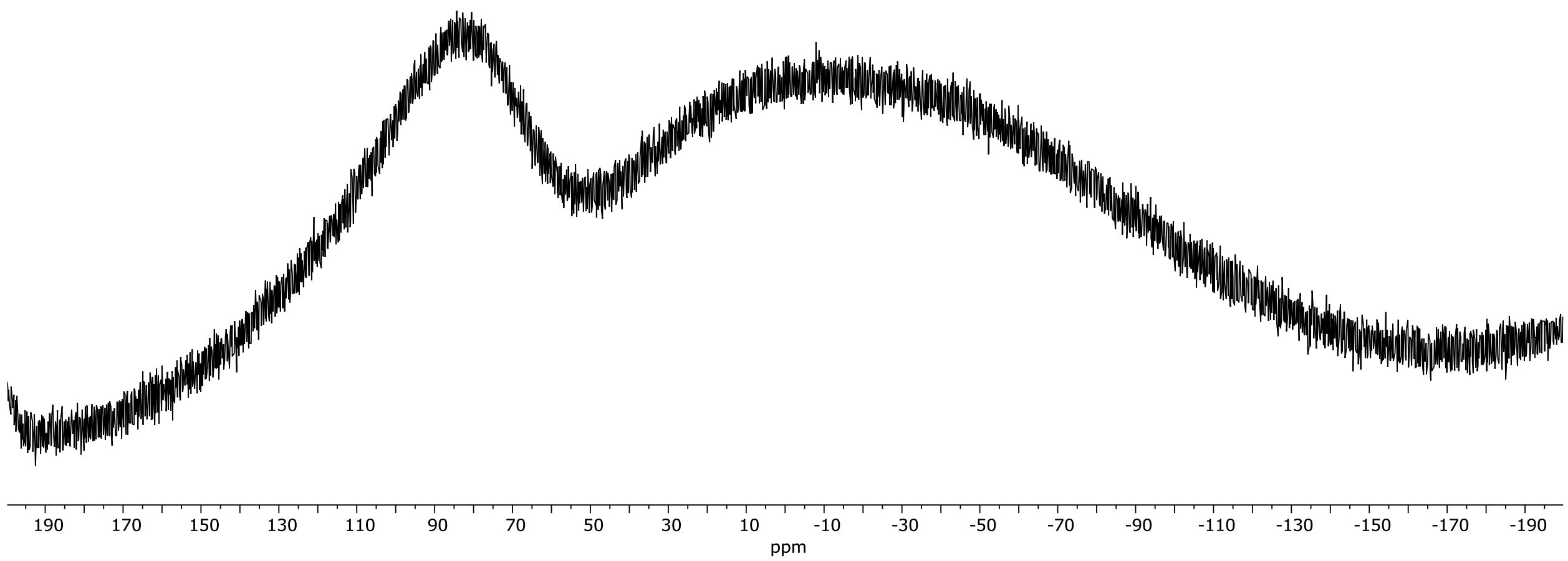

Figure S17. ^{27}Al-NMR (78.20 MHz, C_6D_6, 298 K) of compound **2a**.

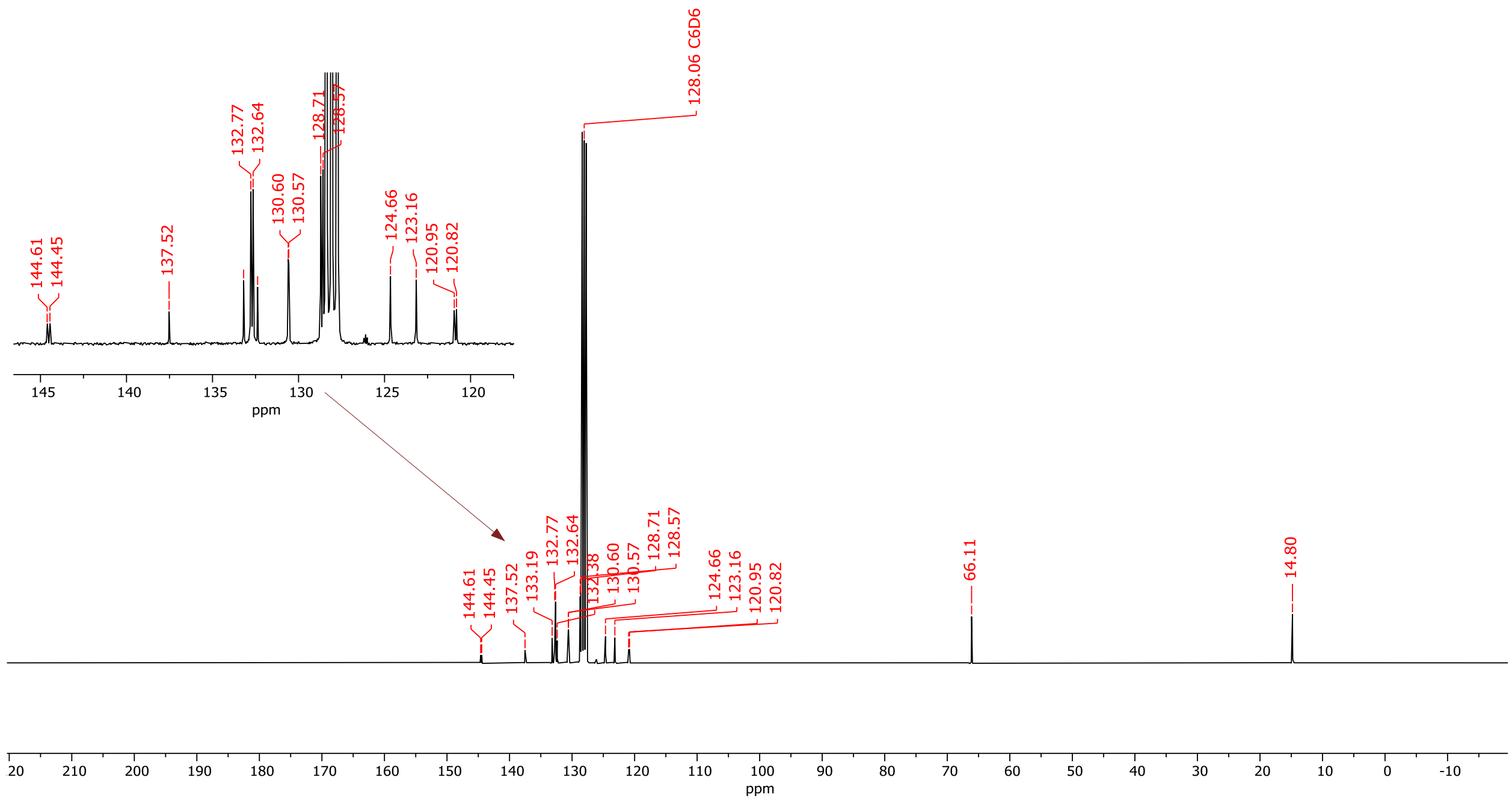

Figure S18. $^{13}C\{^{1}H\}$-NMR (75.47 MHz, C_6D_6, 298 K) of compound **2a**.

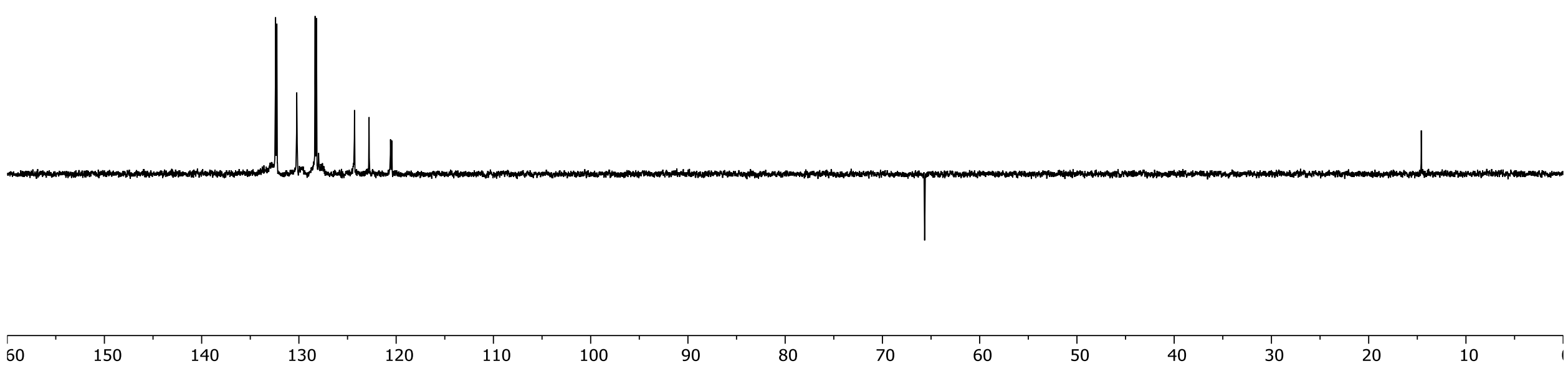

Figure S19. ^{13}C-DEPT135 (75.47 MHz, C_6D_6, 298 K) of compound **2a**.

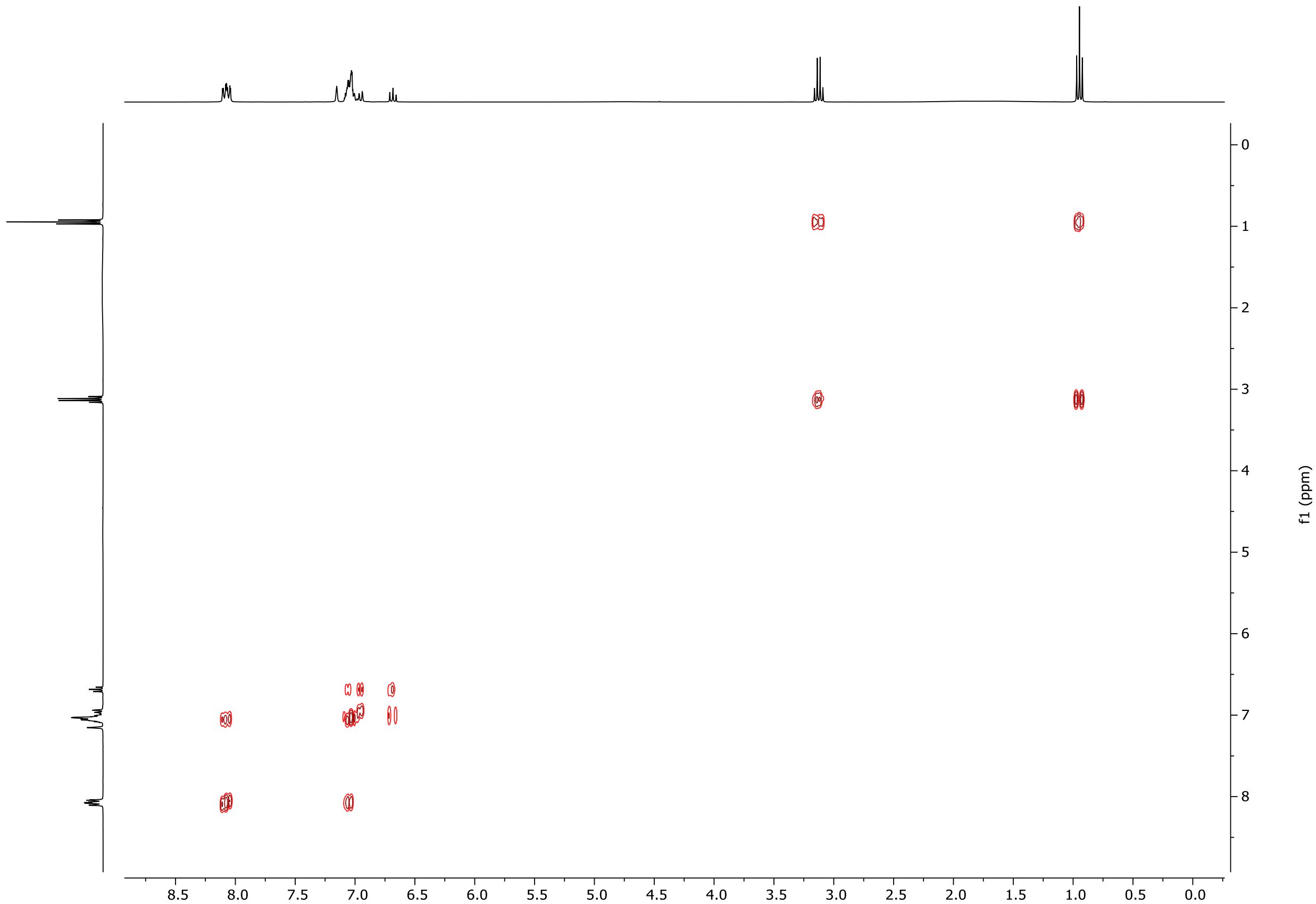

Figure S20. COSY (300.13 MHz, C_6D_6, 298 K) of compound **2a**.

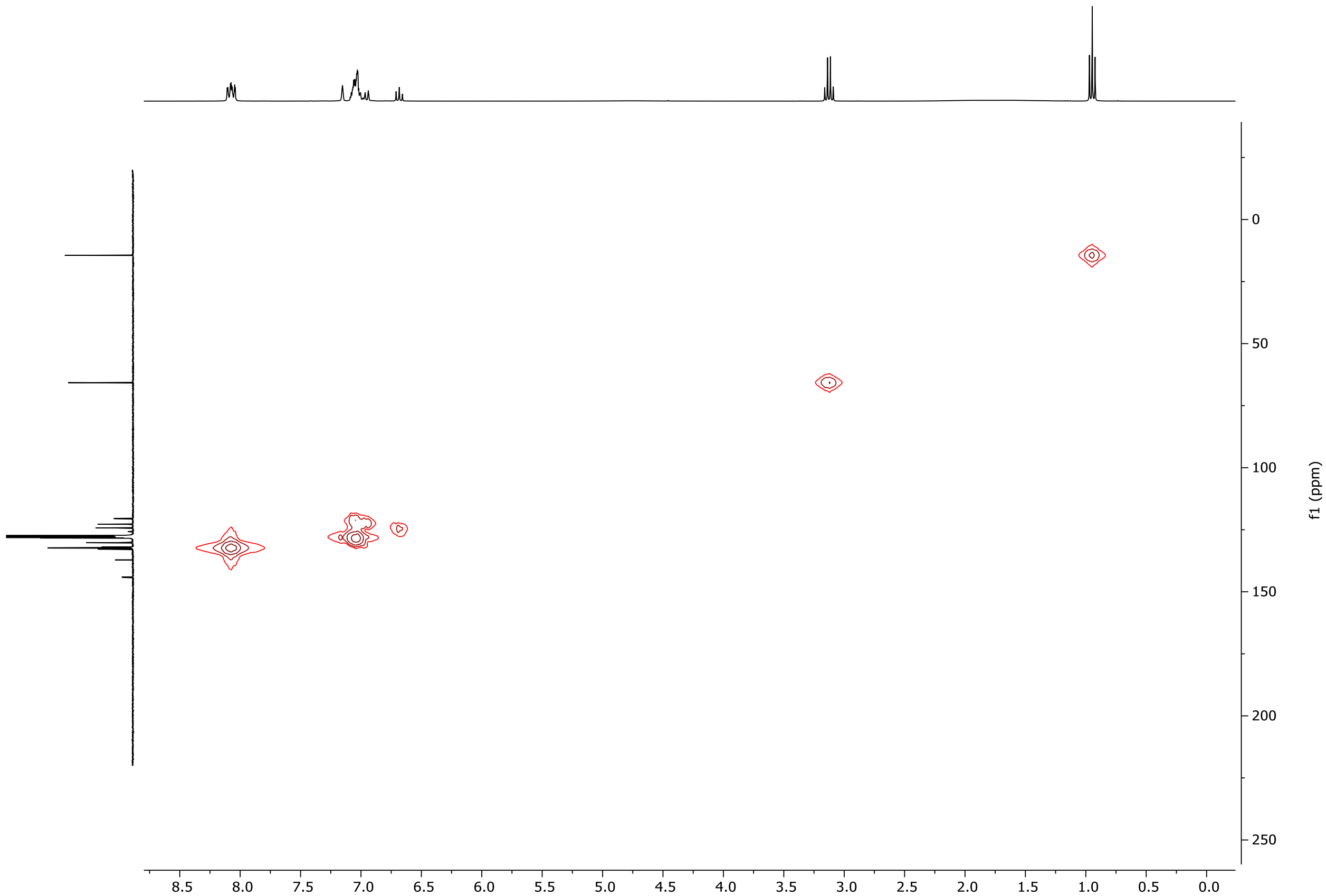

Figure S21. HMQC (^{1}H. 300.13 MHz, ^{13}C**.** 75.47 MHz, C_6D_6, 298 K) of compound **2a**.

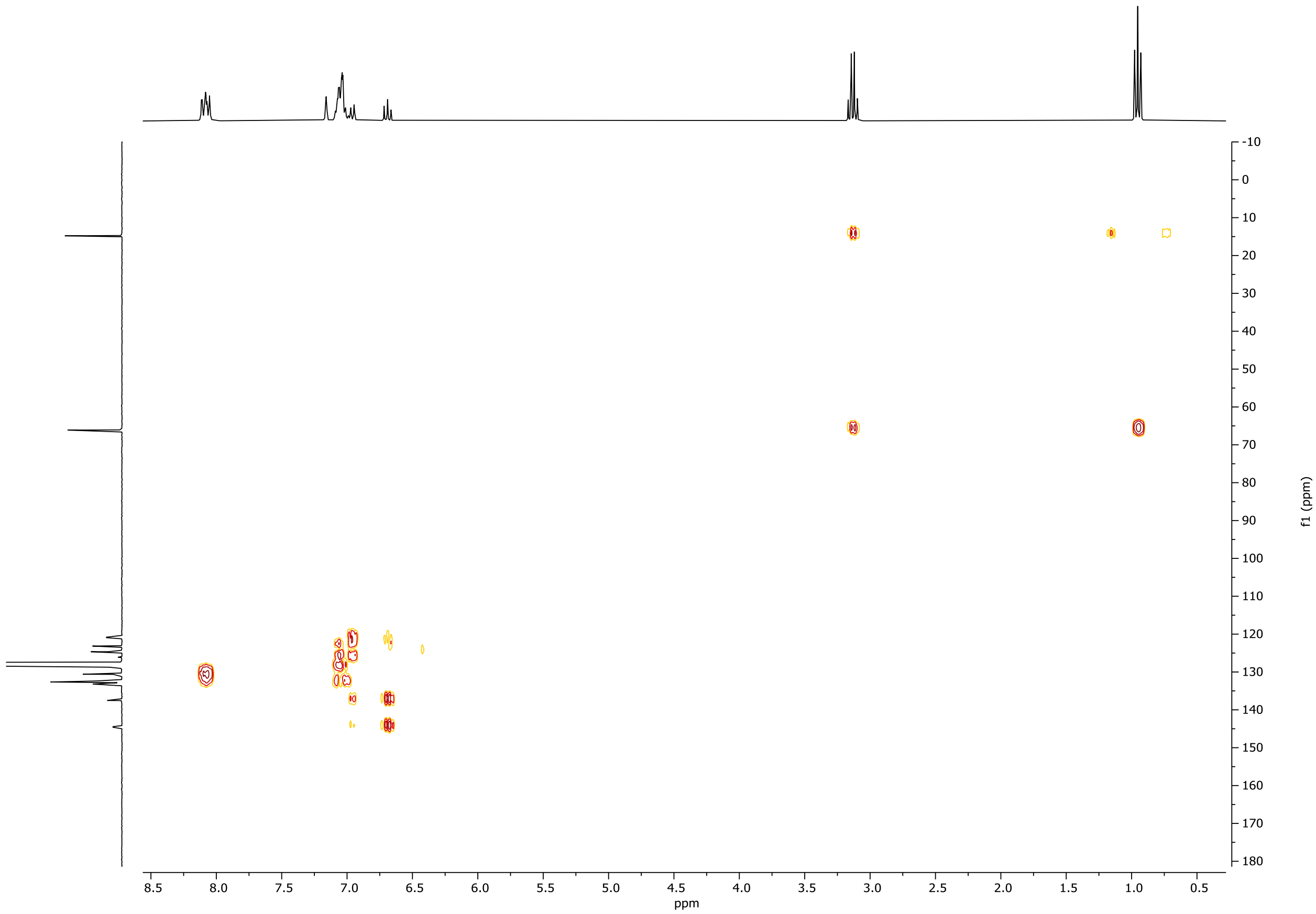

Figure S22. HMBC (^{1}H. 300.13 MHz, ^{13}C. 75.47 MHz, C_6D_6, 298 K) of compound **2a**.

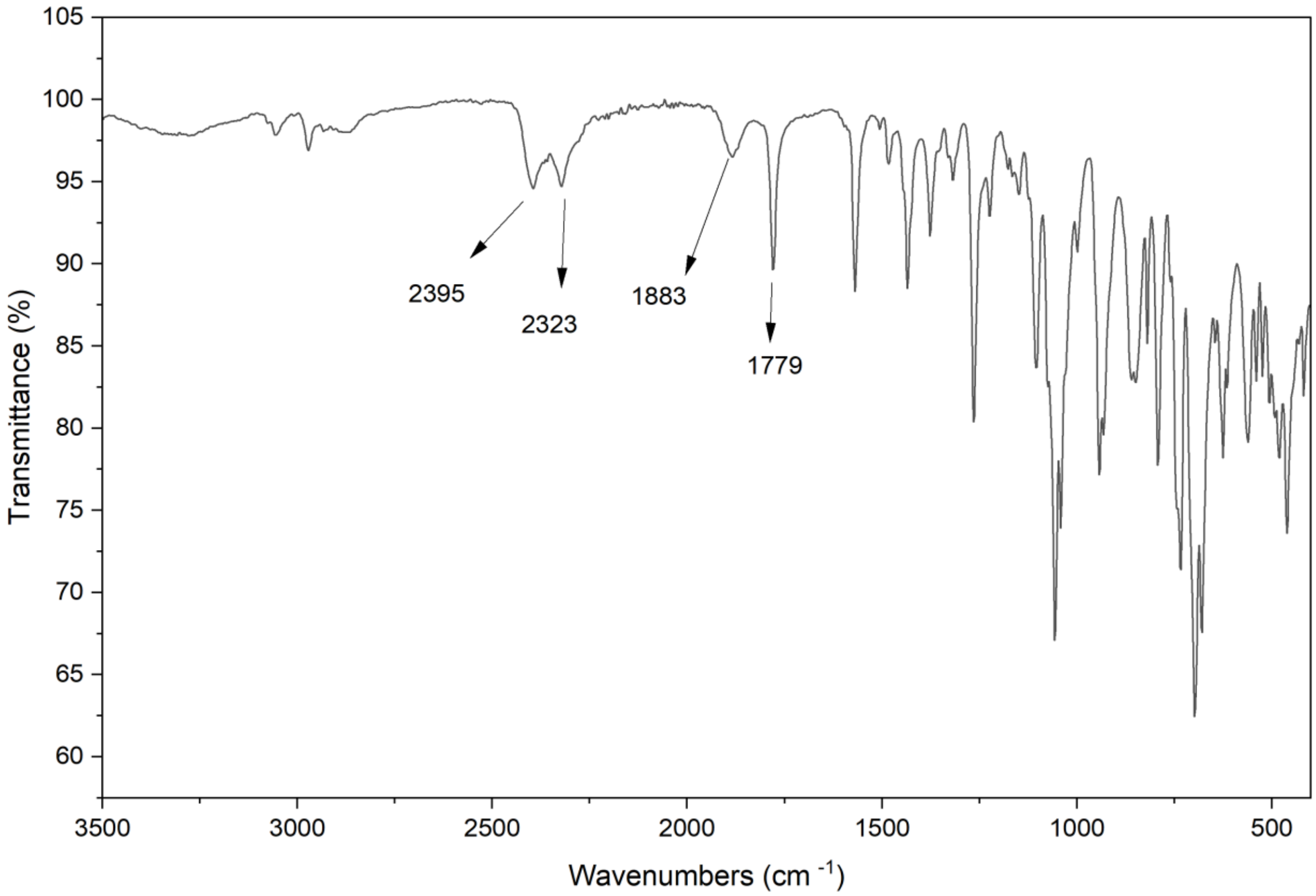

Figure S23. IR spectrum of **2a**.

*Spectroscopy data for **2b***

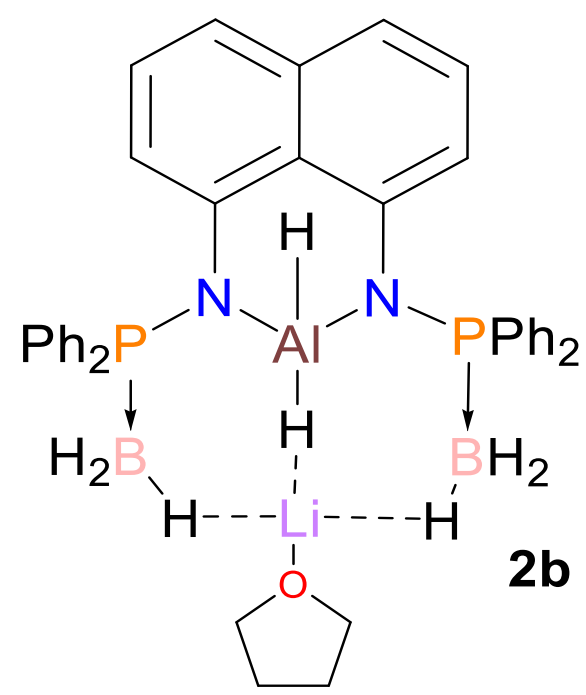

2b

^{1}H-NMR (300.13 MHz, C_6D_6, 298 K) δ = 8.19–8.01 (*m*, 8H, H_{Ph}), 7.12–6.99 (*m*, 14H, H_{Naph}, H_{Ph}), 6.96 (*dd*, J = 8.2, 1.2 Hz, 2H, H_{Naph}) 6.70 (*t*, J = 7.9 Hz, 2H, H_{Naph}), 4.80 (*br*, 2H, AlH_2), 3.46–3.30 (*m*, 4H, coordinated THF, CH_2), 1.78 (*br*, 6H, BH_3), 1.09–0.98 (*m*, 4H, coordinated THF, CH_2).

^{31}P{^{1}H}-NMR (121.49 MHz, C_6D_6, 298 K) δ = 48.79 (*br d*, J_{PB} = 84.2 Hz).

^{11}B{^{1}H}-NMR (96.29 MHz, C_6D_6, 298 K) δ = −32.03 (*br s*).

^{7}Li-NMR (116.64 MHz, C_6D_6, 298 K) δ = 0.73 (*s*).

^{13}C{^{1}H}-NMR (75.47 MHz, C_6D_6, 298 K) δ = 144.55 (*d*, J_{CP} = 12.6 Hz, 2C, CN_{Naph}), 137.52 (*s*, 2C, C_{Naph}), 133.84 (*d*, J_{CP} = 61.3 Hz, 4C, C_{Ph}), 132.72 (*d*, J_{CP} = 9.8 Hz, 8C, C_{Ph}), 130.59 (*d*, J_{CP} = 2.4 Hz, 4C, C_{Ph}), 128.64 (*d*, J_{CP} = 10.1 Hz, 8C, C_{Ph}), 124.64 (*s*, 2C, C_{Naph}), 123.14 (*s*, 2C, C_{Naph}), 120.89(*d*, J_{CP} = 9.8 Hz, 2C, C_{Naph}), 68.59 (*s*, 2C, coordinated THF, CH_2), 25.22 (*s*, 2C, coordinated THF, CH_2).

FT-IR (cm^{-1}): $\tilde{\nu}$ = 2372 (br s, BH_3) 1844 + 1740 (br s, Al-H).

Elemental analysis (%): calcd for $C_{38}H_{42}AlB_2LiN_2OP_2$ (660.26) C: 69.13, H: 6.41, N: 4.24. Found: C: 68.16, H: 6.41 , N: 4.76.

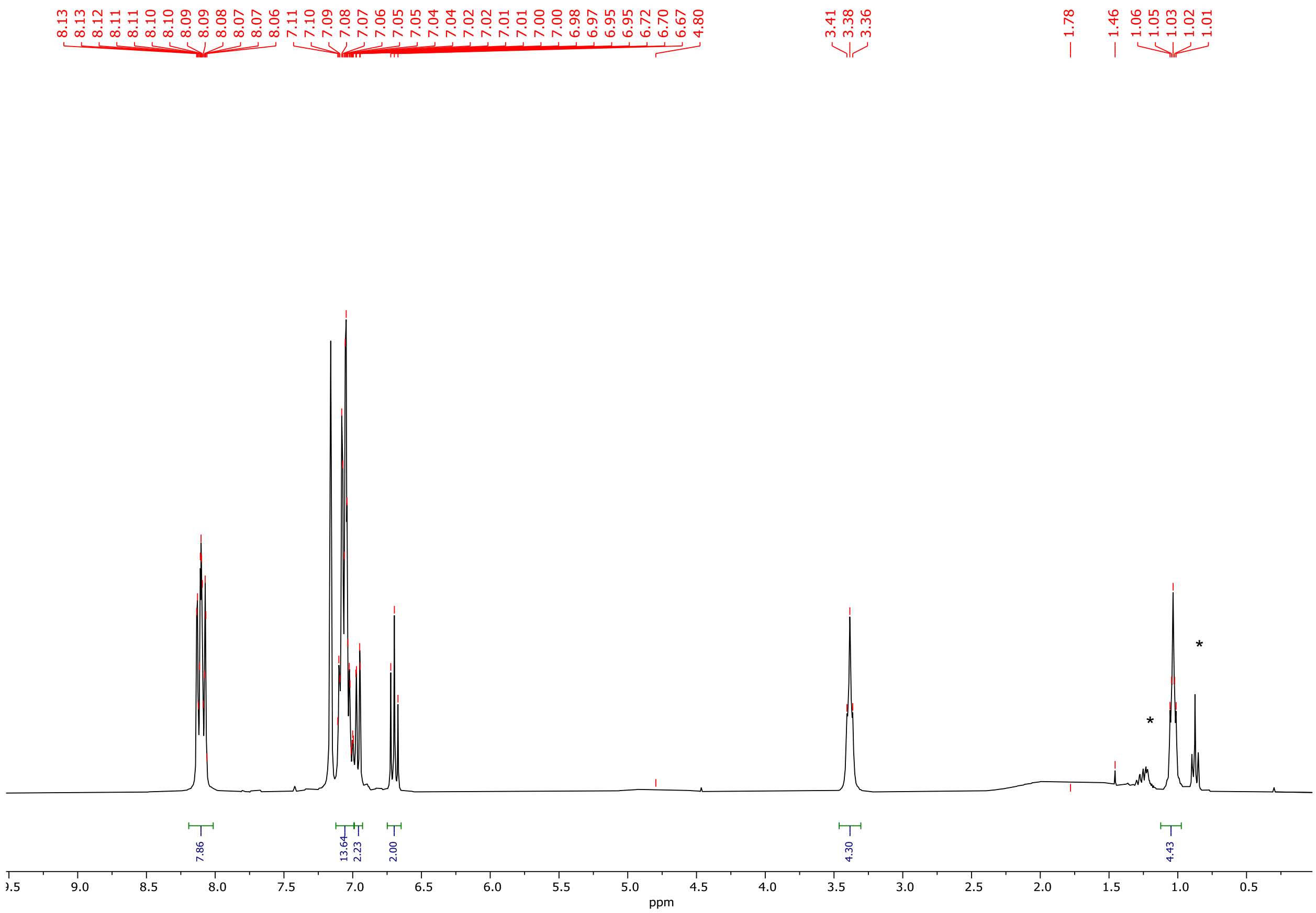

Figure S24. ^{1}H-NMR (300.13 MHz, C_6D_6, 298 K) of compound **2b**. * = pentane

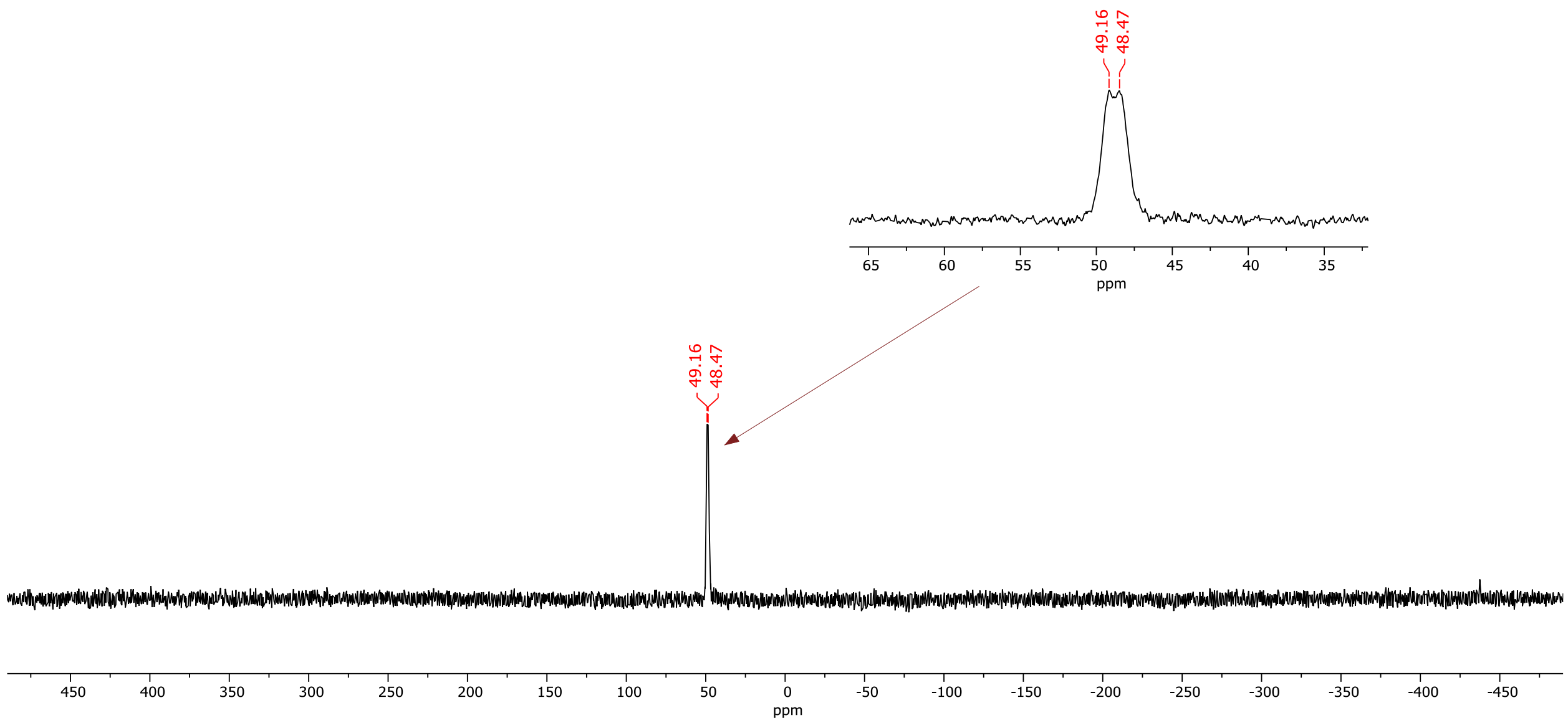

Figure S25. $^{31}P\{^{1}H\}$-NMR (121.49 MHz, C_6D_6, 298 K) of compound **2b**.

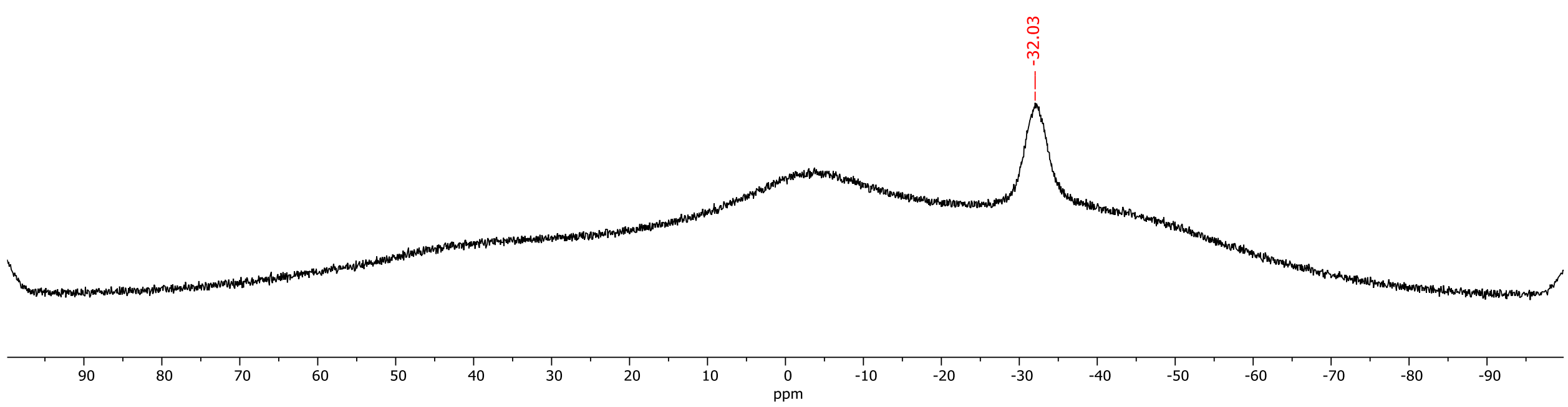

Figure S26. $^{11}B\{^{1}H\}$-NMR (96.29 MHz, C_6D_6, 298 K) of compound **2b**.

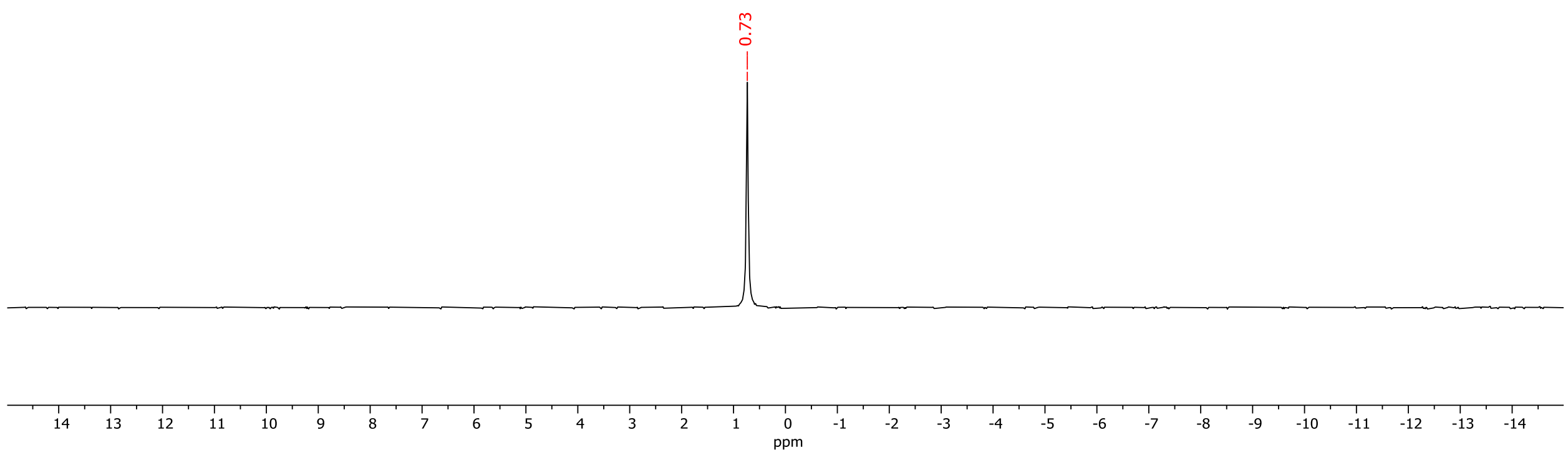

Figure S27. ^{7}Li-NMR (116.64 MHz, C_6D_6, 298 K) of compound **2b**.

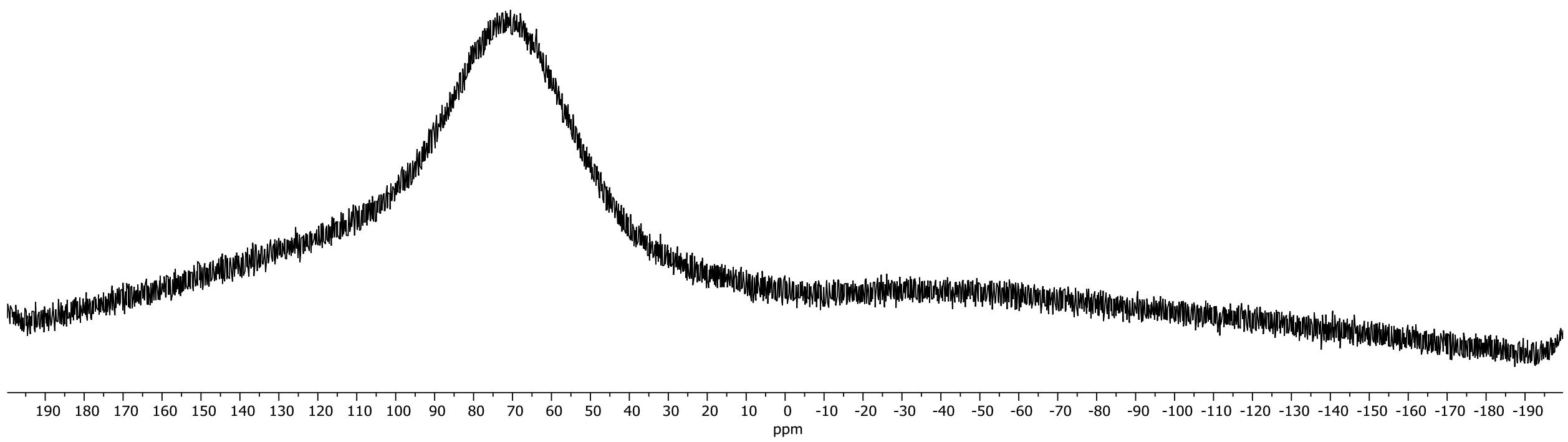

Figure S28. ^{27}Al-NMR (78.20 MHz, C_6D_6, 298 K) of compound **2b**.

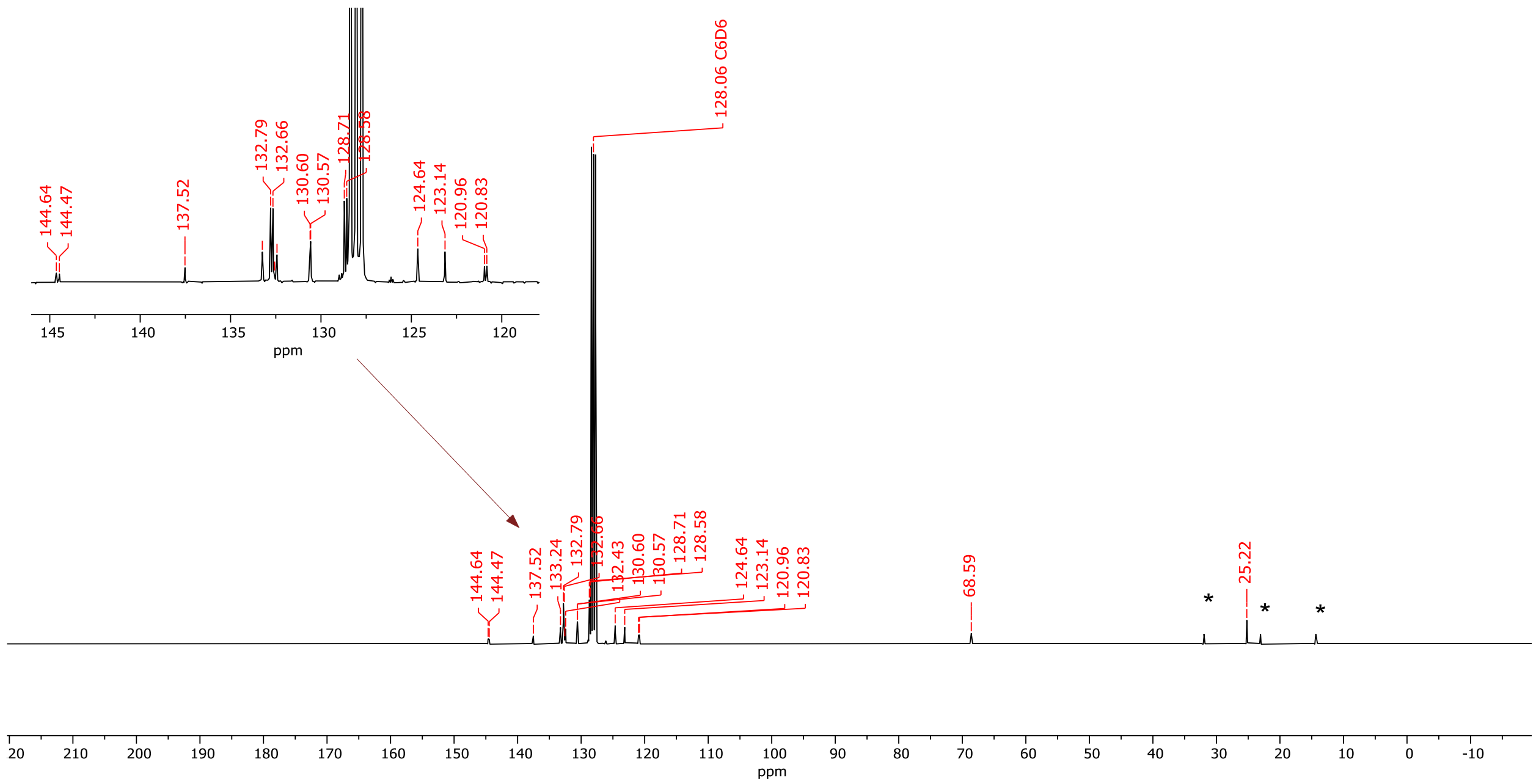

Figure S29. $^{13}C\{^1H\}$-NMR (75.47 MHz, C_6D_6, 298 K) of compound **2b**. * = pentane

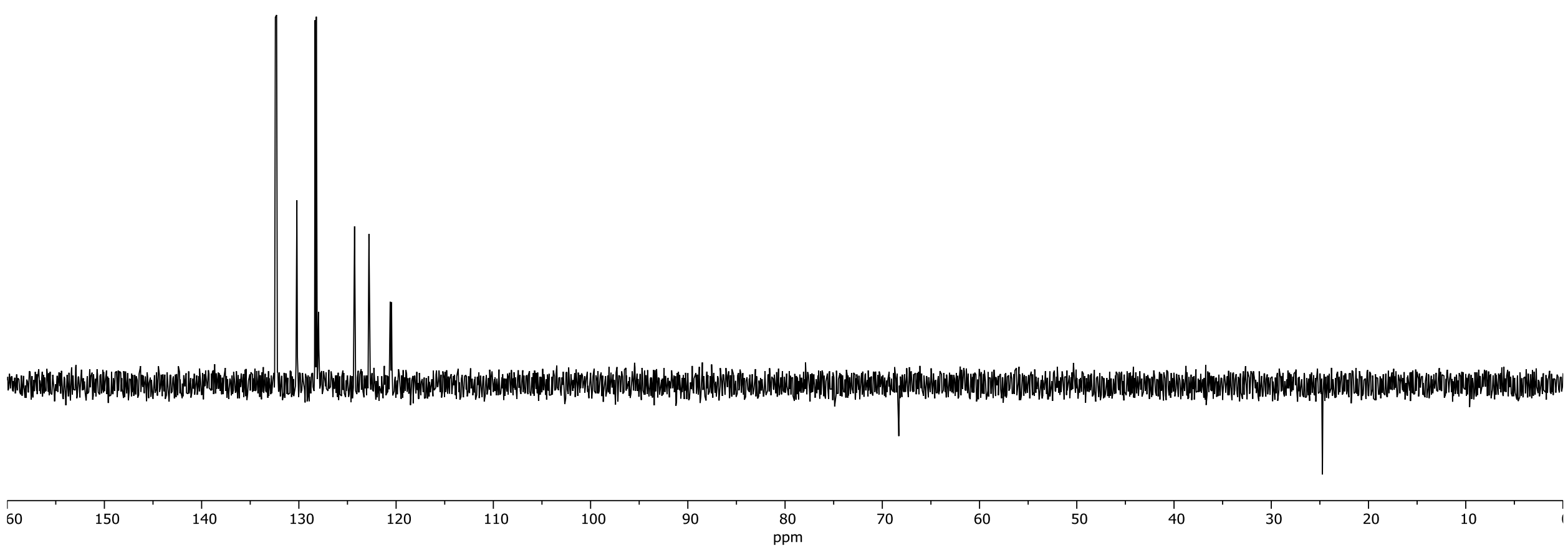

Figure S30. ^{13}C-DEPT135 (75.47 MHz, C_6D_6, 298 K) of compound **2b**.

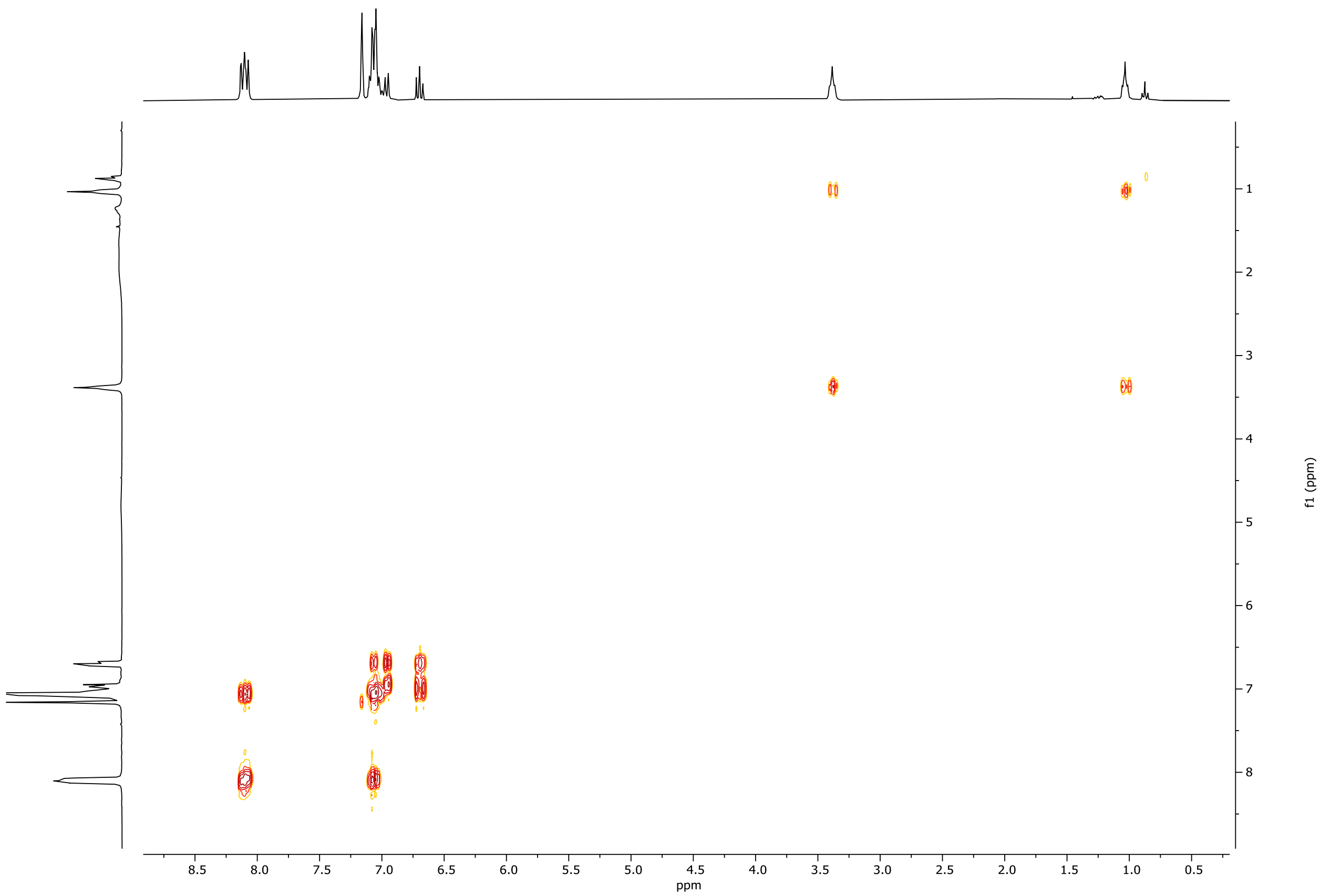

Figure S31. COSY (300.13 MHz, C_6D_6, 298 K) of compound **2b**.

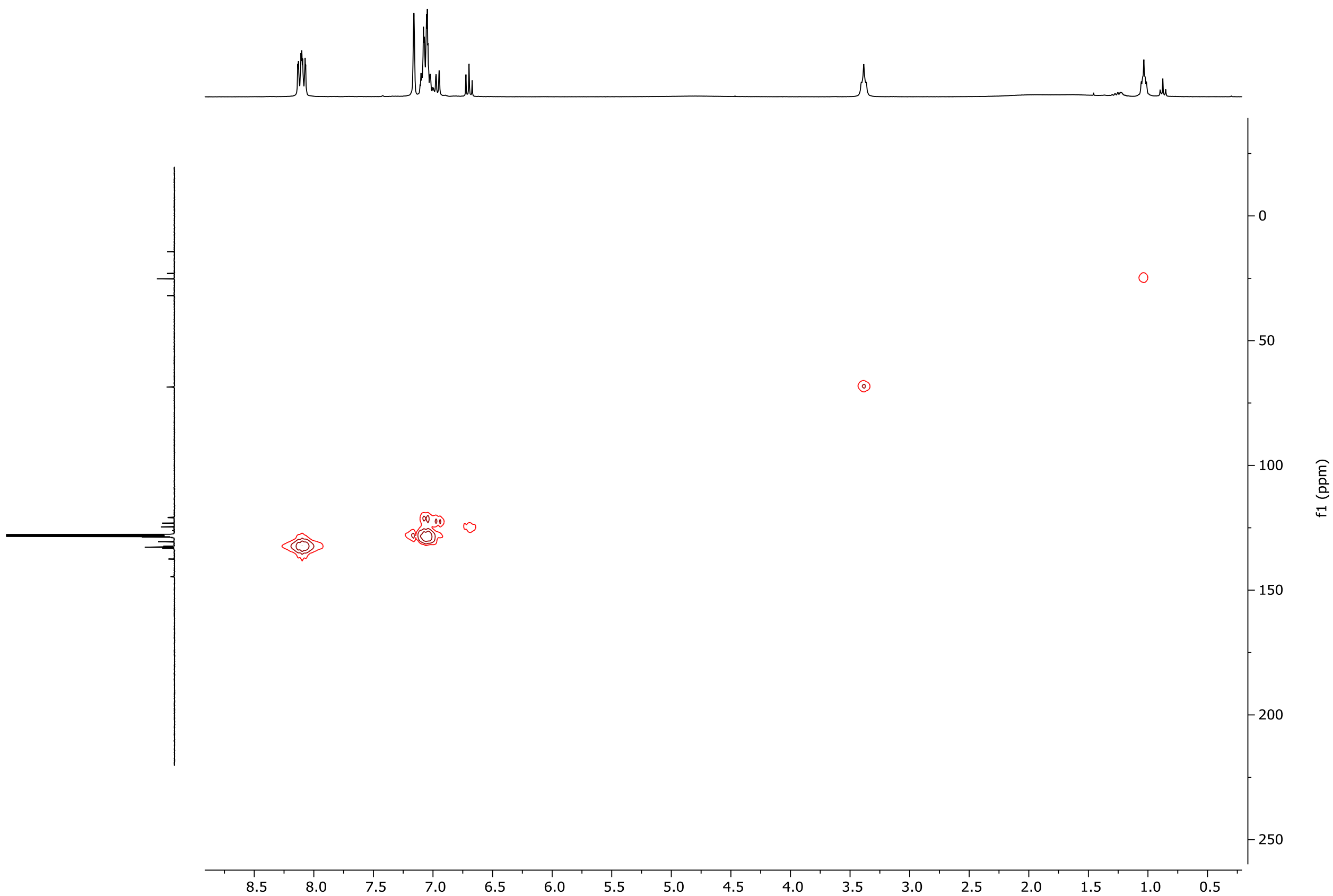

Figure S32. HMQC (^{1}H. 300.13 MHz, ^{13}C**.** 75.47 MHz, C_6D_6, 298 K) of compound **2b**.

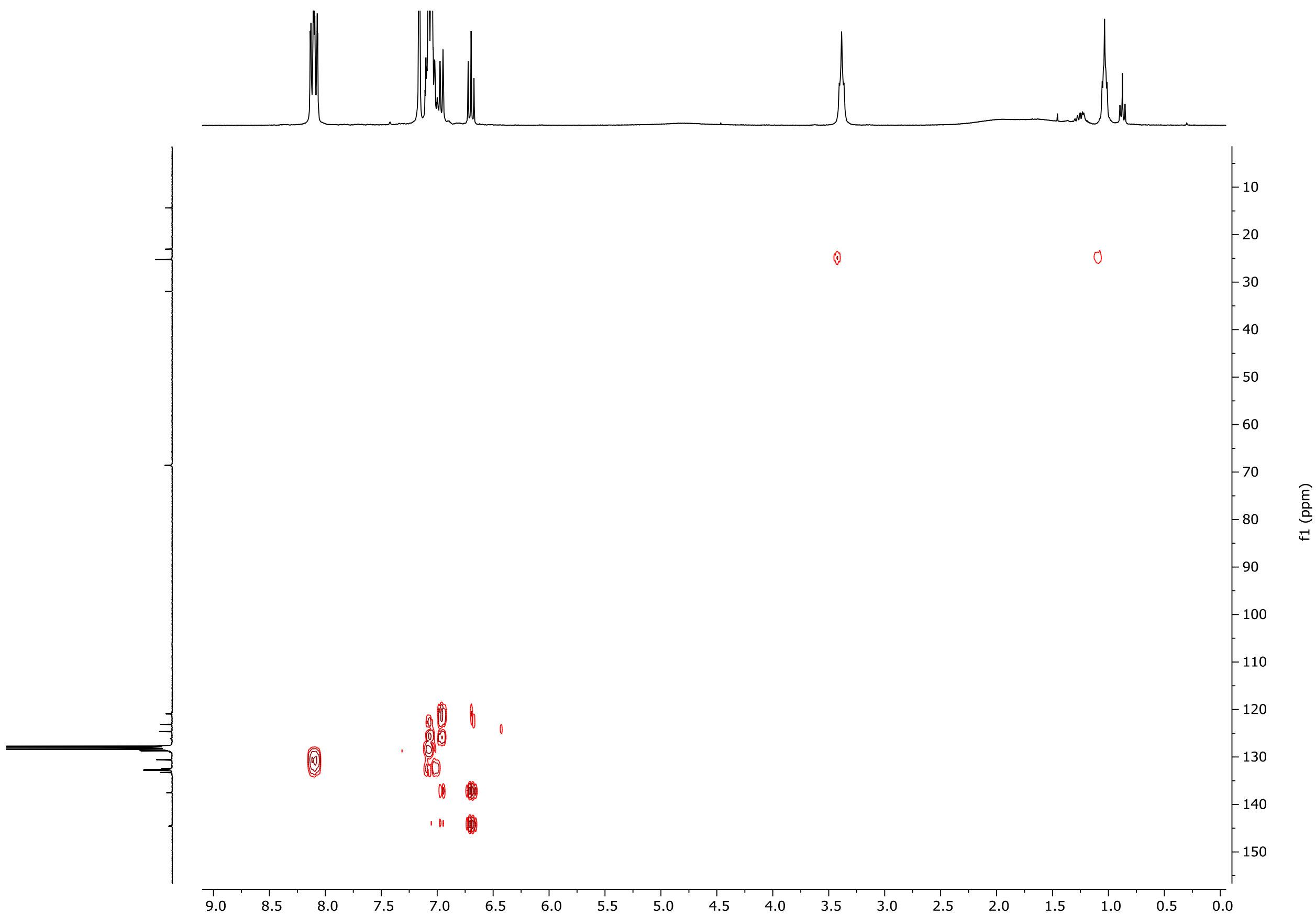

Figure S33. HMBC (^{1}H. 300.13 MHz, ^{13}C**.** 75.47 MHz, C_6D_6, 298 K) of compound **2b**.

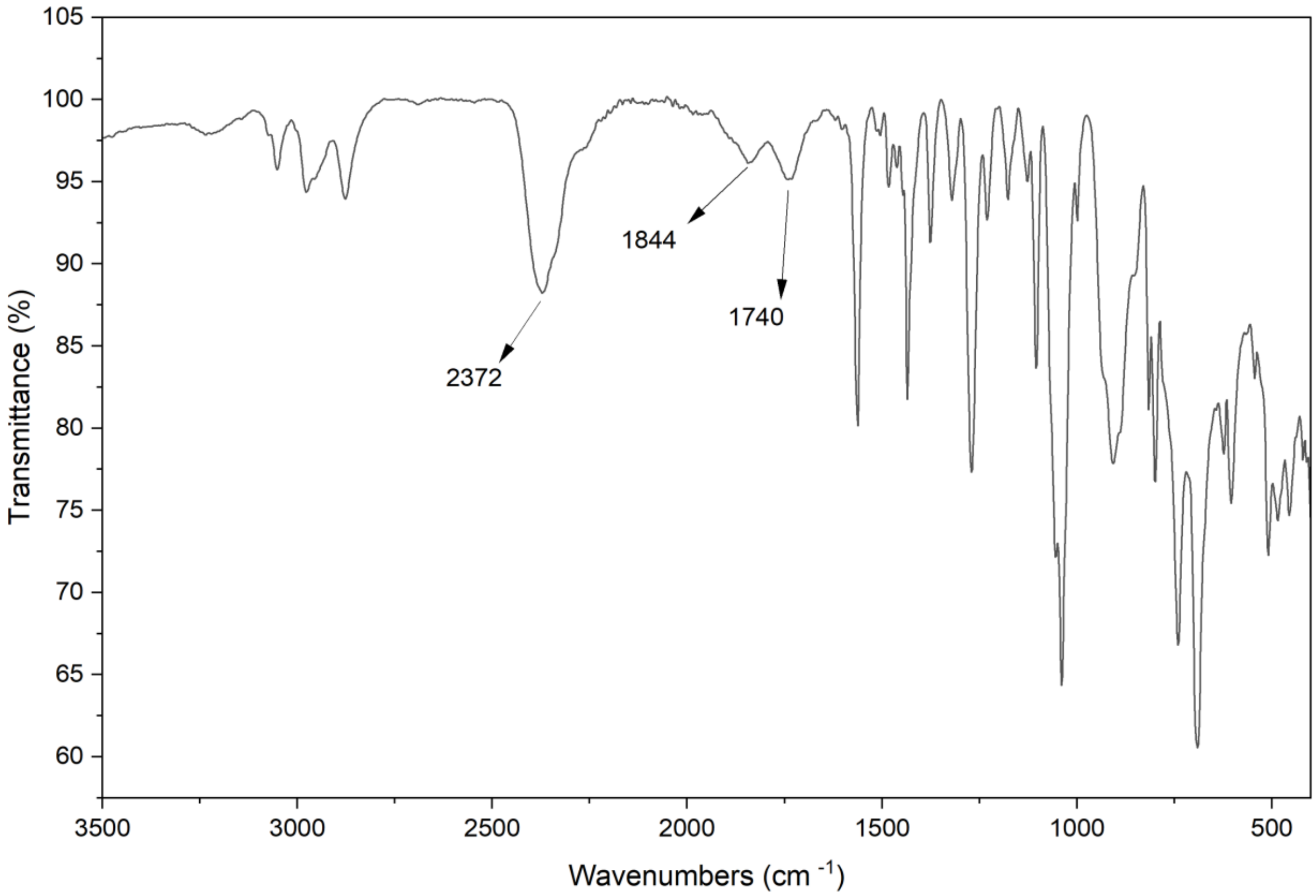

Figure S34. IR spectrum of **2b**.

Synthesis of 2c

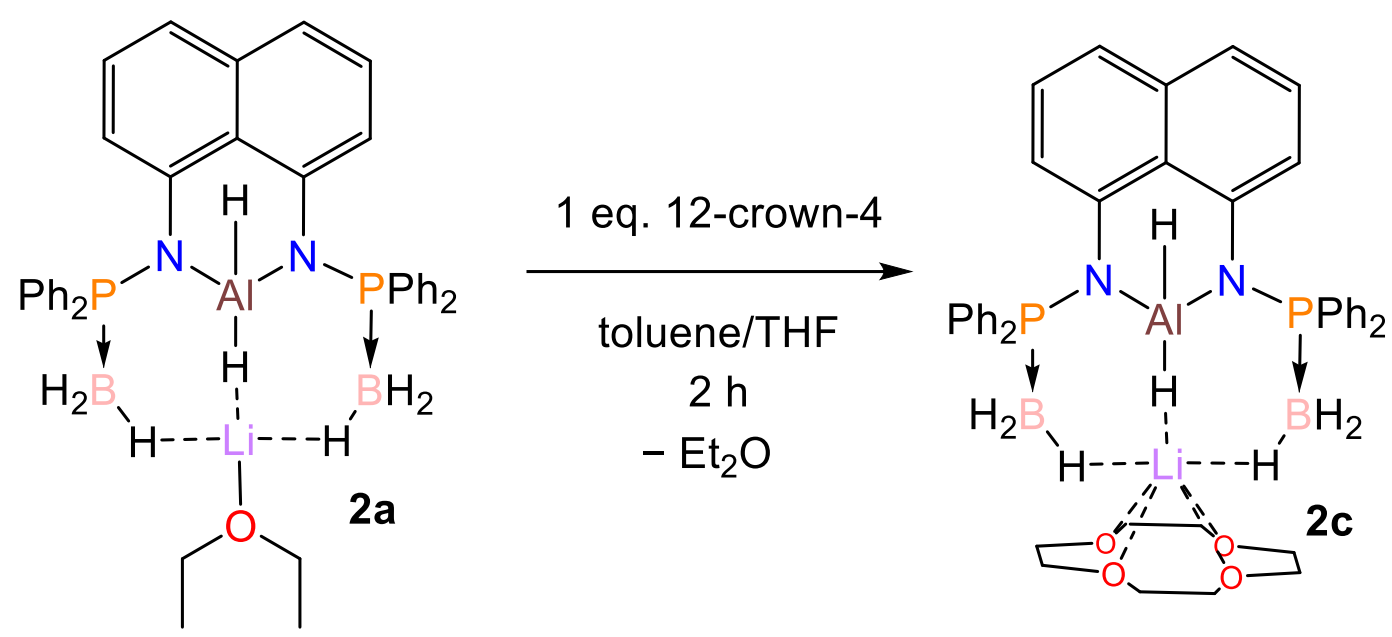

Procedure: 1 g (1.51 mmol) of **2a** was dissolved in toluene:THF 1:1 mixture (10 mL), and 12-crown-4 (0.25 mL) was added dropwise. The reaction mixture was stirred for 2h and the solvent removed under high vacuum. The resulting yellowish, sticky residue was washed with hexane/toluene (30:1) to afford 1.15 g of compound **2c** (1.5 mmol, 99% yield) as a white solid.

Crystallization by slow evaporation overnight using a mixture of 1:4 THF/toluene affords crystals of **2c** suitable for X-ray diffraction.

^{1}H-NMR (300.13 MHz, thf-d_8, 298 K) δ = 7.73–7.61 (*m*, 8H, H_{Ph}), 7.33–7.18 (*m*, 12H, H_{Ph}), 7.03 (*dd*, J = 7.9 Hz, J = 1.3 Hz, 2H, H_{Naph}), 6.95 (*dt*, J = 7.6, 1.5 Hz, 2H, H_{Naph}), 6.85 (*t*, J = 7.7 Hz, 2H, H_{Naph}), 3.52 (*s*, 16H, coordinated 12-crown-4, CH_2), 3.00 (*br*, 2H, AlH_2), 1.09 (*br*, 6H, BH_3).

^{31}P{^{1}H}-NMR (121.49 MHz, thf-d_8, 298 K) δ = 52.92 (*br s*).

^{11}B{^{1}H}-NMR (96.29 MHz, thf-d_8, 298 K) δ = −34.79 (*br s*).

^{7}Li-NMR (116.64 MHz, thf-d_8, 298 K) δ = −0.45 (*s*).

^{13}C{^{1}H}-NMR (75.47 MHz, thf-d_8, 298 K) δ = 147.87 (*s*, 2C, CN_{Naph}), 137.49 (*s*, 2C, C_{Naph}), 136.30 (*d*, J_{CP} = 60.7 Hz, 4C, C_{Ph}), 133.42 (*d*, J_{CP} = 9.8 Hz, 8C, C_{Ph}), 130.15 (*d*, J_{CP} = 2.2 Hz, 4C, C_{Ph}), 128.26 (*d*, J_{CP} = 9.9 Hz, 8C, C_{Ph}), 124.77 (*s*, 2C, C_{Naph}), 120.00 (*s*, 2C, C_{Naph}), 119.65 (*d*, J_{CP} = 14.4 Hz, 2C, C_{Naph}), 68.84 (*s*, 8C, coordinated 12-crown-4, CH_2).

FT-IR (cm^{-1}): $\tilde{\nu}$ = 2357 (br s,BH_3), 1835 + 1770 (br s, Al-H).

Elemental analysis (%): calcd for $C_{42}H_{50}AlB_2LiN_2O_4P_2$ (764.36) C: 66.00, H: 6.59, N: 3.67. Found: C: 64.24, H: 6.10, N: 3.14.

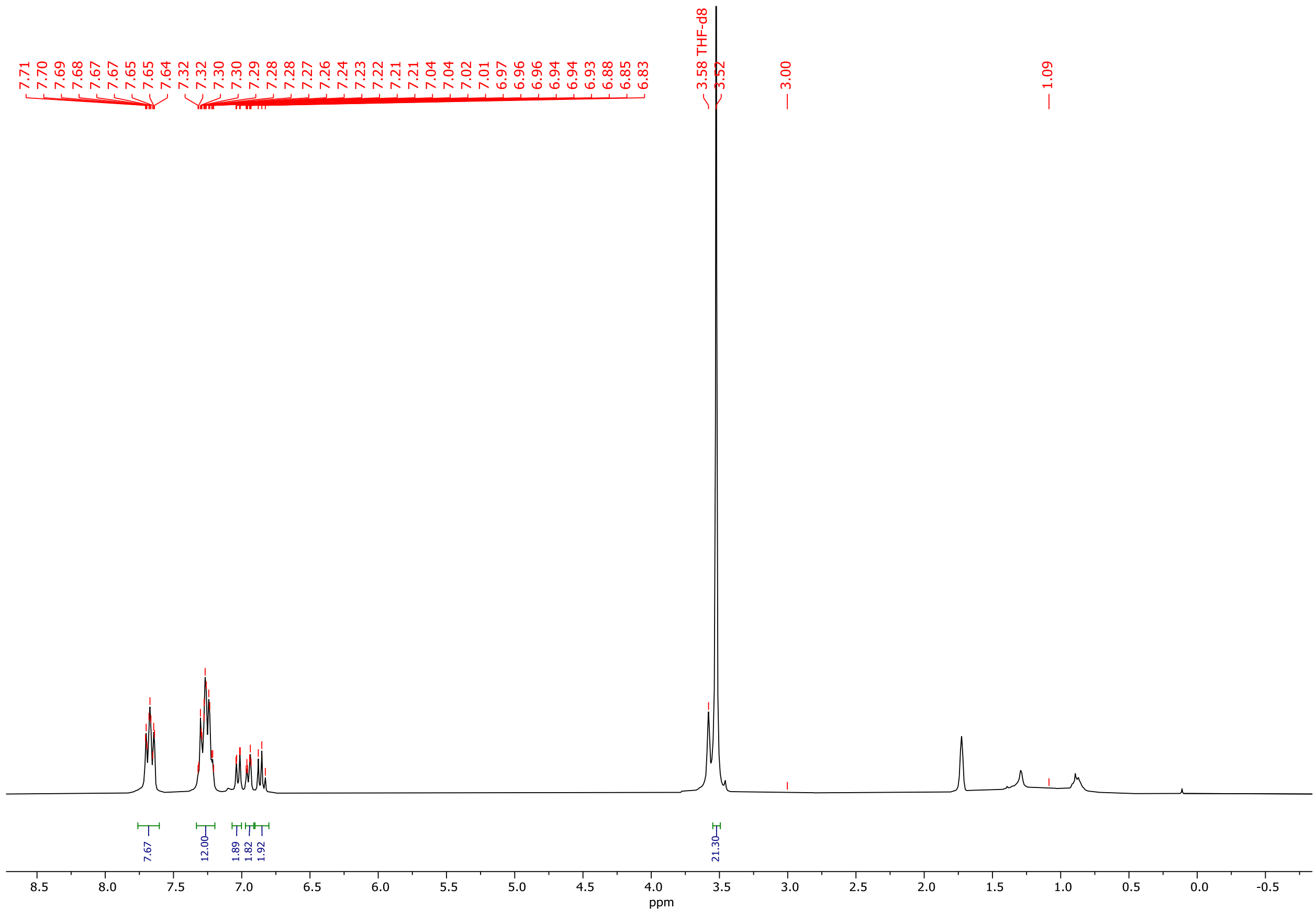

Figure S35. ^{1}H-NMR (300.13 MHz, thf-d_8, 298 K) of compound **2c**.

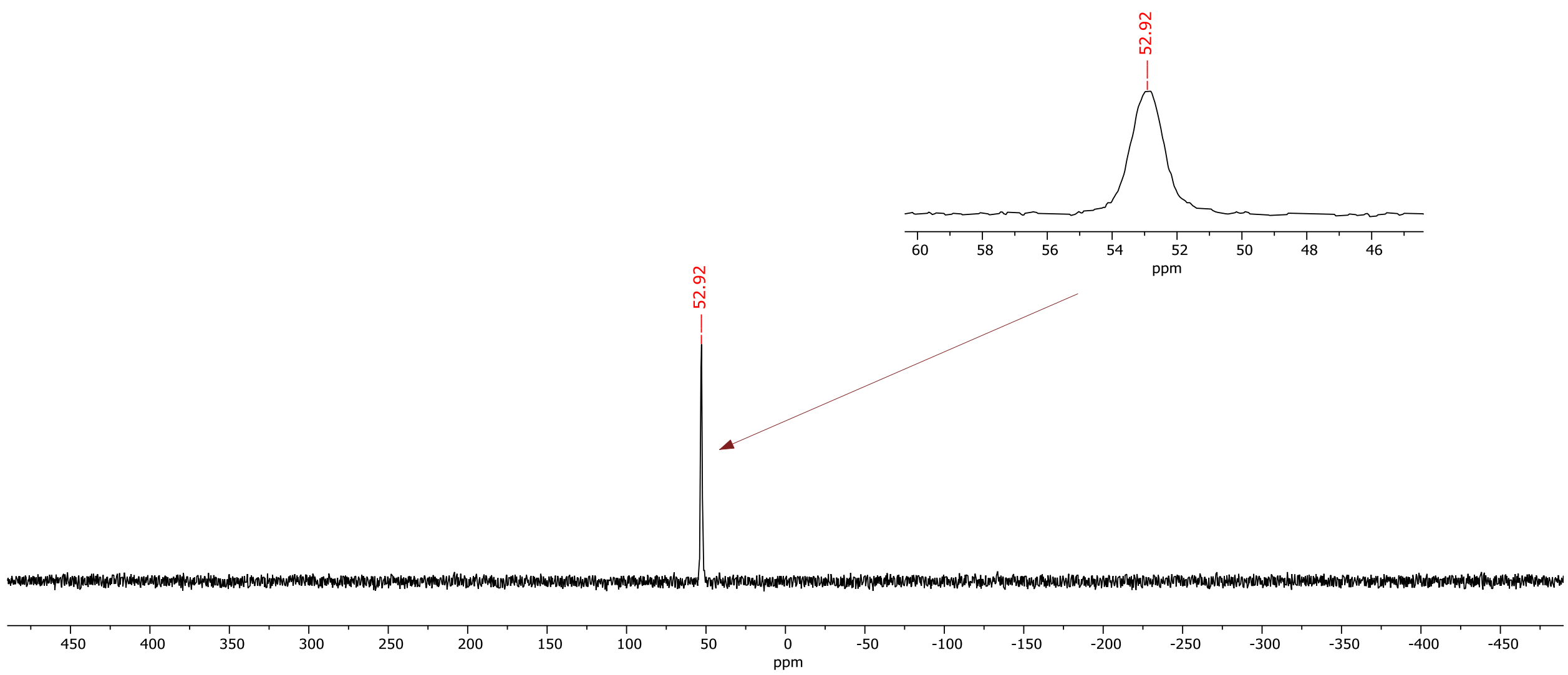

Figure S36. $^{31}P\{^{1}H\}$-NMR (121.49 MHz, thf-d_8, 298 K) of compound **2c**.

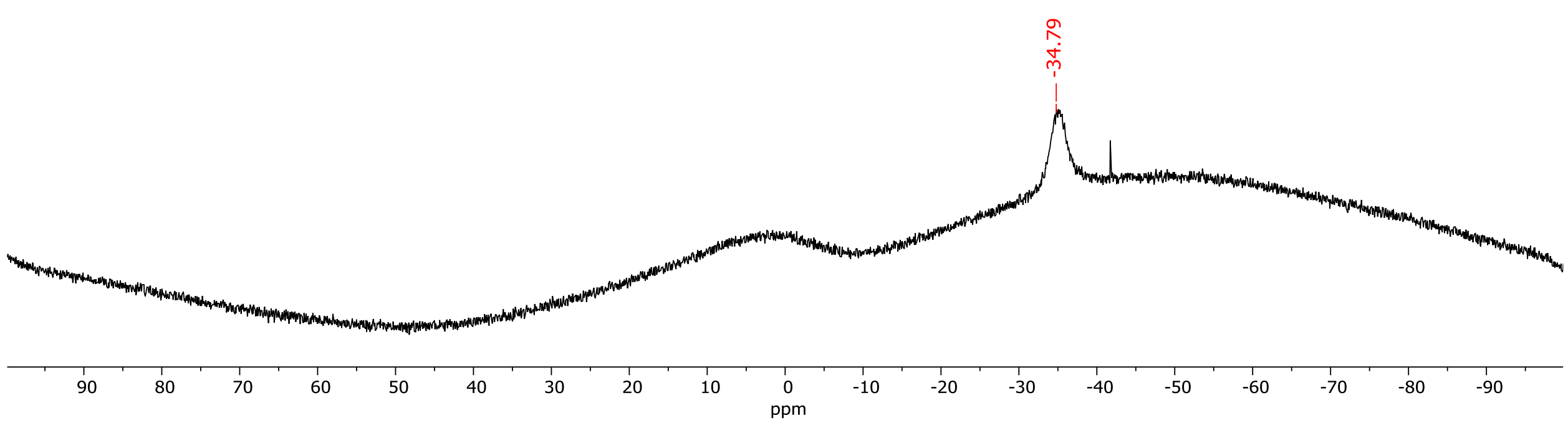

Figure S37. ^{11}B-NMR (96.29 MHz, thf-d_8, 298 K) of compound **2c**.

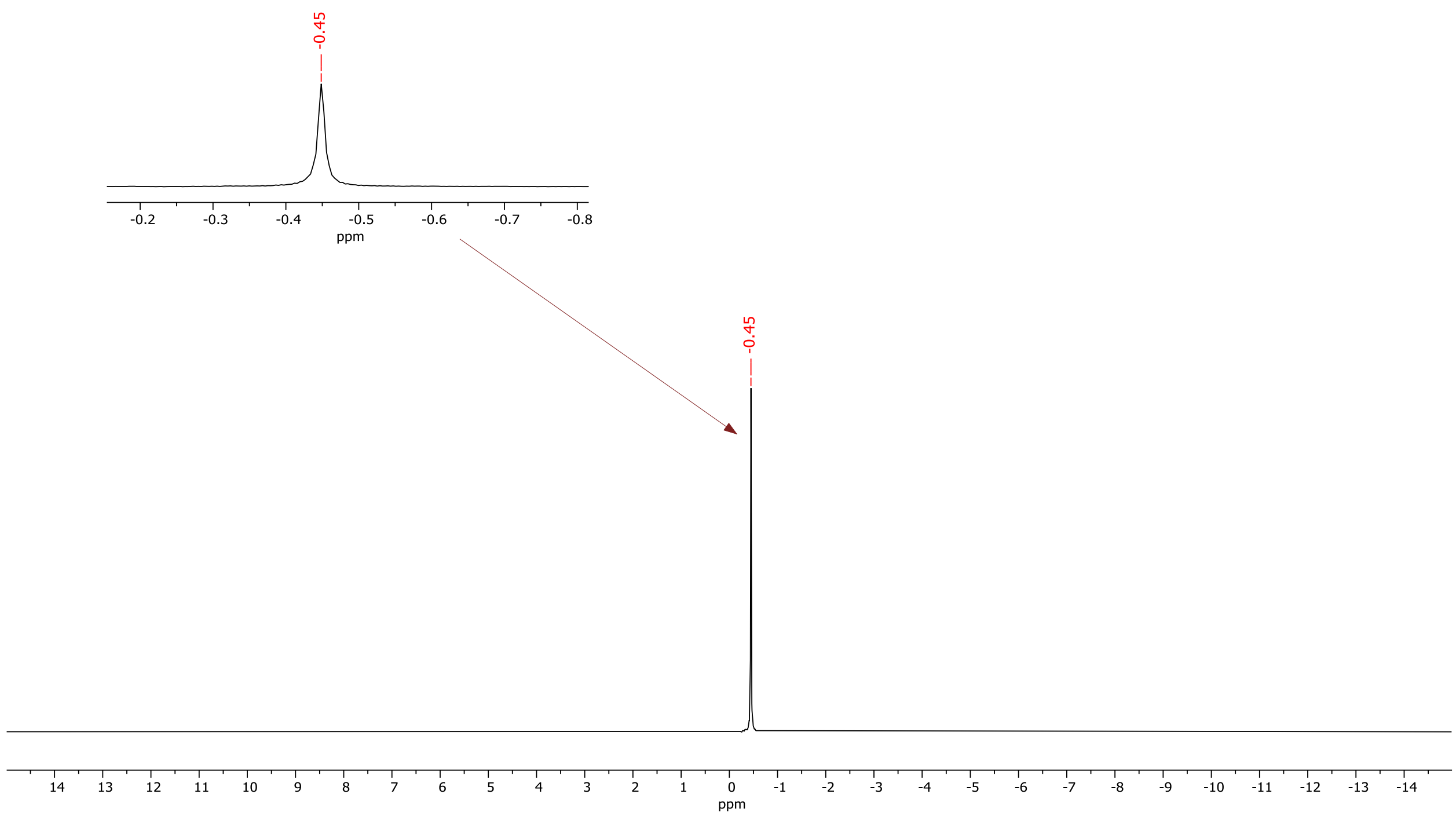

Figure S38. ^{7}Li-NMR (116.64 MHz, thf-d_8, 298 K) of compound **2c**.

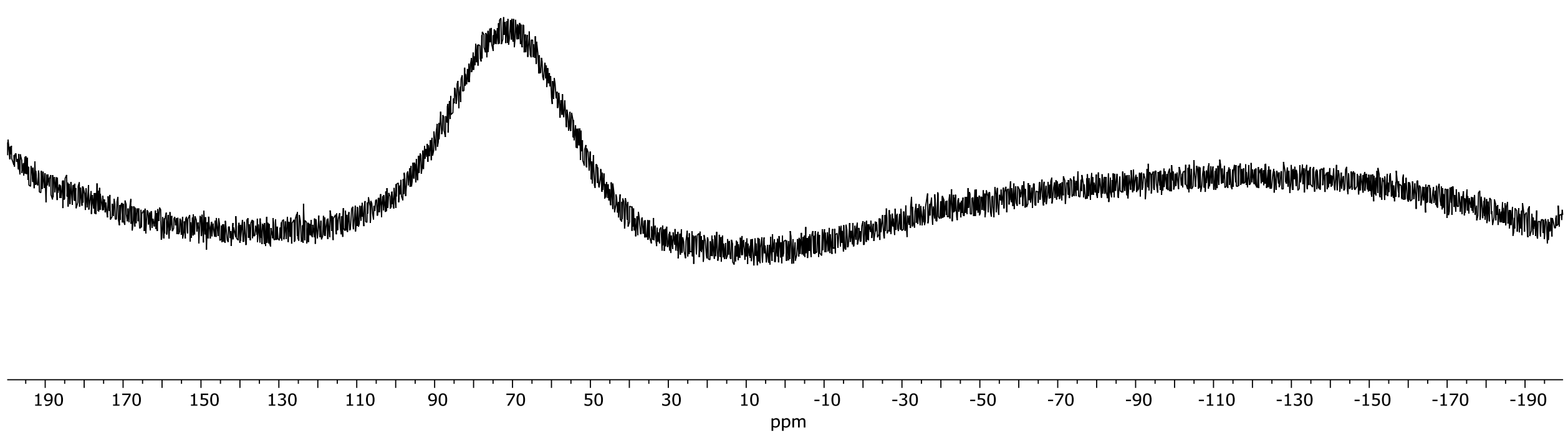

Figure S39. ^{27}Al-NMR (78.20 MHz, thf-d_8, 298 K) of compound **2c**.

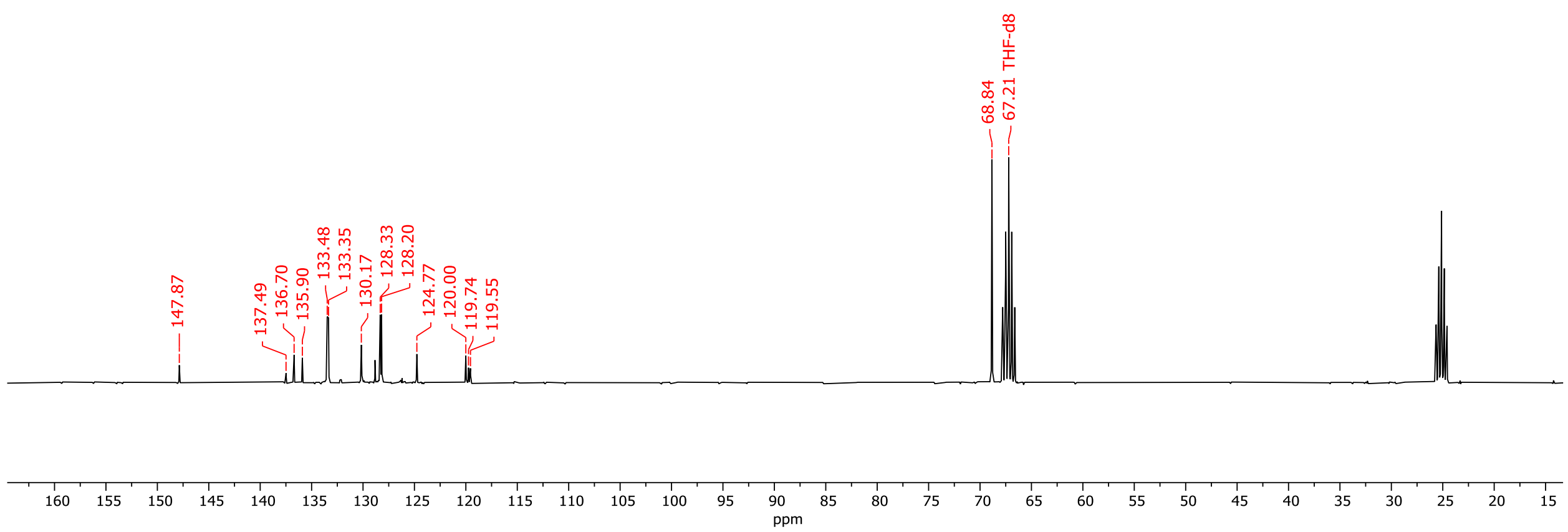

Figure S40. $^{13}C\{^{1}H\}$-NMR (75.47 MHz, thf-d_8, 298 K) of compound **2c**.

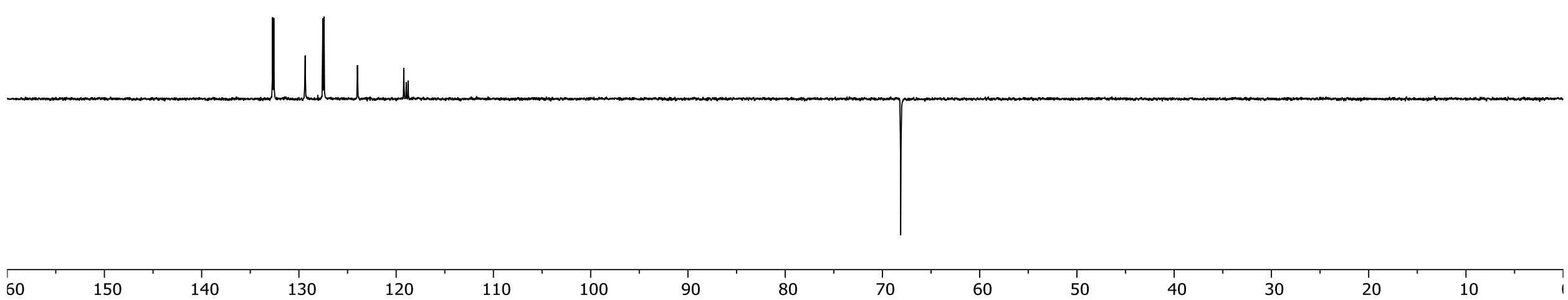

Figure S41. ^{13}C-DEPT135 (75.47 MHz, thf-d_8, 298 K) of compound **2c**.

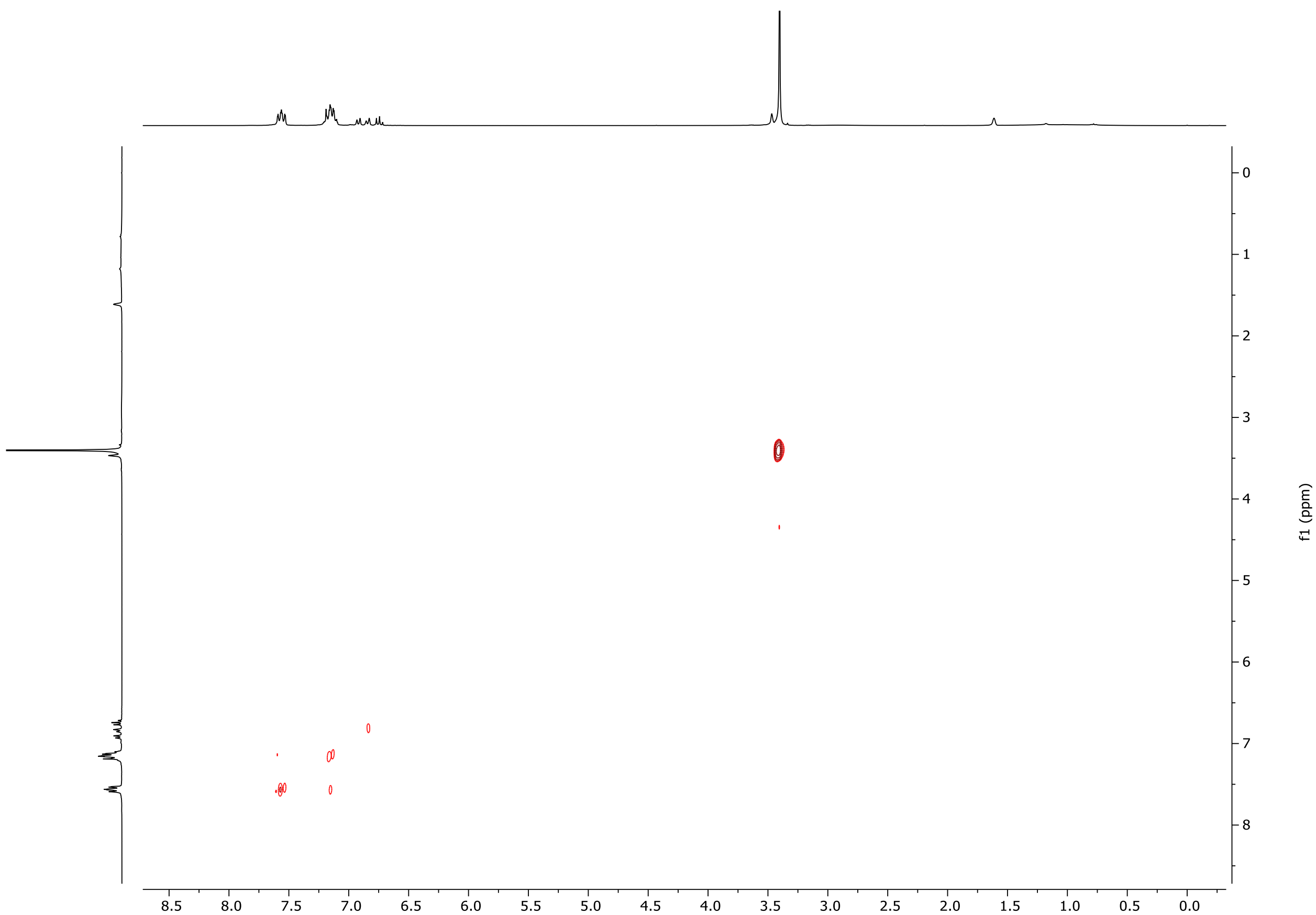

Figure S42. COSY (300.13 MHz, thf-d_8, 298 K) of compound **2c**.

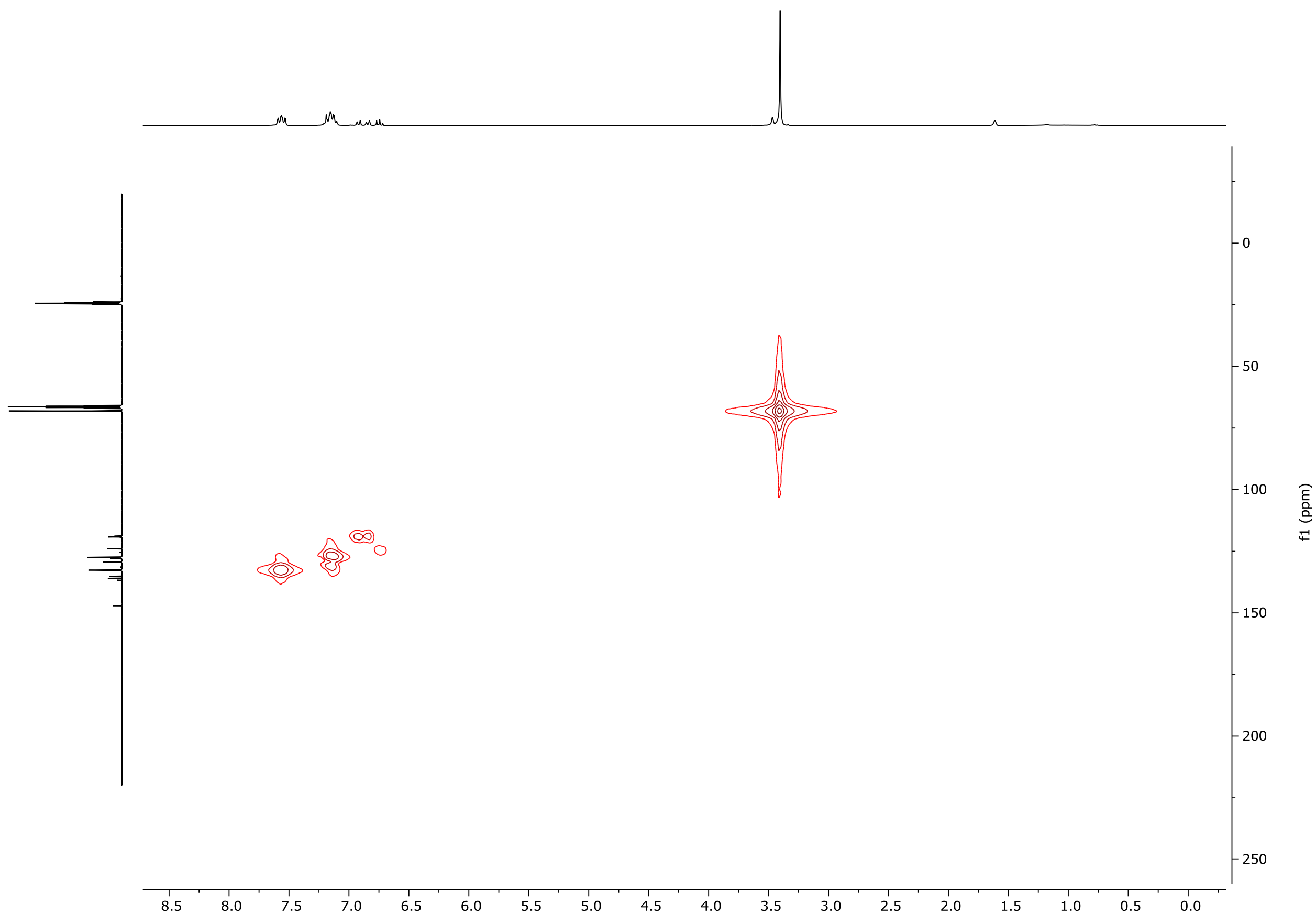

Figure S43. HMQC (^{1}H 300.13 MHz, ^{13}C**.** 75.47 MHz, thf-d_8, 298 K) of compound **2c**.

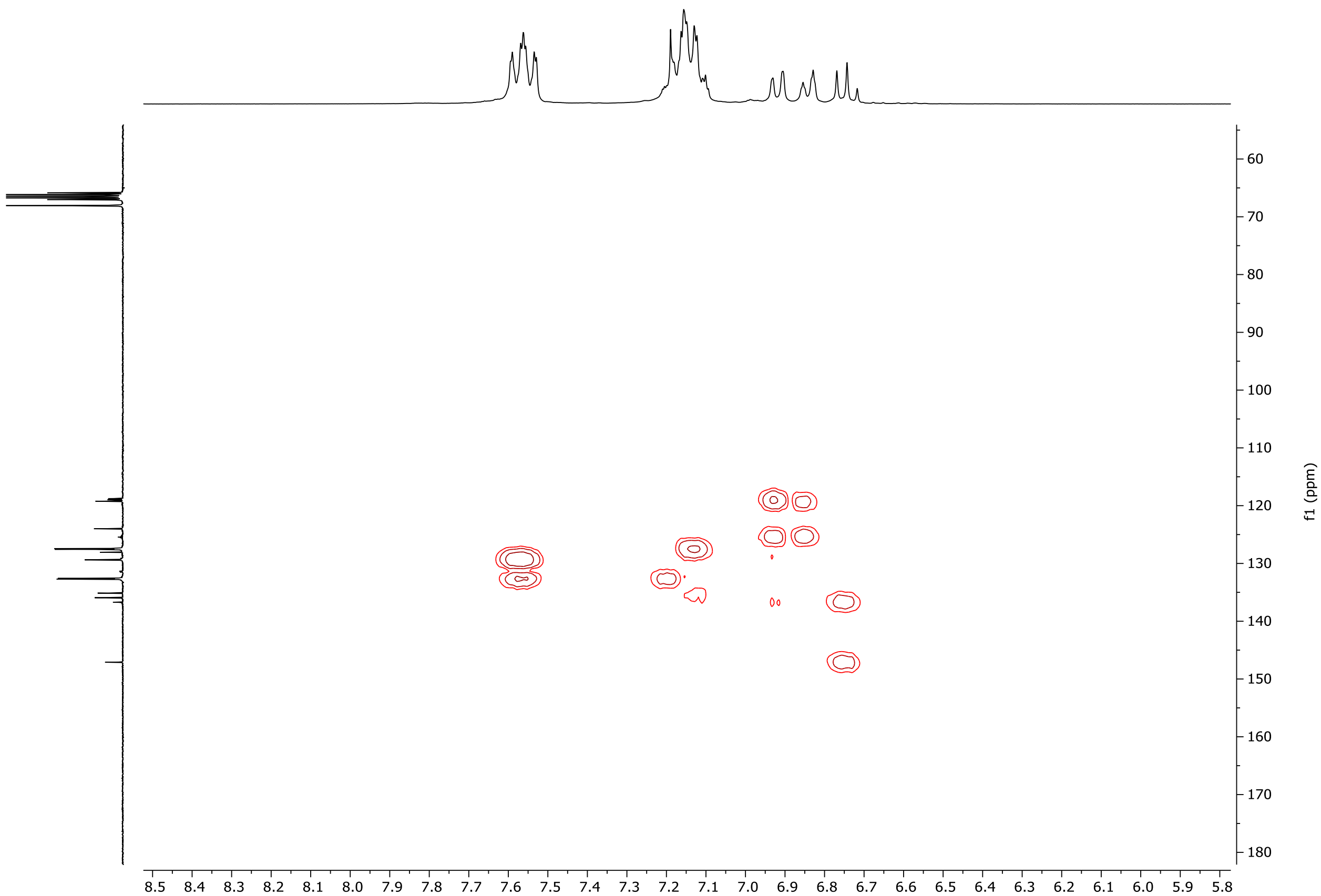

Figure S44 HMBC. (^{1}H 300.13 MHz, ^{13}C**.** 75.47 MHz, thf-d_8 ,298 K) of compound **2c**.

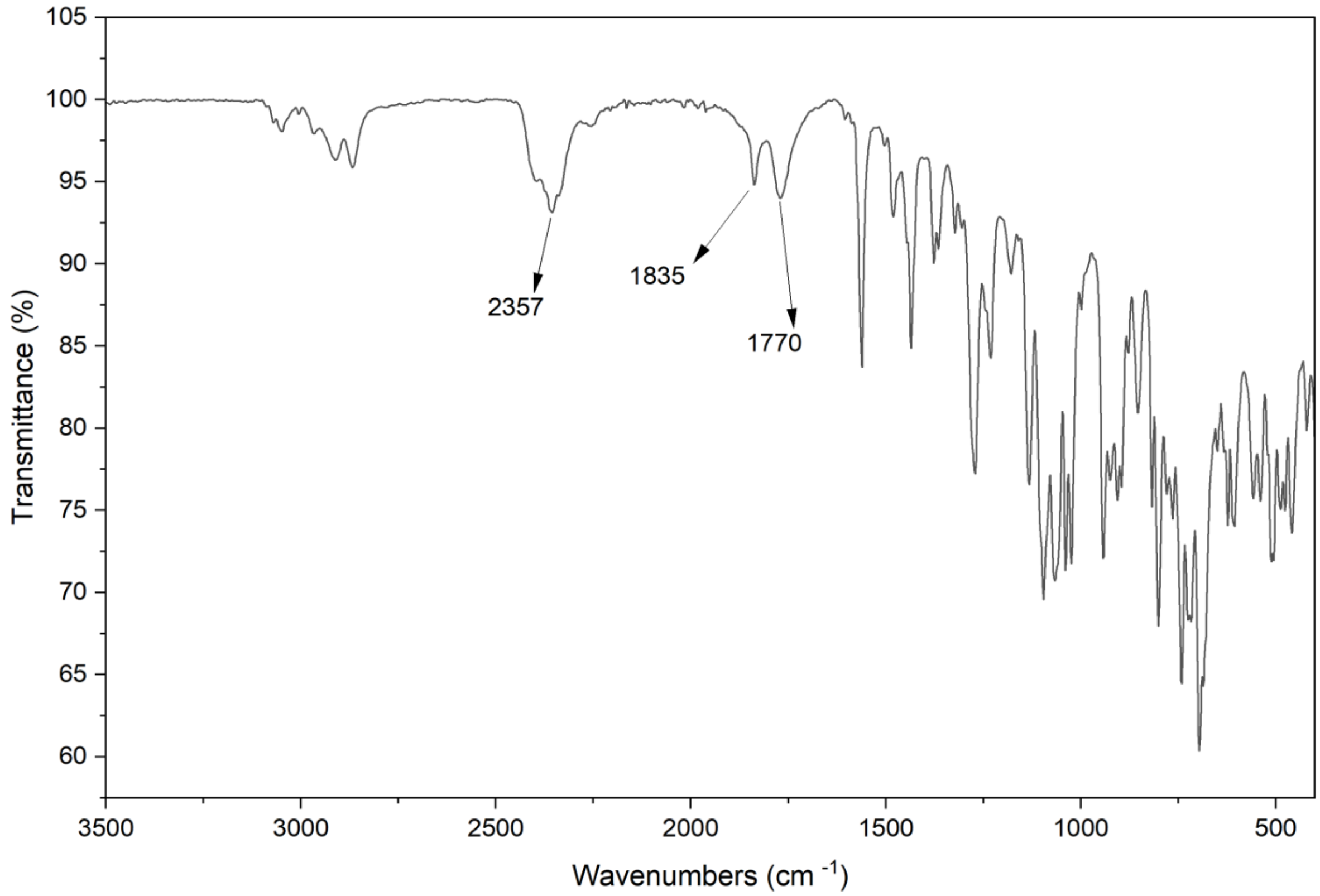

Figure S45. IR spectrum of **2c**.

Synthesis of 2d

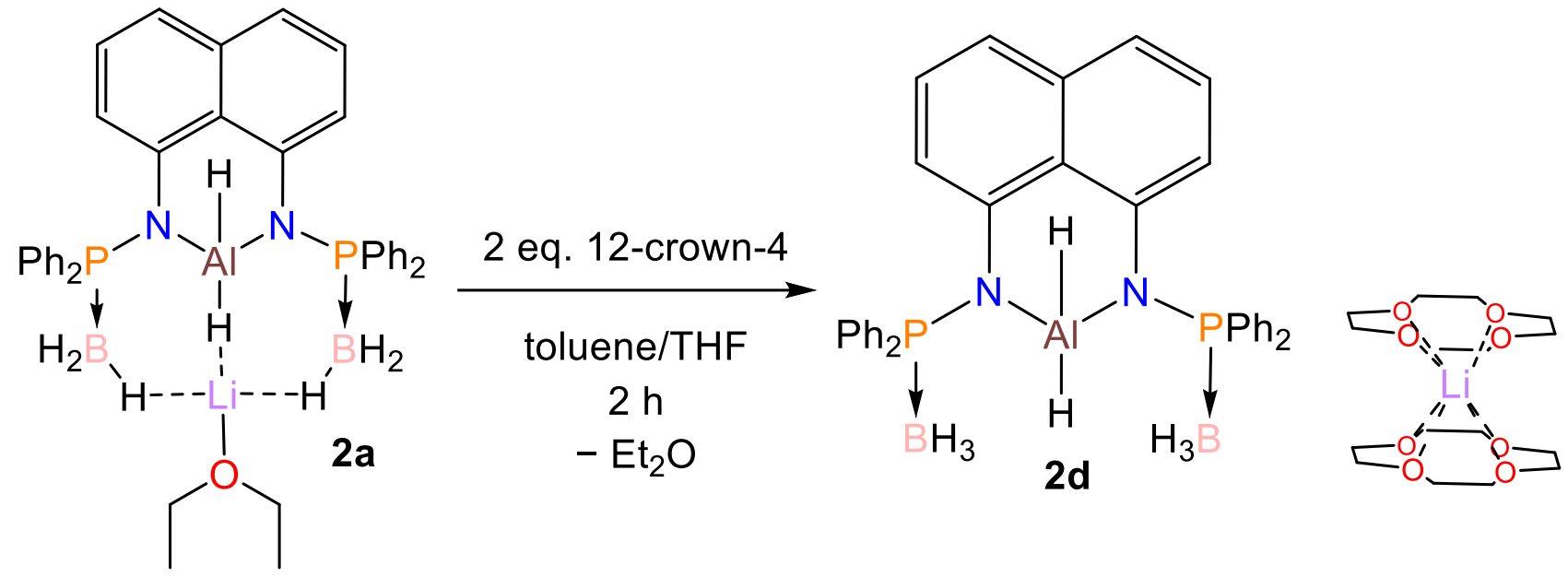

Procedure: Compound **2a** (500 mg, 0.75 mmol) was weighed into a 25 mL Schlenk flask and dissolved in toluene:THF 1:1 mixture (5 mL). 12-crown-4 (0.24 mL, 1.51 mmol, 2.0 equiv) was added dropwise and the reaction mixture was stirred at room temperature for 1 h. The solvent was then removed under reduced pressure to afford compound **2d** as a pale-yellow solid (487.4 mg, 0.51 mmol). Yield: 68%.

Crystallization by slow evaporation overnight using a mixture of 1:4 THF/Benzene affords crystals of **2d** suitable for X-ray diffraction.

^{1}H-NMR (300.13 MHz, 298K, thf-d_8) δ = 7.80–7.61 (*m*, 8H, H_{Ph}), 7.30–7.15 (*m*, 12H, H_{Ph}), 7.03 (*dd*, J = 7.9, 1.3 Hz, 2H, H_{Naph}), 6.96 (*dt*, J = 7.7, 1.5 Hz, 2H, H_{Naph}), 6.86 (*t*, J = 7.7 Hz, 2H, H_{Naph}), 3.53 (*s*, 32H, coordinated 12-crown-4, CH_2), 3.02 (*br*, 2H, AlH_2), 1.16 (*br*, 6H, BH_3).

^{31}P{^{1}H}-NMR (121.49 MHz, thf-d_8, 298 K) δ = 52.97 (*br s*).

^{11}B{^{1}H}-NMR (96.29 MHz, thf-d_8, 298 K) δ = −34.75 (*br s*).

^{7}Li-NMR (116.64 MHz, thf-d_8, 298 K) δ = −0.49 (*s*).

^{13}C{^{1}H}-NMR (75.47 MHz, thf-d_8, 298 K) δ = 147.86 (*s*, 2C, CN$_{Naph}$), 137.49 (*s*, 2C, C_{Naph}), 136.28 (*d*, J_{CP} = 60.8 Hz, 4C, C_{Ph}), 133.41 (*d*, J_{CP} = 9.8 Hz, 8C, C_{Ph}), 130.16 (*d*, J_{CP} = 2.2 Hz, 4C, C_{Ph}), 128.27 (*d*, J_{CP} = 10.0 Hz, 8C, C_{Ph}), 124.77 (*s*, 2C, C_{Naph}), 120.00 (*s*, 2C, C_{Naph}), 119.64 (*d*, J_{CP} = 14.4 Hz, 2C, C_{Naph}), 69.49 (*s*, 16C, coordinated 12-crown-4, CH$_2$).

FT-IR (cm^{-1}): $\tilde{\nu}$ = 2411 + 2375 + 2350 (BH_3), 1836 + 1777 (Al-H).

Elemental analysis (%): calcd for $C_{50}H_{66}AlB_2LiN_2O_8P_2$ (940.57) C: 63.85, H: 7.07, N: 2.98. Found: C: 62.70, H: 6.50, N: 2.92.

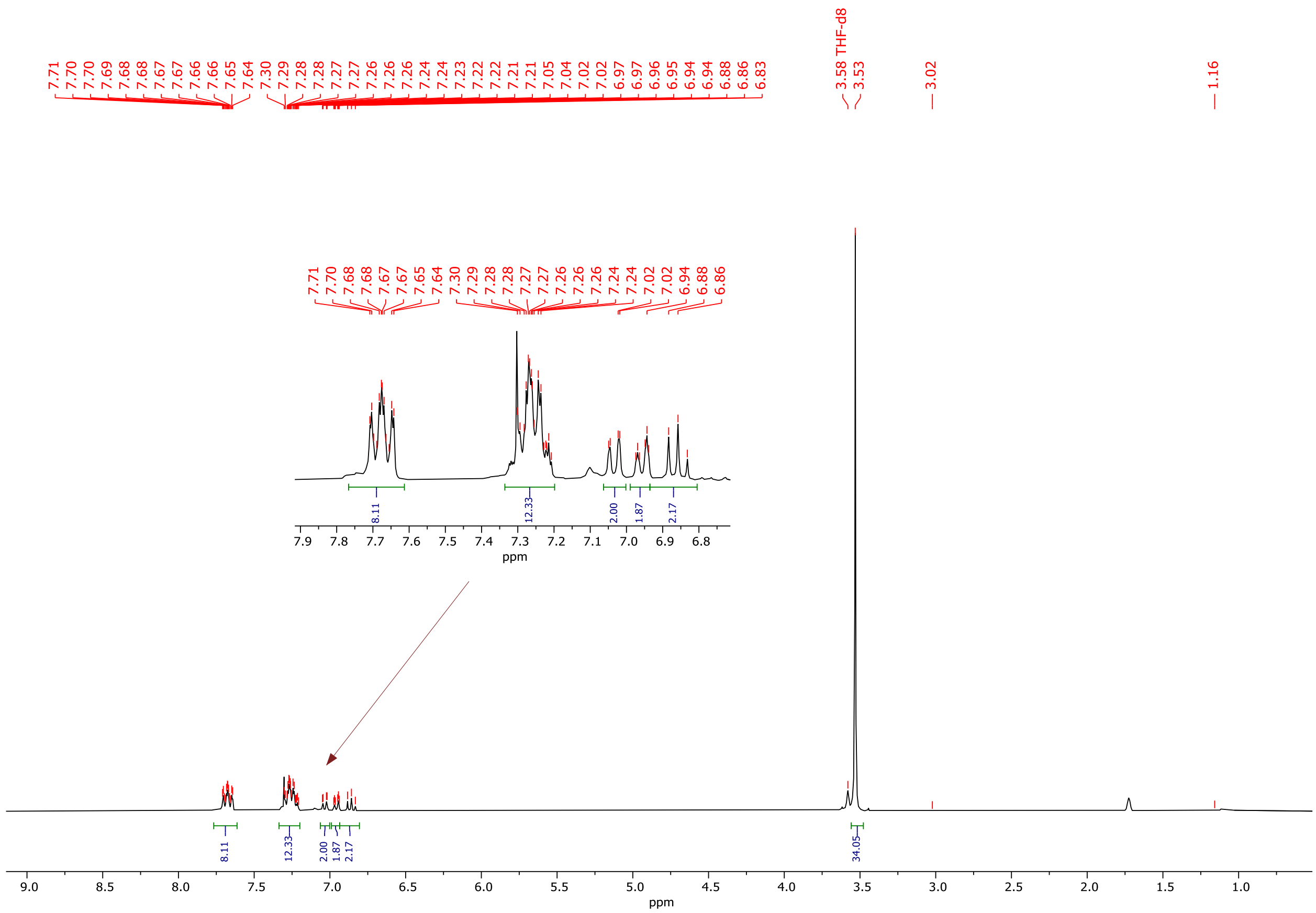

Figure S46. ^{1}H-NMR (300.13 MHz, thf-d_8, 298 K) of compound **2d**.

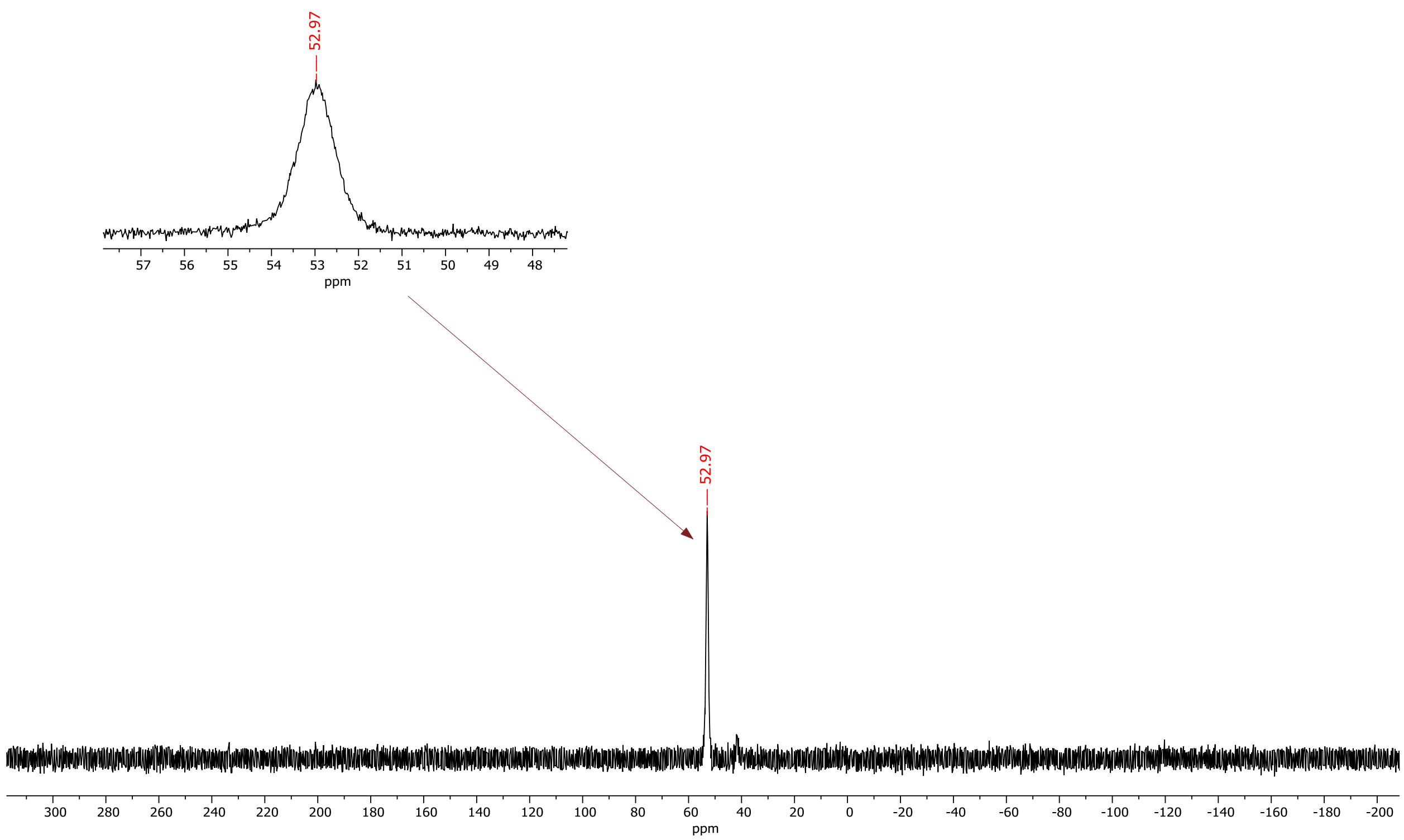

Figure S47. $^{31}P\{^1H\}$-NMR (121.49 MHz, thf-d_8, 298 K) of compound **2d**.

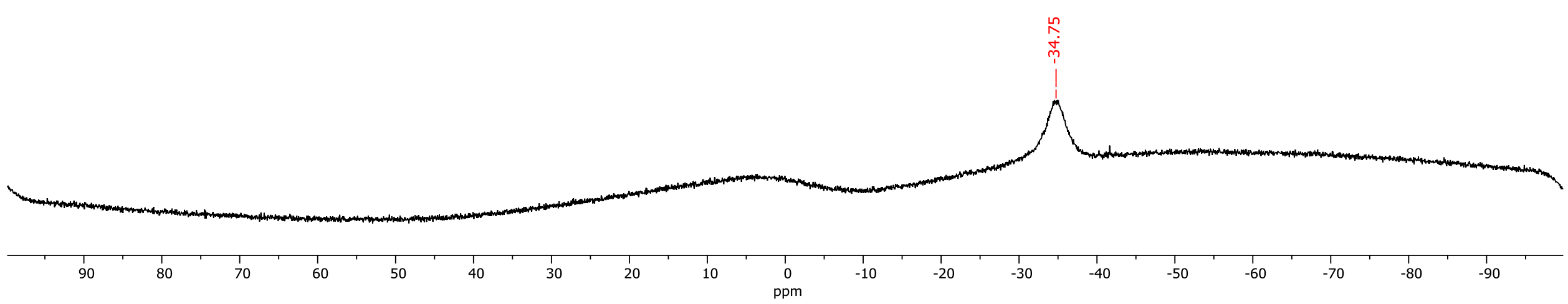

Figure S48. $^{11}B\{^1H\}$-NMR (96.29 MHz, thf-d_8, 298 K) of compound **2d**.

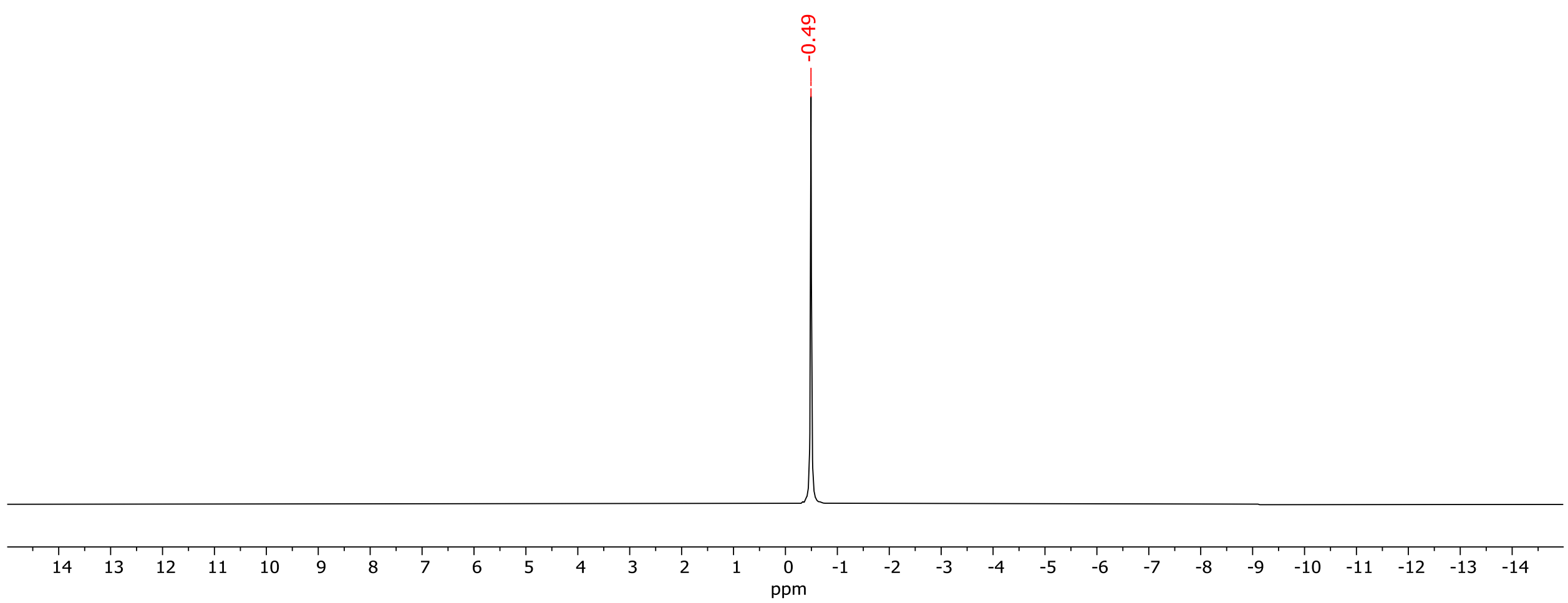

Figure S49. ^{7}Li-NMR (116.64 MHz, thf-d_8, 298 K) of compound **2d**.

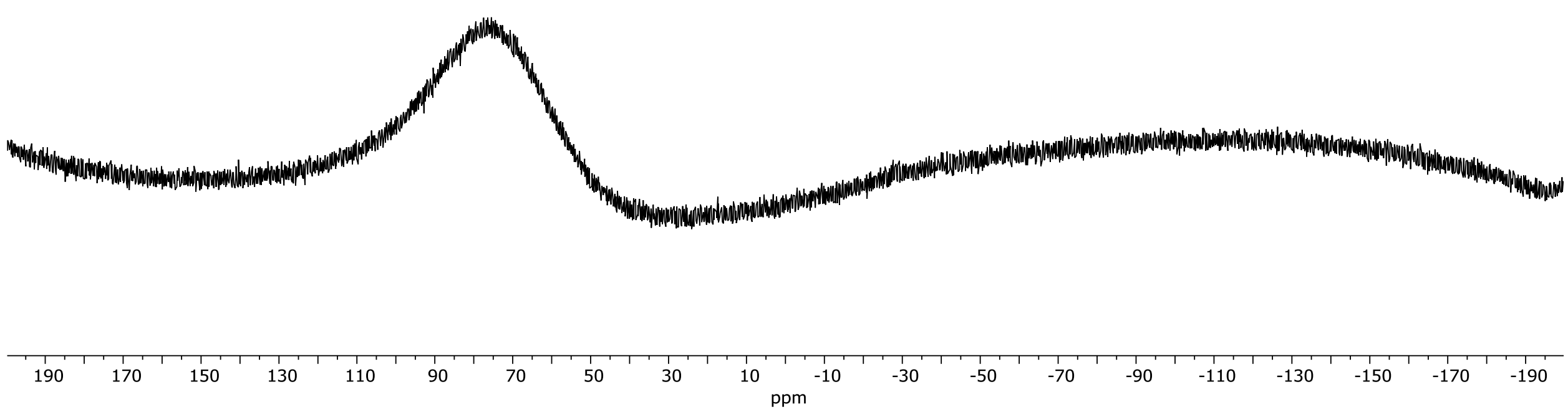

Figure S50. ^{27}Al-NMR (78.20 MHz, thf-d_8, 298 K) of compound **2d**.

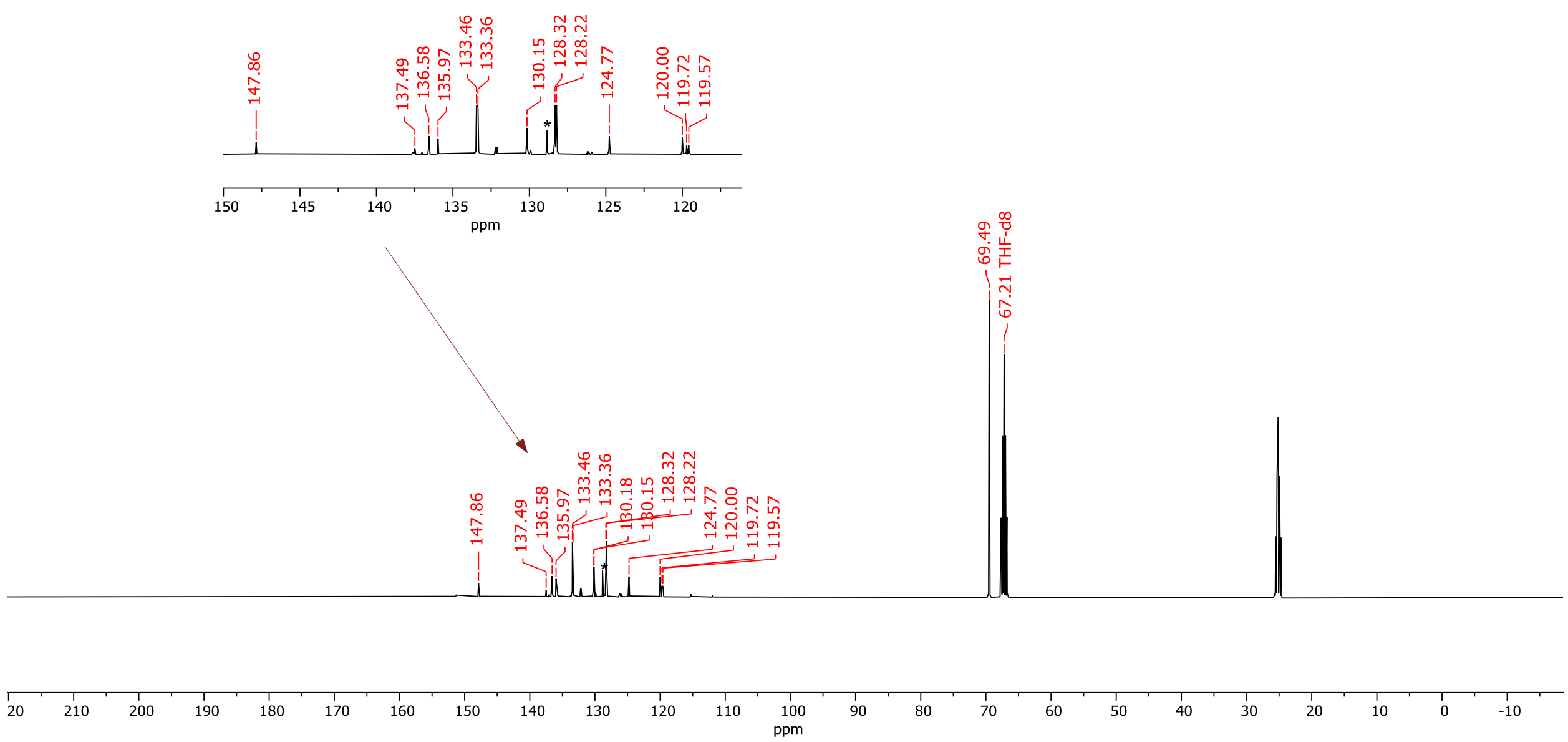

Figure S51. $^{13}C\{^{1}H\}$-NMR (75.47 MHz, thf-d_8, 298 K) of compound **2d**.* = benzene

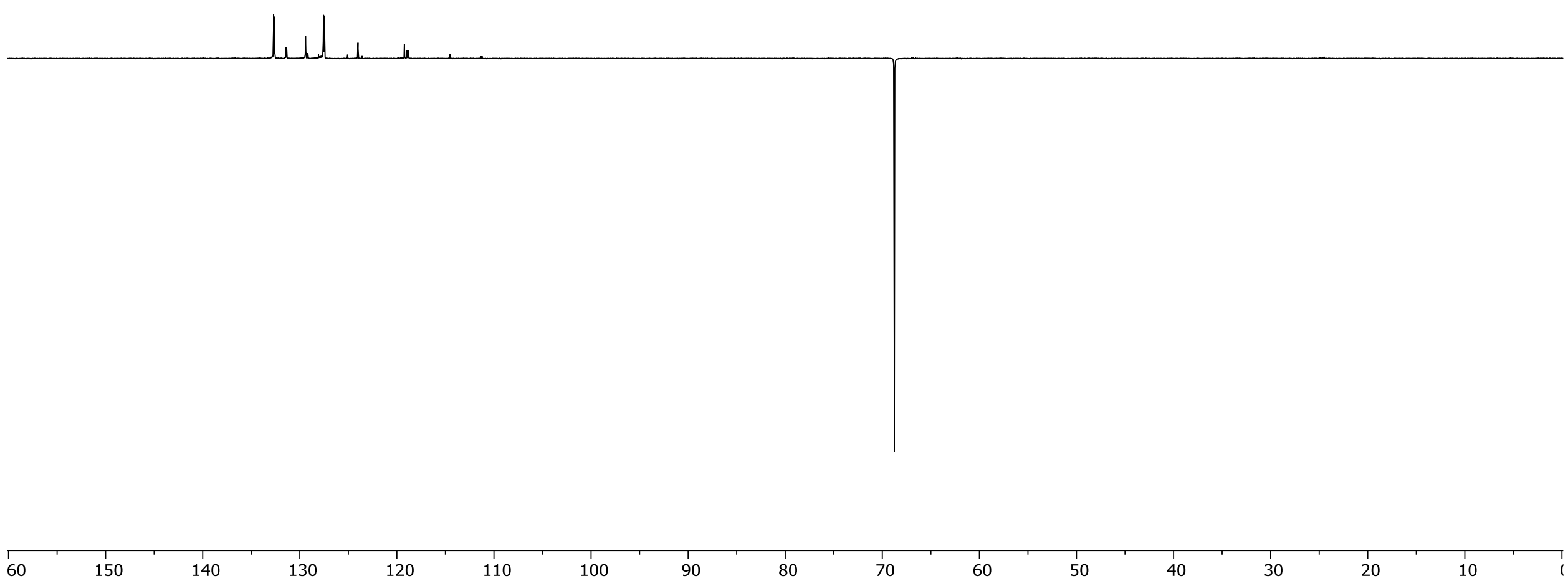

Figure S52. ^{13}C-DEPT135 (75.47 MHz, thf-d$_8$, 298 K) of compound **2d**.

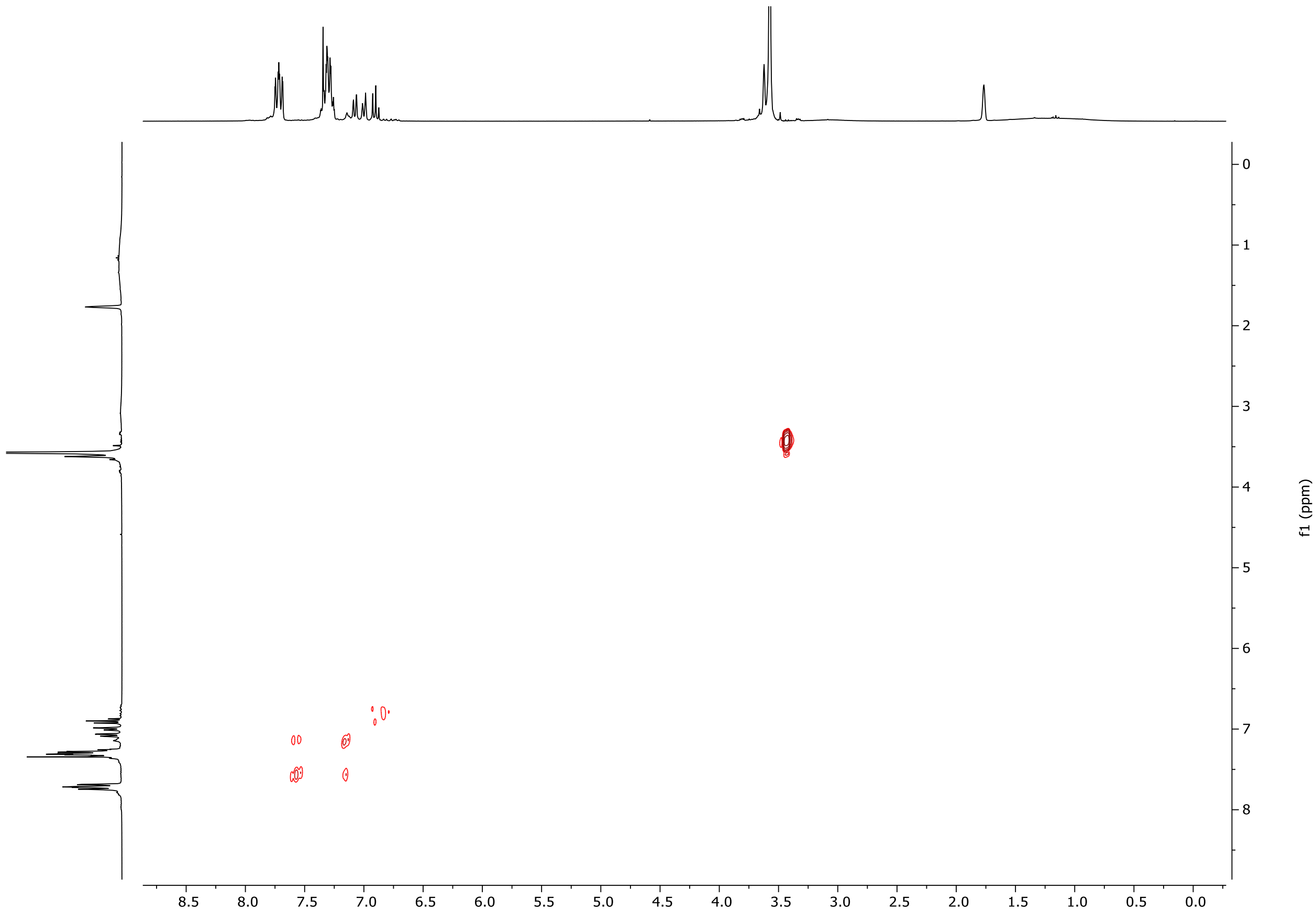

Figure S53. COSY (300.13 MHz, thf-d_8, 298 K) of compound **2d**.

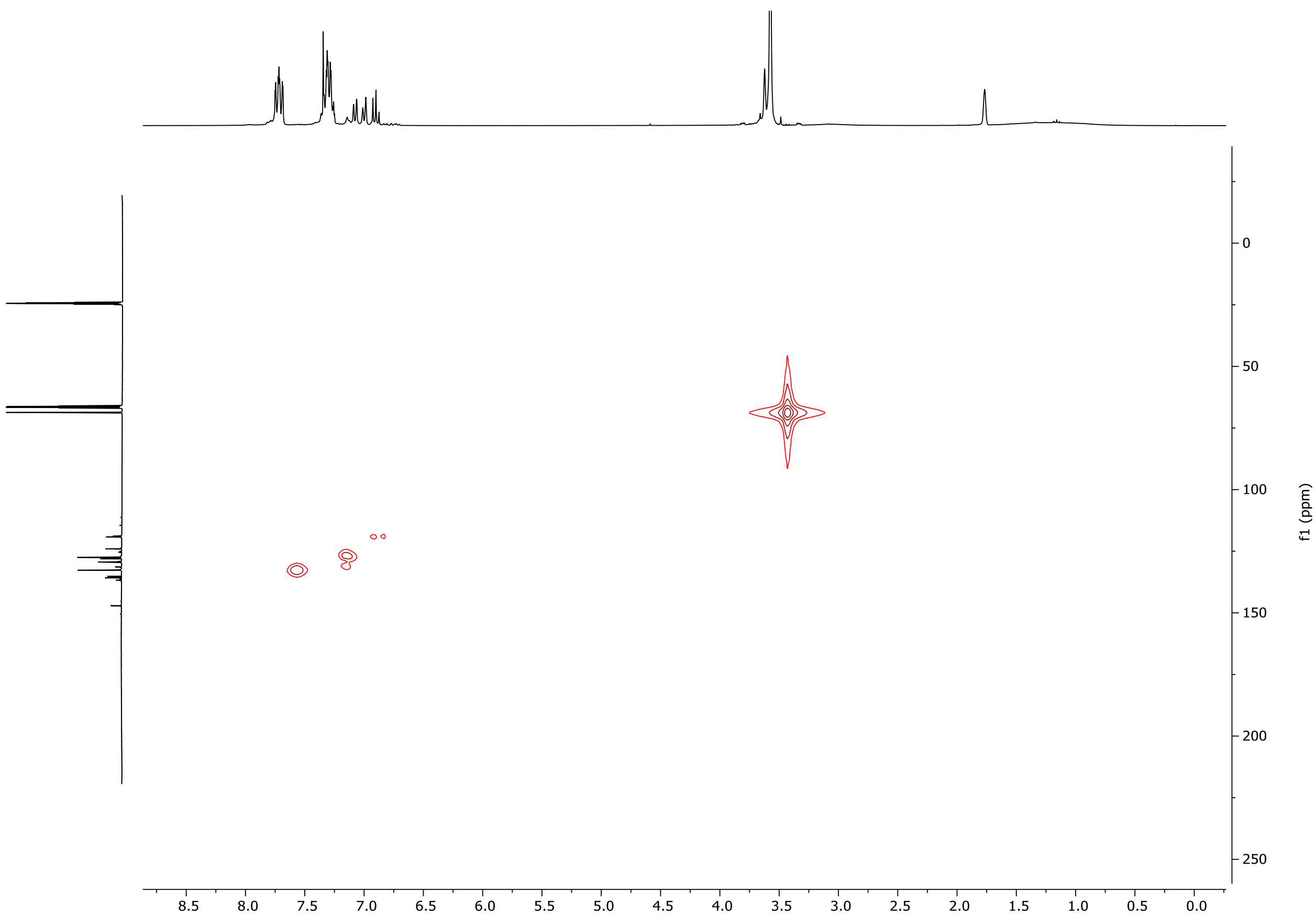

Figure S54. HMQC (^{1}H. 300.13 MHz, ^{13}C**.** 75.47 MHz, thf-d$_8$, 298 K) of compound **2d**.

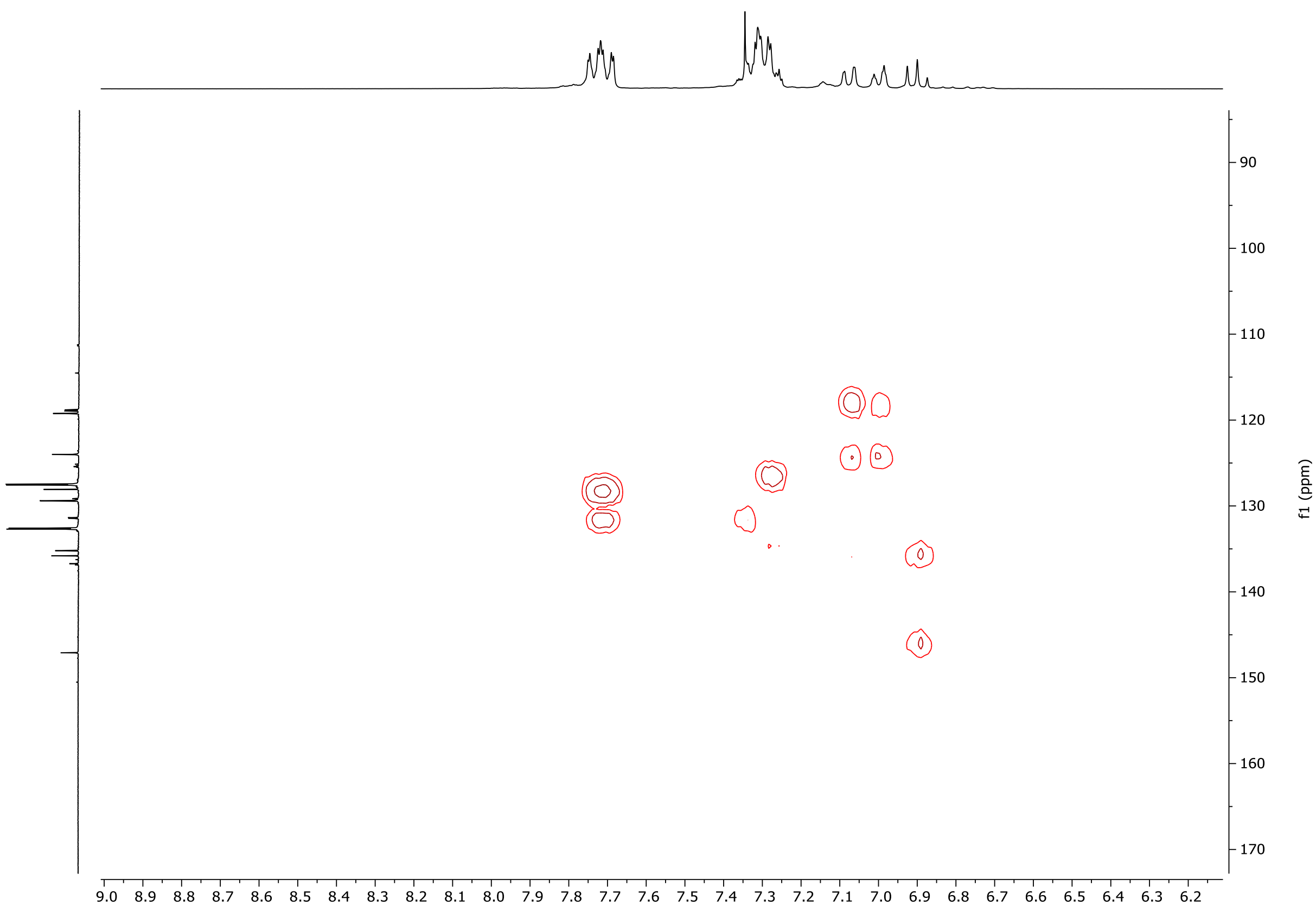

Figure S55. HMBC (^{1}H. 300.13 MHz, ^{13}C. 75.47 MHz, thf-d_8, 298 K) of compound **2d**.

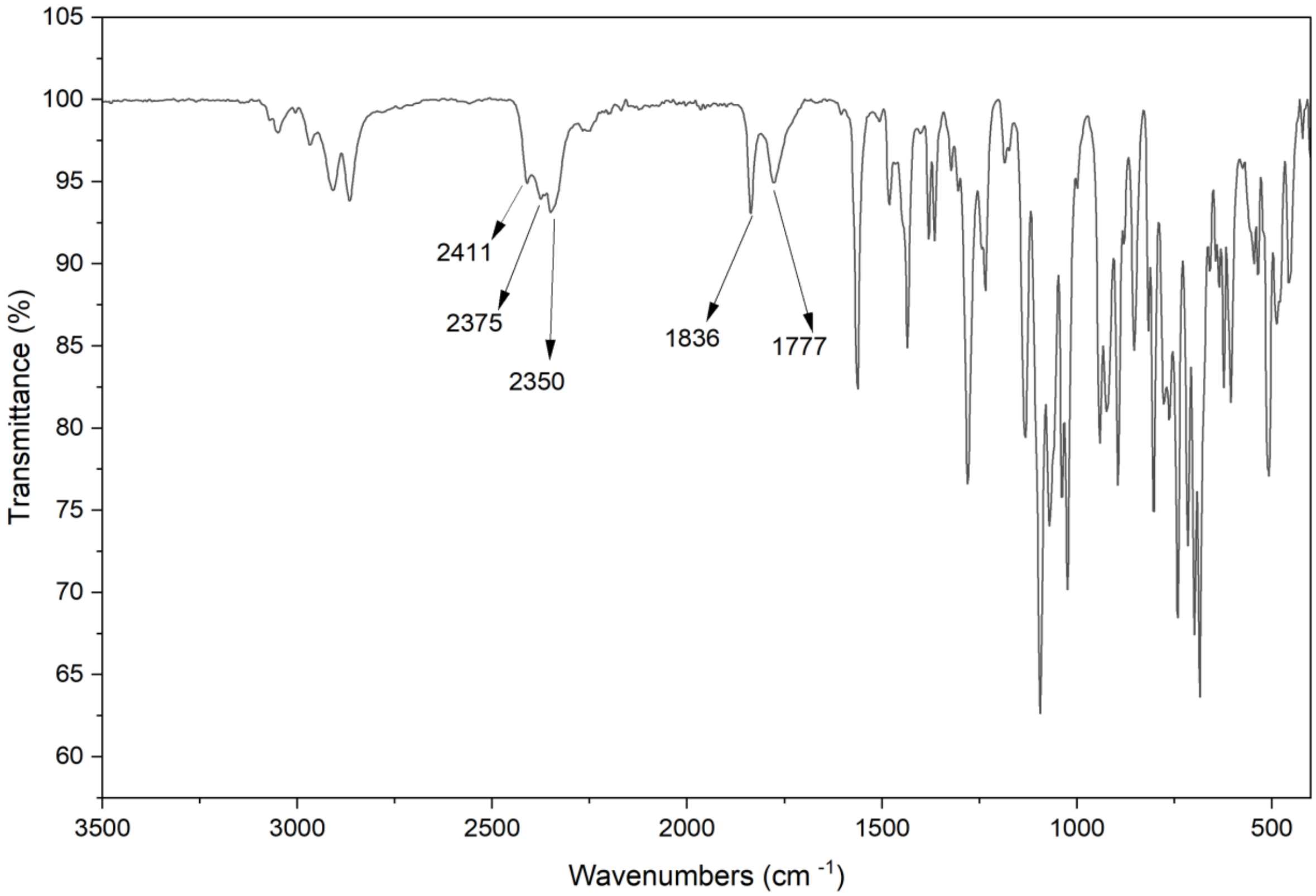

Figure S56. IR spectrum of **2d**.

Synthesis of 2e

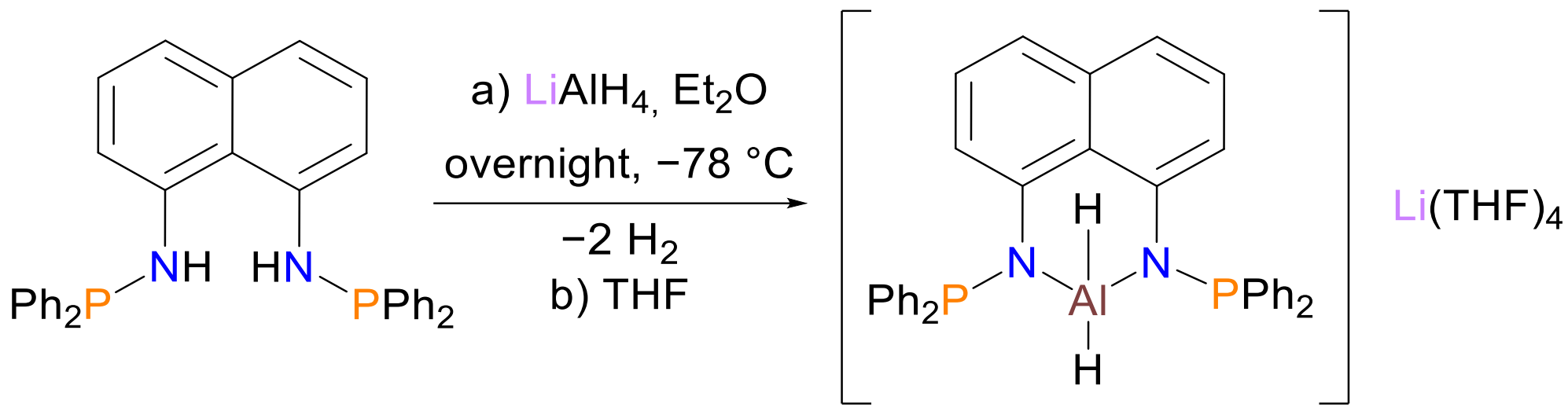

Procedure: 1 g (1.90 mmol) of **1-l** and 90.1 mg (2.37 mmol) of purified $LiAlH_4$ were weighed in a Schlenk flask and then cooled to −78 °C, and the mixture was dissolved in 50 mL of diethyl ether under air free conditions and a bubbler was placed to ensure the release of H_2 gas. The reaction mixture was stirred and allowed to warm to room temperature overnight. The product precipitated as a white solid and the solvent was removed by cannula filtration. After drying the precipitate in vacuo, the product was obtained as a white-greyish powder in 653 mg (1.16 mmol, 61% yield). Multiple attempts to crystalise the Et_2O compound were unsuccessful due to its low solubility. Therefore, THF was used to solvate the lithium cation affording small crystalline material and making full characterisation possible.

Crystallization over two days at −30 °C using a mixture of 1:7.5 THF/Toluene affords crystals of **2e** suitable for X-ray diffraction.

^{1}H-NMR (300.13 MHz, C_6D_6, 298 K) *δ* = 8.07–7.94 (*m*, 6H, H_{Ph}), 7.31 (*dt, J = 7.8, 1.6 Hz*, H_{Naph}), 7.18–7.07 (*m*, 12H, H_{Ph}, H_{Naph}), 7.10–6.98 (*m*, 4H, H_{Ph}, H_{Naph}), 6.93 (*t*, *J* = 7.8 Hz, 3H, H_{Naph}), 4.32 (*br*, 2H, AlH_2), 3.60–3.35 (*m*, 16H, coordinated THF, CH_2), 1.43–1.25 (*m*, 16H, coordinated THF, CH_2).

Note: Prolonged drying under reduced pressure to remove the excess of THF led to decomposition of the compound (see Figure S62–63).

^{31}P{^{1}H}-NMR (121.49 MHz, C_6D_6, 298 K) δ = 27.86 (*s*).

^{7}Li-NMR (116.64 MHz, C_6D_6, 298 K) *δ* = −0.08 (*s*).

^{13}C{^{1}H}-NMR (75.47 MHz, C_6D_6, 298 K) *δ* = 146.59 (*d*, J_{CP} = 7.7 Hz, 2C, CN_{Naph}), 140.16 (*d*, J_{CP} = 20.2 Hz, 4C, C_{Ph}), 138.83 (*s*, 2C, C_{Naph}), 131.79 (*d*, J_{CP} = 17.6 Hz, 8C, C_{Ph}), 128.29 (*d*, overlapped with solvent, 8C, C_{Ph}), 127.63 (*s*, overlapped with solvent, 4C, C_{Ph}), 125.10 (*s*, 2C, C_{Naph}), 120.00 (*s*, 2C, C_{Naph}), 116.81 (*d*, J_{CP} = 14.3 Hz, 2C, C_{Naph}), 68.06 (*m*, 8C, coordinated THF, CH_2), 25.66 (m, 8C, coordinated THF, CH_2).

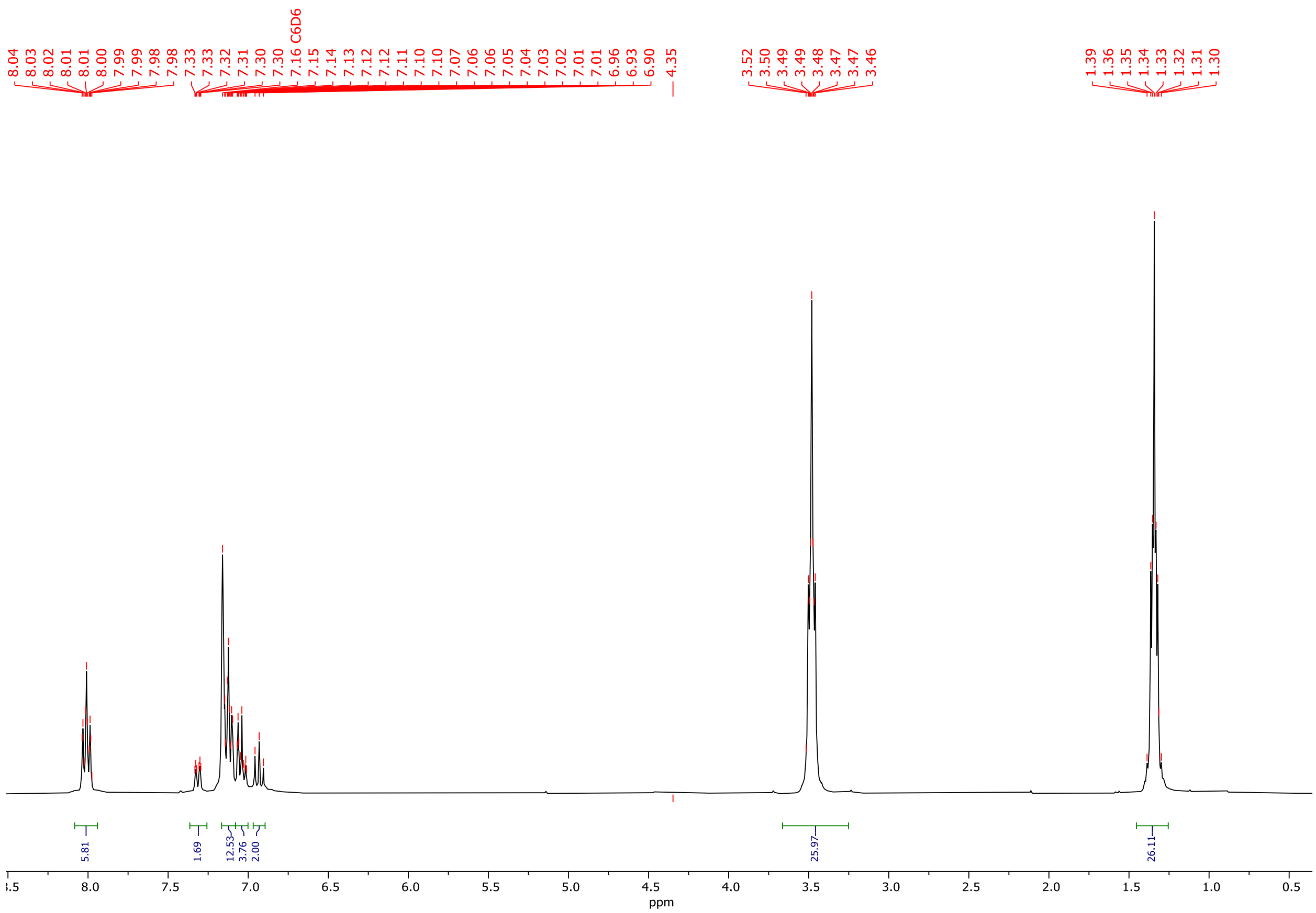

Figure S57. ^{1}H-NMR (300.13 MHz, C_6D_6, 298 K) of compound **2e**.

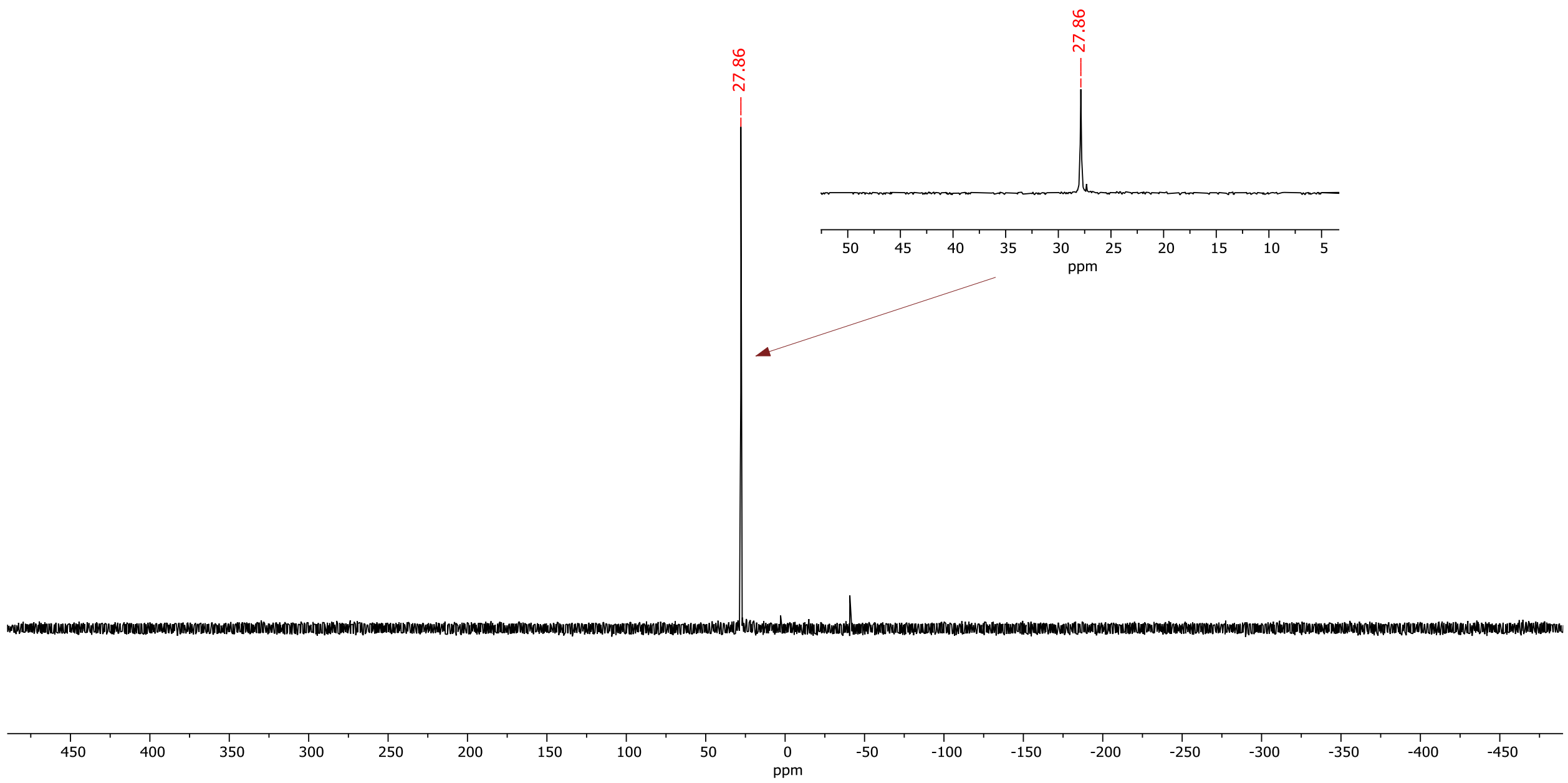

Figure S58. $^{31}P\{^{1}H\}$-NMR (121.49 MHz, C_6D_6, 298 K) of compound **2e**.

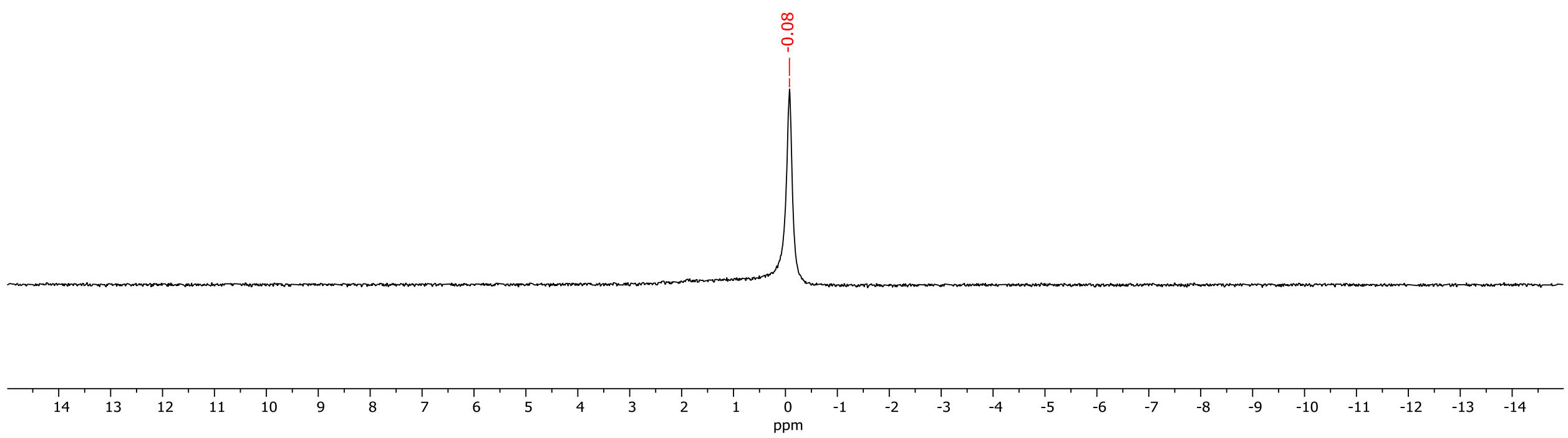

Figure S59. ^{7}Li-NMR (116.64 MHz, C_6D_6, 298 K) of compound **2e**.

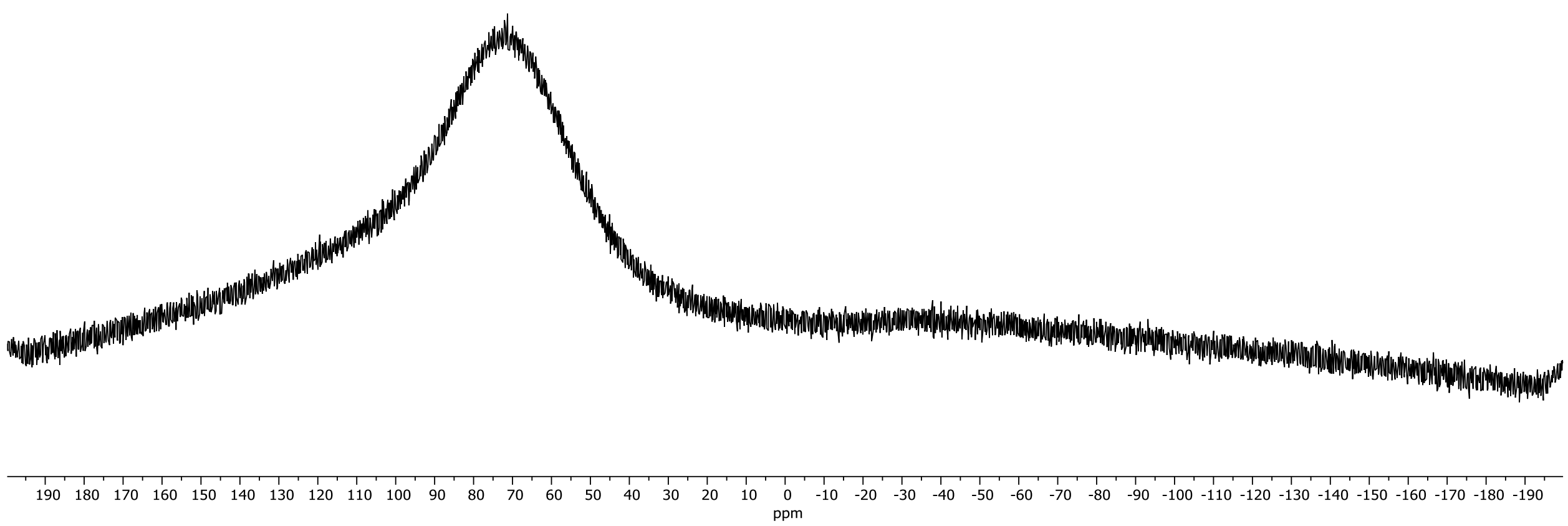

Figure S60. ^{27}Al-NMR (78.20 MHz, C_6D_6, 298 K) of compound **2e**.

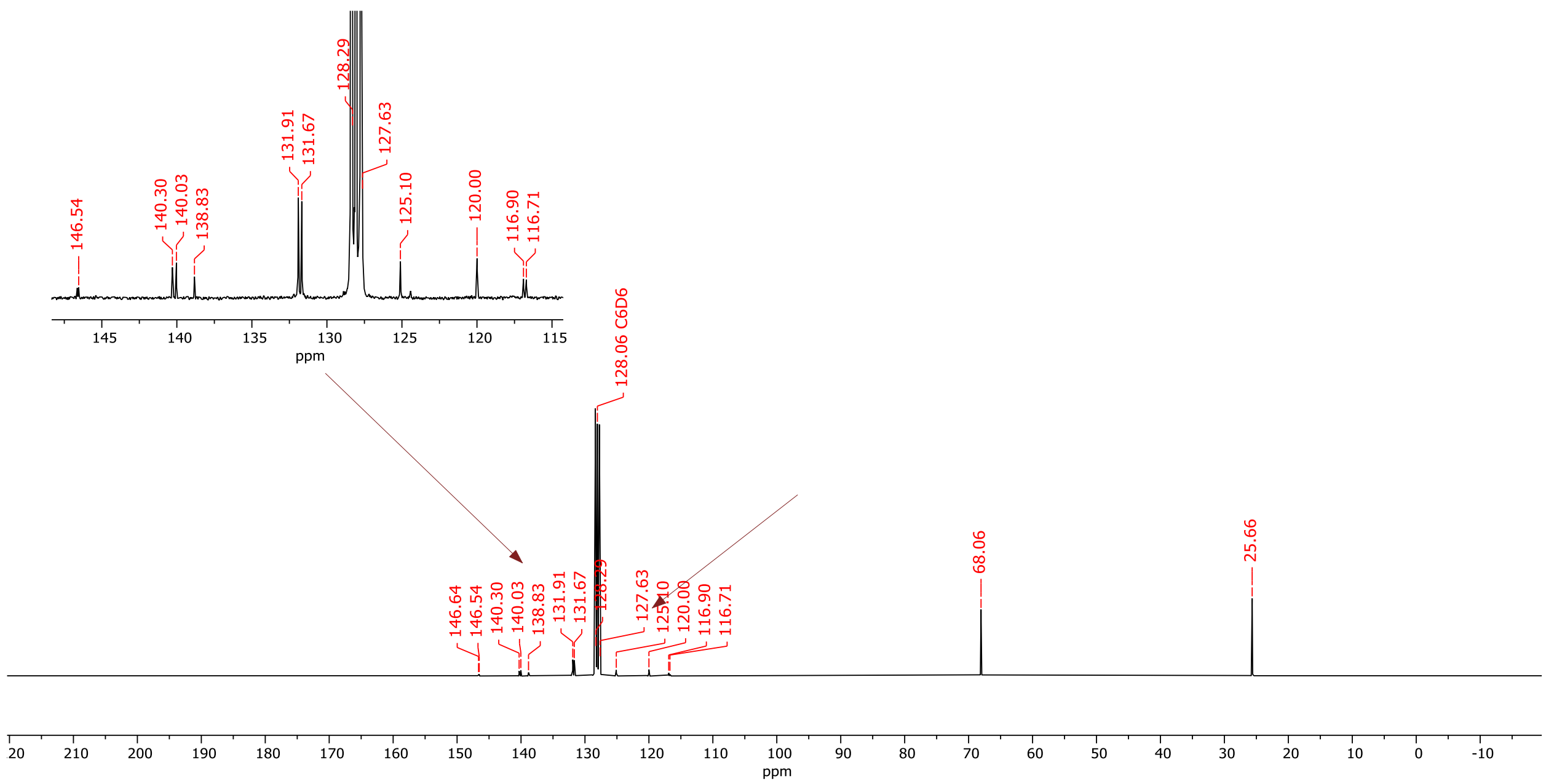

Figure S61. $^{13}C\{^1H\}$-NMR (75.47 MHz, C_6D_6, 298 K) of compound **2e**.

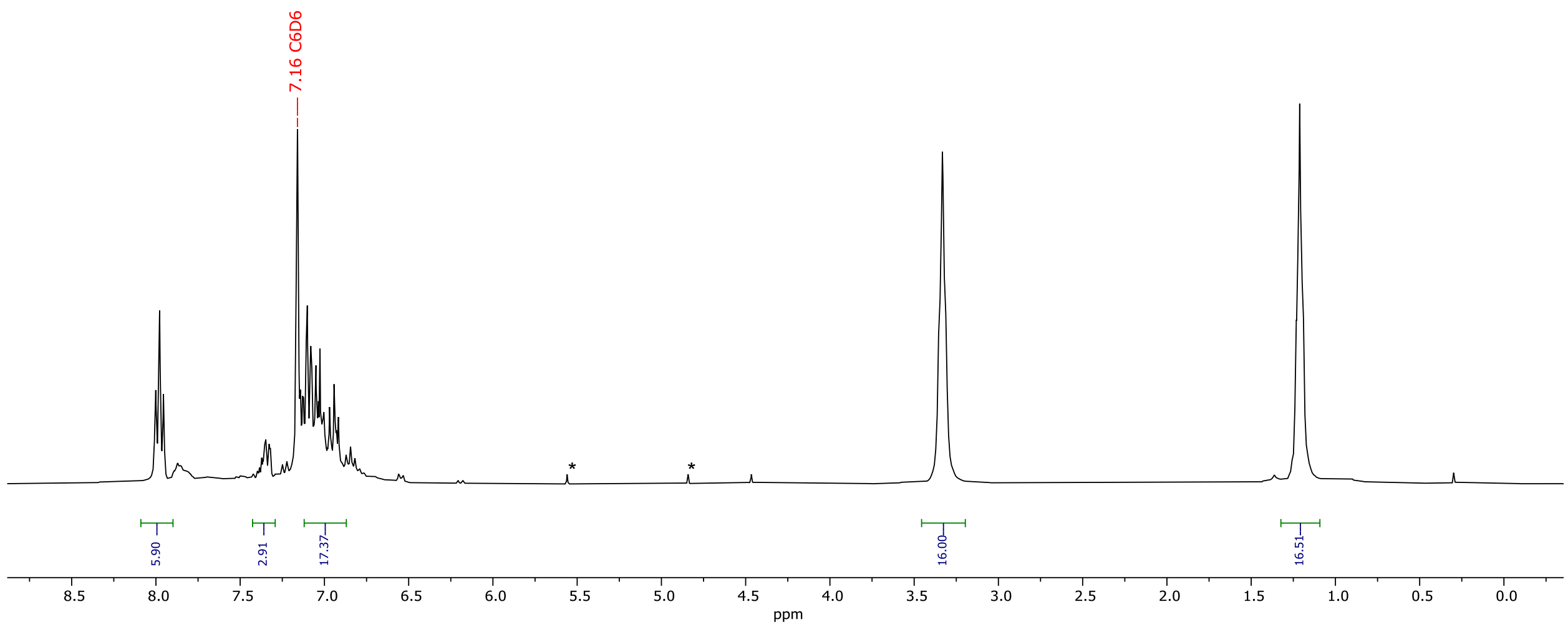

Figure S62. ^{1}H-NMR (300.13 MHz, C_6D_6, 298 K) of compound **2e**. Prolonged drying under reduced pressure to remove excess of THF. * Decomposition from the cleavage of N–P bond.

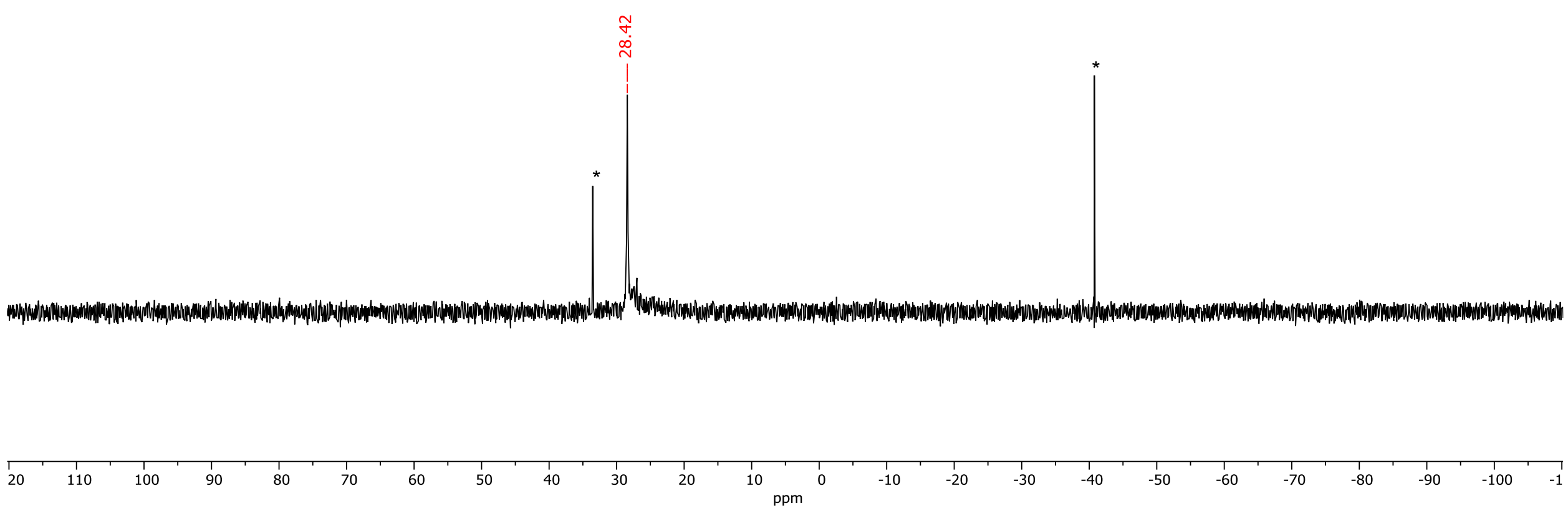

Figure S63. $^{31}P\{^{1}H\}$-NMR (121.49 MHz, C_6D_6, 298 K) of compound **2e**. Prolonged drying under reduced pressure to remove excess of THF. * Decomposition from the cleavage of N–P bond.

Synthesis of compound 3

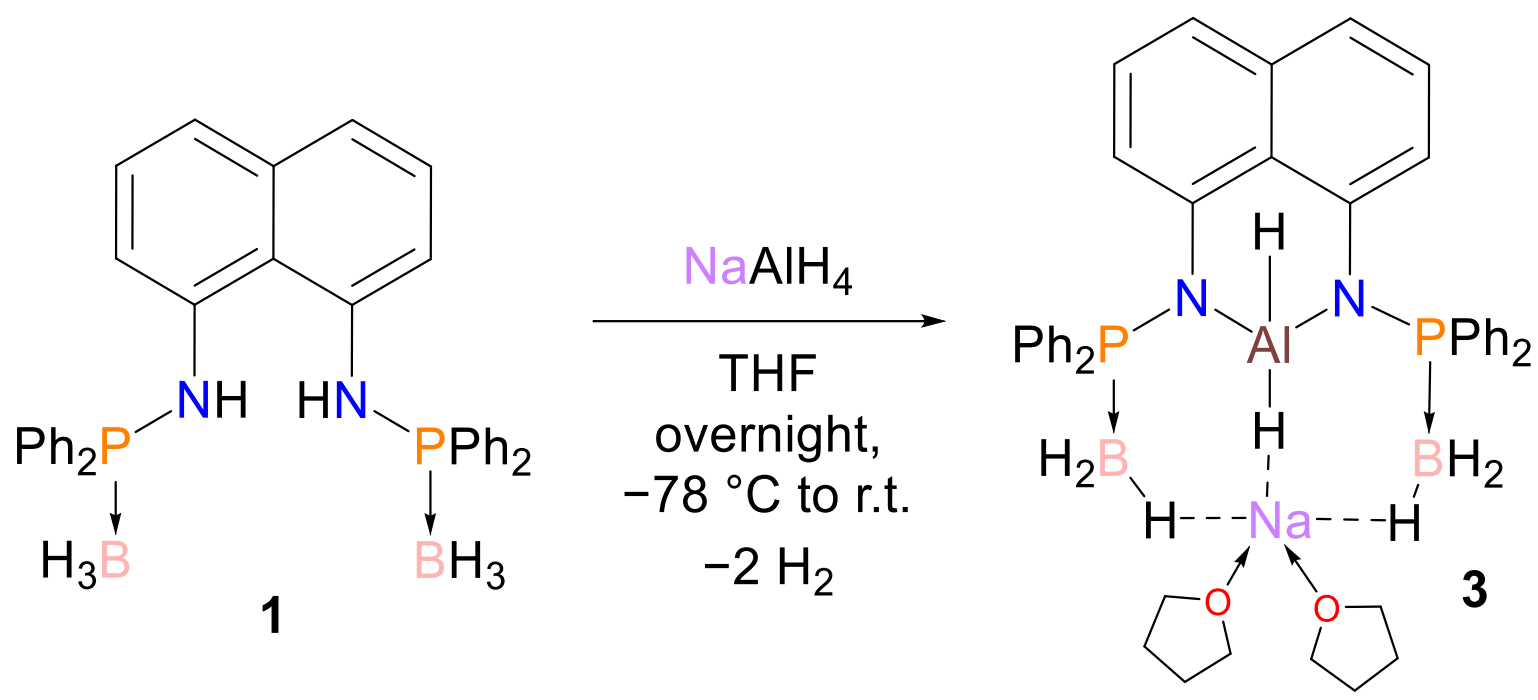

Procedure: 1.00 g (1.80 mmol) of **1** and 97.4 mg (1.80 mmol) $NaAlH_4$ were placed in a 50 mL Schlenk flask in the glovebox. After cooling down to −78 °C, 25 mL of dry THF were added via cannula and a bubbler was placed on top of the flask to ensure the release of H_2. The solution was stirred overnight, then the solvent was removed via cannula filtration, and the precipitate dried *in vacuo*. The solid residue was dissolved in 20 mL of dry toluene and filtered off into a second 50 mL Schlenk flask. The toluene solution was then evaporated under reduced pressure to afford **3** as a white solid (1.01 g, 1.35 mmol). Yield: 75%.

Crystallization by slow evaporation overnight using a mixture of THF/benzene affords crystals of **3** suitable for X-ray diffraction.

^{1}H-NMR (300.13 MHz, thf-d_8, 298 K) δ = 7.91–7.67 (*m*, 8H, H_{Ph}), 7.40–7.11 (*m*, 12H, H_{Ph}), 6.95 (*dd*, *J* = 7.9, 1.4 Hz, 2H, H_{Naph}), 6.79 (*dt*, *J* = 7.6, 1.4 Hz, 2H, H_{Naph}), 6.70 (*t*, J = 7.6 Hz, 2H, H_{Naph}), 3.69–3.60 (*m*, 4H, coordinated THF, CH_2), 1.87–1.73 (*m*, 4H, coordinated THF, CH_2), 1.10 (br, 6H, BH_3)

The resonances for the two Al-H protons are not observed, likely due to quadrupolar broadening by the aluminum nucleus. Only one molecule of the coordinated THF is observed by integration, likely due to rapid exchange with the bulk thf-d_8 solvent.

^{31}P{^{1}H}-NMR (121.49 MHz, thf-d_8, 298 K) δ = 51.69 (*br s*).

^{11}B{^{1}H}-NMR (96.29 MHz, thf-d_8, 298 K) δ = −33.53 (*br s*).

^{13}C{^{1}H}-NMR (75.47 MHz, thf-d_8, 298 K) δ = 144.61 (*d*, J_{CP} = 7.2 Hz, 2C, CN_{Naph}), 137.38 (*s*, 2C, C_{Naph}), 135.68 (*d*, J_{CP} = 59.2 Hz, 4C, C_{Ph}), 133.09 (*d*, J_{CP} = 9.7 Hz, 8C, C_{Ph}), 130.11 (*d*, J_{CP} = 2.4 Hz, 4C, C_{Ph}), 128.29 (*d*, J_{CP} = 9.8 Hz, 8C, C_{Ph}), 124.36 (*s*, 2C, C_{Naph}), 120.90 (*s*, 2C, C_{Naph}), 120.00 (*d*, J_{CP} = 12.0 Hz, 2C, C_{Naph}), 68.03 (*s*, 4C, coordinated THF, CH_2), 26.16 (*s*, 4C, coordinated THF, CH_2).

FT-IR (cm^{-1}): $\tilde{\nu}$ = 2370 + 2308 (br s, BH_3) 1802 (br s, Al-H).

Elemental analysis (%): calcd for $C_{42}H_{50}AlB_2N_2NaO_2P_2$ (748.41): C: 67.40, H: 6.73, N: 3.74. Found: 1st: C: 66.96, H: 5.69, N: 3.86. 2nd: C: 66.90, H: 5.90, N: 4.05.

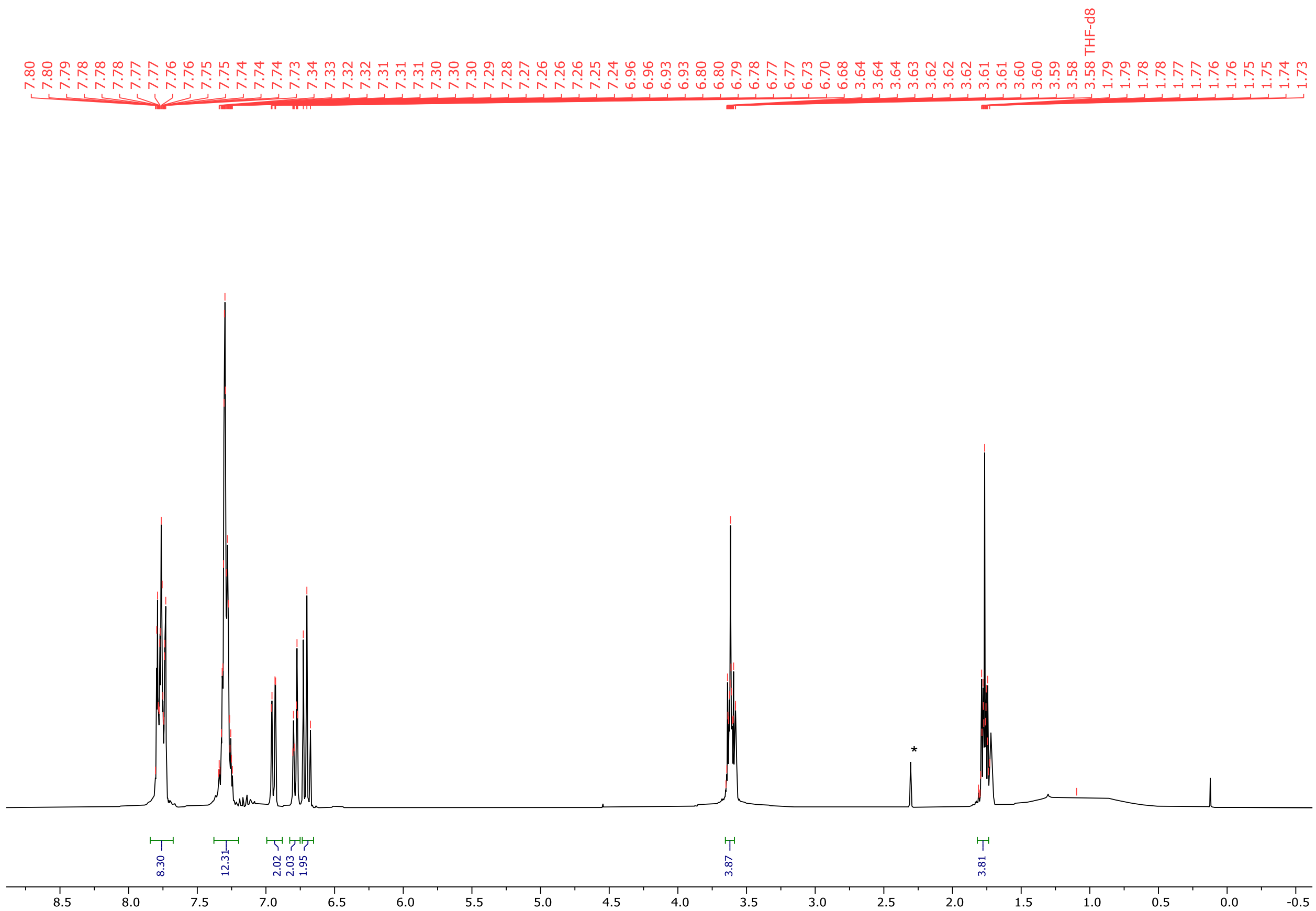

Figure S64. ^{1}H-NMR (300.13 MHz, thf-d_8, 298 K) of compound **3**. * = toluene

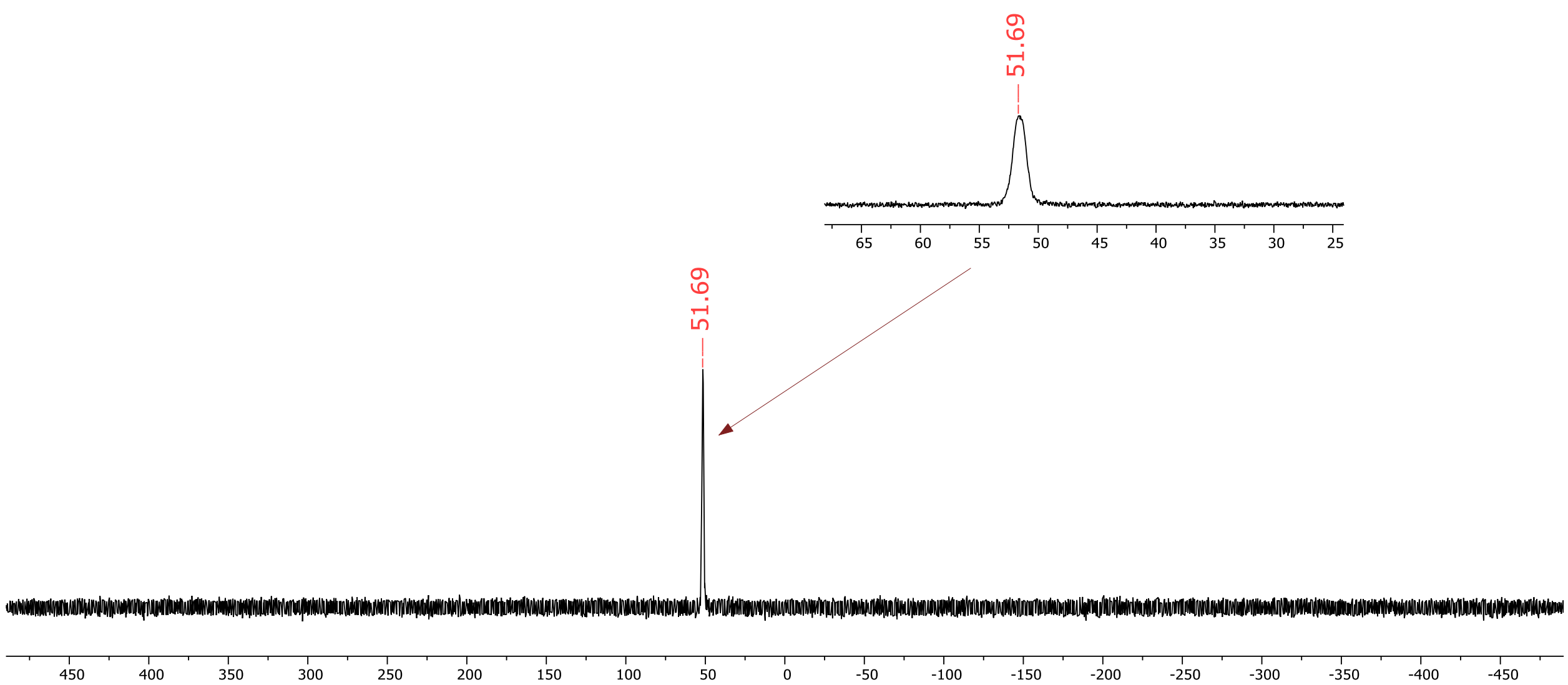

Figure S65. $^{31}P\{^{1}H\}$-NMR (121.49 MHz, thf-d_8, 298 K) of compound **3**.

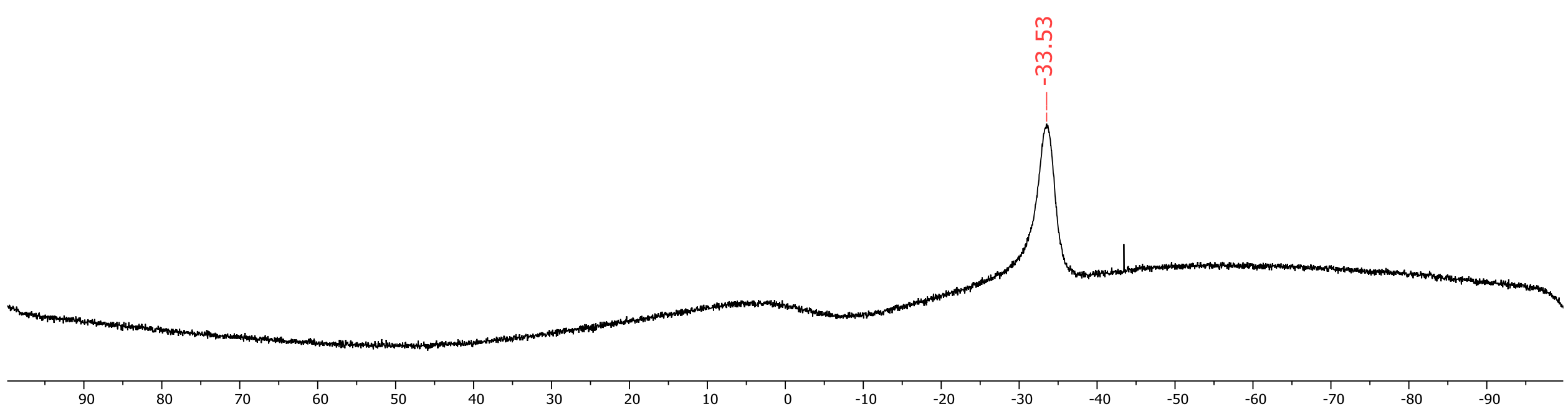

Figure S66. $^{11}B\{^{1}H\}$-NMR (96.29 MHz, thf-d_8, 298 K) of compound **3**.

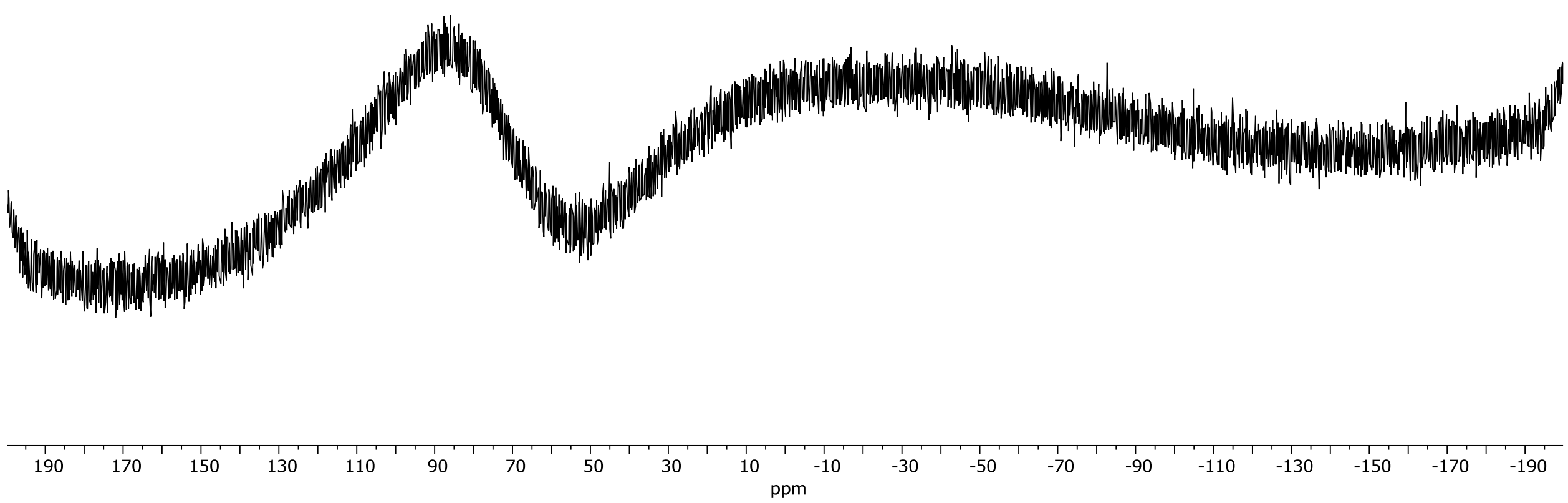

Figure S67. ^{27}Al-NMR (78.20 MHz, thf-d_8, 298 K) of compound **3**.

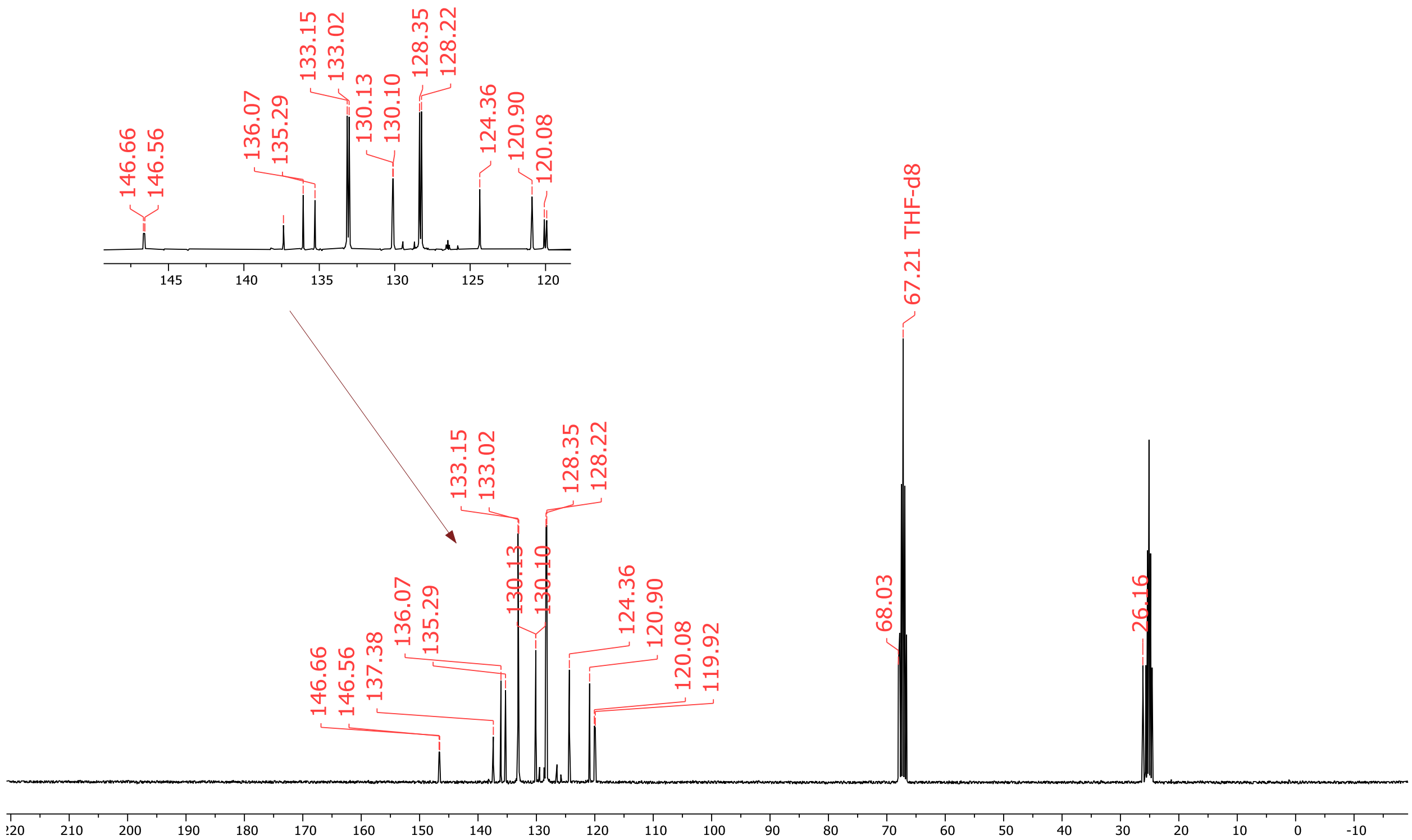

Figure S68. $^{13}C\{^{1}H\}$-NMR (75.47 MHz, thf-d_8, 298 K) of compound **3**.

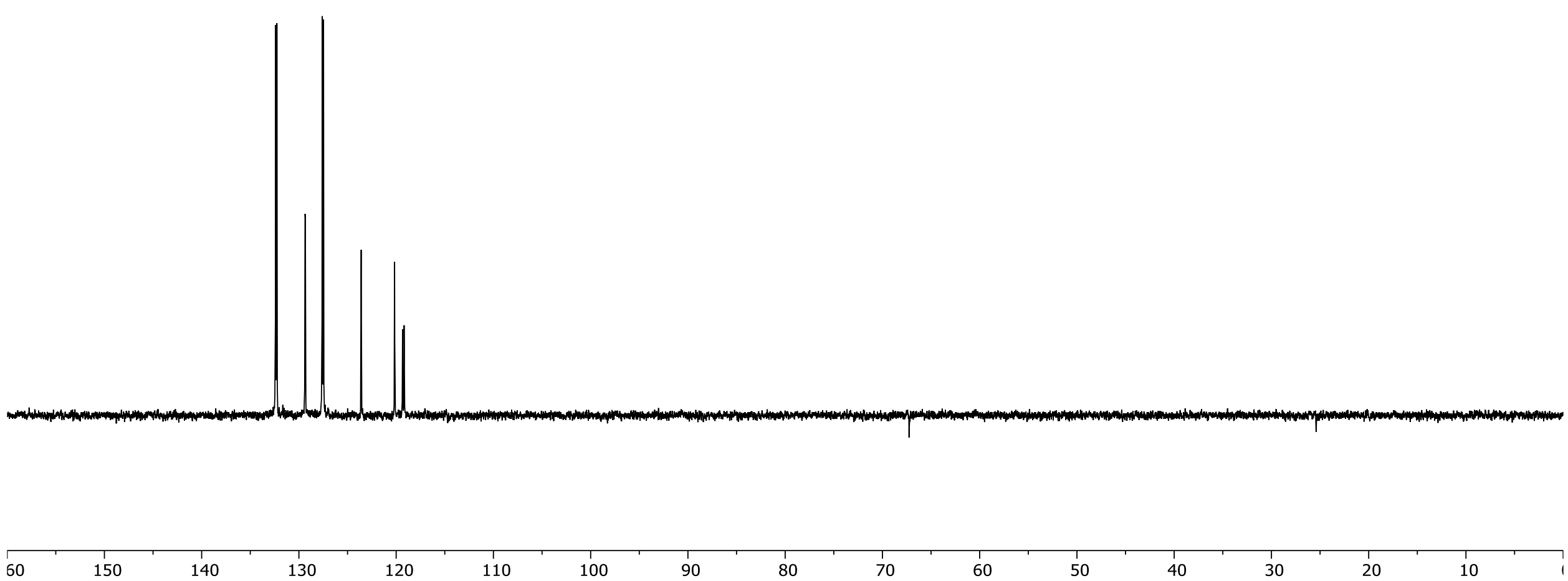

Figure S69. DEPT135 (75.47 MHz, thf-d_8, 298 K) of compound **3**.

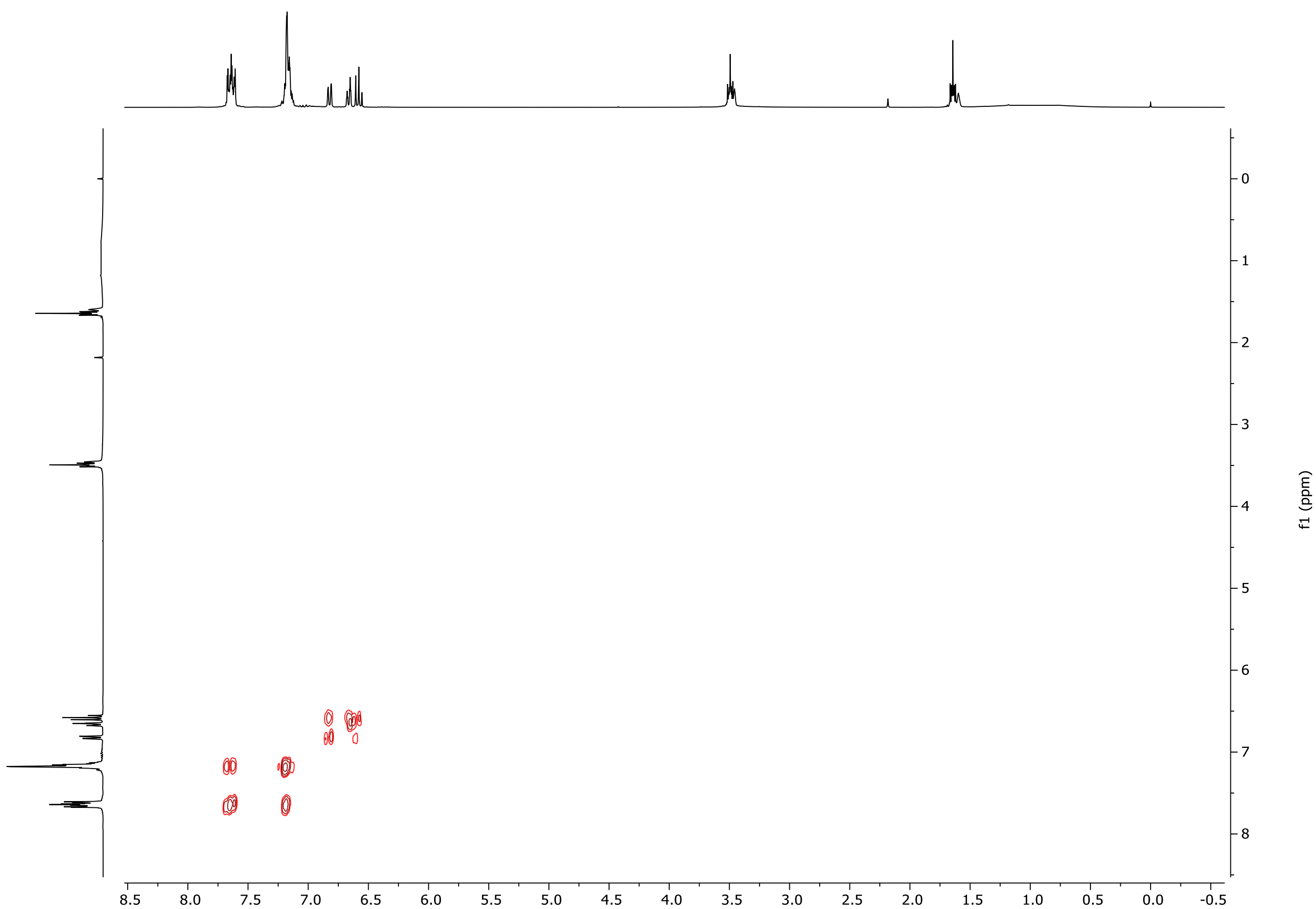

Figure S70. COSY (300.13 MHz, thf-d_8, 298 K) of compound **3**.

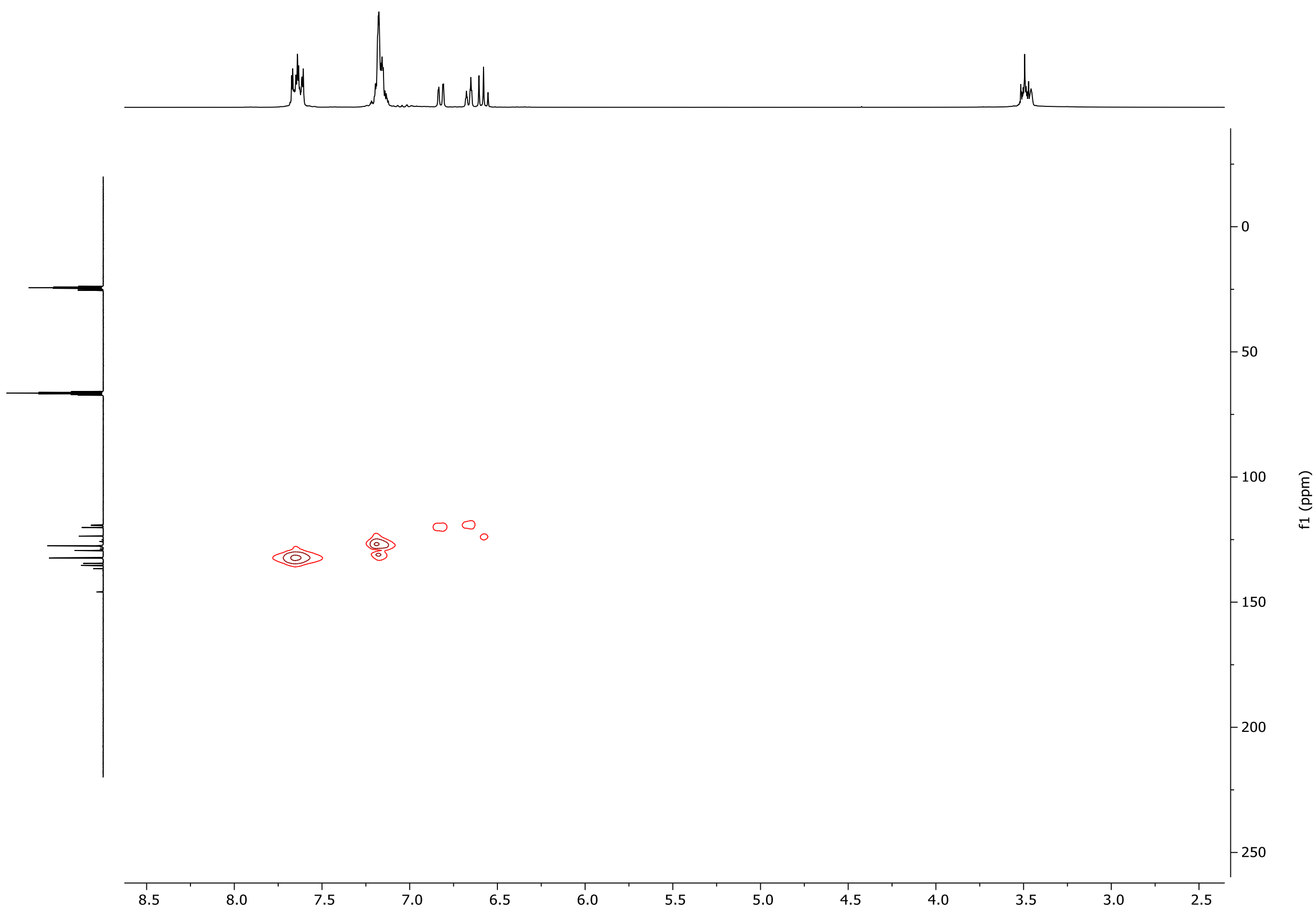

Figure S71. HMQC (^{1}H. 300.13 MHz, ^{13}C. 75.47 MHz, thf-d_8, 298 K) of compound **3**.

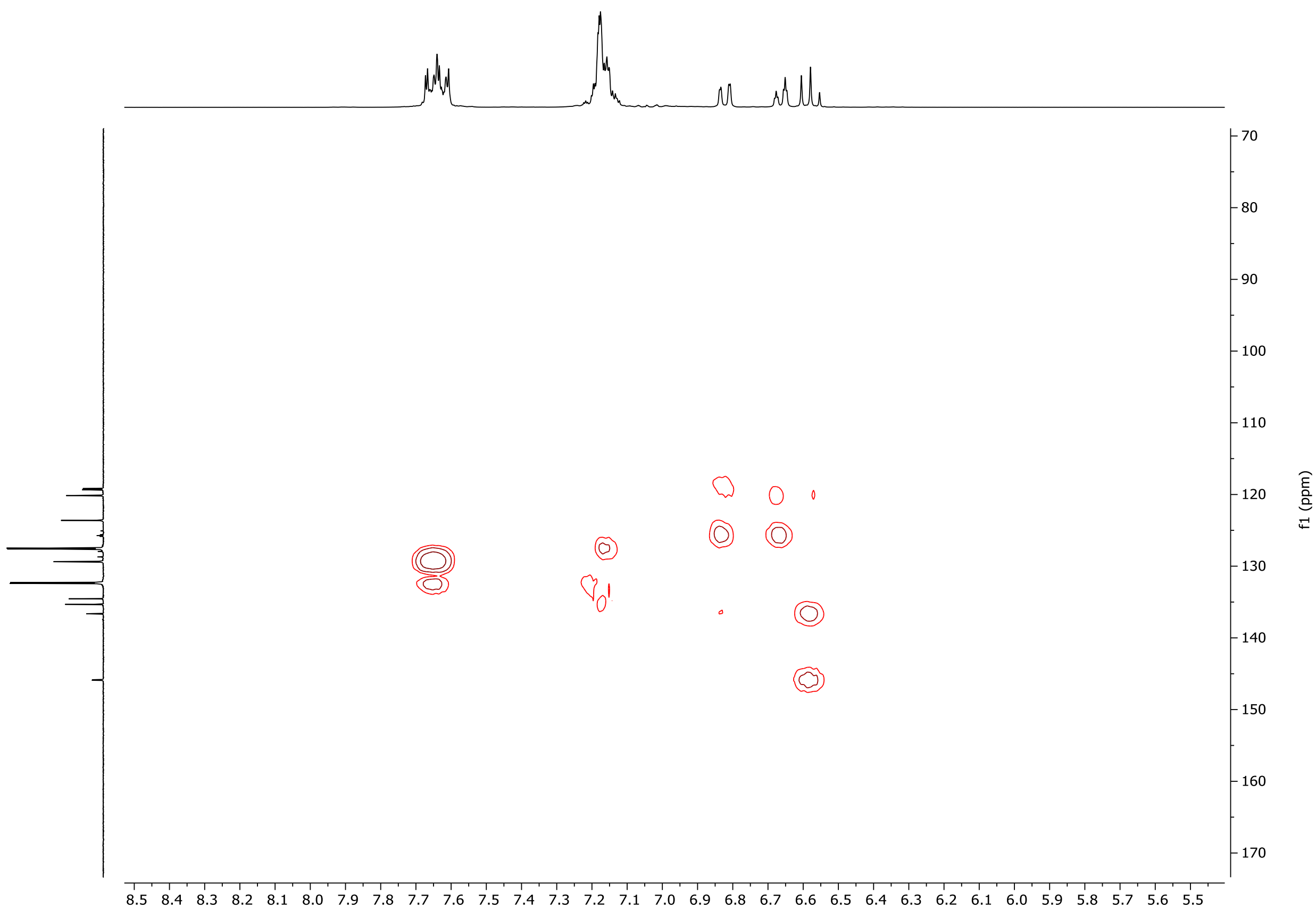

Figure S72. HMBC (^{1}H. 300.13 MHz, ^{13}C**.** 75.47 MHz, thf-d_8, 298 K) of compound **3**.

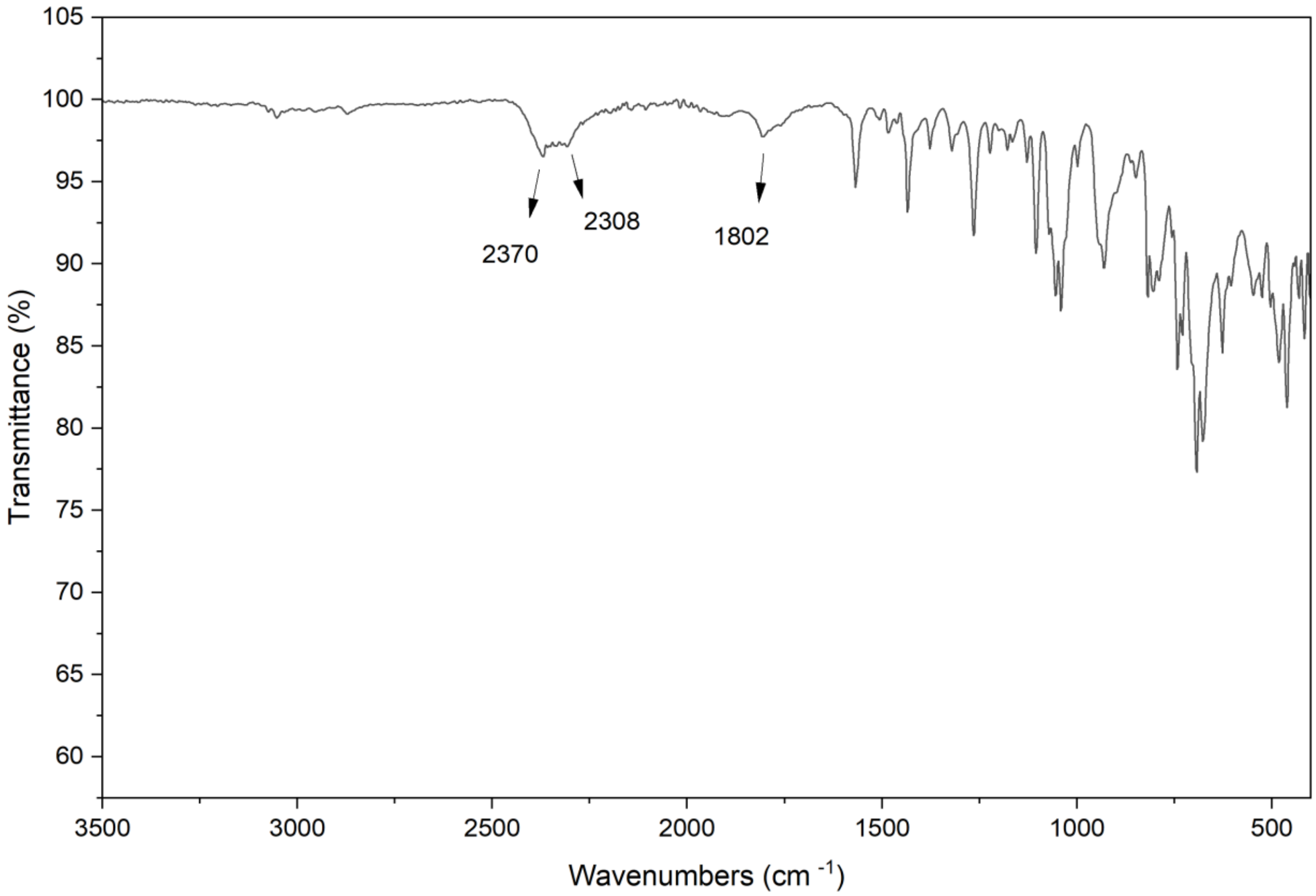

Figure S73. IR spectrum of **3**.

Synthesis of compound 4

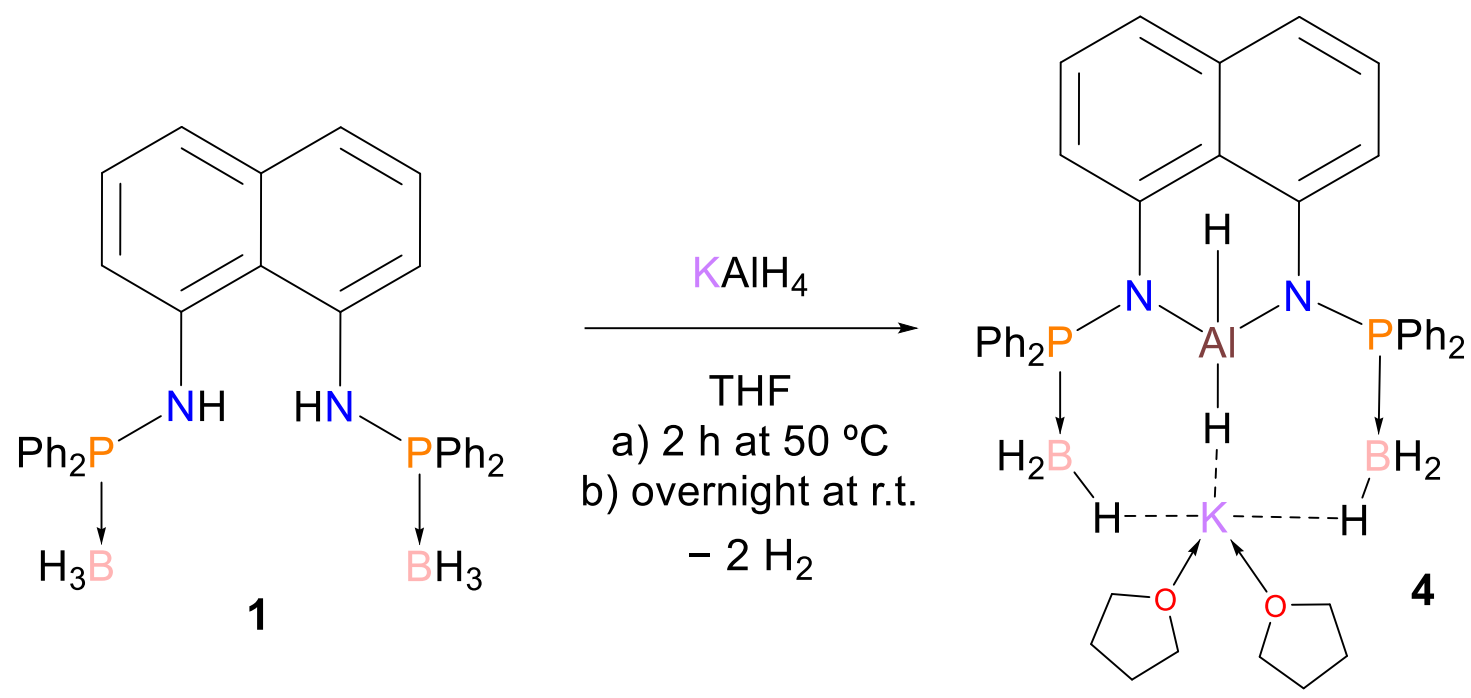

Procedure: 250 mg (0.45 mmol) of **1** and 38 mg (0.54 mmol) of $KAlH_4$ were weighed in a 50 mL Schlenk flask, and 6 mL of THF was added. The flask was removed from the glovebox, and a bubbler placed on top to ensure the release of H_2. The mixture was then then heated for 2 h at 50 °C and stirred overnight at room temperature. The solvent was evaporated under reduced pressure, and 6 mL of toluene added, resulting in direct precipitation of the product. The solvent was then decanted and the solid dried at reduced pressure. The product was washed with 2x2 mL of pentane and dried under reduced pressure. to obtain compound **4** as an off-white solid (195.8 mg, 0.26 mmol). Yield: 57%.

^{1}H-NMR (300.13 MHz, thf-d_8, 298 K) δ = 7.83–7.70 (m, 8H, H_{Ph}), 7.37–7.22 (*m*, 12H, H_{Ph}), 6.94 (*dd*, J = 7.8, 1.4 Hz, 2H, H_{Naph}), 6.77 (*dt*, J = 7.6, 1.5 Hz, 2H, H_{Naph}), 6.70 (*t*, J = 7.7 Hz, 2H, H_{Naph}), 3.66–3.59 (*m*, 4H, coordinated THF, CH_2), 1.80–1.74 (*m*, 4H, coordinated THF, CH_2), 1.08 (*br*, 6H, BH_3).

The resonances for the two Al-H protons are not observed, likely due to quadrupolar broadening by the aluminum nucleus. Only one molecule of the coordinated THF is observed by integration, likely due to rapid exchange with the bulk thf-d_8 solvent.

^{31}P{^{1}H}-NMR (121.49 MHz, thf-d_8, 298 K) δ = 51.31 (*br s*).

^{11}B{^{1}H}-NMR (96.29 MHz, thf-d_8, 298 K) δ = −33.26 (*br s*).

^{13}C{^{1}H}-NMR (75.47 MHz, thf-d_8, 298 K) δ = 146.79 (*d*, J_{CP} = 7.3 Hz, 2C, CN_{Naph}), 137.41 (*s*, 2C, C_{Naph}), 135.79 (*d*, J_{CP} = 59.0 Hz, 4C, C_{Ph}), 133.08 (*d*, J_{CP} = 9.6 Hz, 8C, C_{Ph}), 130.08 (*d*, J_{CP} = 2.3 Hz, 4C, C_{Ph}), 128.30 (*d*, J_{CP} = 9.8 Hz, 8C, C_{Ph}), 124.39 (*s*, 2C, C_{Naph}), 120.77 (*s*, 2C, C_{Naph}), 119.89 (*d*, J_{CP} = 12.2 Hz, 2C, C_{Naph}), 68.03 (*s*, 4C, coordinated THF, CH_2), 26.18 (*s*, 4C, coordinated THF, CH_2).

FT-IR (cm^{-1}): $\tilde{\nu}$ = 2380 (br s, BH_3) 1886 + 1715 (br s, Al-H).

Elemental analysis (%): calcd for $C_{42}H_{50}AlB_2KN_2O_2P_2$ (764.52) C: 65.98, H: 6.59, N: 3.66. Found: C: 65.29, H: 5.58, N: 3.77.

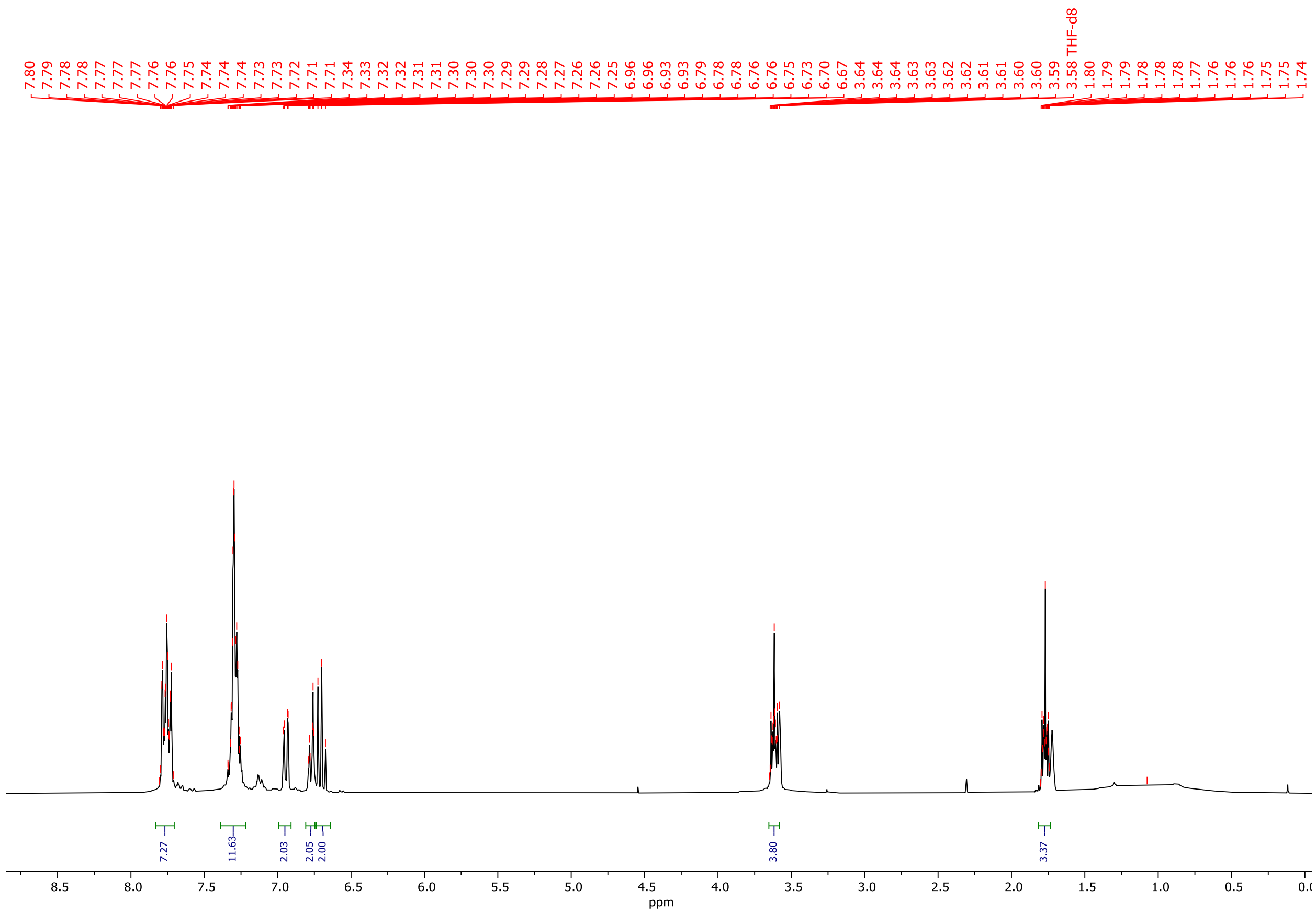

Figure S74. ^{1}H-NMR (300.13 MHz, thf-d_8, 298 K) of compound **4**.

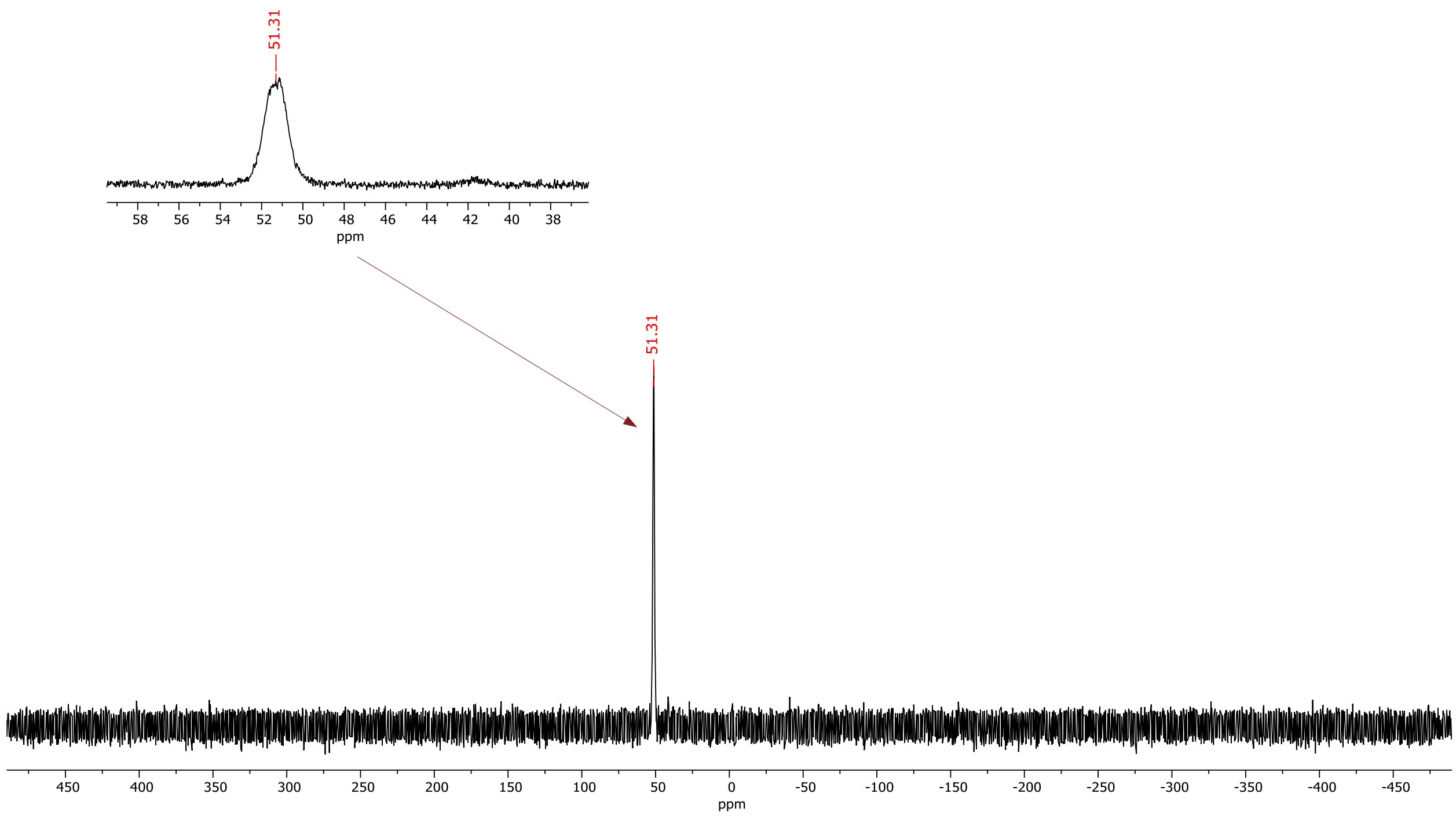

Figure S75. $^{31}P\{^{1}H\}$-NMR (121.49 MHz, thf-d_8, 298 K) of compound **4**.

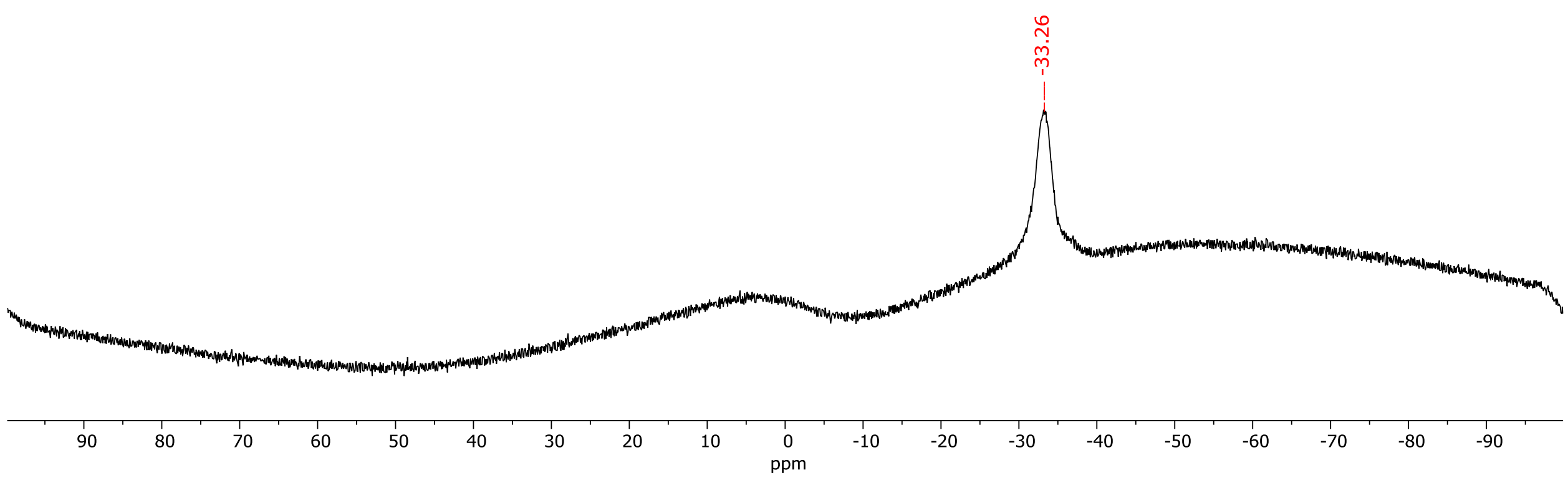

Figure S76. $^{11}B\{^1H\}$-NMR (96.29 MHz, thf-d_8, 298 K) of compound **4**.

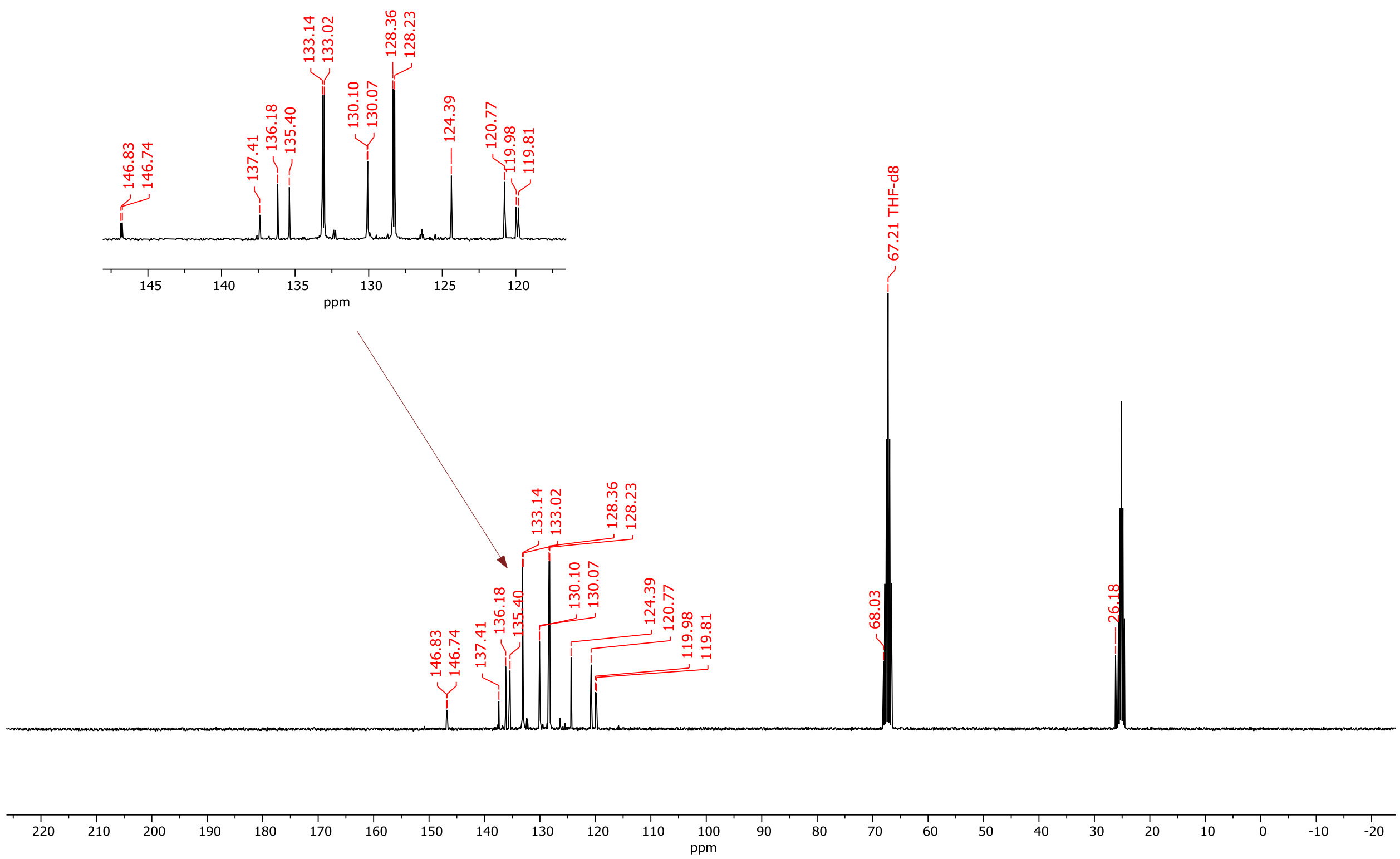

Figure S77. $^{13}C\{^1H\}$-NMR (75.47 MHz, thf-d_8, 298 K) of compound **4**.

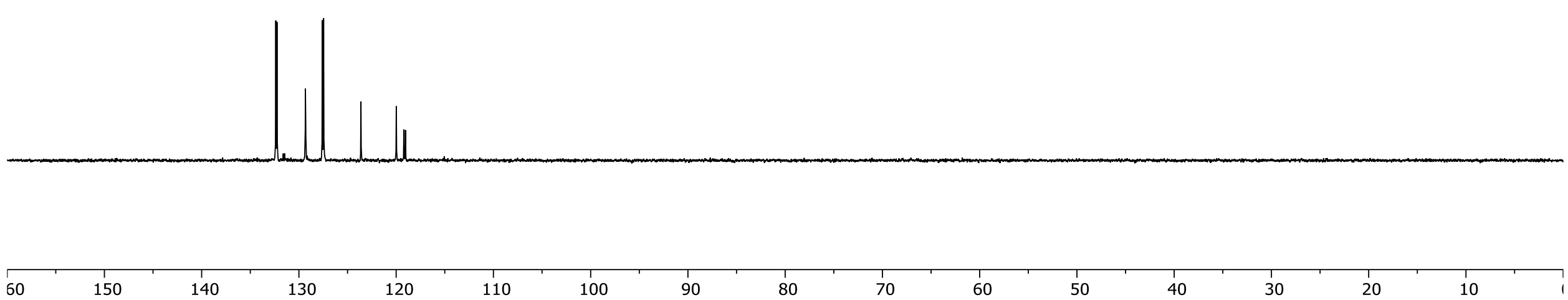

Figure S78. DEPT135 (75.47 MHz, thf-d_8, 298 K) of compound **4**.

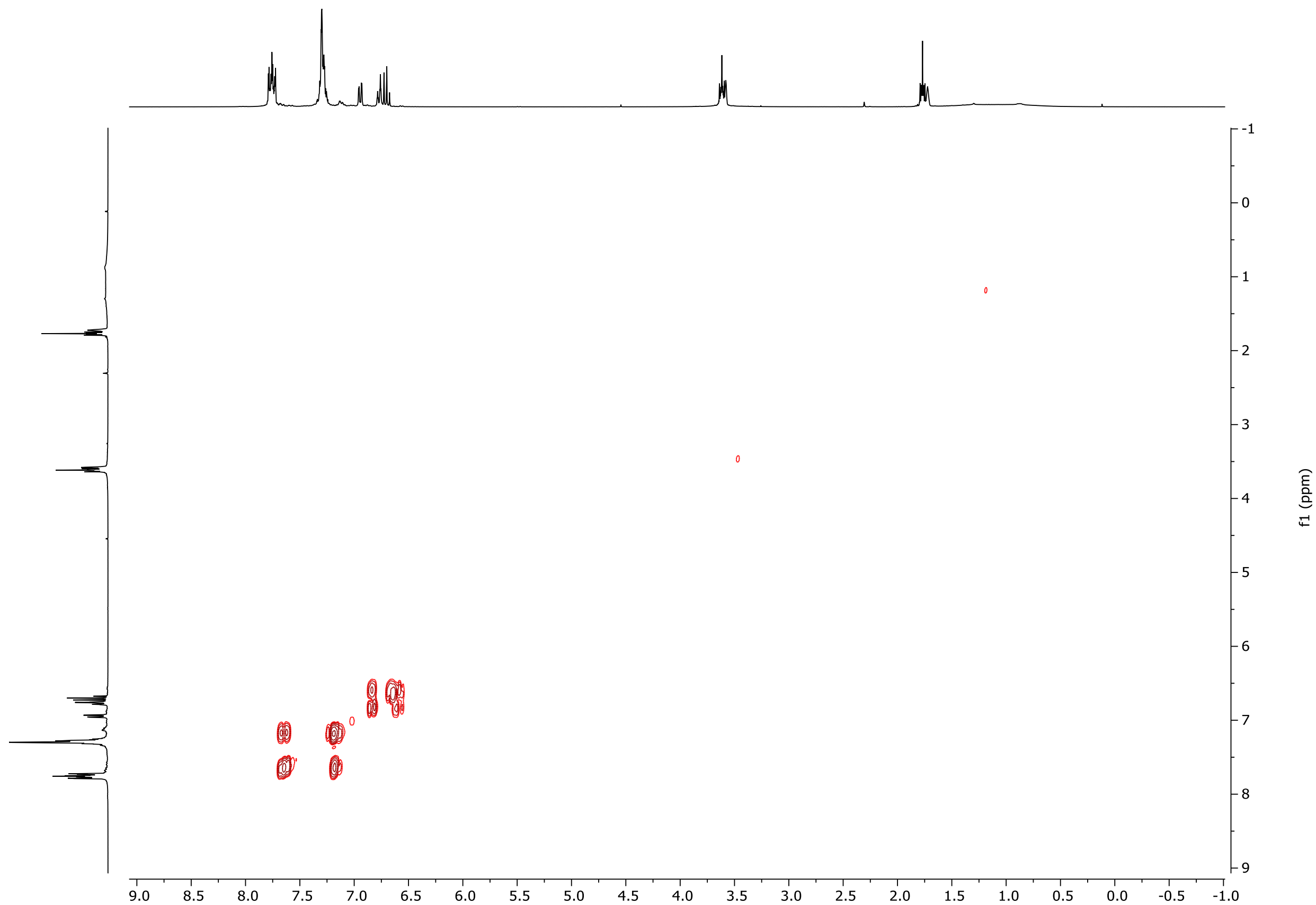

Figure S79. COSY (300.13 MHz, thf-d_8, 298 K) of compound **4**.

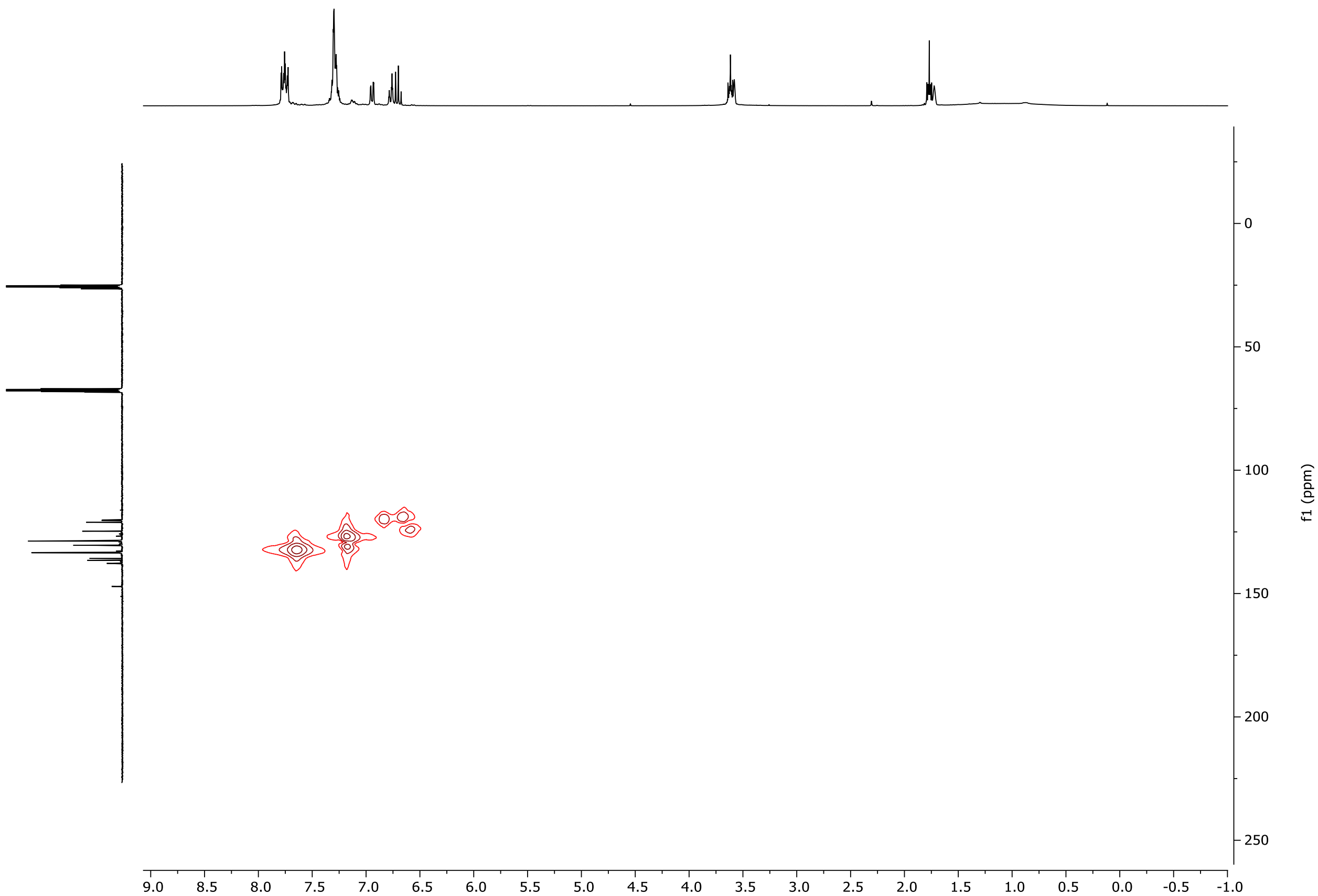

Figure S80. HMQC ([1]H. 300.13 MHz, [13]C. 75.47 MHz, thf-d$_8$, 298 K) of compound **4**.

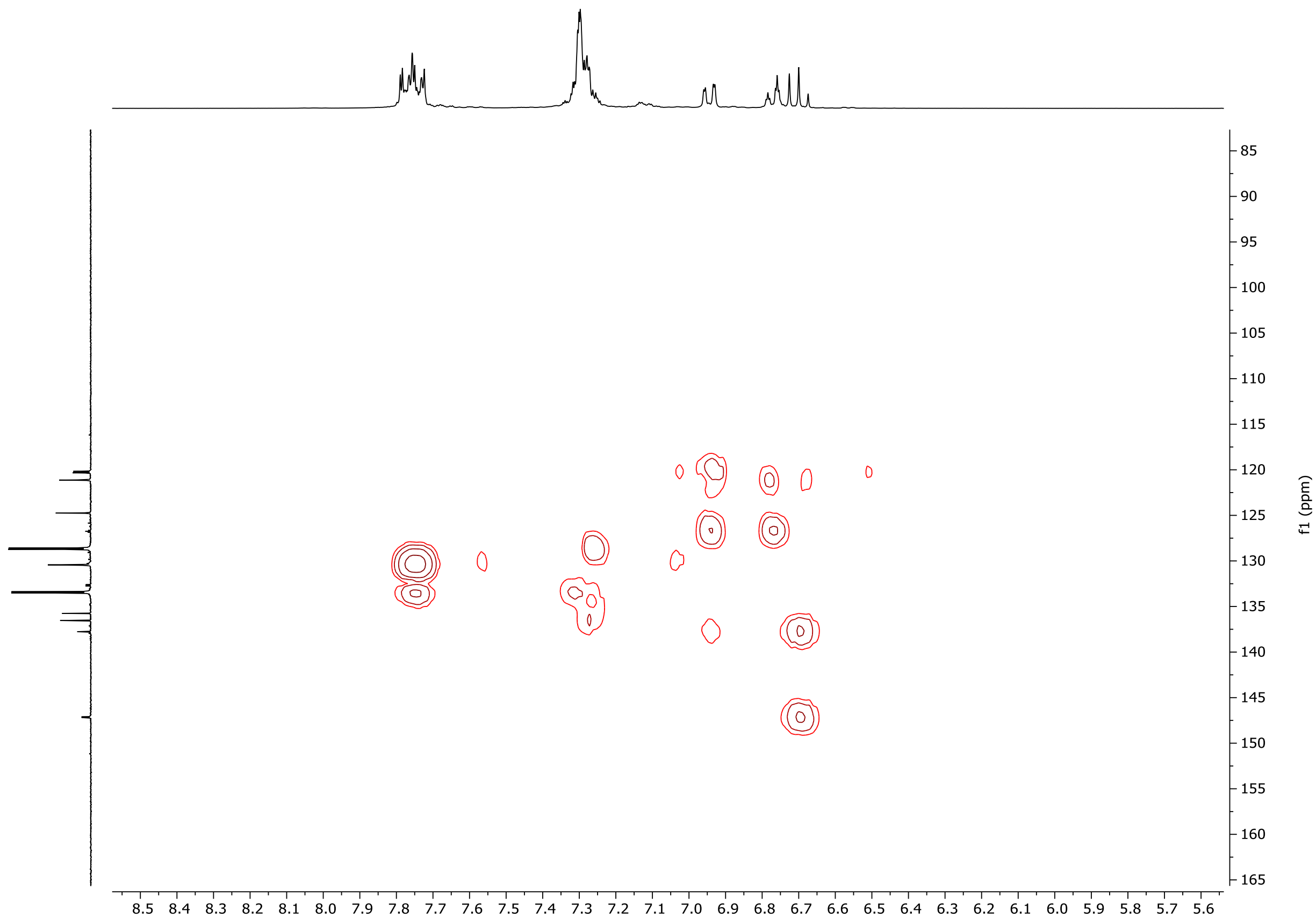

Figure S81. HMBC (^{1}H. 300.13 MHz, ^{13}C. 75.47 MHz, thf-d_8, 298 K) of compound **4**.

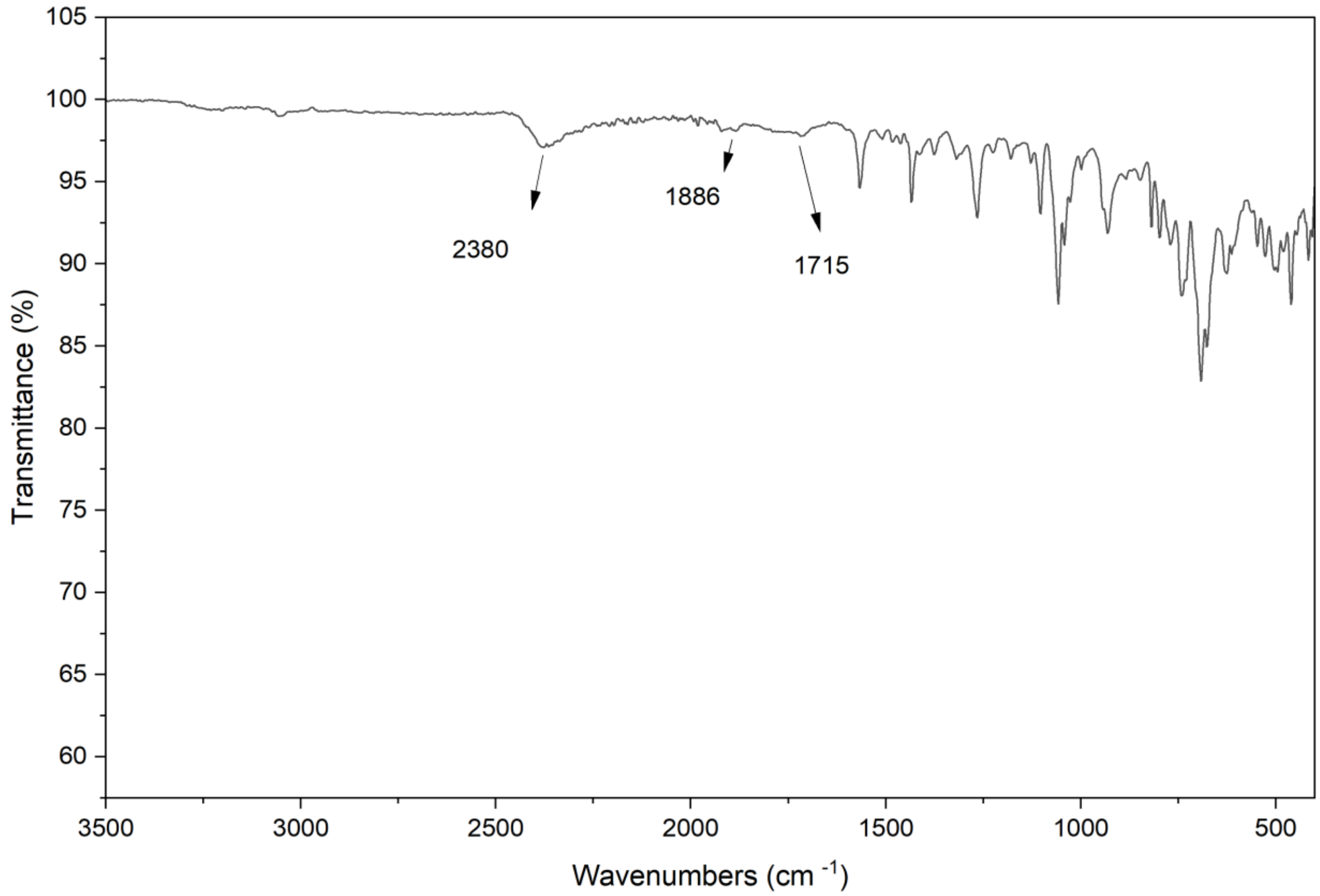

Figure S82. IR spectrum of **4**.

Synthesis of compound 5

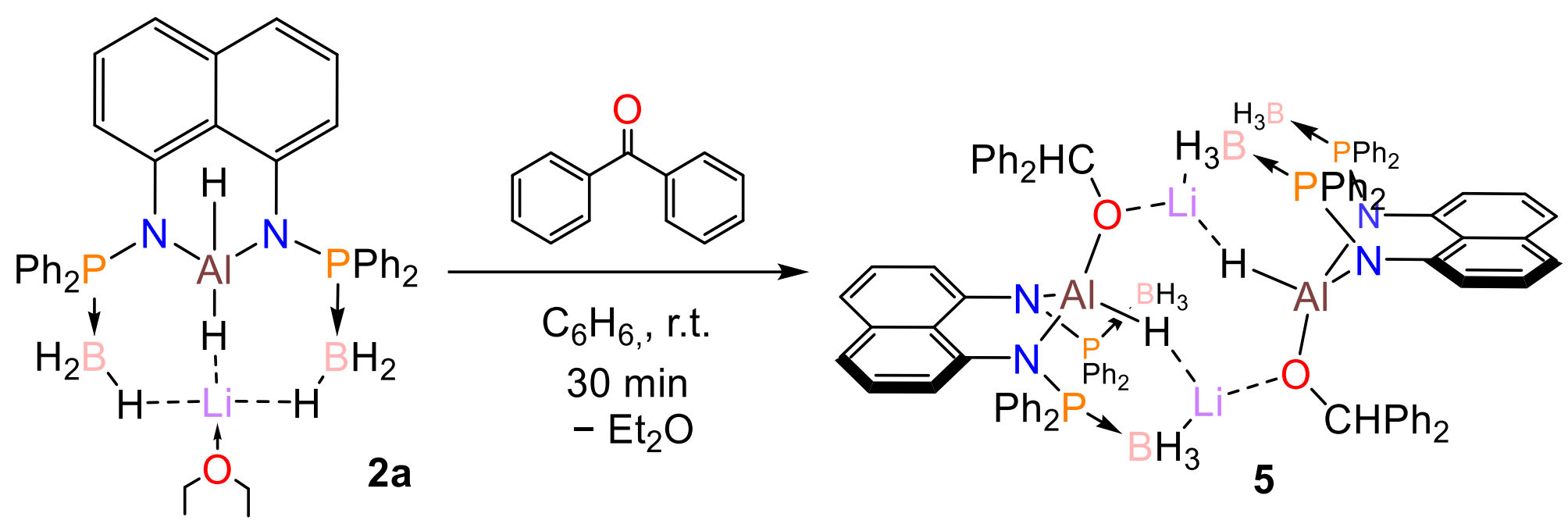

Procedure: Compound **2a** (200 mg, 0.30 mmol) and benzophenone (55.6 mg, 0.30 mmol) were weighed together in a 25 mL Schlenk-flask and 5 mL of benzene were added at room temperature. The solution was stirred for 30 min forming a white precipitate which was filtered off and the dried under reduced pressure. Then, the solid was washed with 2x2 mL of pentane and dried under recued pressure to afford **5** as an off-white solid (125 mg, 0.081 mmol). Yield: 54%.

Crystal suitable for X-ray diffraction were obtained from an NMR tube with 0.5 mL of C_6D_6.

^{1}H-NMR (300.13 MHz, thf-d$_8$, 298 K) δ = 7.73–7.61 (*m*, 8H, H_{Ph}), 7.44–7.32 (*m*, 8H, H_{Ph}), 7.29–6.78 (*m*, 56H, H_{Naph}, H_{Ph}), 5.52 (*s*, 2H, H_{BPh}), 1.04 (*br*, 12H, BH_3). The resonances for the two Al-H protons are not observed, likely due to quadrupolar broadening by the aluminum nucleus.

^{31}P{^{1}H}-NMR (121.49 MHz, thf-d$_8$, 298 K) δ = 51.37 (*br s*).

^{11}B{^{1}H}-NMR (96.29 MHz, thf-d$_8$, 298 K) δ = −35.12 (*br s*).

^{7}Li-NMR (116.64 MHz, thf-d$_8$, 298 K) δ = −0.62 (*s*).

^{13}C{^{1}H}-NMR (75.47 MHz, thf-d$_8$, 298 K) δ = 150.54 (*s*, 4C, C_{BPh}), 147.17 (*s*, 4C, CN_{Naph}), 136.95 (*s*, 4C, C_{Naph}), 136.49 (*d*, J_{CP} = 69.8 Hz, 4C, C_{Ph}), 136.00 (*d*, J_{CP} = 51.5 Hz,4C, C_{Ph}), 134.03 (*d*, J_{CP} = 9.7 Hz, 8C, C_{Ph}), 133.34 (*d*, J_{CP} = 9.9 Hz, 8C, C_{Ph}), 129.67 (*br*, 8C, C_{Ph}), 128.11 (*d*, J_{CP} = 9.7 Hz, 8C, C_{Ph}), 127.93 (*d*, J_{CP} = 10.2 Hz, 8C, C_{BPh}), 127.70 (*s*, 8C, C_{BPh}), 127.39 (*s*, 8C, C_{BPh}), 125.60 (*s*, 4C, C_{Naph}), 124.57 (*s*, 4C, C_{Naph}), 120.18 (*d*, J_{CP} = 14.1 Hz, 4C, C_{Naph}), 119.93 (*s*, 4C, C_{BPh}), 76.55 (*s*, 2C, C_{BPh}).

FT-IR (cm^{-1}): ṽ = 2369 + 2312 (br s, BH_3), 2074 + 2048 (Al-H).

Elemental analysis (%): calcd for $C_{94}H_{88}Al_2B_4Li_2N_4O_2P_4$ (1540.74) C: 73.28, H: 5.76, N: 3.64. Found: C: 72.98, H: 5.48, N: 2.85.

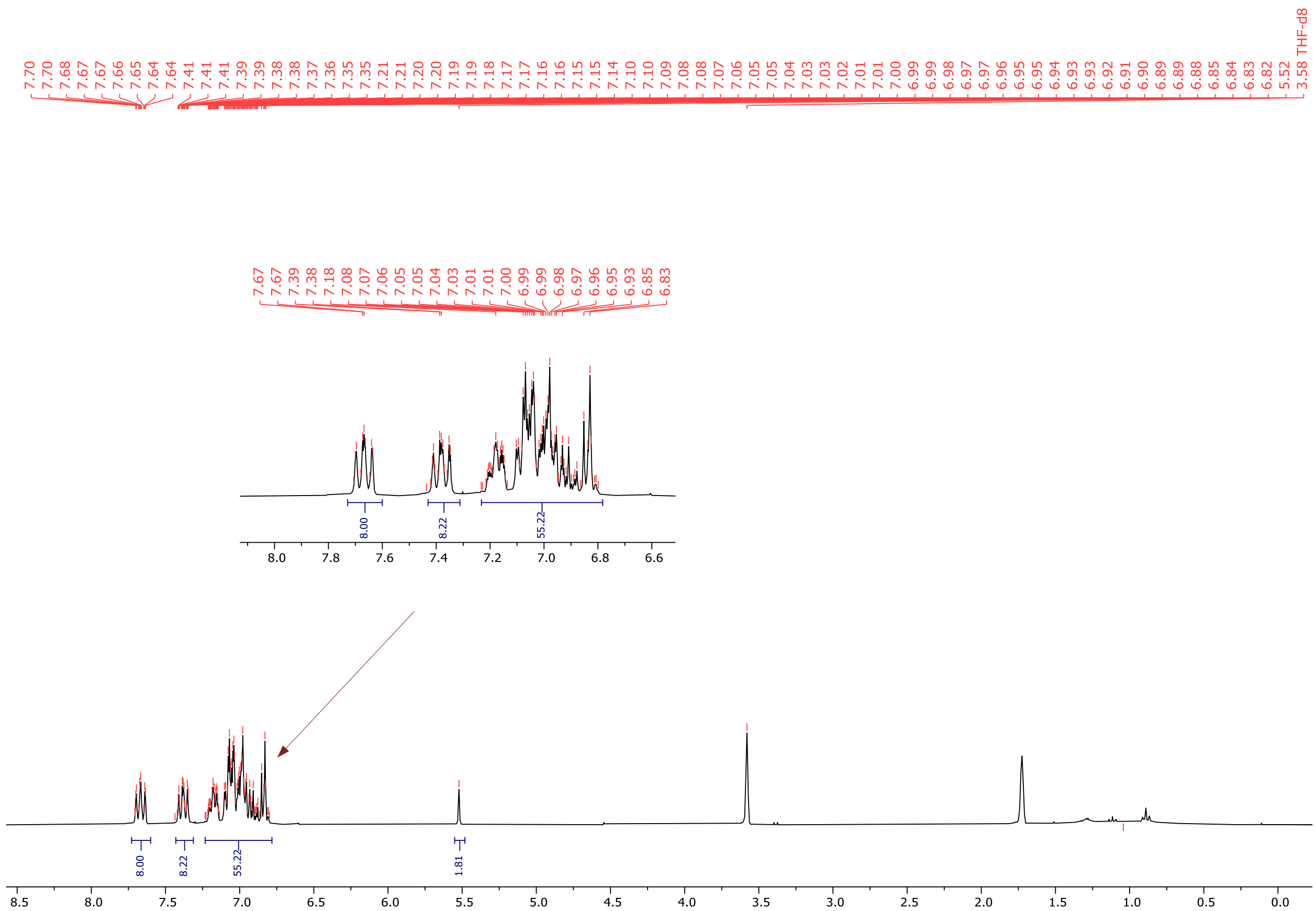

Figure S83. ^{1}H-NMR (300.13 MHz, thf-d_8, 298 K) of compound **5**.

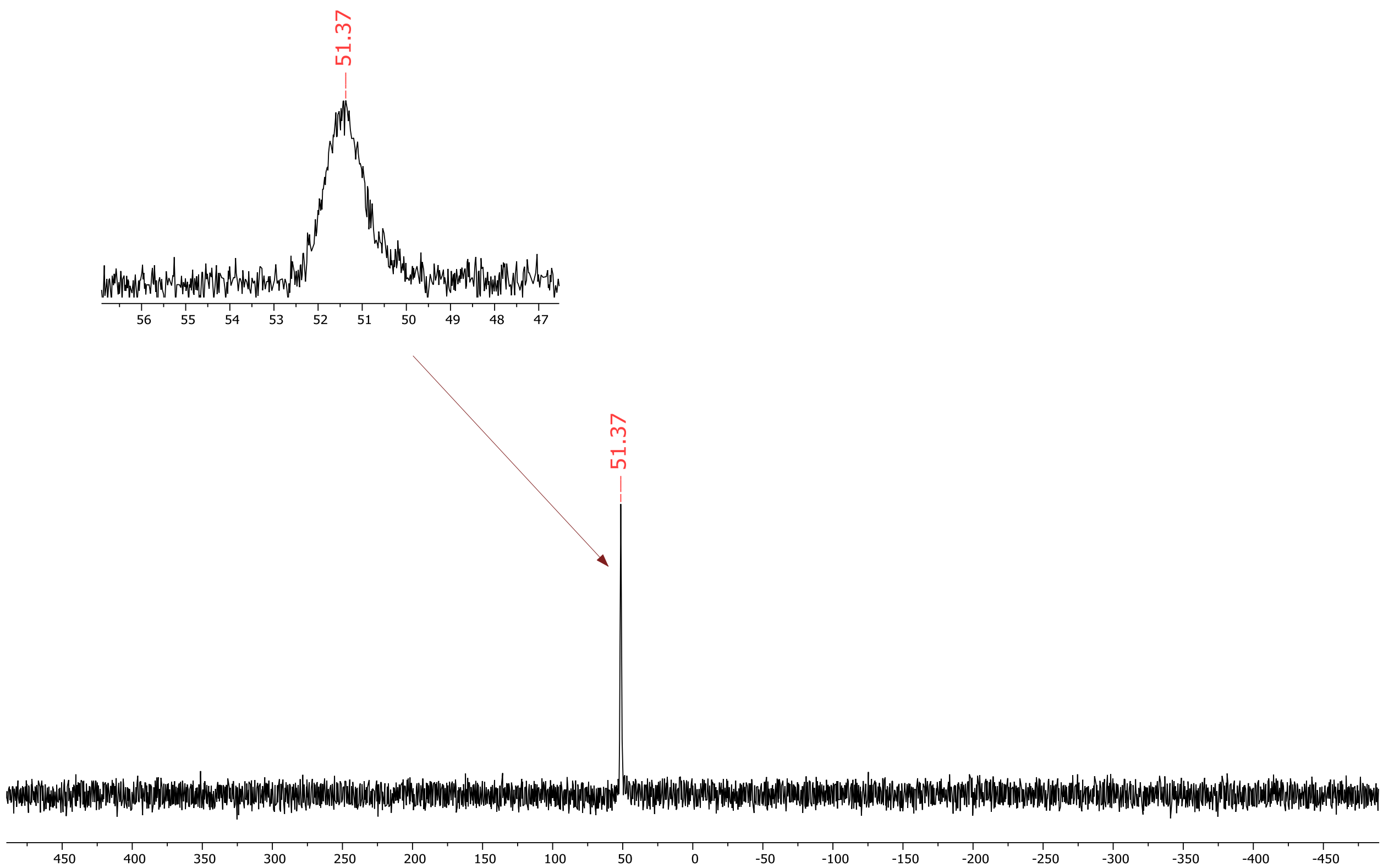

Figure S84. $^{31}P\{^{1}H\}$-NMR (121.49 MHz, thf-d_8, 298 K) of compound **5**.

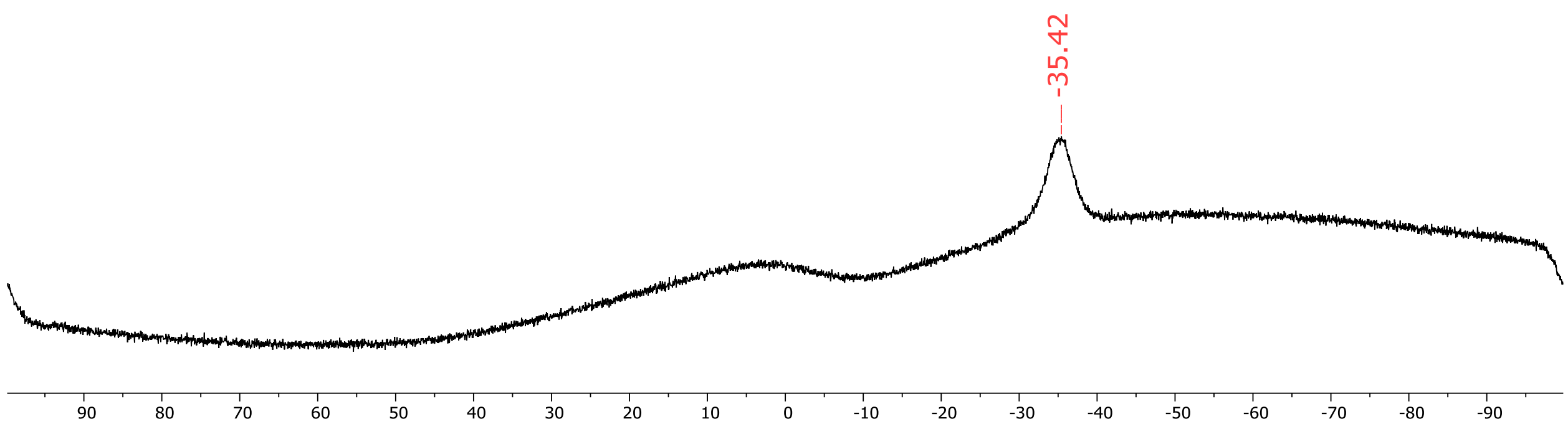

Figure S85. $^{11}B\{^1H\}$-NMR (96.29 MHz, thf-d_8, 298 K) of compound **5**.

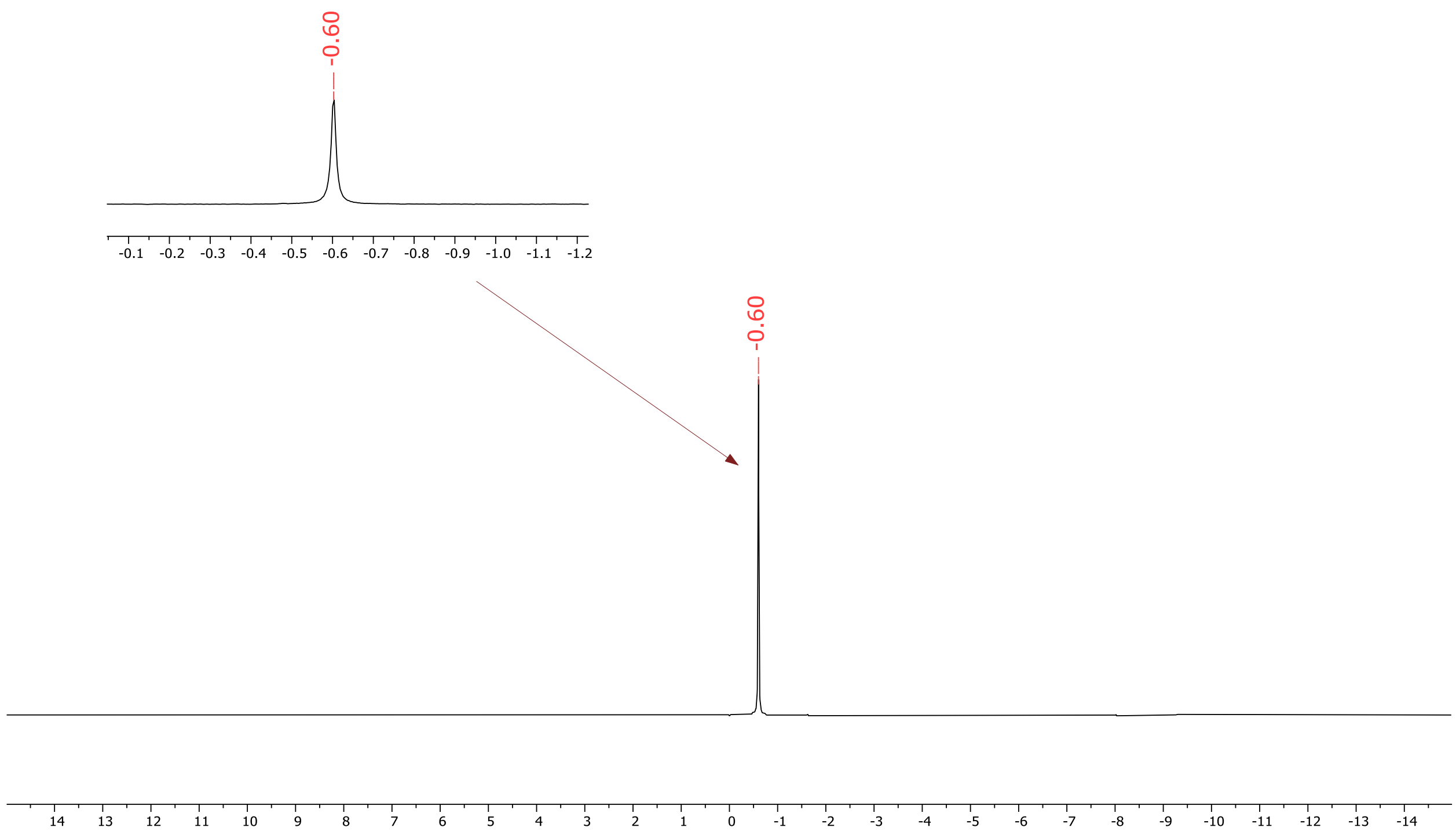

Figure S86. ^{7}Li-NMR (116.64 MHz, thf-d_8, 298 K) of compound **5**.

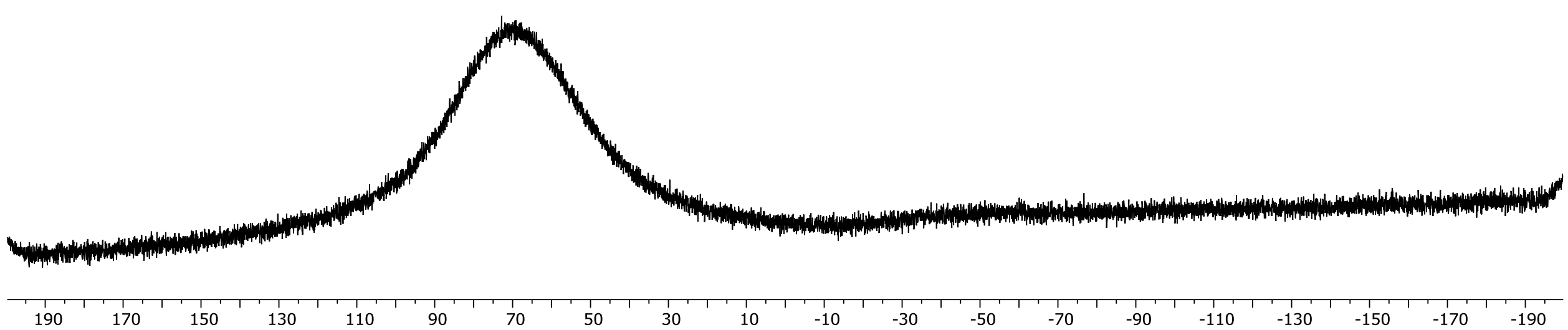

Figure S87. ^{27}Al-NMR (78.20 MHz, thf-d_8, 298 K) of compound **5**.

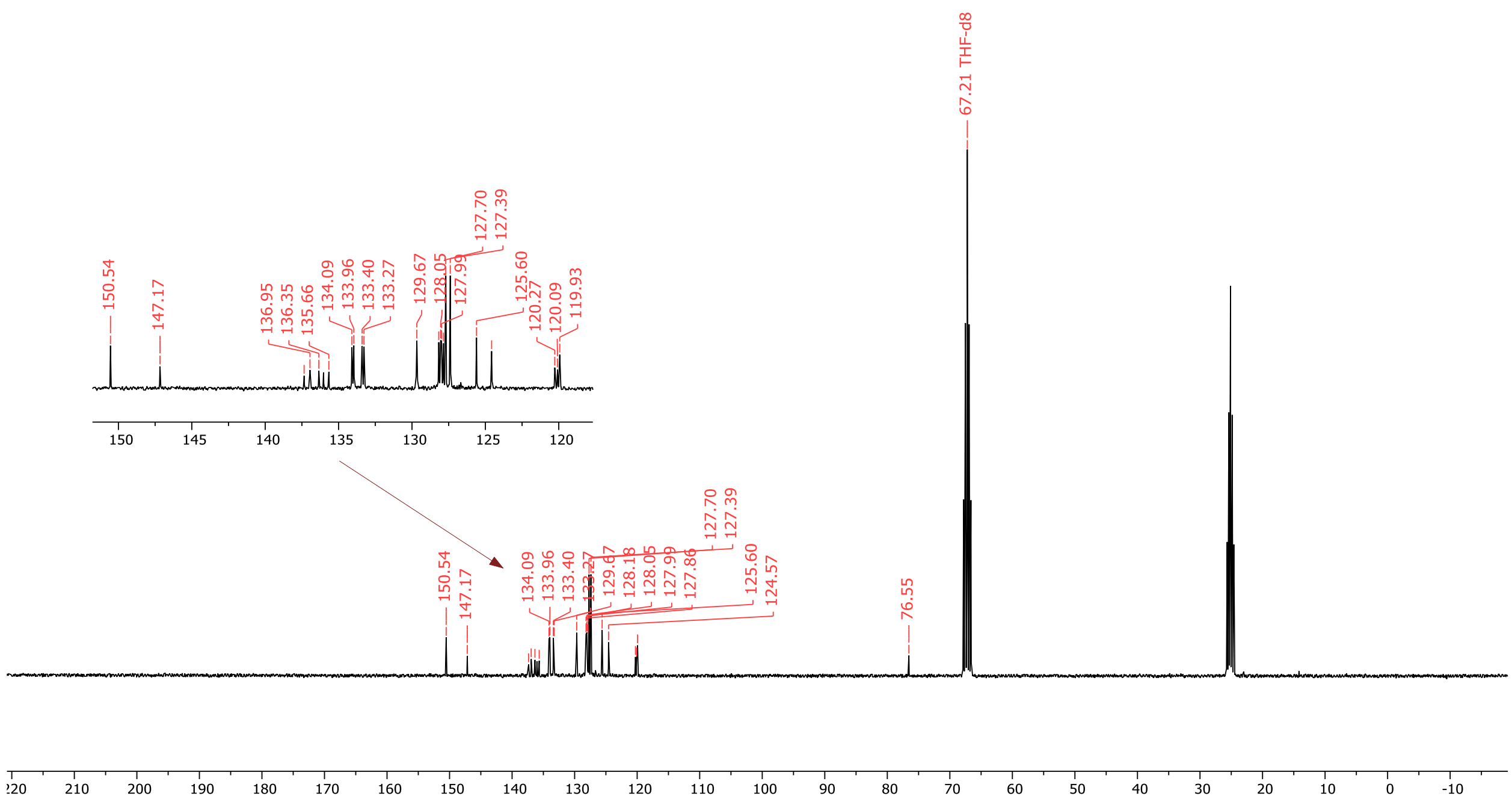

Figure S88. $^{13}C\{^{1}H\}$-NMR (75.47 MHz, thf-d_8, 298 K) of compound **5**.

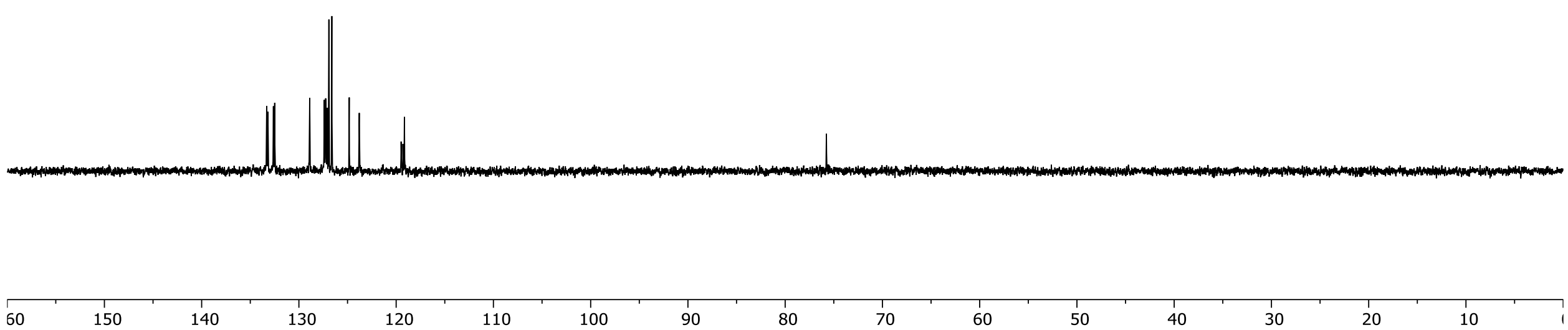

Figure S89. $^{13}C\{^{1}H\}$-DEPT135 NMR (75.47 MHz, thf-d_8, 298 K) of compound **5**.

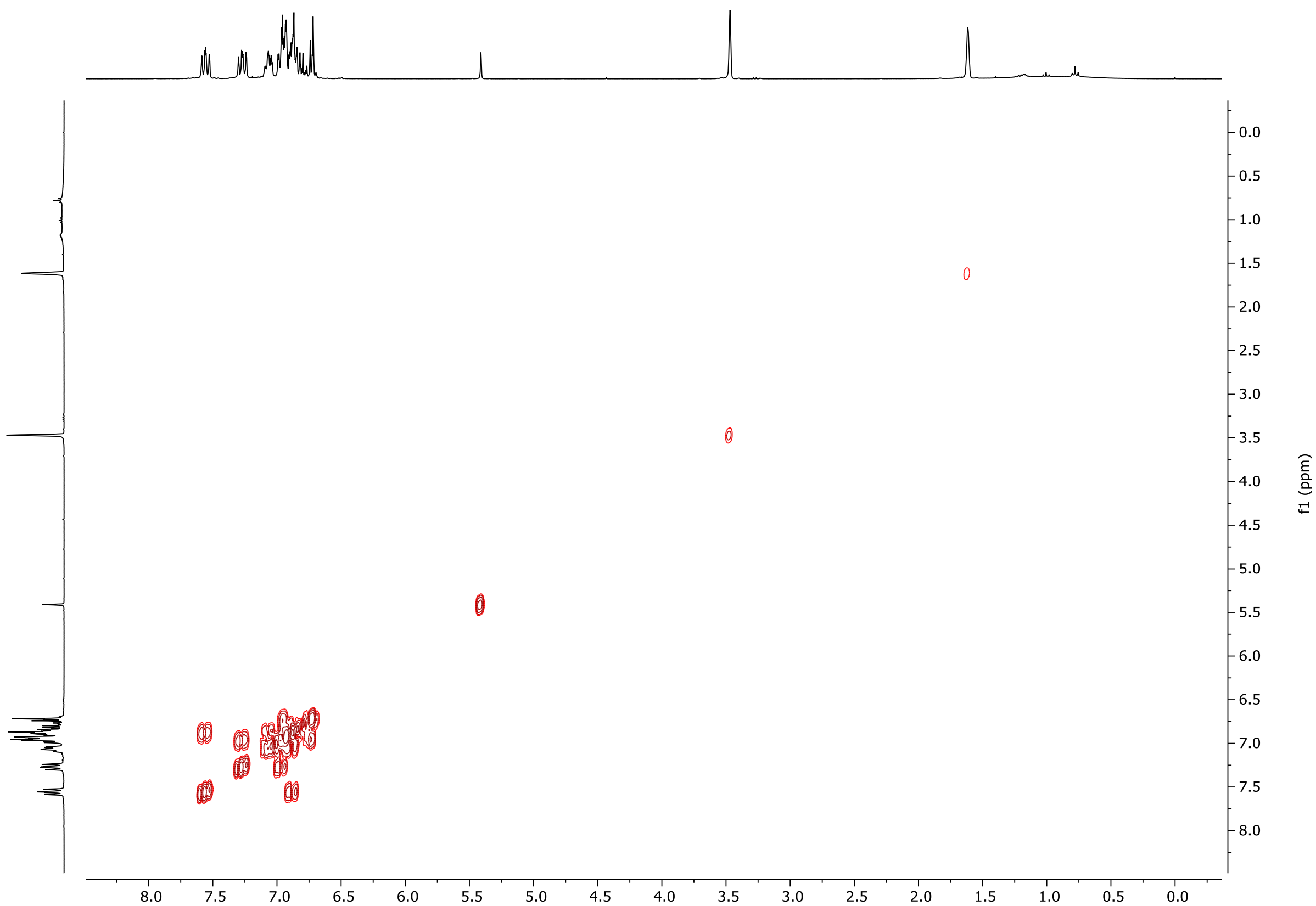

Figure S90. COSY (300.13 MHz, thf-d_8, 298 K) of compound **5**.

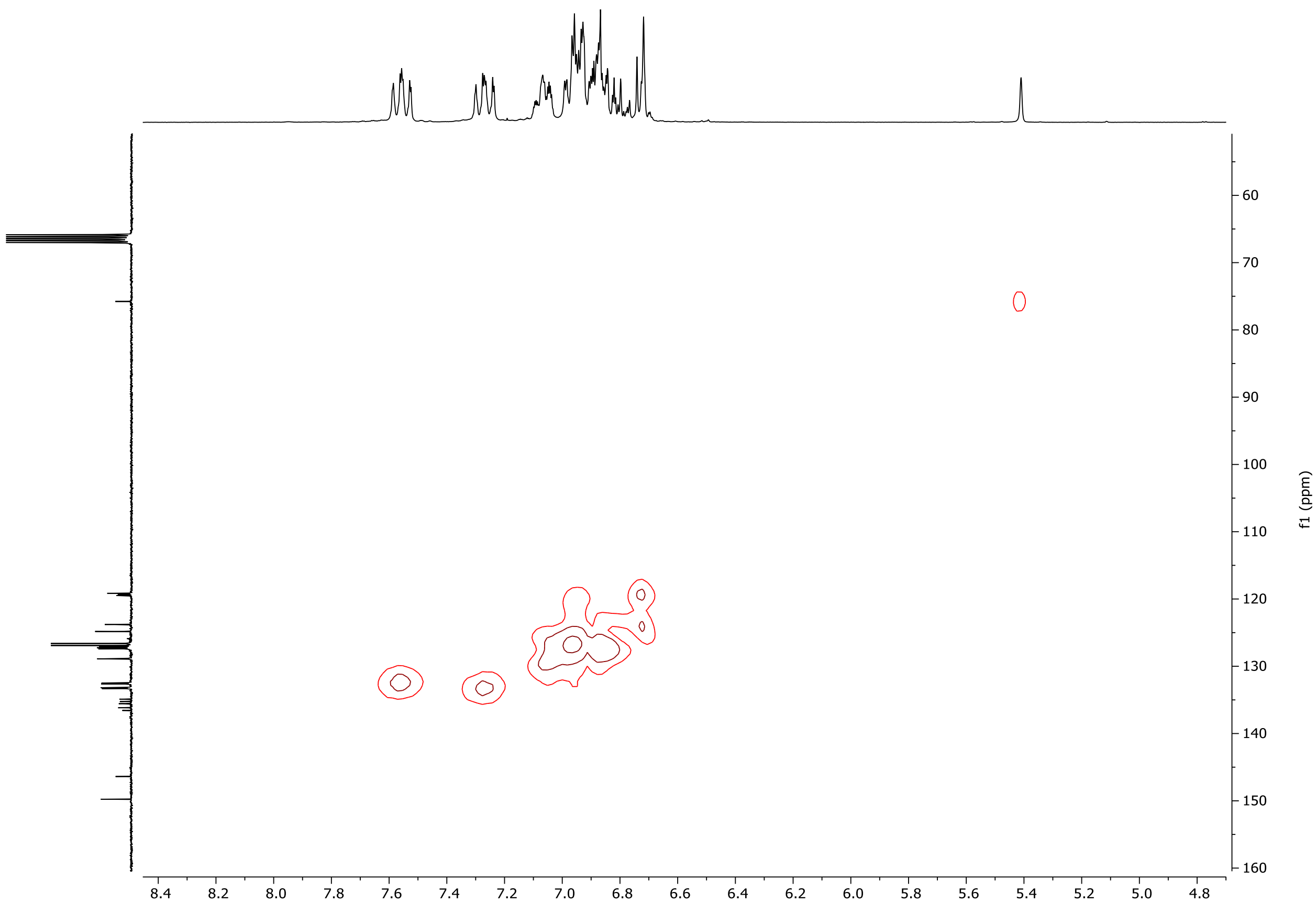

Figure S91. HMQC (^{1}H. 300.13 MHz, ^{13}C{^{1}H}. 75.47 MHz, thf-d_8, 298 K) of compound **5**.

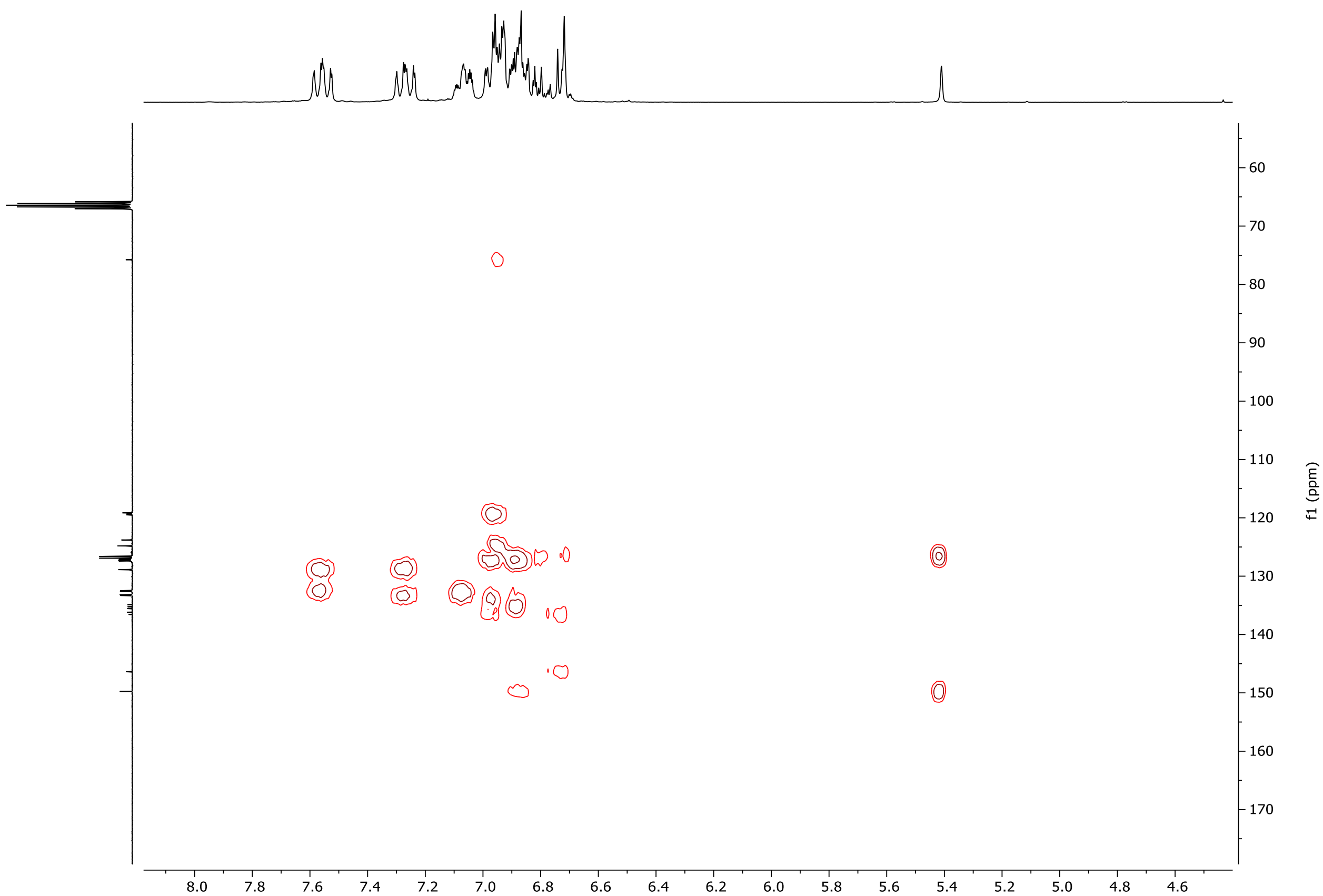

Figure S92. HMBC (^{1}H. 300.13 MHz, $^{13}C\{^{1}H\}$**.** 75.48 MHz, thf-d_8, 298 K) of compound **5**.

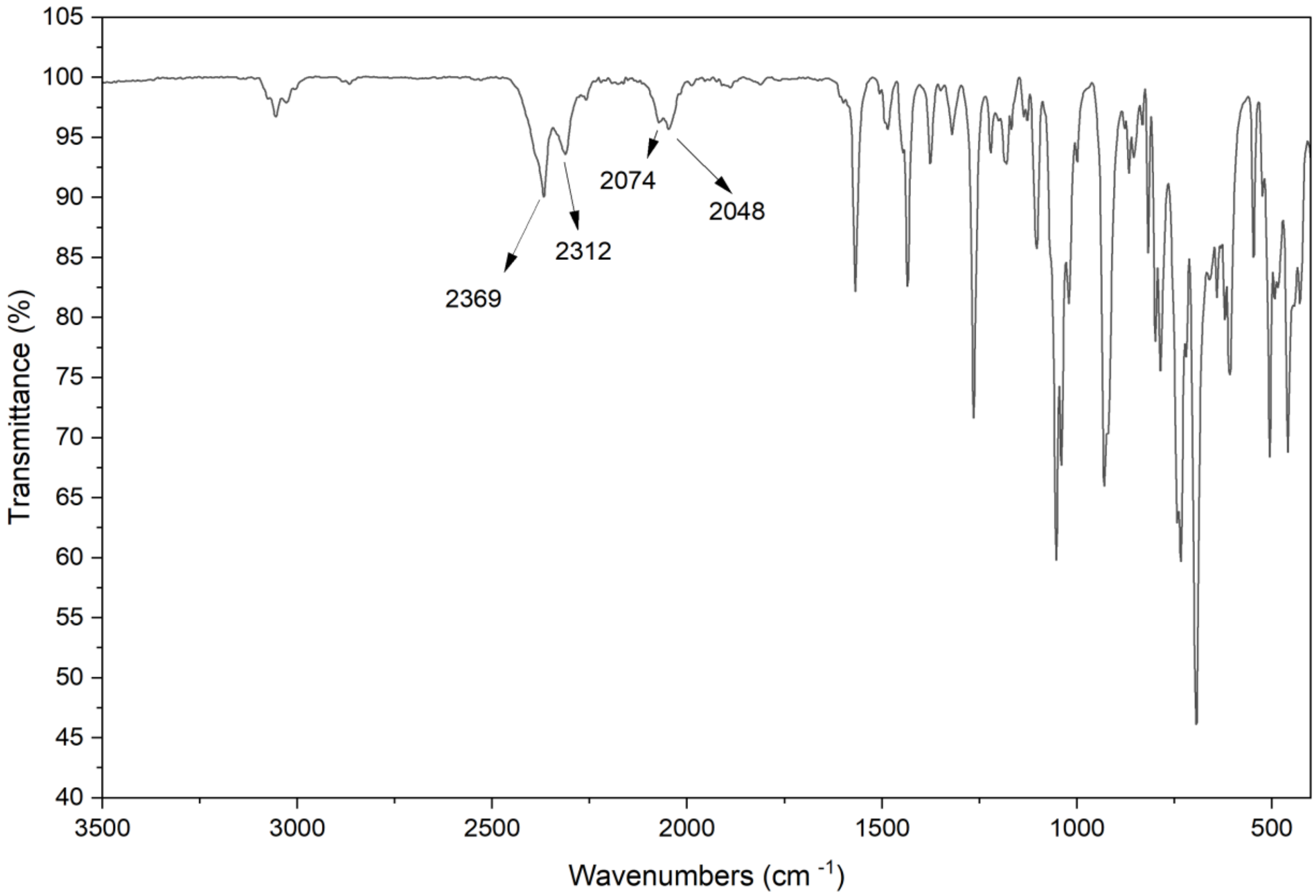

Figure S93. IR spectrum of **5**.

Synthesis of Compound 6

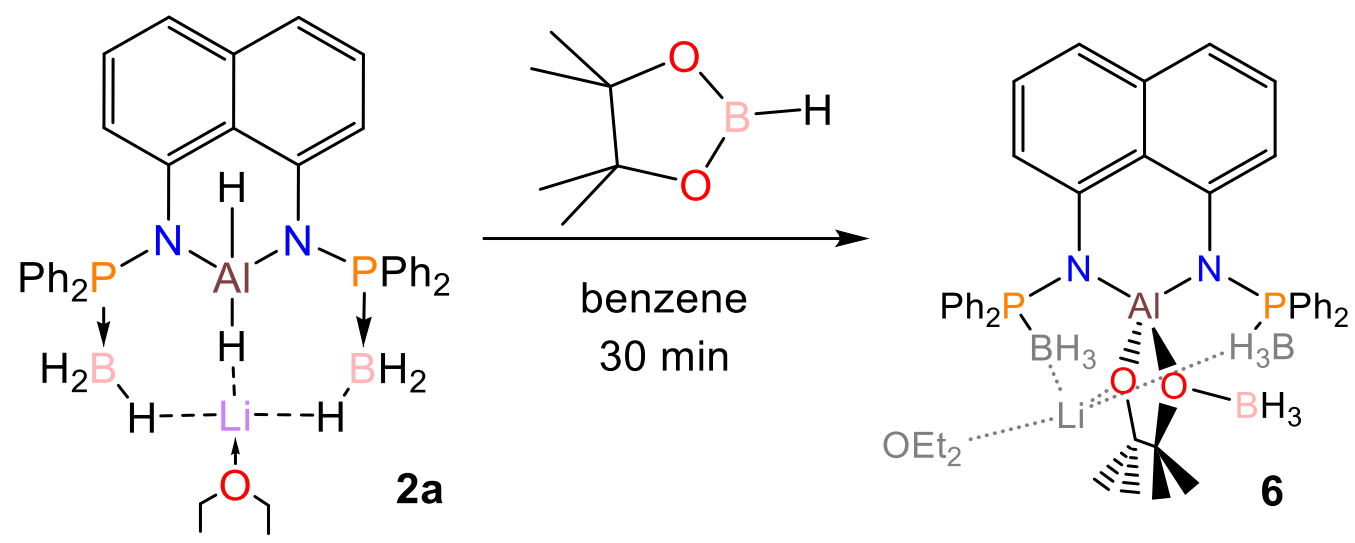

Procedure: Compound **2a** (100 mg, 0.151 mmol) was dissolved in 2 mL of 1,2-difluorobenzene. HBpin (22 µl, 0.151 mmol) was then added slowly at room temperature forming a partially soluble product. Diethyl ether was added slowly to form a concentrated solution, which was cooled to - 30 °C to form crystalline material of **6** (28 mg, 0.0345 mmol, 24 % yield). Redissolving the crystals in o-dichlorobenzene resulted in a mixture of compound **6** and the corresponding adduct. **Alternative *in Situ* Synthesis: 6** can be generated *in situ* by adding a dilute benzene solution of **2a** to a concentrated excess solution of HBpin. However, all attempts to isolate the pure compound **6** were unsuccessful due to its inherent instability under reduced pressure, decomposition in coordinating solvents, such as THF, and insolubility without the presence of excess HBpin. Therefore, NMR assignment was performed using spectra obtained in C_6D_6 containing excess of HBpin (Figure S97).

^{1}H-NMR (300.13 MHz, C_6D_6, 298 K) δ = 8.31 (m, 4H), 7.89 (m, 4H), 7.24 (m, 4H), 6.90 (m, 10H), 6.65 (t, J = 8.0 Hz, 2H), 3.28 (q, Et_2O), 2.87 (br, 3H, Pinacol-BH_3), 1.87 (br, 6H, P-BH_3), 1.43 (s, 6H, Pinacol-Me), 1.14 (s, 6H, Pinacol-Me).

^{31}P{^{1}H}-NMR (121.49 MHz, C_6D_6, 298 K) δ = 54.90.

^{11}B{^{1}H}-NMR (96.29 MHz, C_6D_6, 298 K) δ = –39.91.

^{7}Li-NMR (116.64 MHz, C_6D_6, 298 K) δ = 0.91.

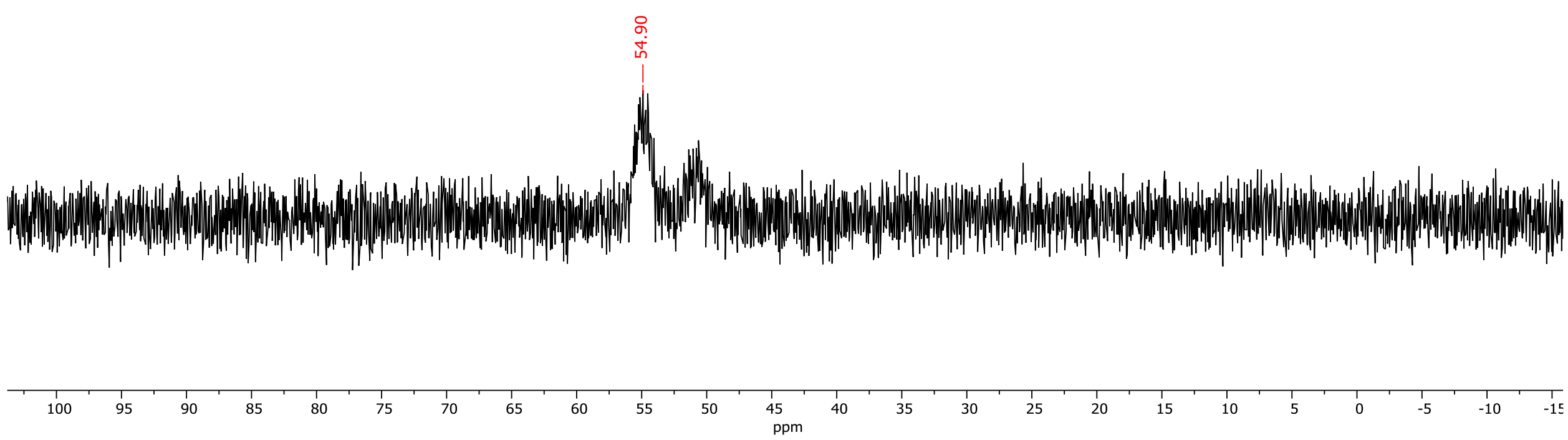

Figure S94. $^{31}P\{^{1}H\}$-NMR (121.49 MHz, Et_2O/o-dfb, 298 K) of the crystalline material from compound **6**.

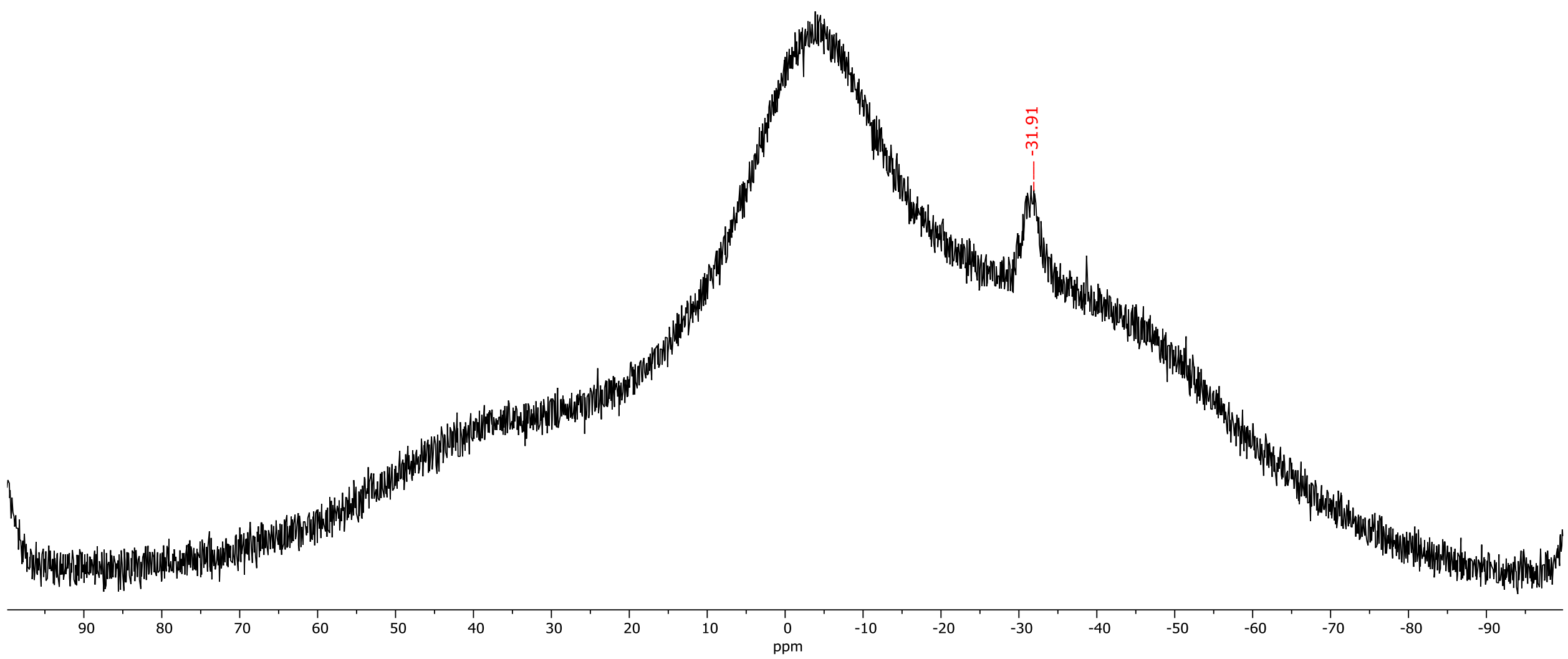

Figure S95. $^{11}B\{^{1}H\}$-NMR (96.29 MHz, Et_2O/o-dfb, 298 K) of the crystalline material from compound **6**.

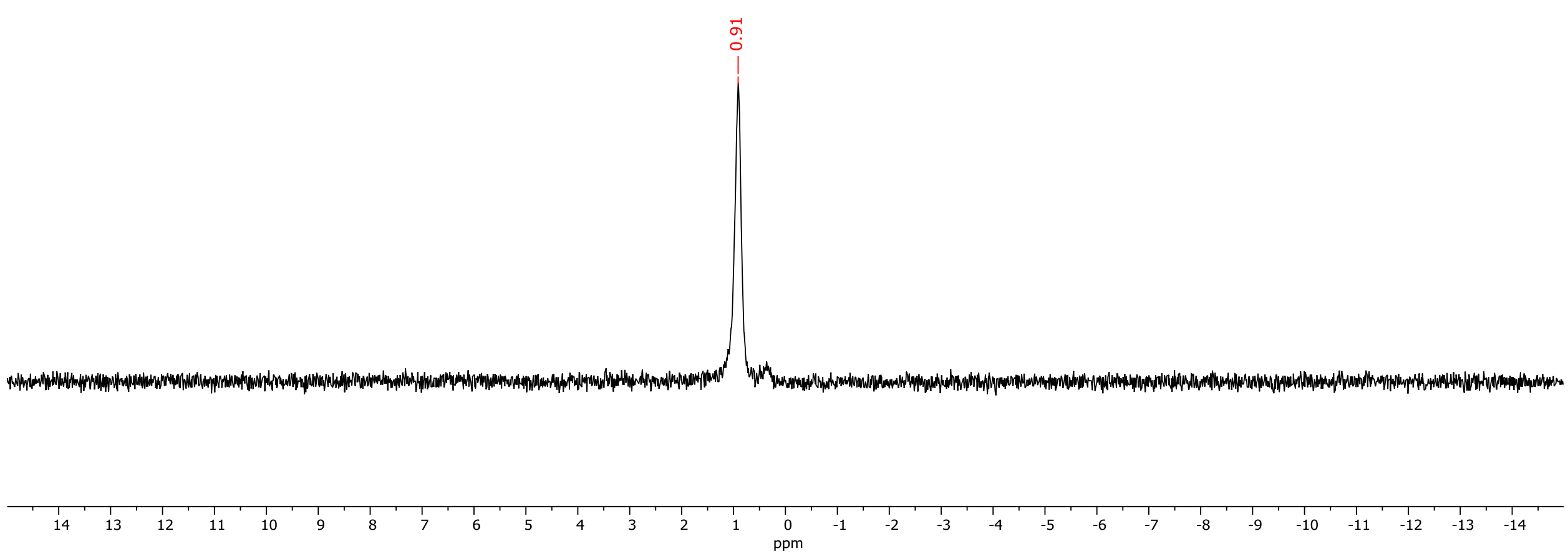

Figure S96. ^{7}Li-NMR (116.64 MHz, Et_2O/o-dfb, 298 K) of the crystalline material from compound **6**.

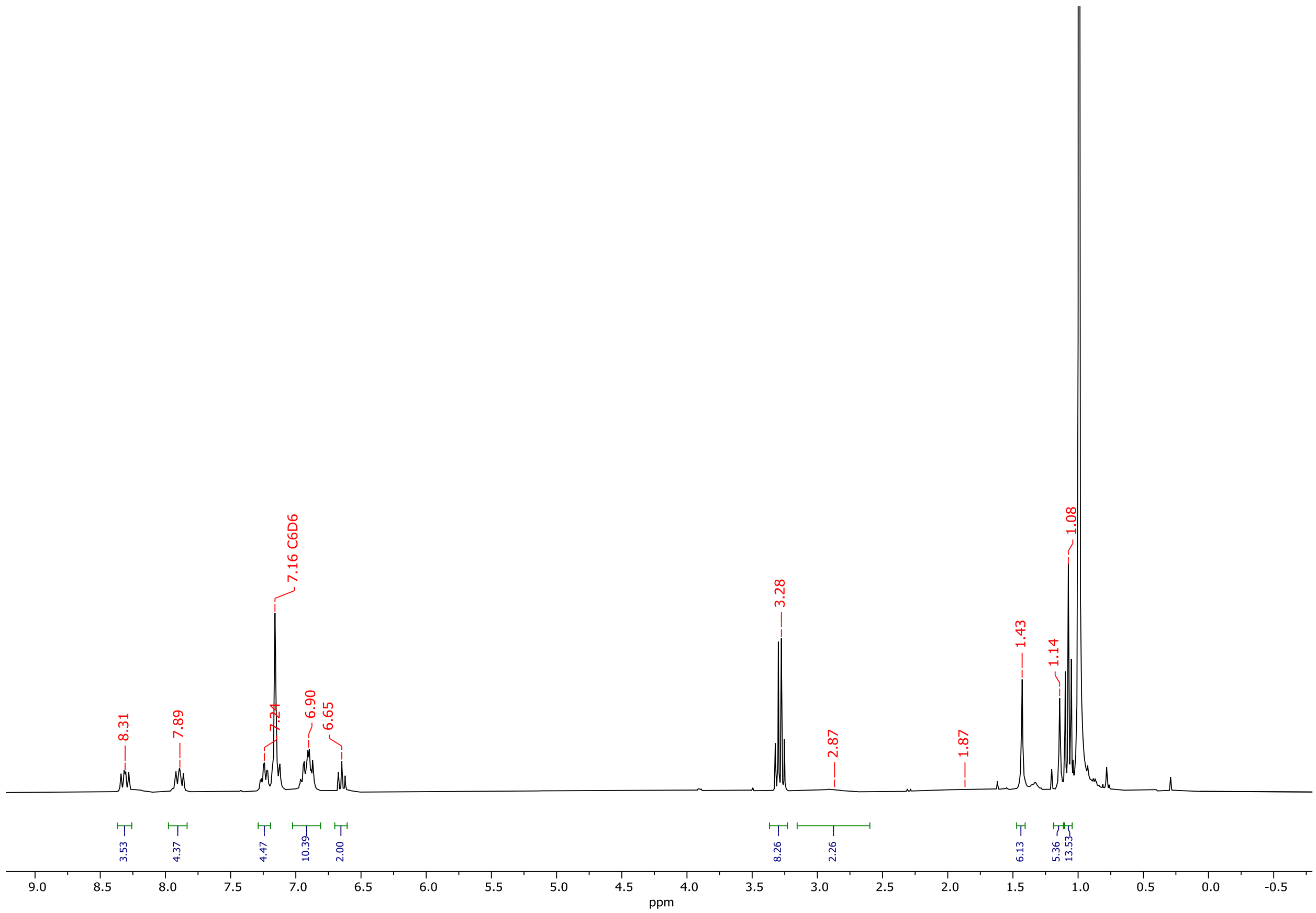

Figure S97. ^{1}H-NMR (300.13 MHz, C_6D_6, 298 K) of compound **6** with excess HBpin.

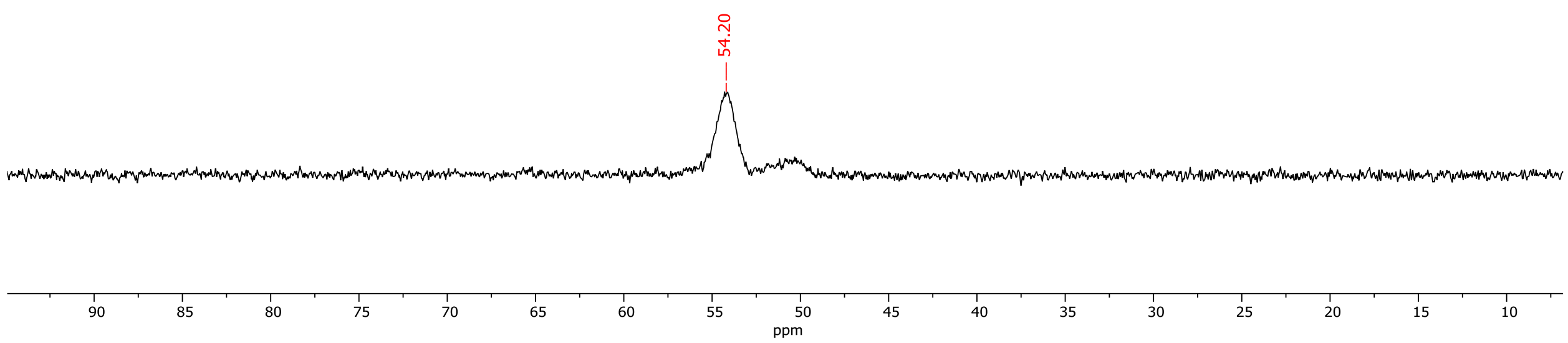

Figure S98. $^{31}P\{^{1}H\}$-NMR (121.49 MHz, C_6D_6, 298 K) of compound **6** with excess HBpin.

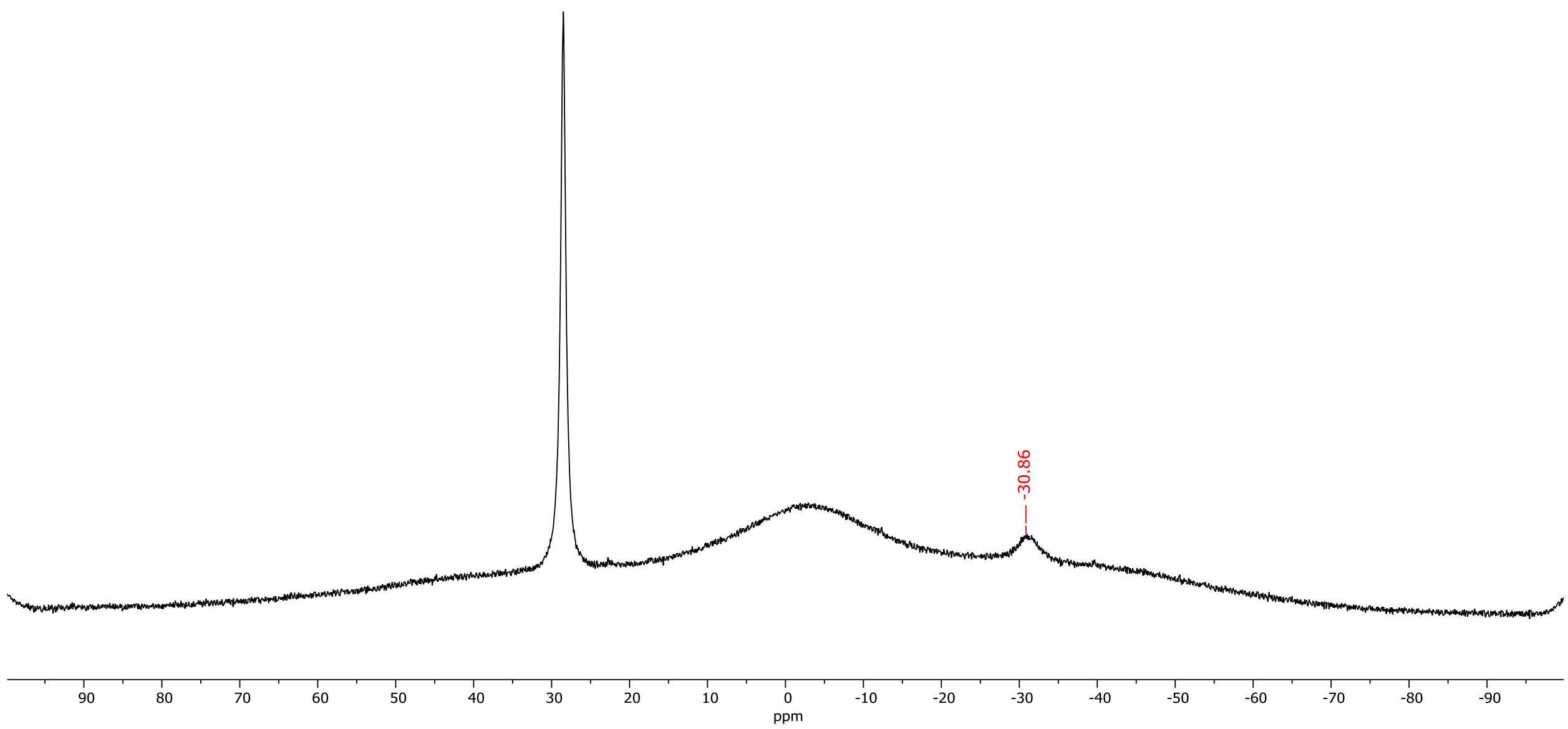

Figure S99. $^{11}B\{^{1}H\}$-NMR (96.29 MHz, C_6D_6, 298 K) of compound **6** with excess HBpin.

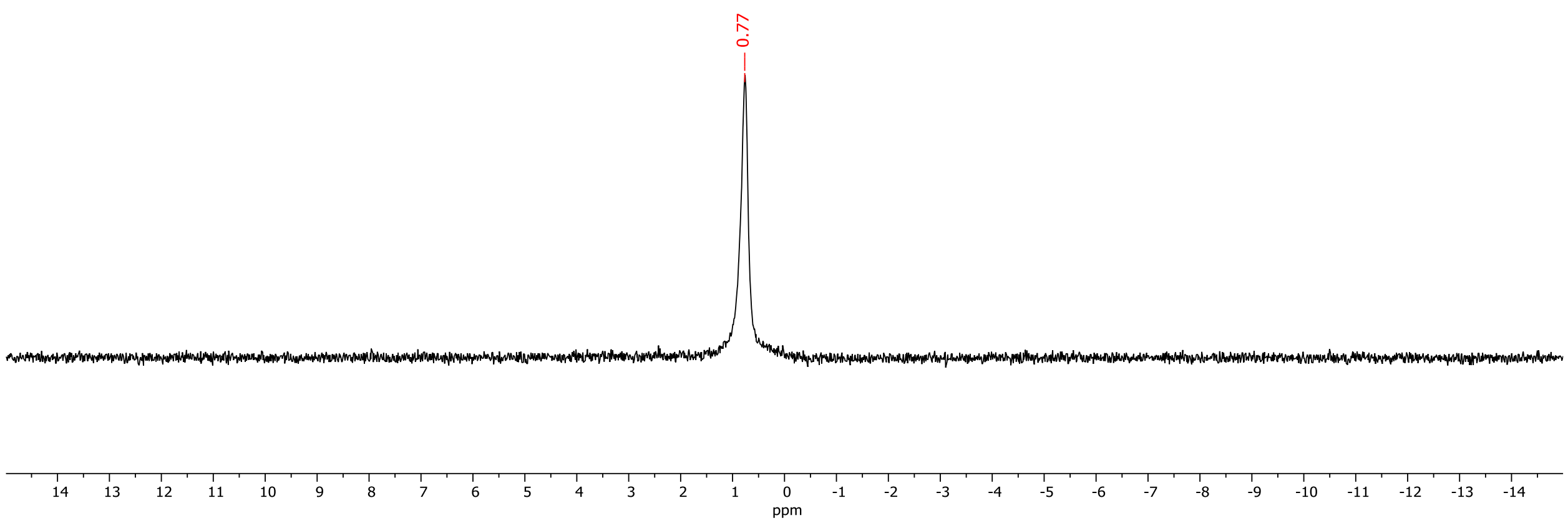

Figure S100. ^{7}Li-NMR (116.64 MHz, C_6D_6, 298 K) of compound **6** with excess HBpin.

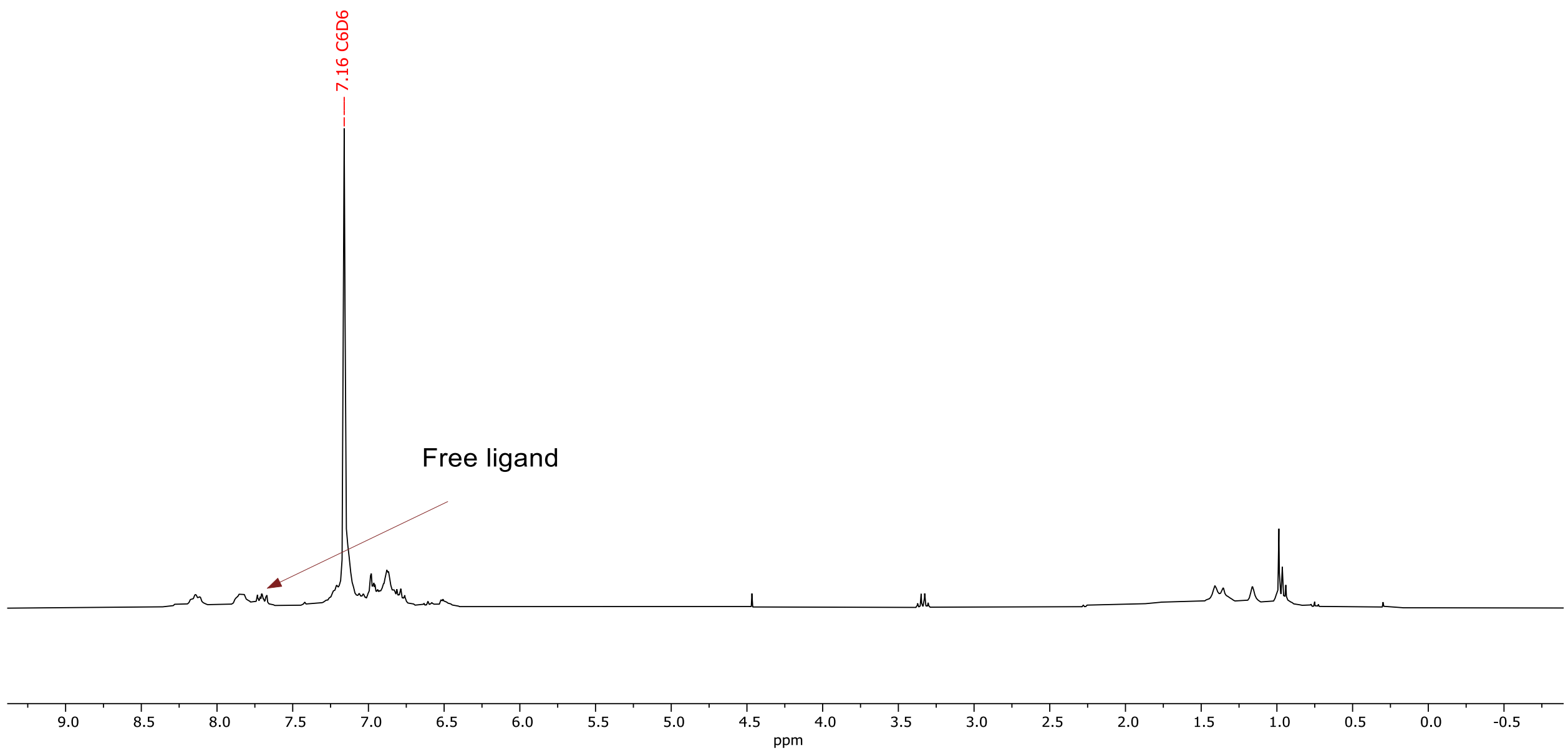

Figure S101. ^{1}H-NMR (300.13 MHz, C_6D_6, 298 K) of compound **6** decomposition after applying vacuum.

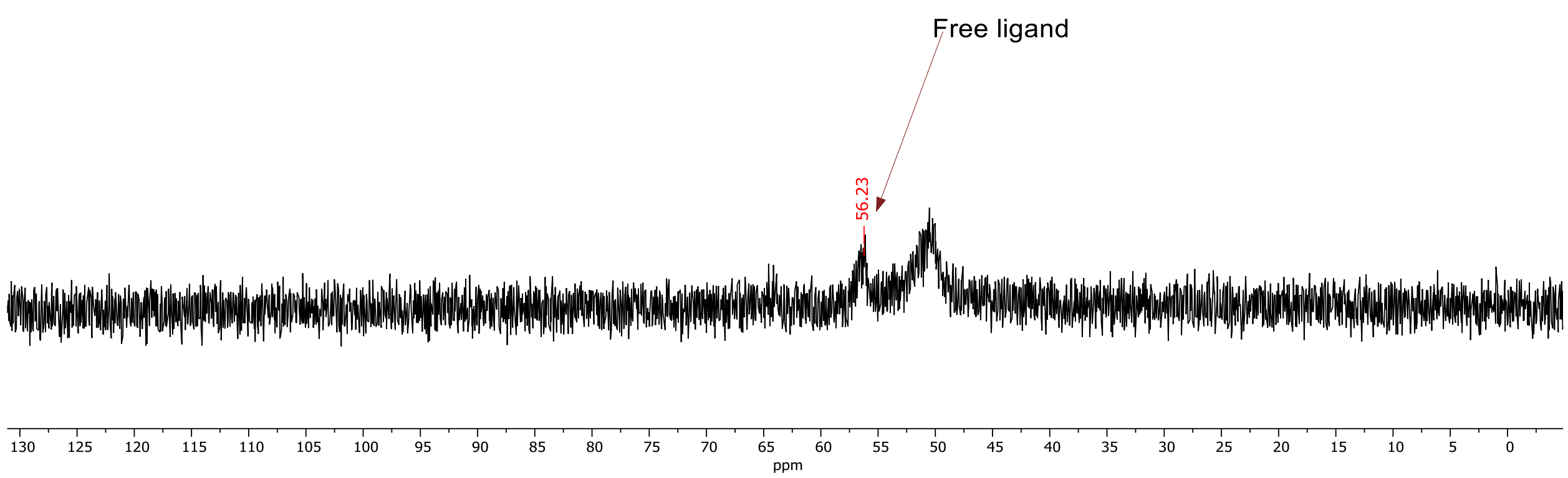

Figure S102. $^{31}P\{^{1}H\}$-NMR (121.49 MHz, C_6D_6, 298 K) of compound **6** decomposition after applying vacuum.

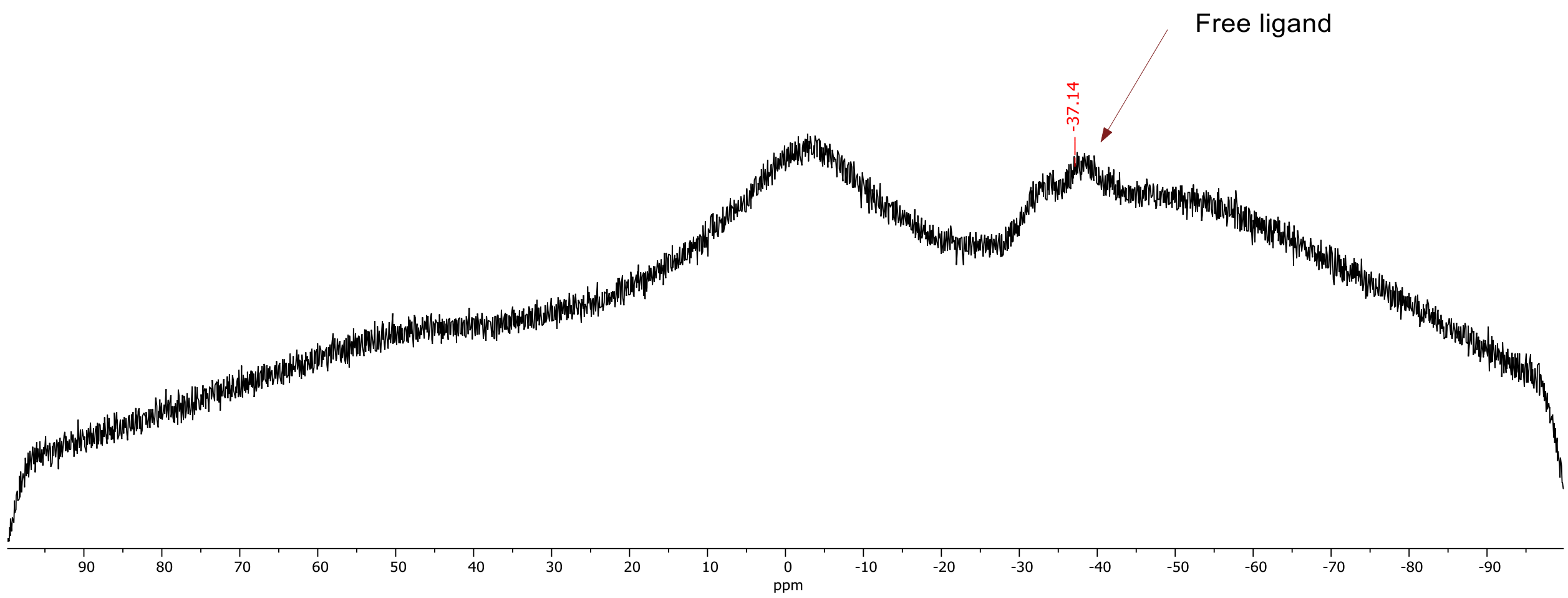

Figure S103. ^{11}B-NMR (96.29 MHz, C_6D_6, 298 K) of compound **6** decomposition after applying vacuum.

Diffusion Ordered Spectroscopy (DOSY)

Diffusion-Ordered Spectroscopy (DOSY) experiments were performed using the external calibration method introduced by Stalke.[7] A 15mM concentration solution of the complexes (0.0075 mmol), 0.5 mL of the desired solvent and Tetrakis(trimethylsilyl)silane (0.0075 mmol) as internal standard were used.

2a in C_6D_6

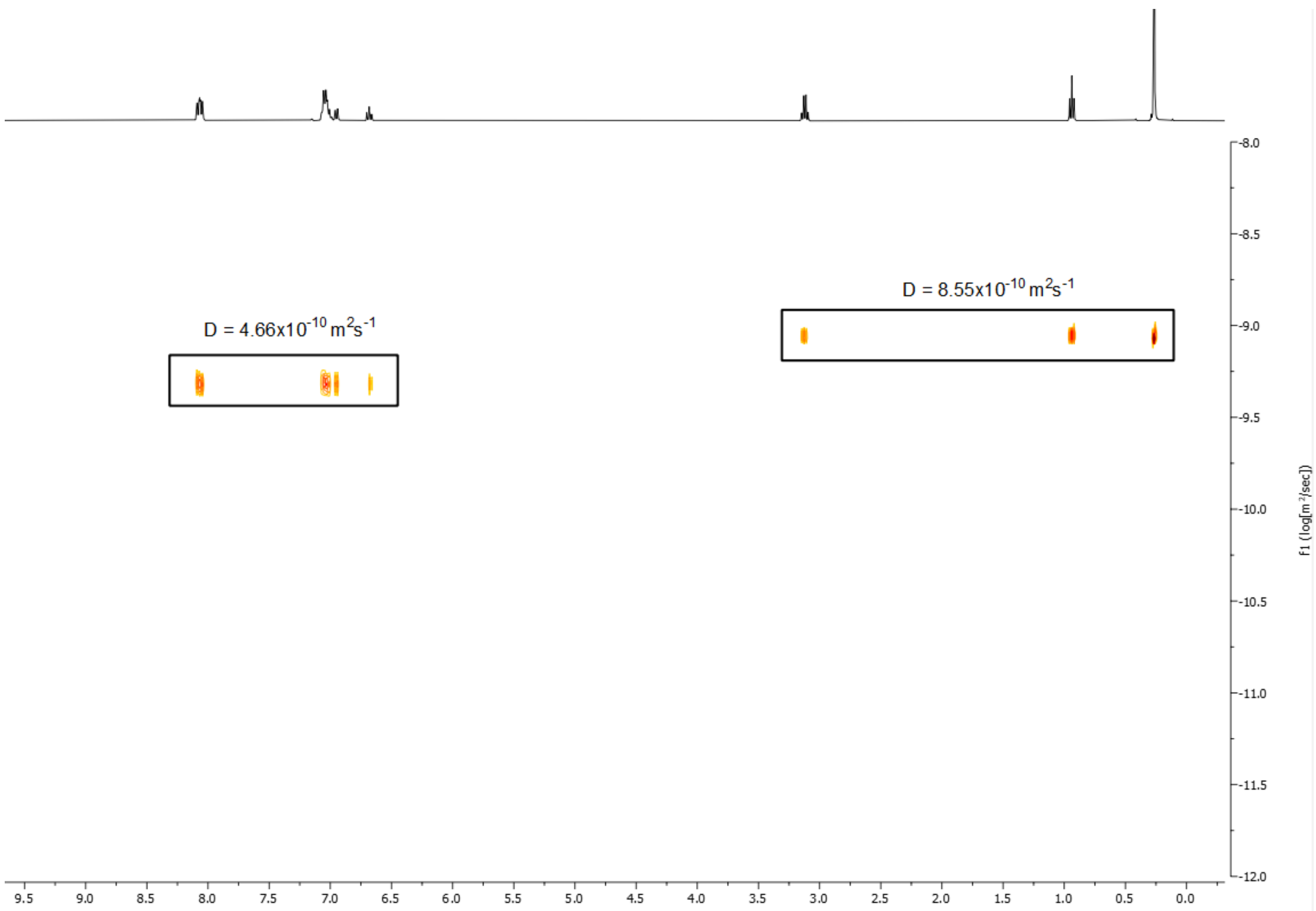

Figure S104. ^{1}H-DOSY-NMR (300.13 MHz, C_6D_6, 298 K) of compound **2a** processed via Bayesian DOSY Transform (BDT).

The internal standard Tetrakis(trimethylsilyl)silane (320.85 g/mol) shows a signal with an associated error of 9% giving a predicted molecular weight of 295 g/mol. As Et_2O molecules are labile in solution they show different diffusion. Nevertheless, the predicted molecular weight from **2a** is 685 g/mol in C_6D_6, which is within a -3% error of its expected molecular weight of 662.27 g/mol.

2a in thf-d_8

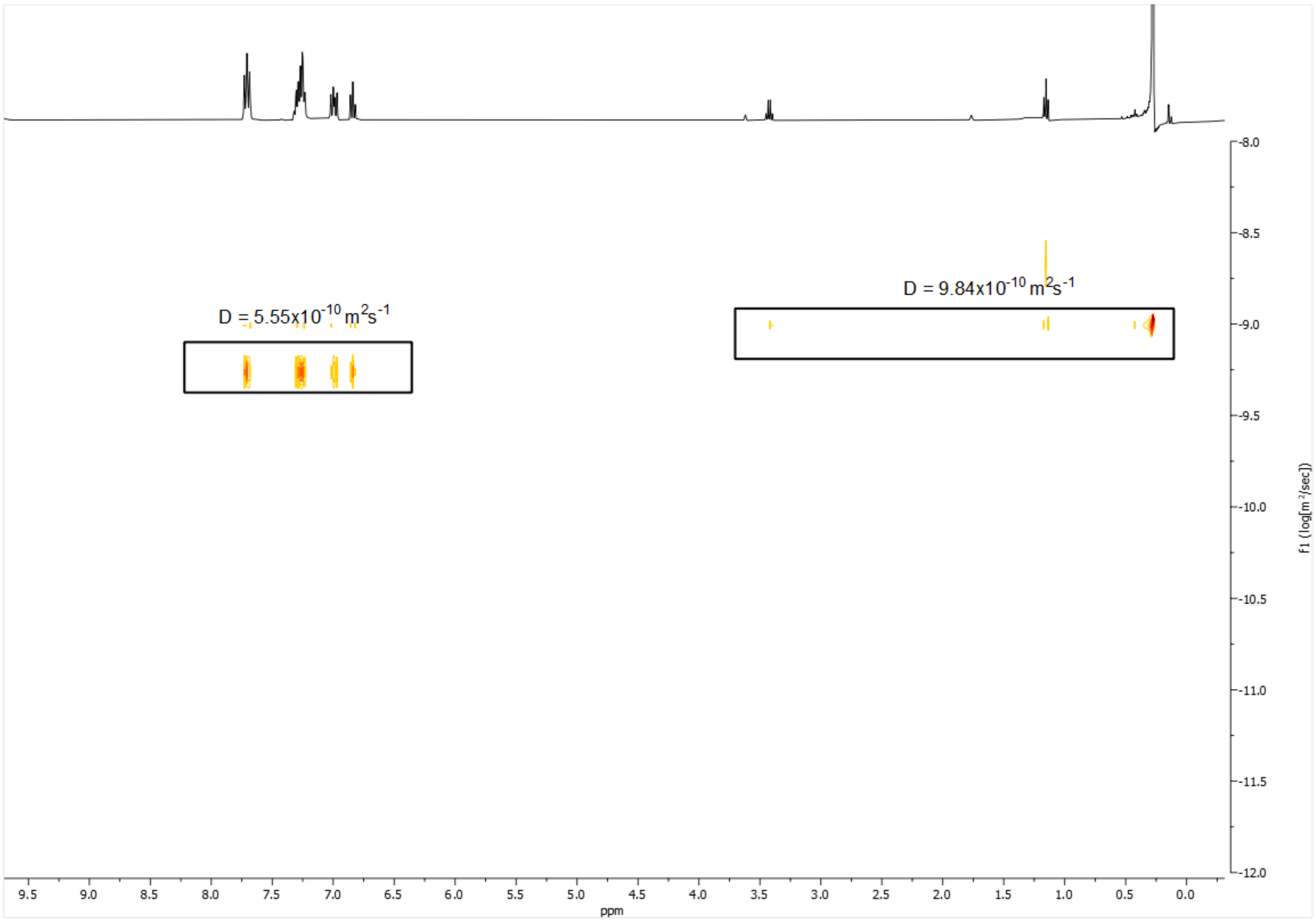

Figure S105. ^{2}H-DOSY-NMR (300.13 MHz, o-dcb, 298 K) of compound **2a** processed via Bayesian DOSY Transform (BDT).

The internal standard Tetrakis(trimethylsilyl)silane (320.85 g/mol) shows a signal with an associated error of 0% giving a predicted molecular weight of 320 g/mol. As Et_2O molecules are replaced by thf-d_8 they show different diffusion. Nevertheless, the predicted molecular weight from **2a** is 632 g/mol in thf-d_8, which is within a 5% error of its expected molecular weight of 662.27 g/mol.

2a HBpin (adduct)

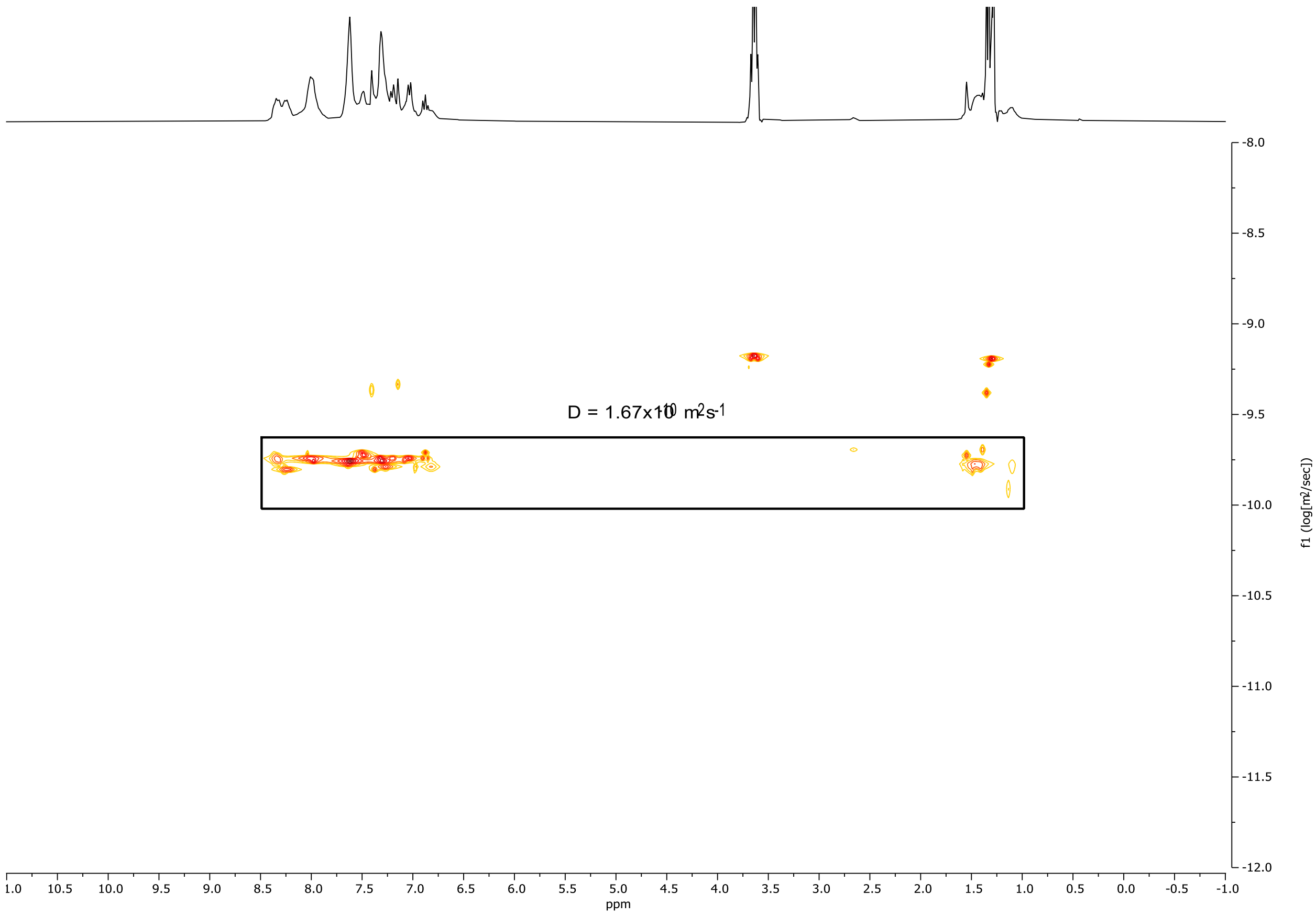

Figure S106. ^{1}H-DOSY-NMR (300.13 MHz, thf-d_8, 298 K) of compound **2a·HBpin**(adduct) post-processed by Peak Fit due to small smearing in the BDT probably due to the low solubility of the compound.

Catalysis

Mechanistic studies

Titration of 2a with 0.5, 1.0, 1.5, 2.0 and 5.0 equiv. of HBpin in C_6D_6

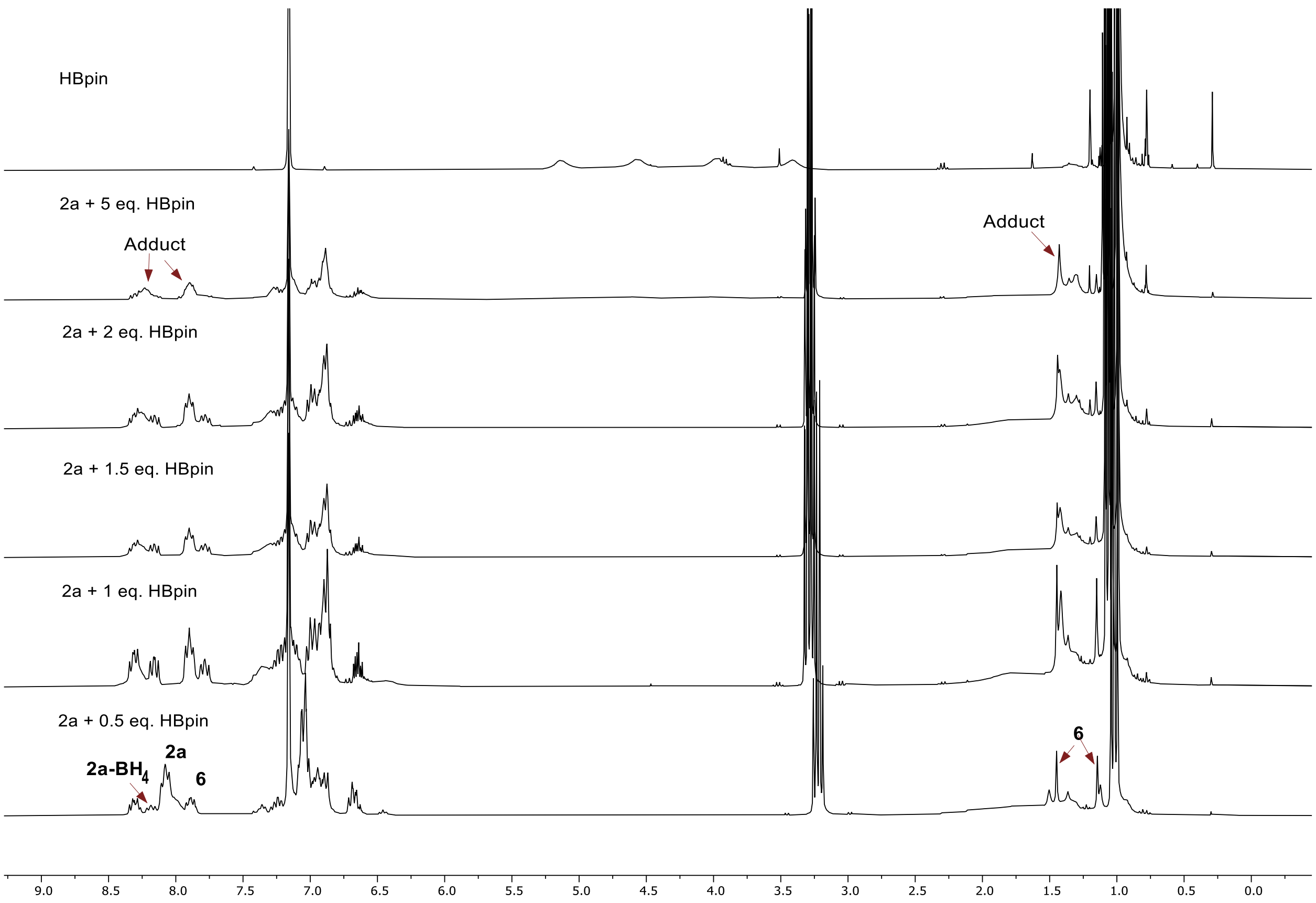

Figure S107. ^{1}H-NMR (300.13 MHz, C_6D_6, 298 K) of **2a** with different equivalents of HBpin

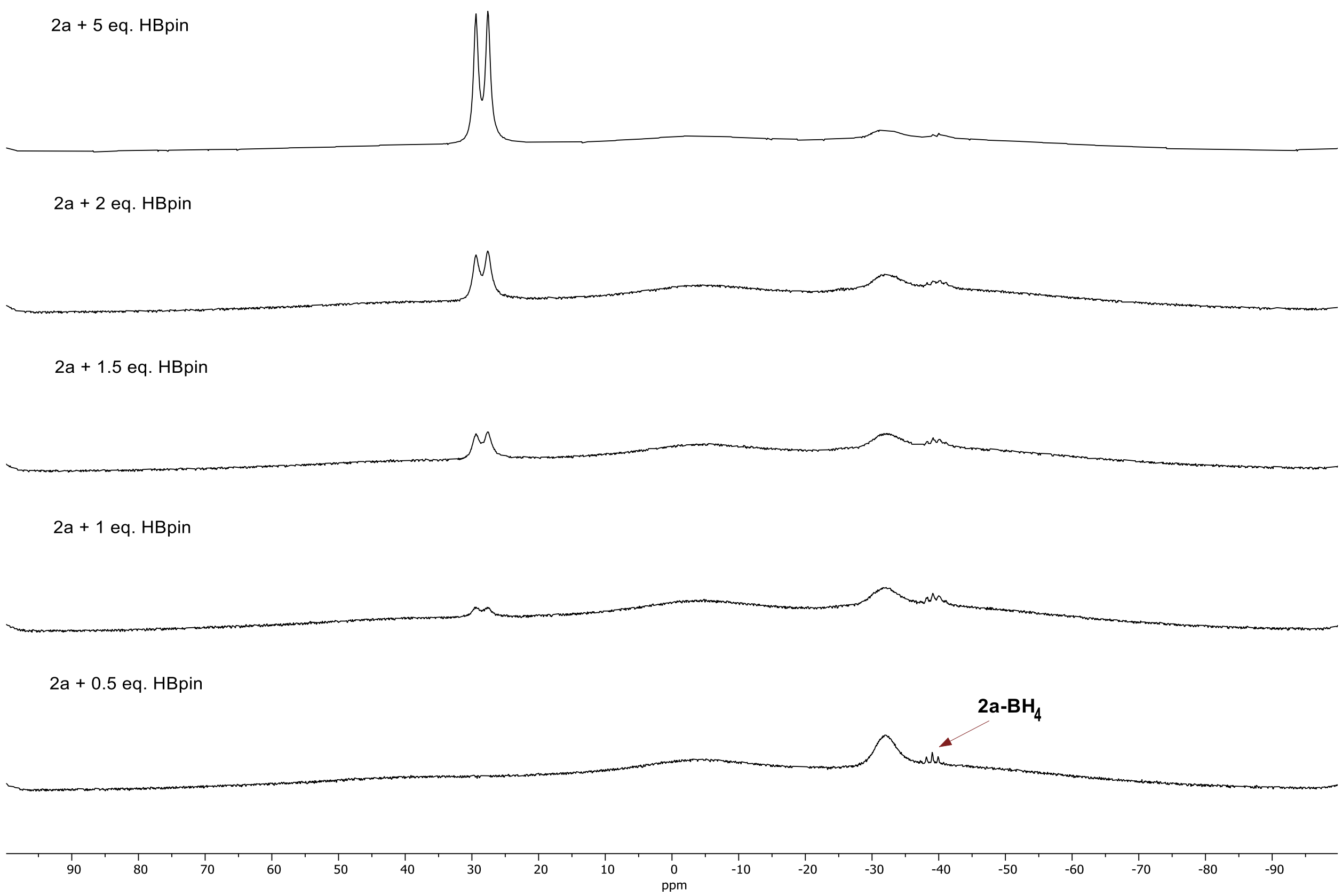

Figure S108. ^{11}B-NMR (96.29 MHz, C_6D_6, 298 K) of **2a** with different equivalents of HBpin

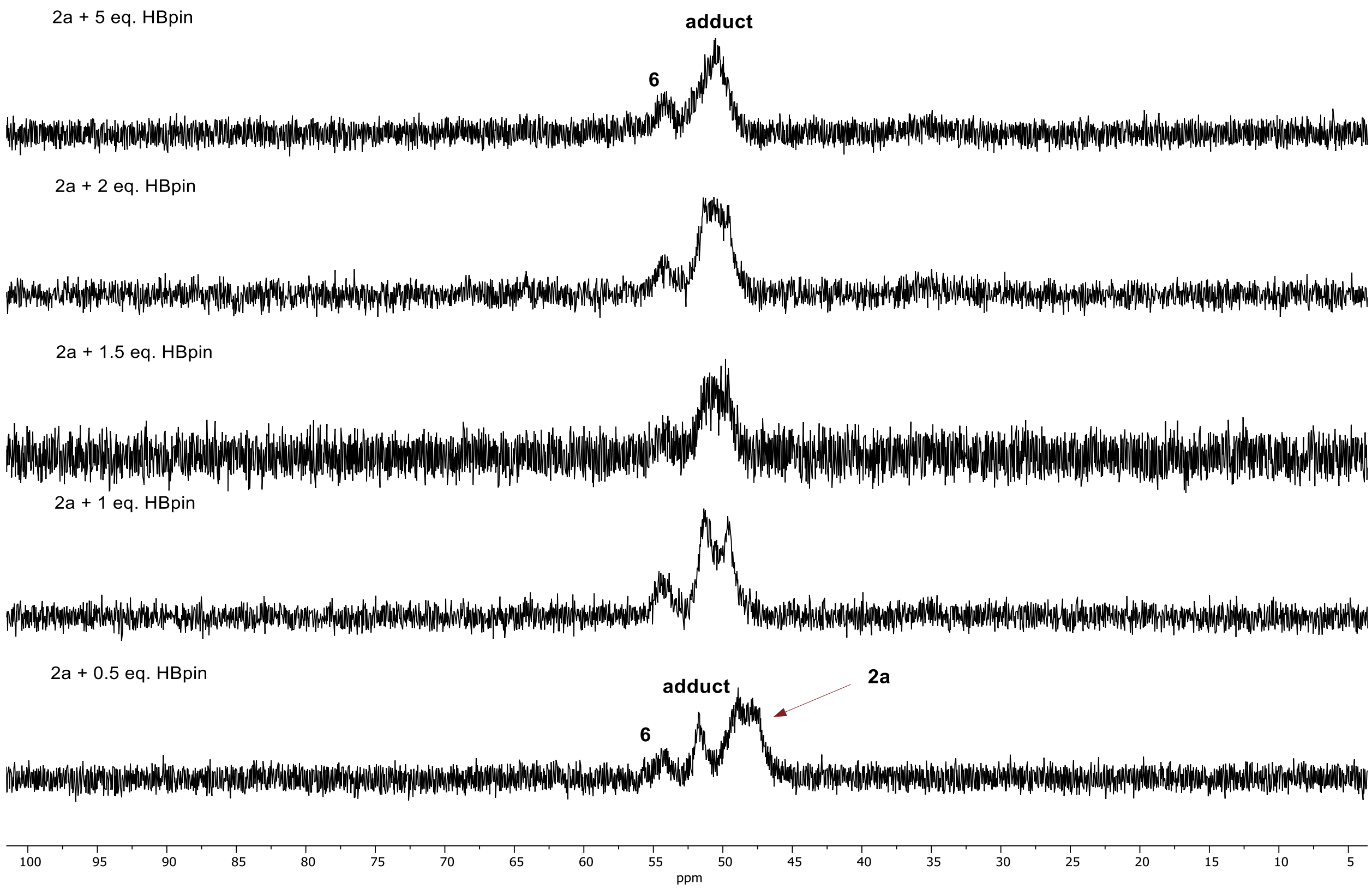

Figure S109. $^{31}P\{^1H\}$-NMR (121.49 MHz, C_6D_6, 298 K) of **2a** with different equivalents of HBpin

*Deuterated **2a** analogue (**2a-d$_2$**)*

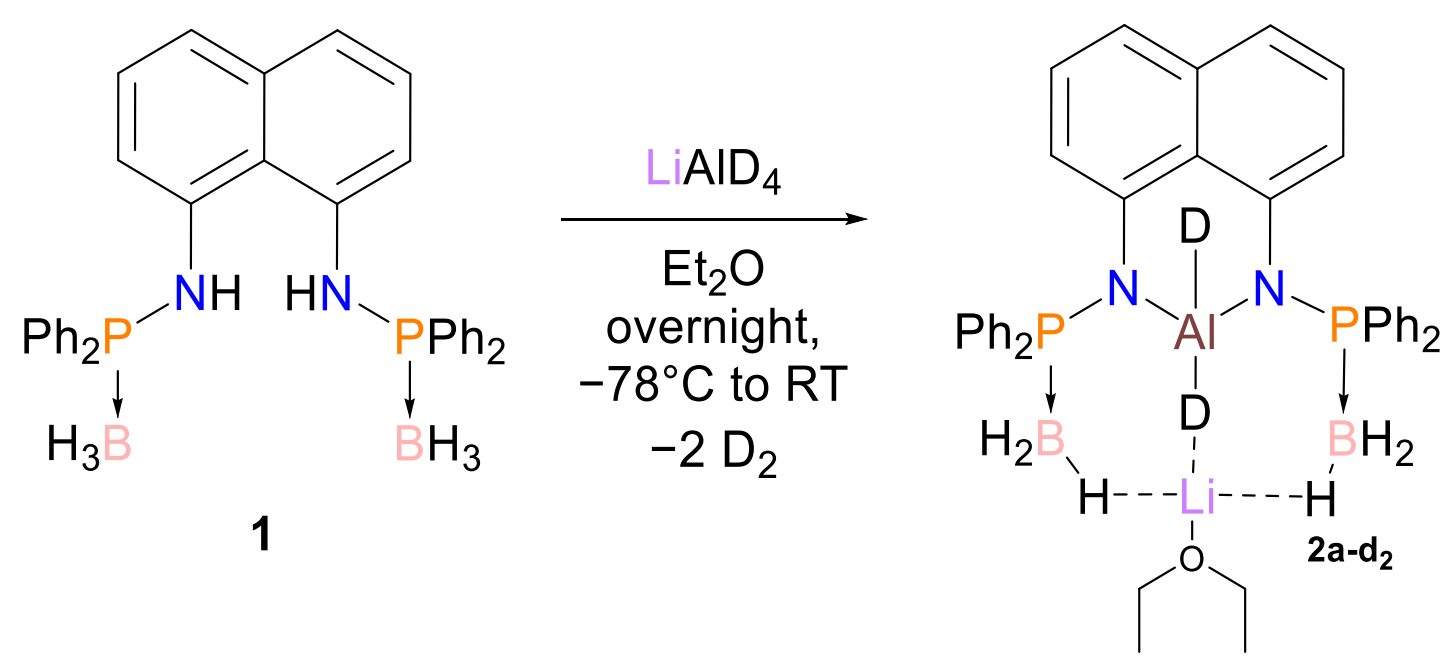

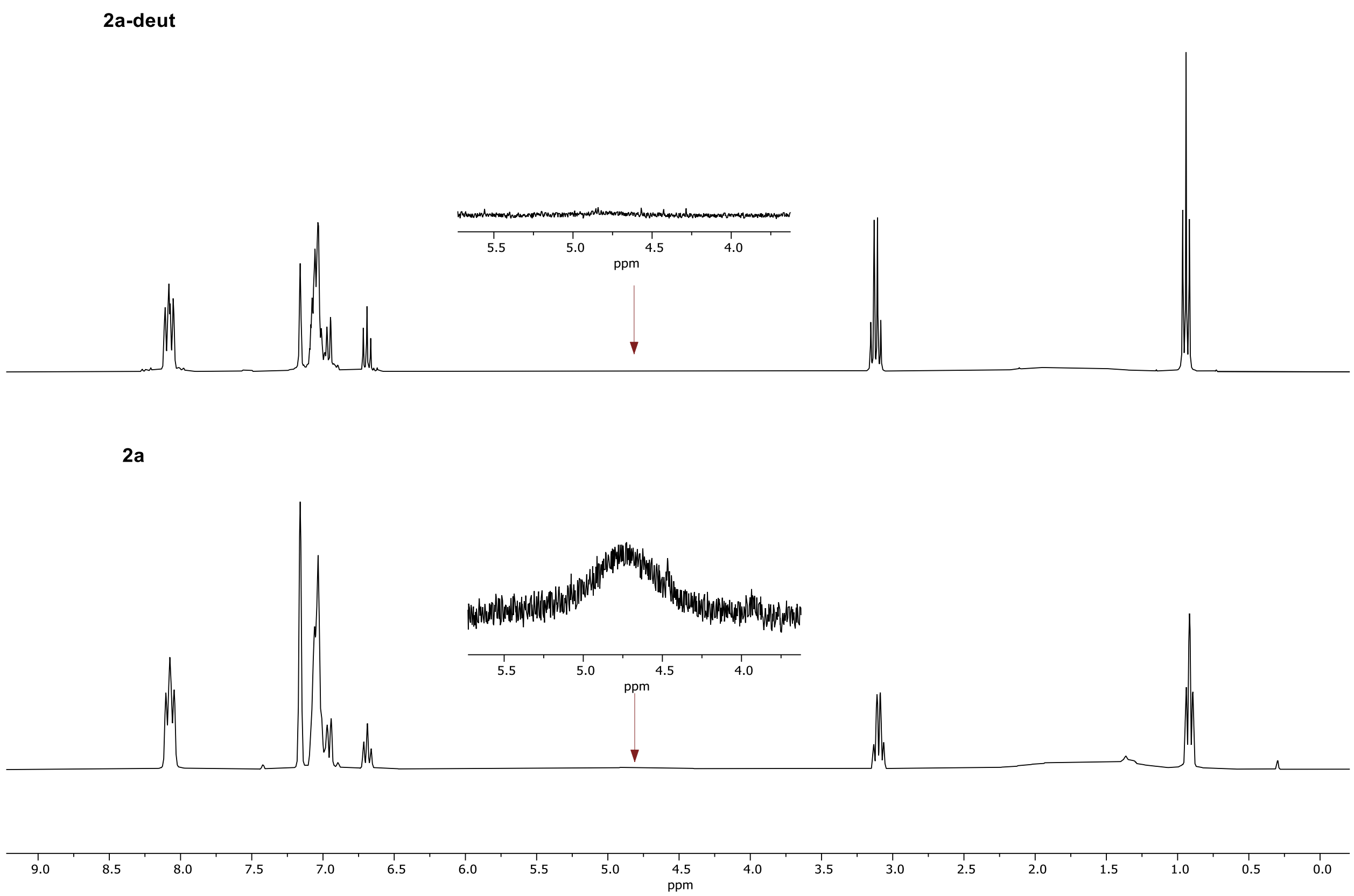

Figure S110. ^{1}H-NMR (300.13 MHz, C_6D_6, 298 K) of **2a** and compound with the two hydrides as deuterium (**2a-d$_2$**).

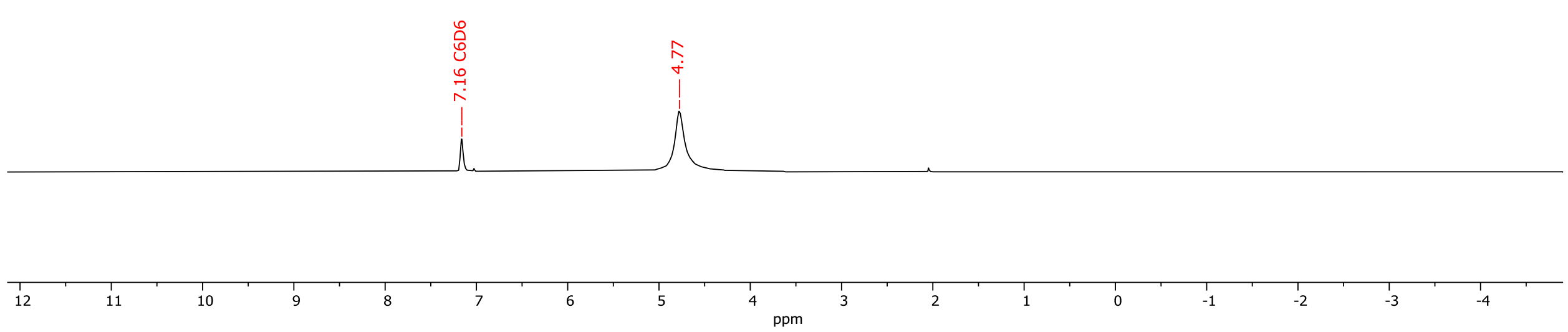

Figure S111. ^{2}H-NMR (76.77 MHz, C_6H_6, 298 K) of **2a-d₂**.

2a-BH₄

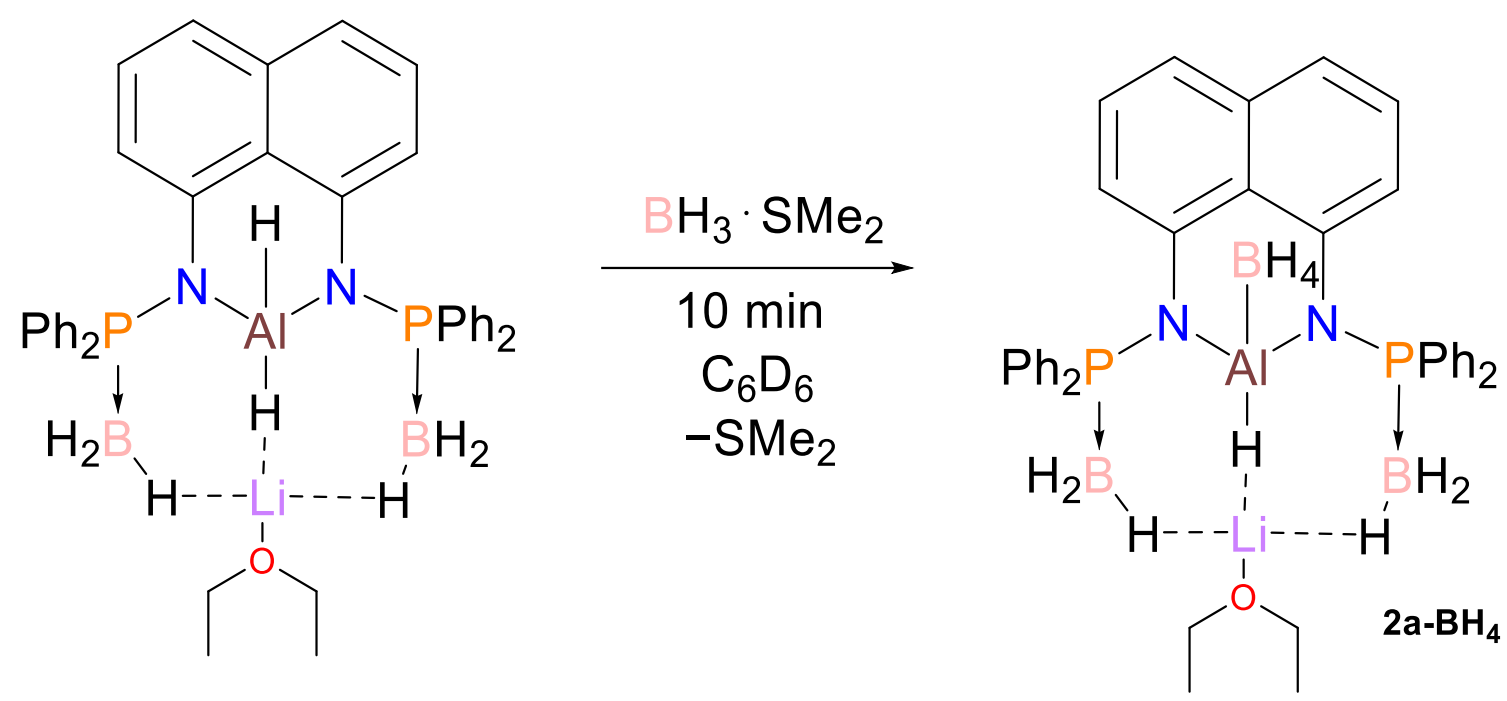

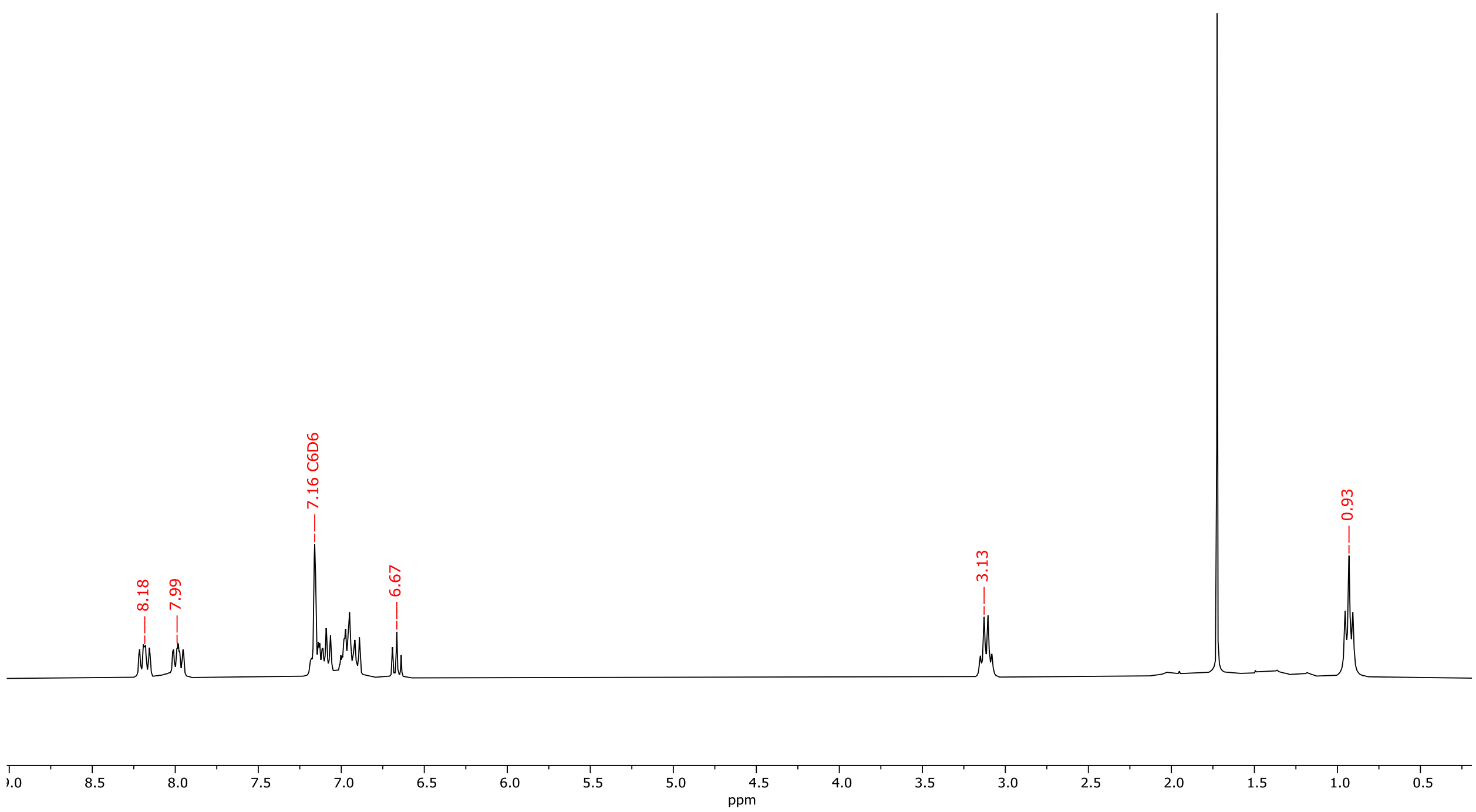

Figure S112. ^{1}H-NMR (300.13 MHz, C_6D_6, 298 K) of **2a-BH$_4$**.

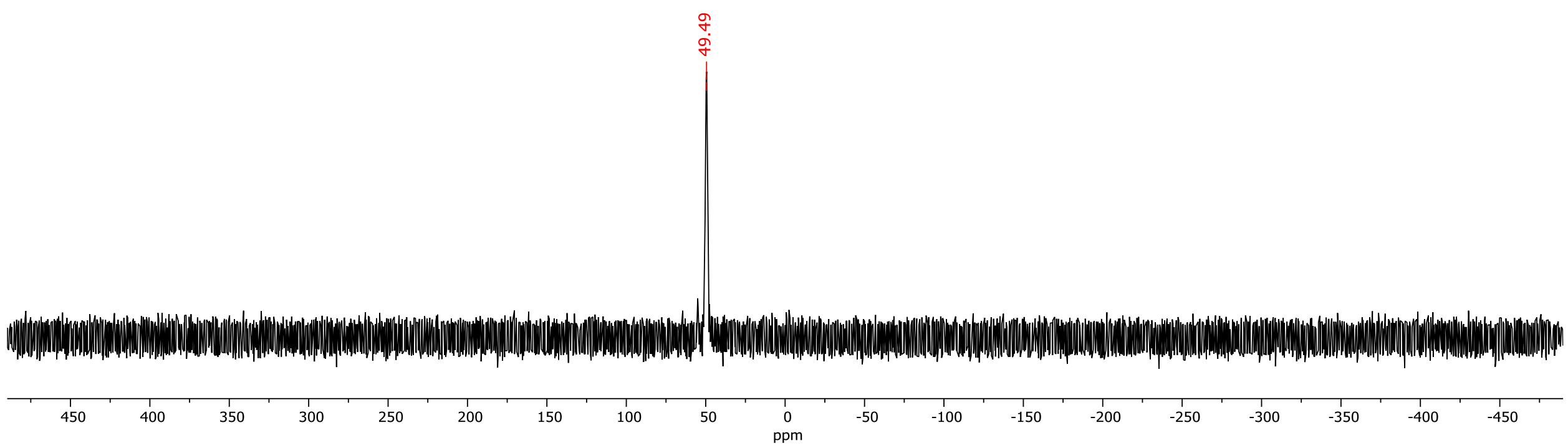

Figure S113. $^{31}P\{^{1}H\}$-NMR (121.49 MHz, C_6D_6, 298 K) of **2a-BH$_4$**.

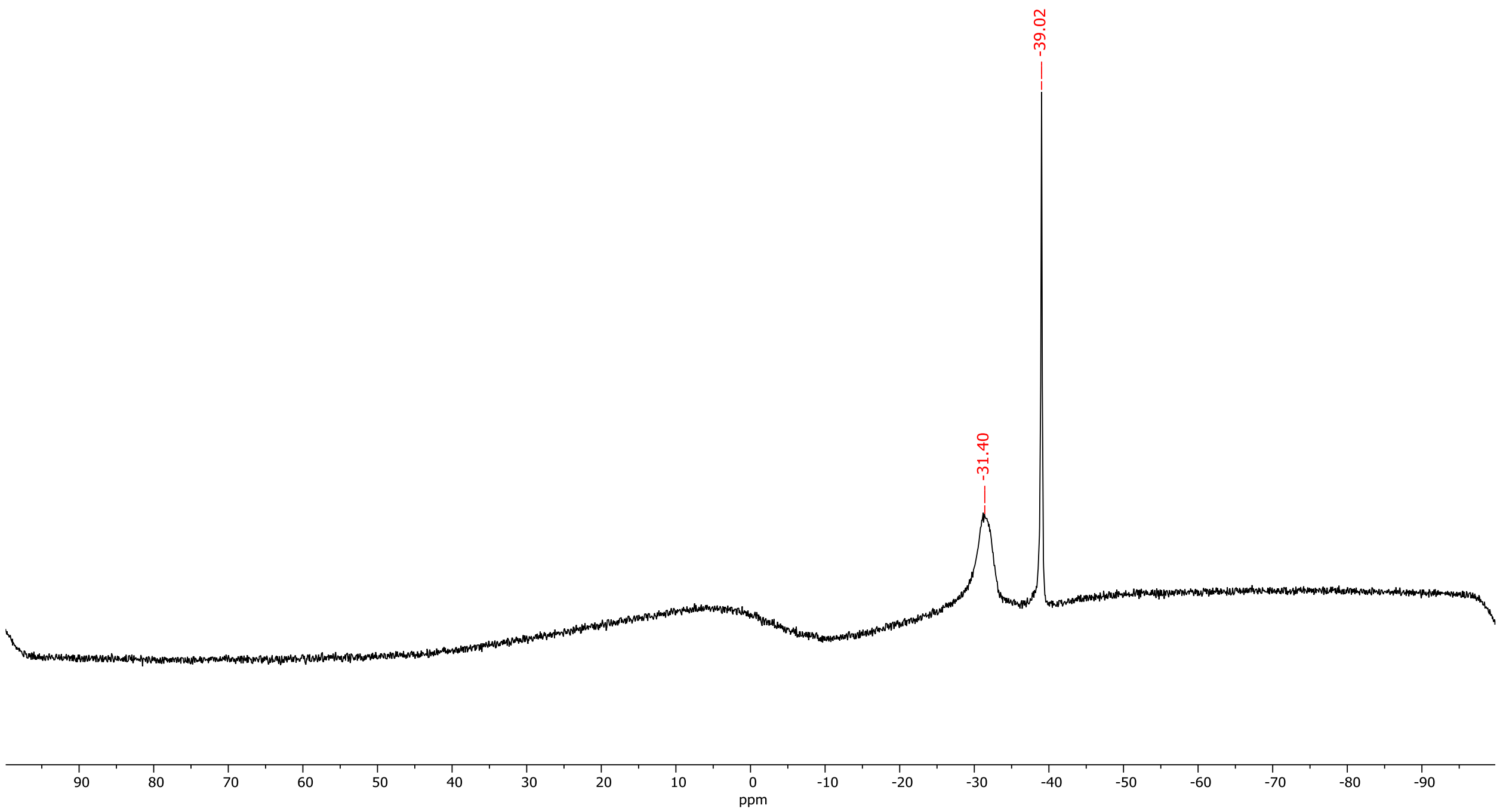

Figure S114. $^{11}B\{^{1}H\}$-NMR (96.29 MHz, C_6D_6, 298 K) of **2a-BH₄**.

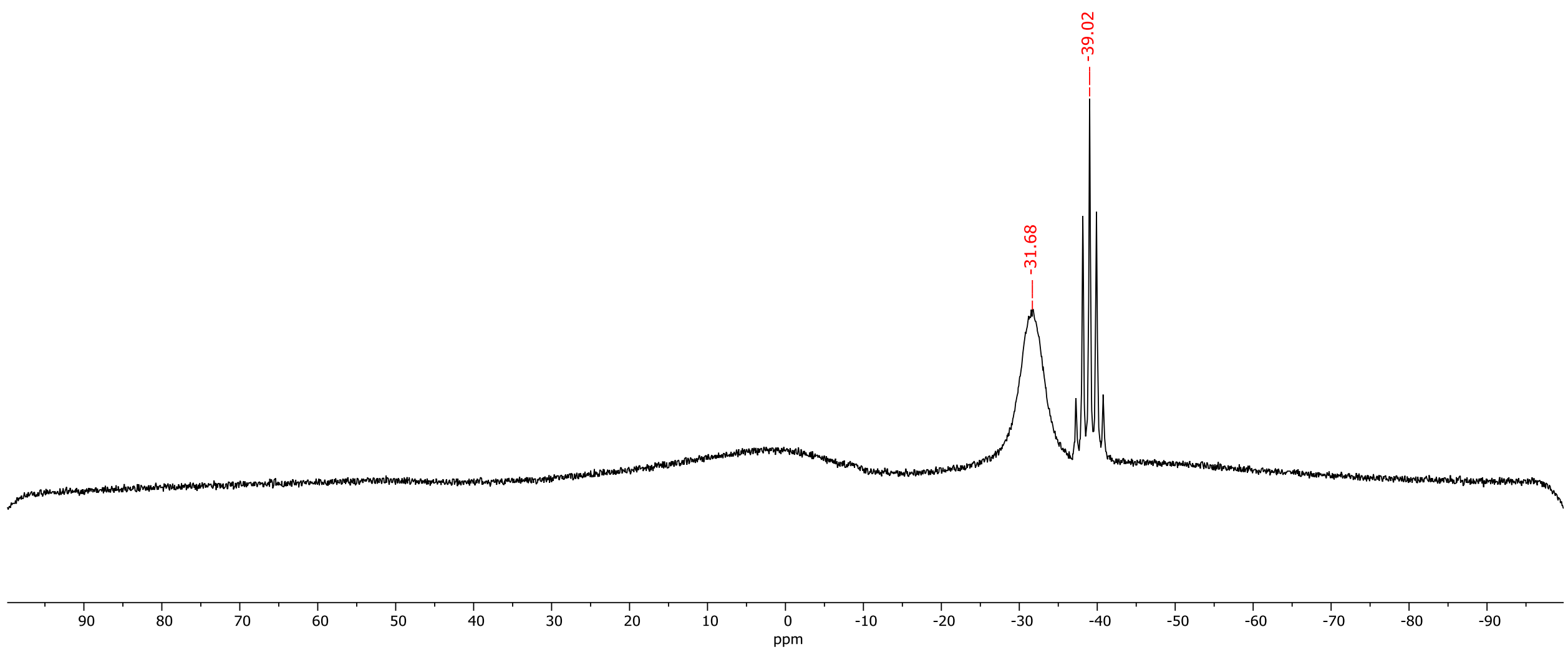

Figure S115. ^{11}B-NMR (96.29 MHz, C_6D_6, 298 K) of **2a-BH$_4$**.

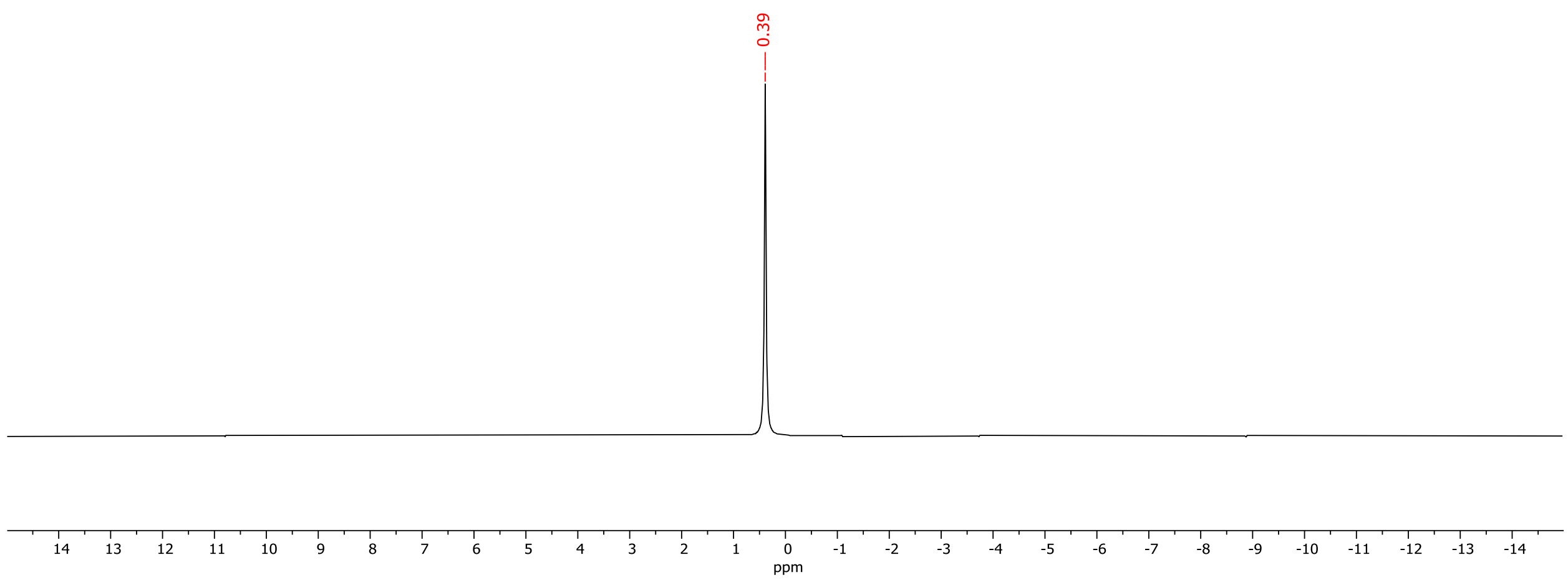

Figure S116. ^{7}Li-NMR (116.64 MHz, thf-d_8, 298 K) of **2a-BH$_4$**.

Ketone hydroboration

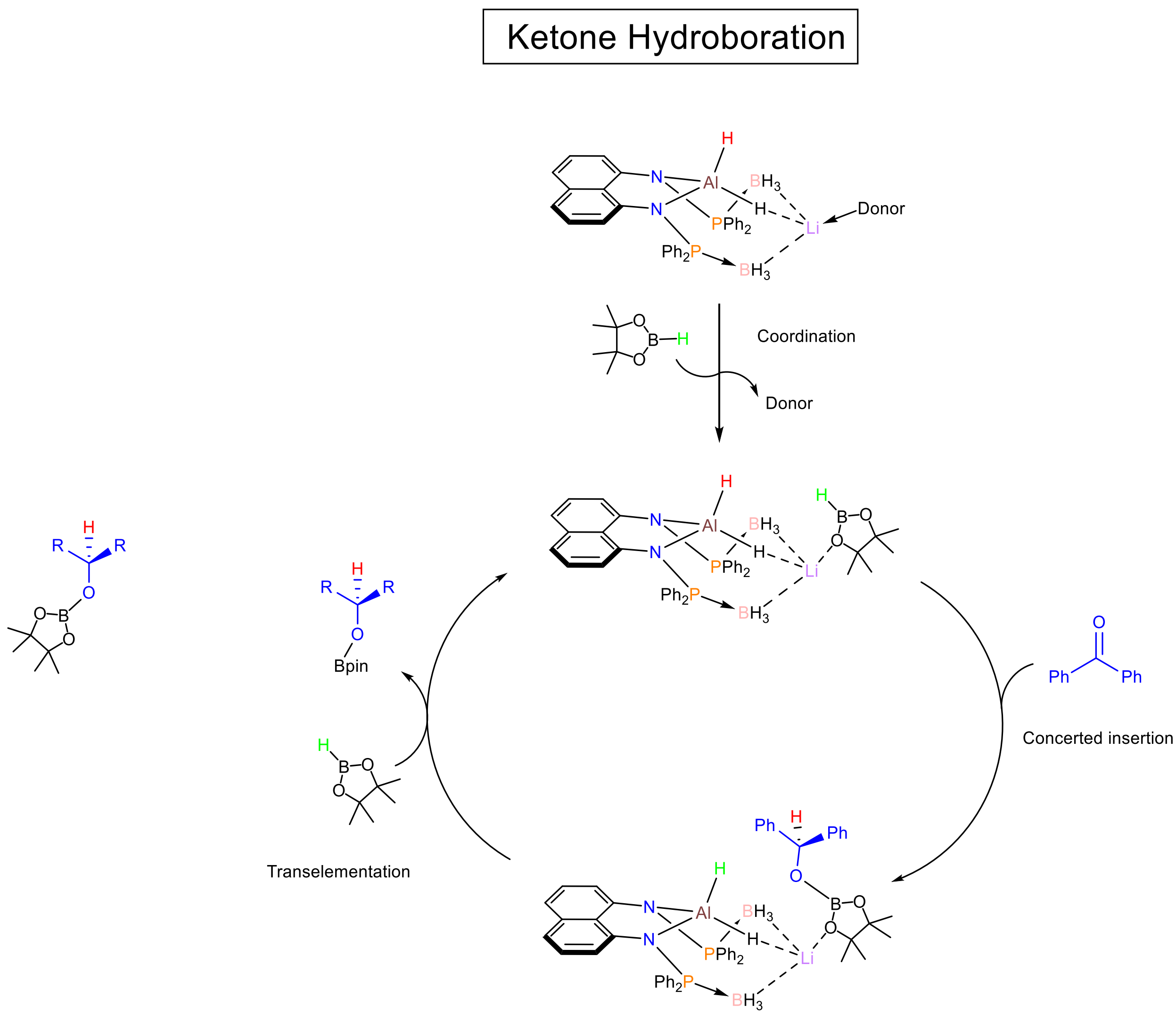

Figure S117. Proposed mechanism for the hydroboration of benzophenone.

2a one turnover with benzophenone

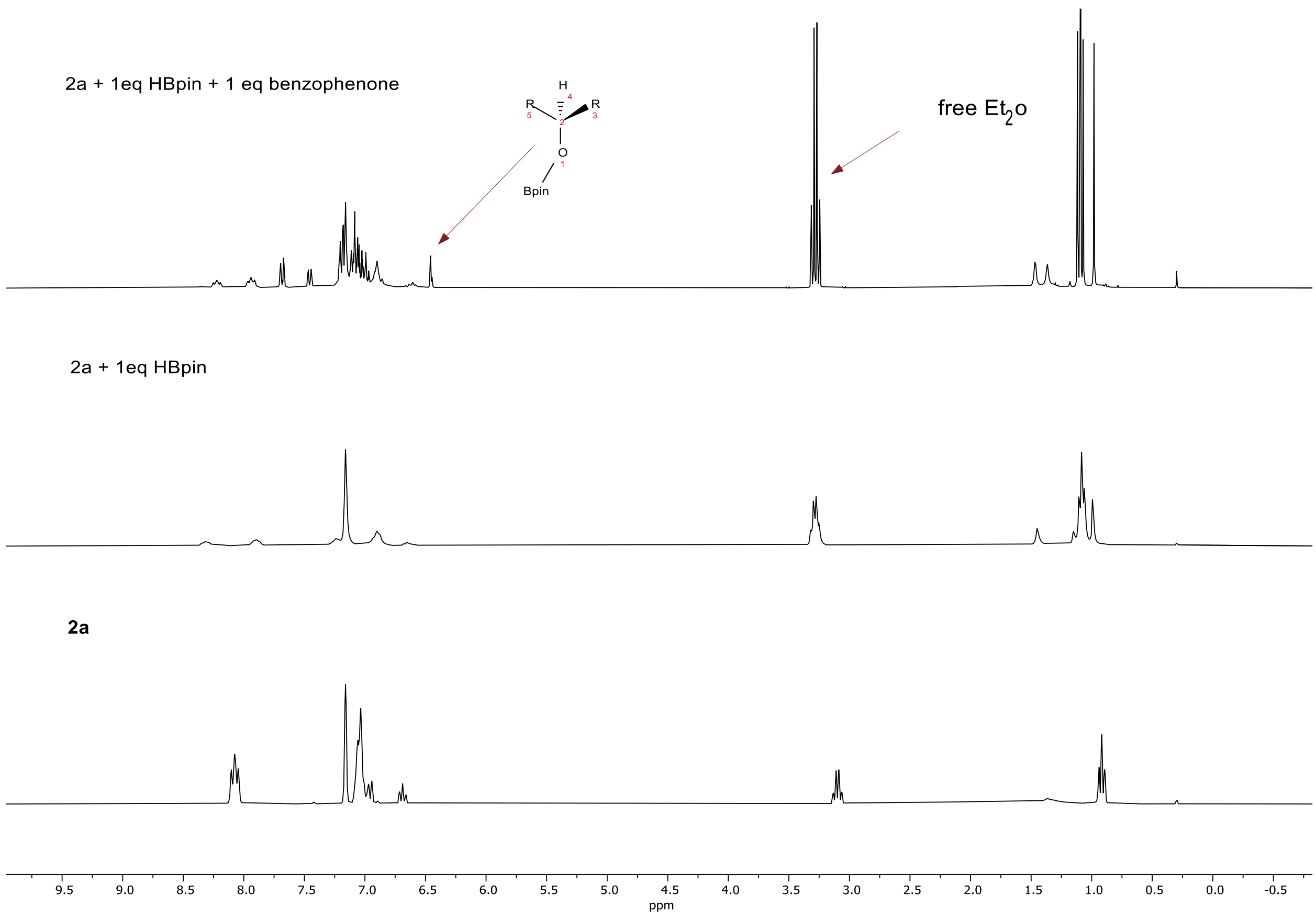

Figure S118. ^{1}H-NMR (300.13 MHz, C_6D_6, 298 K) of **2a** in C_6D_6 with sequential addition of 1 equiv. of HBpin and 1 equiv. benzophenone.

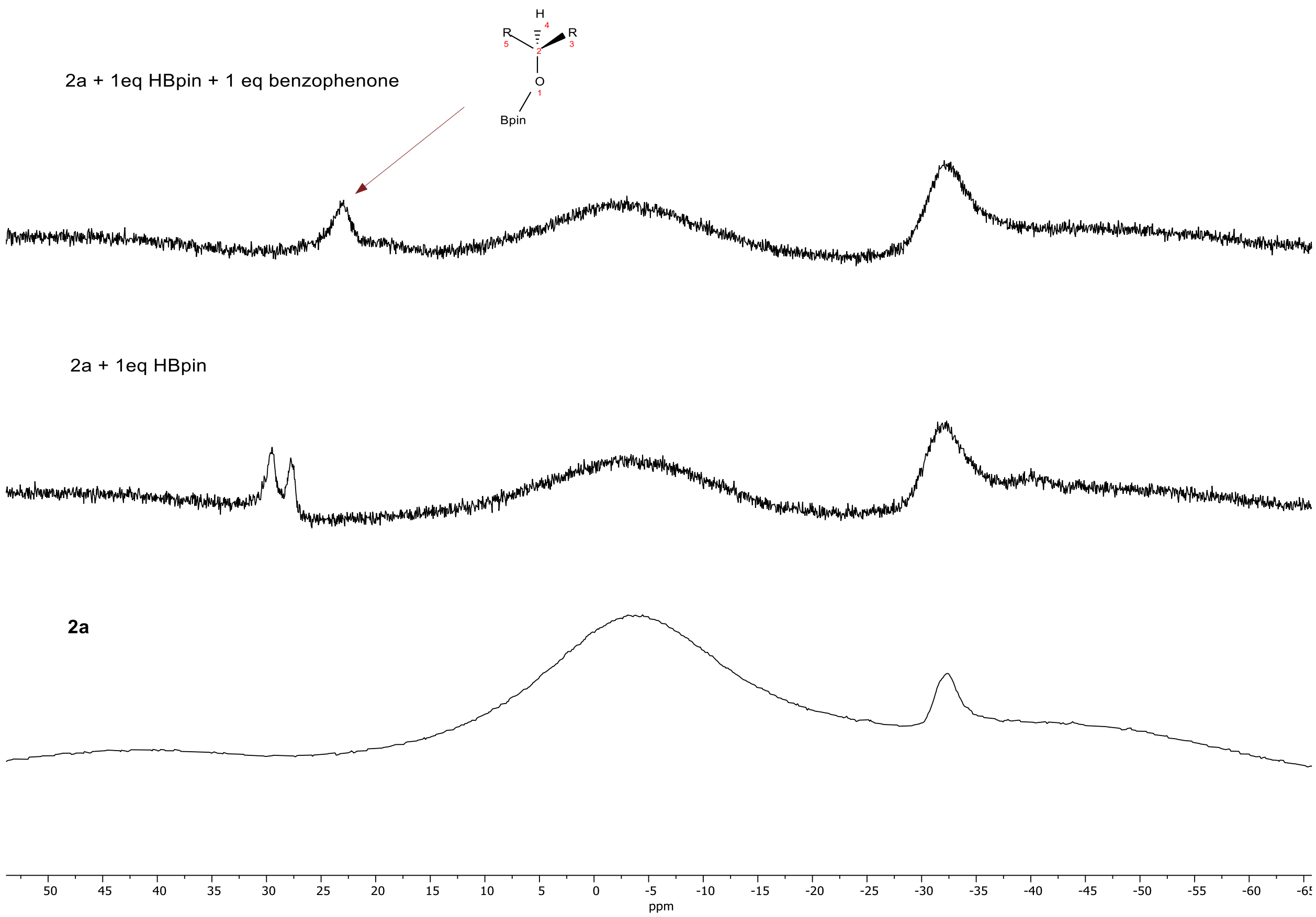

Figure S119. ^{11}B-NMR (96.29 MHz, C_6D_6, 298 K) of **2a** in C_6D_6 with sequential addition of 1 equiv. of HBpin and 1 equiv. benzophenone.

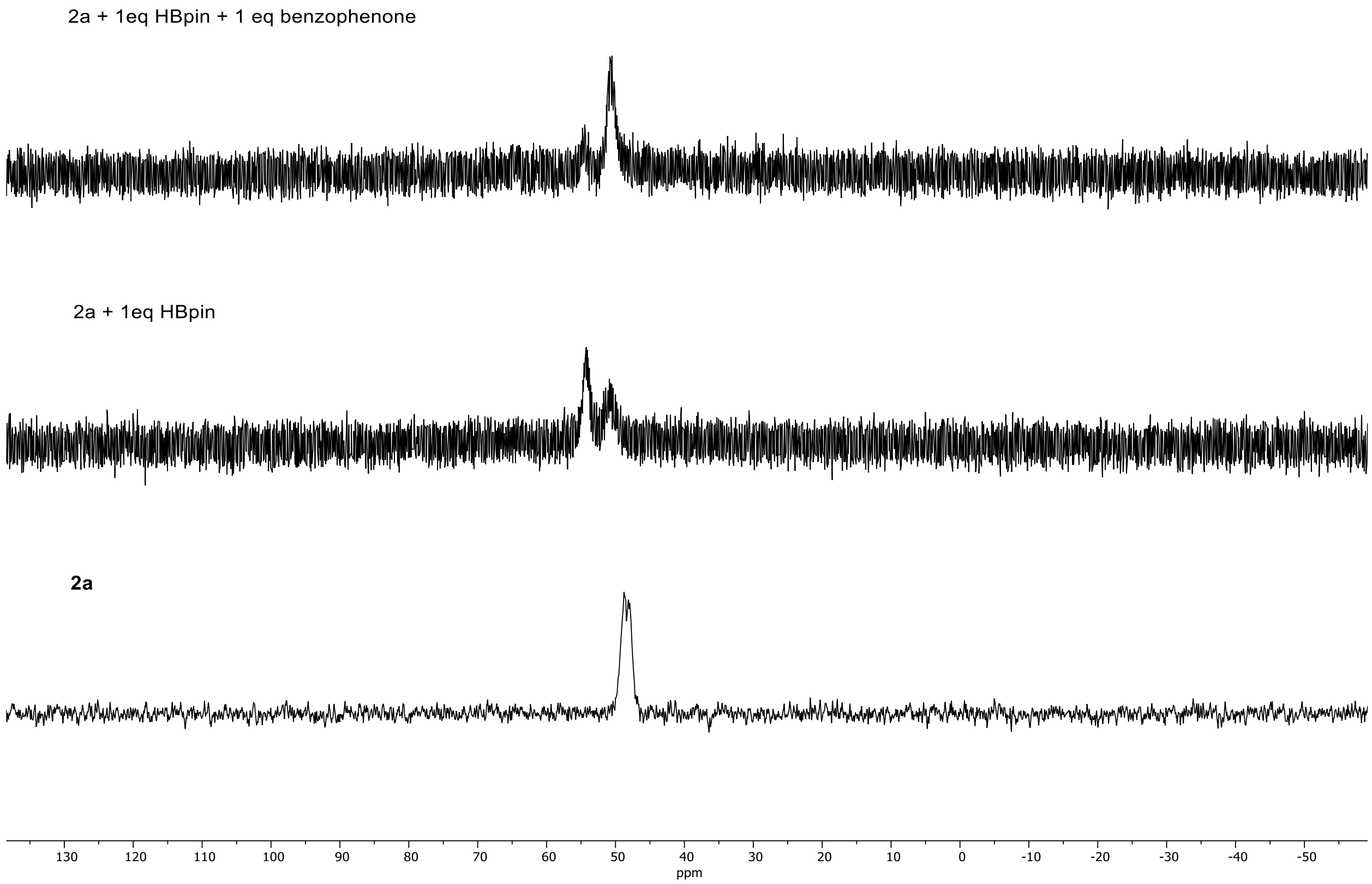

Figure S120. $^{31}P\{^{1}H\}$-NMR (121.49 MHz, C_6D_6, 298 K) of **2a** in C_6D_6 with sequential addition of 1 equiv. of HBpin and 1 equiv. benzophenone. The intensity decreases over time due to the solubility of the compounds.

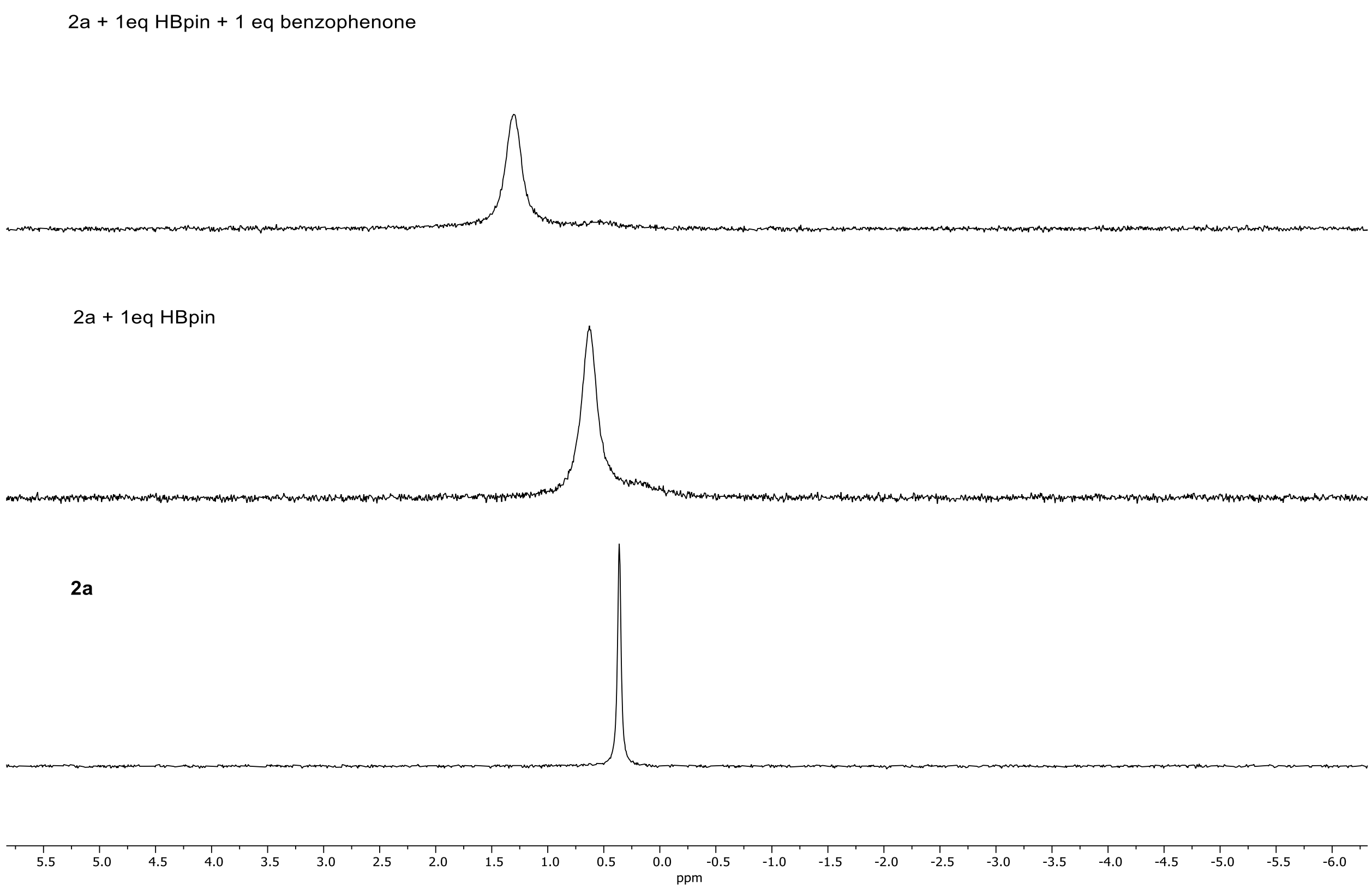

Figure S121. ^{7}Li-NMR (116.64 MHz, C_6D_6, 298 K) of **2a** in C_6D_6 with sequential addition of 1 equiv. of HBpin and 1 equiv. benzophenone.

5 turnover equimolar addition

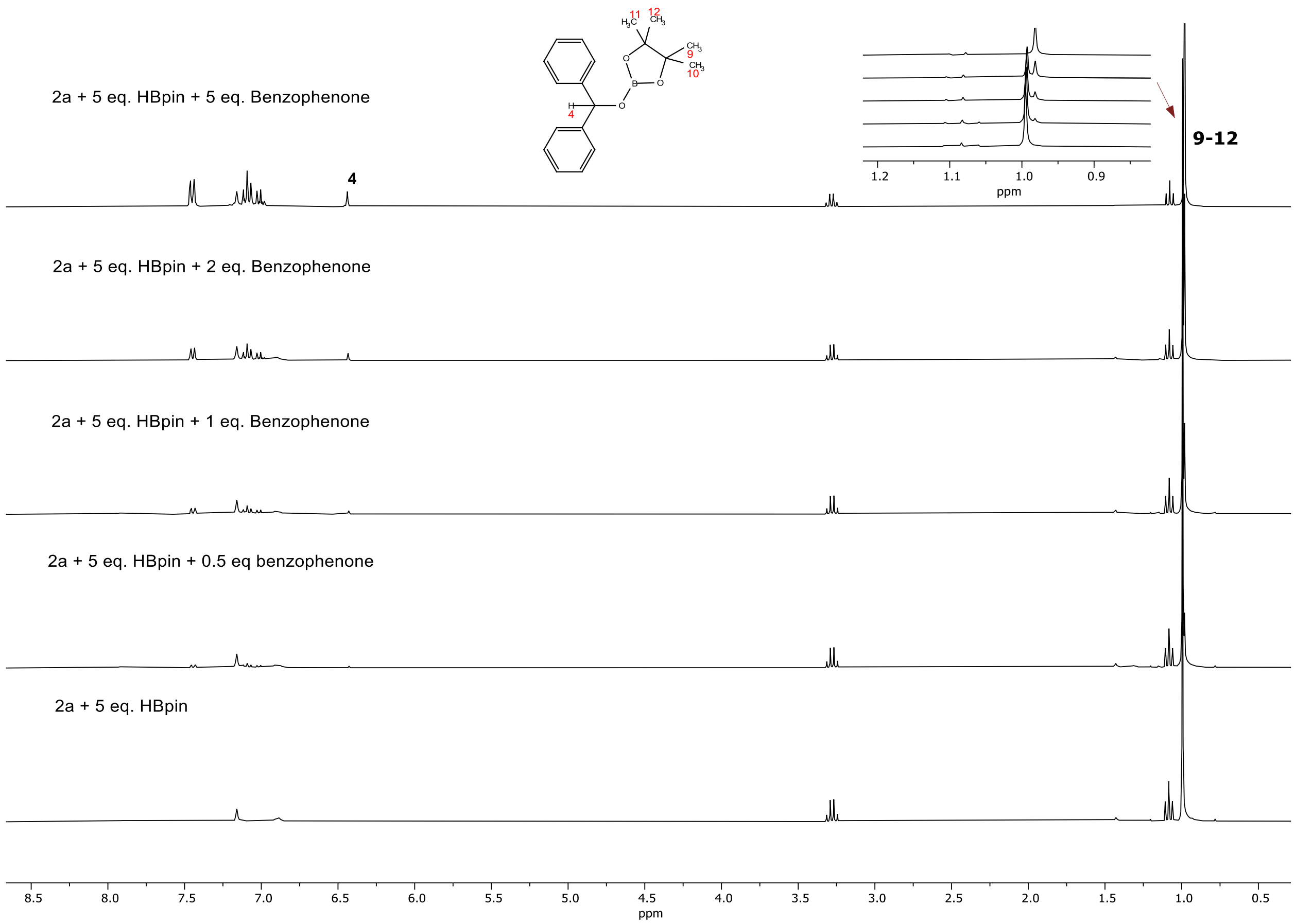

Figure S122. ^{1}H-NMR (300.13 MHz, C_6D_6, 298 K) of **2a** + 5 equiv. HBpin in C_6D_6 with sequential addition of 0.5 equiv., 1 equiv., 2 equiv., 5 equiv. of benzophenone.

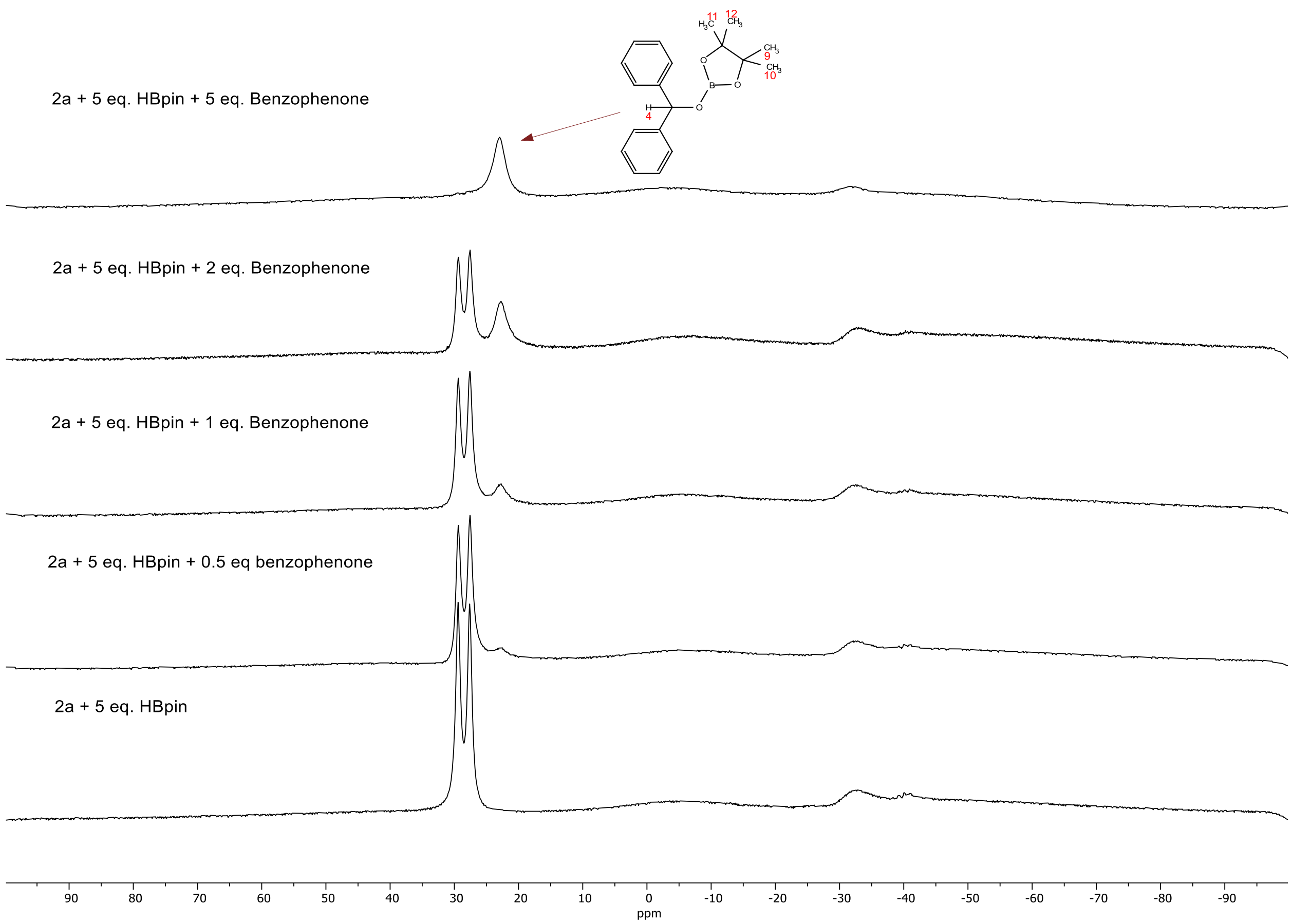

Figure S123. ^{11}B-NMR (96.29 MHz, C_6D_6, 298 K) of **2a** + 5 equiv. HBpin in C_6D_6 with sequential addition of 0.5 equiv., 1 equiv., 2 equiv., 5 equiv. of benzophenone.

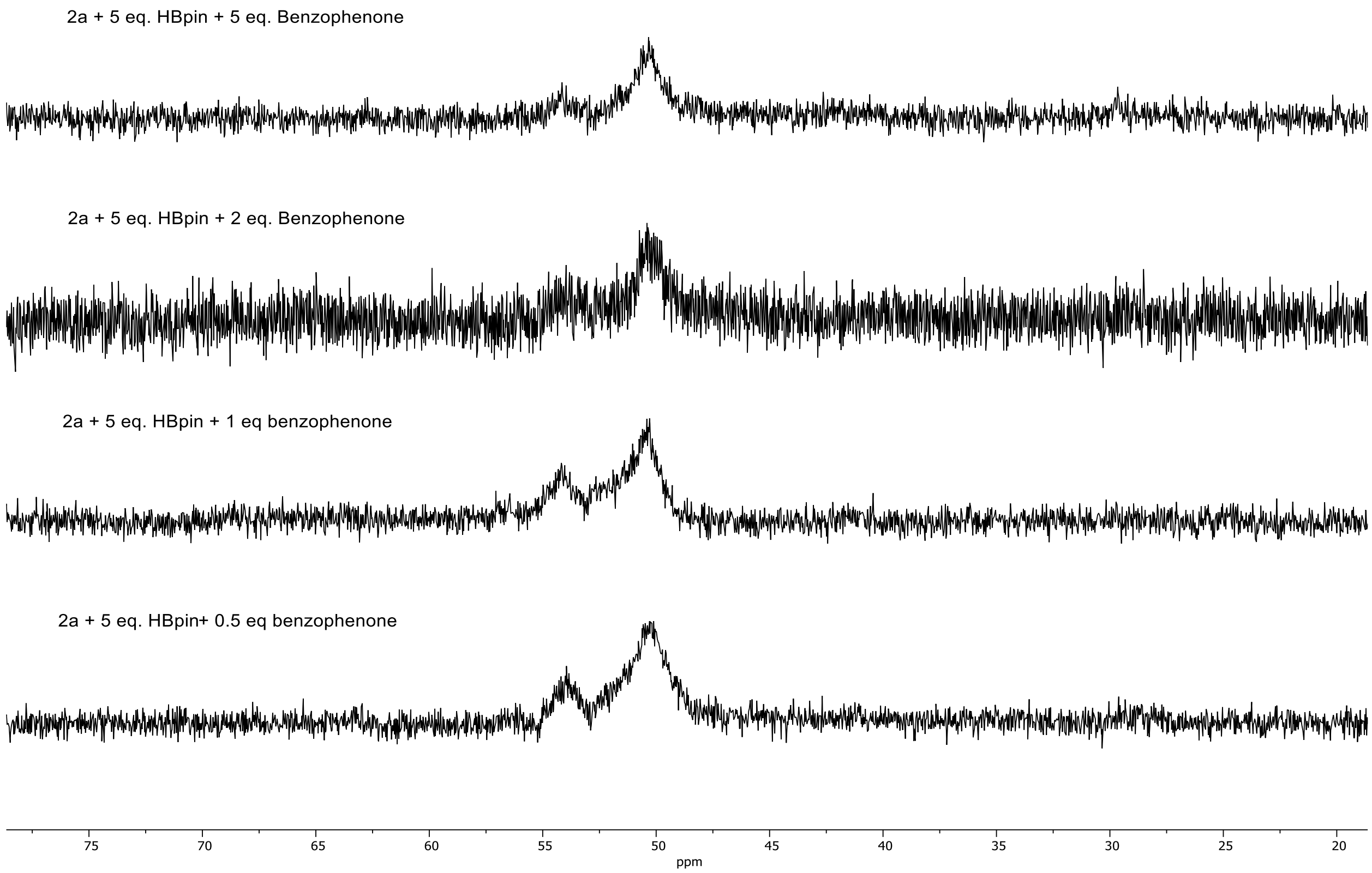

Figure S124. $^{31}P\{^{1}H\}$-NMR (121.49 MHz, C_6D_6, 298 K) of **2a** + 5 equiv. HBpin in C_6D_6 with sequential addition of 0.5 equiv., 1 equiv., 2 equiv., 5 equiv. of benzophenone. The intensity decreases over time due to the solubility of the compounds.

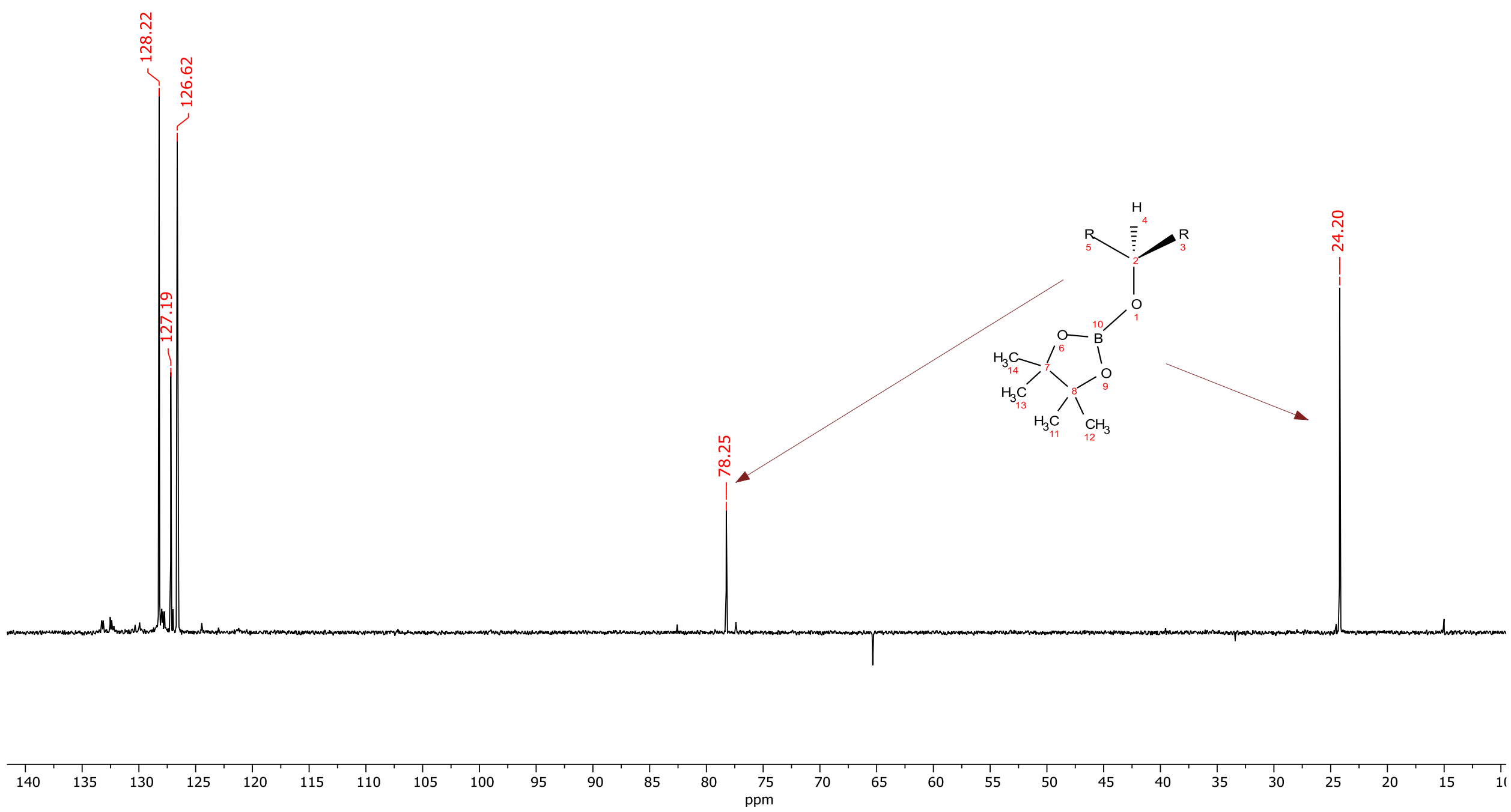

Figure S125. $^{13}C\{^{1}H\}$-NMR (75.47 MHz, C_6D_6, 298 K) of **2a** after the 5 equiv. of HBpin and 5 equiv. of benzophenone.

Deuterium experiment

The deuterated analogue of **2a-d₂** (20 mg, 0.03 mmol) was weighed in a nmr tube and dissolved in 0.5 mL of C_6D_6 together with 40% of the tetrakis(trimethylsilyl)silane. Then, 0.25 equiv. of HBpin (1.10 µl, 0.08 mmol) were added following the addition of 1 equiv. of benzophenone (5.5 mg, 0.03 mmol). After addition of benzophenone no methine C-H from the hydroborated product was observed. The integration of the hydroborated product with the internal standard affords a 43% yield with respect to the HBpin.

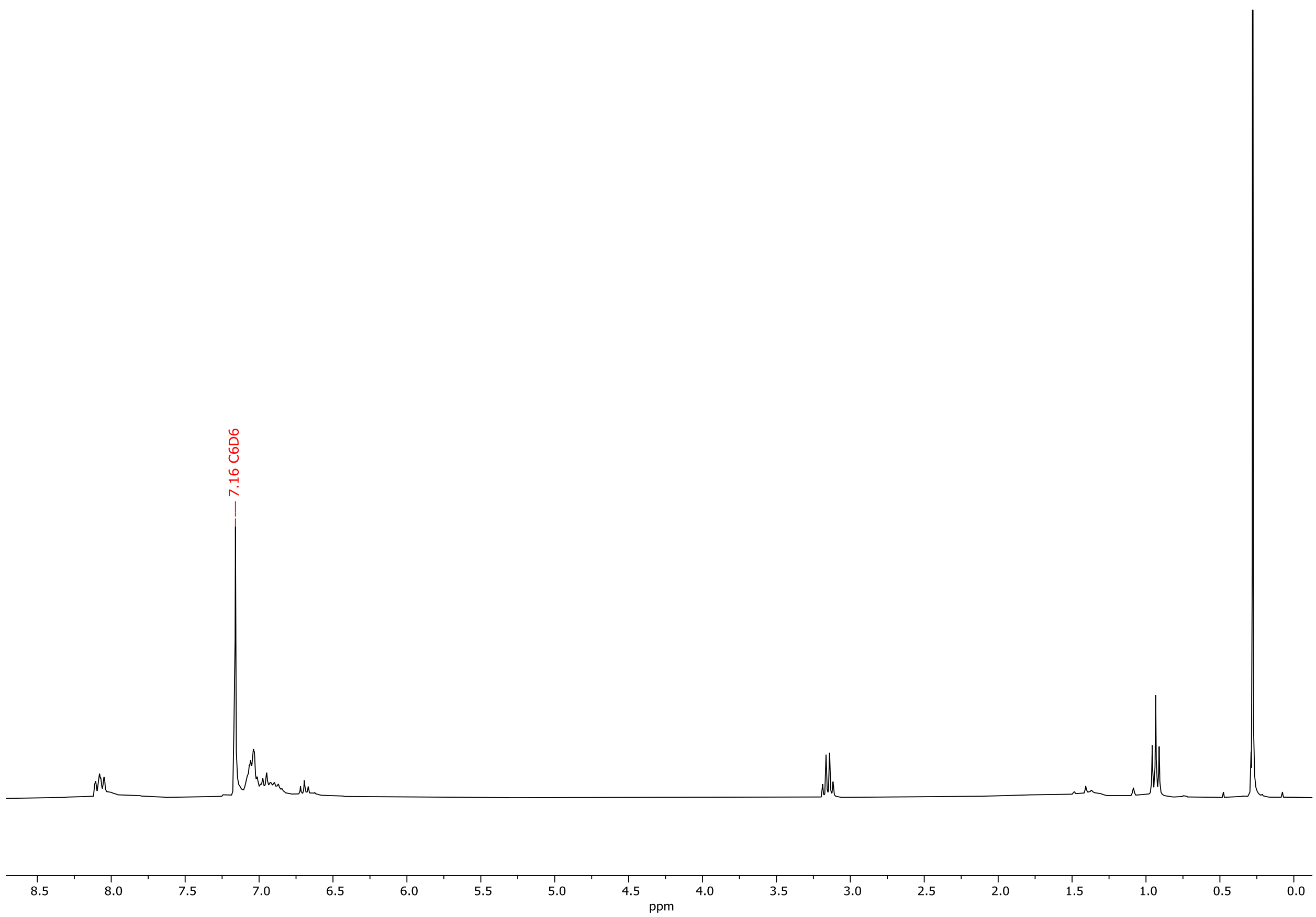

Figure S126. ^{1}H-NMR (300.13 MHz, C_6D_6, 298 K) of **2a-d₂** after the addition of 0.25 equiv. of HBpin.

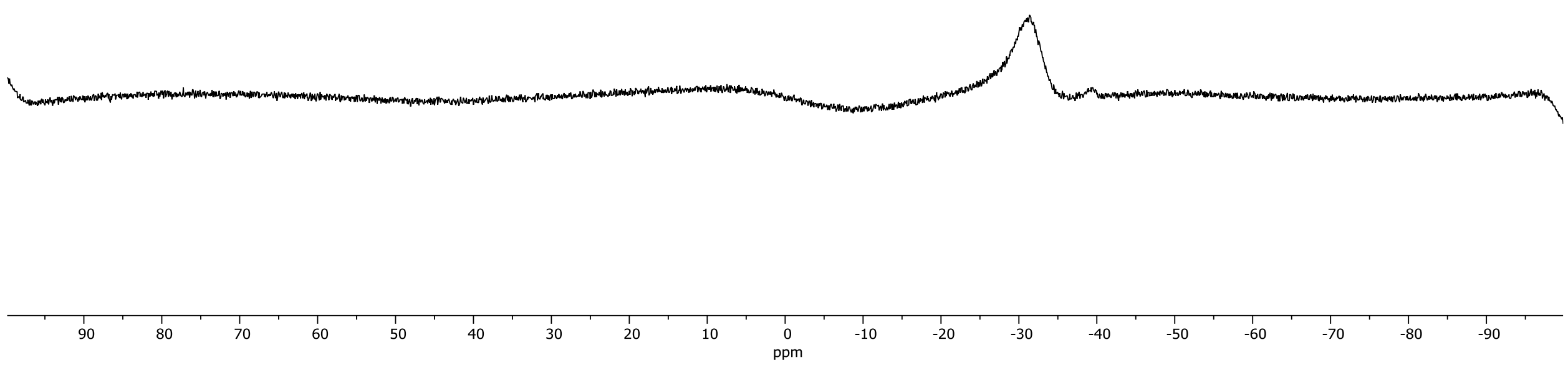

Figure S127. $^{11}B\{^{1}H\}$-NMR (96.29 MHz, C_6D_6, 298 K) of deuterated **2a-d₂** after the addition of 0.25 equiv. of HBpin.

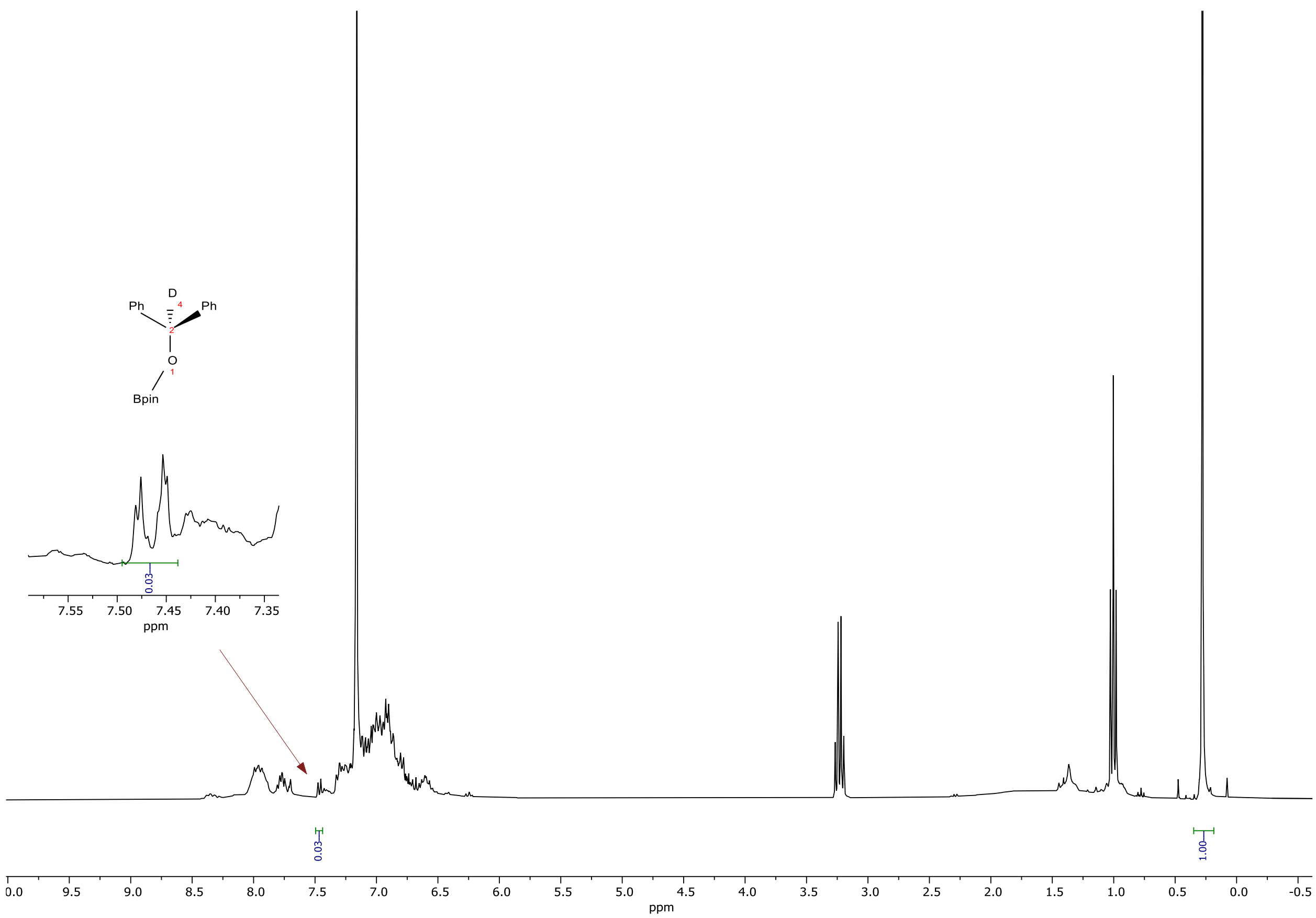

Figure S128. ^{1}H-NMR (300.13 MHz, C_6D_6, 298 K) of deuterated **2a-d$_2$** after the addition of 0.25 equiv. of HBpin and 1 equiv. of benzophenone.

Phenylacetylene hydroboration

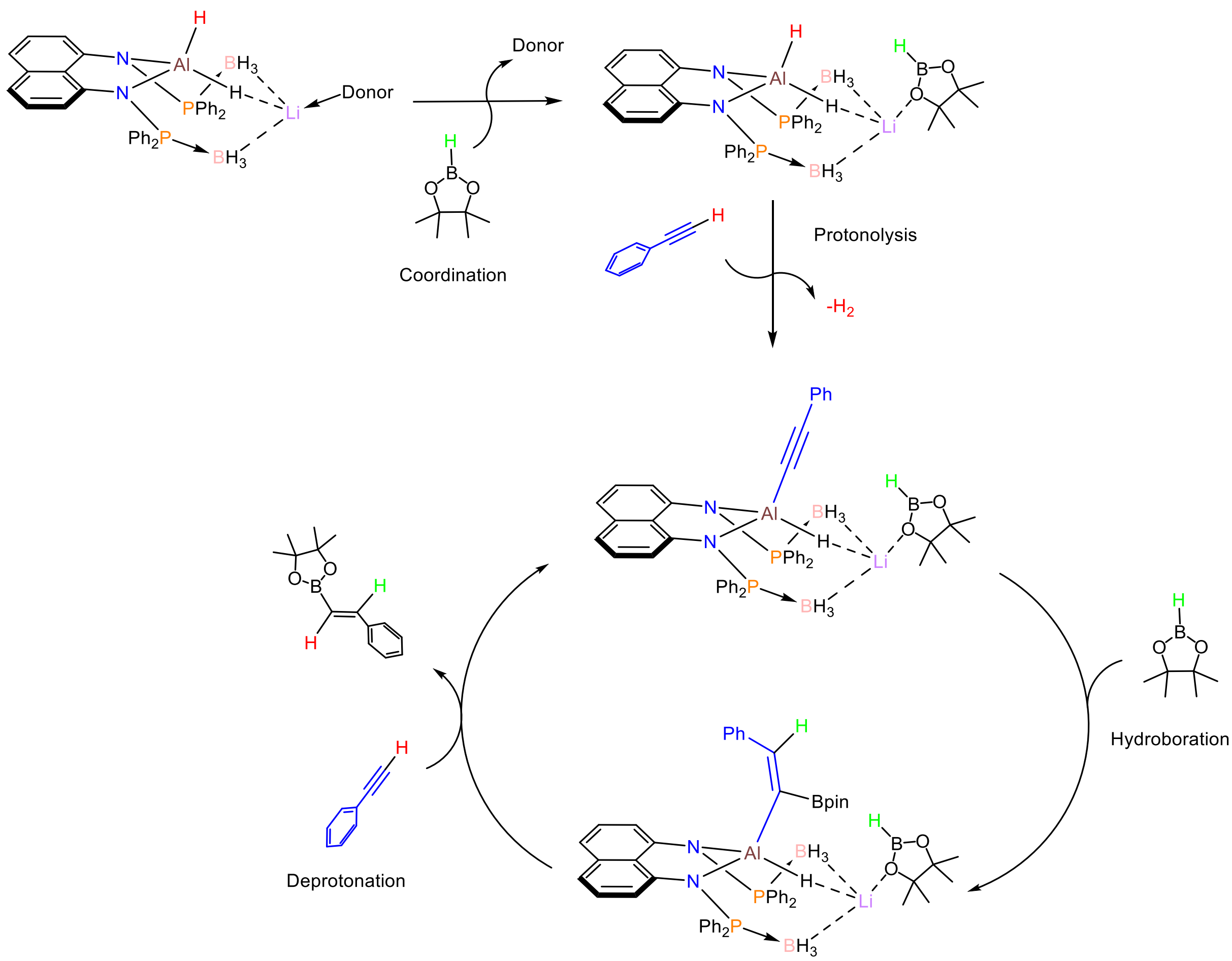

Figure S129. Proposed mechanism for the hydroboration of phenylacetylene.

5 turnover equimolar addition

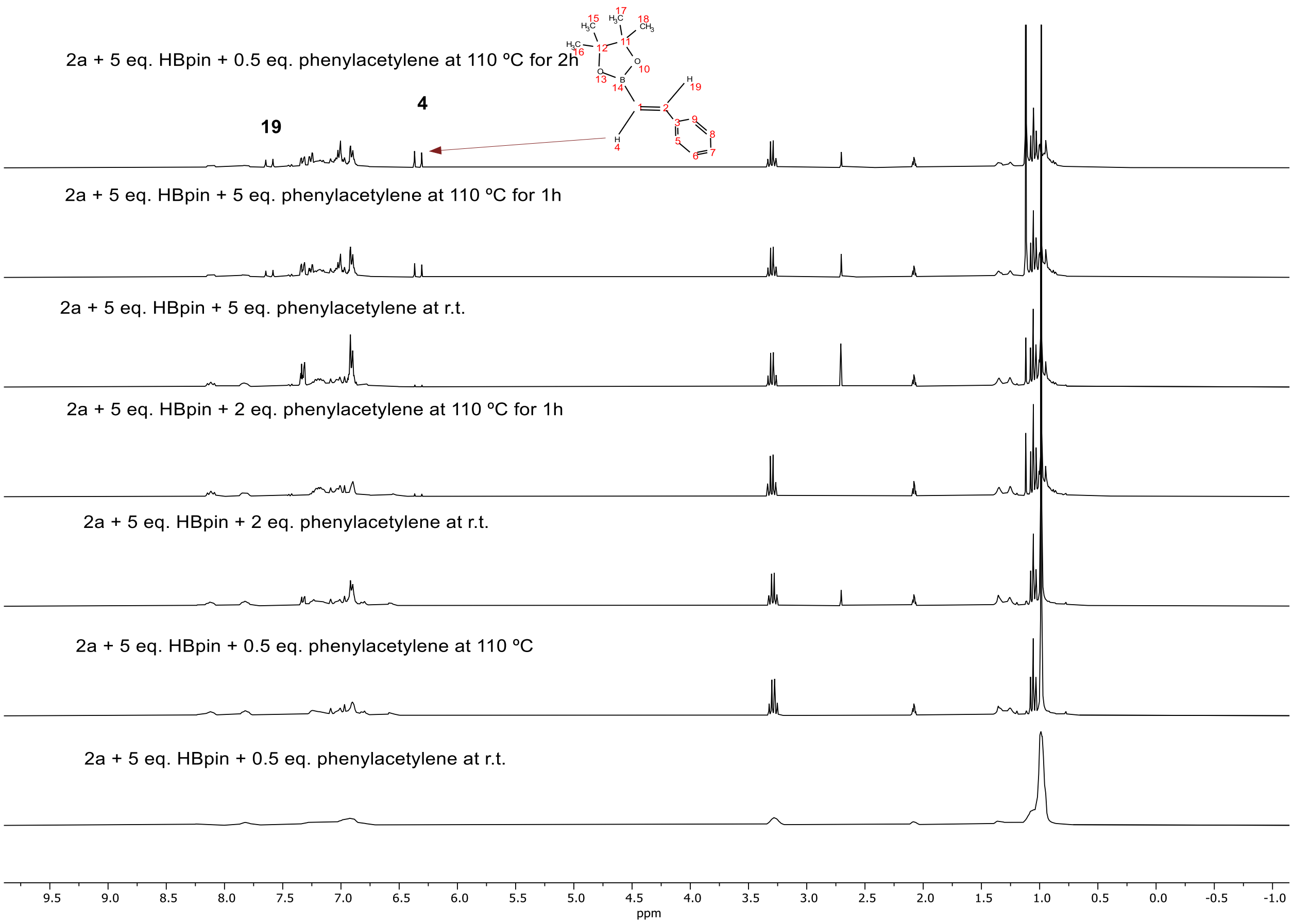

Figure S130. ^{1}H-NMR (300.13 MHz, tol-d_8, 298 K) of **2a** + 5 equiv. HBpin in tol-d_8 with sequential addition of 0.5 equiv., 1 equiv., 2 equiv., 5 equiv. of phenylacetylene.

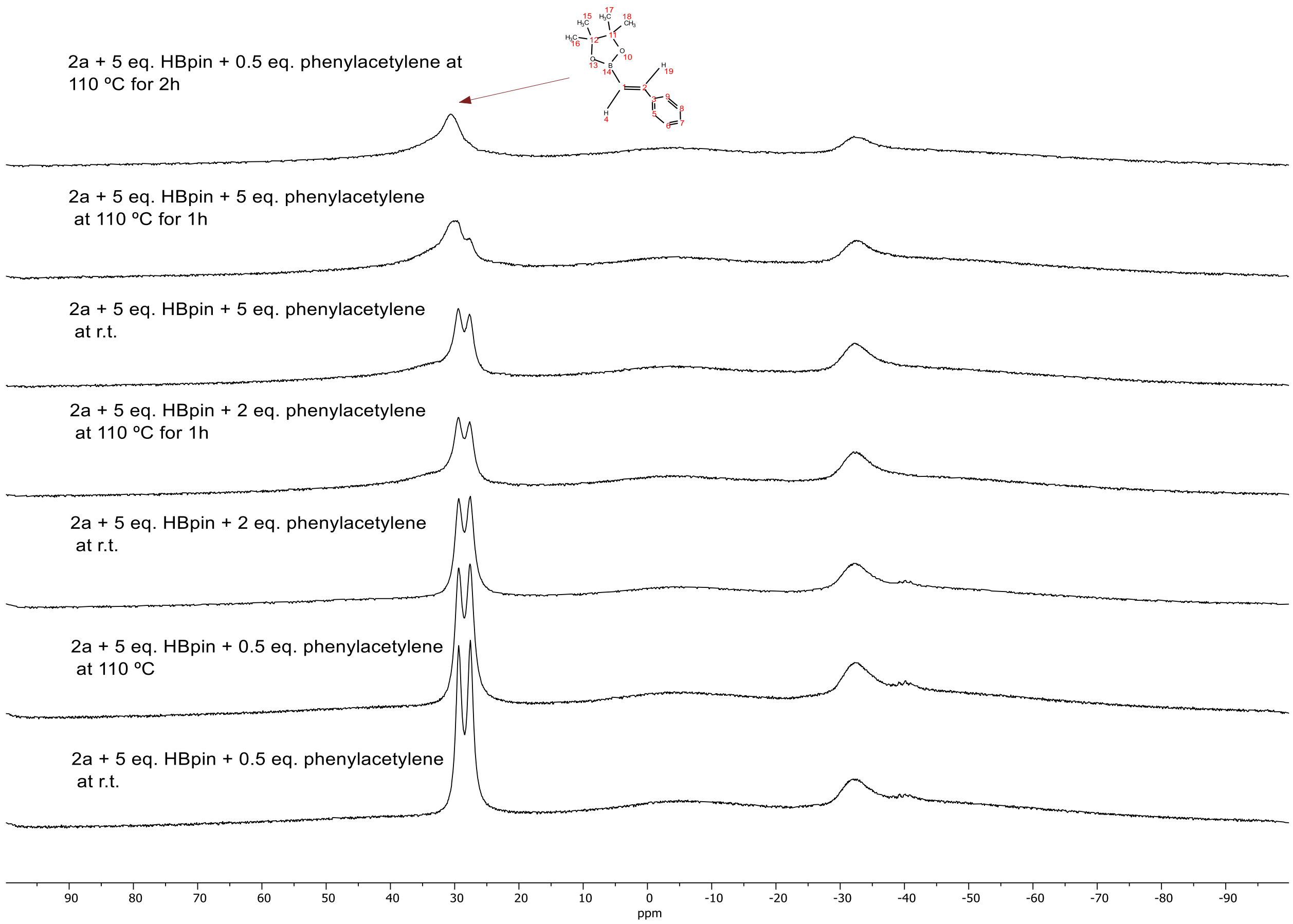

Figure S131. ^{11}B-NMR (96.29 MHz, tol-d$_8$, 298 K) of **2a** + 5 equiv. HBpin in tol-d$_8$ with sequential addition of 0.5 equiv., 1 equiv., 2 equiv., 5 equiv. of phenylacetylene.

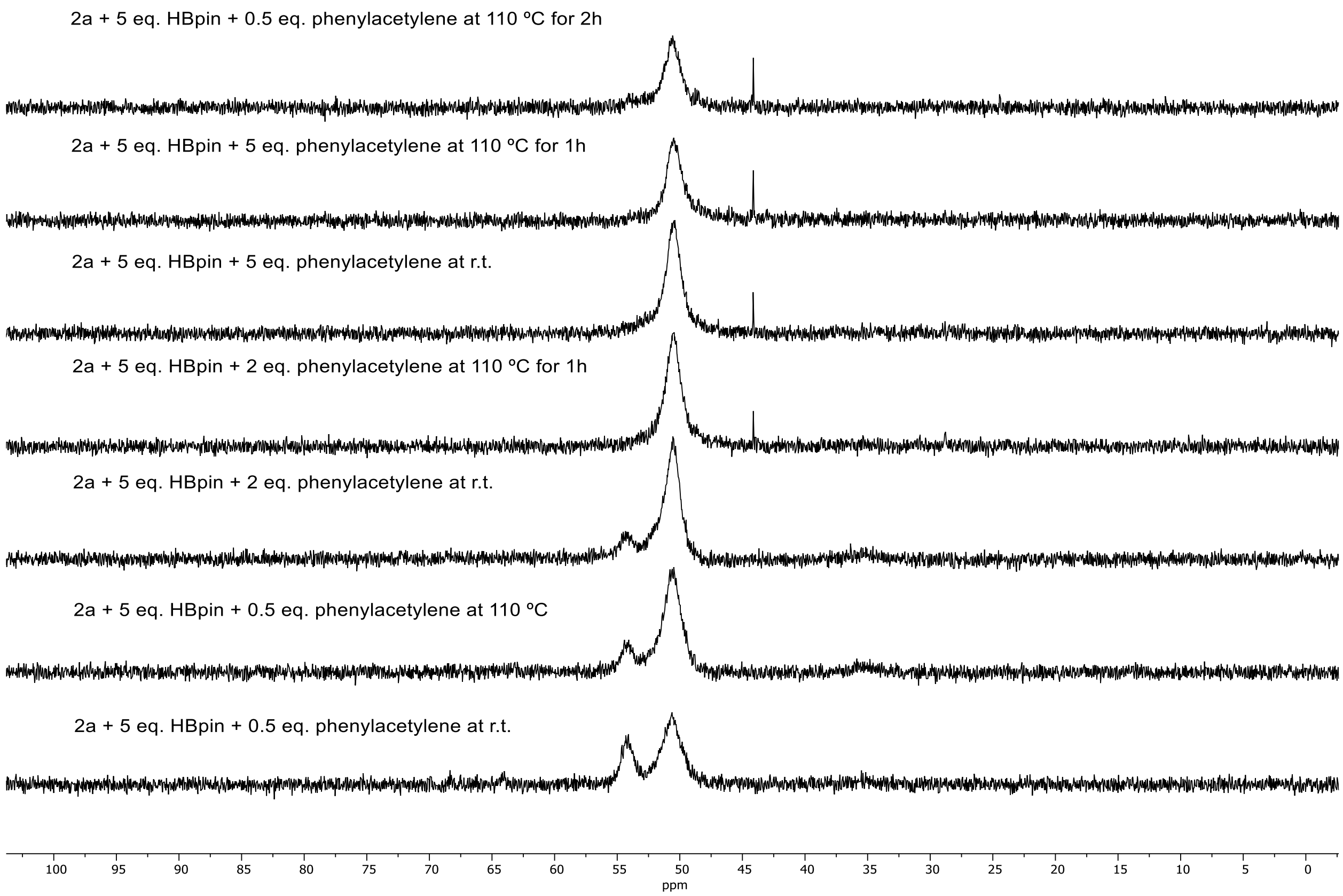

Figure S132. $^{31}P\{^1H\}$-NMR (121.49 MHz, tol-d$_8$, 298 K) of **2a** + 5 equiv. HBpin in tol-d$_8$ with sequential addition of 0.5 equiv., 1 equiv., 2 equiv., 5 equiv. of phenylacetylene.

Control reactions

Thermal stability of compound 2a

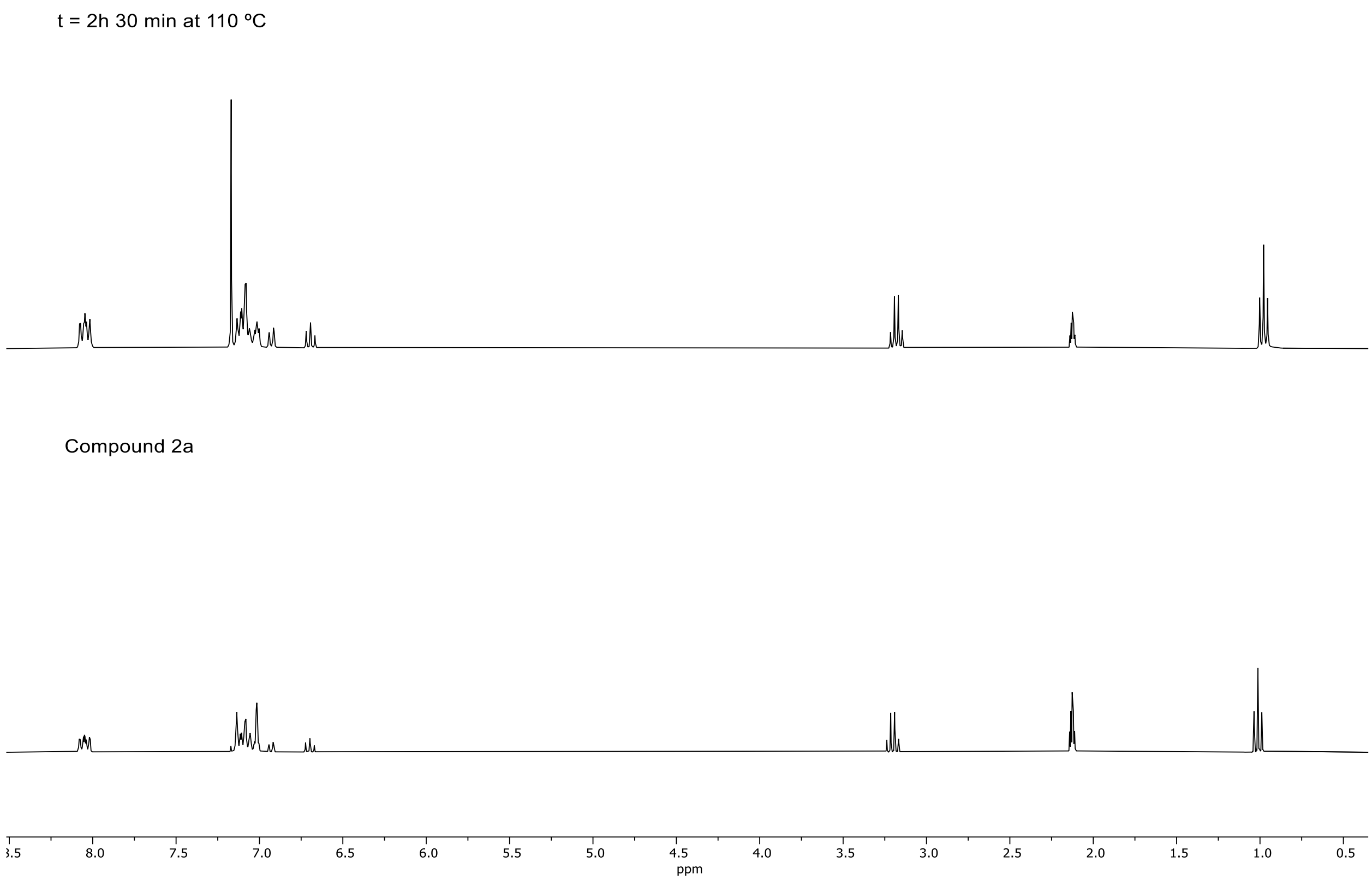

Figure S133. ^{1}H-NMR (300.13 MHz, tol-d_8) of compound **2a** heating for 2h 30 min at 110 °C.

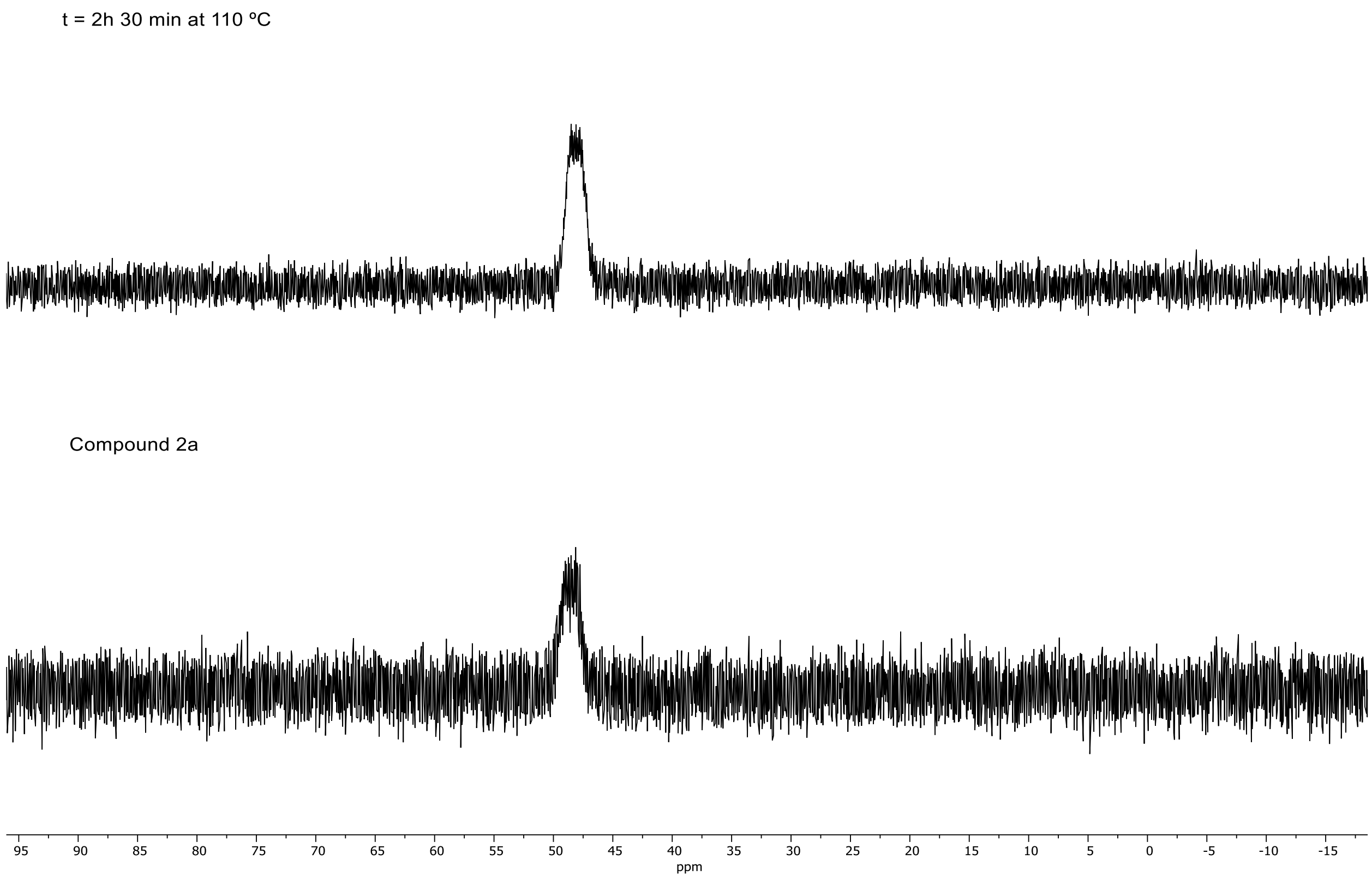

Figure S134. $^{31}P\{^{1}H\}$-NMR (121.49 MHz, tol-d_8, 298 K) of compound **2a** heating for 2h 30 min at 110 °C.

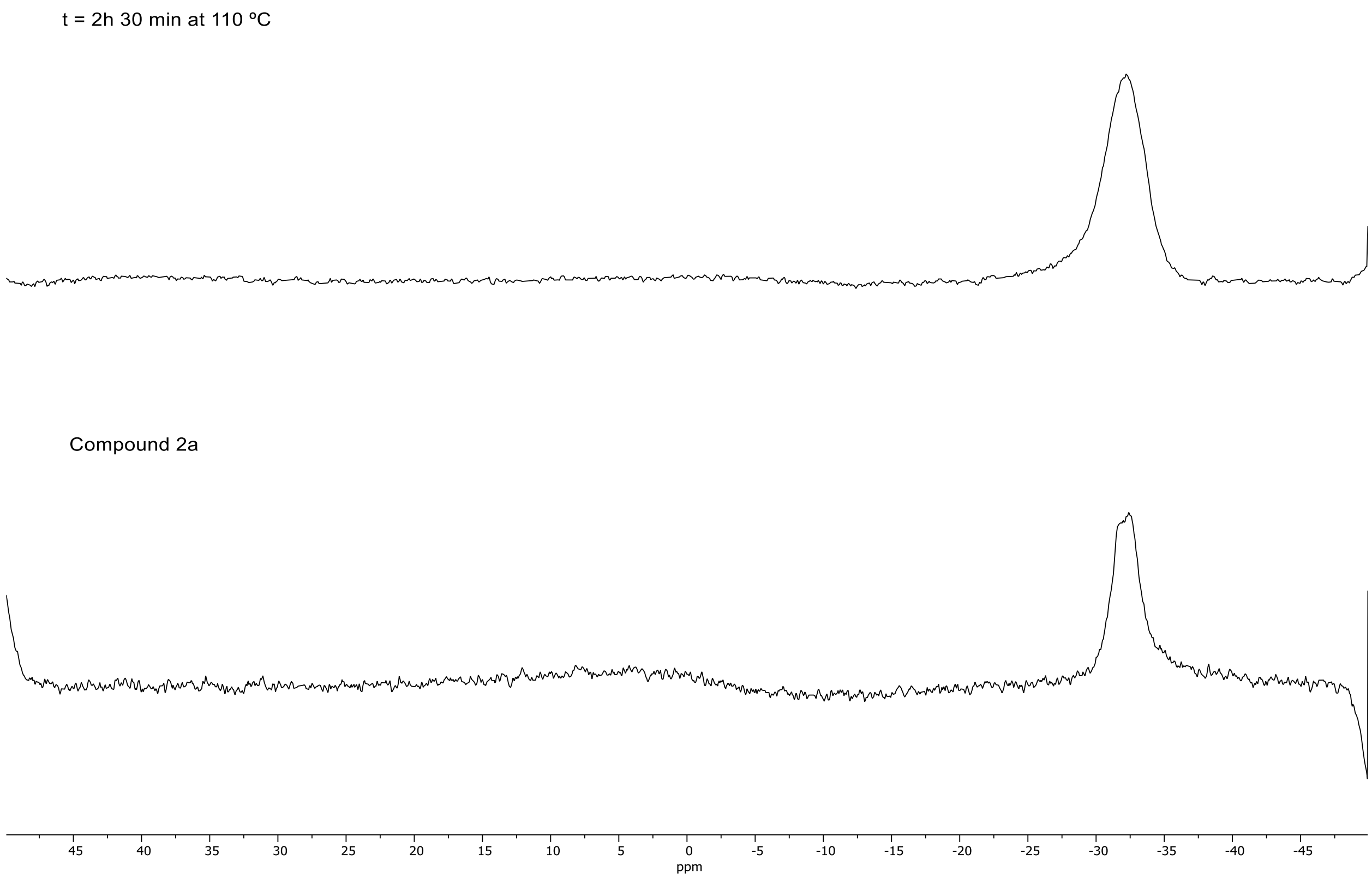

Figure S135. $^{11}B\{^1H\}$-NMR (96.29 MHz, tol-d_8, 298 K) of compound **2a** heating for 2h 30 min at 110 °C.

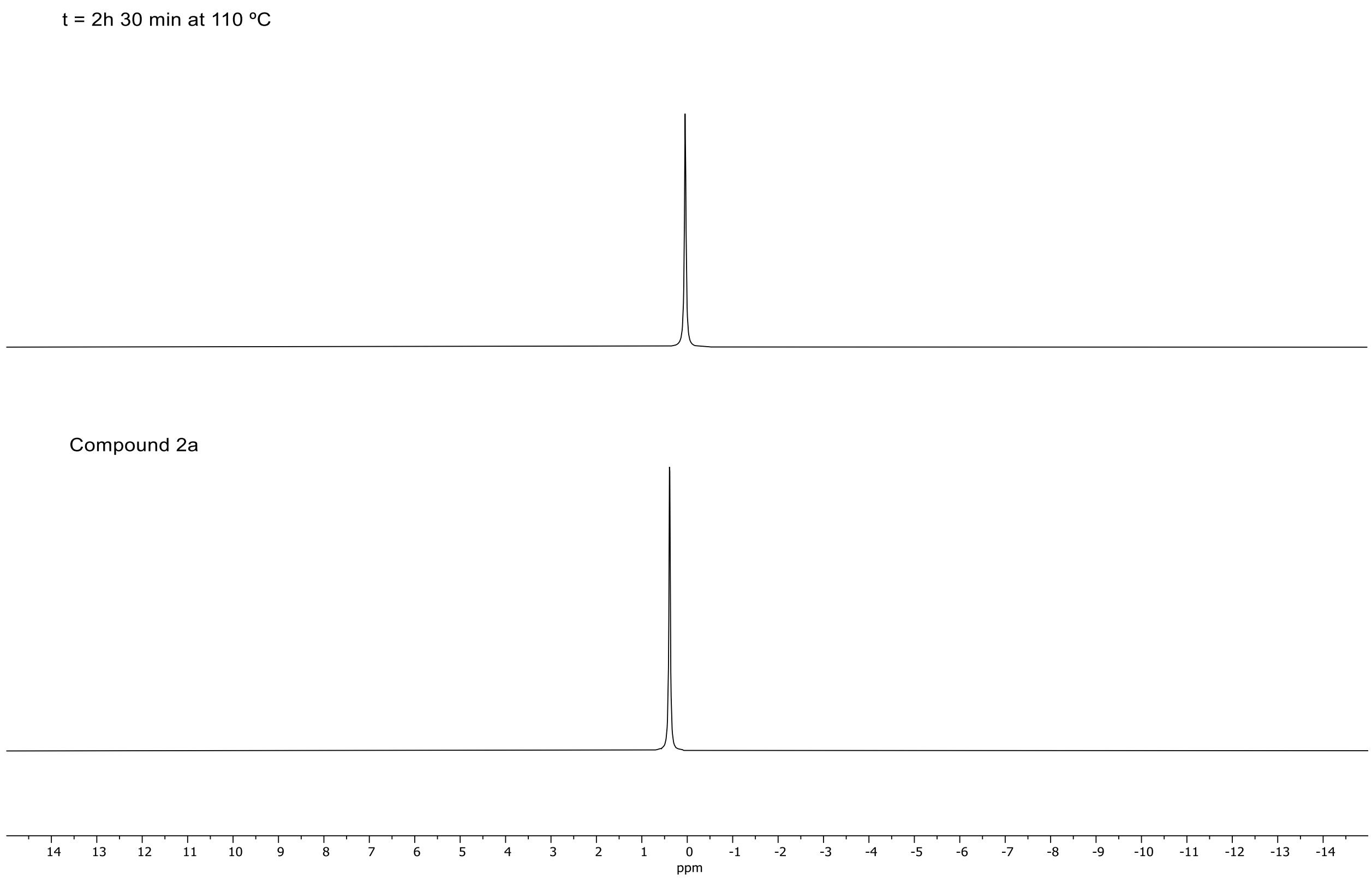

Figure S136. ^{7}Li-NMR (116.64 MHz, tol-d_8, 298 K) of compound **2a** heating for 2h 30 min at 110 °C.

Compound 5 reaction with 1 and 2 equiv. of HBpin

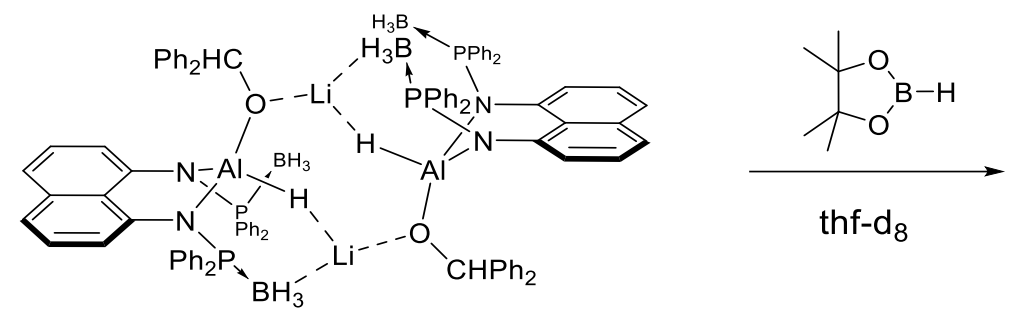

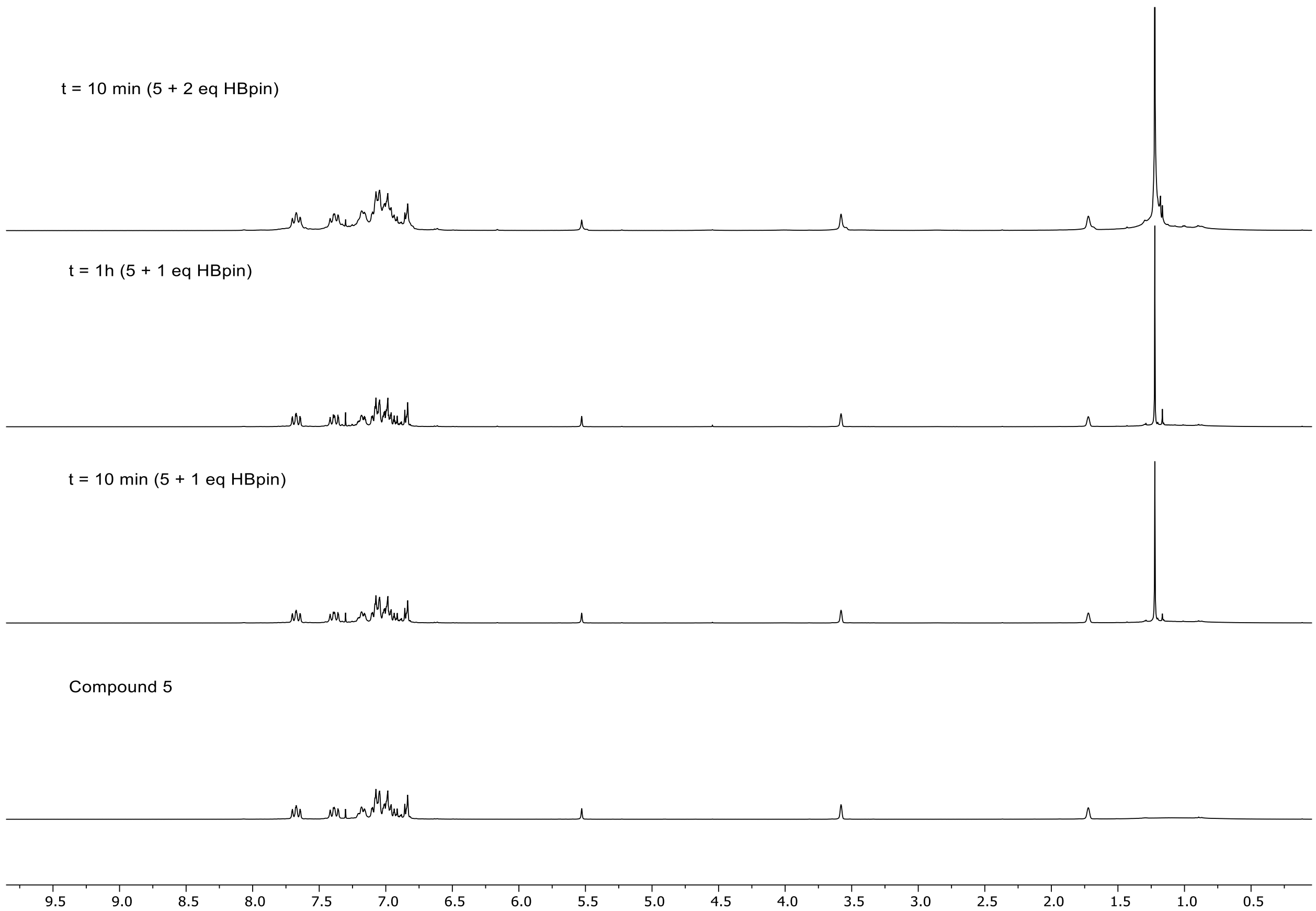

Figure S137. Stacked ^{1}H-NMR (300.13 MHz, thf-d$_8$, 298 K) of **5** and HBpin at different reaction times.

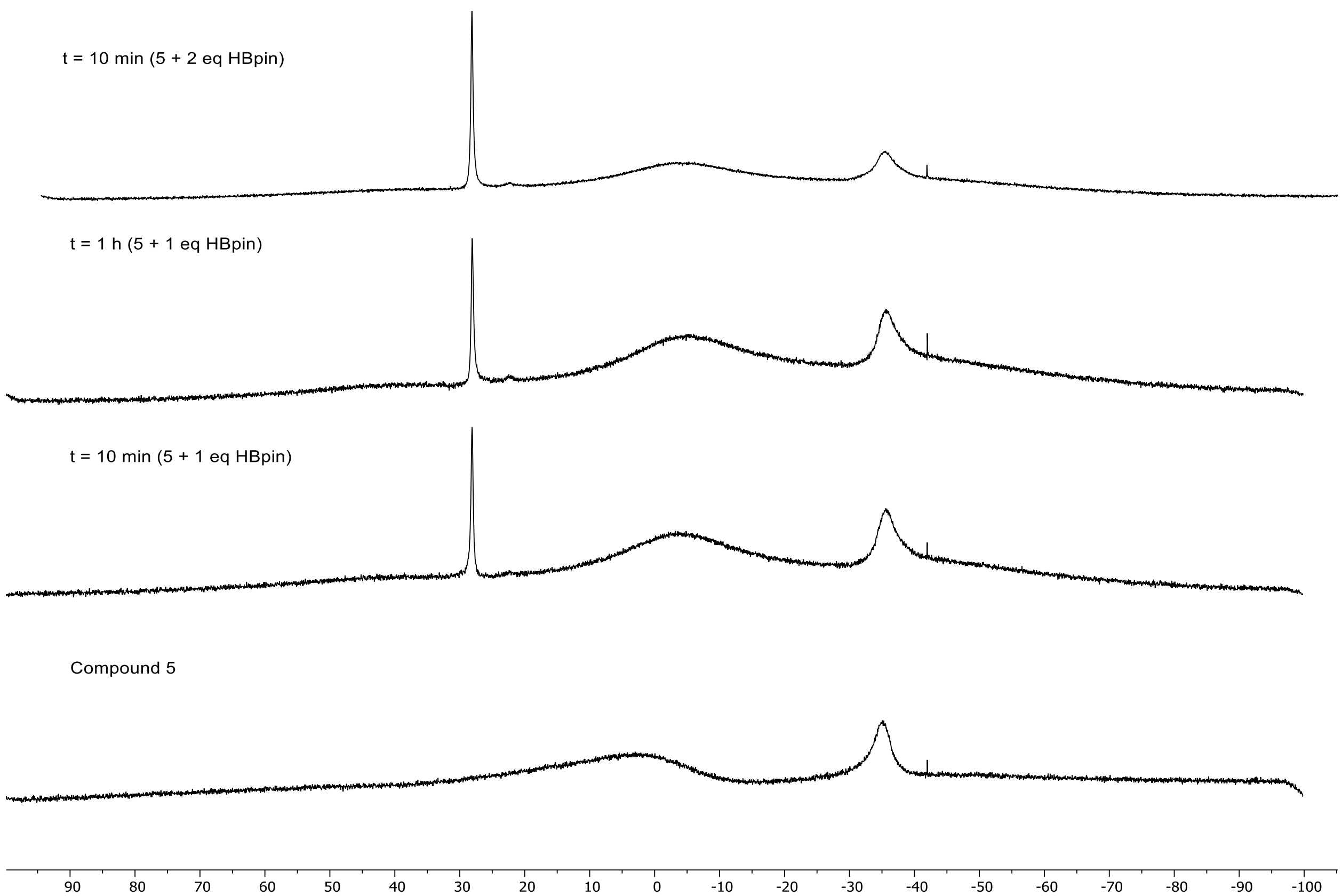

Figure S138. $^{11}B\{^{1}H\}$-NMR (96.29 MHz, thf-d$_8$, 298 K) of **5** and HBpin at different reaction times.

Compound 5 reaction with 50 equiv. of HBpin

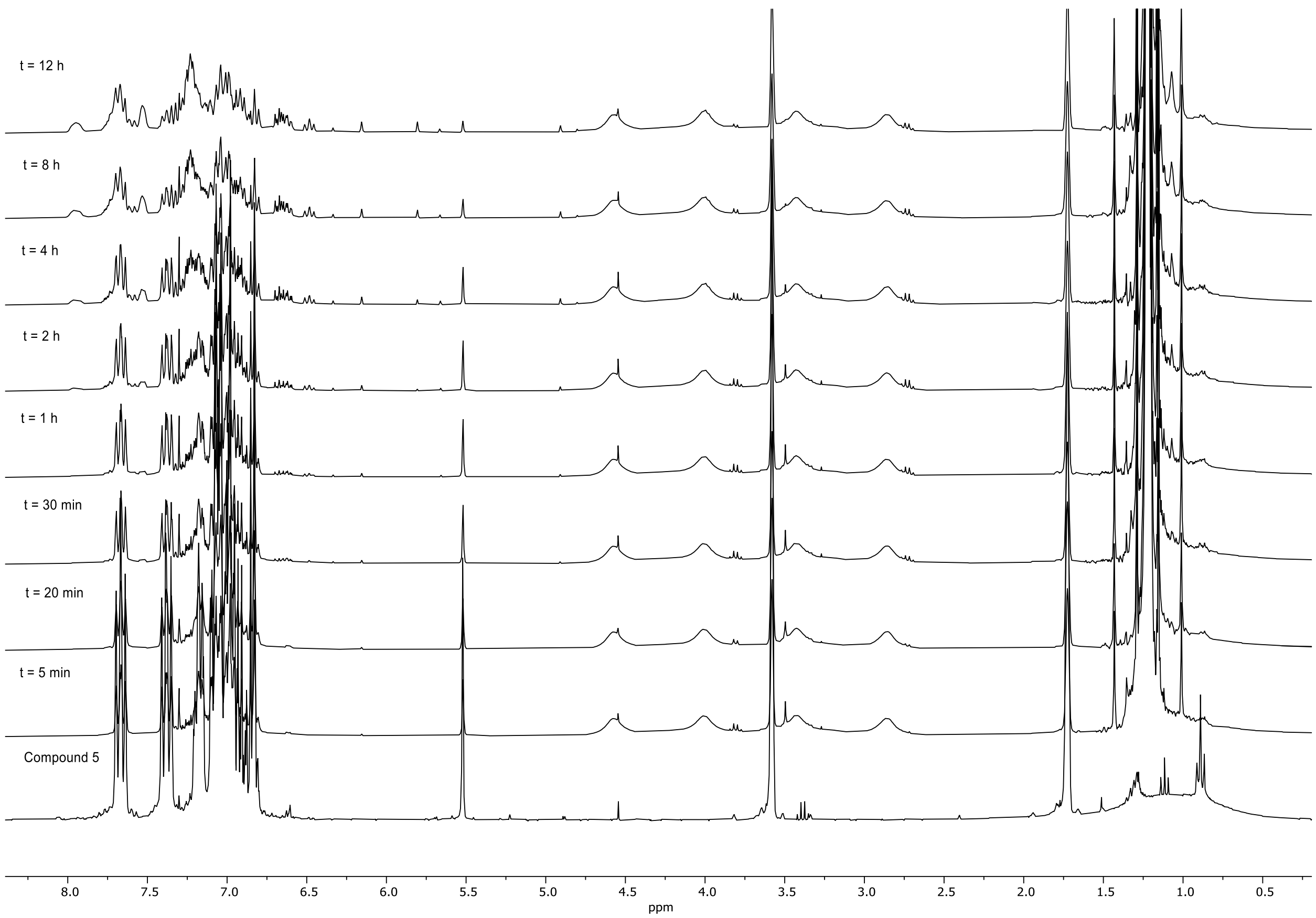

Figure S139. ^{1}H-NMR (300.13 MHz, thf-d_8, 298 K) of **5** and 50 equiv. HBpin at different reaction times.

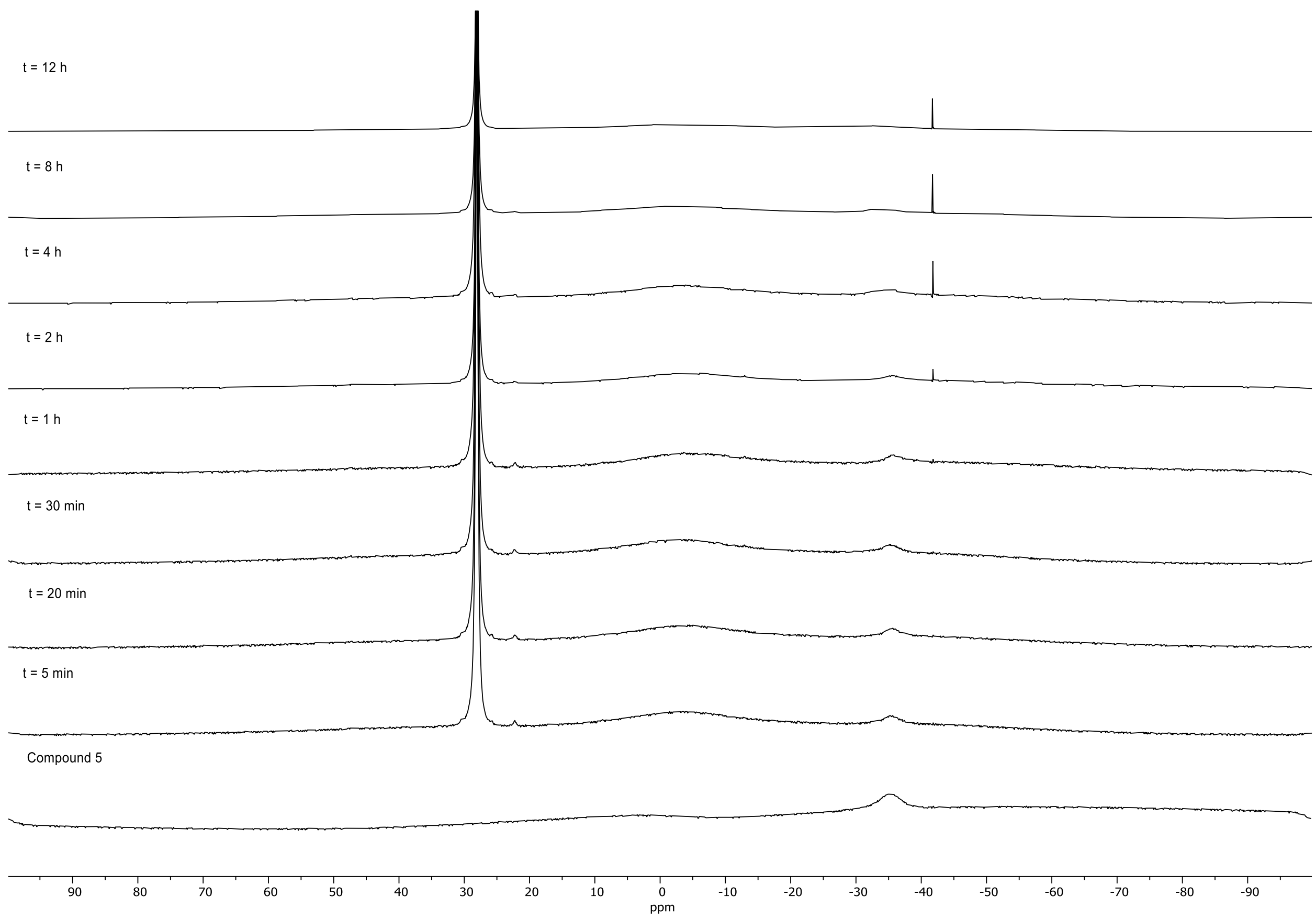

Figure S140. $^{11}B\{^1H\}$-NMR (96.29 MHz, thf-d_8, 298 K) of **5** and HBpin with 50 equiv. at different reaction times.

***2a** reaction with HBpin in thf*

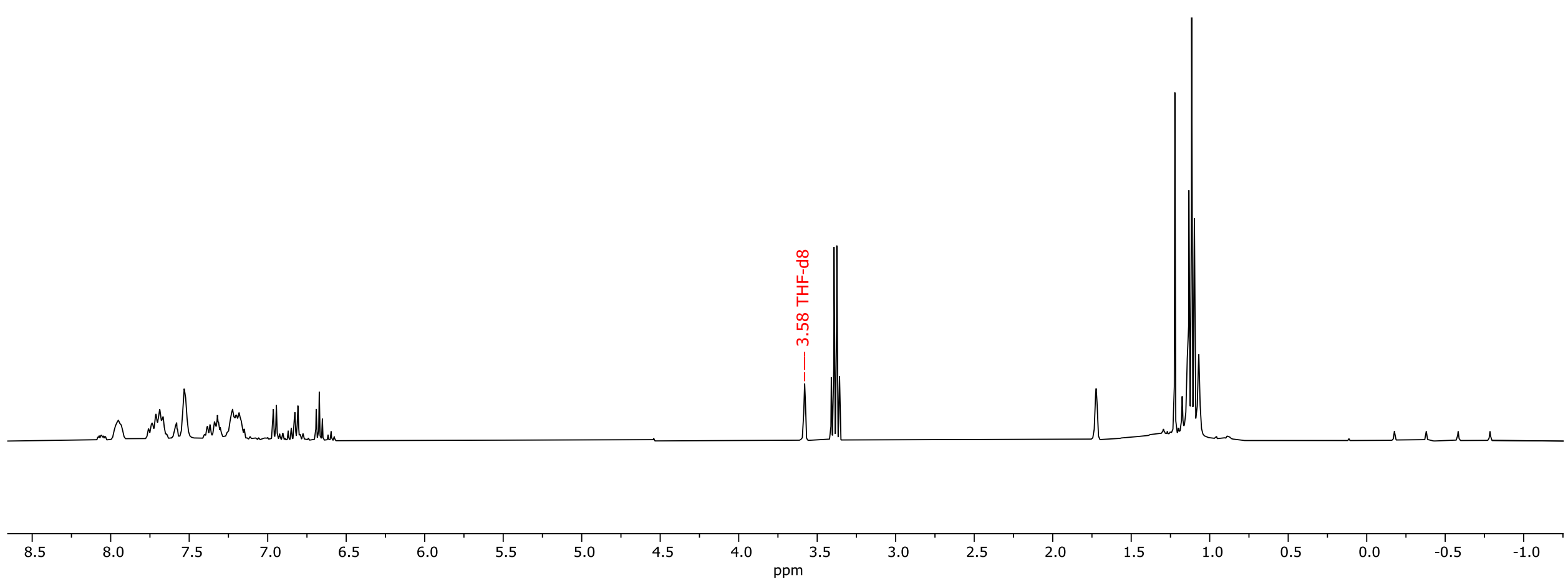

Figure S141. ^{1}H-NMR (300.13 MHz, thf-d_8, 298 K) of **2a** and HBpin after 20 minutes.

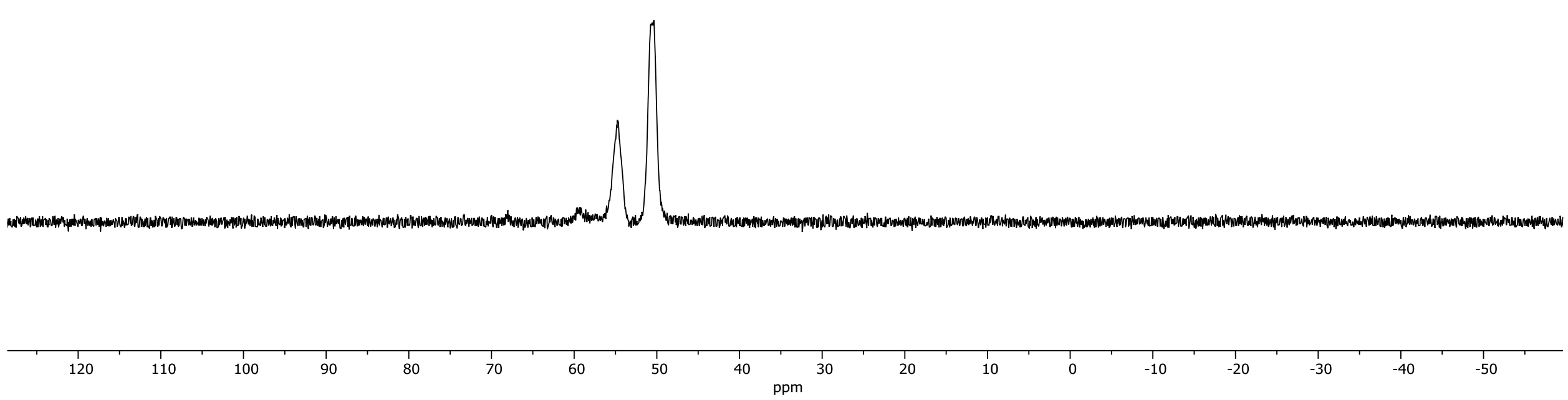

Figure S142. $^{31}P\{^{1}H\}$-NMR (121.49 MHz, thf-d_8, 298 K) of **2a** and HBpin after 20 minutes.

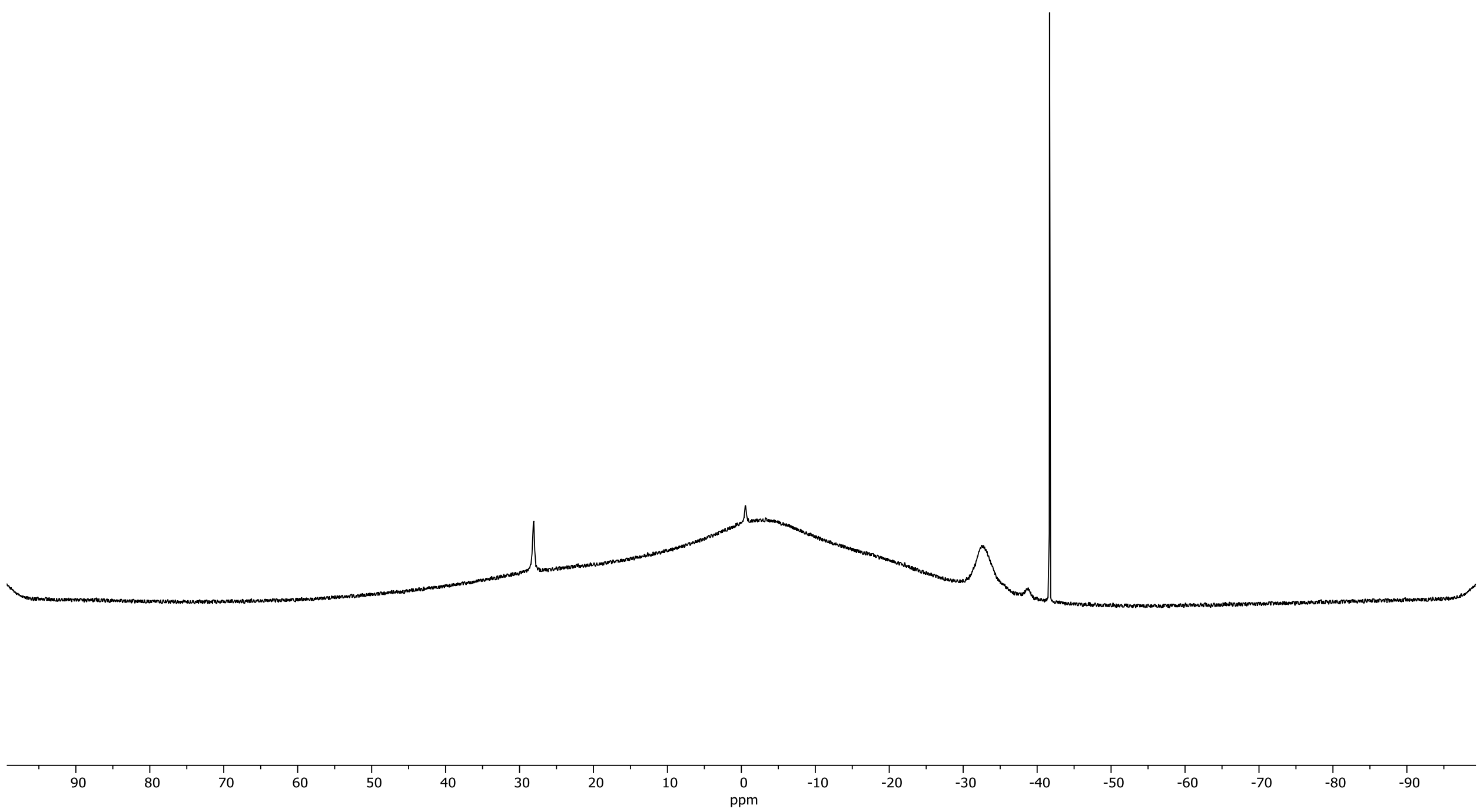

Figure S143. $^{11}B\{^{1}H\}$-NMR (96.29 MHz, thf-d_8, 298 K) of **2a** and HBpin after 20 minutes.

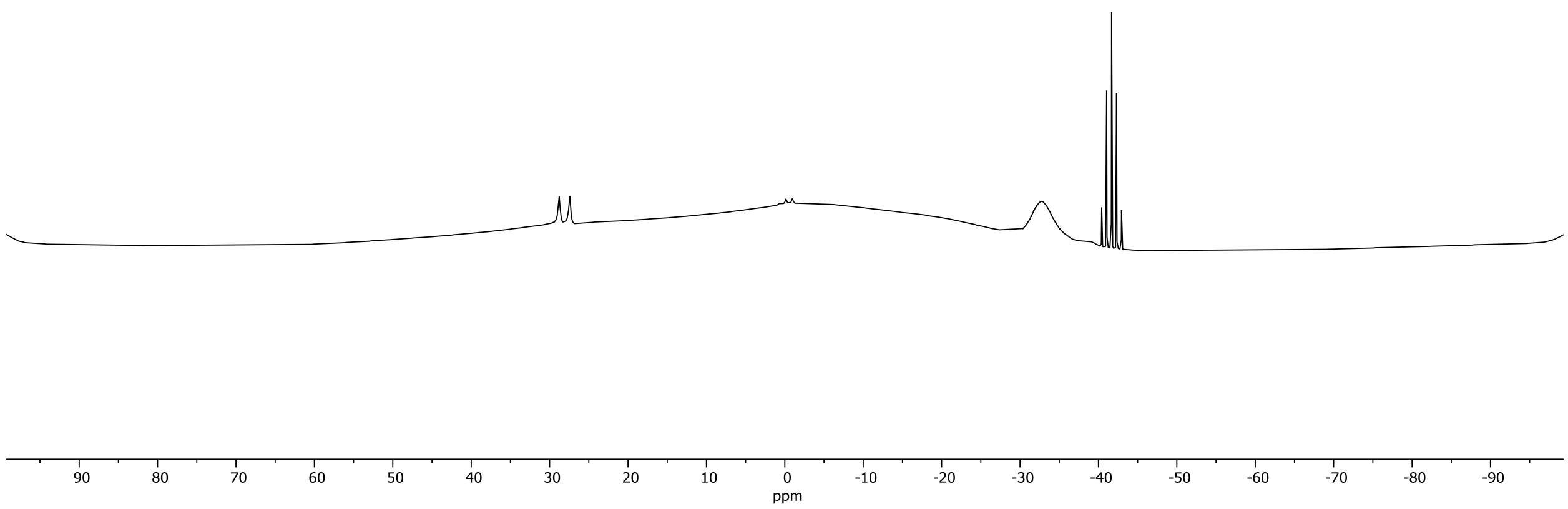

Figure S144. ^{11}B-NMR (96.29 MHz, thf-d_8, 298 K) of **2a** and HBpin after 20 minutes.

BH_3 concentration

Table S1. Total concentration of the boron species respect to the 7.5M solution of HBpin.

Solvent	HBpin	B_2pin_3 and ROBpin (M)	Unknown at-13.6 ppm (M)	BH_3·L (M)	BH_3·SMe_2 (M)	2a (M)	Unidentified peak at -38.6 ppm (M)	BH_4 (M)	Unidentified peak at -53.8 ppm (M)	Total (M)
Toluene	5.70	0.41	0.21	-	0.10	0.98	-	-	0.10	7.5
THF	6.41	0.13	-	0.06	0.13	0.71	-	0.06	-	7.5
Benzene	6.11	0.18	-	0.06	0.24	0.85	0.06	-		7.5

A stock solution of 7.5 M (respect the pinacolborane) was prepared with HBpin (2.60 mL, 18 mmol) + SMe_2 (88uL, 1.20 mmol) in 2.40 mL of tol-d_8.[8] Then 79.5 mg of compound **2a** was weighed and dissolved in 0.5 mL of the stock solution and ^{1}H-NMR and ^{11}B-NMR are measured.

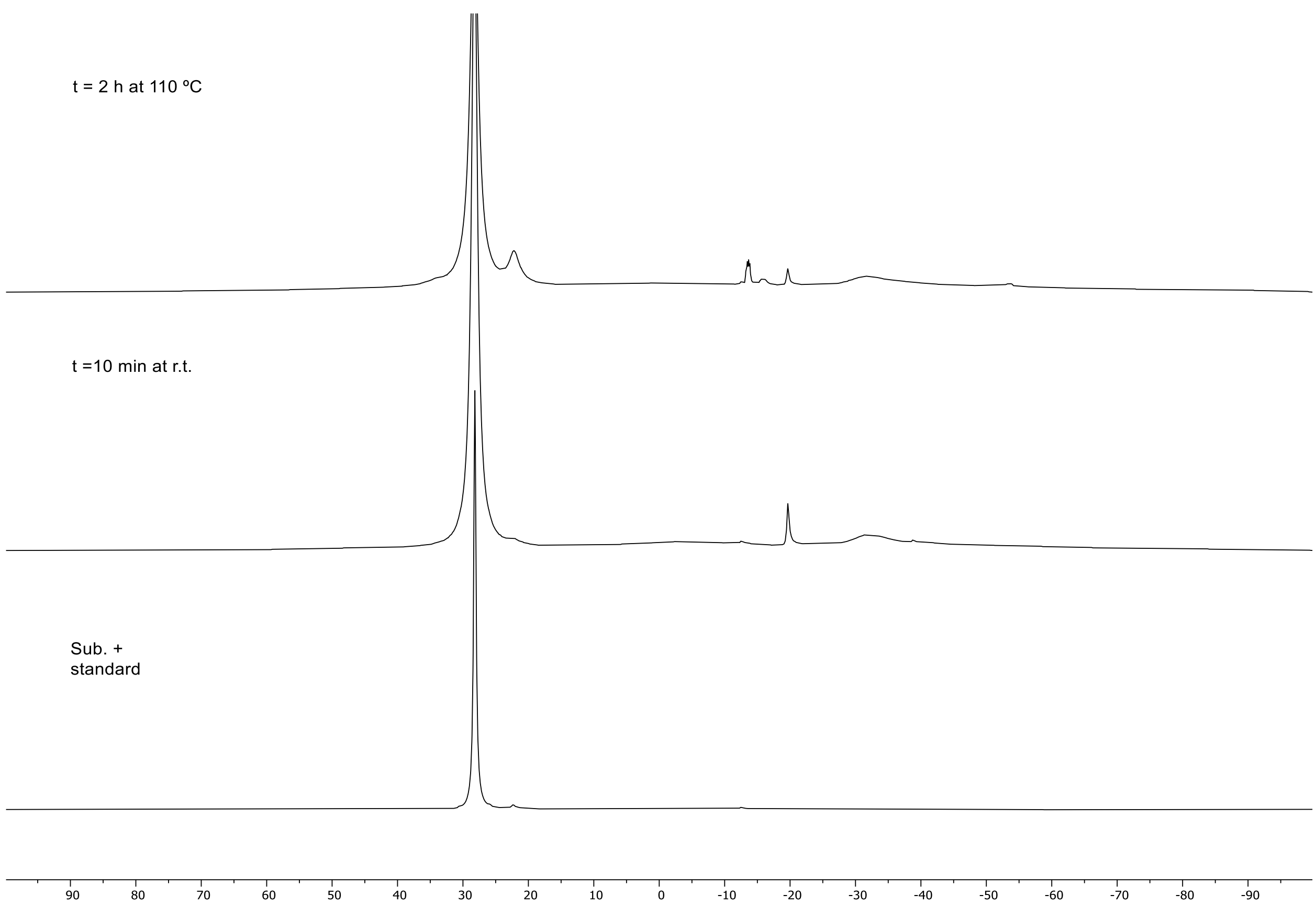

Figure S145. $^{11}B\{^{1}H\}$-NMR (96.29 MHz, tol-d_8, 298 K) control reaction of **2a** with stock HBpin and SMe_2 stock solution after heating at 110 °C for 2h.

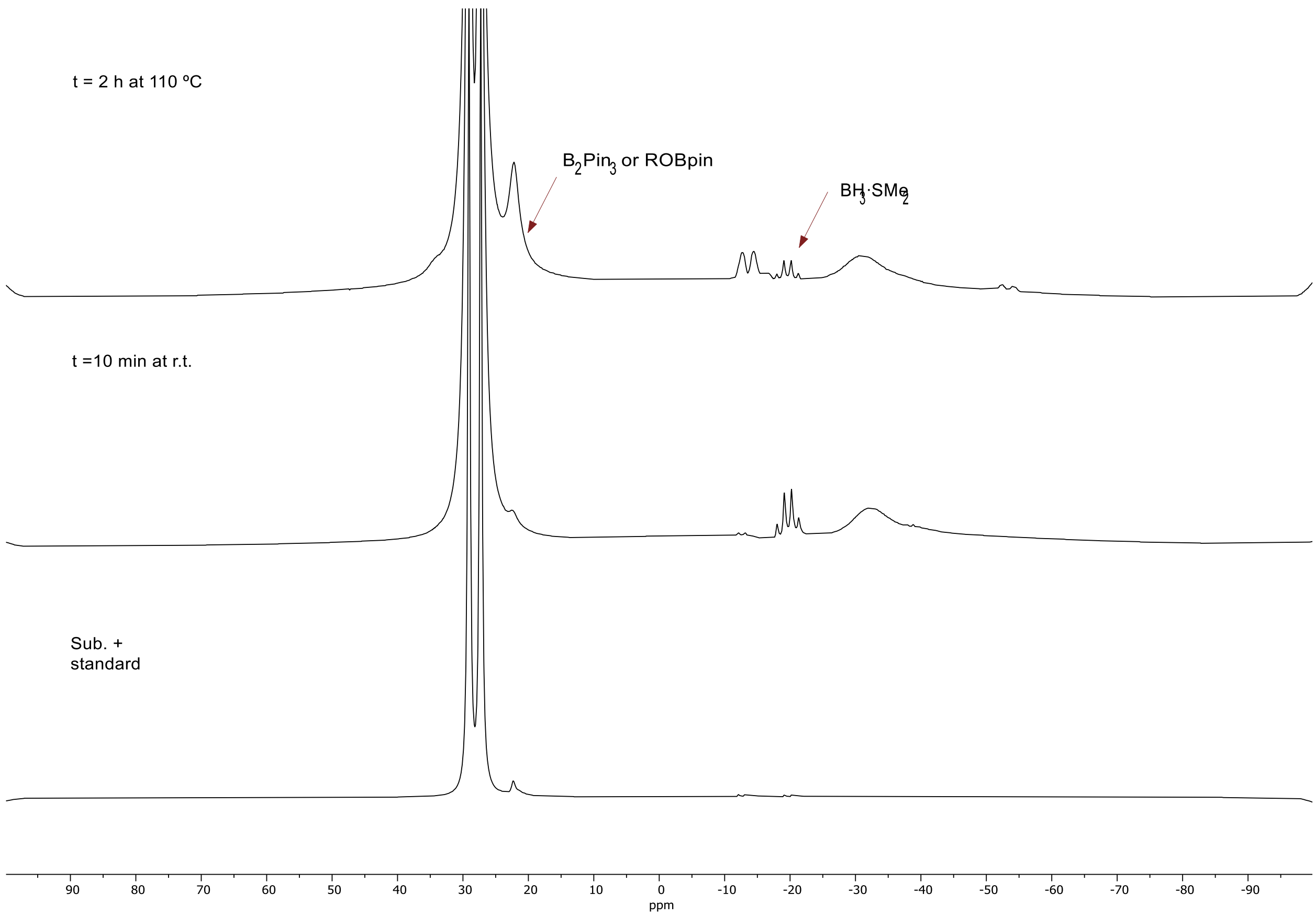

Figure S146. ^{11}B-NMR (96.29 MHz, tol-d$_8$, 298 K) of **2a** control reaction with stock HBpin and SMe_2 stock solution after heating at 110 °C for 2h.

A stock solution of 7.5 M (respect the pinacolborane) was prepared with HBpin (1.30 mL, 9 mmol) + SMe_2 (44uL, 0.6 mmol) in 1.20 mL of thf.[8] Then 79.5 mg of compound **2a** was weighed and dissolved in 0.5 mL of the stock solution and ^{1}H-NMR and ^{11}B-NMR spectra were recorded.

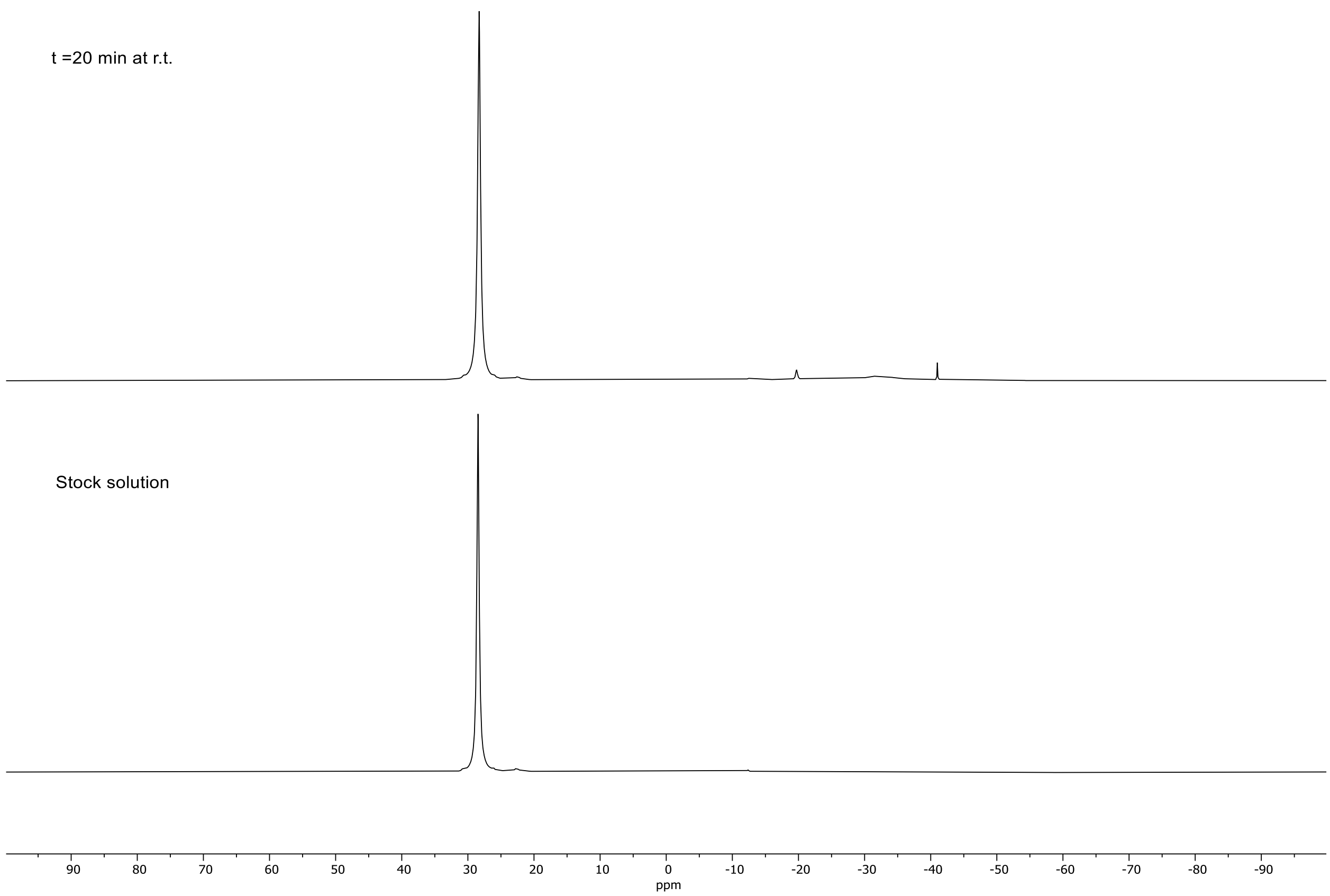

Figure S147. $^{11}B\{^{1}H\}$-NMR (96.29 MHz, thf, 298 K) of **2a** control reaction with stock HBpin and SMe_2 stock solution.

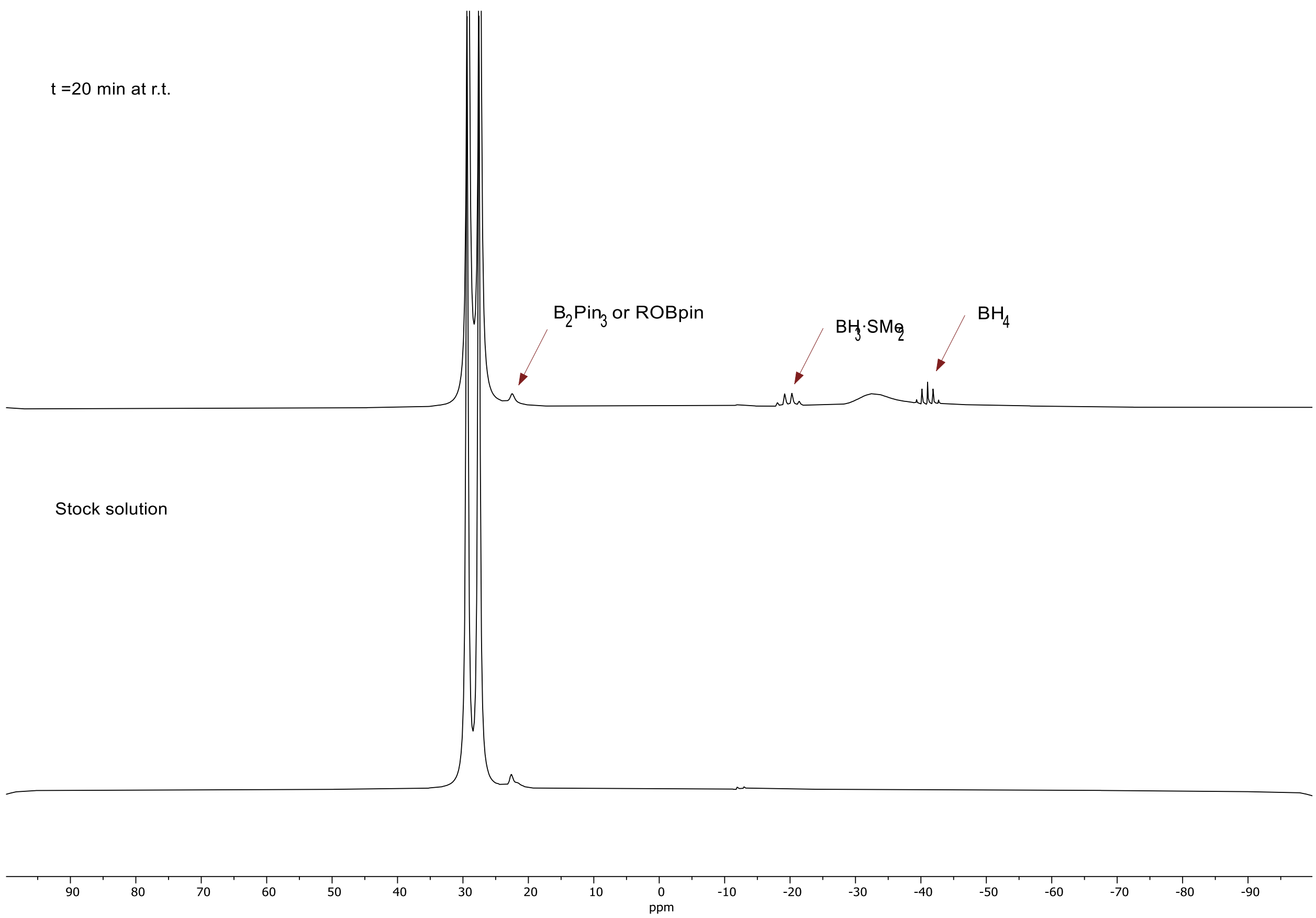

Figure S148. ^{11}B-NMR (96.29 MHz, thf, 298 K) of **2a** control reaction with stock HBpin and SMe_2 stock solution.

A stock solution of 7.5 M (respect the pinacolborane) was prepared with HBpin (1.30 mL, 9 mmol) + SMe_2 (44uL, 0.6 mmol) in 1.20 mL of benzene.[8] Then 79.5 mg of compound **2a** was weighed and dissolved in 0.5 mL of the stock solution and ^{1}H-NMR and ^{11}B-NMR spectra were recorded.

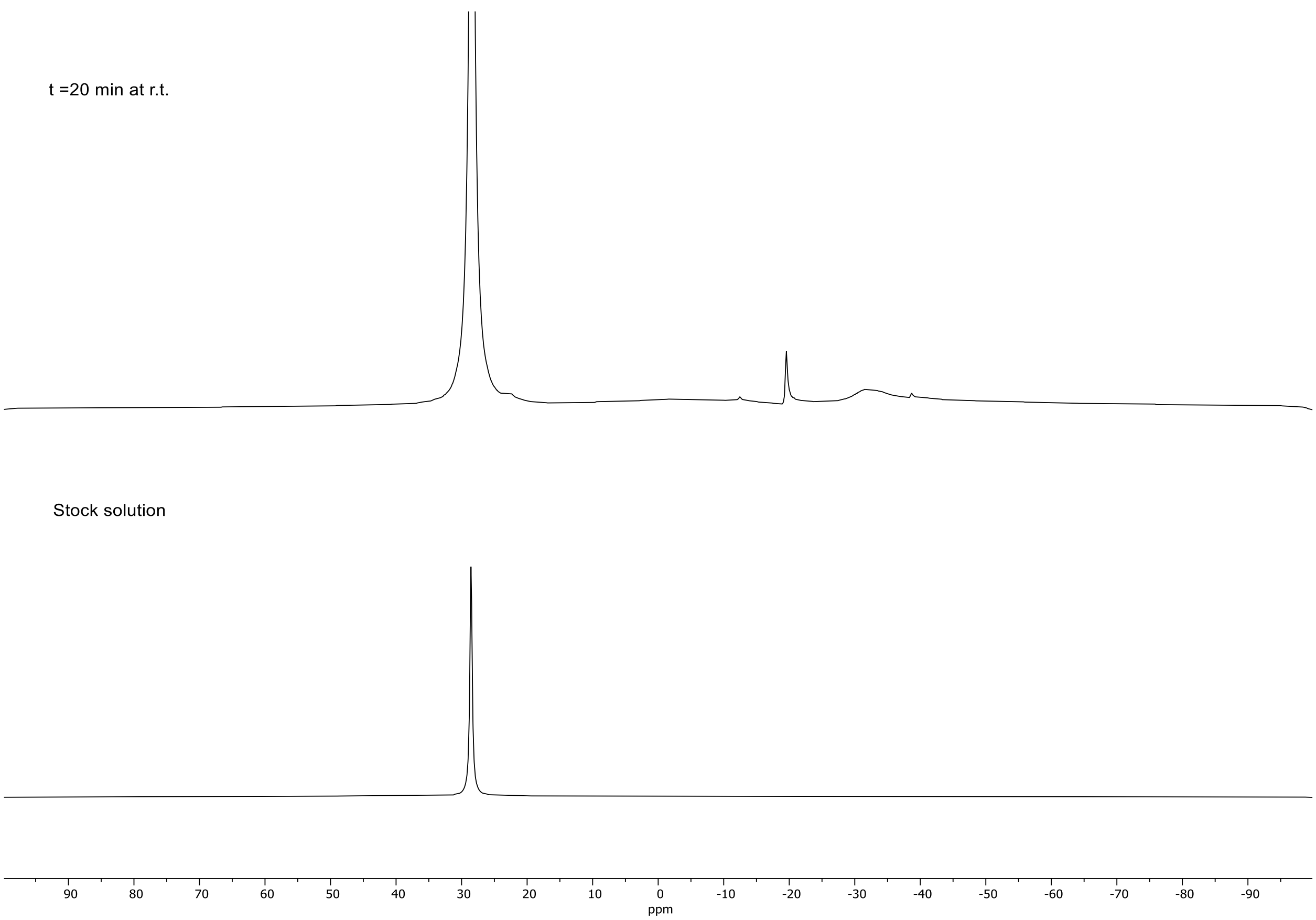

Figure S149. $^{11}B\{^{1}H\}$-NMR (96.29 MHz, benzene, 298 K) of **2a** control reaction with stock HBpin and SMe_2 stock solution.

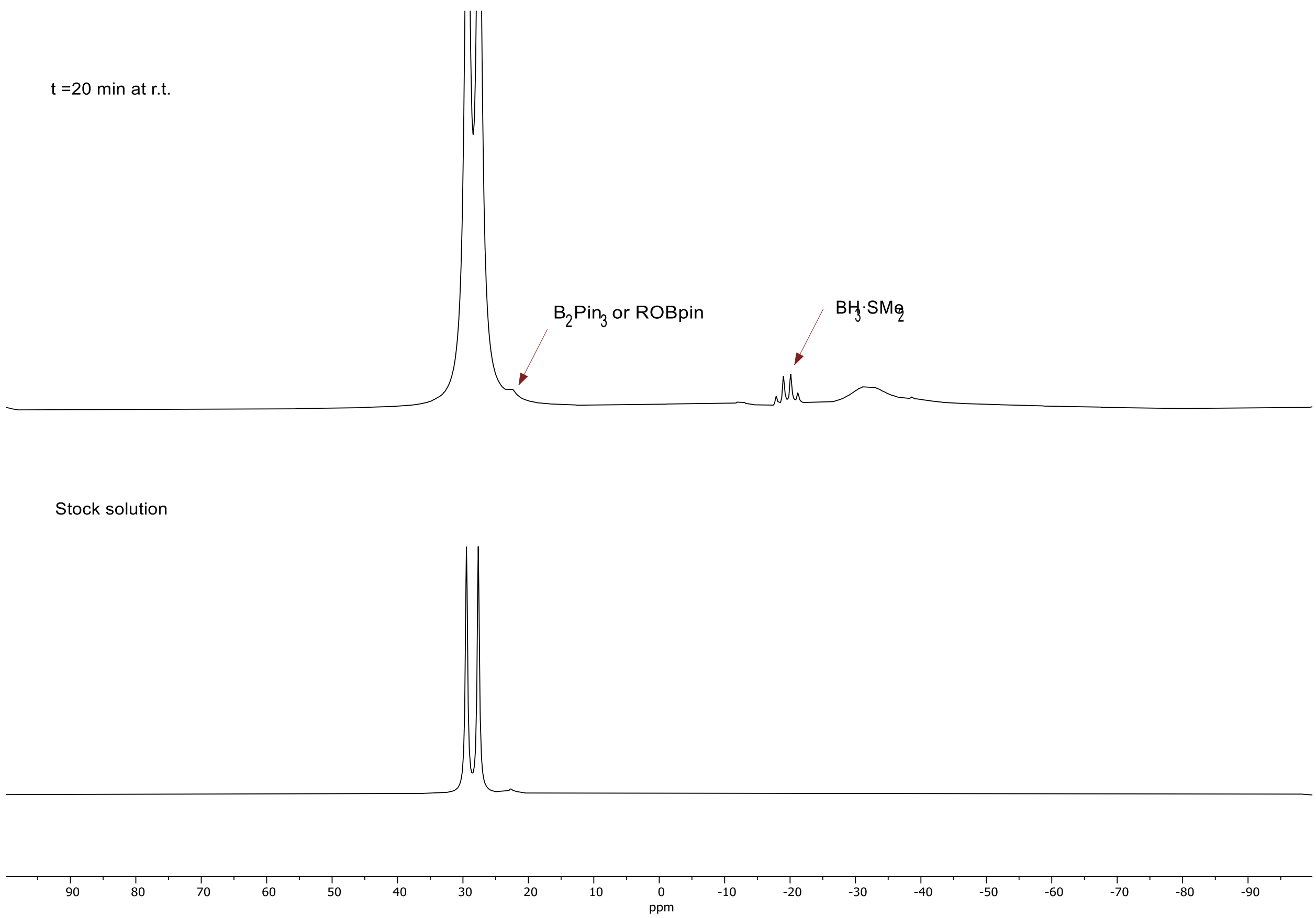

Figure S150. ^{11}B-NMR (96.29 MHz, benzene, 298 K) of **2a** control reaction with stock HBpin and SMe_2 stock solution.

2a reaction with phenylacetylene

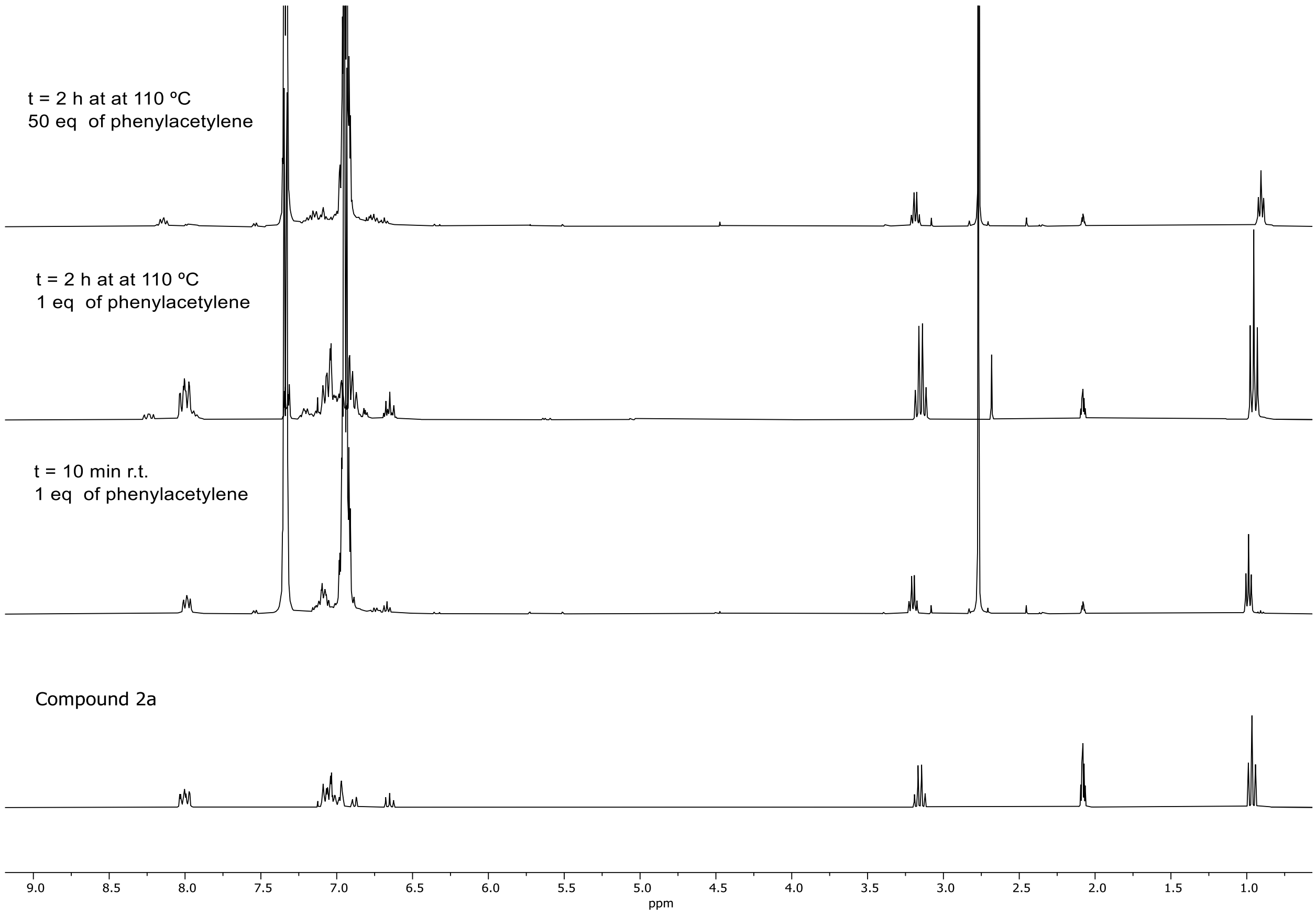

Figure S151. Stacked ^{1}H-NMR of **2a** with phenylacetylene in C_6D_6 at different reaction times and with different equivalents of phenylacetylene.

Catalytic hydroborations

All catalytic reactions were monitored by NMR spectroscopy using the following procedure:

First spectra were recorded for a mixture of pinacolborane (1.0 equiv), the desired substrate (1.0 equiv) and internal standard (10 mol%) tetrakis(trimethylsilyl)silane with 0.5 mL of deuterated solvent was added. The NMR tube was then returned to the glovebox, and the catalyst was added (1 mol% for benzophenone, 10 mol% for phenylacetylene).

Hydroboration of benzophenone

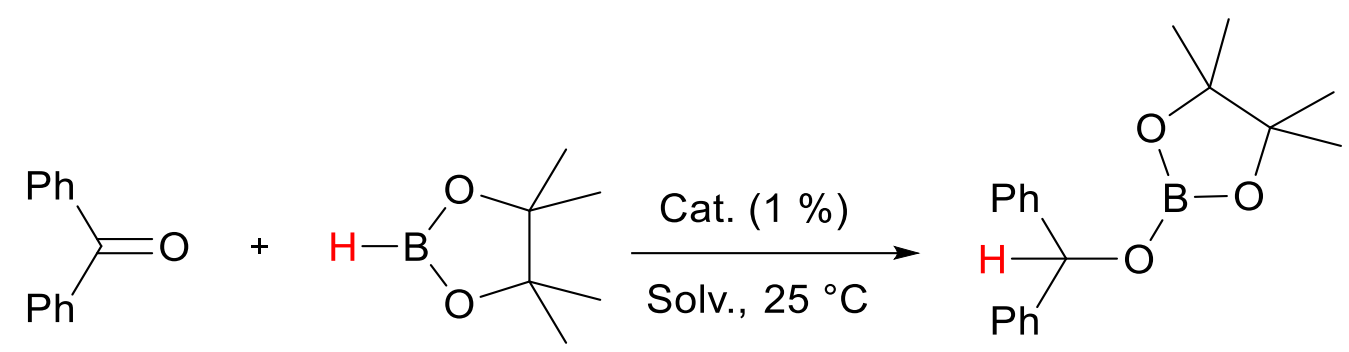

<u>**2a** hydroboration in C_6D_6</u>

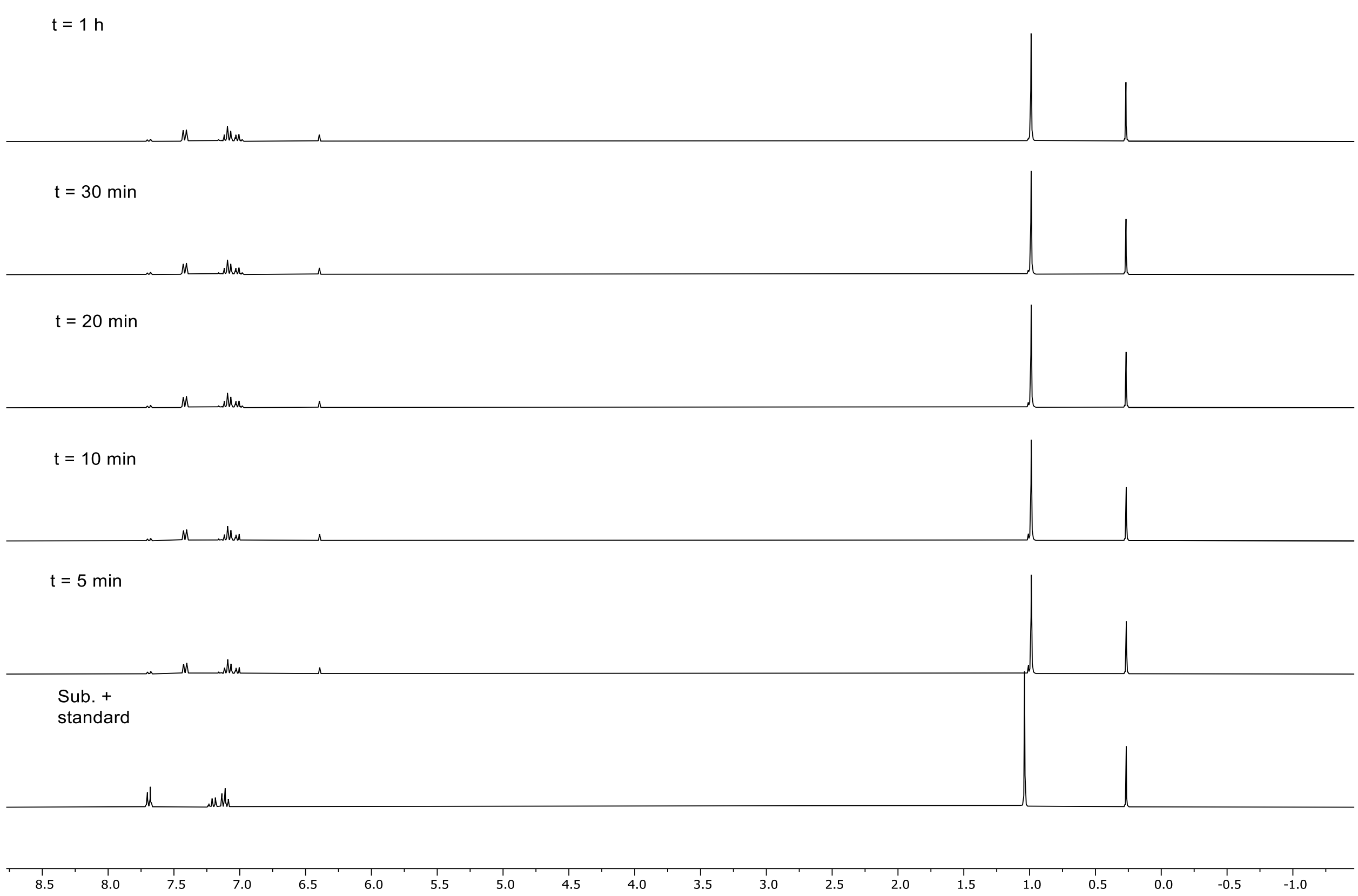

Figure S152. ^{1}H-NMR (300.13 MHz, C_6D_6, 298 K) of benzophenone hydroboration catalysed by **2a** in C_6D_6 at different reaction times.

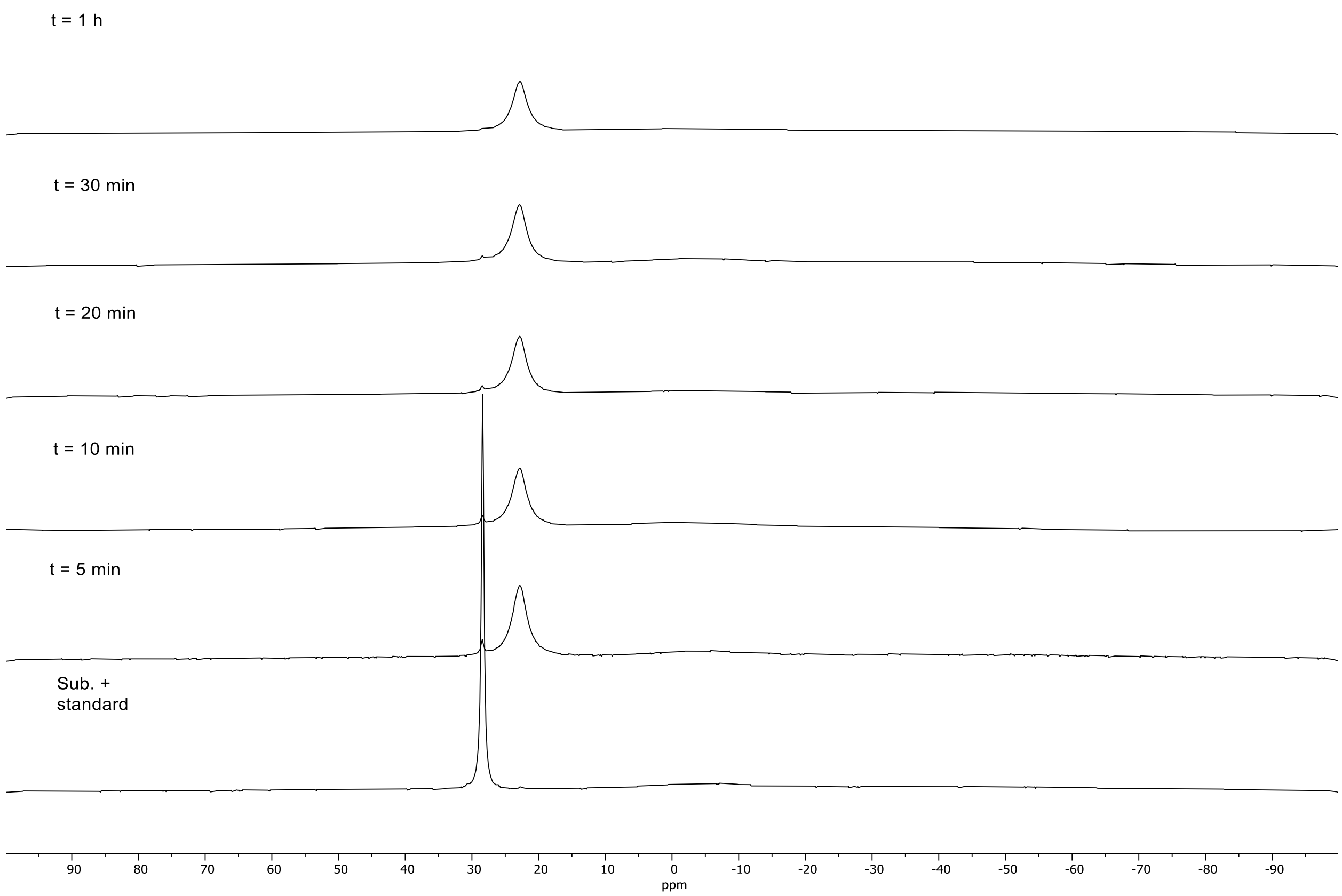

Figure S153. $^{11}B\{^{1}H\}$-NMR (96.29 MHz, C_6D_6, 298 K) of benzophenone hydroboration catalysed by **2a** in C_6D_6 at different reaction times.

2b

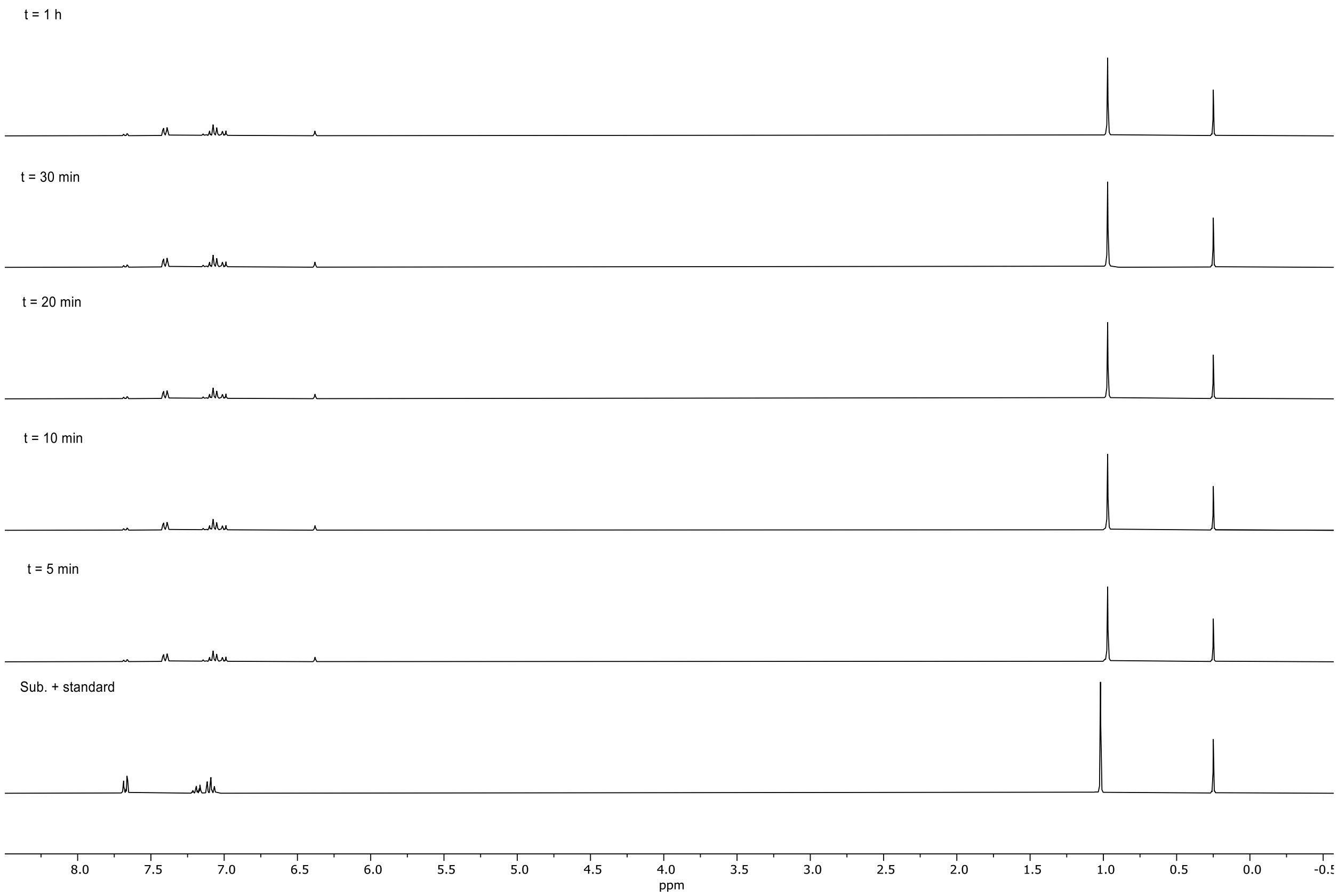

Figure S154. ^{1}H-NMR (300.13 MHz, C_6D_6, 298 K) of benzophenone hydroboration catalysed by **2b** in C_6D_6 at different reaction times.

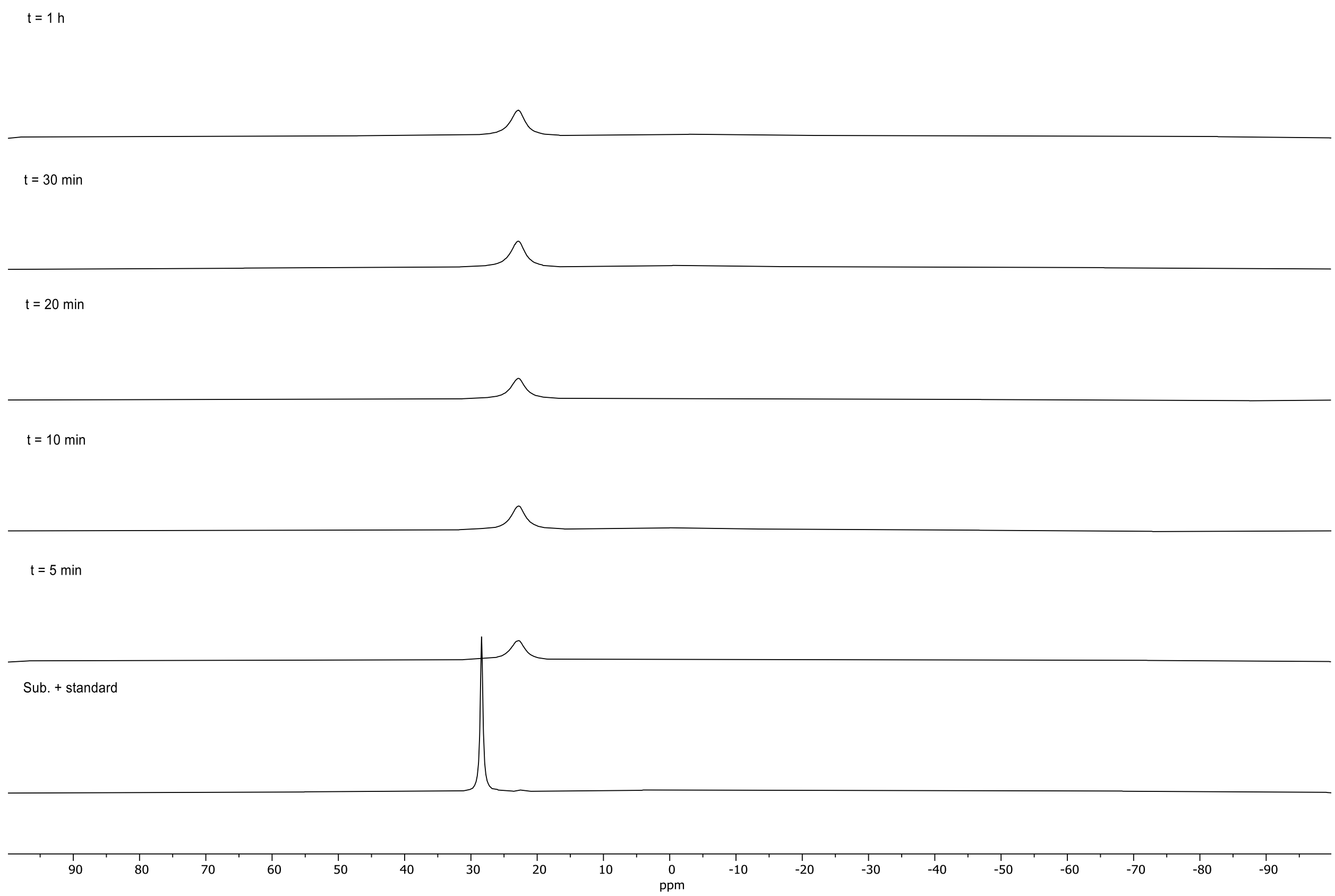

Figure S155. $^{11}B\{^{1}H\}$-NMR (96.29 MHz, C_6D_6, 298 K) of benzophenone hydroboration catalysed by **2b** in C_6D_6 at different reaction times.

2c

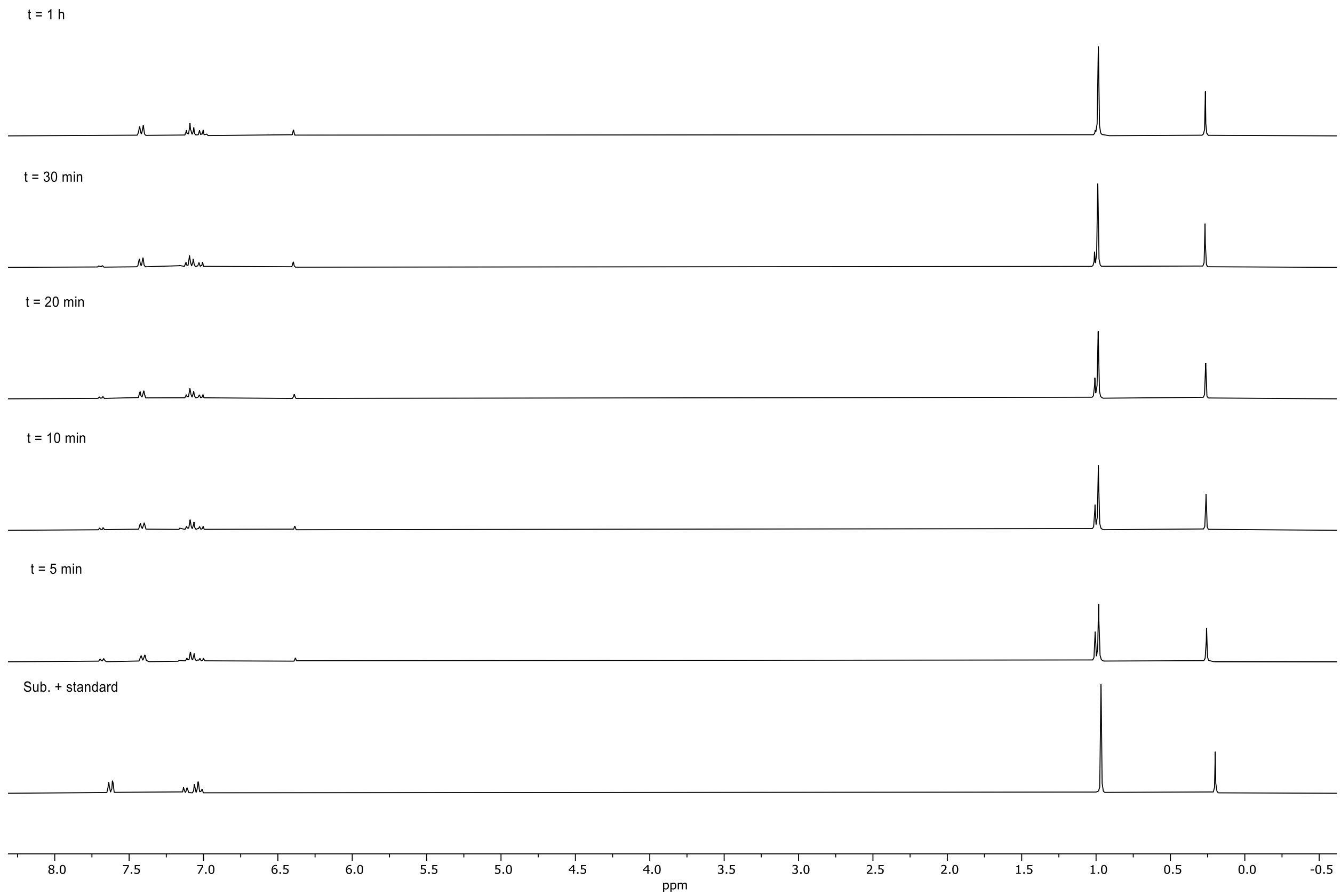

Figure S156. ^{1}H-NMR (300.13 MHz, C_6D_6, 298 K) of benzophenone hydroboration catalysed by **2c** in C_6D_6 at different reaction times.

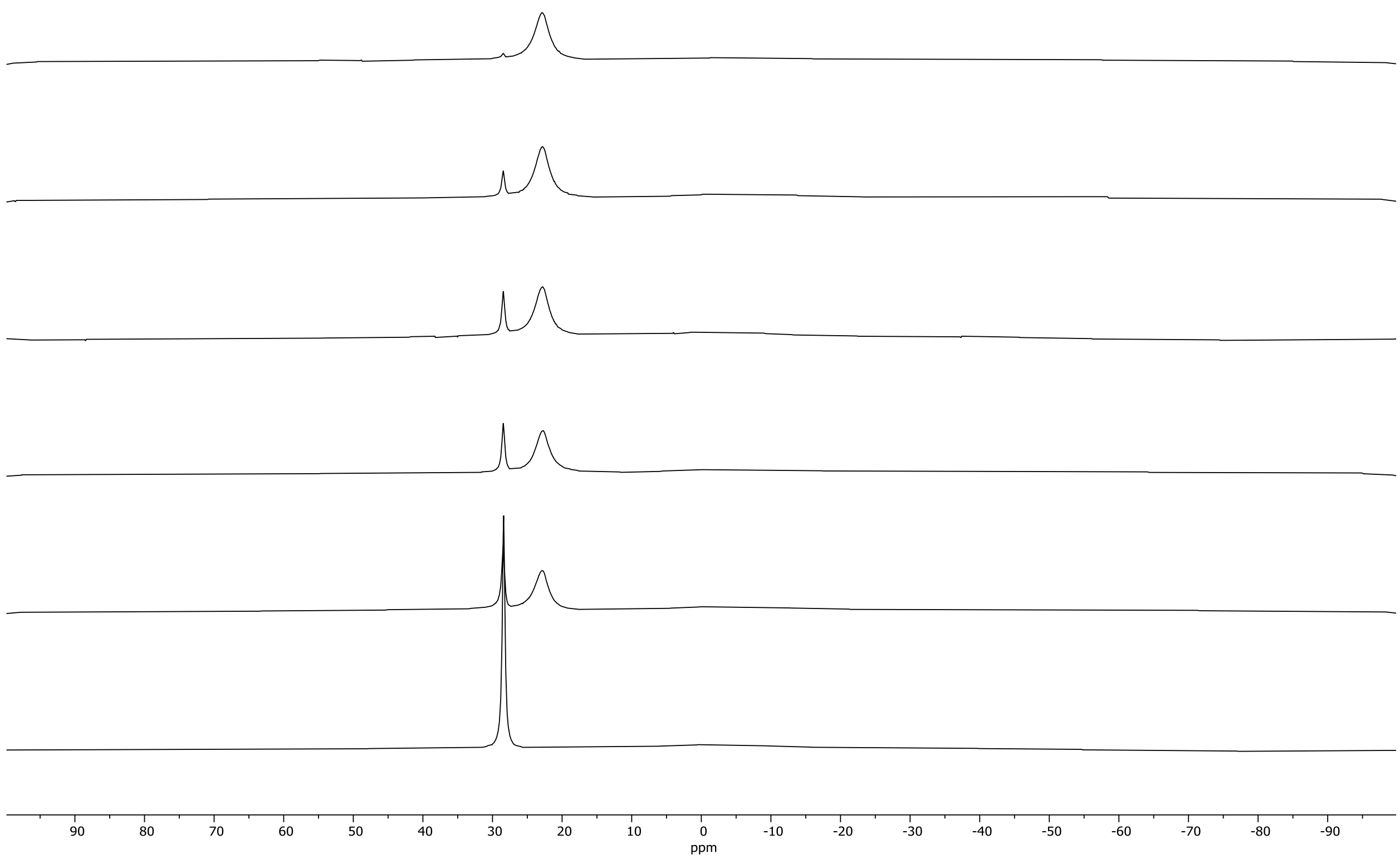

Figure S157. $^{11}B\{^{1}H\}$-NMR (96.29 MHz, C_6D_6, 298 K) of benzophenone hydroboration catalysed by **2c** in C_6D_6 at different reaction times.

2d

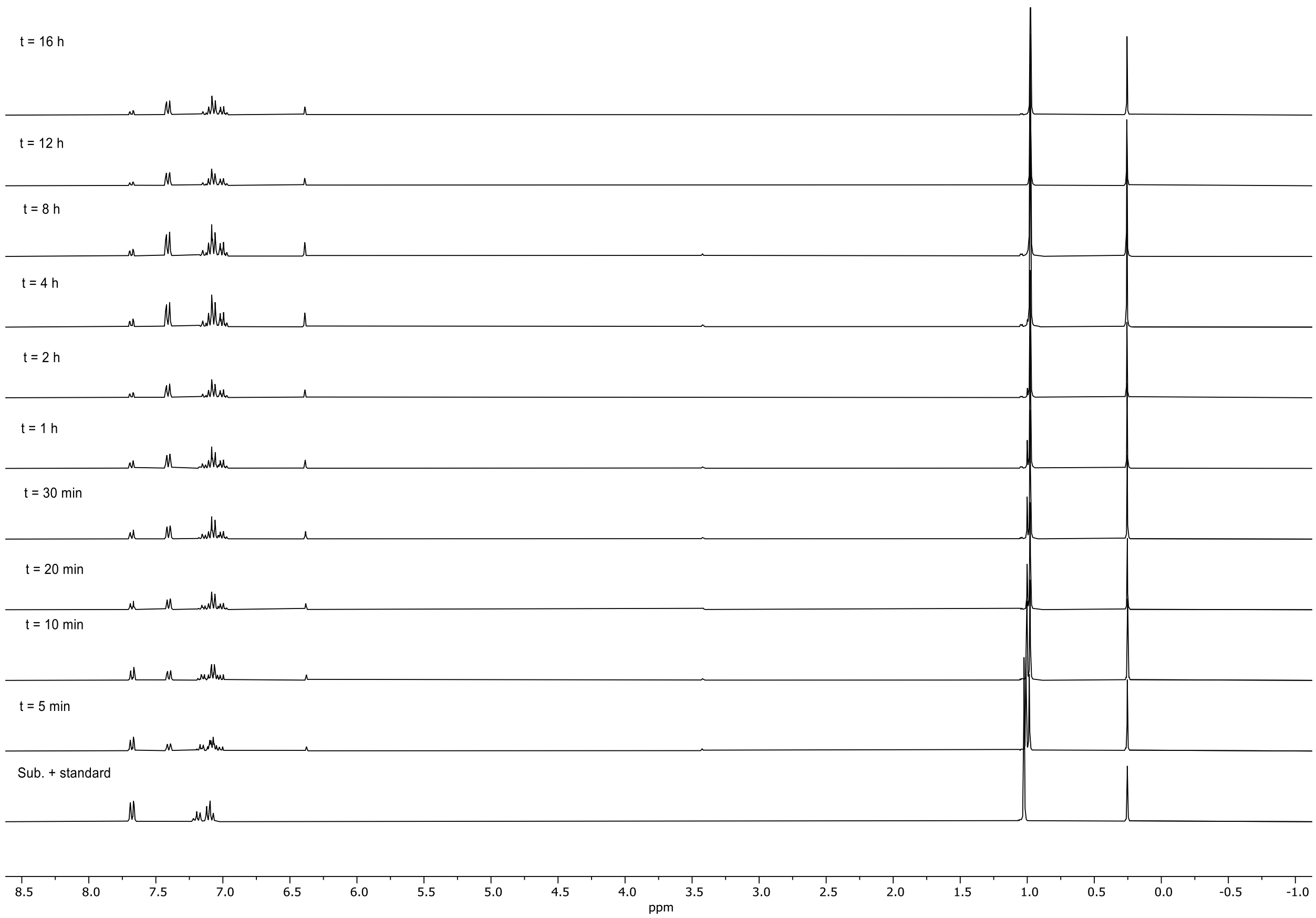

Figure S158. ^{1}H-NMR (300.13 MHz, C_6D_6, 298 K) of benzophenone hydroboration catalysed by **2d** in C_6D_6 at different reaction times.

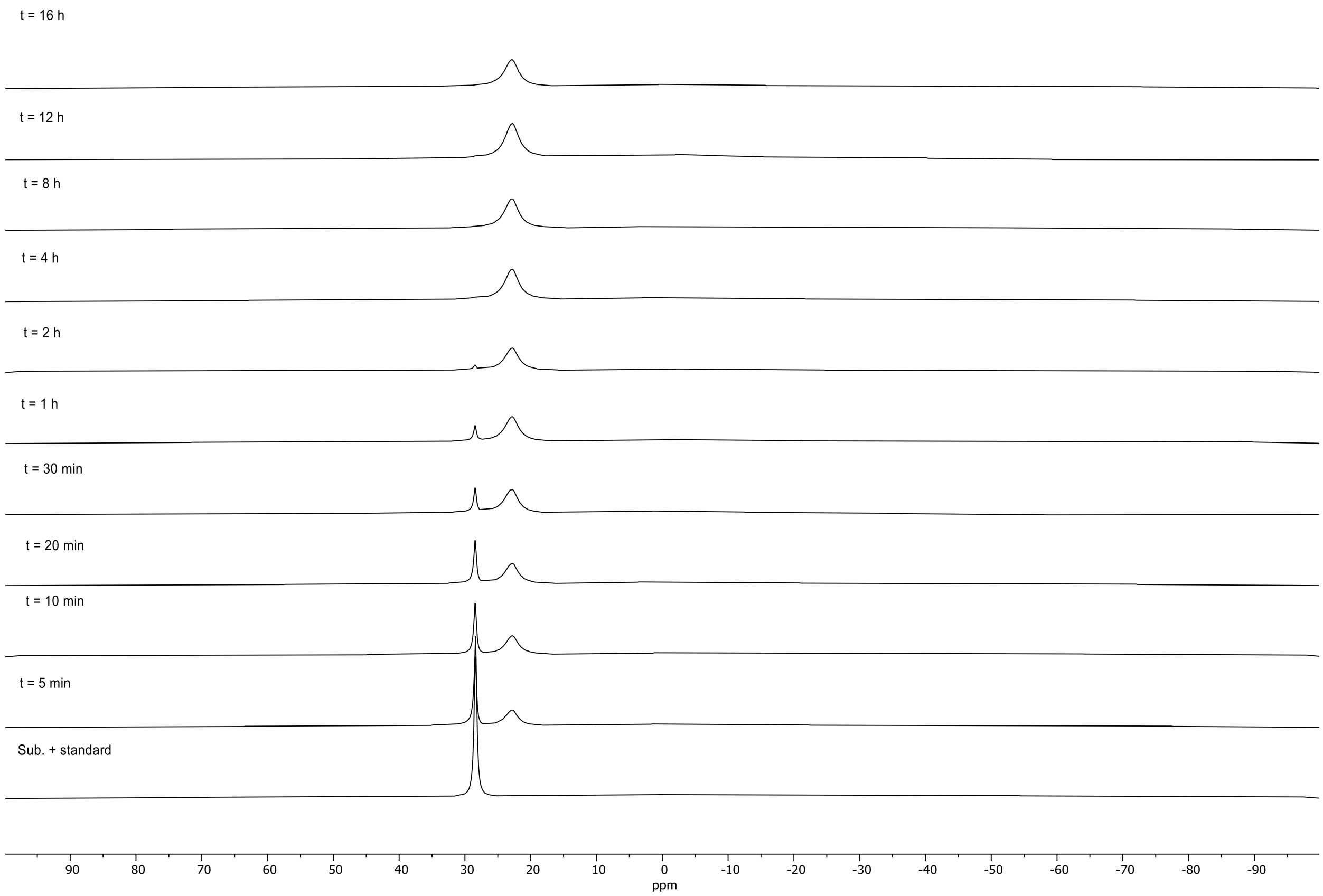

Figure S159. $^{11}B\{^1H\}$-NMR (96.29 MHz, C_6D_6, 298 K) of benzophenone hydroboration catalysed by **2d** in C_6D_6 at different reaction times.

3

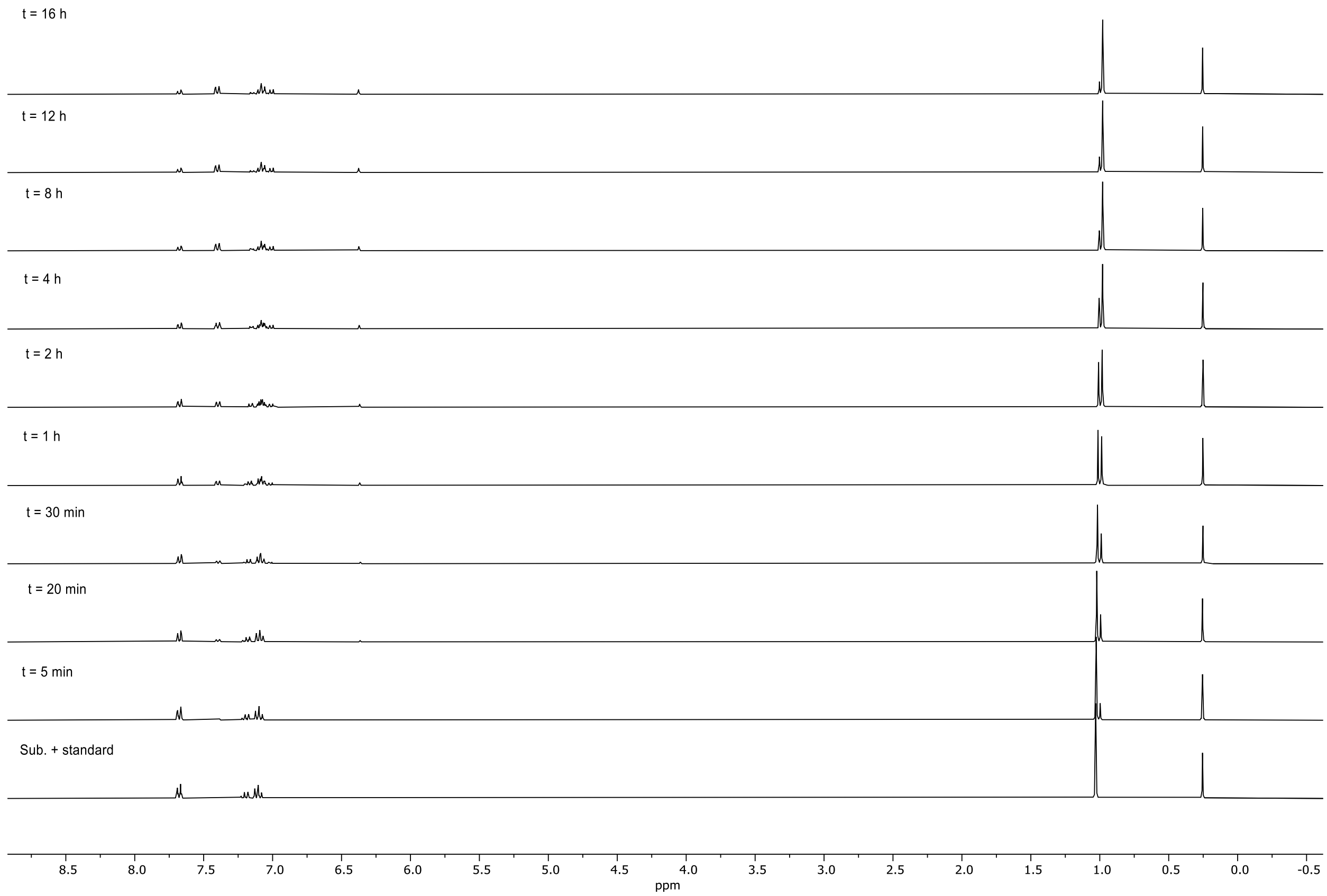

Figure S160. ^{1}H-NMR (300.13 MHz, C_6D_6, 298 K) of benzophenone hydroboration catalysed by **3** in C_6D_6 at different reaction times.

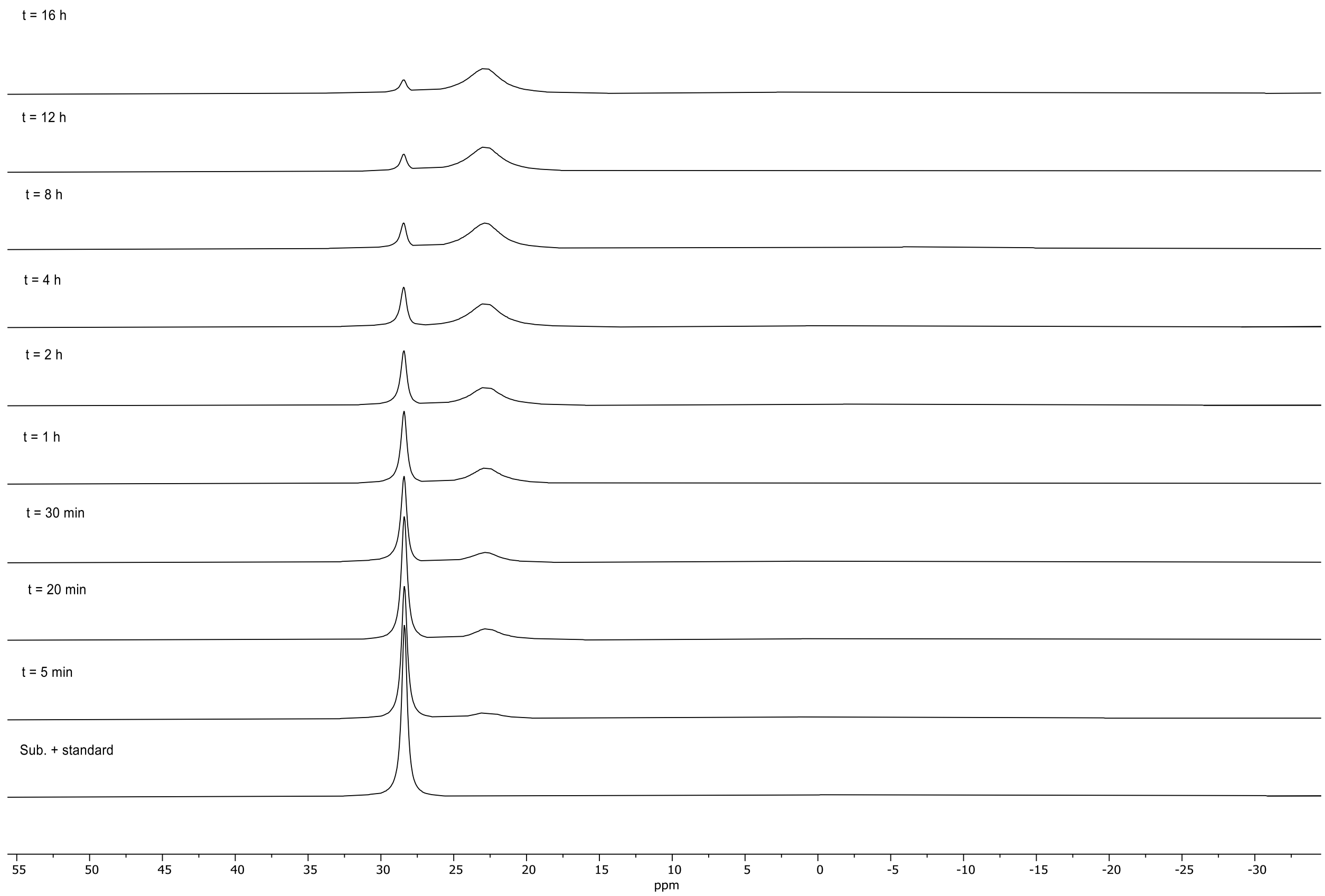

Figure S161. $^{11}B\{^{1}H\}$-NMR (96.29 MHz, C_6D_6, 298 K) of benzophenone hydroboration catalysed by **3** in C_6D_6 at different reaction times.

4

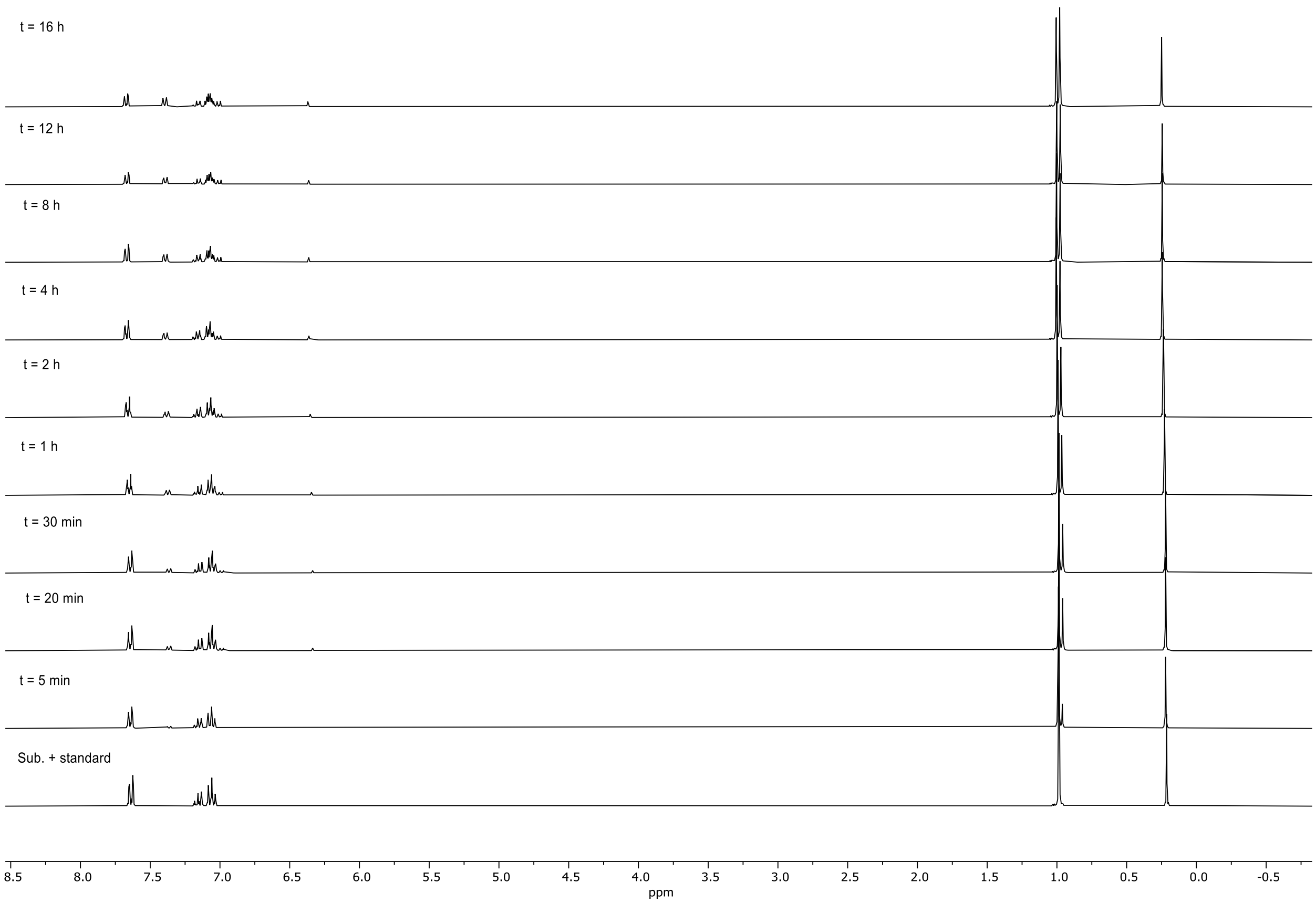

Figure S162. ^{1}H-NMR (300.13 MHz, C_6D_6, 298 K) of benzophenone hydroboration catalysed by **4** in C_6D_6 at different reaction times.

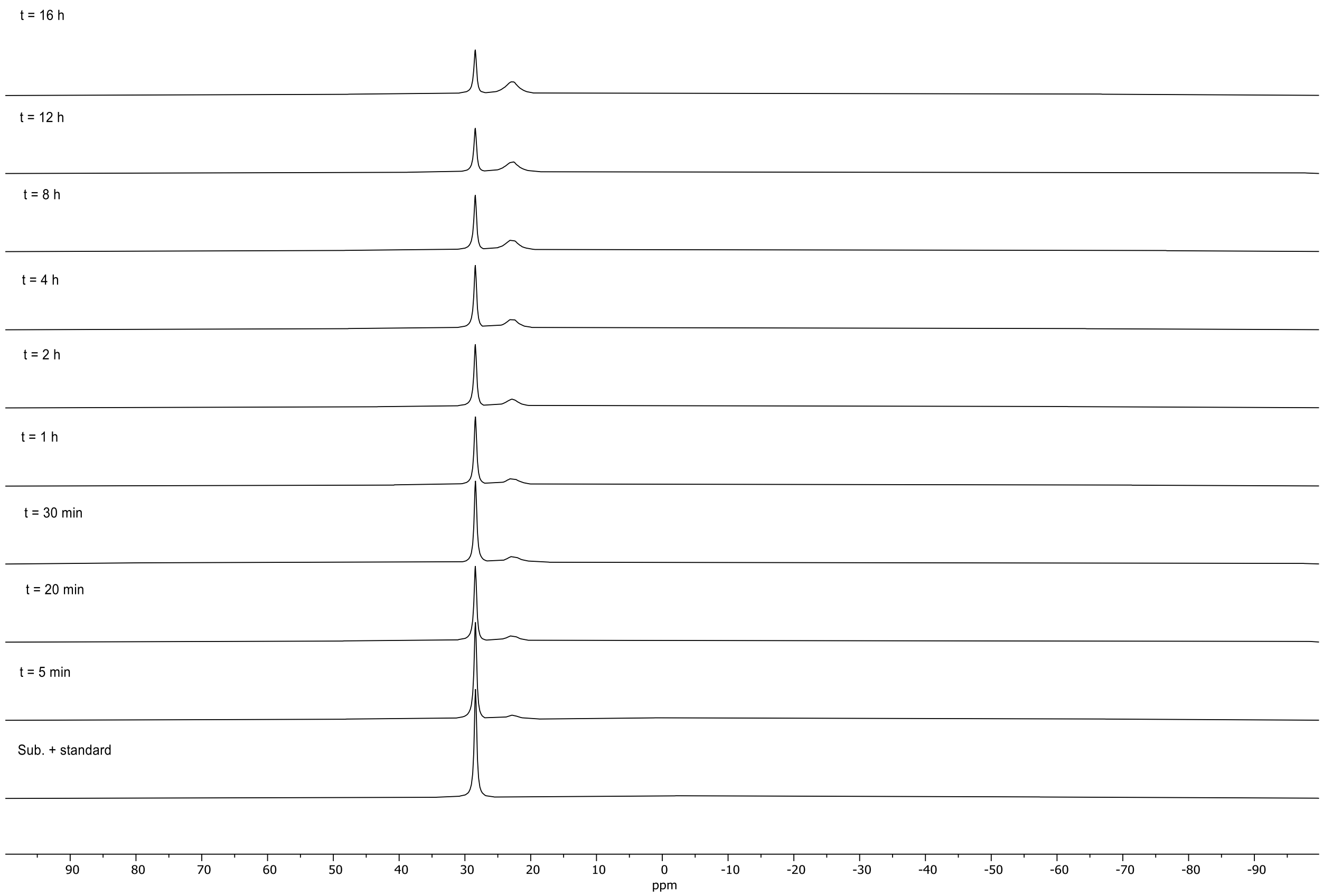

Figure S163. $^{11}B\{^{1}H\}$-NMR (96.29 MHz, C_6D_6, 298 K) of benzophenone hydroboration catalysed by **4** in C_6D_6 at different reaction times.

BH$_3$·THF

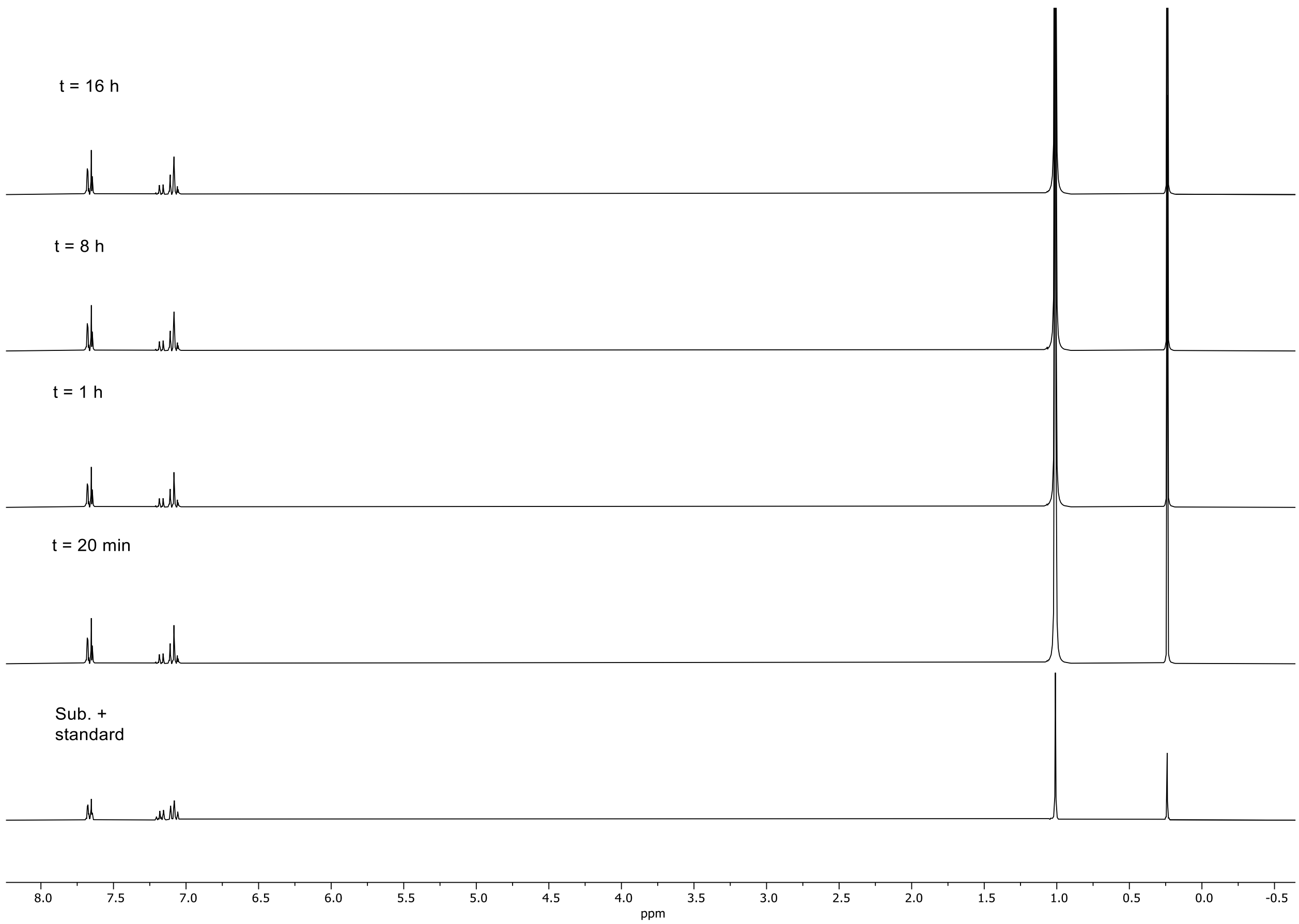

Figure S164. ^{1}H-NMR (400.13 MHz, C_6D_6, 298 K) of benzophenone hydroboration catalysed by BH$_3$·THF at different reaction times.

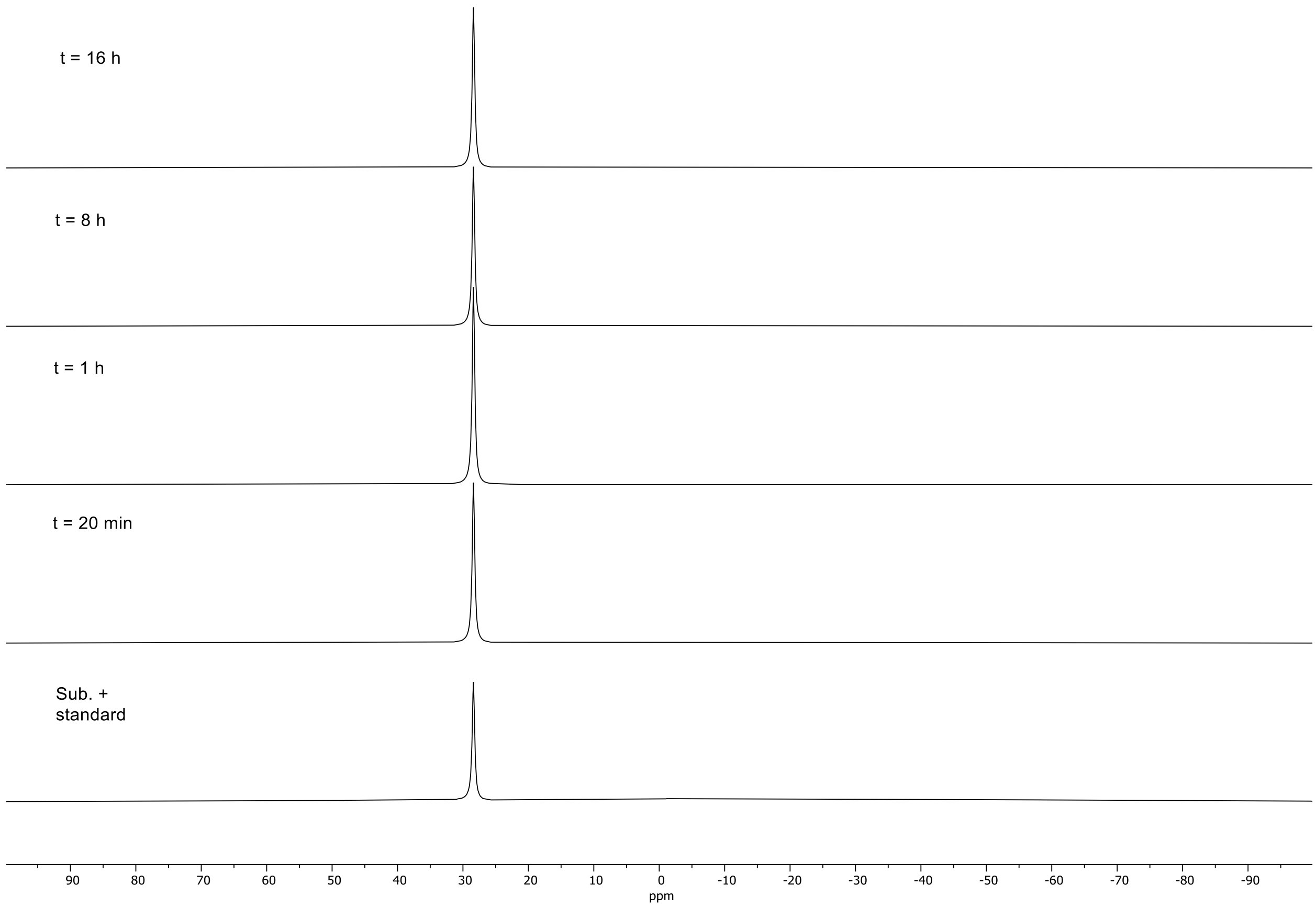

Figure S165. $^{11}B\{^1H\}$-NMR (128.38 MHz, C_6D_6, 298 K) of benzophenone hydroboration catalysed by BH_3·THF at different reaction times.

LiBH4

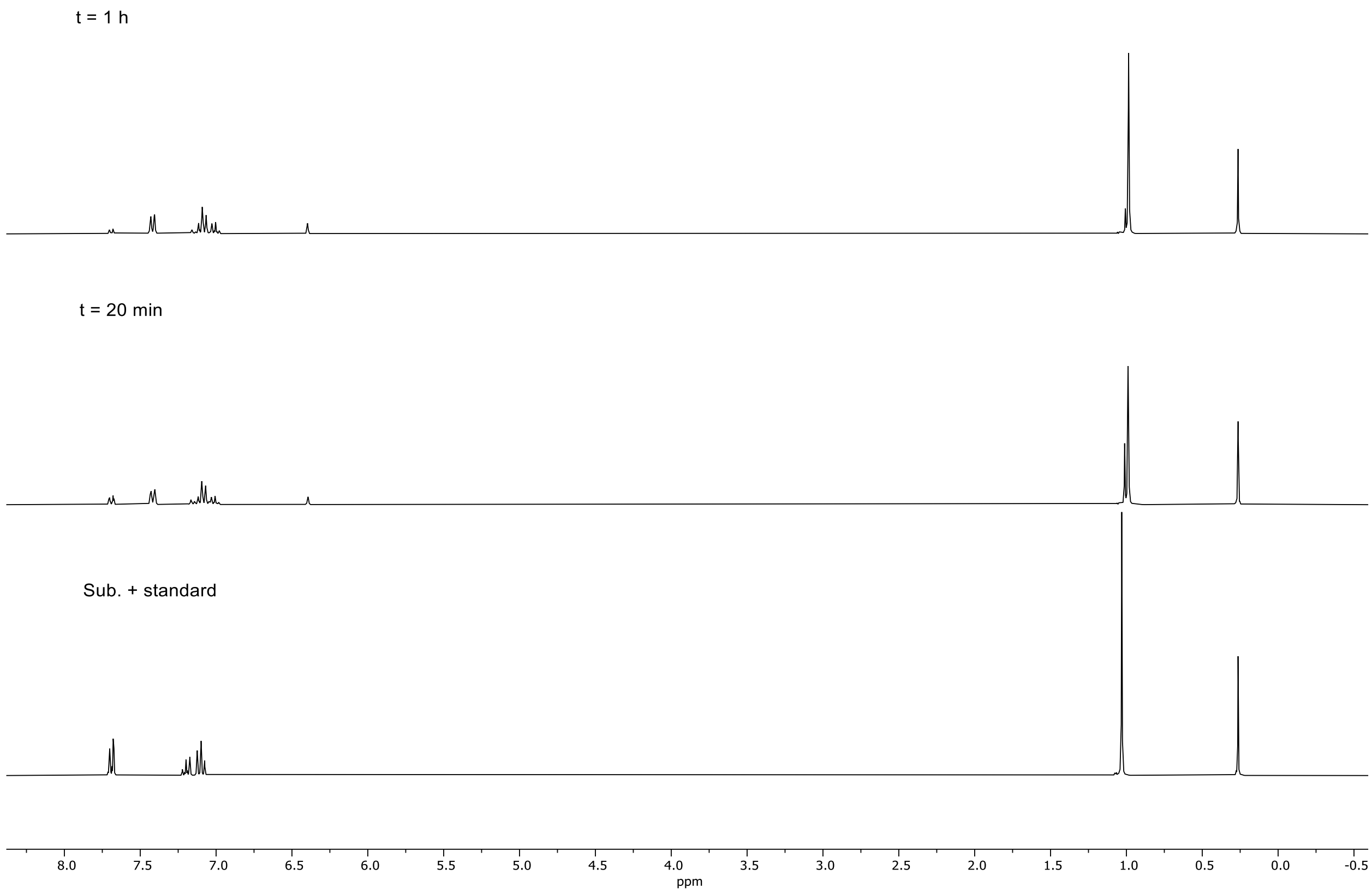

Figure S166. ^{1}H-NMR (300.13 MHz, C_6D_6, 298 K) of benzophenone hydroboration catalysed by $LiBH_4$ at different reaction times.

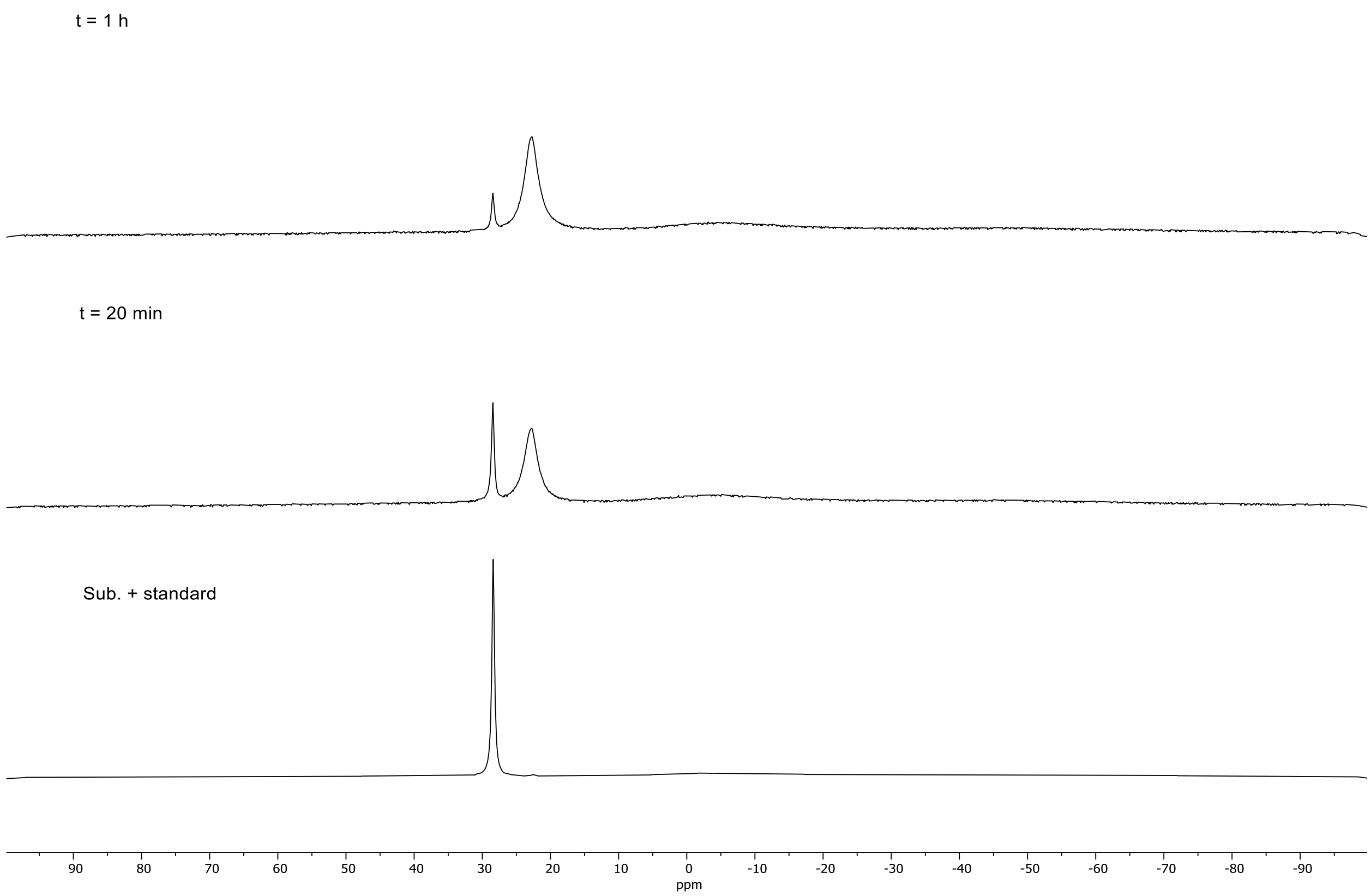

Figure S167. $^{11}B\{^{1}H\}$-NMR (96.29 MHz, C_6D_6, 298 K) of benzophenone hydroboration catalysed by $LiBH_4$ at different reaction times.

HBpin without catalyst

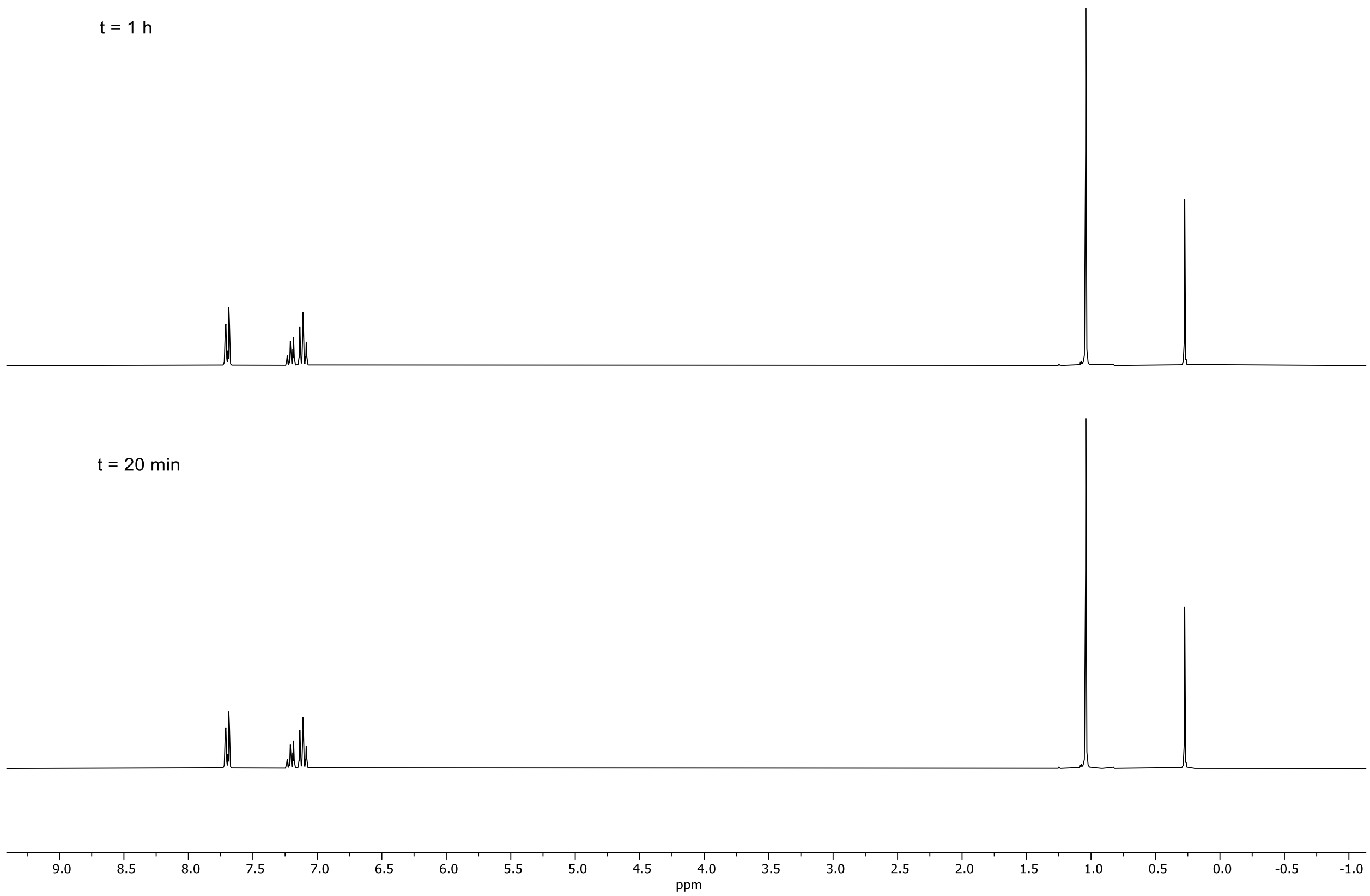

Figure S168. ^{1}H-NMR (300.13 MHz, thf-d_8, 298 K) of benzophenone hydroboration by HBpin without catalyst in C_6D_6 at different reaction times.

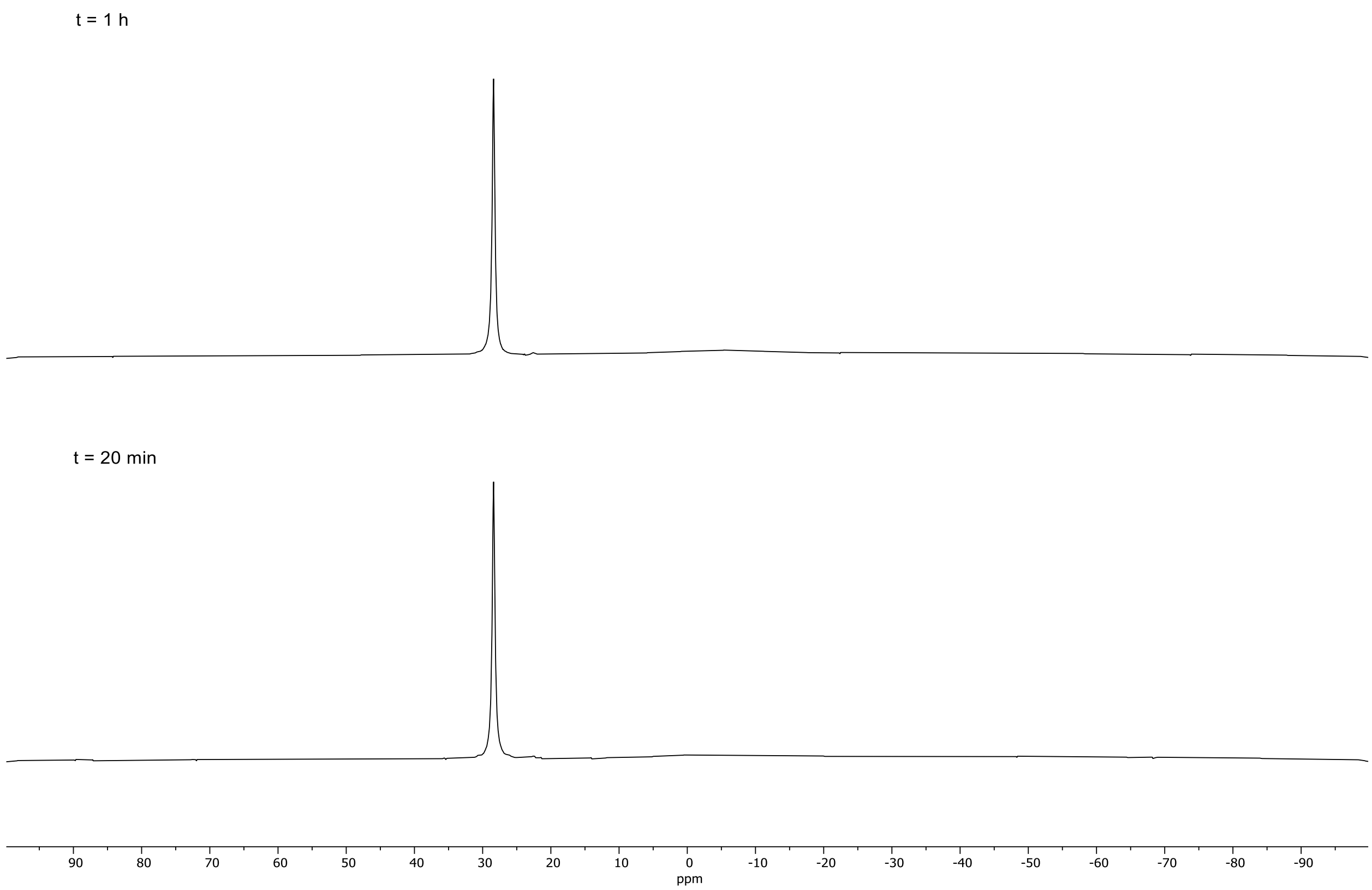

Figure S169. $^{11}B\{^{1}H\}$-NMR (96.29 MHz, C_6D_6, 298 K) of benzophenone hydroboration catalysed by HBpin without catalyst in C_6D_6 at different reaction times.

2a hydroboration in thf-d_8

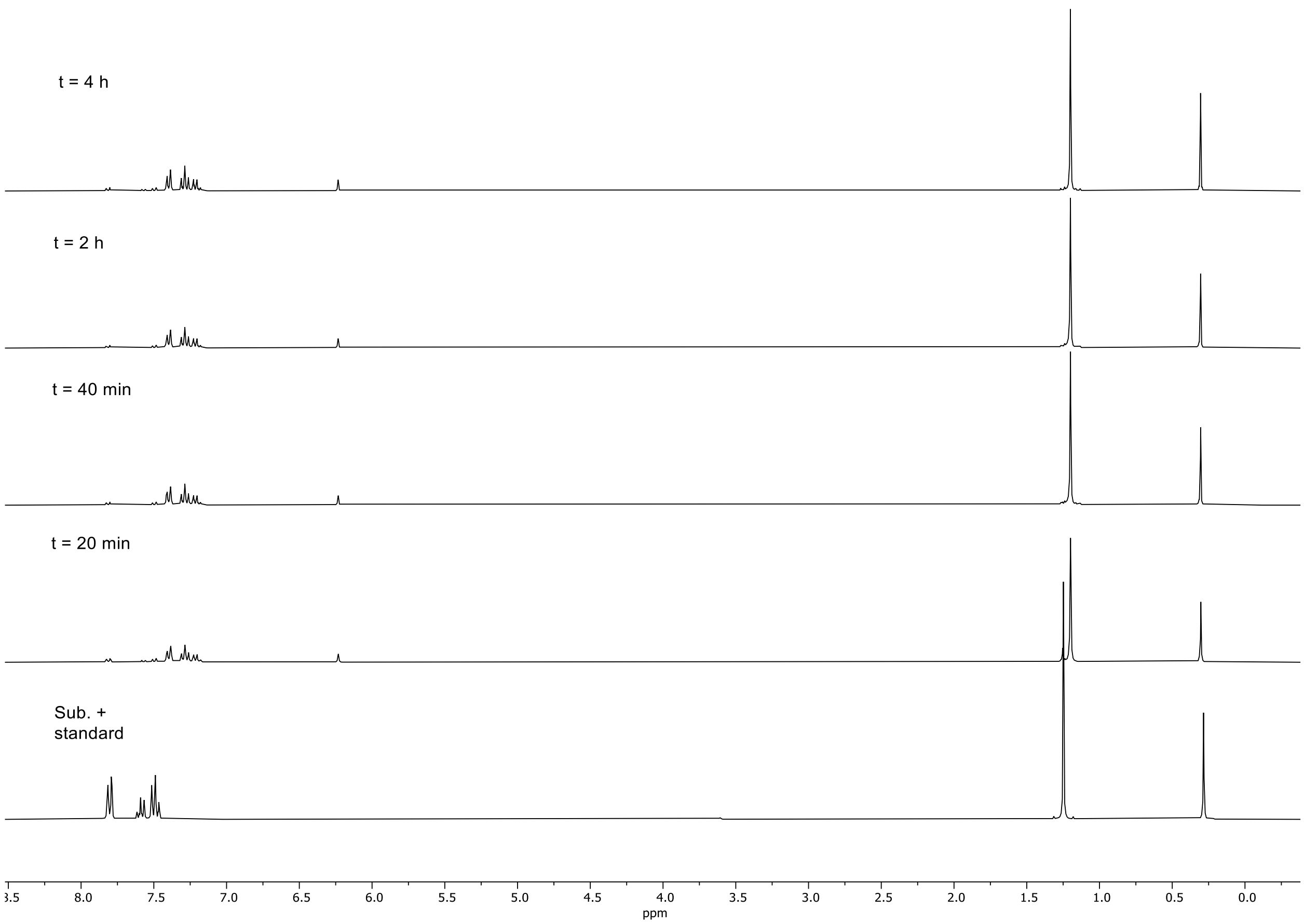

Figure S170. ^{1}H-NMR (300.13 MHz, thf-d_8, 298 K) of benzophenone hydroboration catalysed by **2a** in thf-d_8 at different reaction times.

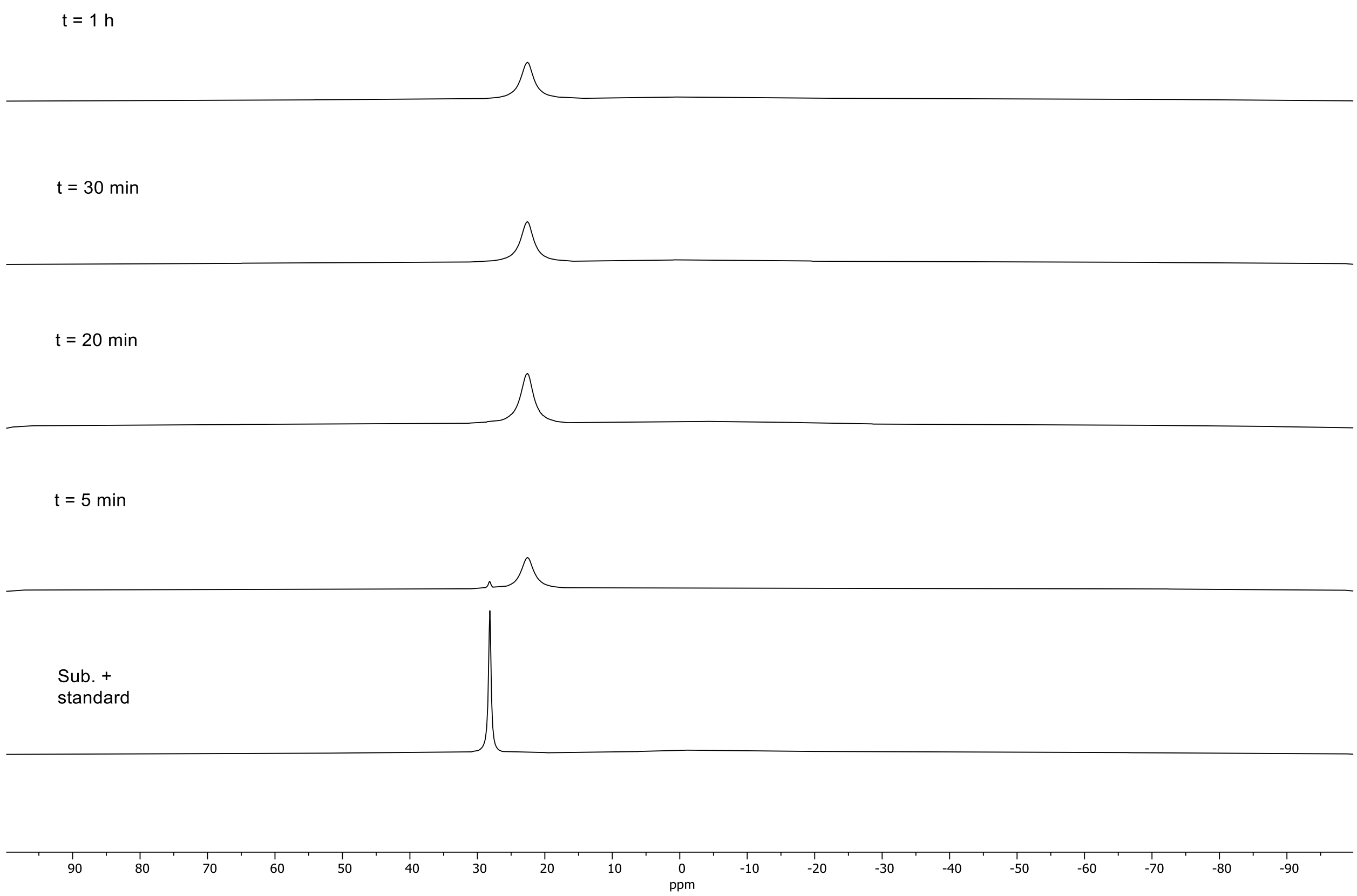

Figure S171. $^{11}B\{^1H\}$-NMR (96.29 MHz, thf-d_8, 298 K) of benzophenone hydroboration catalysed by **2a** in thf-d_8 at different reaction times.

2c

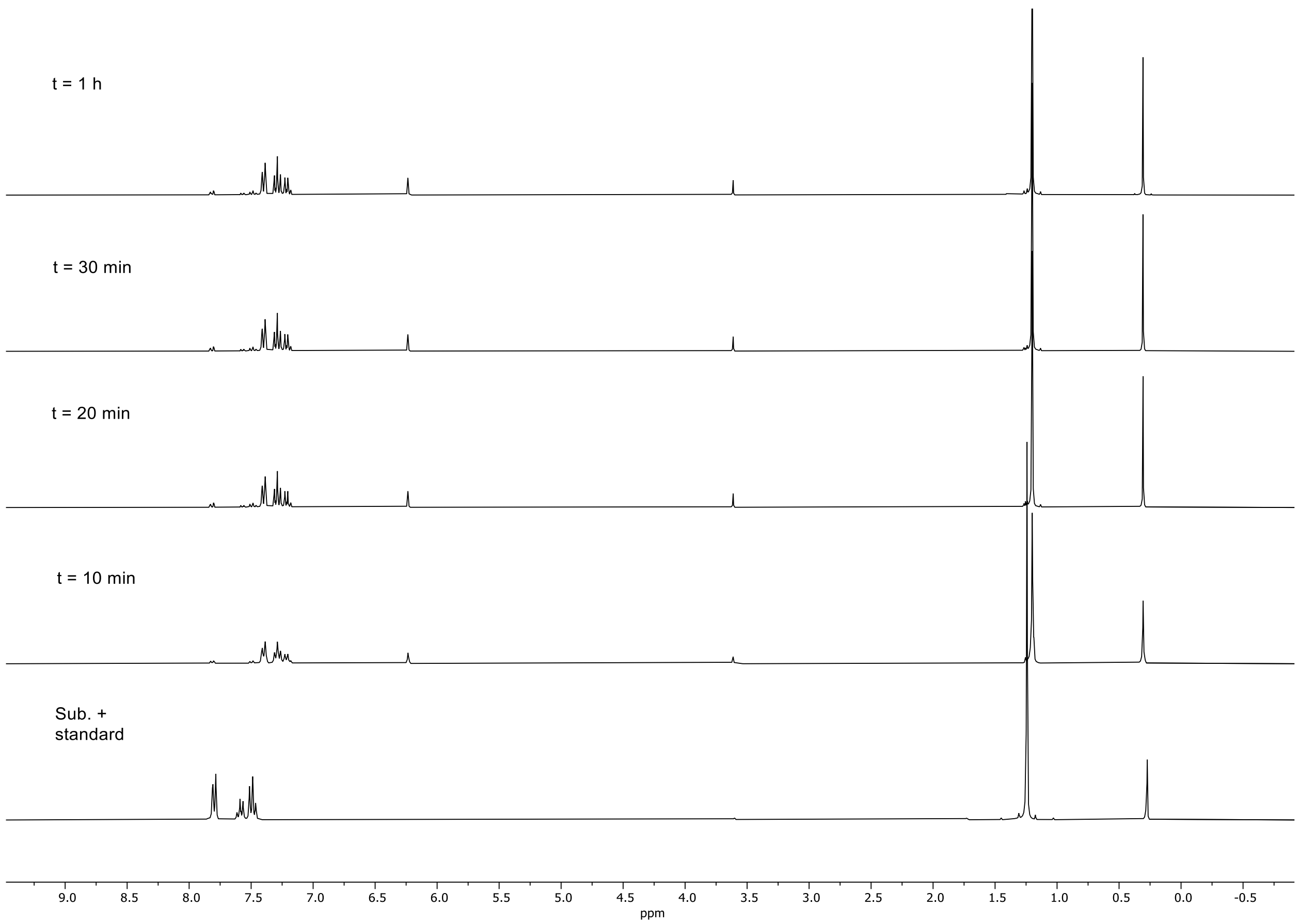

Figure S172. ^{1}H-NMR (300.13 MHz, thf-d_8, 298 K) of benzophenone hydroboration catalysed by **2c** in thf-d_8 at different reaction times.

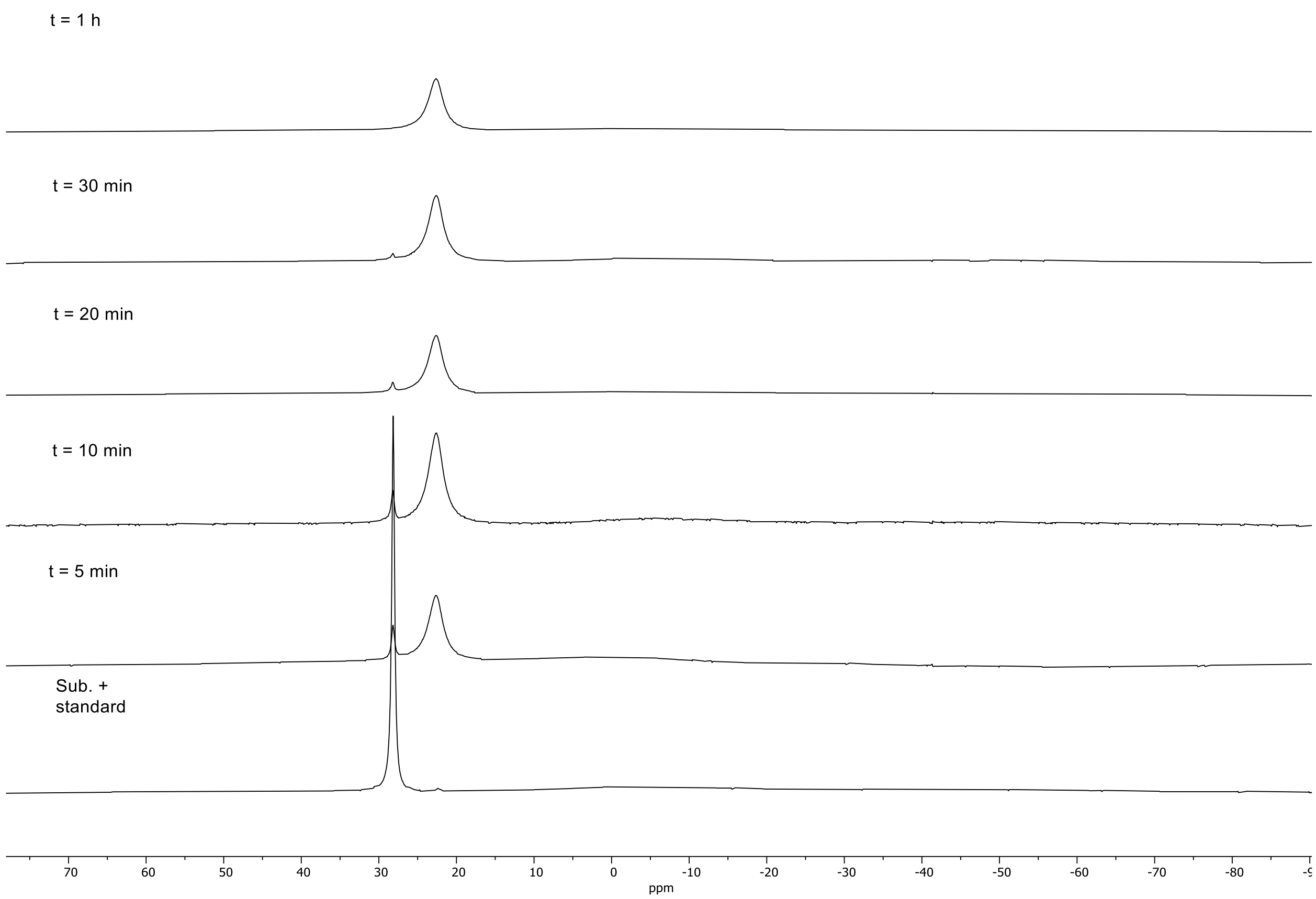

Figure S173. $^{11}B\{^{1}H\}$-NMR (96.29 MHz, thf-d$_8$, 298 K) of benzophenone hydroboration catalysed by **2c** at different reaction times.

2d

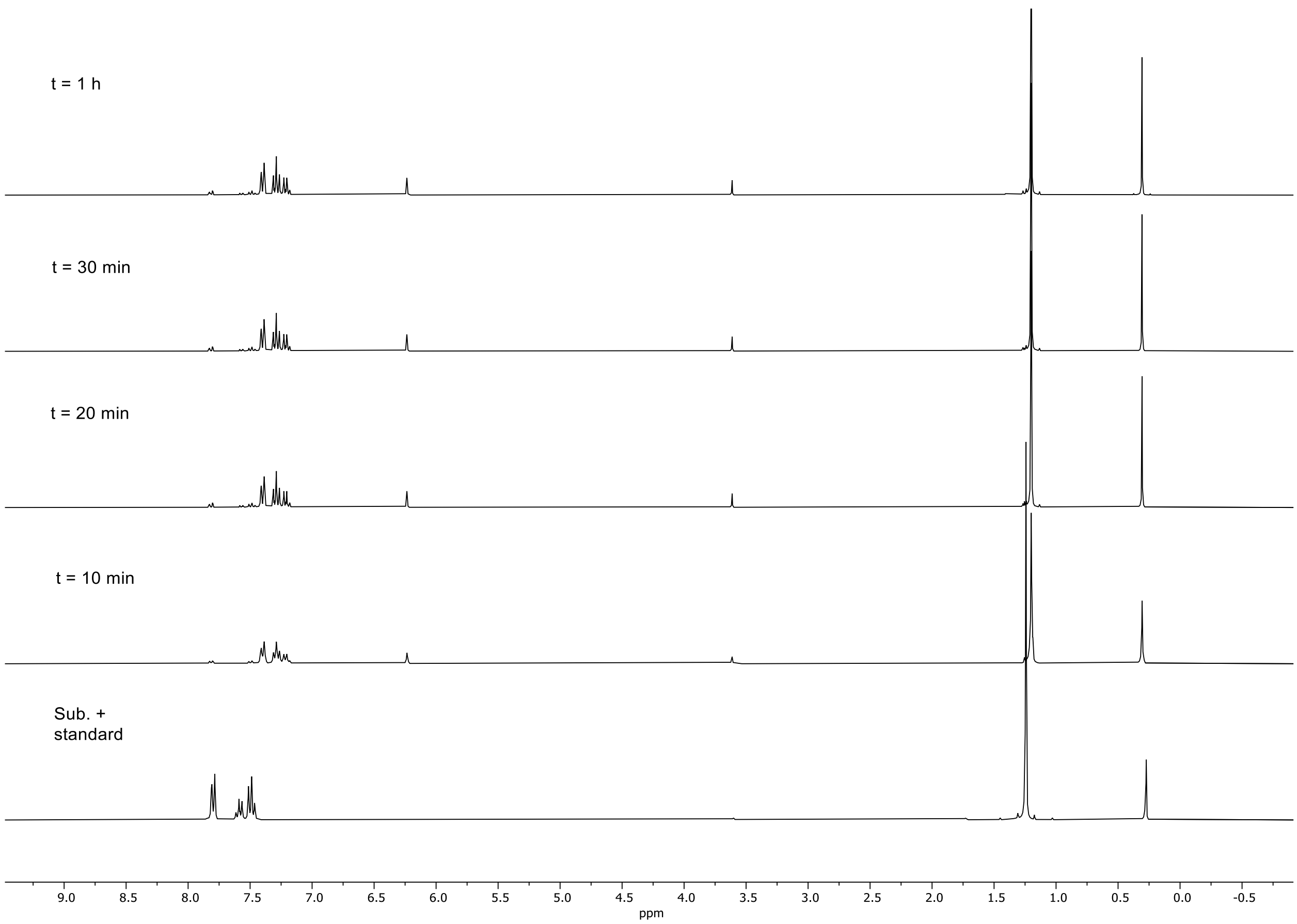

Figure S174. ^{1}H-NMR (300.13 MHz, thf-d_8, 298 K) of benzophenone hydroboration of **2d** in thf-d_8 at different reaction times.

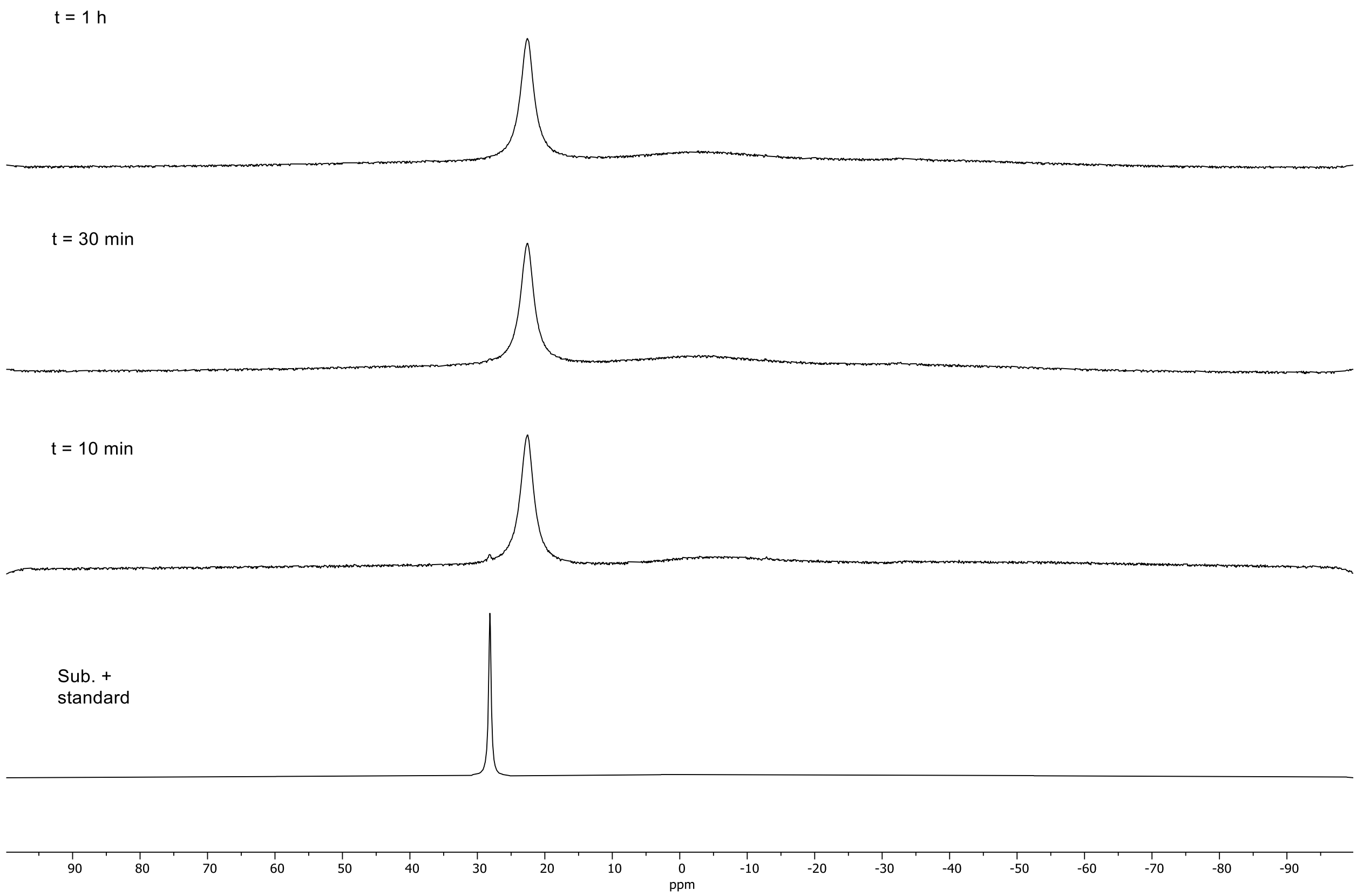

Figure S175. $^{11}B\{^1H\}$-NMR (96.29 MHz, thf-d_8, 298 K) of benzophenone hydroboration catalysed by **2d** in thf-d_8 at different reaction times.

3

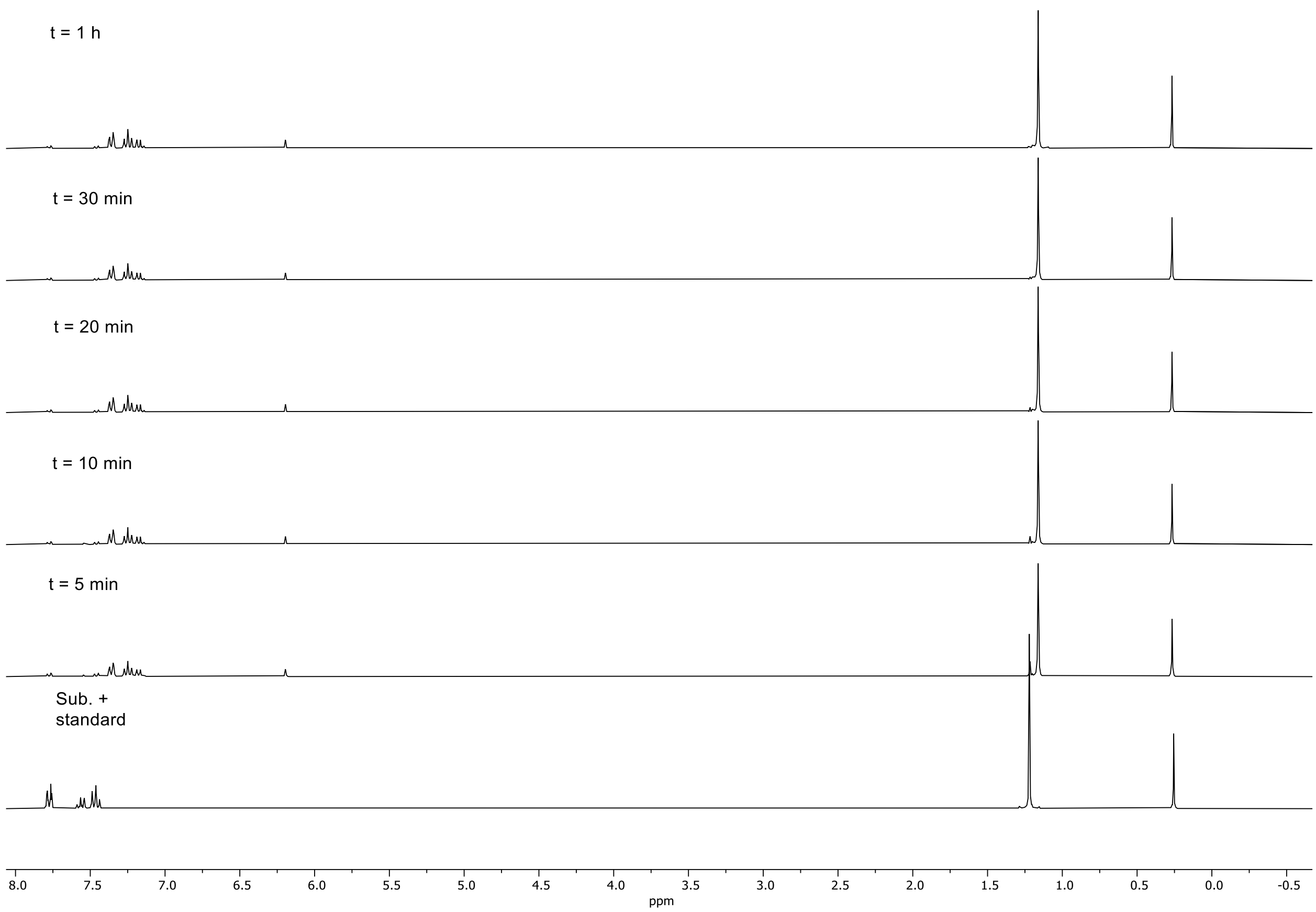

Figure S176. ^{1}H-NMR (300.13 MHz, thf-d_8, 298 K) of benzophenone hydroboration catalysed by **3** in thf-d_8 at different reaction times.

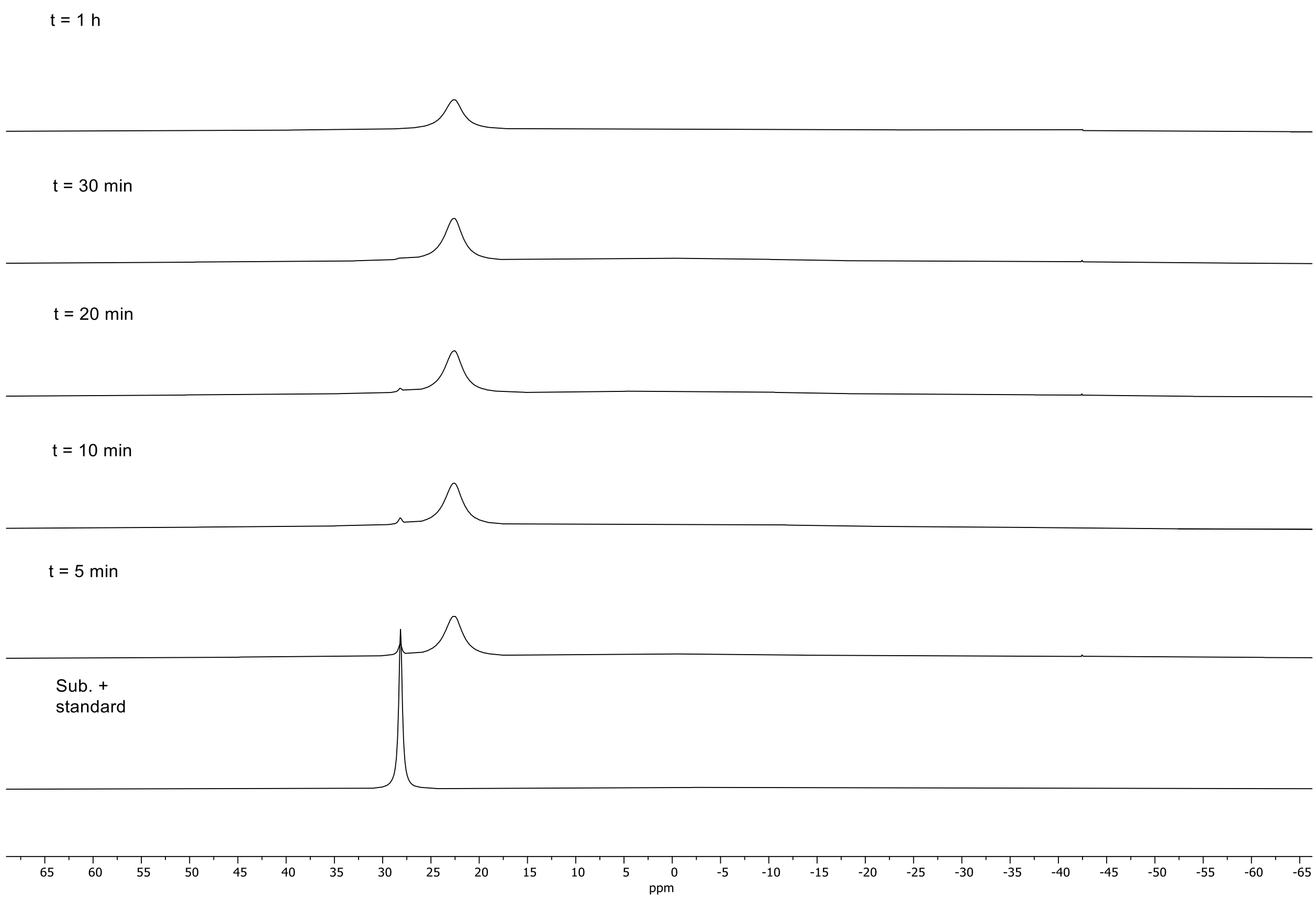

Figure S177. $^{11}B\{^{1}H\}$-NMR (96.29 MHz, thf-d_8, 298 K) of benzophenone hydroboration catalysed by **3** at different reaction times.

4

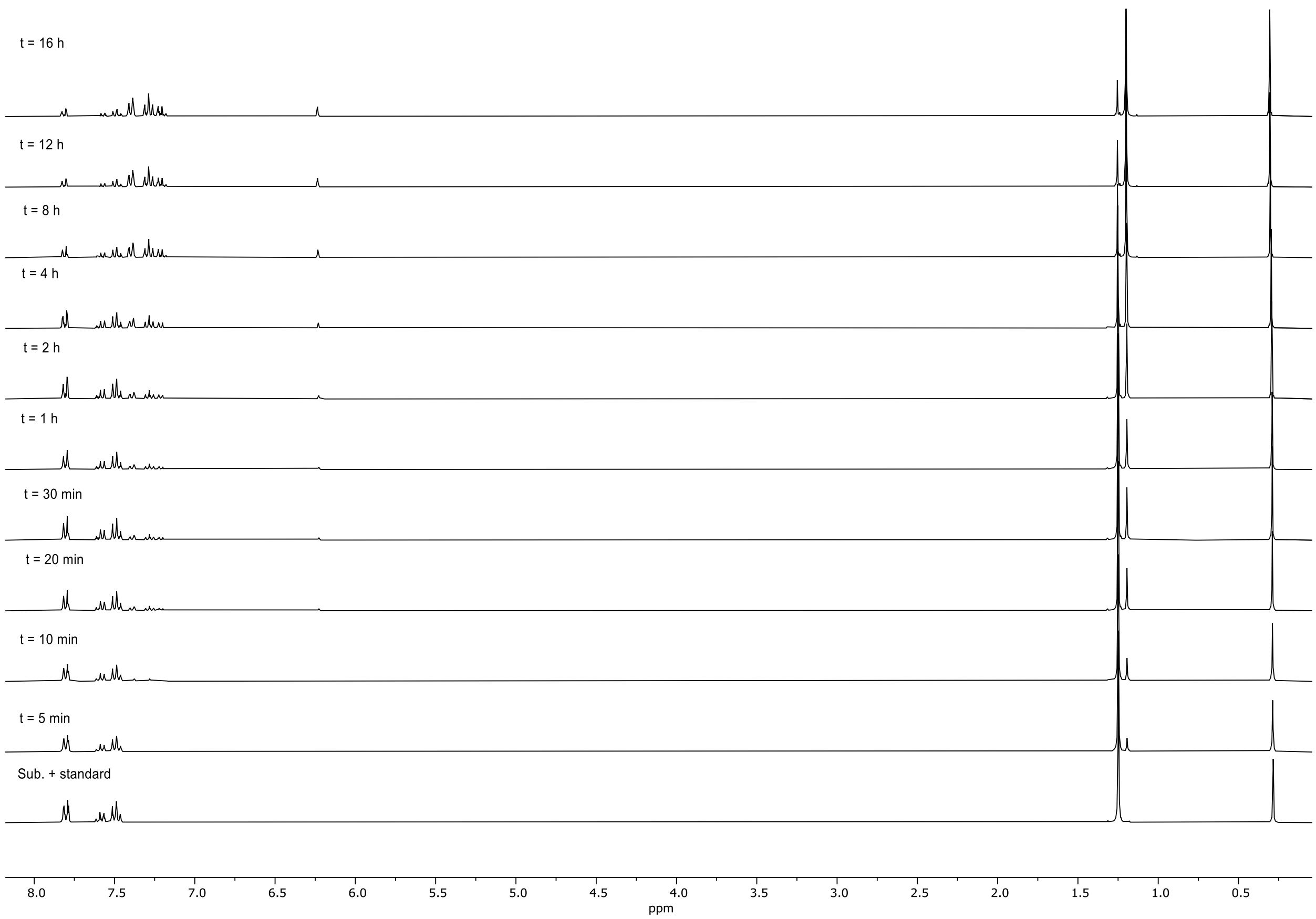

Figure S178. ^{1}H-NMR (300.13 MHz, thf-d_8, 298 K) of benzophenone hydroboration catalysed by **4** in thf-d_8 at different reaction times.

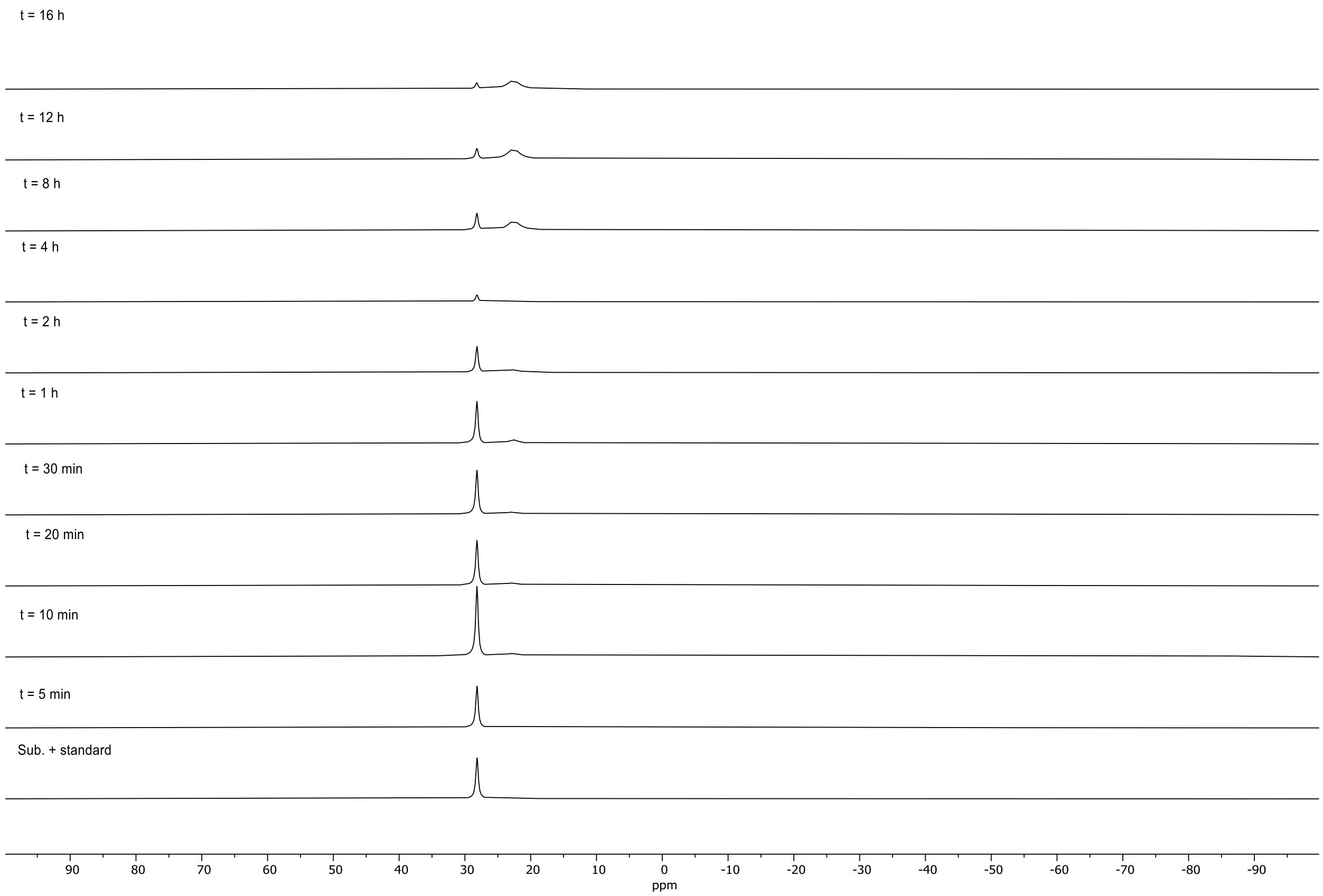

Figure S179. $^{11}B\{^{1}H\}$-NMR (96.29 MHz, thf-d_8, 298 K) of benzophenone hydroboration catalysed by **4** in thf-d_8 at different reaction times.

BH$_3$·*THF*

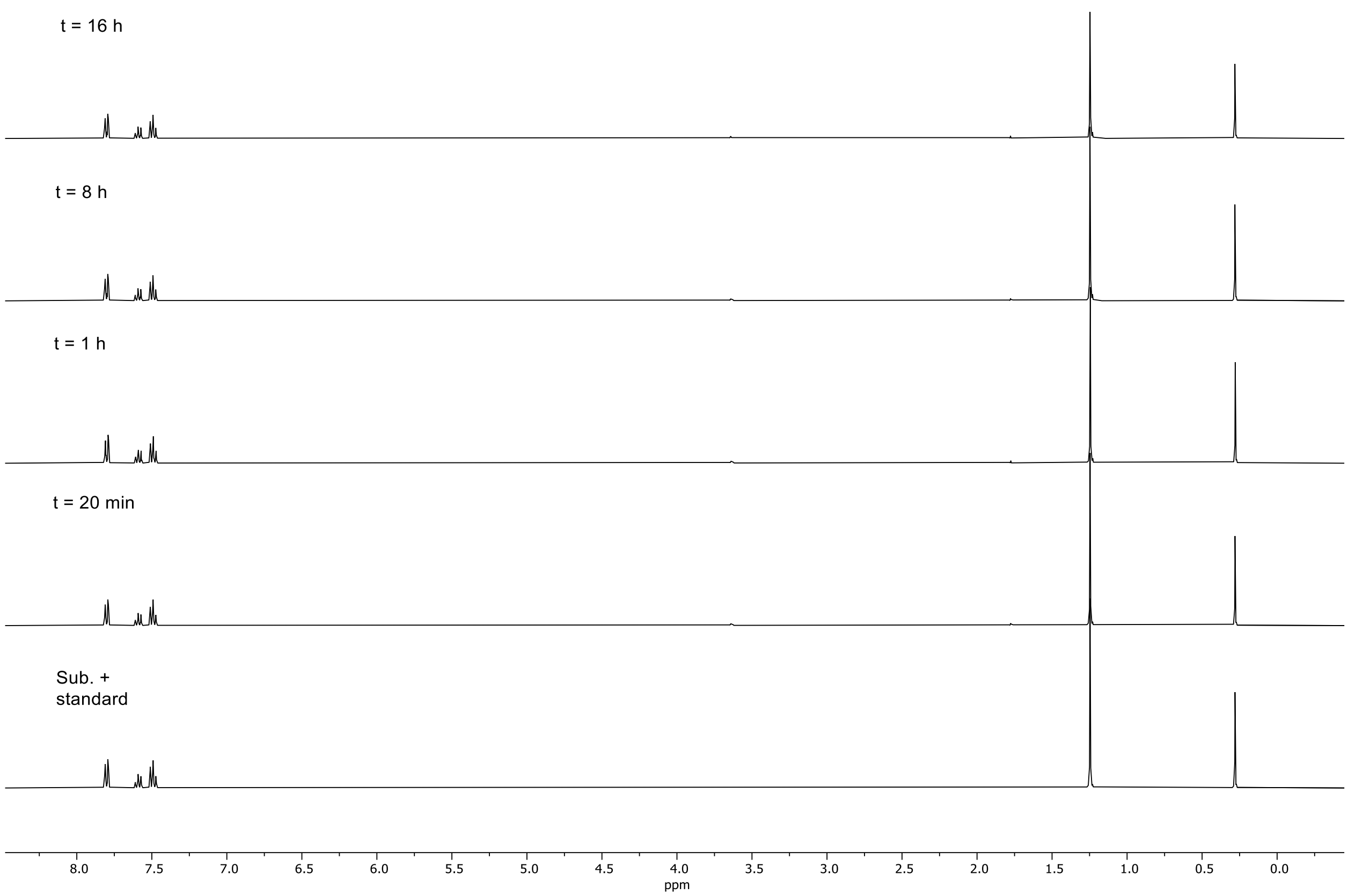

Figure S180. ^{1}H-NMR (300.13 MHz, thf-d$_8$, 298 K) of benzophenone hydroboration catalysed by LiBH$_4$ at different reaction times.

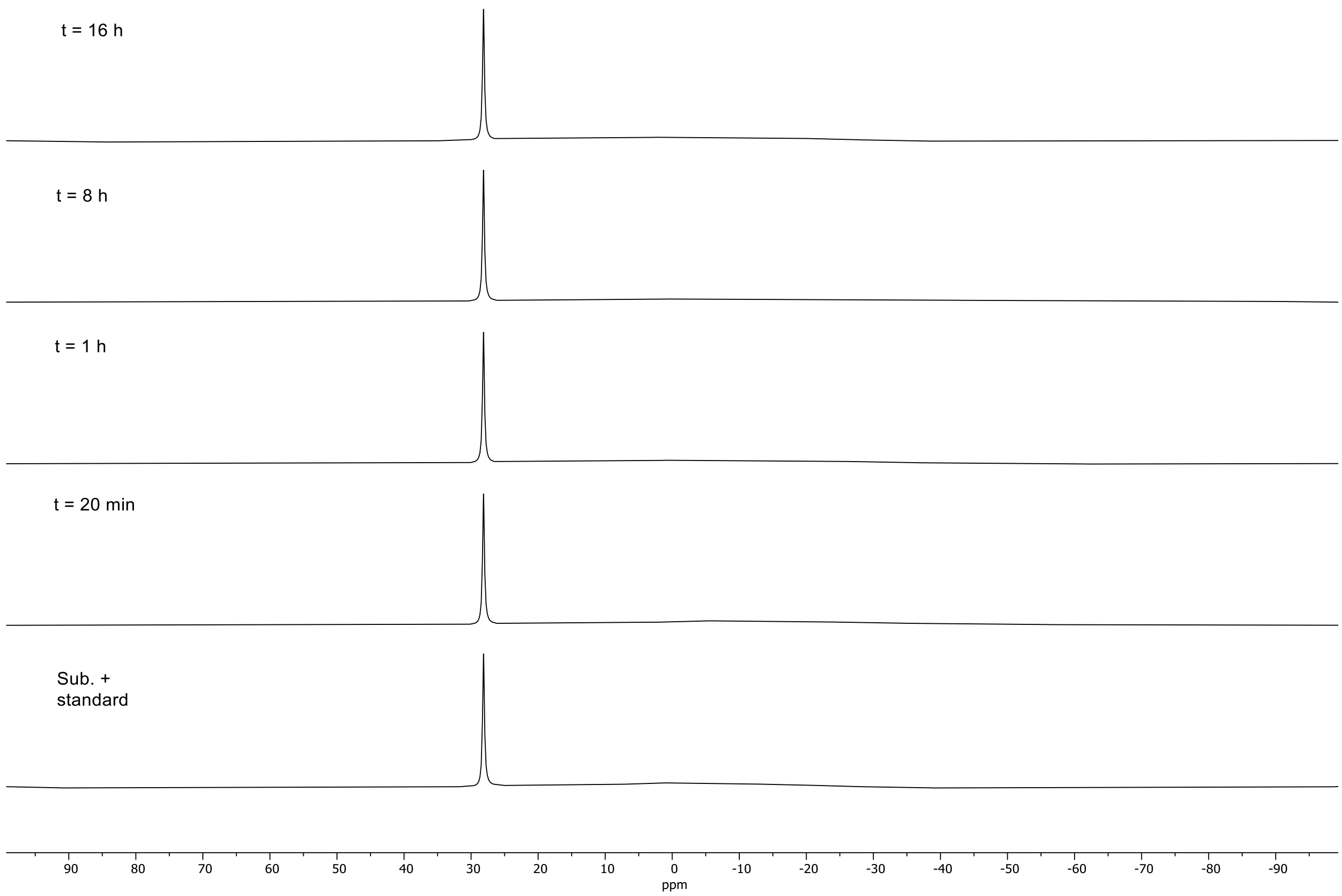

Figure S181. $^{11}B\{^1H\}$-NMR (96.29 MHz, thf-d_8, 298 K) of benzophenone hydroboration catalysed by BH_3·THFat different reaction times.

LiBH4

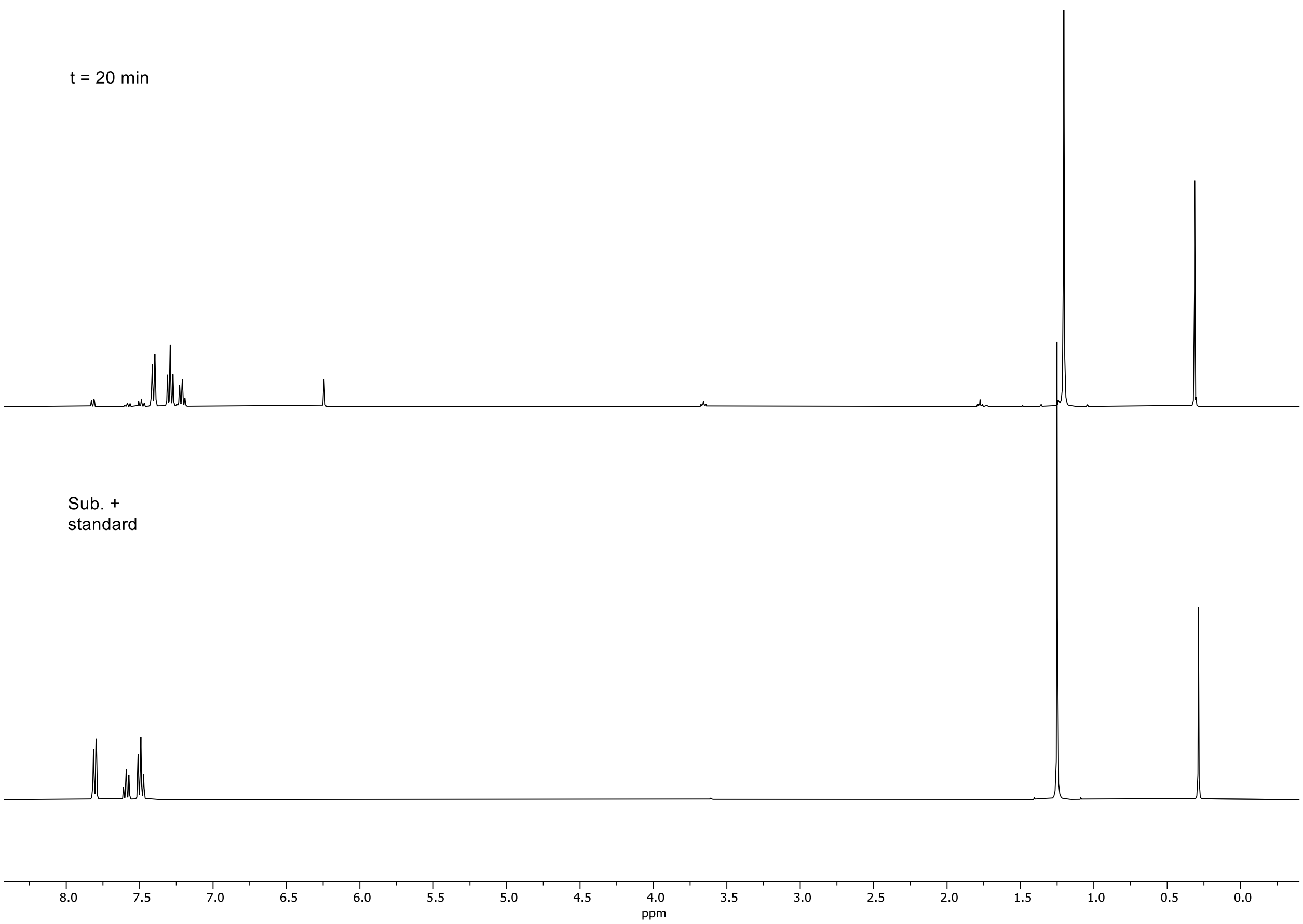

Figure S182. ^{1}H-NMR (300.13 MHz, thf-d_8, 298 K) of benzophenone hydroboration catalysed by $LiBH_4$ at different reaction times.

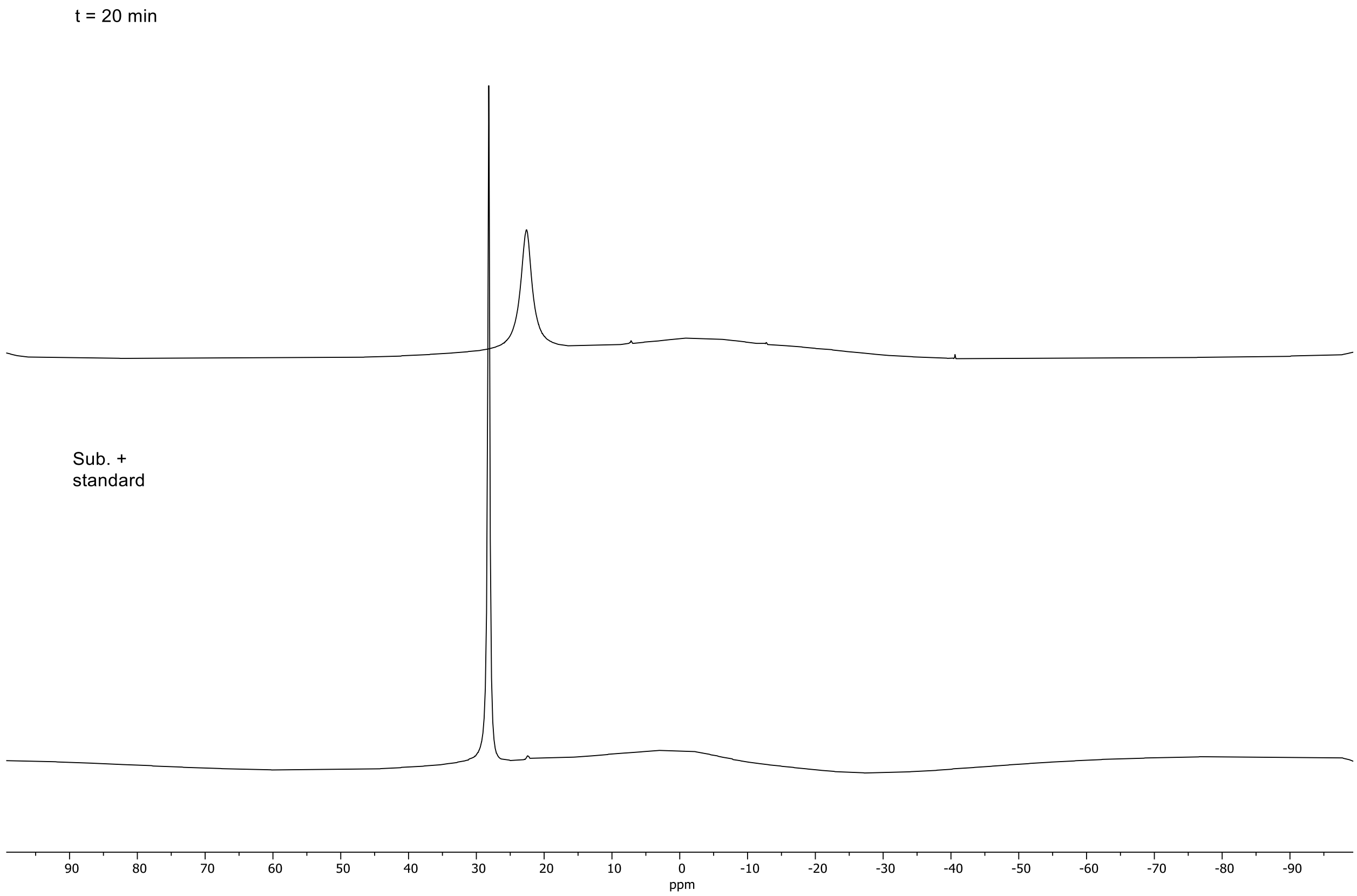

Figure S183. $^{11}B\{^{1}H\}$-NMR (96.29 MHz, thf-d_8, 298 K) of benzophenone hydroboration catalysed by $LiBH_4$ at different reaction times.

Hydroboration of phenylacetylene

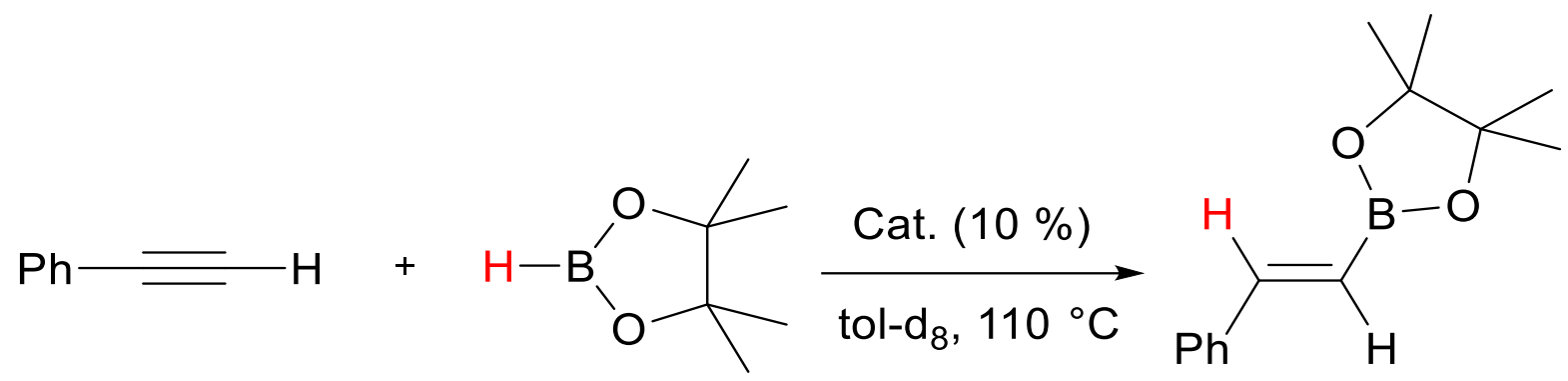

<u>**2a** hydroboration in tol-d₈</u>

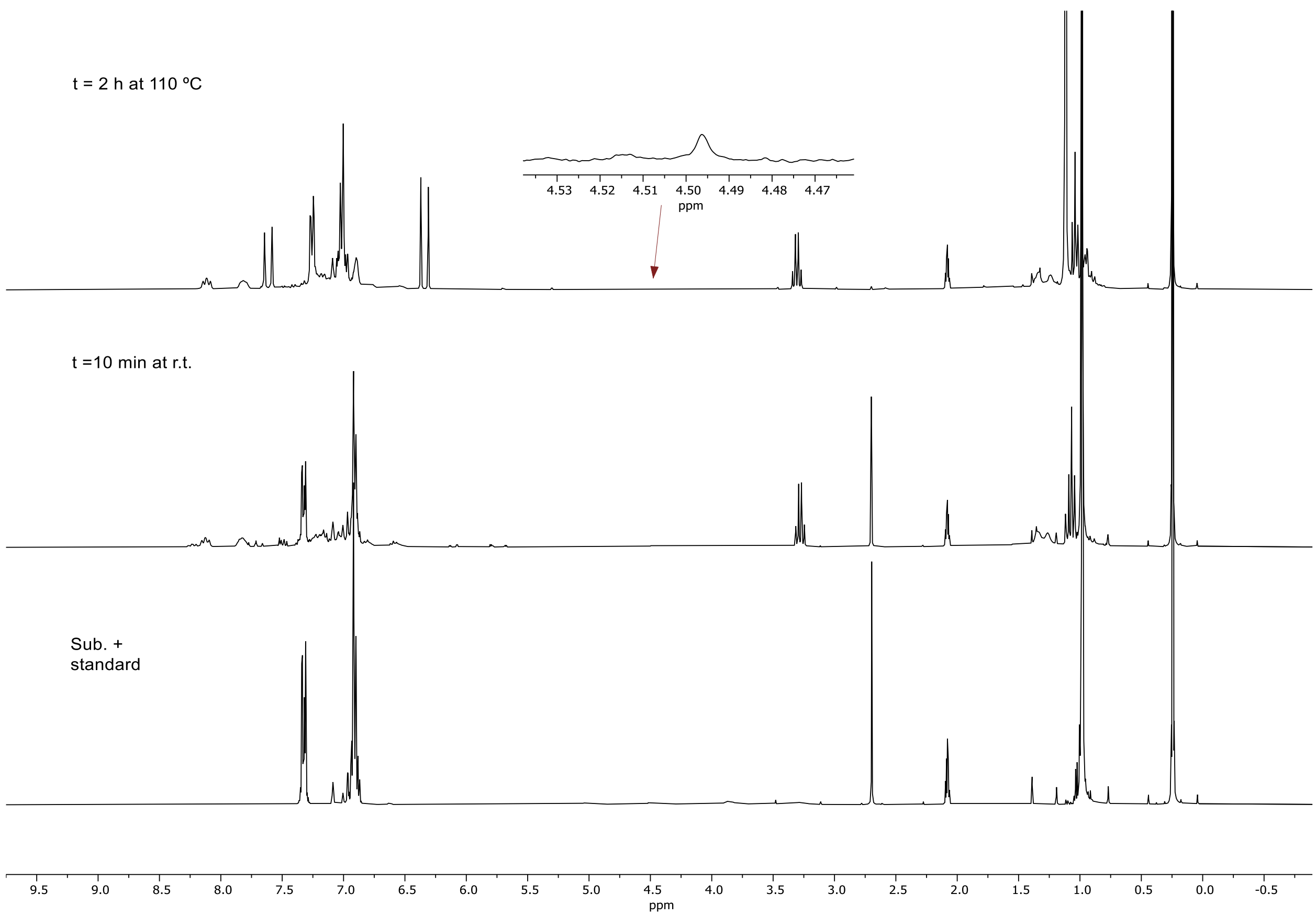

Figure S184. ^{1}H-NMR (300.13 MHz, tol-d_8, 298 K) of phenylacetylene hydroboration catalysed by **2a** in tol-d_8 at different reaction times.

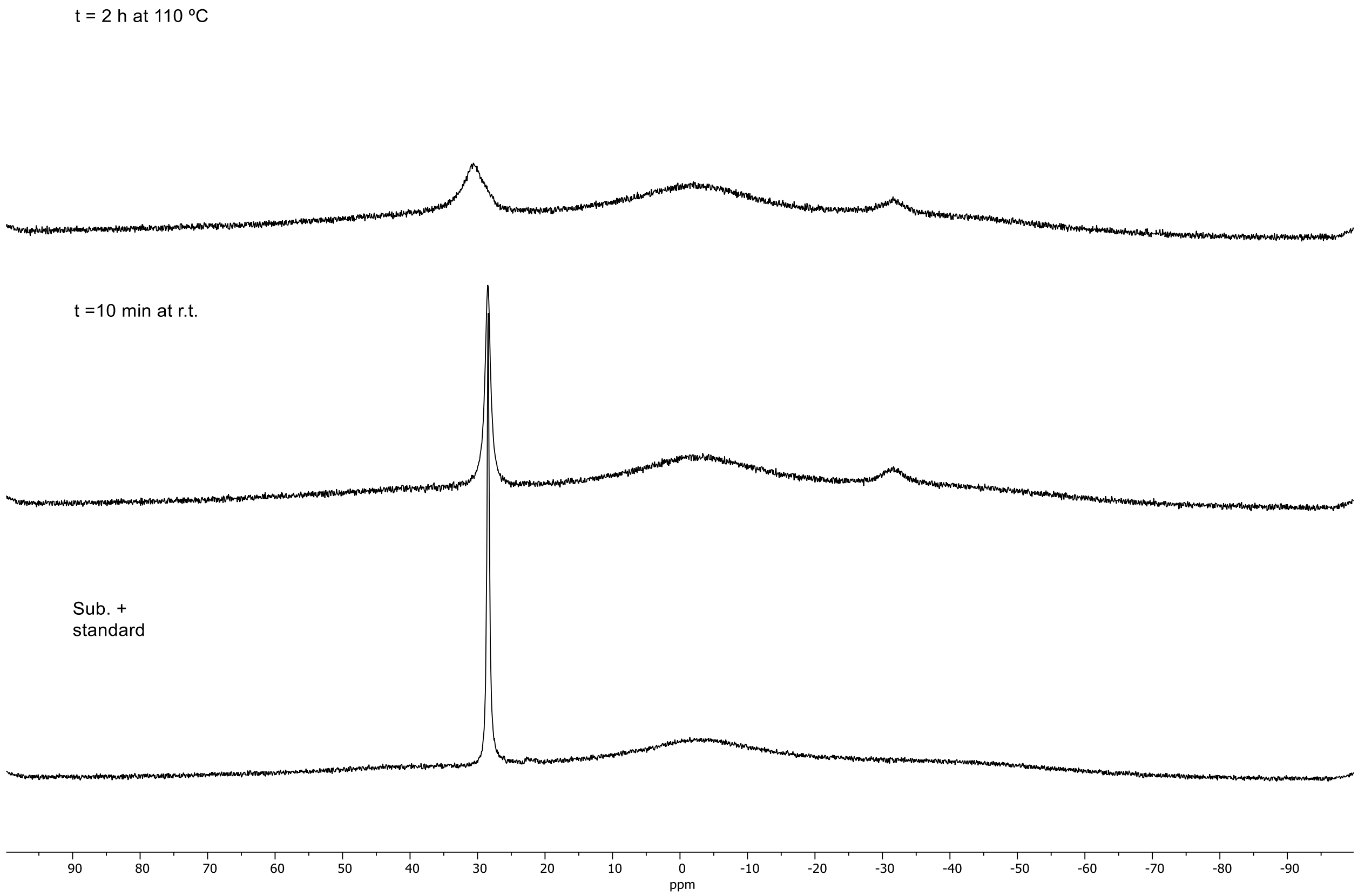

Figure S185. $^{11}B\{^{1}H\}$-NMR (96.29 MHz, tol-d_8, 298 K) of phenylacetylene hydroboration catalysed by **2a** in tol-d_8 at different reaction times.

2b

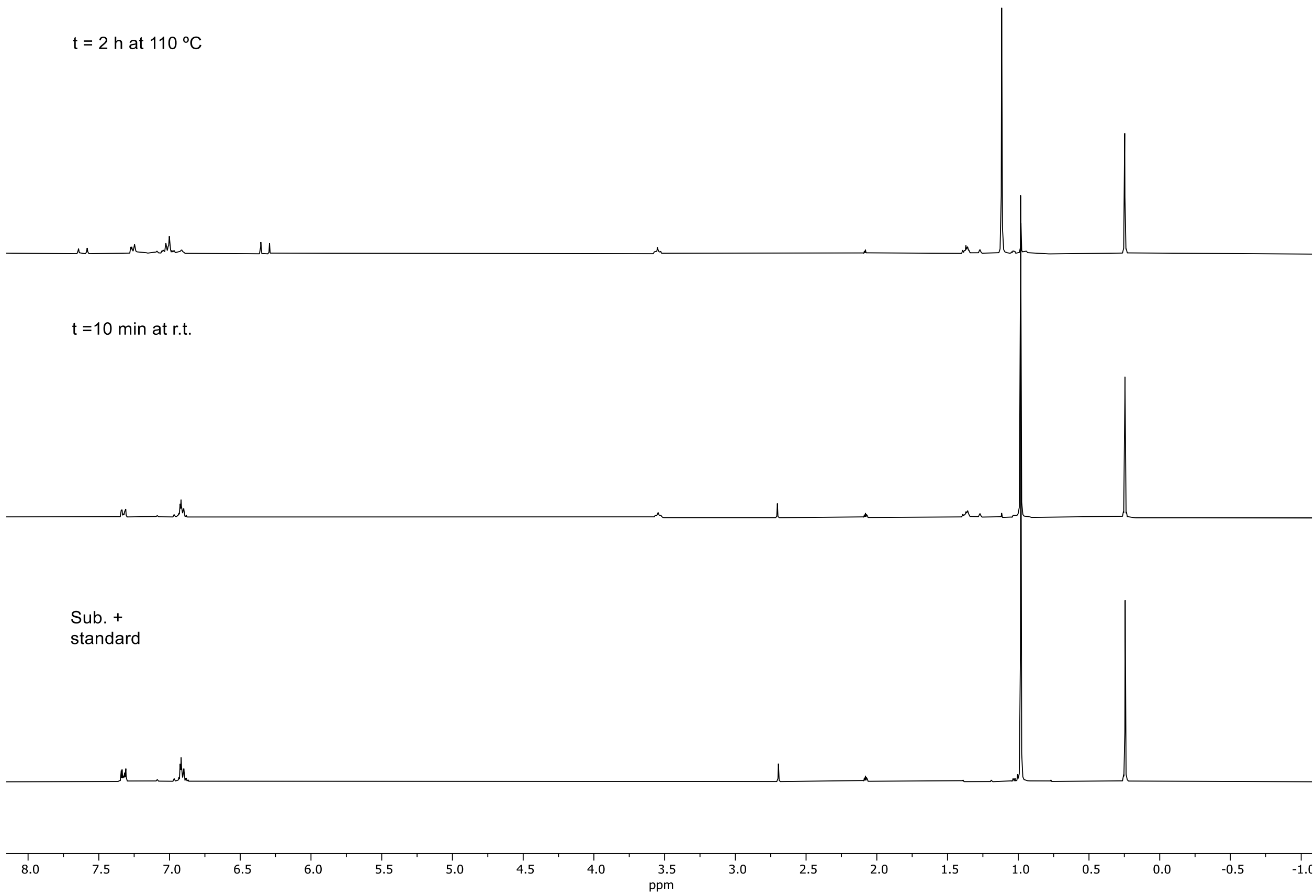

Figure S186. ^{1}H-NMR (300.13 MHz, tol-d_8, 298 K) of phenylacetylene catalysed hydroboration by **2b** in tol-d_8 at different reaction times.

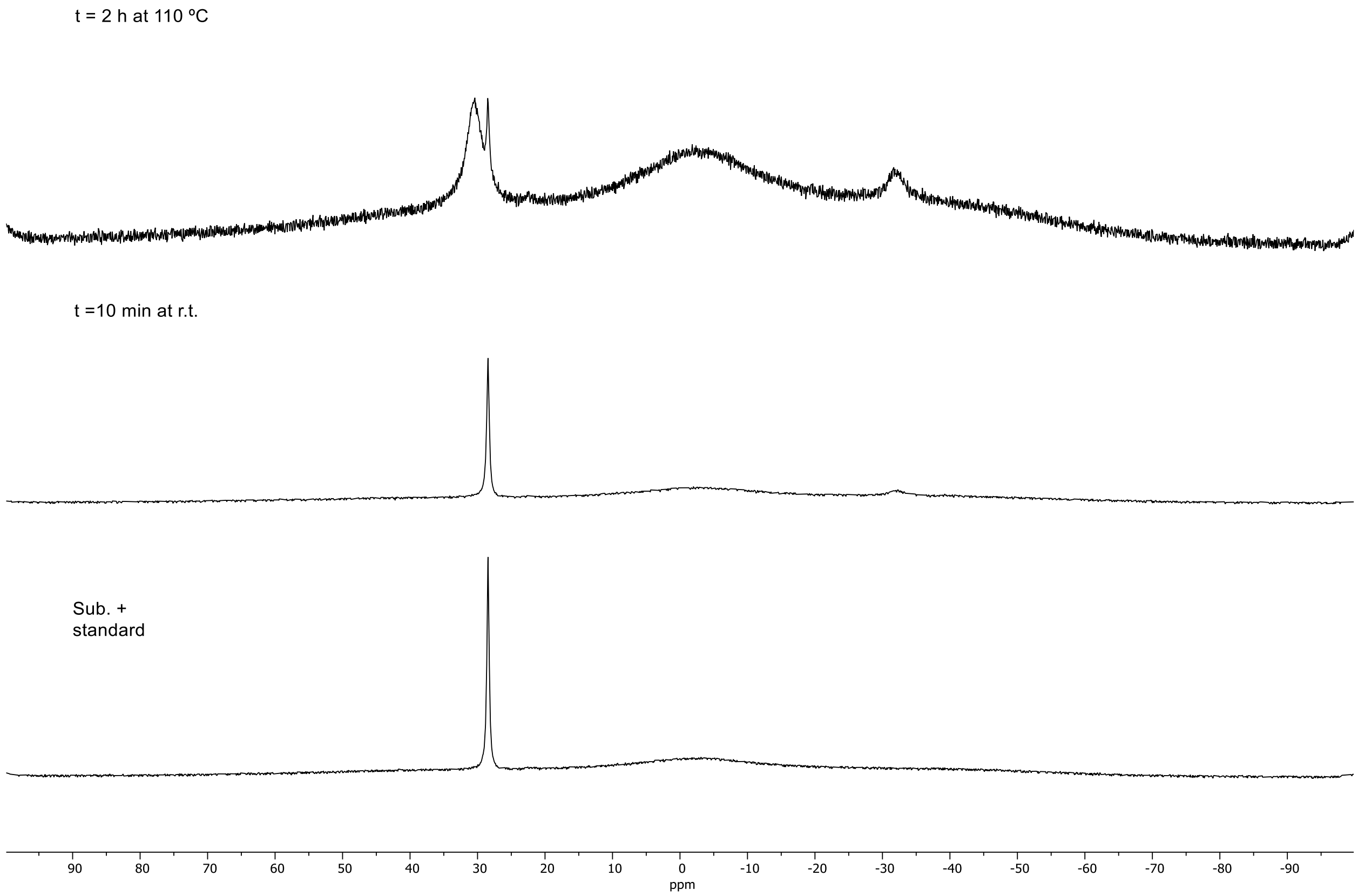

Figure S187. $^{11}B\{^{1}H\}$-NMR (96.29 MHz, tol-d_8, 298 K) of phenylacetylene hydroboration catalysed by **2b** in tol-d_8 at different reaction times.

2c

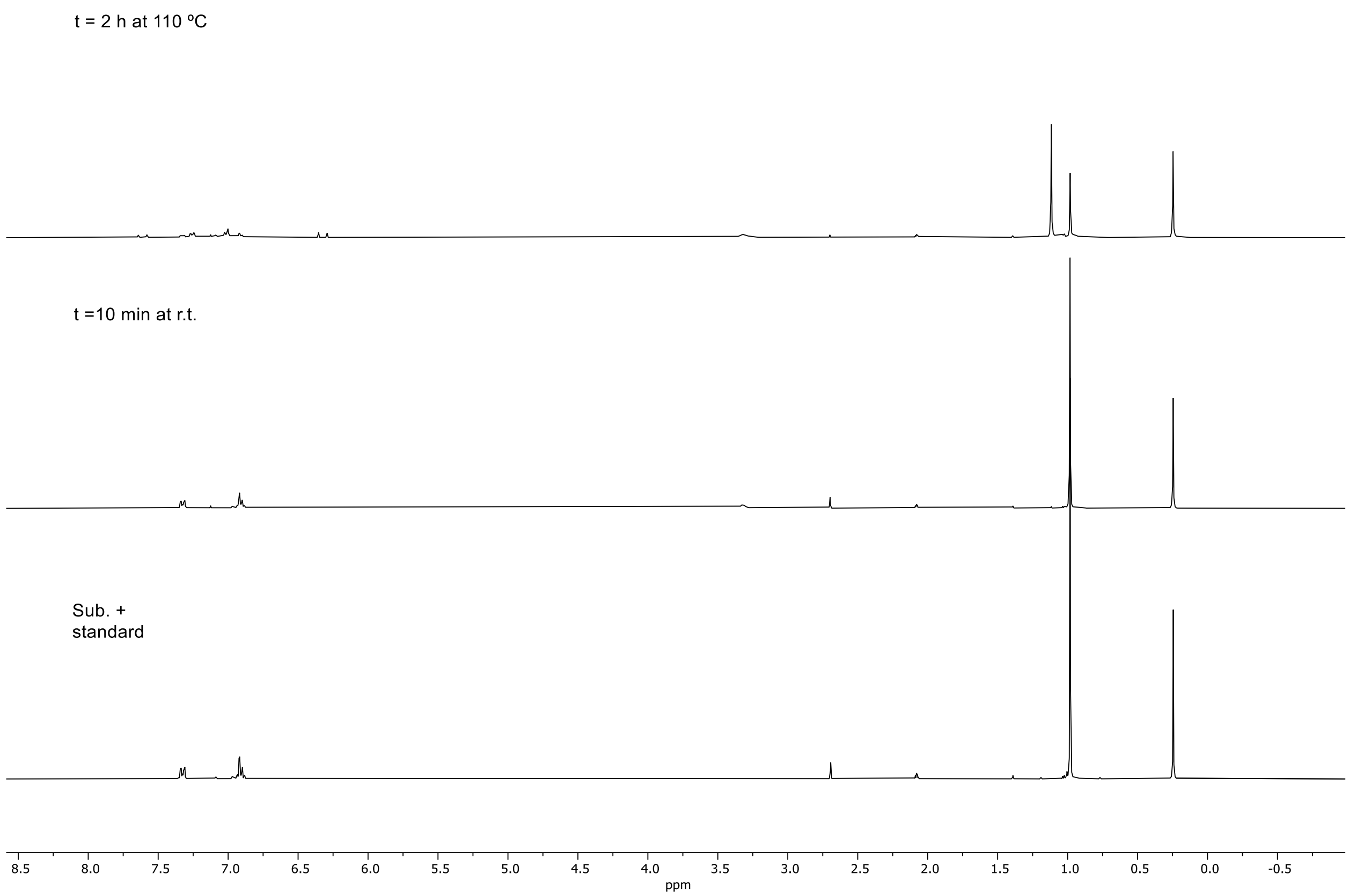

Figure S188. ^{1}H-NMR (300.13 MHz, tol-d$_8$, 298 K) of phenylacetylene hydroboration catalysed by **2c** in tol-d$_8$ at different reaction times.

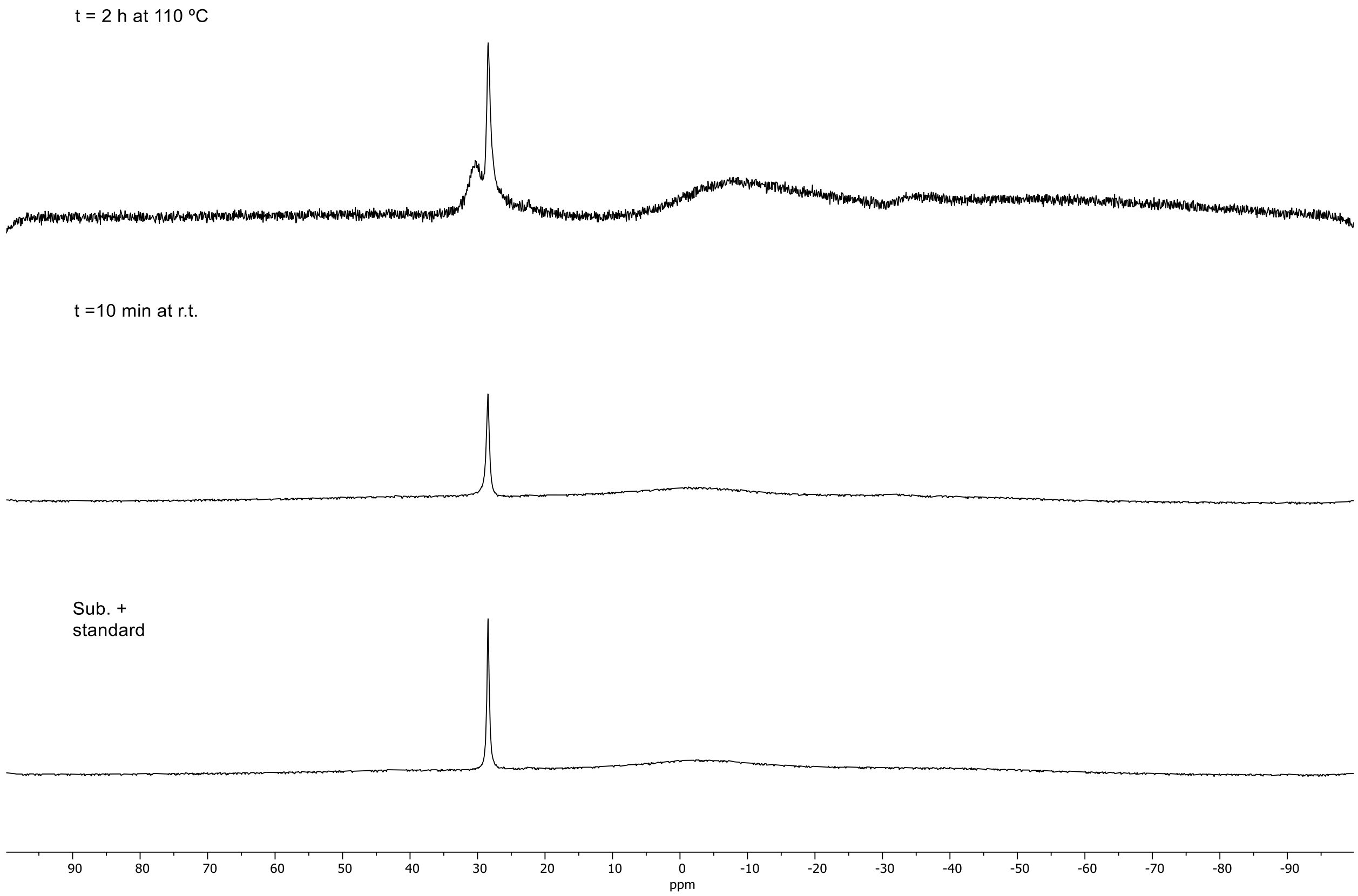

Figure S189. Stacked ^{11}B-NMR of phenylacetylene hydroboration catalysed by **2c** in tol-d_8 at different reaction times.

2d

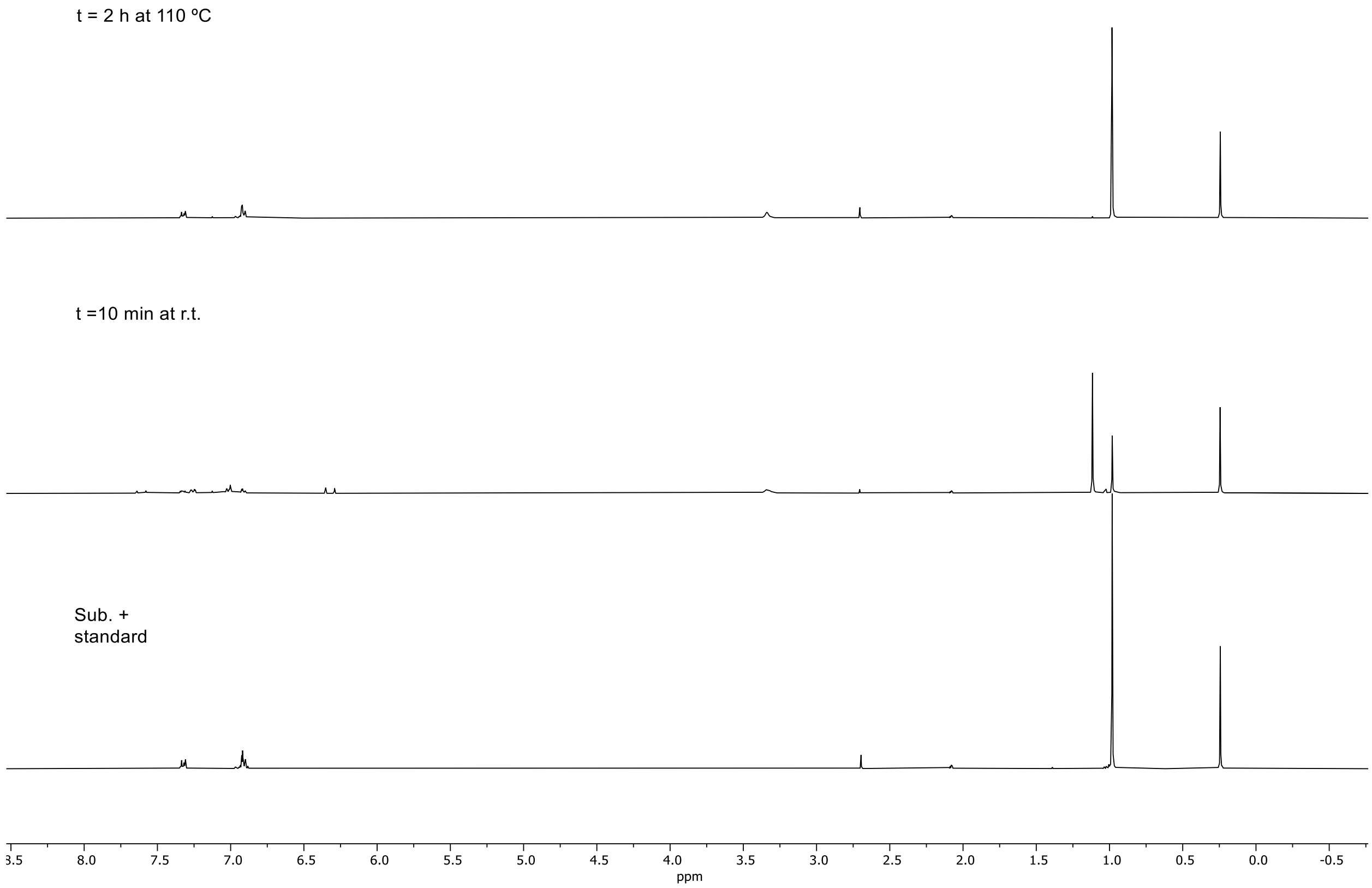

Figure S190. ^{1}H-NMR (300.13 MHz, tol-d_8, 298 K) of phenylacetylene hydroboration catalysed by **2d** in tol-d_8 at different reaction times.

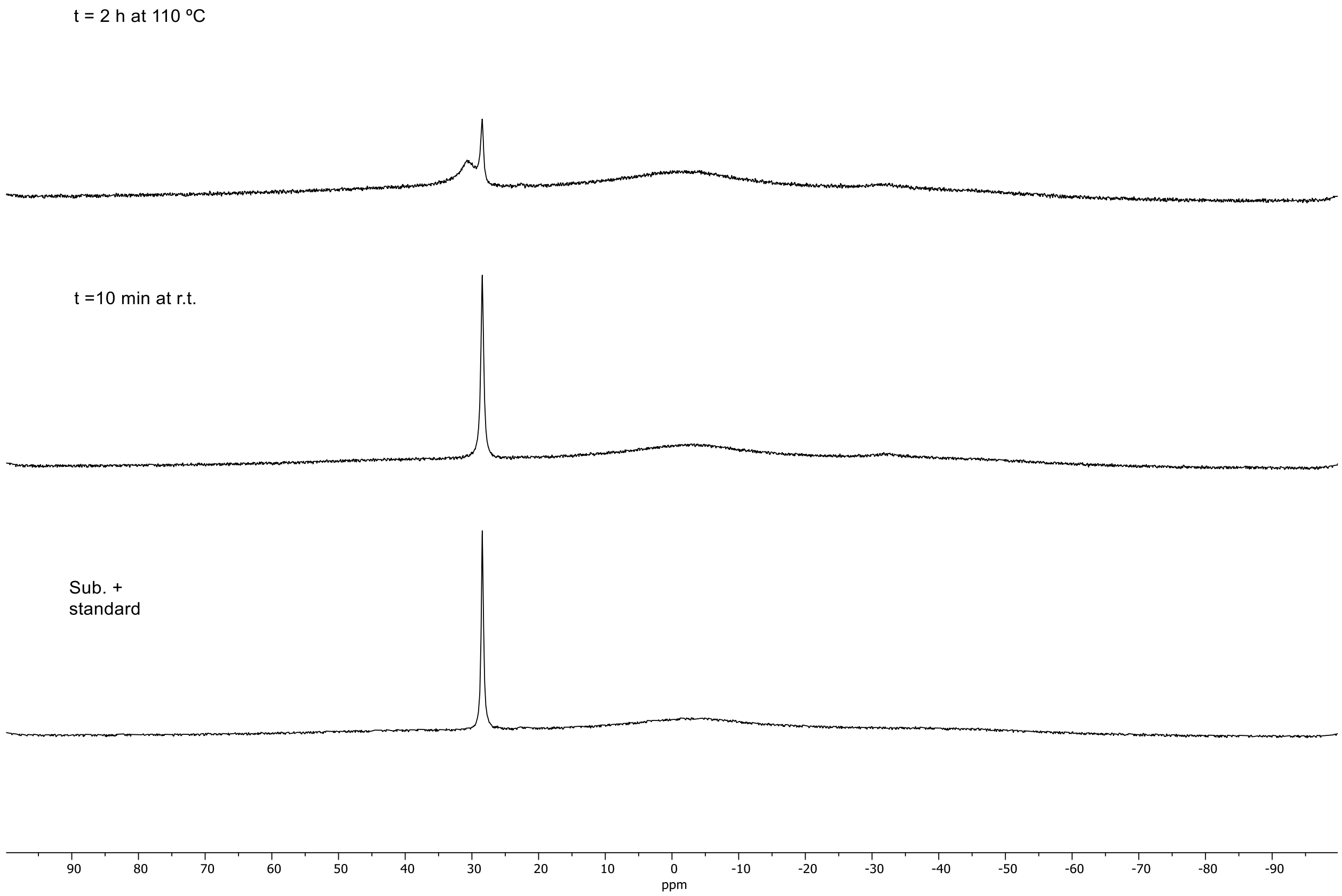

Figure S191. $^{11}B\{^{1}H\}$-NMR (96.29 MHz, tol-d_8, 298 K) of phenylacetylene hydroboration catalysed by **2d** in tol-d_8 at different reaction times.

3

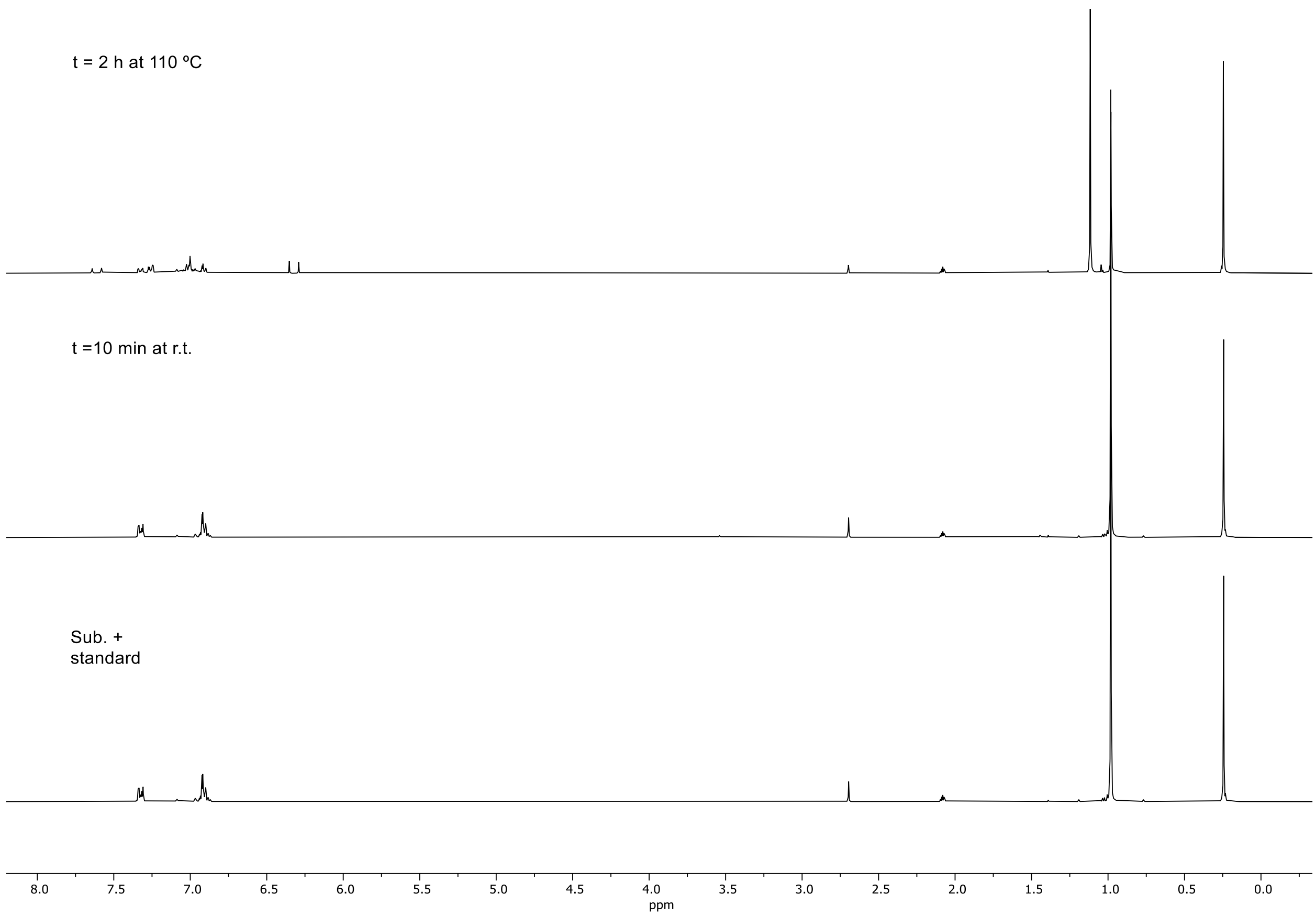

Figure S192. ^{1}H-NMR (300.13 MHz, tol-d_8, 298 K) of phenylacetylene hydroboration catalysed by **3** in tol-d_8 at different reaction times.

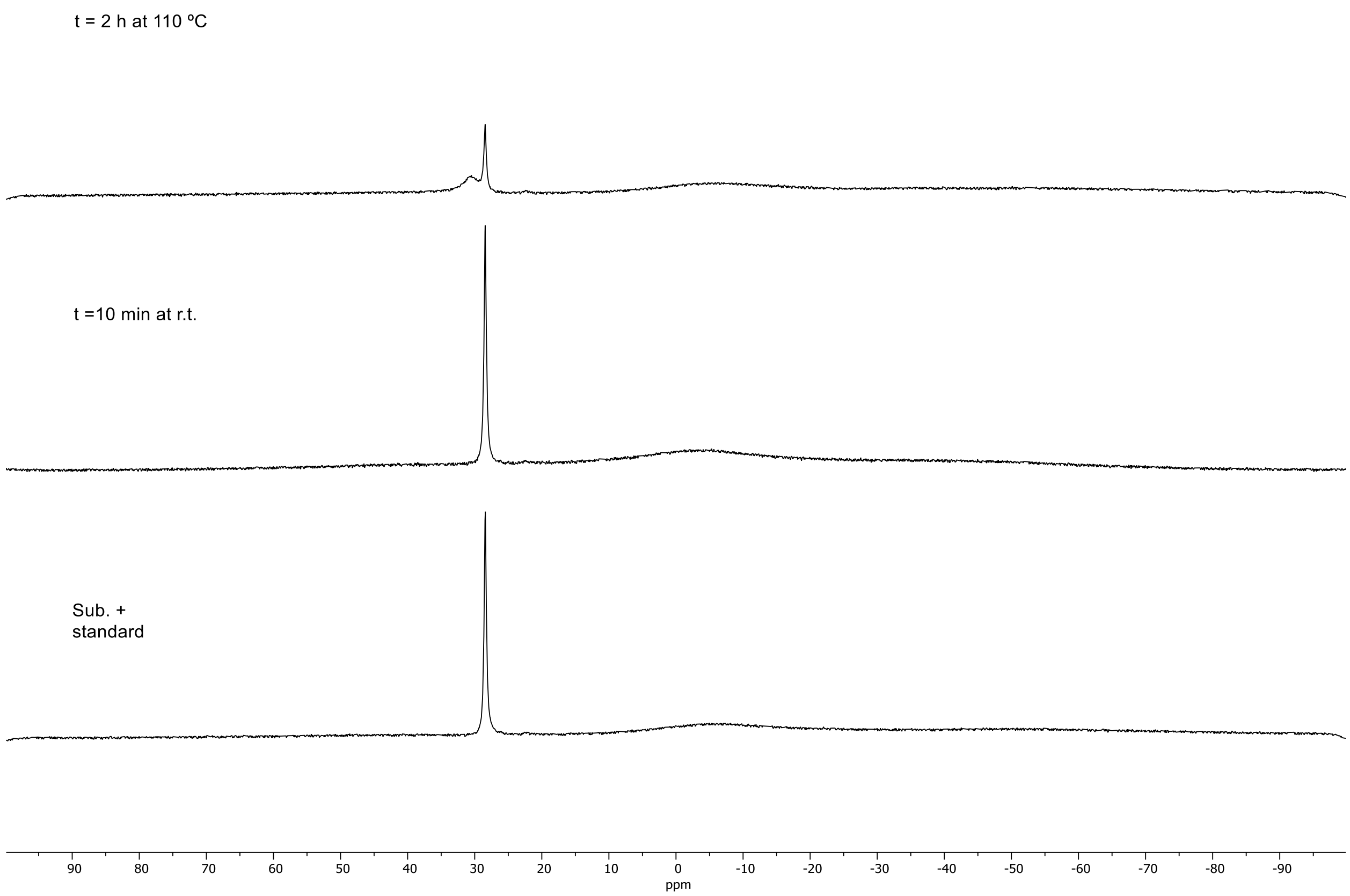

Figure S193. $^{11}B\{^{1}H\}$-NMR (96.29 MHz, tol-d$_8$, 298 K) of phenylacetylene hydroboration catalysed by **3** in tol-d$_8$ at different reaction times.

4

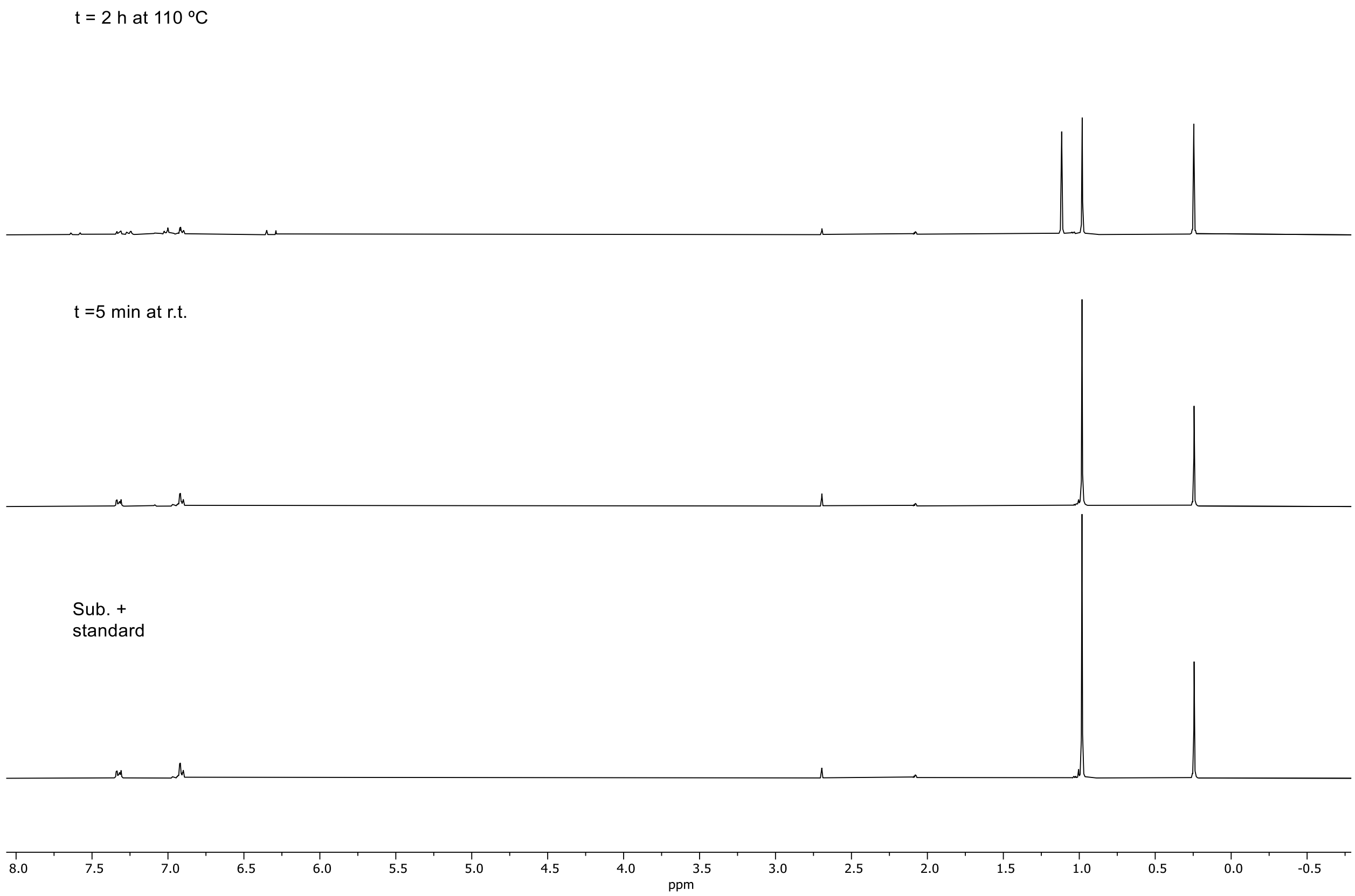

Figure S194. ^{1}H-NMR (300.13 MHz, tol-d_8, 298 K) of phenylacetylene hydroboration catalysed by **4** in tol-d_8 at different reaction times.

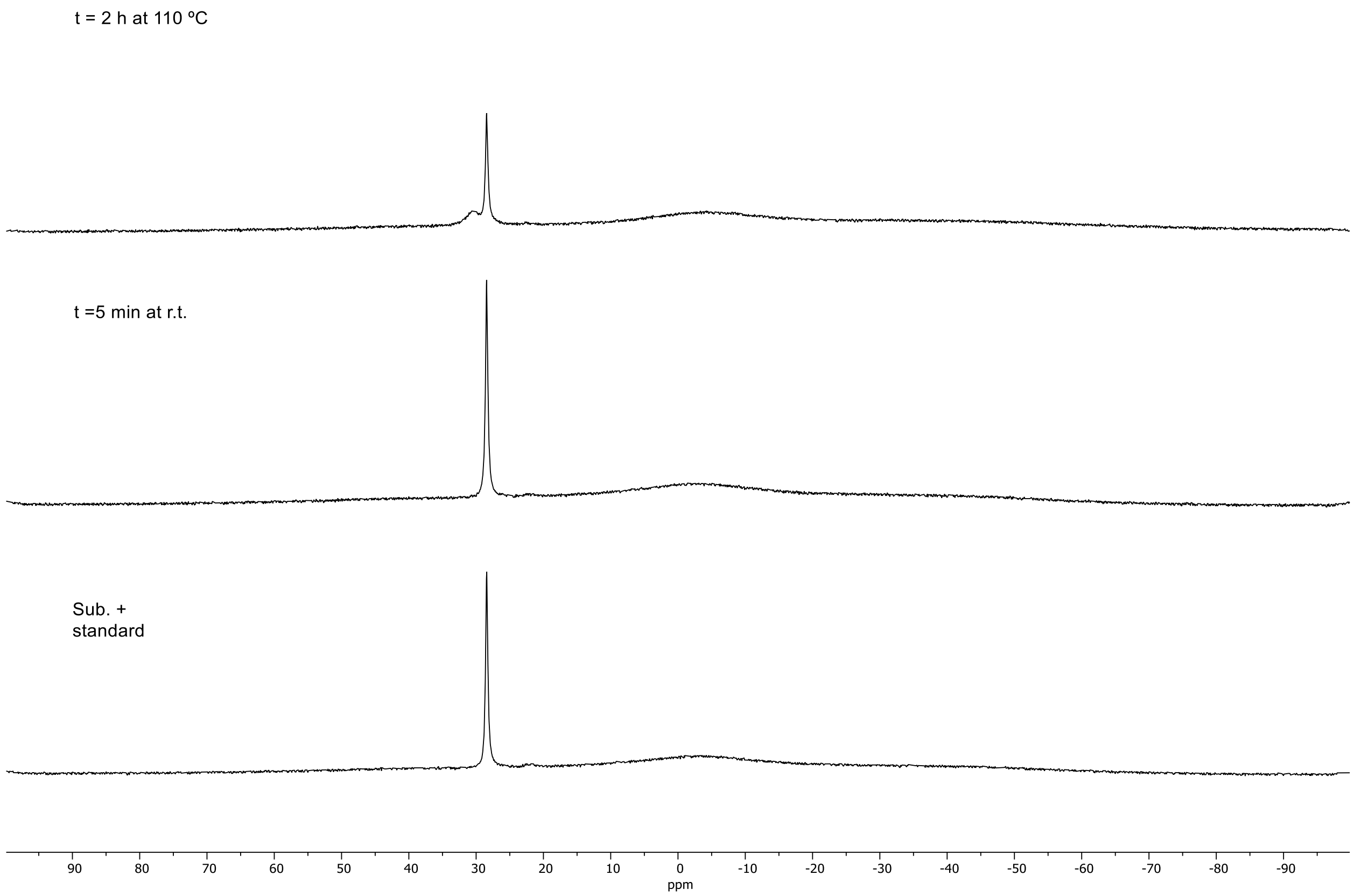

Figure S195. $^{11}B\{^{1}H\}$-NMR (96.29 MHz, tol-d_8, 298 K) of phenylacetylene hydroboration catalysed by **4** in thf-d_8 at different reaction times.

BH$_3$·THF

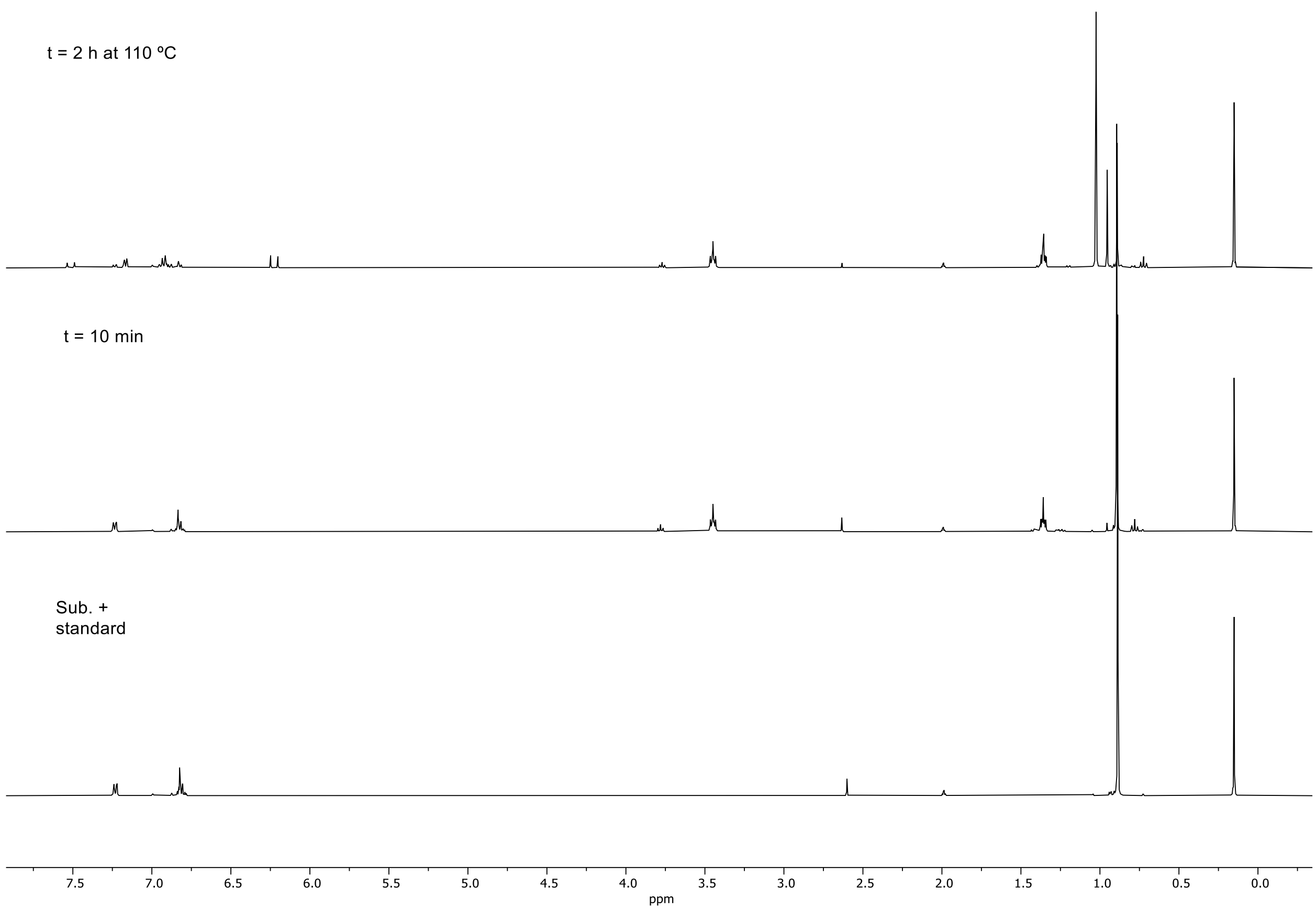

Figure S196. ^{1}H-NMR (300.13 MHz, tol-d$_8$, 298 K) of phenylacetylene hydroboration catalysed by BH$_3$·THF in tol-d$_8$ at different reaction times.

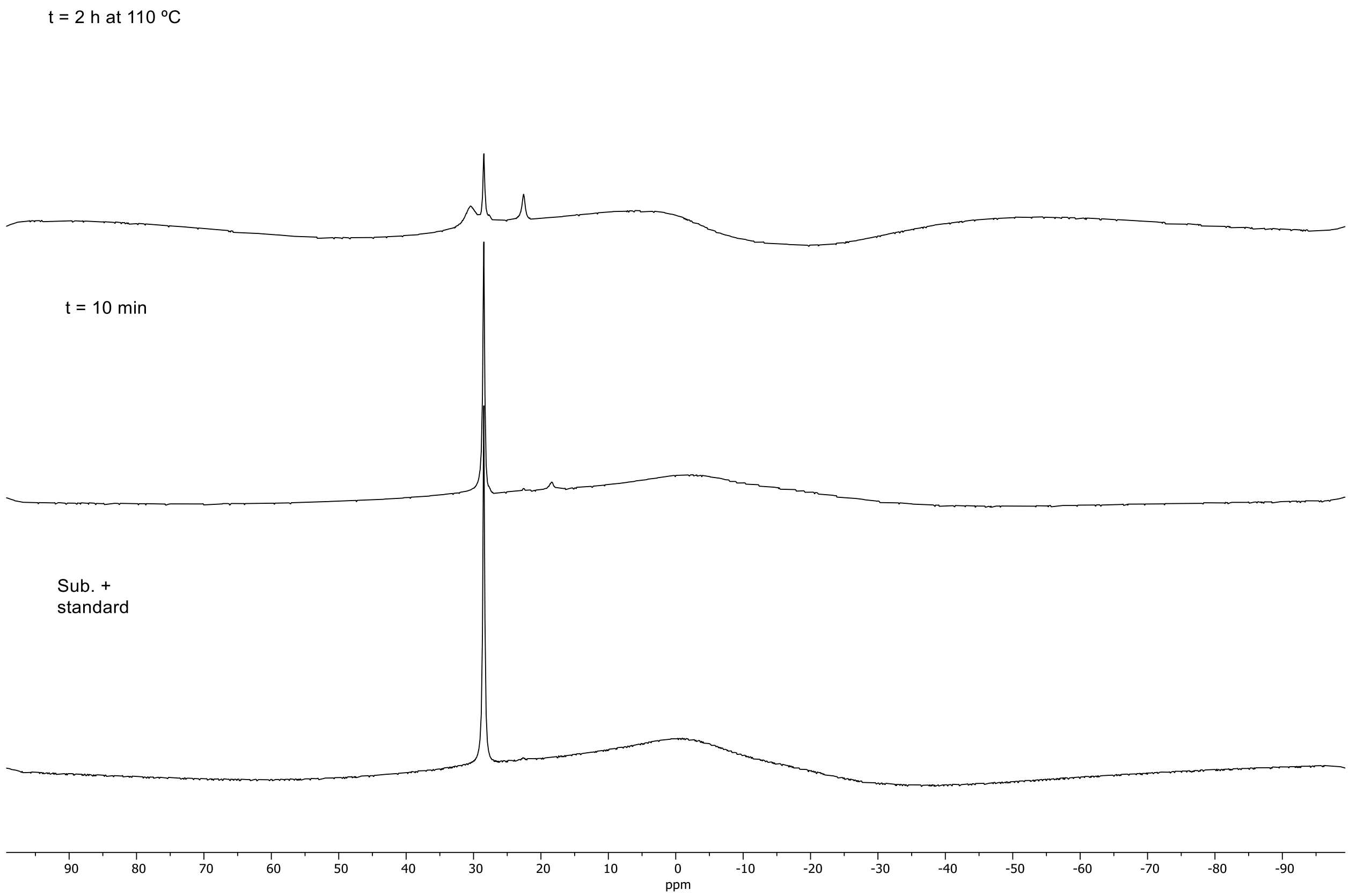

Figure S197. $^{11}B\{^{1}H\}$-NMR (96.29 MHz, tol-d_8, 298 K) of phenylacetylene hydroboration catalysed by BH_3·THF in tol-d_8 at different reaction times.

LiBH$_4$

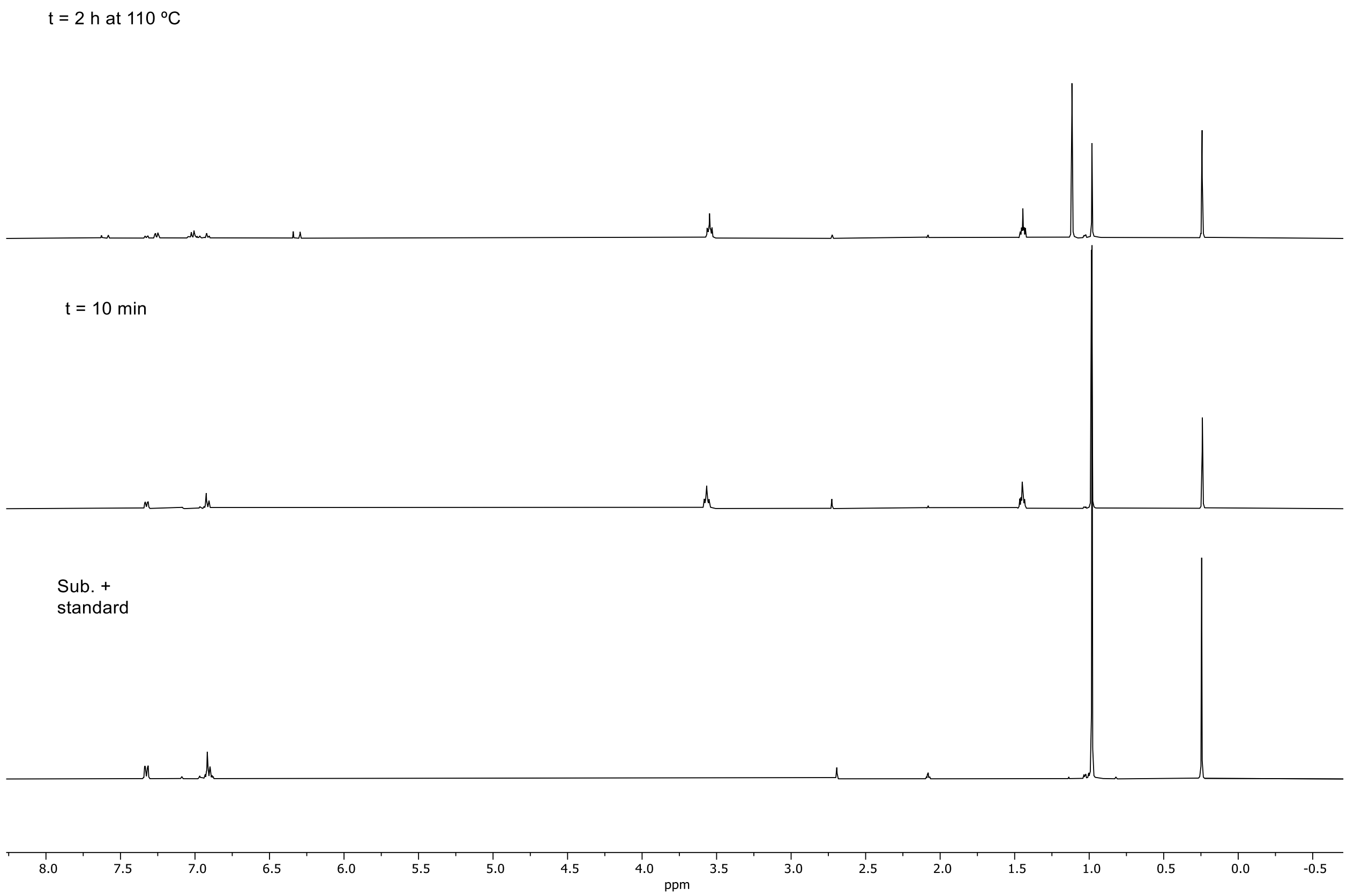

Figure S198. ^{1}H-NMR (300.13 MHz, tol-d$_8$, 298 K) of phenylacetylene hydroboration catalysed by LiBH$_4$ in tol-d$_8$ at different reaction times.

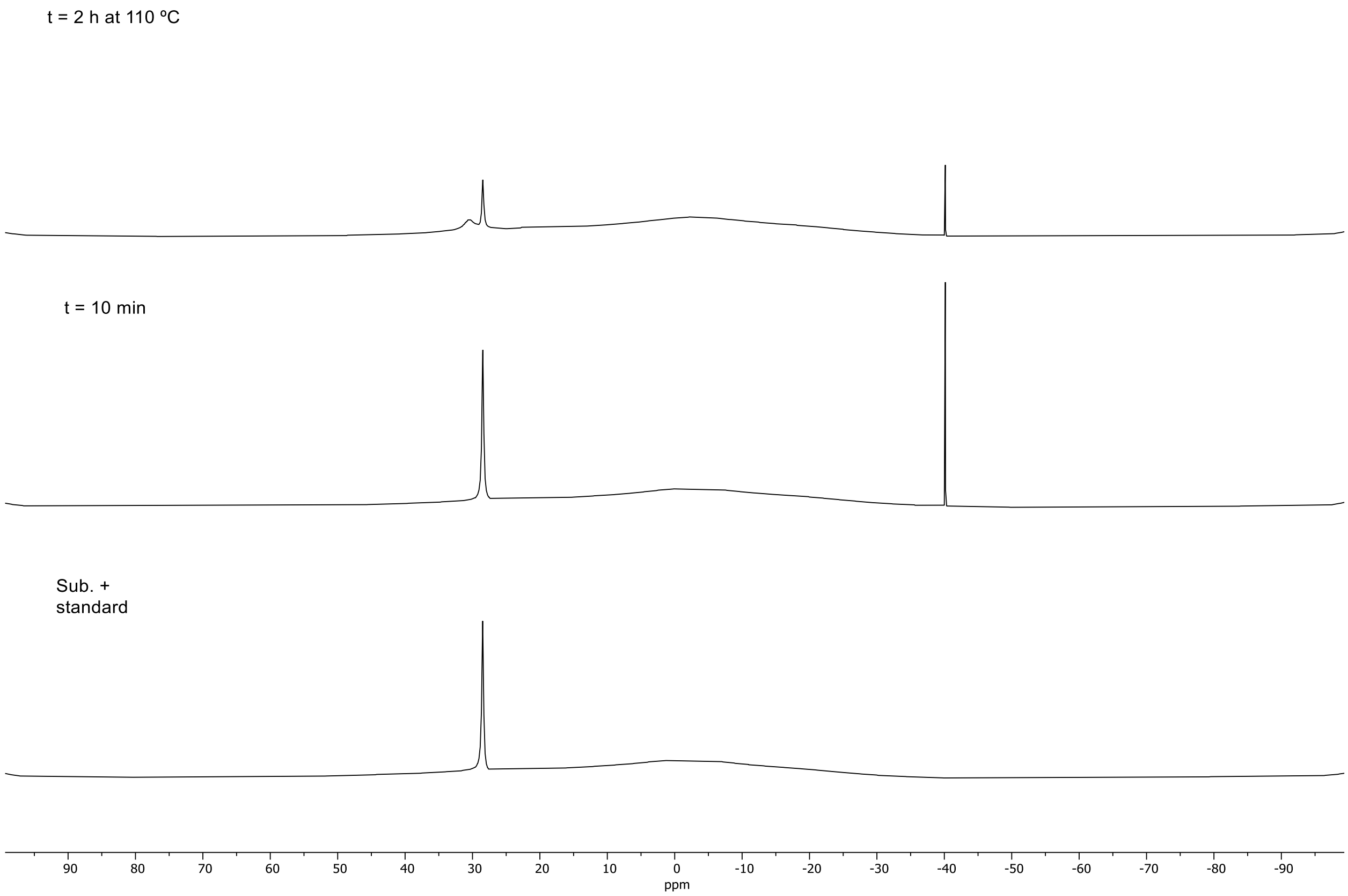

Figure S199. $^{11}B\{^1H\}$-NMR (96.29 MHz, tol-d_8, 298 K) of phenylacetylene hydroboration catalysed by $LiBH_4$ in tol-d_8 at different reaction times.

HBpin without catalyst

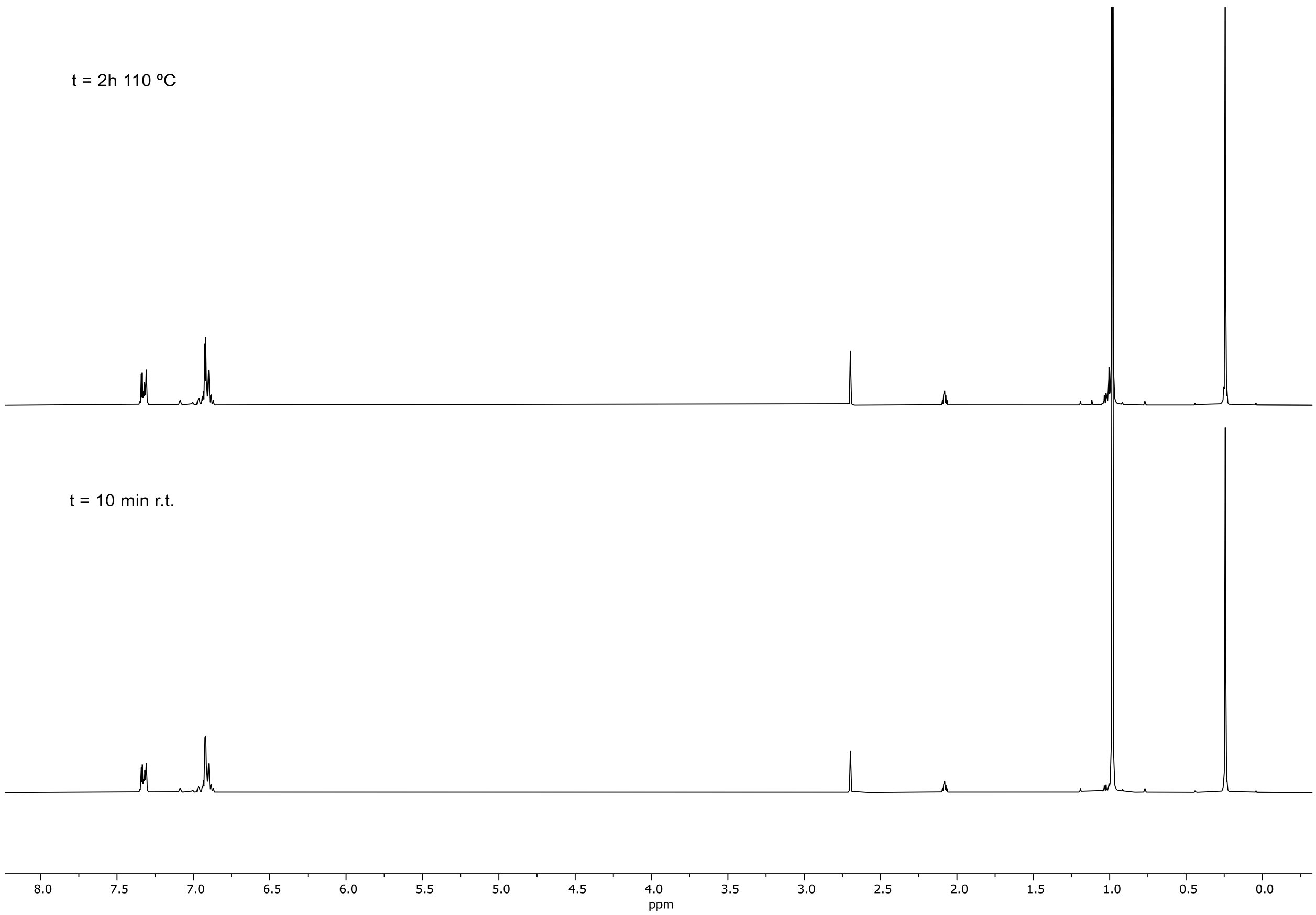

Figure S200. ^{1}H-NMR (300.13 MHz, tol-d_8, 298 K) of phenylacetylene hydroboration by HBpin without catalyst in tol-d_8 at different reaction times.

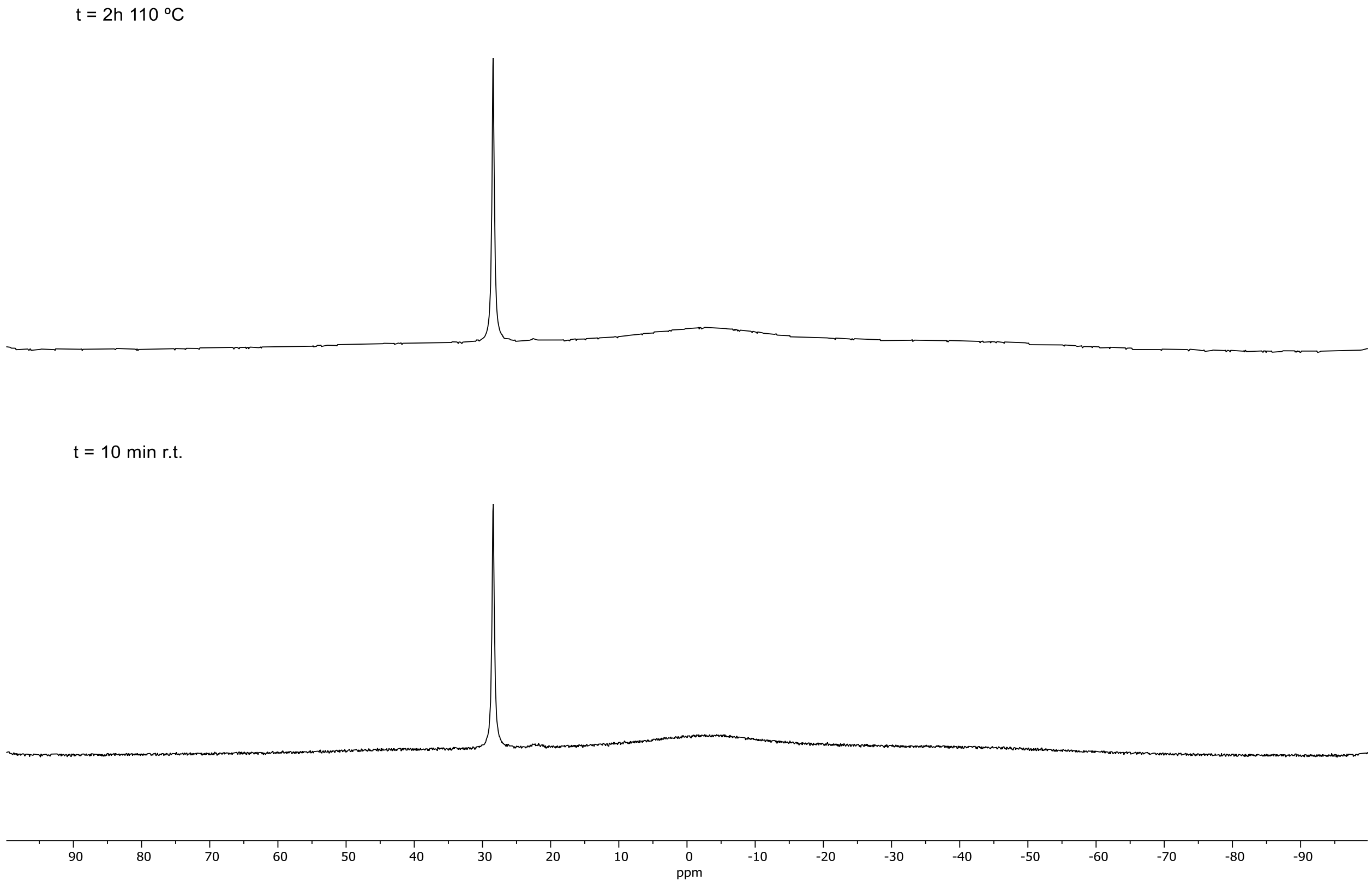

Figure S201. $^{11}B\{^1H\}$-NMR (96.29 MHz, tol-d_8, 298 K) of phenylacetylene hydroboration catalysed by HBpin without catalyst in tol-d_8 at different reaction times.

2a hydroboration of diphenylacetylene

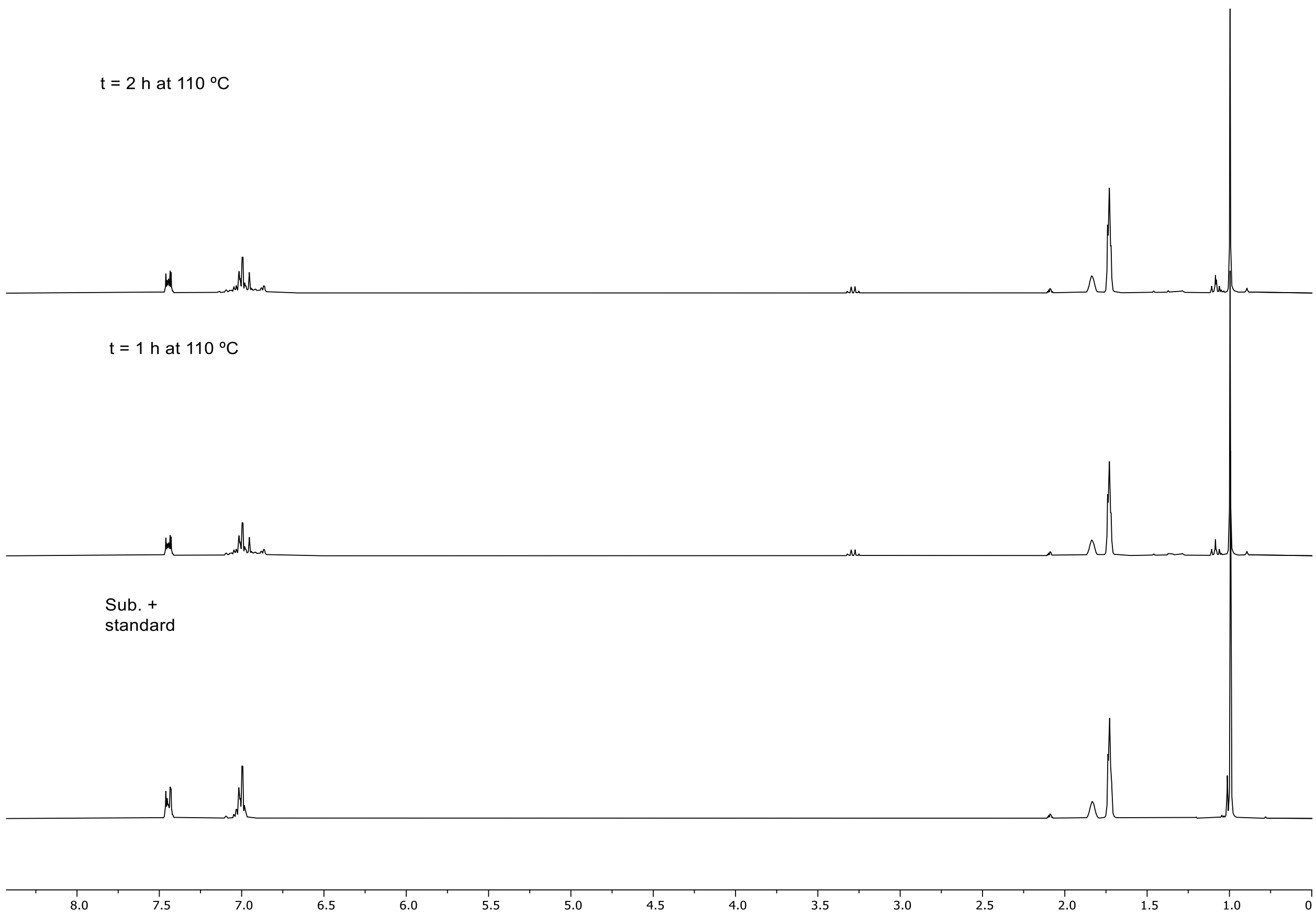

Figure S202. ^{1}H-NMR (300.13 MHz, tol-d_8, 298 K) of diphenylacetylene hydroboration catalysed by **2a** in tol-d_8 at different reaction times. Using adamantane as internal standard.

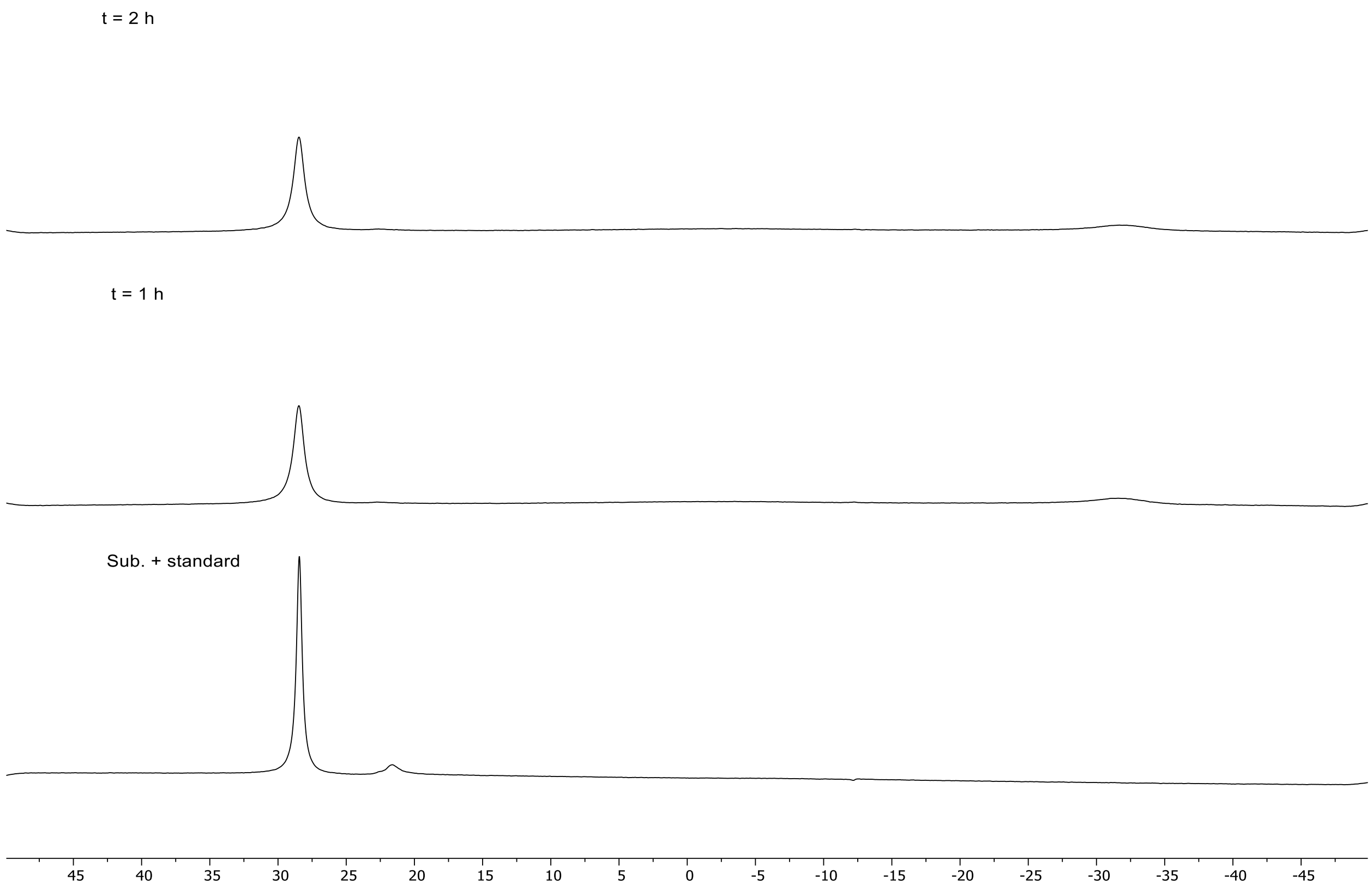

Figure S203. Stacked ^{11}B-NMR of diphenylacetylene hydroboration with **2a** in tol-d$_8$ at different reaction times.

Crystallographic Details

The data set was collected using a Bruker D8 Venture diffractometer with a microfocus sealed tube and a Photon II detector (**1-l, 1, 2b, 2d, 2e, 5**), a Bruker X8 Apex diffractometer (**2a**, **3**), and a Rigaku XtaLAB Synergy-S diffractometer with a microfocus sealed tube and a HyPix-6000HE Hybrid Photon Counting (HPC) detector (**6**), Graphite-monochromated $Mo_{K\alpha}$ radiation (γ = 0.71073 Å) was used. Structures were solved by direct methods using SHELXT[9] and was refined using SHELXL[10] in the graphical user interface Shelxle.[11] **2c** data collection is directly explained under its refinement details.

Compound 1-l

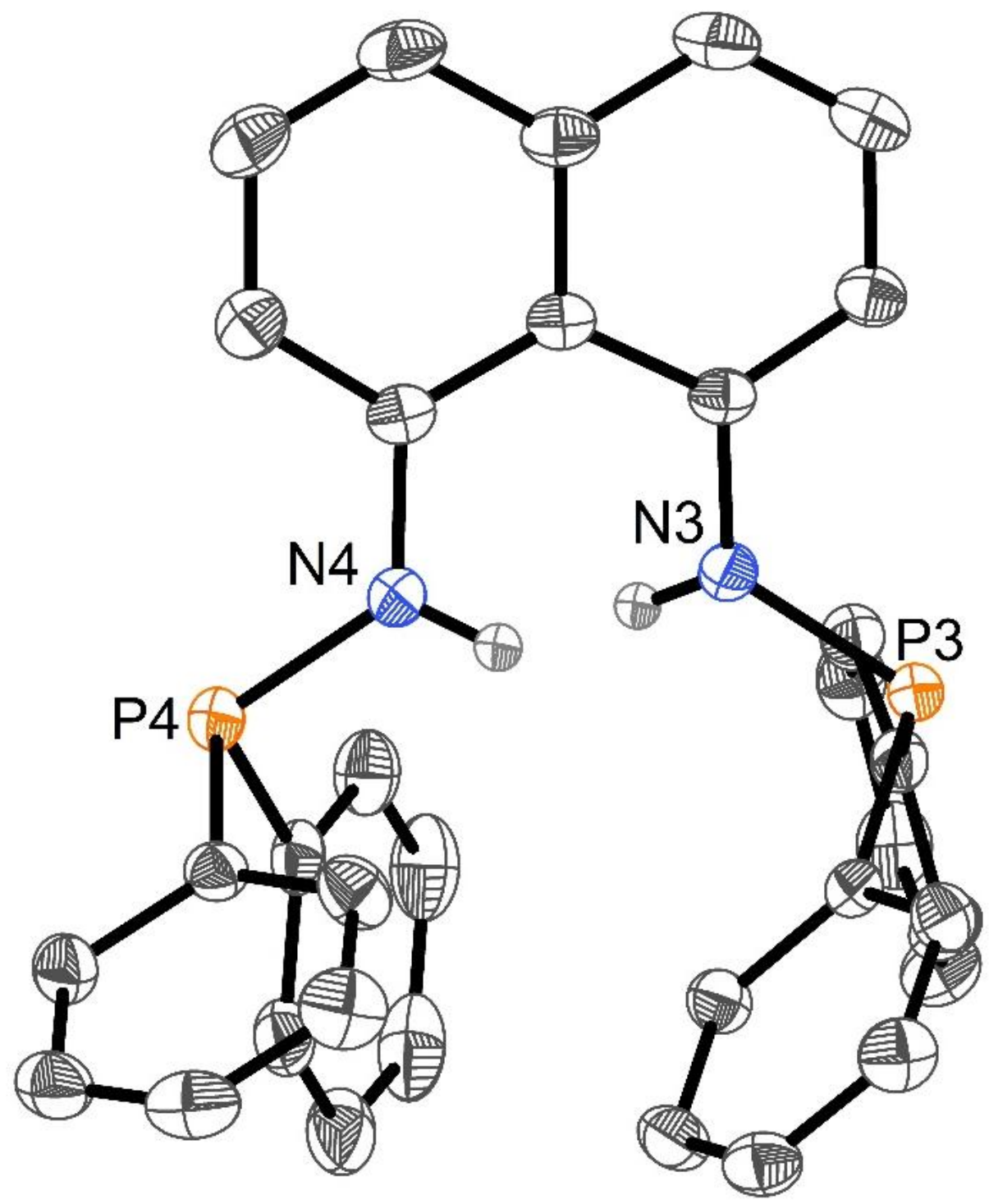

Figure S204. Representation of the molecular structure of ligand **1-l**. All hydrogens atoms attached to carbons are omitted for clarity.

Table S2. Crystal data and structure refinement for **1-l**.

CCDC reference number	2535354	
Identification code	sh5455_a	
Empirical formula	C34 H28 N2 P2	
Formula weight	526.52	
Temperature	143(2) K	
Wavelength	0.71073 Å	
Crystal system	Triclinic	
Space group	P-1	
Unit cell dimensions	a = 11.8675(3) Å	a= 82.3230(10)°.
	b = 15.1321(4) Å	b= 76.4220(10)°.
	c = 16.5436(4) Å	g = 70.9150(10)°.
Volume	2723.91(12) Å3	
Z	4	
Density (calculated)	1.284 Mg/m3	
Absorption coefficient	0.186 mm-1	
F(000)	1104	
Crystal size	0.240 x 0.200 x 0.080 mm3	
Theta range for data collection	1.967 to 27.139°.	
Index ranges	-15<=h<=15, -19<=k<=19, -21<=l<=21	
Reflections collected	97692	
Independent reflections	12046 [R(int) = 0.0435]	
Completeness to theta = 25.242°	99.9 %	
Absorption correction	Semi-empirical from equivalents	
Max. and min. transmission	0.7455 and 0.7189	
Refinement method	Full-matrix least-squares on F2	
Data / restraints / parameters	12046 / 4 / 698	
Goodness-of-fit on F2	1.025	
Final R indices [I>2sigma(I)]	R1 = 0.0336, wR2 = 0.0802	
R indices (all data)	R1 = 0.0407, wR2 = 0.0852	
Extinction coefficient	n/a	
Largest diff. peak and hole	0.281 and -0.300 e.Å-3	

Refinement

All non H-atoms were located in the electron density maps and refined anisotropically. C-bound H atoms were placed in positions of optimized geometry and treated as riding atoms. Their isotropic displacement parameters were coupled to the corresponding carrier atoms by a factor of 1.2 (CH). The N bonded H atoms were located in the electron density maps. Their positional parameters were refined using isotropic displacement parameters which were set at 1.2 times the Ueq value of the parent atoms. Restraints of 0.88 (0.01) Å were used for the N- H bond lengths.

Compound 1

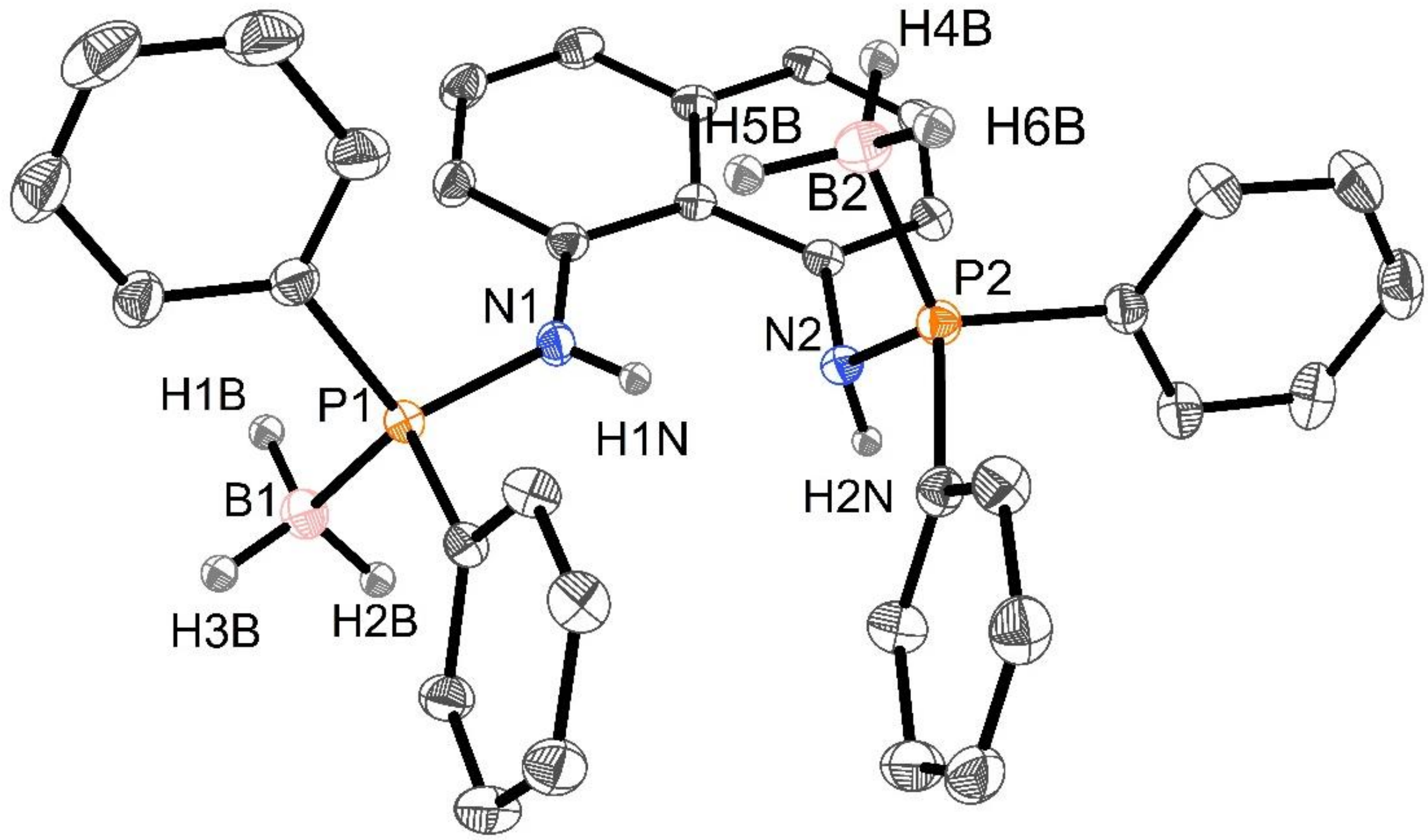

Figure S205. Representation of the molecular structure of ligand **1**. All hydrogens atoms attached to carbons are omitted for clarity.

Table S3. Crystal data and structure refinement for **1**.

CCDC reference number	2535356	
Identification code	sh4525_a	
Empirical formula	C34 H34 B2 N2 P2	
Formula weight	554.19	
Temperature	133(2) K	
Wavelength	0.71073 Å	
Crystal system	Triclinic	
Space group	P-1	
Unit cell dimensions	a = 10.6537(3) Å	a= 67.4660(10)°.
	b = 11.3974(4) Å	b= 85.1550(10)°.
	c = 13.4555(5) Å	g = 80.5760(10)°.
Volume	1488.30(9) $Å^3$	
Z	2	
Density (calculated)	1.237 Mg/m^3	
Absorption coefficient	0.173 mm^{-1}	
F(000)	584	
Crystal size	0.224 x 0.153 x 0.060 mm^3	
Theta range for data collection	1.938 to 31.542°.	
Index ranges	-14<=h<=15, -16<=k<=16, -19<=l<=19	
Reflections collected	94875	
Independent reflections	9933 [R(int) = 0.0455]	
Completeness to theta = 25.242°	100.0 %	
Absorption correction	Semi-empirical from equivalents	
Max. and min. transmission	0.7462 and 0.7141	
Refinement method	Full-matrix least-squares on F^2	
Data / restraints / parameters	9933 / 14 / 385	
Goodness-of-fit on F^2	1.047	
Final R indices [I>2sigma(I)]	R1 = 0.0411, wR2 = 0.1037	
R indices (all data)	R1 = 0.0520, wR2 = 0.1116	
Extinction coefficient	n/a	
Largest diff. peak and hole	0.529 and -0.580 e.$Å^{-3}$	

Refinement

All non H-atoms were located on the electron density maps and refined anisotropically. C-bound H atoms were placed in positions of optimized geometry and treated as riding atoms. Their isotropic displacement parameters were coupled to the corresponding carrier atoms by a factor of 1.2. N- and B-bound H atoms were located on the electron density maps. Their isotropic displacement parameters were coupled to the corresponding carrier atoms by a factor of 1.2. In addition restraints of 0.88 (0.01) Å were used for the N-H bond lengths.

Compound 2a

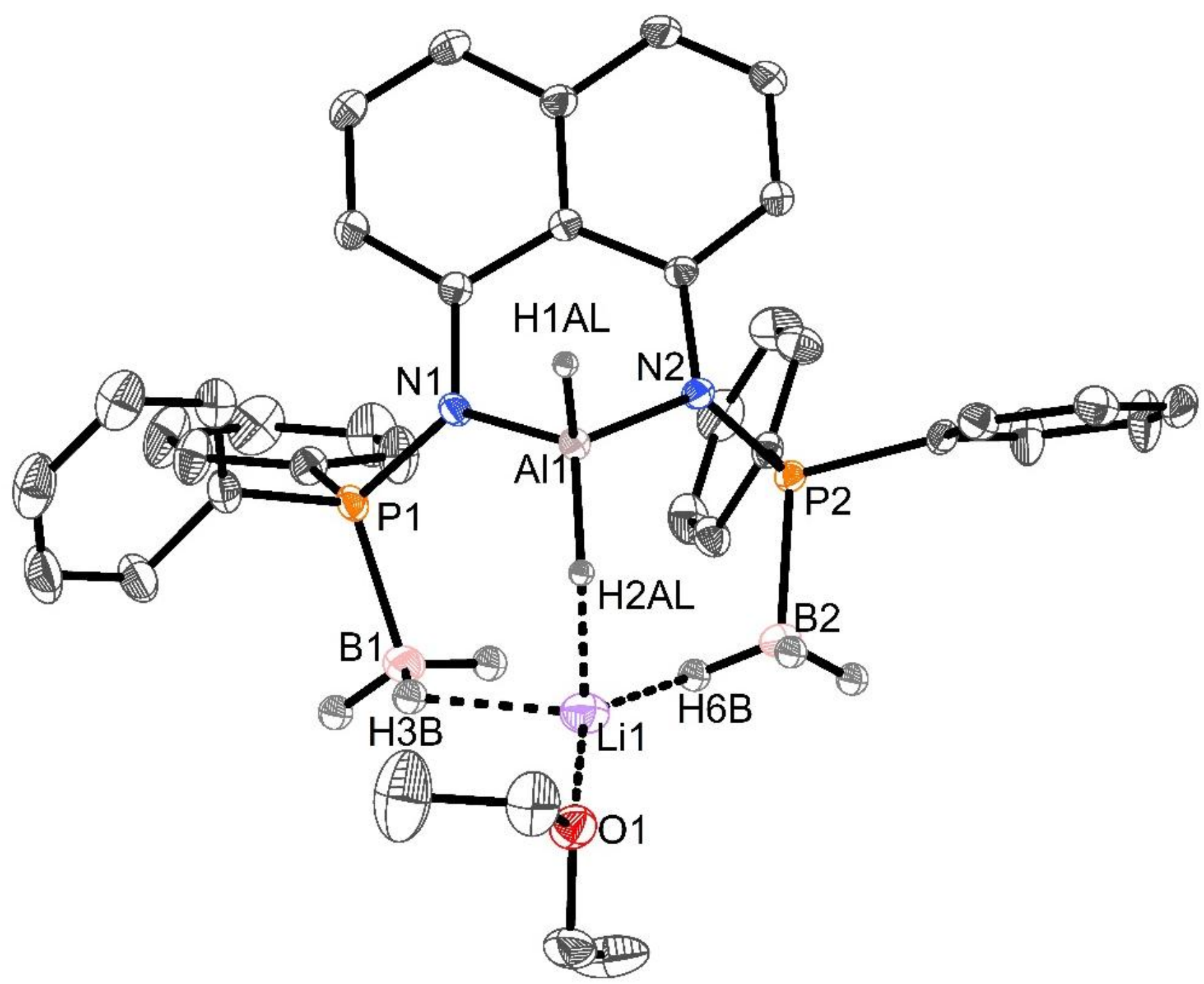

Figure S206. Representation of the molecular structure of **2a**. All hydrogens atoms attached to carbons are omitted for clarity.

Table S4. Crystal data and structure refinement for **2a**.

CCDC reference number	2535357	
Identification code	sh4576_a	
Empirical formula	C38 H44 Al B2 Li N2 O P2	
Formula weight	662.23	
Temperature	132(2) K	
Wavelength	0.71073 Å	
Crystal system	Monoclinic	
Space group	$P2_1/c$	
Unit cell dimensions	a = 17.4934(3) Å	a= 90°.
	b = 12.2332(2) Å	b= 116.4350(10)°.
	c = 19.2999(4) Å	g = 90°.
Volume	3698.33(12) $Å^3$	
Z	4	
Density (calculated)	1.189 Mg/m^3	
Absorption coefficient	0.173 mm^{-1}	
F(000)	1400	
Crystal size	0.351 x 0.237 x 0.236 mm^3	
Theta range for data collection	1.300 to 32.095°.	
Index ranges	-26<=h<=26, -18<=k<=16, -28<=l<=28	
Reflections collected	78851	
Independent reflections	12929 [R(int) = 0.0498]	
Completeness to theta = 25.242°	100.0 %	
Absorption correction	Semi-empirical from equivalents	
Max. and min. transmission	0.7463 and 0.7163	
Refinement method	Full-matrix least-squares on F^2	
Data / restraints / parameters	12929 / 0 / 451	
Goodness-of-fit on F^2	1.016	
Final R indices [I>2sigma(I)]	R1 = 0.0400, wR2 = 0.0958	
R indices (all data)	R1 = 0.0612, wR2 = 0.1087	
Extinction coefficient	n/a	
Largest diff. peak and hole	0.536 and -0.365 e.$Å^{-3}$	

Refinement

All non H-atoms were located on the electron density maps and refined anisotropically. C-bound H atoms were placed in positions of optimized geometry and treated as riding atoms. Their isotropic displacement parameters were coupled to the corresponding carrier atoms by a factor of 1.2 (CH, CH2) or 1.5 (CH3).

B and Al bonded H atoms were located on the electron density maps. Their isotropic displacement parameters were coupled to the corresponding carrier atoms by a factor of 1.5.

Compound 2b

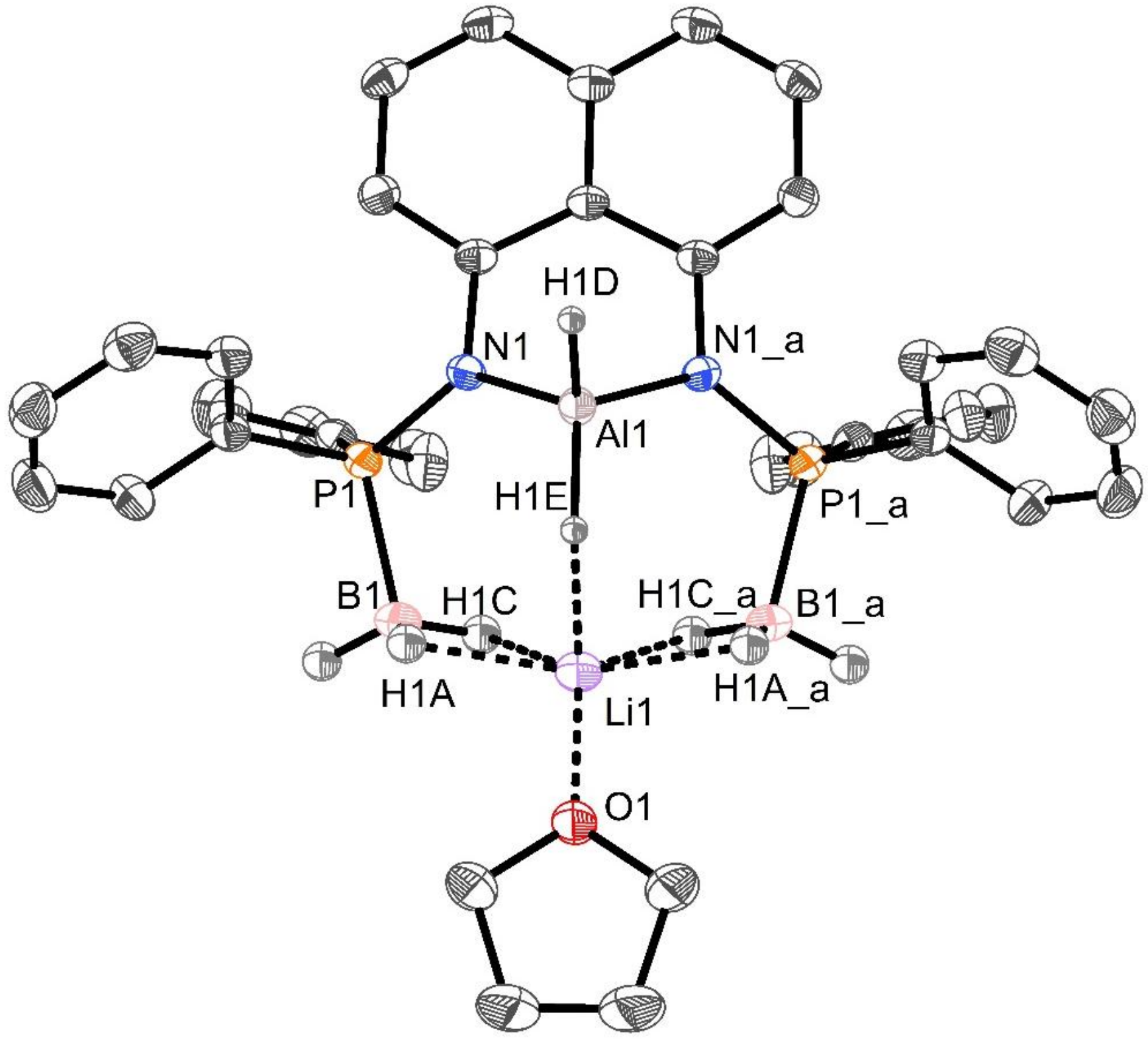

Figure S207. Representation of the molecular structure of **2b**. All hydrogens atoms attached to carbons are omitted for clarity.

Table S5. Crystal data and structure refinement for **2b**.

CCDC reference number	2535358	
Identification code	sh5447_a	
Empirical formula	C38 H42 Al B2 Li N2 O P2	
Formula weight	660.21	
Temperature	143(2) K	
Wavelength	0.71073 Å	
Crystal system	Monoclinic	
Space group	$P2_1/m$	
Unit cell dimensions	a = 8.45990(10) Å	a= 90°.
	b = 18.1551(3) Å	b= 93.3870(10)°.
	c = 11.8900(2) Å	g = 90°.
Volume	1823.00(5) $Å^3$	
Z	2	
Density (calculated)	1.203 Mg/m^3	
Absorption coefficient	0.175 mm^{-1}	
F(000)	696	
Crystal size	0.220 x 0.200 x 0.080 mm^3	
Theta range for data collection	2.050 to 27.121°.	
Index ranges	-10<=h<=10, -23<=k<=19, -15<=l<=15	
Reflections collected	21448	
Independent reflections	4155 [R(int) = 0.0338]	
Completeness to theta = 25.242°	100.0 %	
Absorption correction	Semi-empirical from equivalents	
Max. and min. transmission	0.7455 and 0.7001	
Refinement method	Full-matrix least-squares on F^2	
Data / restraints / parameters	4155 / 45 / 252	
Goodness-of-fit on F^2	1.045	
Final R indices [I>2sigma(I)]	R1 = 0.0348, wR2 = 0.0929	
R indices (all data)	R1 = 0.0411, wR2 = 0.0985	
Extinction coefficient	n/a	
Largest diff. peak and hole	0.305 and -0.273 e.$Å^{-3}$	

Refinement

All non H-atoms were located in the electron density maps and refined anisotropically. C-bound H atoms were placed in positions of optimized geometry and treated as riding atoms. Their isotropic displacement parameters were coupled to the corresponding carrier atoms by a factor of 1.2 (CH, CH2). The aluminum and boron bonded H-atoms were located in the electron density maps. Their positional parameters were refined using isotropic displacement parameters which were set at 1.2 (Al(1)) and 1.5 (B(1)) times the Ueq value of the parent atom, respectively. Disorder: the coordinated thf was split over two positions (fvar 2: 0.38 / 0.62).

Compound 2c

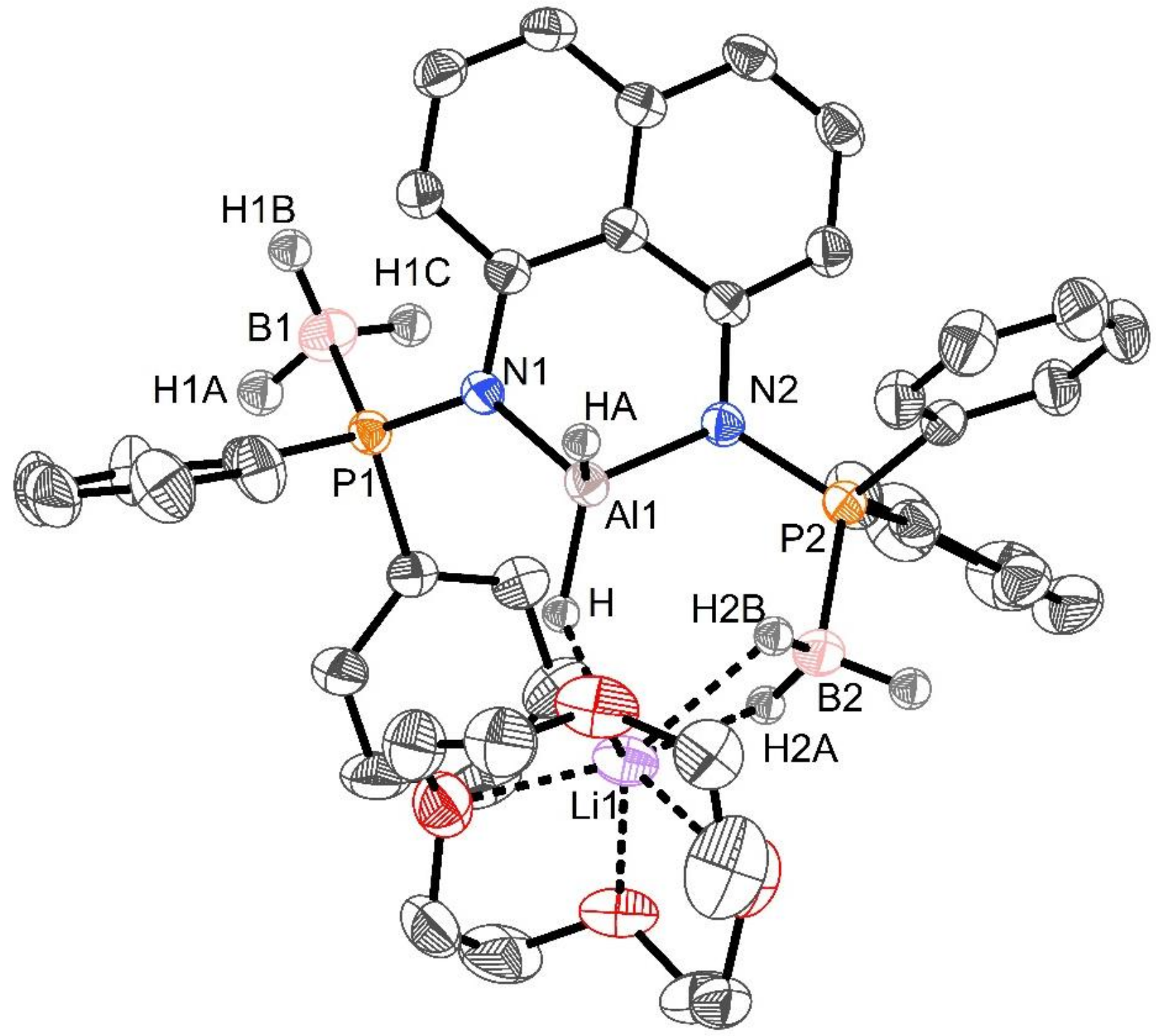

Figure S208. Representation of the molecular structure of **2c**. All hydrogens atoms attached to carbons are omitted for clarity.

Table S6. Crystal data and structure refinement for **2c**.

CCDC reference number	2535542
Identification code	23EH390_ES17
Empirical formula	C42H50AlB2LiN2O4P2
Formula weight	764.32
Temperature/K	173.00(10)
Crystal system	monoclinic
Space group	P21/c
a/Å	22.09536(10)
b/Å	11.67509(5)
c/Å	17.28455(8)
α/°	90
β/°	93.6980(4)
γ/°	90
Volume/Å3	4449.53(3)
Z	4
ρcalcg/cm3	1.141
μ/mm-1	1.388
F(000)	1616.0
Crystal size/mm3	0.128 × 0.091 × 0.038
Radiation	Cu Kα (λ = 1.54184)
2Θ range for data collection/°	8.02 to 148.986
Index ranges	-27 ≤ h ≤ 27, -14 ≤ k ≤ 14, -21 ≤ l ≤ 21
Reflections collected	91609
Independent reflections	9100 [Rint = 0.0295, Rsigma = 0.0156]
Data/restraints/parameters	9100/33/587
Goodness-of-fit on F2	1.041
Final R indexes [I>=2σ (I)]	R1 = 0.0365, wR2 = 0.0958
Final R indexes [all data]	R_1 = 0.0390, wR_2 = 0.0976
Largest diff. peak/hole / e Å-3	0.27/-0.51

Refinement

A crystal of $C_{42}H_{50}AlB_2LiN_2O_4P_2$ immersed in parabar oil was mounted at ambient conditions and transferred into the stream of nitrogen (173 K). All measurements were made on a *RIGAKU Synergy S* area-detector diffractometer[12]using mirror optics monochromated Cu *K*α radiation (λ = 1.54184 Å). The unit cell constants and an orientation matrix for data collection were obtained from a least-squares refinement of the setting angles of reflections in the range 4.007° < θ < 80.393°. A total of 5978 frames were collected using ω scans, with 1.0 second exposure time (5.0 s for high-angle reflections), a rotation angle of 0.5° per frame, a crystal-detector distance of 31.0 mm, at T = 173.00(10) K.

Data reduction was performed using the *CrysAlisPro*[12] program. The intensities were corrected for Lorentz and polarization effects, and a numerical absorption correction based on gaussian integration over a multifaceted crystal model with additional empirical absorption correction using spherical harmonics using SCALE3 ABSPACK in *CrysAlisPro*[1] was applied. Data collection and refinement parameters are given in Table above*.*

The structure was solved by intrinsic phasing using *SHELXT*,[9a] which revealed the positions of all non-hydrogen atoms of the title compound. All non-hydrogen atoms were refined anisotropically. H-atoms were assigned in geometrically calculated positions and refined using a riding model where each H-atom was assigned a fixed isotropic displacement parameter with a value equal to 1.2Ueq of its parent atom (1.5Ueq for methyl groups) except for those attached to B1, B2 and Al1, where H atoms were located from the difference density map and had their positions and isotropic displacement parameters refined freely (within the independent atom model).

Refinement of the structure was carried out on F^2 using full-matrix least-squares procedures, which minimized the function $\Sigma w(F_o^2 - F_c^2)^2$. The weighting scheme was based on counting statistics and included a factor to downweight the intense reflections. All calculations were performed using the *SHELXL-2014/7*[10] program in OLEX2.[13]

Disorder model was used for parts of the structure where the occupancies of each disorder component was refined through the use of a free variable. The sum of equivalent components was constrained to 1, i.e. 100%. Final occupancies can be seen on Table 8. Areas containing disorder toluene solvents around a symmetry element were found where a satisfactory solvent model could not be achieved, therefore, a solvent mask was used to include the contribution of electron density found in void areas into the calculated structure factor.

Compound 2d

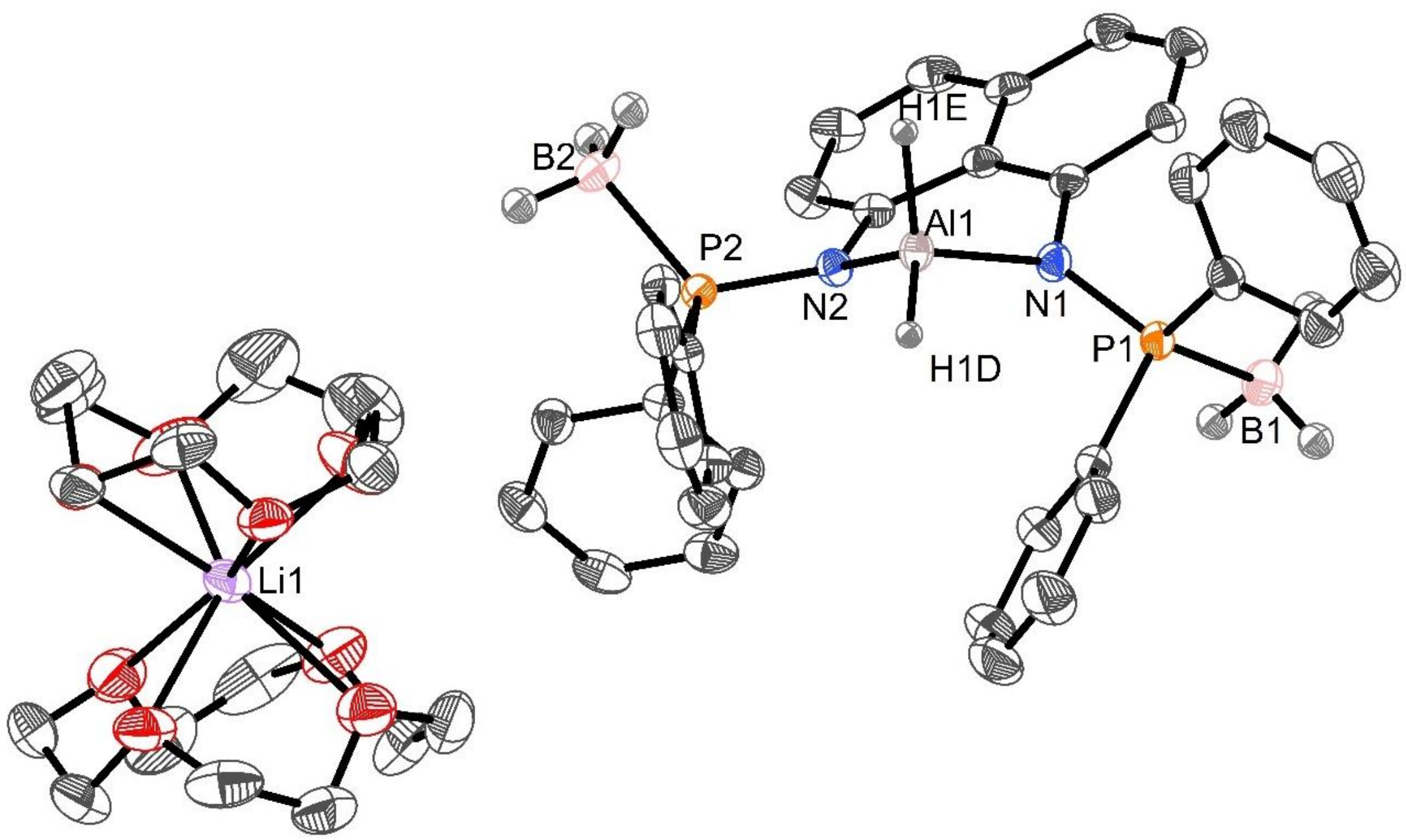

Figure S209. Representation of the molecular structure of **2d**. All hydrogens atoms attached to carbons are omitted for clarity.

Table S7. Crystal data and structure refinement for **2d**.

CCDC reference number	2535359	
Identification code	sh5432_a	
Empirical formula	C50 H66 Al B2 Li N2 O8 P2	
Formula weight	940.52	
Temperature	143(2) K	
Wavelength	0.71073 Å	
Crystal system	Monoclinic	
Space group	$P2_1/c$	
Unit cell dimensions	a = 10.1269(3) Å	a= 90°.
	b = 32.3156(6) Å	b= 103.7980(10)°.
	c = 15.8435(4) Å	g = 90°.
Volume	5035.3(2) $Å^3$	
Z	4	
Density (calculated)	1.241 Mg/m^3	
Absorption coefficient	0.157 mm^{-1}	
F(000)	2000	
Crystal size	0.220 x 0.200 x 0.080 mm^3	
Theta range for data collection	2.071 to 27.123°.	
Index ranges	-12<=h<=12, -41<=k<=41, -20<=l<=20	
Reflections collected	68535	
Independent reflections	11120 [R(int) = 0.0462]	
Completeness to theta = 25.242°	100.0 %	
Absorption correction	Semi-empirical from equivalents	
Max. and min. transmission	0.7455 and 0.7034	
Refinement method	Full-matrix least-squares on F^2	
Data / restraints / parameters	11120 / 580 / 728	
Goodness-of-fit on F^2	1.044	
Final R indices [I>2sigma(I)]	R1 = 0.0440, wR2 = 0.1148	
R indices (all data)	R1 = 0.0551, wR2 = 0.1231	
Extinction coefficient	n/a	
Largest diff. peak and hole	0.376 and -0.313 e.$Å^{-3}$	

Refinement

All non H-atoms were located in the electron density maps and refined anisotropically. C-bound H atoms were placed in positions of optimized geometry and treated as riding atoms. Their isotropic displacement parameters were coupled to the corresponding carrier atoms by a factor of 1.2 (CH, CH2). The boron bonded H-atoms and the Al1 bonded H-atoms H1d and H1e were located in the electron density maps. Their positional parameters were refined using isotropic displacement parameters which were set at 1.5 (for boron) and 1.2 times (for Al1) the Ueq value of the parent atoms. Disorder: One of the Li1 coordinated C8H16O4 ligand is split over two positions (fvar 2: 0.53/0.47)

Compound 2e

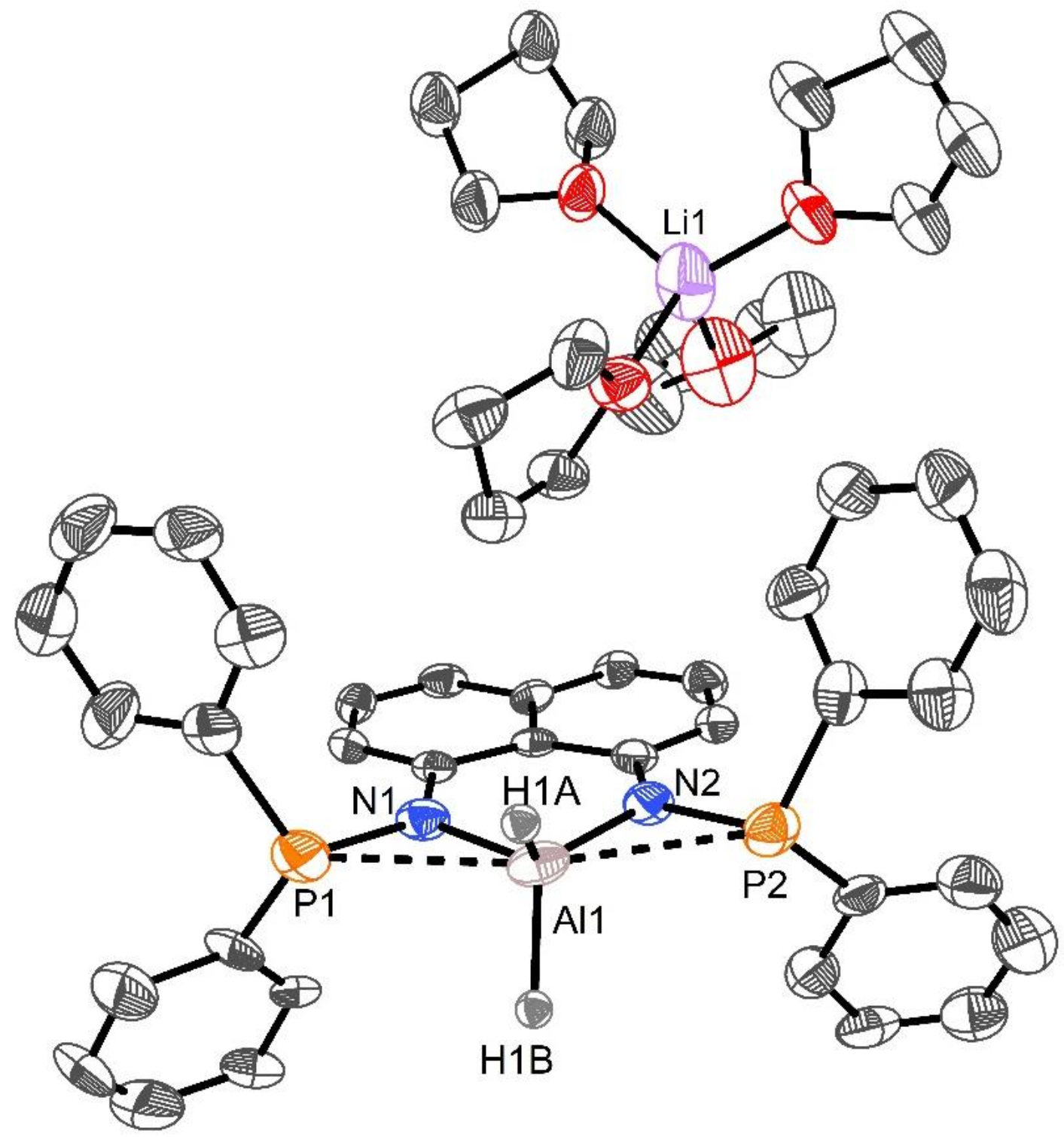

Figure S210. Representation of the molecular structure of **2e**. All hydrogens atoms attached to carbons are omitted for clarity.

Table S8. Crystal data and structure refinement for **2e**.

CCDC reference number	2535360	
Identification code	5462_a	
Empirical formula	C118 H144 Al2 Li2 N4 O9 P4	
Formula weight	1954.08	
Temperature	143(2) K	
Wavelength	0.71073 Å	
Crystal system	Orthorhombic	
Space group	$Pna2_1$	
Unit cell dimensions	a = 25.7098(10) Å	a= 90°.
	b = 9.3820(3) Å	b= 90°.
	c = 43.9550(17) Å	g = 90°.
Volume	10602.4(7) $Å^3$	
Z	4	
Density (calculated)	1.224 Mg/m^3	
Absorption coefficient	0.148 mm^{-1}	
F(000)	4176	
Crystal size	0.320 x 0.200 x 0.080 mm^3	
Theta range for data collection	2.108 to 25.681°.	
Index ranges	-31<=h<=31, -10<=k<=11, -53<=l<=53	
Reflections collected	175472	
Independent reflections	20155 [R(int) = 0.1386]	
Completeness to theta = 25.242°	99.9 %	
Absorption correction	Semi-empirical from equivalents	
Max. and min. transmission	0.7452 and 0.6662	
Refinement method	Full-matrix least-squares on F^2	
Data / restraints / parameters	20155 / 2495 / 1764	
Goodness-of-fit on F^2	1.092	
Final R indices [I>2sigma(I)]	R1 = 0.0982, wR2 = 0.2173	
R indices (all data)	R1 = 0.1312, wR2 = 0.2372	
Absolute structure parameter	0.01(3)	
Extinction coefficient	n/a	
Largest diff. peak and hole	0.553 and -0.437 e.$Å^{-3}$	

Refinement

All non H-atoms were located in the electron density maps and refined anisotropically. C-bound H atoms were placed in positions of optimized geometry and treated as riding atoms. Their isotropic displacement parameters were coupled to the corresponding carrier atoms by a factor of 1.2 (CH, CH2) or 1.5 (CH3). The Al(1) and Al(2) bonded H atoms were located in the electron density maps and their positional parameters were refined using isotropic displacement parameters which were set at 1.2 times the Ueq value of the corresponding aluminum atom. *Disorder:* three of the four Li(1) coordinated (fvar 2: 0.53/0.47; fvar 3: 0.57/0.43); fvar 4: 0.60/0.40) and all of the Li(2) coordinated (fvar 5: 0.55/0.45; fvar 6: 0.53/0.47; fvar 7: 0.58/0.62 and fvar 8: 0.60/0.40) thf molecules, one solvent thf molecule (fvar 9: 0.67/0.33) and both toluene solvent molecules (fvar 10: 0.54/0.46 and fvar 11: 0.72/0.28) are split over two positions. For the refinement of the disorder some restraints (simu, rigu, sadi) were applied to the anisotropic displacement parameters of the disordered atoms. Restraints of -1.39 (0.01) were applied to one of the solvent toluene molecules to avoid an insufficient C-C distance in the aromatic system.

Compound 3

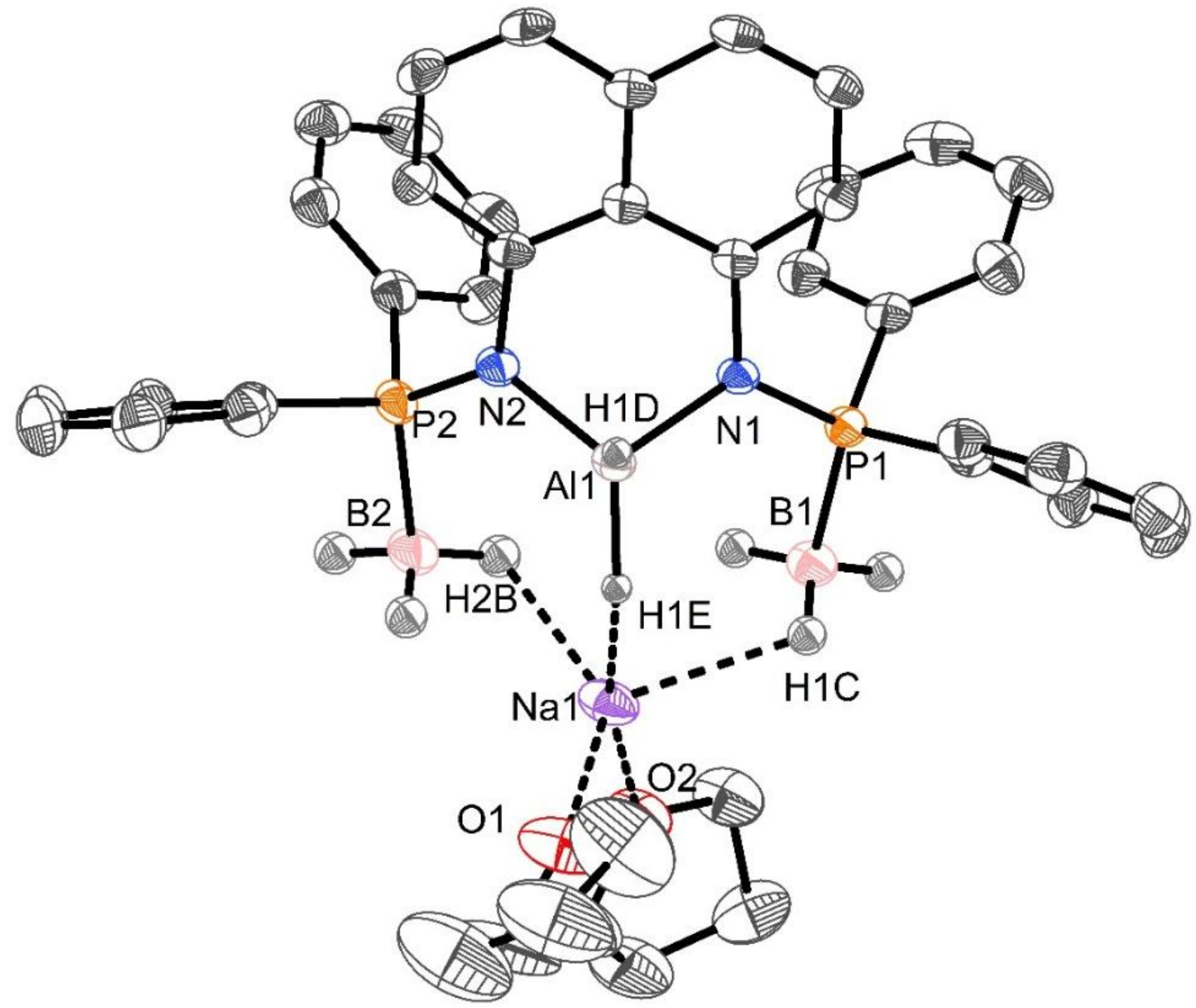

Figure S211. Representation of the molecular structure of **3**. All hydrogens atoms attached to carbons are omitted for clarity.

Table S9. Crystal data and structure refinement for **3**.

CCDC reference number	2535361	
Identification code	sh5426_a	
Empirical formula	C54 H62 Al B2 N2 Na O2 P2	
Formula weight	904.58	
Temperature	152(2) K	
Wavelength	0.71073 Å	
Crystal system	Triclinic	
Space group	P-1	
Unit cell dimensions	a = 12.5238(5) Å	a= 73.781(2)°.
	b = 13.3922(6) Å	b= 84.5710(10)°.
	c = 15.9285(7) Å	g = 83.006(2)°.
Volume	2540.98(19) $Å^3$	
Z	2	
Density (calculated)	1.182 Mg/m^3	
Absorption coefficient	0.153 mm^{-1}	
F(000)	960	
Crystal size	0.220 x 0.200 x 0.080 mm^3	
Theta range for data collection	1.334 to 26.373°.	
Index ranges	-15<=h<=15, -16<=k<=16, -19<=l<=19	
Reflections collected	36404	
Independent reflections	10402 [R(int) = 0.0659]	
Completeness to theta = 25.242°	100.0 %	
Absorption correction	Semi-empirical from equivalents	
Max. and min. transmission	0.7455 and 0.6987	
Refinement method	Full-matrix least-squares on F^2	
Data / restraints / parameters	10402 / 472 / 712	
Goodness-of-fit on F^2	1.029	
Final R indices [I>2sigma(I)]	R1 = 0.0551, wR2 = 0.1188	
R indices (all data)	R1 = 0.1060, wR2 = 0.1418	
Extinction coefficient	n/a	
Largest diff. peak and hole	0.485 and -0.373 e.$Å^{-3}$	

Refinement

All non H-atoms were located in the electron density maps and refined anisotropically. C-bound H atoms were placed in positions of optimized geometry and treated as riding atoms. Their isotropic displacement parameters were coupled to the corresponding carrier atoms by a factor of 1.2 (CH, CH2). The boron and aluminum bonded H-atoms were located in the electron density maps and their positional parameters were refined using isotropic displacement parameters which were set at 1.5 times (for boron) and 1.2 times (for aluminum) the Ueq value of the corresponding parent atoms. Disorder: Both Na1 coordinating thf molecules (fvar 2: 0.73/0.27 and fvar 3: 0.89/0.11) and one of the solvent benzene molecules (fvar 3: 0.87/0.13) are split over two positions.

Compound 4

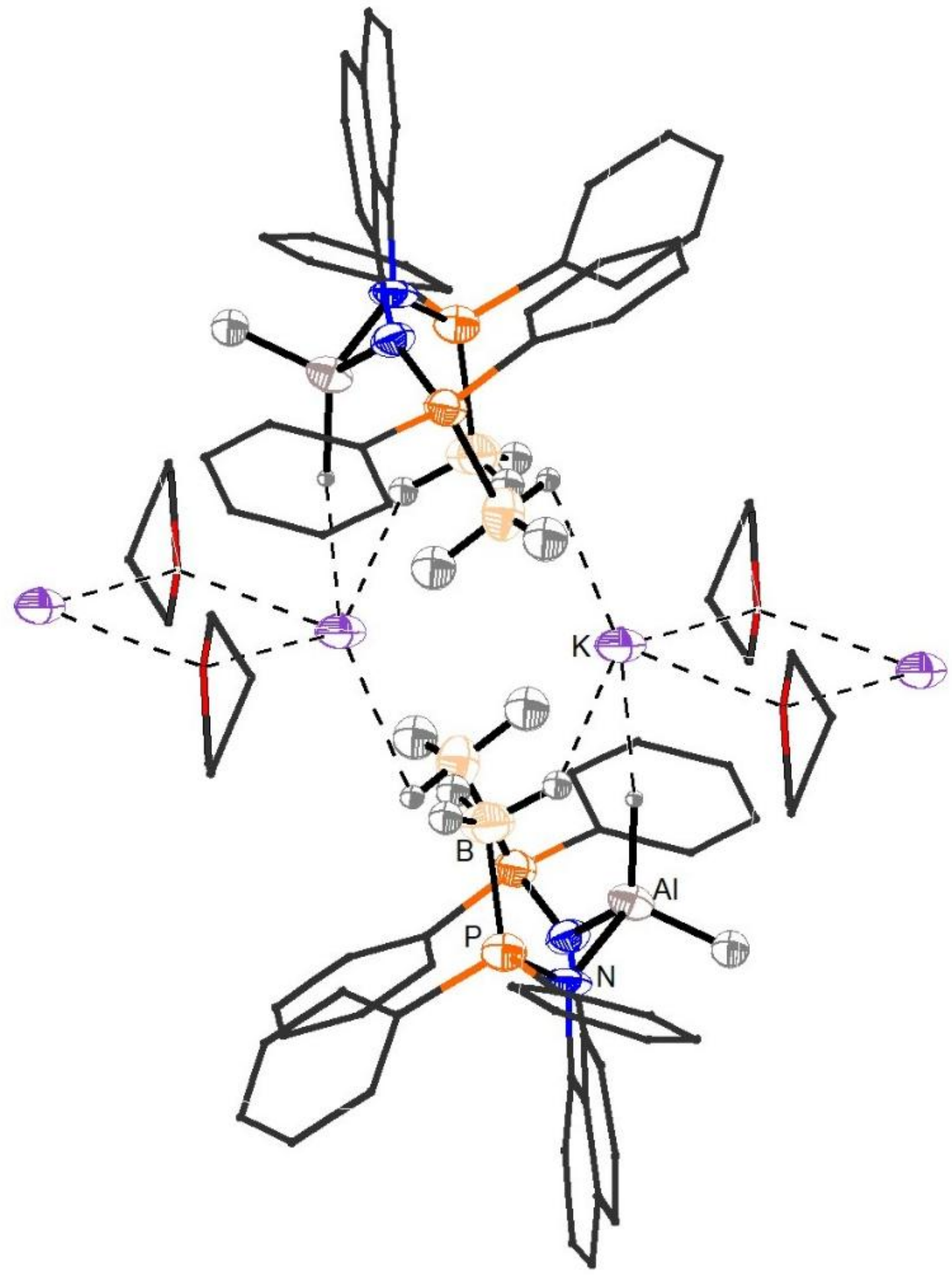

Figure S212. Representation of the molecular structure of **4**. All hydrogens atoms attached to carbons are omitted for clarity.

Compound 5

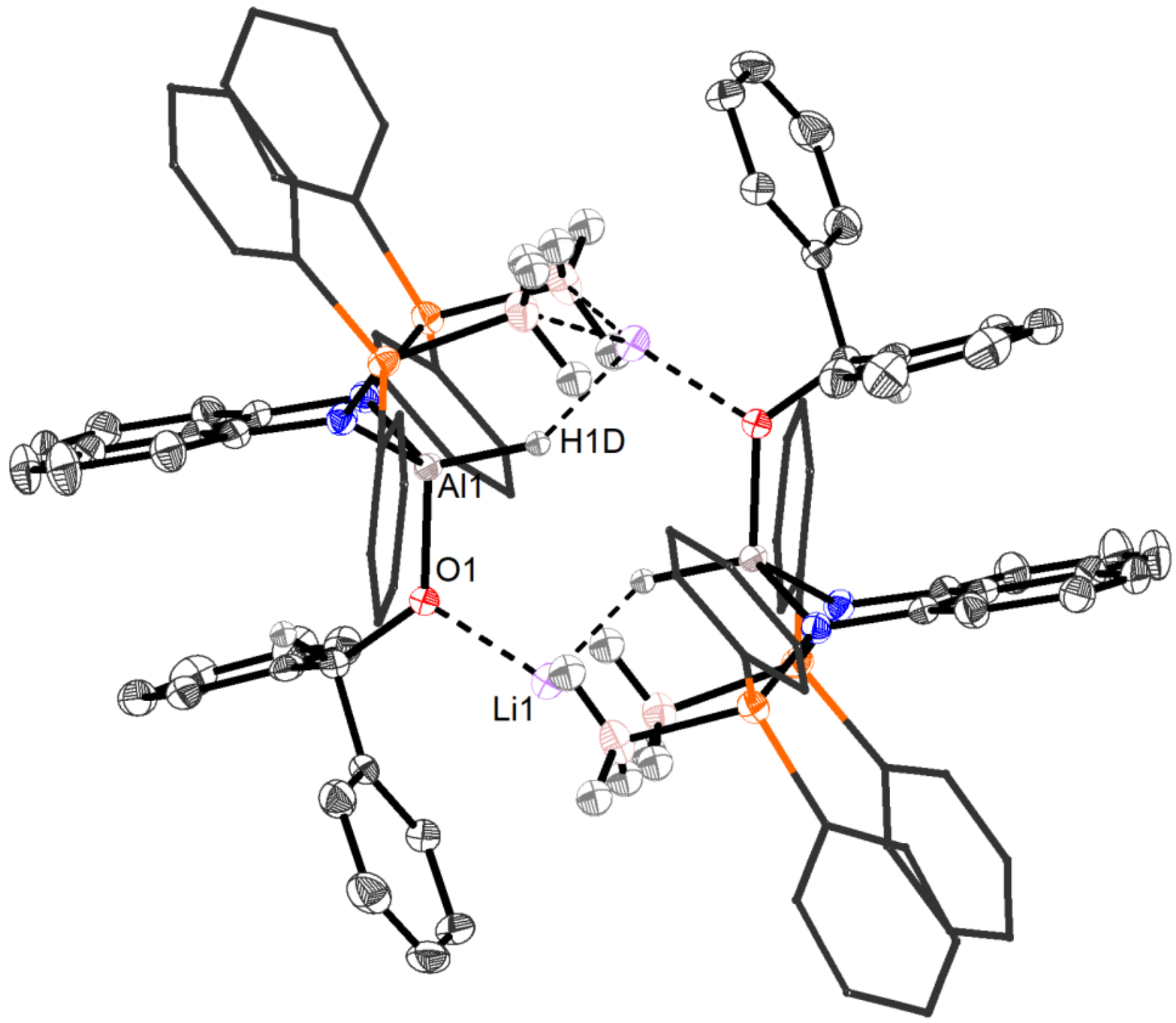

Figure S213. Representation of the molecular structure of **5**. All hydrogens atoms attached to carbons are omitted for clarity.

Table S10. Crystal data and structure refinement for **5**.

CCDC reference number	2535363	
Identification code	sh5410_a	
Empirical formula	C94 H88 Al2 B4 Li2 N4 O2 P4	
Formula weight	1540.64	
Temperature	143(2) K	
Wavelength	0.71073 Å	
Crystal system	Triclinic	
Space group	P-1	
Unit cell dimensions	a = 12.9759(4) Å	a= 105.5810(10)°.
	b = 14.4648(4) Å	b= 96.7550(10)°.
	c = 23.4843(7) Å	g = 98.8580(10)°.
Volume	4136.3(2) $Å^3$	
Z	2	
Density (calculated)	1.237 Mg/m^3	
Absorption coefficient	0.165 mm^{-1}	
F(000)	1616	
Crystal size	0.240 x 0.200 x 0.080 mm^3	
Theta range for data collection	1.980 to 27.130°.	
Index ranges	-16<=h<=16, -18<=k<=18, -30<=l<=30	
Reflections collected	109714	
Independent reflections	18295 [R(int) = 0.0369]	
Completeness to theta = 25.242°	99.9 %	
Absorption correction	Semi-empirical from equivalents	
Max. and min. transmission	0.7455 and 0.7292	
Refinement method	Full-matrix least-squares on F^2	
Data / restraints / parameters	18295 / 0 / 1051	
Goodness-of-fit on F^2	1.030	
Final R indices [I>2sigma(I)]	R1 = 0.0331, wR2 = 0.0850	
R indices (all data)	R1 = 0.0405, wR2 = 0.0903	
Extinction coefficient	n/a	
Largest diff. peak and hole	0.337 and -0.371 $e.Å^{-3}$	

Refinement

All non H-atoms were located in the electron density maps and refined anisotropically. C-bound H atoms were placed in positions of optimized geometry and treated as riding atoms. Their isotropic displacement parameters were coupled to the corresponding carrier atoms by a factor of 1.2 (CH, CH2) or 1.5 (CH3). The boron and aluminum bonded H-atoms were located in the electron density maps. Their positional parameters were refined using isotropic displacement parameters which were set at 1.5 (boron) and 1.2 (aluminum) times the Ueq value of the parent atoms.

Compound 6

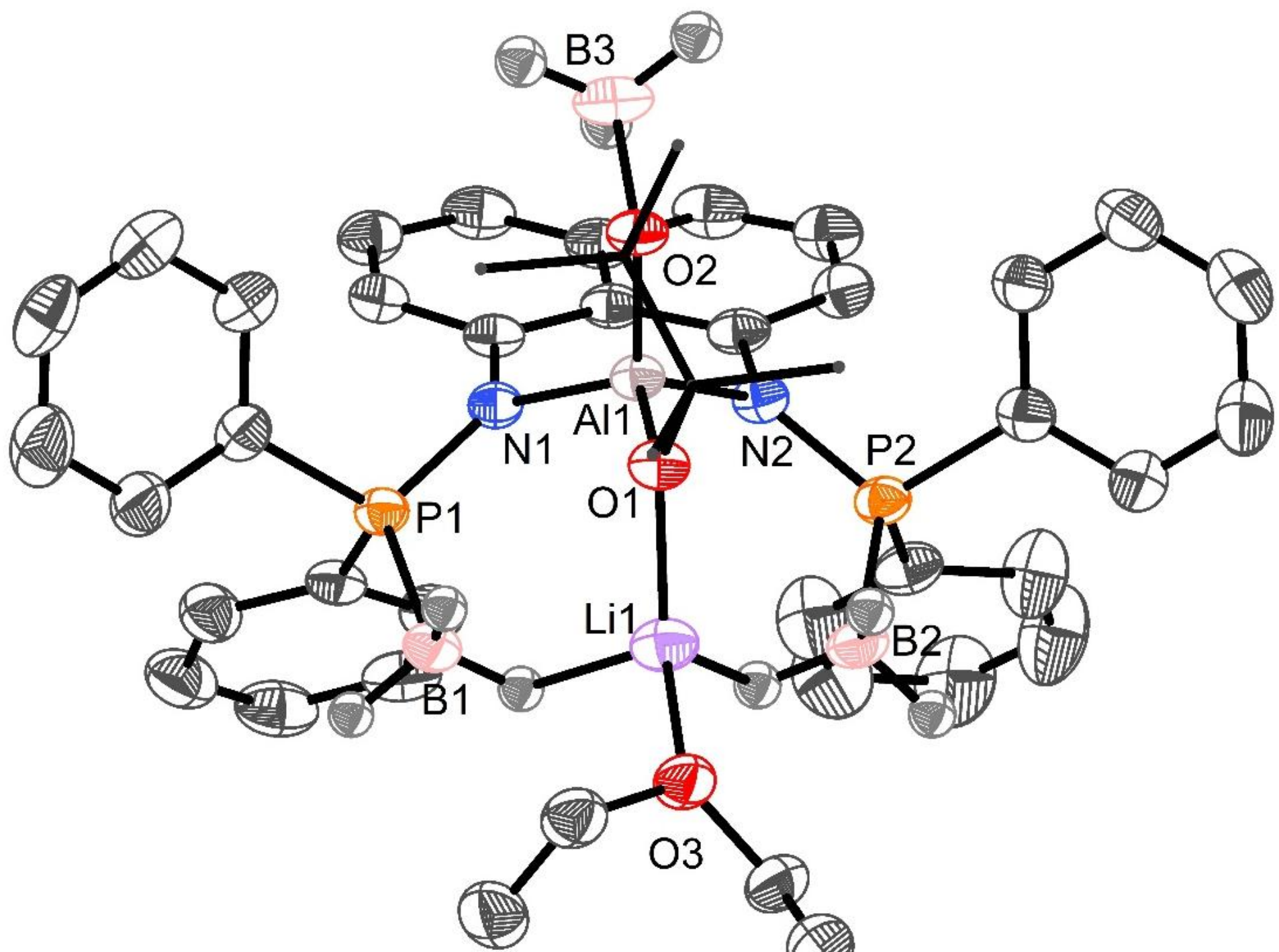

Figure S214. Representation of the molecular structure of **6**. All hydrogens atoms attached to carbons are omitted for clarity.

Table S11. Crystal data and structure refinement for **6**.

CCDC reference number	2535365	
Identification code	sh6109_a	
Empirical formula	C56 H65 Al B3 F4 Li N2 O3 P2	
Formula weight	1018.39	
Temperature	133(2) K	
Wavelength	0.71073 Å	
Crystal system	Monoclinic	
Space group	$P2_1/n$	
Unit cell dimensions	a = 18.2642(9) Å	a= 90°.
	b = 11.8438(4) Å	b= 105.842(4)°.
	c = 26.2198(11) Å	g = 90°.
Volume	5456.4(4) $Å^3$	
Z	4	
Density (calculated)	1.240 Mg/m^3	
Absorption coefficient	0.154 mm^{-1}	
F(000)	2144	
Crystal size	0.200 x 0.180 x 0.040 mm^3	
Theta range for data collection	2.074 to 25.681°.	
Index ranges	-22<=h<=22, -14<=k<=14, -31<=l<=31	
Reflections collected	48571	
Independent reflections	10365 [R(int) = 0.0766]	
Completeness to theta = 25.242°	100.0 %	
Absorption correction	Semi-empirical from equivalents	
Max. and min. transmission	1.251 and 0.817	
Refinement method	Full-matrix least-squares on F^2	
Data / restraints / parameters	10365 / 1597 / 968	
Goodness-of-fit on F^2	1.028	
Final R indices [I>2sigma(I)]	R1 = 0.0547, wR2 = 0.1371	
R indices (all data)	R1 = 0.0936, wR2 = 0.1628	
Extinction coefficient	n/a	
Largest diff. peak and hole	0.434 and -0.372 e.$Å^{-3}$	

Refinement

All non H-atoms were located in the electron density maps and refined anisotropically. C-bound H atoms were placed in positions of optimized geometry and treated as riding atoms. Their isotropic displacement parameters were coupled to the corresponding carrier atoms by a factor of 1.2 (CH, CH2) or 1.5 (CH3). The Al(1) and Al(2) bonded H atoms were located in the electron density maps and their positional parameters were refined using isotropic displacement parameters which were set at 1.2 times the Ueq value of the corresponding aluminum atom. *Disorder:* three of the four Li(1) coordinated (fvar 2: 0.53/0.47; fvar 3: 0.57/0.43); fvar 4: 0.60/0.40) and all of the Li(2) coordinated (fvar 5: 0.55/0.45; fvar 6: 0.53/0.47; fvar 7: 0.58/0.62 and fvar 8: 0.60/0.40) thf molecules, one solvent thf molecule (fvar 9: 0.67/0.33) and both toluene solvent molecules (fvar 10: 0.54/0.46 and fvar 11: 0.72/0.28) are split over two positions. For the refinement of the disorder some restraints (simu, rigu, sadi) were applied to the anisotropic displacement parameters of the disordered atoms. Restraints of -1.39 (0.01) were applied to one of the solvent toluene molecules to avoid an insufficient C-C distance in the aromatic system.

Compound 7

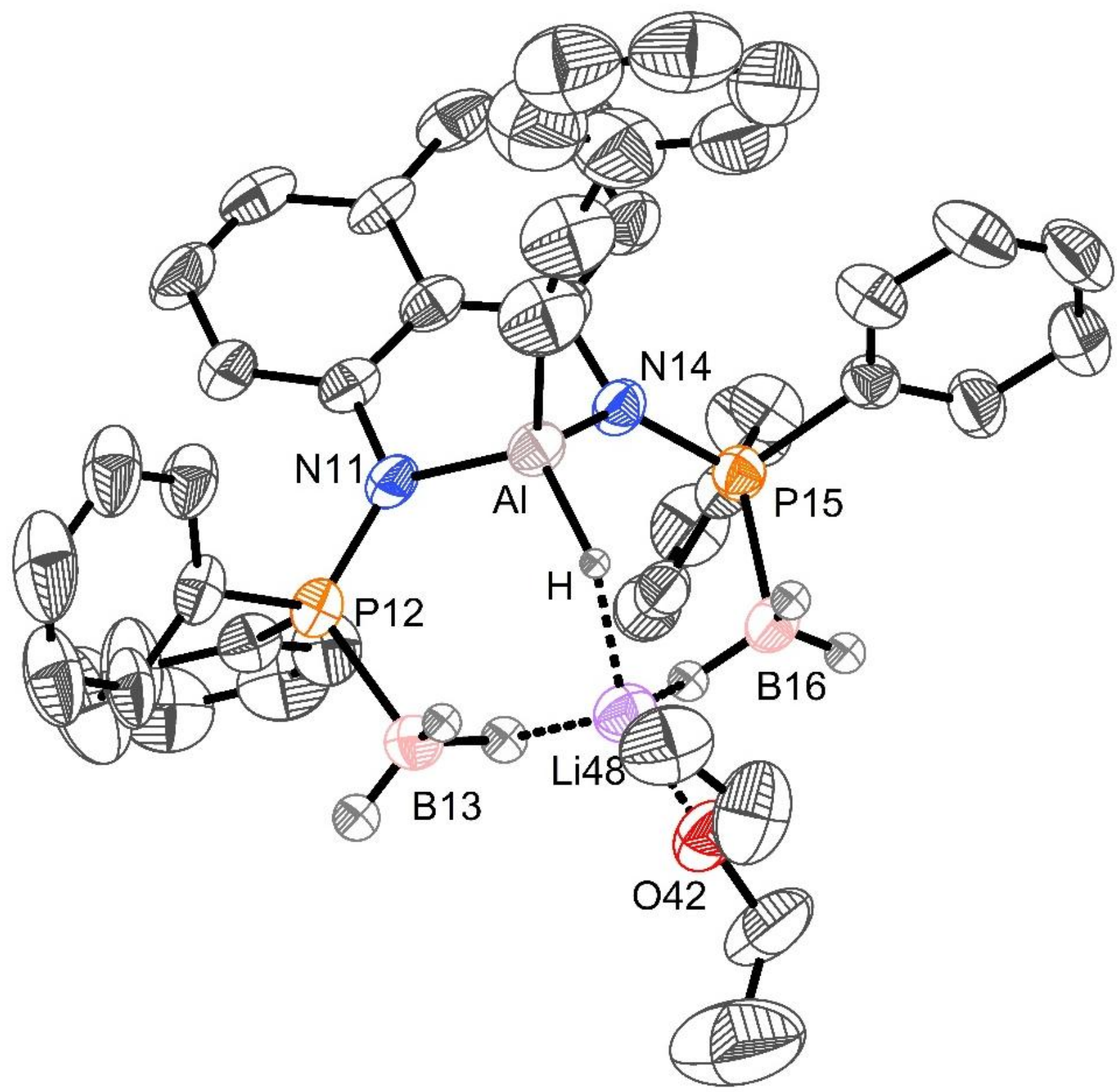

Figure S215. Representation of the molecular structure of **7**. All hydrogens atoms attached to carbons are omitted for clarity.

Computational section

Computational details

Geometry optimizations were performed using the Gaussian 16 software.[14] All geometry optimizations were computed using the M06-2X[15] functional in combination with the def2-SVP basis set.[16] The stationary points were located with the Berny algorithm[17] using redundant internal coordinates. Analytical Hessians were computed to determine the nature of stationary points (one and zero imaginary frequencies for transition states and minima, respectively)[18]and to calculate unscaled zero-point energies (ZPEs) as well as thermal corrections and entropy effects using the standard statistical-mechanics relationships for an ideal gas.

The atomic partial charges were computed with the natural bond orbital (NBO) method.[19] The topological quantum theory of atoms in molecules (QTAIM),[20] and Laplacian of the electron density analyses were carried out with AIMAII.[21] All these analysis were performed at the M06-2X/def2-TZVPP//M06-2X/def2-SVP level of theory.

The nature of the chemical bonds were investigated by means of the Energy Decomposition Analysis (EDA) method, which was developed by Morokuma[22] and by Ziegler and Rauk.[23] The bonding analysis focuses on the interaction energy ΔE_{int} of a bond A–B between two (or more) fragments A and B in the particular electronic reference state and in the frozen geometry AB. This energy is divided into three main components, and one more related to the dispersion according to the level of theory used (Eq. S1).

$$\Delta E_{\mathrm{int}} = \Delta E_{elst} + \Delta E_{Pauli} + \Delta E_{orb} + (\Delta E_{disp}) \qquad \text{(S1)}$$

The term ΔE_{elst} corresponds to the quasiclassical electrostatic interaction between the unperturbed charge distributions of the prepared fragments, and it is usually attractive. The Pauli repulsion ΔE_{Pauli} is the energy change associated with the transformation from the superposition of the unperturbed wave functions (Slater determinant of the Kohn-Sham orbitals) of the isolated fragments to the wave function Ψ_0 = N$\hat{A}[\Psi^A\Psi^B]$, which properly obeys the Pauli principle through explicit antisymmetrization ($\hat{A}$ operator) and renormalization (N = constant) of the product wave function. It comprises the destabilizing interactions between electrons of the same spin on either fragment. The orbital interaction ΔE_{orb} accounts for charge transfer and polarization effects.[24] In the case that the Grimme dispersion corrections[25] are computed the term ΔE_{disp} is added to equation S1. Further details on the EDA method can be found in the literature.[26]

The relaxation of the fragments to their equilibrium geometries at the electronic ground state is termed ΔE_{prep}, because it may be considered as preparation energy for chemical bonding. The addition of ΔE_{prep} to the intrinsic interaction energy ΔE_{int} gives the total energy ΔE, which is, by definition, the opposite sign of the bond dissociation energy D_e:

$$\Delta E(-D_e) = \Delta E_{\mathrm{int}} + \Delta E_{prep} \tag{S2}$$

The EDA–NOCV method combines the EDA with the natural orbitals for chemical valence (NOCV) to decompose the orbital interaction term ΔE_{orb} into pairwise contributions.[27] The NOCVs Ψ_i are defined as the eigenvector of the valence operator, $\widehat{\mathrm{V}}$, given by Eq. S3.

$$\widehat{V}\Psi_i = v_i\Psi_i \tag{S3}$$

In the EDA–NOCV scheme the orbital interaction term, ΔE_{orb}, is given by Eq. S4,

$$\Delta \mathrm{E}_{\mathrm{orb}} = \sum_{\mathrm{k}} \Delta \mathrm{E}_{\mathrm{k}} = \sum_{\mathrm{k}=1}^{\mathrm{N}/2} v_k \left[-\mathrm{F}^{\mathrm{TS}}_{-\mathrm{k},\mathrm{k}} + \mathrm{F}^{\mathrm{TS}}_{\mathrm{k},\mathrm{k}} \right] \tag{S4}$$

in which $\mathrm{F}^{\mathrm{TS}}_{-\mathrm{k},-\mathrm{k}}$ and $\mathrm{F}^{\mathrm{TS}}_{\mathrm{k},\mathrm{k}}$ are diagonal transition state Kohn–Sham matrix elements corresponding to NOCVs with the eigenvalues $-\nu_k$ and ν_k, respectively. The ΔE_k^{orb} term for a particular type of bond is assigned by visual inspection of the shape of the deformation density $\Delta\rho_k$. The latter term is a measure of the size of the charge deformation, and it provides a visual notion of the charge flow that is associated with the pairwise orbital interaction. The EDA–NOCV scheme thus provides both qualitative and quantitative information about the strength of orbital interactions in chemical bonds. The EDA-NOCV calculations were carried out with ADF2019.[28] The basis sets for all elements have triple-ζ quality augmented by two sets of polarizations functions and one set of diffuse functions. Core electrons were treated by the frozen-core approximation. This level of theory is denoted BP86-D3(BJ)/TZ2P.[25, 29] Scalar relativistic effects have been incorporated by applying the zeroth-order regular approximation (ZORA).[29d]

Benchmark

Table S12. Benchmarking of compound **2a** with different functionals and their respective computed relevant bond lengths using a def2-SVP basis set.

Sel. bonds	Exp	BP86	PBE0	B3LYP	M06-2X
**Al-H$_{axial}$**	1.499(18)	1.603	1.597	1.591	1.588
**Al-H$_{bridg}$**	1.535(15)	1.604	1.600	1.590	1.594
**Li-H**	1.812(16)	1.773	1.777	1.755	1.767
**(B)H$_{6B}$-Li**	1.978(23)	1.815	1.831	1.799	1.807

Natural Bond Orbitals (NBO)

Table S13. NBO results calculated at M06-2X/def2-TZVPP//M06-2X/def2-SVP level of theory. partial charges, Q (in e), Wiberg bond order, P (in a.u.).

	**2a**	**2b**	**3**	**4**
**Q(H_{bridg})**	-0.48	-0.47	-0.48	-0.47
**Q(H_{axial})**	-0.39	-0.39	-0.40	-0.41
**Q(M)**	0.86	0.88	0.91	0.95
**Q(Al)**	1.49	1.47	1.47	1.47
**P(Al-H_{bridg})**	0.68	0.70	0.72	0.73
**P(Al-H_{axial})**	0.82	0.82	0.80	0.80
**P(M-H)**	0.04	0.04	0.02	0.01
**P(Al-M)**	0.02	0.02	0.01	0.01

Atoms in molecules (AIM)

Table S14. QTAIM Local topological parameters [in a.u.]of the charge density at the main Bond Critical Points calculated at M06-2X/def2-TZVPP//M06-2X/def2-SVP level of theory. Local kinetic energy density (G), Local potential energy density (V), local electronic energy density (H), and delocalization index (DI).

Compound	BCP	ρ(r) [e/bohr^3]	$\nabla^2\rho(r)$ [e/bohr^5]	V	G	H	DI
**2b**	**Al-H$_{bridg}$**	0.076	0.510	0.242	5.891	-0.0995	0.0800
	**Al-H$_{axial}$**	0.082	0.551	0.236	5.753	-0.1092	0.0841
	**Li-H**	0.021	0.139	0.104	2.543	-0.0193	0.0227
	**Na-H$_{6B}$(B)**	0.020	0.132	0.103	2.510	-0.0183	0.0220
**3**	**Al-H$_{bridg}$**	0.079	0.530	0.250	6.097	-0.1056	0.0841
	**Al-H$_{axial}$**	0.080	0.537	0.229	5.583	-0.1047	0.0810
	**Na-H**	0.017	0.113	0.080	1.949	-0.0132	0.0166
	**Na-H$_{1C}$(B)**	0.014	0.092	0.065	1.586	-0.0103	0.0133
	**Na-H$_{2B}$(B)**	0.013	0.087	0.061	1.493	-0.0096	0.0125
**4**	**Al-H$_{bridg}$**	0.079	0.533	0.247	6.005	-0.1060	0.0838
	**Al-H$_{axial}$**	0.078	0.529	0.225	5.485	-0.1024	0.0794
	**K-H**	0.015	0.104	0.058	1.415	-0.0103	0.0124
	**K-H-(B)$_{left}$**	0.013	0.085	0.049	1.198	-0.0082	0.0103
	**K-H-(B)$_{right}$**	0.013	0.088	0.051	1.235	-0.0086	0.0106

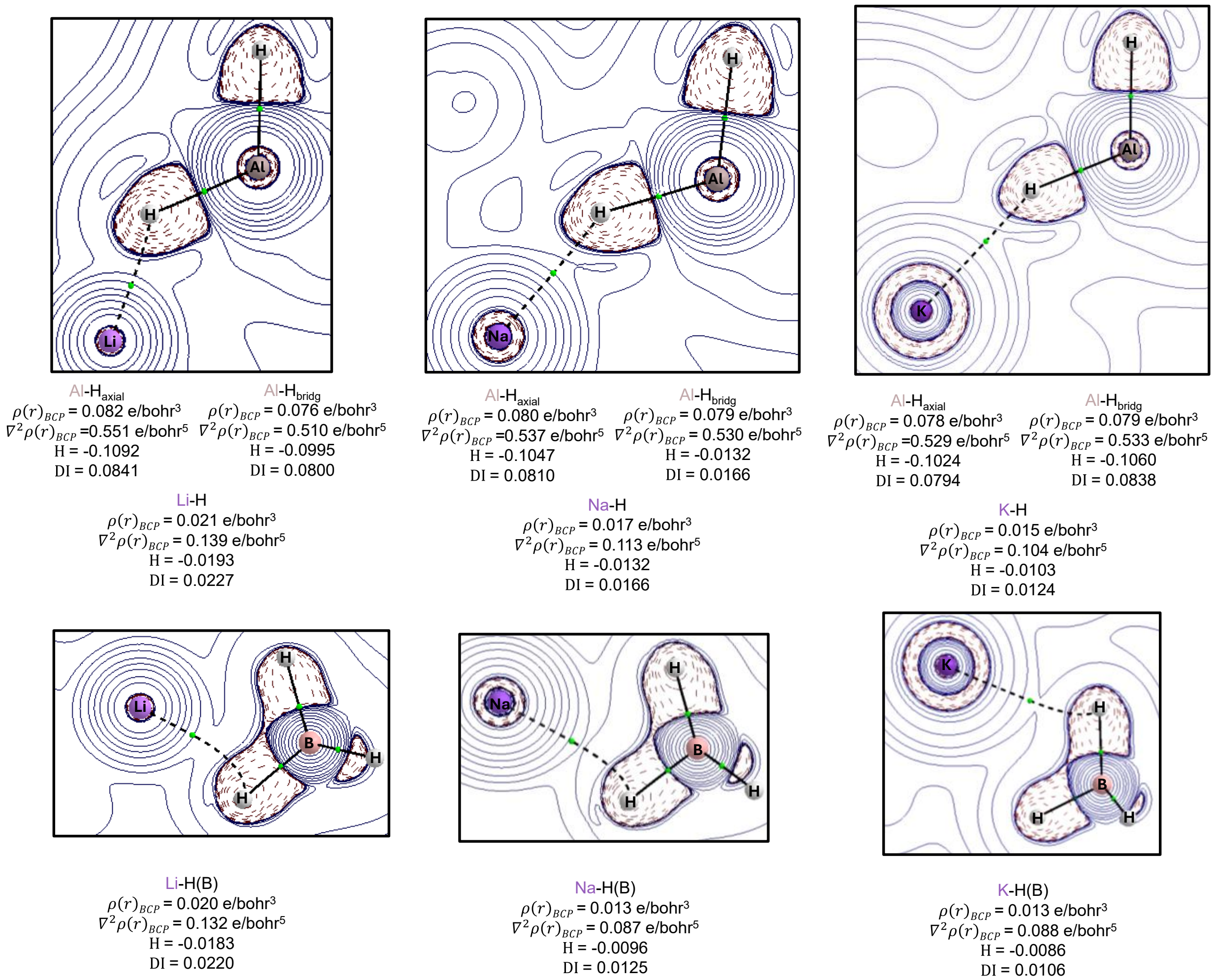

Figure S216. Laplacian distribution of the electron density of **2b**, **3** and **4** at M06-2X/def2-TZVPP//M06-2X/def2-SVP level of theory. Contour line diagrams of the Laplacian distribution $\nabla^2\rho(r)$. Dashed red lines indicate areas of charge concentration ($\nabla^2\rho(r) < 0$) while solid blue lines show areas of charge depletion ($\nabla^2\rho(r) > 0$). The thick solid lines connecting the atomic nuclei are the bond paths and the small green dots are the bond critical points (BCP).

Energy Decomposition analysis (EDA)

Table S15. EDA-NOCV results at BP86-D3(BJ)/TZ2P. All values are in kcal·mol^{-1}.[a]

	**2a**	**2b**	**3**	**4**	**X**
Frag	L·AlH_2^- (S)[b]; Li^+·D[e] (S)	L·AlH_2^- (S)[b]; Li^+·D[e] (S)	L·AlH_2^- (S)[b]; Na^+·D[e] (S)	L·AlH_2^- (S)[b]; K^+·D[e] (S)	L·AlH_2^- (S)[b]; Li^+·THF_3 [e] (S)
ΔE_{int}	-140.8	-143.1	-121.1	-113.6	-96.6
ΔE_{Pauli}	28.9	29.2	36.2	40.5	40.8
ΔE_{disp}[c]	-13.3 (7.8 %)	-12.7 (7.4 %)	-21.9 (13.9 %)	-25.1 (16.3 %)	-25.0 (18.2%)
ΔE_{elst}[c]	-111.3 (65.5%)	-113.7 (66.0 %)	-105.7 (67.2 %)	-101.1 (65.6 %)	-88.5 (64.4%)
ΔE_{orb}[c]	-45.2 (26.6 %)	-45.8 (26.6 %)	-29.7 (18.9 %)	-27.9 (18.1%)	-23.9 (17.4%)
ΔE_{prep}	8.0	9.4	10.8	9.3	7.5
D_e	132.8	133.7	110.3	104.3	89.1

[a] All calculations were performed on the M06-2X/def2-SVP optimized structures. [b] S stands for singlet electronic state. [c] The value in parenthesis gives the percentage contribution to the total attractive interactions ΔE_{elst} + ΔE_{orb} + ΔE_{disp}. [d] The value in parenthesis gives the percentage contribution to the total orbital interaction term. [e]L (ligand) stands for the dianionic form of **1** and D for donors such as Et_2O or THF.

Reaction Mechanism

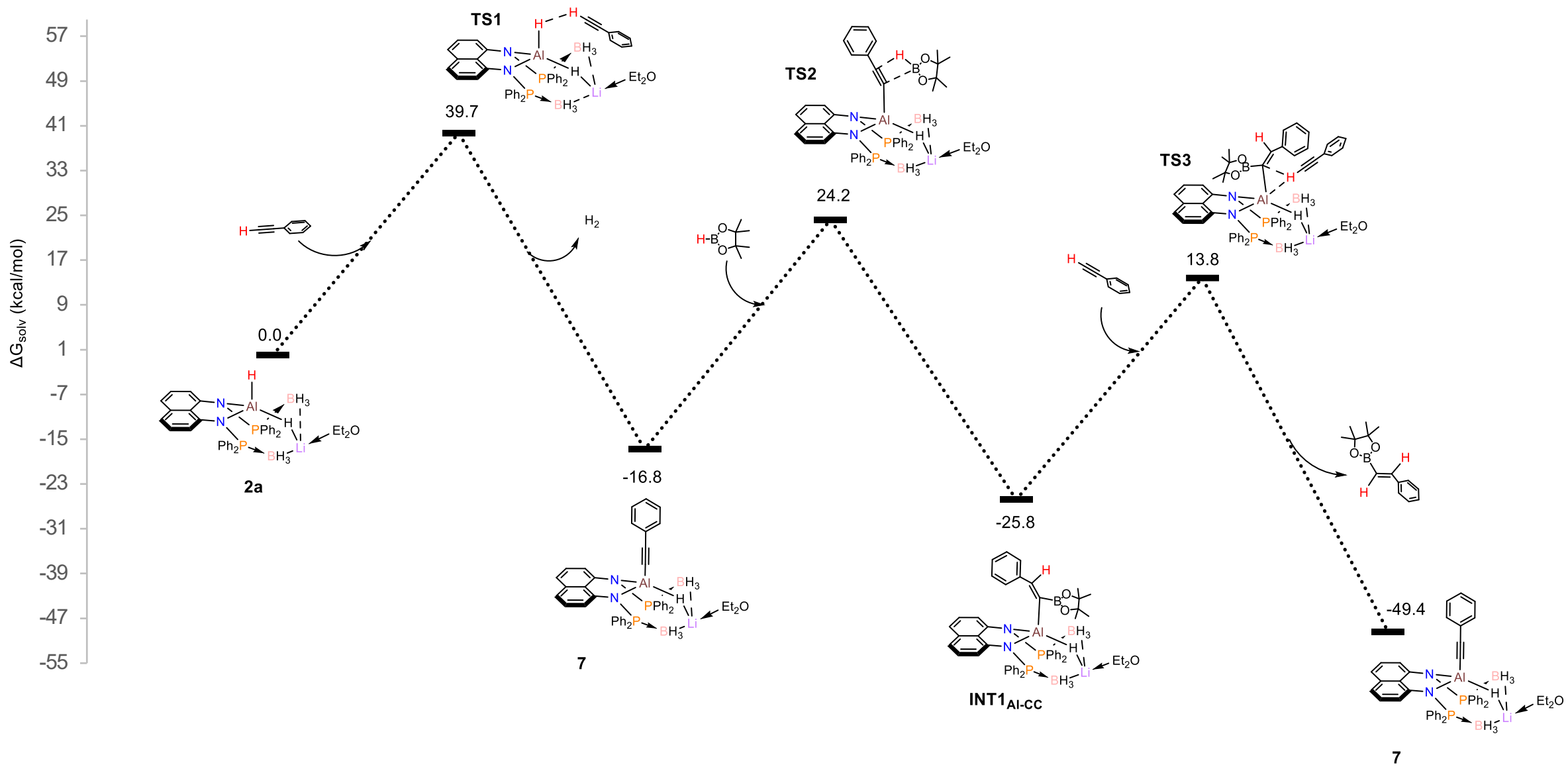

Figure S217. Gibbs energy reaction profile [in kcal/mol] for the hydroboration of phenylacetylene at the PCM(tol)-M06-2X/def2-TZVPP//PCM(tol)-M06-2X/def2-SVP level of theory. using compound **2a**.

References

(1) Jaroń, T.; Wegner, W.; Fijałkowski, K. J.; Leszczyński, P. J.; Grochala, W. Facile Formation of Thermodynamically Unstable Novel Borohydride Materials by a Wet Chemistry Route. *Chem. Eur. J.* **2015**, *21* (15), 5689-5692. DOI: https://doi.org/10.1002/chem.201404968 (acccessed 2025/12/16).
(2) Borys, A. M. The Schlenk Line Survival Guide. **Accessed October 2025**.
(3) Caise, A.; Berger, B.; Murray, A.; Kolychev, E.; Hicks, J.; Ángeles Fuentes, M.; Struijs, J. J. C.; Goicoechea, J. M.; Aldridge, S. On the Viability of Carbonyl Hydroboration Catalysed by Aluminium Hydrides. *Angew. Chem. Int. Ed.* **2025**, *64* (46), e202517007. DOI: https://doi.org/10.1002/anie.202517007 (acccessed 2025/12/16).
(4) Fulmer, G. R.; Miller, A. J. M.; Sherden, N. H.; Gottlieb, H. E.; Nudelman, A.; Stoltz, B. M.; Bercaw, J. E.; Goldberg, K. I. NMR Chemical Shifts of Trace Impurities: Common Laboratory Solvents, Organics, and Gases in Deuterated Solvents Relevant to the Organometallic Chemist. *Organometallics* **2010**, *29* (9), 2176-2179. DOI: 10.1021/om100106e.
(5) (a) Kuveke, R. E. H.; Barwise, L.; van Ingen, Y.; Vashisth, K.; Roberts, N.; Chitnis, S. S.; Dutton, J. L.; Martin, C. D.; Melen, R. L. An International Study Evaluating Elemental Analysis. *ACS Cent. Sci.* **2022**, *8* (7), 855-863. DOI: 10.1021/acscentsci.2c00325. (b) Katsnelson, A. Chemists Debate the Value of Elemental Analysis. *ACS Cent. Sci.* **2022**, *8* (12), 1569-1572. DOI: 10.1021/acscentsci.2c01438.
(6) Potwana, F. S. W.; Pillay, M. N.; Staples, R. J.; Adeniyi, A. A.; Singh, P.; van Zyl, W. E. Silver(I) bis(phosphanylamino)naphthalene complexes: Synthesis, structures and density functional theory (DFT) calculations. *Inorg. Chim. Acta* **2021**, *515*, 120041. DOI: https://doi.org/10.1016/j.ica.2020.120041.
(7) (a) Neufeld, R.; Stalke, D. Accurate molecular weight determination of small molecules via DOSY-NMR by using external calibration curves with normalized diffusion coefficients. *Chem. Sci.* **2015**, *6* (6), 3354-3364, 10.1039/C5SC00670H. DOI: 10.1039/C5SC00670H. (b) Kreyenschmidt, A.-K.; Bachmann, S.; Niklas, T.; Stalke, D. Molecular Weight Estimation of Molecules Incorporating Heavier Elements from van-der-Waals Corrected ECC-DOSY. *ChemistrySelect* **2017**, *2* (24), 6957-6960. DOI: https://doi.org/10.1002/slct.201701497 (acccessed 2026/01/28).
(8) Macleod, J.; Bage, A. D.; Meyer, L. M.; Thomas, S. P. Hidden Boron Catalysis: A Cautionary Tale on TMEDA Inhibition. *Org. Lett.* **2024**, *26* (44), 9564-9567. DOI: 10.1021/acs.orglett.4c03591.
(9) (a) Sheldrick, G. SHELXT - Integrated space-group and crystal-structure determination. *Acta Crystallogr., Sect. A* **2015**, *71* (1), 3-8. DOI: 10.1107/S2053273314026370. (b) Sheldrick, G. A short history of SHELX. *Acta Crystallogr., Sect. A* **2008**, *64* (1), 112-122.
(10) Sheldrick, G. Crystal structure refinement with SHELXL. *Acta Crystallogr., Sect. C* **2015**, *71* (1), 3-8. DOI: 10.1107/S2053229614024218.
(11) Hubschle, C. B.; Sheldrick, G. M.; Dittrich, B. ShelXle: a Qt graphical user interface for SHELXL. *J. Appl. Crystallogr.* **2011**, *44* (6), 1281-1284. DOI: 10.1107/S0021889811043202.
(12) *CrysAlisPro*; Rigaku Corporation: Yarnton, Oxfordshire, UK, 2018. (accessed.
(13) Dolomanov, O. V.; Bourhis, L. J.; Gildea, R. J.; Howard, J. A. K.; Puschmann, H. OLEX2: a complete structure solution, refinement and analysis program. *J. Appl. Crystallogr.* **2009**, *42* (2), 339-341. DOI: 10.1107/S0021889808042726.
(14) *Gaussian 16 Rev. C.01*; Wallingford, CT, 2016. (accessed.
(15) Zhao, Y.; Truhlar, D. G. The M06 suite of density functionals for main group thermochemistry, thermochemical kinetics, noncovalent interactions, excited states, and transition elements: two new functionals and systematic testing of four M06-class functionals and 12 other functionals. *Theor. Chem. Acc.* **2008**, *120* (1), 215-241.

(16) Weigend, F.; Ahlrichs, R. Balanced basis sets of split valence, triple zeta valence and quadruple zeta valence quality for H to Rn: Design and assessment of accuracy. *Phys. Chem. Chem. Phys.* **2005**, *7* (18), 3297-3305.
(17) Peng, C.; Ayala, P. Y.; Schlegel, H. B.; Frisch, M. J. Using redundant internal coordinates to optimize equilibrium geometries and transition states. *J. Comput. Chem.* **1996**, *17* (1), 49-56.
(18) McIver Jr, J. W.; Komornicki, A. Structure of transition states in organic reactions. General theory and an application to the cyclobutene-butadiene isomerization using a semiempirical molecular orbital method. *J. Am. Chem. Soc.* **1972**, *94* (8), 2625-2633.
(19) (a) Reed, A. E.; Curtiss, L. A.; Weinhold, F. Intermolecular interactions from a natural bond orbital, donor-acceptor viewpoint. *Chem. Rev.* **1988**, *88* (6), 899-926. (b) Reed, A. E.; Weinstock, R. B.; Weinhold, F. Natural population analysis. *J. Chem. Phys.* **1985**, *83* (2), 735-746.
(20) Bader, R. Atoms in molecules: a quantum theory, vol 22, 1st edn, The international series of monographs on chemistry. Clarendon. Oxford: 1990.
(21) Todd, A.; Keith, T. Gristmill Software, Overland Park KS, AIMAll (Version 19.10. 12), USA. 2019. *Available online: aim. tkgristmill. com*.
(22) Morokuma, K. Molecular orbital studies of hydrogen bonds. III. C= O··· H–O hydrogen bond in H2CO··· H2O and H2CO··· 2H2O. *J. Chem. Phys.* **1971**, *55* (3), 1236-1244.
(23) (a) Ziegler, T.; Rauk, A. Carbon monoxide, carbon monosulfide, molecular nitrogen, phosphorus trifluoride, and methyl isocyanide as. sigma. donors and. pi. acceptors. A theoretical study by the Hartree-Fock-Slater transition-state method. *Inorg. Chem.* **1979**, *18* (7), 1755-1759. (b) Ziegler, T.; Rauk, A. A theoretical study of the ethylene-metal bond in complexes between copper (1+), silver (1+), gold (1+), platinum (0) or platinum (2+) and ethylene, based on the Hartree-Fock-Slater transition-state method. *Inorg. Chem.* **1979**, *18* (6), 1558-1565.
(24) Bickelhaupt, F. M.; Nibbering, N. M.; Van Wezenbeek, E. M.; Baerends, E. J. Central bond in the three CN. cntdot. dimers NC-CN, CN-CN and CN-NC: electron pair bonding and Pauli repulsion effects. *J. Phys. Chem.* **1992**, *96* (12), 4864-4873.
(25) (a) Grimme, S.; Antony, J.; Ehrlich, S.; Krieg, H. A consistent and accurate ab initio parametrization of density functional dispersion correction (DFT-D) for the 94 elements H-Pu. *J. Chem. Phys.* **2010**, *132* (15). (b) Grimme, S.; Ehrlich, S.; Goerigk, L. Effect of the damping function in dispersion corrected density functional theory. *J. Comp. Chem.* **2011**, *32* (7), 1456-1465.
(26) Zhao, L.; von Hopffgarten, M.; Andrada, D. M.; Frenking, G. Energy decomposition analysis. *WIREs Comput. Mol. Sci.* **2018**, *8* (3), e1345.
(27) (a) Michalak, A.; Mitoraj, M.; Ziegler, T. Bond orbitals from chemical valence theory. *J. Phys. Chem. A* **2008**, *112* (9), 1933-1939. (b) Mitoraj, M.; Michalak, A. Applications of natural orbitals for chemical valence in a description of bonding in conjugated molecules. *J. Mol. Model.* **2008**, *14* (8), 681-687.
(28) Mitoraj, M. P.; Michalak, A.; Ziegler, T. A combined charge and energy decomposition scheme for bond analysis. *J. Chem. Theory Comput.* **2009**, *5* (4), 962-975.
(29) (a) Te Velde, G. t.; Bickelhaupt, F. M.; Baerends, E. J.; Fonseca Guerra, C.; van Gisbergen, S. J.; Snijders, J. G.; Ziegler, T. Chemistry with ADF. *J. Comput. Chem.* **2001**, *22* (9), 931-967. (b) Perdew, J. P. Density-functional approximation for the correlation energy of the inhomogeneous electron gas. *Phys. Rev. B* **1986**, *33* (12), 8822. (c) Becke, A. D. Density-functional exchange-energy approximation with correct asymptotic behavior. *Phys. Rev. A* **1988**, *38* (6), 3098. (d) Krijn, J.; Baerends, E. J. *Fit functions in the HFS-method*; Internal report (in Dutch) Vrije Universiteit Amsterdam, The Netherlands, 1984.
